# Photoinduced Remote Functionalisations by Iminyl Radical Promoted C−C and C−H Bond Cleavage Cascades

**DOI:** 10.1002/anie.201710790

**Published:** 2017-12-04

**Authors:** Elizabeth M. Dauncey, Sara P. Morcillo, James J. Douglas, Nadeem S. Sheikh, Daniele Leonori

**Affiliations:** ^1^ School of Chemistry University of Manchester Oxford Road Manchester M13 9PL UK; ^2^ Early Chemical Development, Pharmaceutical Sciences, IMED Biotech Unit AstraZeneca Macclesfield SK10 2NA UK; ^3^ Department of Chemistry Faculty of Science King Faisal University P.O. Box 380 Al-Ahsa 31982 Saudi Arabia

**Keywords:** azidation, cascade reactions, chlorination, fluorination, photoredox catalysis

## Abstract

A photoinduced cascade strategy leading to a variety of differentially functionalised nitriles and ketones has been developed. These reactions rely on the oxidative generation of iminyl radicals from simple oximes. Radical transposition by C(sp^3^)−(sp^3^) and C(sp^3^)−H bond cleavage gives access to distal carbon radicals that undergo S_H_2 functionalisations. These mild, visible‐light‐mediated procedures can be used for remote fluorination, chlorination, and azidation, and were applied to the modification of bioactive and structurally complex molecules.

The development of methods for the selective functionalisation of non‐activated sp^3^ carbon atoms is a longstanding endeavour in organic synthesis.^1^ Radical strategies are attractive options owing to the ability of odd‐electron species to undergo transposition processes.^2^ If a suitably reactive radical is generated in a specific position of an organic molecule, then C(sp^3^)−C(sp^3^) and C(sp^3^)−H bond cleavage can be triggered, leading to remote functionalisations. Classical examples are the ring opening of α‐cyclopropyl radicals (radical clock)^3^ and the 1,5‐hydrogen atom abstraction of nitrogen radicals—the key step of the Hofmann–Löffler–Freytag (HLF) reaction^4^ (Scheme 1 A).^5^

**Scheme 1:**
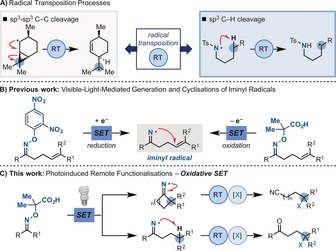
Radical transposition processes and our current work on using iminyl radicals.

We have recently developed two classes of oximes that provide access to iminyl radicals by both reductive and oxidative visible‐light‐mediated single electron transfer (SET) and employed them in intramolecular cyclisation processes (Scheme 1 B).^6^ Whereas amidyl and *N*‐tosyl aminyl radicals have been extensively exploited in visible‐light‐mediated transposition reactions,^5^, ^7^ the implementation of iminyl radicals has been considerably overlooked.^8^ Inspired by the pioneering work of Zard8e–8g and Forrester,8a–8c we wondered whether the key transposition steps of the radical clock and HLF processes could be harnessed as part of a general interrupted cascade strategy that leads to a variety of functionalised molecules from an iminyl radical. Herein, we report the development of two photoinduced manifolds that enable the preparation of remotely functionalised nitriles and ketones in a cascade process comprising iminyl radical generation, C(sp^3^)−C(sp^3^) and C(sp^3^)−H bond cleavage, and functionalisation (Scheme 1 C).

At the outset, we realised that our reductive SET approach for iminyl radical generation and cyclisation was electronically mismatched as illustrated in Scheme 2 using a ring‐opening/functionalisation process. In fact, upon SET reduction and fragmentation of precursor **A**, the iminyl radical **B** should undergo a fast β‐fission generating the δ‐CN radical **C**. As this species is expected to be nucleophilic, it benefits from the reaction with polarised SOMOphiles (X−Y) where the X atom/group has a partially positive character. Following radical atom/group transfer (S_H_2), **D** is generated together with the radical Y^.^. Owing to its electrophilic nature, a final SET oxidation will be highly endergonic, thus thwarting the development of a redox‐neutral process. We therefore selected the carboxylic acid containing oxime **E** as the starting point. We were hopeful that upon deprotonation, a sequence of SET oxidation and CO_2_ and acetone extrusion would lead to the formation of **B**. Following β‐fission/S_H_2 cascade would generate the product **D** and the electrophilic radical Y^.^, which should undergo exergonic SET reduction, ensuring efficient reactivity.

**Scheme 2:**
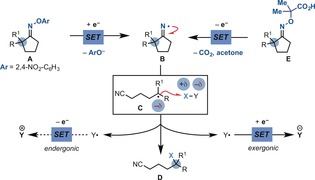
Mechanistic analysis of iminyl radical transposition/functionalisation.

We started our investigations by preparing oxime **1 a** by condensation of 2,2‐dimethylcyclopentanone with a commercially available hydroxylamine on gram scale (Scheme 3 A).^9^ Using Fukuzumi's acridinium salt **2** as the photoredox catalyst (*E_1/2_=2.2 V vs. SCE),6b, 10 we evaluated several potential radical fluorinating agents (X−Y),^11^ bases, and solvents under blue LED irradiation. While the use of *N*‐fluorobenzenesulfonimide (NFSI) gave unreacted **1 a** (entry 1), we were pleased to see that with Selectfluor, the desired iminyl radical generation/ring opening/fluorination cascade proceeded, albeit in low yield (entry 2). However, by using H_2_O as the co‐solvent (entry 3) and K_2_CO_3_ as the base (entry 4), **3 a** was obtained in high yield in just 15 min. This represents a radical “abnormal” Beckmann fragmentation/fluorination process.^12^ Control experiments confirmed the requirement for base, **2**, and continuous blue‐light irradiation. A quantum yield^13^ of *Φ*=2.8 was determined for this transformation, which suggests that productive short‐lived radical chain propagation processes are operating together with the photoredox pathway.^9^

**Scheme 3:**
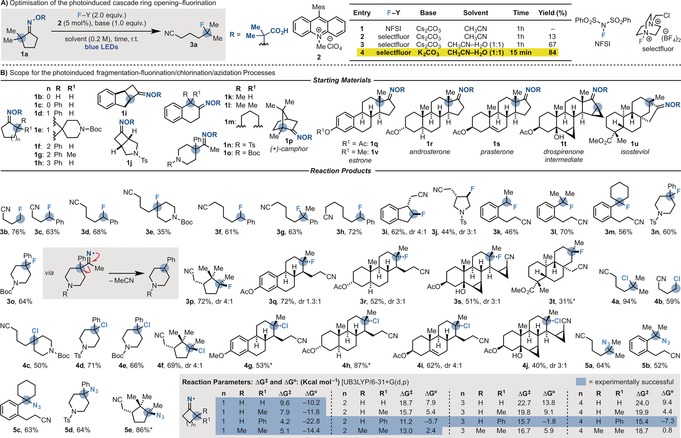
Development and scope of the photoinduced ring opening/fluorination, chlorination, and azidation cascade reactions via iminyl radicals. *: isolated as a single diastereomer.

With an optimised set of conditions in hand, the scope of the reaction was evaluated (Scheme 3 B). We achieved the oxidation/ring opening/fluorination of oximes derived from cyclobutanones (**1 b**, **c**→**3 b**, **c** and **1 i**, **j**→**3 i**, **j**), cyclopentanones (**1 d**, **e**→**3 d**, **e**), cyclohexanones (**1 f**, **g**→**3 f**, **g** and **1 k**–**m**→**3 k**–**m**), and, remarkably, cycloheptanones (**1 h**→**3 h**), obtaining a broad range of fluorinated products in good yields. This method was also implemented as part of a strategy for the preparation of 4‐fluorinated 4‐aryl piperidines (**1 n**, **o**→**3 n**, **o**), which constitute the structural core of many opioid analgesics and antipsychotics such as pethidine and haloperidol. In this case, upon iminyl radical generation, the fragmentation process expels acetonitrile.

We were particularly interested in applying this strategy to the structural modification of natural products. As these unique architectures are tuned to allow for optimal interactions with specific biological targets, a method providing access to compounds with natural‐product‐like cores has great promise in phenotype‐based drug discovery.^14^ Pleasingly, we were able to deconstruct and fluorinate (+)‐camphor (**1 p**→**3 p**), the steroids estrone (**1 q**→**3 q**) and androsterone (**1 r**→**3 r**), a synthetic intermediate of the oral contraceptive drospirenone (**1 t**→**3 s**), and the diterpene isosteviol (**1 u**→**3 t**).

Furthermore, *N*‐chlorosuccinimide (NCS) and triisopropylsulfonyl azide^15^ were effective among a range of reagents investigated to achieve a novel cascade approach towards chlorinated (**4 a**–**j**) and azidated (**5 a**–**e**) nitriles.^9^ Here, the optimum base and solvent were identified as KOAc/CH_3_CN and Cs_2_CO_3_/CH_2_Cl_2_ for the chlorination and azidation processes, respectively.

Based on a combination of density functional theory (DFT) and experimental studies, we determined that while cyclobutanone‐derived oximes undergo very facile fragmentation, the radical ring opening of larger cycles is considerably more difficult. However, by introducing either an α‐aromatic substituent or two α‐alkyl groups (e.g., gem‐Me_2_), we were able to effectively engage cyclopentanone derivatives. In the case of cyclohexanone‐ and cycloheptanone‐derived oximes, the radical fragmentations became increasingly challenging, but could be achieved in good yields when aromatic α‐substituents were present.^9^

Having achieved tandem iminyl radical generation and C(sp^3^)−C(sp^3^) bond fragmentation/functionalisation, we evaluated the development of a strategy based on C(sp^3^)−H bond cleavage. Success here would deliver a method for the selective γ‐functionalisation of ketones^16^ via iminyl radicals, which is currently underdeveloped. Specifically, we envisaged a cascade process starting with the oxidative SET fragmentation of precursor **F** leading to iminyl **G**. In this case, as C−C bond fragmentation is not favourable, a 1,5‐hydrogen abstraction2d would deliver the nucleophilic γ‐carbon radical **H** (Scheme 4 A). A polarity‐matched S_H_2 reaction with a SOMOphile (to give **I**) and hydrolysis would provide the γ‐functionalised ketone **J**, which would be unreactive under classical ionic reaction conditions. We were, however, concerned by the fact that 1) 1,5‐hydrogen abstractions involving iminyl radicals are known to require acidic conditions,8b,8c which would not allow for decarboxylation in our system, and that 2) radical **H** is prone to undergo intramolecular cyclisation when R=aromatic as reported by Forrester and Nevado.8b,8c,8m

**Scheme 4:**
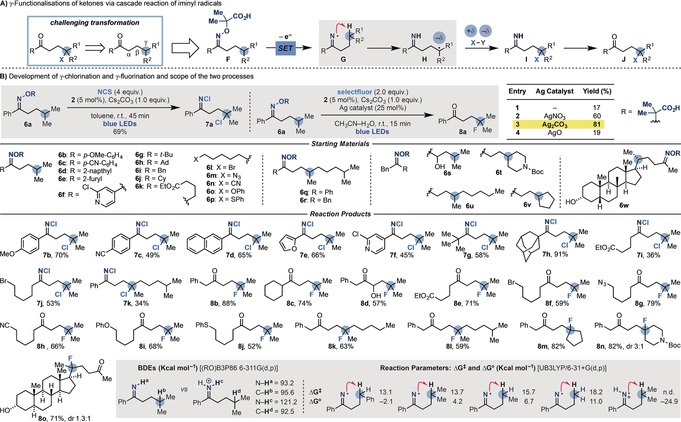
Development and scope of the photoinduced γ‐chlorination and γ‐fluorination reactions via iminyl radicals.

Despite these concerns, we started our investigations by preparing oxime **6 a** and exposing it to various SOMOphiles with **2** as the photocatalyst under blue LED irradiation (Scheme 4 B).^9^ We were pleased to see that in the presence of NCS, γ‐chlorination occurred smoothly.^17^ However, upon isolation and full characterisation of the reaction product, we realised that the imine intermediate **I** had reacted with the excess NCS to provide the N−Cl imine **7 a**, which was stable and could be purified by column chromatography.^18^ We then hoped to expand this strategy and develop a sought‐after γ‐fluorination process. We were particularly interested in the fact that going via an iminyl radical would bypass some of the limitations of approaches based on the photoexcitation of ketones, which are mostly suited for the fluorination of structurally congested molecules as elegantly demonstrated by Lectka.^19^ In this case, however, a screen of several radical F donors, bases, photocatalysts, and solvents led to very low product formation (entry 1). Pleasingly, by using Selectfluor as the F source and Ag_2_CO_3_ as a co‐catalyst,^20^ γ‐fluorinated ketone **8 a** was obtained in high yield in just 15 min (entry 3). Control experiments confirmed the necessity for **2**, Ag^I^, and continuous blue LED irradiation. For this reaction, the calculated quantum yield *Φ*=4.8 is consistent with productive radical chain propagation along with the photoredox manifold.^9^

With these conditions in hand, we evaluated the scope of the processes with a range of oximes with different substitution patterns. This enabled the divergent preparation of a variety of γ‐Cl,N‐Cl imines (**7 b**–**k**) and γ‐F ketones (**8 b**–**n**) in good yields. Overall, the reactions were largely undeterred by a variety of functional groups as demonstrated by the successful formation of products containing electron‐rich and ‐poor (hetero)aromatic, nitrile, azide, ester, free alcohol, (thio)ether, and *N*‐Boc groups. Furthermore, we were able to apply the γ‐fluorination to the modification of a structurally complex lithocolic acid derivative (**7 o**).

The challenges associated with the development of these radical transpositions can be appreciated by evaluating the reaction parameters. According to our calculations, there is a very small gain in BDEs^21^ going from iminyl species **G** to carbon radical **H** (compare the BDEs for N−**H^a^** and C−**H^b^**), and these 1,5‐abstraction process are generally endergonic (for Δ*G*°=4.2 kcal mol^−1^; Scheme 4).^9^ This is in line with our experimental results showing the ability of these γ‐chlorination and γ‐fluorination cascades to functionalise tertiary centres, which are the most activated.^22^ Our results also explain why previous methods required a stoichiometric acid. Protonation of the iminyl radical renders the reaction extremely exergonic and leads to a significant gain in BDEs for the abstraction process (compare the BDEs for N−**H^c^** and C−**H^d^**).^23^

In conclusion, we have developed a photoinduced strategy for the preparation of remotely functionalised nitriles and ketones. These reactions are triggered by the organophotoredox generation of iminyl radicals that give access to distal carbon radicals upon transposition by C(sp^3^)−C(sp^3^) or C(sp^3^)−H bond cleavage.

## Conflict of interest

The authors declare no conflict of interest.

## Supporting information

As a service to our authors and readers, this journal provides supporting information supplied by the authors. Such materials are peer reviewed and may be re‐organized for online delivery, but are not copy‐edited or typeset. Technical support issues arising from supporting information (other than missing files) should be addressed to the authors.

Supplementary
