# Supplementary material for: Photoinduced Remote Functionalisations by Iminyl Radical Promoted C−C and C−H Bond Cleavage Cascades
Source: Angew Chem Int Ed Engl. 2017 Dec 4;57(3):744–8. doi: 10.1002/anie.201710790 (PMC5814920; doi:10.1002/anie.201710790)
Supplement: Supplementary file 1 — Supplementary [file ANIE-57-744-s001.pdf]

Angewandte Chemie
Eine Zeitschrift der Gesellschaft Deutscher Chemiker

Supporting Information

Photoinduced Remote Functionalisations by Iminyl Radical Promoted C−C and C−H Bond Cleavage Cascades

*Elizabeth M. Dauncey[+], Sara P. Morcillo[+], James J. Douglas, Nadeem S. Sheikh, and Daniele Leonori**

anie_201710790_sm_miscellaneous_information.pdf

Table of Contents

1 General Experimental Details 3

2 Starting Material Synthesis 4

2.1 Synthesis of Ketones 4

2.2 Synthesis of Oximes 19

3 Reaction Optimizations 40

3.1 Cascade Ring-Opening-Fluorination 40

3.2 Cascade Ring-Opening-Chlorination 42

3.3 Cascade Ring Opening-Azidation 44

3.4 Cascade 1,5-H Abstraction-Chlorination 46

3.5 Cascade 1,5-H Abstraction-Fluorination 48

4 Picture of Reaction Set-Up 52

5 Reaction Products 53

5.1 Cascade Ring-Opening-Fluorination 53

5.2 Cascade Ring-Opening-Chlorination 62

5.3 Cascade Ring Opening-Azidation 66

5.4 Cascade 1,5-H Abstraction-Chlorination 68

5.5 Cascade 1,5-H Abstraction-Fluorination 73

6 Mechanistic Considerations 80

6.1 Emission Quenching Experiments 80

6.2 Quantum Yield Determination **83**
6.3 Ring-Opening: DFT Reactivity Scales **84**
6.4 1,5-H Abstraction: DFT Reactivity Scales **85**
6.5 Role of Ag(I) in the 1,5-H Abstraction Fluorination **86**

7 Computational Studies **89**
7.1 Computational Methods **89**
7.2 Activation Energy ($\Delta G^{\ddagger}$) and Reaction Energy (ΔG^{o}) for Ring-opening Reactions **90**
7.3 Activation Energy ($\Delta G^{\ddagger}$) and Reaction Energy (ΔG^{o}) for 1,5-H atom Abstraction **128**
7.4 Bond Dissociation Enthalpies **150**

8 NMR Spectra **191**

9 References **333**

1 General Experimental Details

All required fine chemicals were used directly without purification unless stated otherwise. All air and moisture sensitive reactions were carried out under nitrogen atmosphere using standard Schlenk manifold technique. THF was distilled from sodium/benzophenone, CH_2Cl_2 and was distilled from CaH_2, CH_3CN was distilled from activated 4Å molecular sieves, EtN(*i*-Pr)$_2$ was distilled over KOH. ^{1}H and ^{13}C Nuclear Magnetic Resonance (NMR) spectra were acquired at various field strengths as indicated and were referenced to $CHCl_3$ (7.26 and 77.0 ppm for ^{1}H and ^{13}C respectively). ^{1}H NMR coupling constants are reported in Hertz and refer to apparent multiplicities and not true coupling constants. Data are reported as follows: chemical shift, integration, multiplicity (s = singlet, br s = broad singlet, d = doublet, t = triplet, q = quartet, qi = quintet, sx = sextet, sp = septet, m = multiplet, dd = doublet of doublets, etc.), proton assignment (determined by 2D NMR experiments: COSY, HSQC and HMBC) where possible. High-resolution mass spectra were obtained using a JEOL JMS-700 spectrometer or a Fissions VG Trio 2000 quadrupole mass spectrometer. Spectra were obtained using electron impact ionization (EI) and chemical ionization (CI) techniques, or positive electrospray (ES). Infra-red spectra were recorded using a JASCO FT/IR 410 spectrometer or using an ATI Mattson Genesis Seris FTIR spectrometer as evaporated films or liquid films. Analytical TLC: aluminum backed plates pre-coated (0.25 mm) with Merck Silica Gel 60 F254. Compounds were visualized by exposure to UV-light or by dipping the plates in permanganate ($KMnO_4$) stain followed by heating. Flash column chromatography was performed using Merck Silica Gel 60 (40–63 μm). All mixed solvent eluents are reported as v/v solutions. UV/Vis spectra were obtained using an Agilent 6453 spectrometer and 1 mm High Precision Cell made of quartz from Hellma Analytics.

The LEDs were bought from LEDLightZone.

All the reactions were conducted in CEM 10 mL glass microwave tubes.

2 Starting Material Synthesis

2.1 Synthesis of Ketones

2,2a,7,7a-Tetrahydro-1*H*-cyclobuta[*a*]inden-1-one (S1)

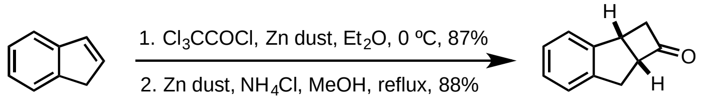

S1

A solution of trichloroacetyl chloride (0.9 mL, 7.8 mmol, 1.5 equiv.) in dry Et_2O (20 mL) was added dropwise under sonication over 45 min. to a solution of indene (0.6 g, 5.2 mmol, 1.0 equiv.) in dry Et_2O (40 mL) containing zinc dust (1.0 g, 15.5 mmol, 3.0 equiv.), at a rate such that the temperature did not exceed 15-20 °C. Sonication was continued for additional 30 min. The mixture was diluted with Et_2O (50 mL) and filtered over a pad of Celite. The filtrate was washed with H_2O (2 x 25 mL), Na_2CO_3sat (4 x 25 mL), and brine (2 x 25 mL). The organic layer was dried ($MgSO_4$), filtered, evaporated and purified by column chromatography on silica gel (1.03 g, 87%). The product was solubilised in MeOH (50 mL) and ammonium chloride (1.88 g, 9.2 mmol, 2.0 equiv.) and zinc dust (1.2 g, 18.4 mmol, and 4.0 equiv.) were added. The reaction mixture was heated under reflux overnight. The mixture was cooled to room temperature, diluted with CH_2Cl_2 (30 mL) and filtered over Celite. The filtrate was washed with H_2O (2 x 25 mL), Na_2CO_{3sat} (4 x 25 mL), and brine (2 x 25 mL). The organic layer was dried ($MgSO_4$), filtered, evaporated and purified by column chromatography on silica gel eluting with petrol–EtOAc (9:1) to give **S1** as an oil (720 mg, 88%). ^{1}H NMR (400 MHz, $CDCl_3$) δ 7.40–7.32 (1H, m, *J* = 6.7, 2.1 Hz), 7.32–7.20 (3H, m, *J* = 7.0, 3.1 Hz), 4.18–4.01 (2H, m), 3.70–3.58 (1H, m), 3.35 (1H, d, *J* = 16.9 Hz), 3.20–3.06 (1H, m), 2.93 (1H, d, *J* = 17.5 Hz); ^{13}C NMR (101 MHz, $CDCl_3$) δ 212.4, 144.6, 143.1, 127.4, 127.3, 125.4, 125.1, 62.8, 55.7, 36.6, 34.0. Data in accordance with the literature.[1]

1,1-Dimethyl-3,4-dihydronaphthalen-2(1*H*)-one (S2)

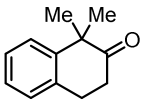

A solution of β-tetralone (400 mg, 2.74 mmol, 1.0 equiv.) in THF (12 mL) was treated with NaH (138 mg, 5.75 mmol, 2.1 equiv., 60% dispersion in mineral oil) and the mixture stirred at room temperature for 10 min. MeI (0.5 mL, 8.22 mmol, 3.0 equiv.) was added and the mixture was heated under reflux for 2 hours. The mixture was cooled to room temperature, diluted with NH_4Cl_{sat} (10 mL) and extracted with Et_2O (3 x 20 mL). The combined organic layers were dried ($MgSO_4$), filtered and evaporated. Purification by column chromatography

on silica gel, eluting with petrol–EtOAc (98:2), gave **S2** (270 mg, 57%) as an oil. ^{1}H NMR (400 MHz, $CDCl_3$) δ 7.35 (1H, d, J = 7.8 Hz), 7.30–7.24 (1H, m), 7.23–7.15 (2H, m), 3.11 (2H, t, J = 6.9 Hz), 2.72–2.67 (2H, m), 1.44 (6H, s); ^{13}C NMR (101 MHz, $CDCl_3$) δ 214.8, 143.5, 135.2, 128.1, 127.1, 126.4, 126.1, 47.8, 37.2, 28.6, 26.9. Data in accordance with the literature.[2]

3',4'-Dihydro-2'H-spiro[cyclohexane-1,1'-naphthalen]-2'-one (S3)

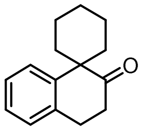

A solution of β-tetralone (400 mg, 2.74 mmol, 1.0 equiv.) in THF (12 mL) was treated with NaH (138 mg, 5.75 mmol, 2.1 equiv., 60% dispersion in mineral oil) and the mixture stirred at room temperature for 10 min. 1,5-Dibromopentane (0.75 mL, 5.48 mmol, 3.0 equiv.) was added and the reaction refluxed at 105 °C for 4 hours. The mixture was cooled to room temperature, diluted with NH_4Cl_{sat} (10 mL) and extracted with Et_2O (3 x 20 mL). The combined organic layers were dried ($MgSO_4$), filtered and evaporated. Purification by column chromatography on silica gel, eluting with petrol–EtOAc (98:2), gave **S3** (390 mg, 67%) as a solid. ^{1}H NMR (500 MHz, $CDCl_3$) δ 7.39 (1H, d, J = 7.9 Hz), 7.26–7.23 (1H, m), 7.18 (1H, t, J = 7.3 Hz), 7.13 (1H, d, J = 7.4 Hz), 3.20 (2H, t, J = 7.1 Hz), 2.70 (2H, t, J = 7.1 Hz), 2.18–2.11 (2H, m), 1.79–1.62 (7H, m), 1.38–1.29 (1H, m); ^{13}C NMR (126 MHz, $CDCl_3$) δ 214.2, 144.1, 135.8, 128.3, 126.9, 126.4, 126.4, 51.9, 36.4, 34.5, 30.1, 25.8, 23.3. This compound was known in the literature,[3] but no spectroscopic data was given.

1-(4-Phenyl-1-tosylpiperidin-4-yl)ethan-1-one (S4)

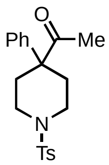

A solution of 1-(4-phenyl-4-piperidinyl)-ethanone hydrochloride (500 mg, 1.97 mmol, 1 equiv.) in CH_2Cl_2 (5.0 mL) was treated with Et_3N (0.7 mL, 4.33 mmol, 2.2 equiv.) and *p*-TsCl (561 mg, 2.96 mmol, 1.5 equiv.). The reaction mixture was stirred for 2 h and then quenched with H_2O (5.0 mL). The layers were separated and the aqueous layer was extracted with CH_2Cl_2 (3 x 30 mL). The combined organic layers were dried ($MgSO_4$), filtered and evaporated. Purification by column chromatography on silica gel eluting with petrol–EtOAc (9:1), gave **S4** (432 mg, 1.20 mmol, 61%) as a solid. FT-IR ν_{max} (film)/cm^{-1} 2925, 1702,

1353, 1212, 1092, 934, 724; ^{1}H NMR (400 MHz, $CDCl_3$) δ 7.61 (2H, d, *J* = 7.7 Hz), 7.38–7.27 (5H, m), 7.21 (2H, d, *J* = 7.8 Hz), 3.66–3.55 (2H, m), 2.63–2.46 (4H, m), 2.43 (3H, s), 2.15–2.06 (2H, m), 1.81 (3H, s); ^{13}C NMR (126 MHz, $CDCl_3$) δ 208.7, 143.7, 140.7, 133.1, 129.8, 129.2, 127.6, 127.55, 126.0, 54.1, 43.7, 32.2, 25.6, 21.6; HRMS (ESI) Found MK^+ 369.1028, $C_{20}H_{23}O_3NKS$ requires 396.1030

tert-Butyl 4-Acetyl-4-phenylpiperidine-1-carboxylate (S5)

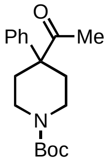

A solution of 1-(4-phenyl-4-piperidinyl)-ethanone hydrochloride (500 mg, 1.97 mmol, 1 equiv.) in CH_2Cl_2 (5.0 mL) was treated with Et_3N (0.7 mL, 4.33 mmol, 2.2 equiv.) and Boc_2O (470 mg, 2.17 mmol, 1.1 equiv.) in CH_2Cl_2 (5 mL). The reaction mixture was stirred for 2 h and then quenched with H_2O (5.0 mL). The layers were separated and the aqueous layer was extracted with CH_2Cl_2 (3 x 30 mL). The combined organic layers were dried ($MgSO_4$), filtered and evaporated. Purification by column chromatography on silica gel eluting with petrol–EtOAc (9:1), gave **S5** (507 mg, 1.67 mmol, 85%) as an oil. ^{1}H NMR (400 MHz, $CDCl_3$) δ 7.33–7.26 (2H, m), 7.24–7.18 (3H, m,), 3.70 (2H, br s), 3.17–3.02 (2H, m), 2.34–2.25 (2H, m), 1.92 (2H, br s), 1.84 (3H, s), 1.37 (9H, s); ^{13}C NMR (126 MHz, $CDCl_3$) δ 209.0, 154.8, 141.0, 129.1, 127.4, 126.3, 79.6, 54.8, 41.4, 40.6, 32.8, 32.2, 28.4, 25.6.

(3*R*,5*S*,8*S*,9*S*,10*S*,13*S*,14*S*)-10-Methyl-17-oxohexadecahydro-1*H*-cyclopenta[*a*] phenanthren-3-yl Acetate (S6)

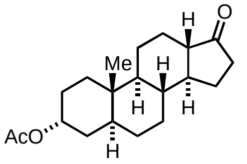

A solution of *trans*-Androsterone (250 mg, 0.86 mmol, 1 equiv.) in CH_2Cl_2 (2.0 mL) was cooled to 0 ºC, treated with DMAP (10 mg, 0.09 mmol, 0.1 equiv.), pyridine (0.35 mL, 4.30 mmol, 5.0 equiv.) and Ac_2O (0.25 mL, 2.58 mmol, 3.0 equiv.) and allowed to warm to room temperature overnight. The reaction mixture was diluted with H_2O (5 mL) and EtOAc (5 mL) and the layers were separated. The aqueous layer was extracted with EtOAc (3 x 10 mL). The combined organic layers were dried ($MgSO_4$), filtered and evaporated to give **S6** (200 mg, 0.6 mmol, 70%) as a solid. ^{1}H NMR (500 MHz, $CDCl_3$) δ 4.75–4.62 (1H, m), 2.43 (1H, dd, *J*

= 19.3, 8.6 Hz), 2.11–2.03 (1H, m), 2.02 (3H, s), 1.98–1.87 (1H, m), 1.84–1.70 (4H, m), 1.68–1.60 (2H, m), 1.56–1.44 (3H, m), 1.42–1.13 (7H, m), 1.08–0.91 (2H, m), 0.85 (3H, d, *J* = 2.3 Hz), 0.85 (3H, s), 0.71 (1H, td, *J* = 11.6, 4.0 Hz); ^{13}C NMR (126 MHz, $CDCl_3$) δ 221.3, 170.7, 73.5, 54.3, 51.4, 47.8, 44.7, 36.7, 35.9, 35.7, 35.0, 34.0, 31.5, 30.8, 28.3, 27.4, 21.8, 21.5, 20.5, 13.8, 12.2. Data in accordance with the literature.[4]

(2*S*,4a*R*,4b*S*,6a*S*,7a*S*,8a*S*,8b*S*,8c*R*,8d*R*,9a*R*,9b*R*)-9b-Hydroxy-4a,6a-dimethyl-7-oxooctadecahydro-1H-cyclopropa[4,5]cyclopenta[1,2-a]cyclopropa[l]phenanthren-2-yl Acetate (S7)

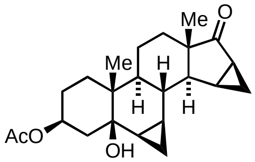

A solution of (2S,4a*R*,4b*S*,6a*S*,7a*S*,8a*S*,8b*S*,8c*R*,8d*R*,9a*R*,9b*R*)-2,9b-dihydroxy-4a,6a-dimethyloctadecahydro-7H-cyclopropa[4,5]cyclopenta[1,2-a]cyclopropa[l]phenanthren -7-one (200 mg, 0.61 mmol) in CH_2Cl_2 (1.5 mL) was cooled to 0 °C, treated with DMAP (15 mg, 0.12 mmol, 0.2 equiv.), pyridine (0.5 mL, 6.1 mmol, 10.0 equiv.) and Ac_2O (0.35 mL, 3.64 mmol, 6.0 equiv.) and allowed to warm to room temperature overnight. The reaction was diluted with H_2O (3 mL) and EtOAc (3 mL) the layers were separated. The aqueous layer was extracted with EtOAc (3 x 10 mL). The combined organic layers were dried ($MgSO_4$), filtered and evaporated to give **S7** (210 mg, 92%) as an off white solid. FT-IR ν_{max} (film)/cm^{-1} 2936, 1716, 1375, 1249, 908; ^{1}H NMR (500 MHz, $CDCl_3$) δ 5.15–5.11 (1H, m), 2.29 (1H, dd, *J* = 15.4, 3.7 Hz), 2.21–2.11 (2H, m), 2.09 (3H, s), 2.05–2.00 (1H, m), 1.82–1.74 (2H, m), 1.73–1.62 (5H, m), 1.55–1.42 (3H, m), 1.34–1.28 (2H, m), 1.21–1.15 (1H, m), 1.13–1.07 (1H, m), 0.95 (3H, s), 0.90 (3H, s), 0.88–0.80 (2H, m), 0.74–0.68 (m, 1H); ^{13}C NMR (126 MHz, $CDCl_3$) δ 216.2, 169.9, 73.0, 70.5, 52.2, 42.9, 41.4, 40.5, 35.1, 33.5, 31.0, 26.0, 24.6, 22.4, 22.2, 21.5, 21.3, 20.2, 20.2, 18.9, 17.7, 13.8, 11.4; HRMS (ESI) Found MNa^+ 395.2195, $C_{23}H_{32}O_4Na$ requires 395.2193.

Methyl (4*R*,4a*S*,6a*R*,9*S*,11a*R*,11b*S*)-4,9,11b-Trimethyl-8-oxotetradecahydro-6a,9-methanocyclohepta[a]naphthalene-4-carboxylate (S8)

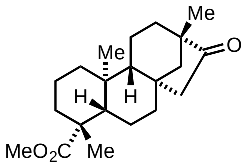

A solution of isosteviol (1.0 g, 3.1 mmol, 1.0 equiv.) in DMF (10 mL) was treated with K_2CO_3 (740 mg, 5.3 mmol, 1.7 equiv.) and MeI (0.35 mL, 5.3 mmol, 1.7 equiv.). The mixture was stirred at room temperature overnight and then diluted with 1M HCl (20 mL) and CH_2Cl_2 (30 mL). The layers were separated and the aqueous layer was extracted with mixture was extracted with CH_2Cl_2 (x 3). The combined organic layers were dried ($MgSO_4$), filtered and evaporated to give **S8** (820 mg, 78%) as a solid. ^{1}H NMR (400 MHz, $CDCl_3$) δ 3.63 (3H, s), 2.62 (1H, dd, *J* = 18.5, 3.5 Hz), 2.17 (1H, d, *J* = 13.2 Hz), 2.05–1.51 (8H, m), 1.51–1.31 (2H, m), 1.18 (3H, s), 1.28–0.85 (5H, m), 0.97 (3H, s), 0.68 (3H, s); ^{13}C NMR (100 MHz, CDCl3): δ 222.4, 177.8, 57.0, 54.7, 54.3, 51.2, 48.7, 48.5, 48.4, 43.8, 41.5, 39.8, 39.4, 37.9, 37.3, 28.8, 21.7, 20.3, 19.9, 18.9, 13.2. Data in accordance with the literature.[5]

General Procedure for the Synthesis of Ketones S9–22 – GP1

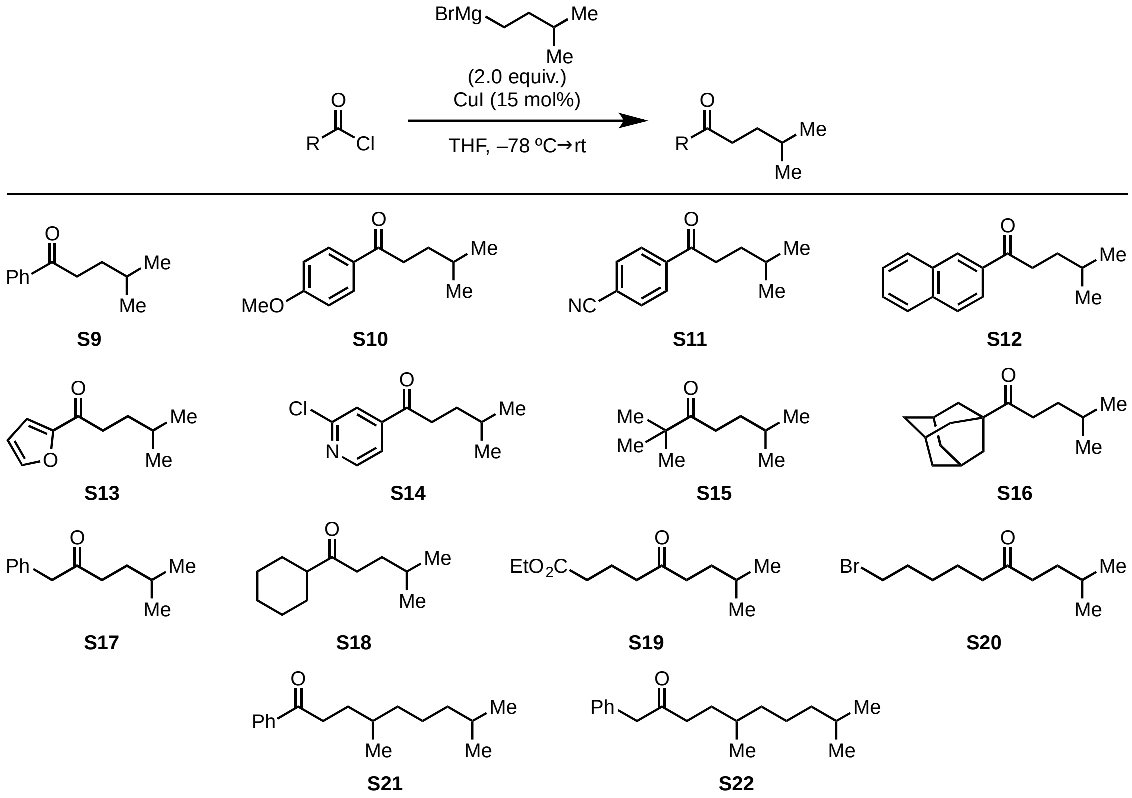

A dry Schlenk tube was charged with CuI (0.15 equiv.), THF (0.1 M) and the acid chloride (1.0 equiv.). The mixture was cooled to –78 °C and *i*-pentylmagnesium bromide (1.25 equiv.) was added dropwise. The mixture was warmed to room temperature overnight. The mixture was diluted with NH_4Cl and EtOAc. The layers were separated and the aqueous layer was extracted with EtOAc (x 3). The combined organic layers were dried ($MgSO_4$), filtered and evaporated. The crude was purified by column chromatography on silica gel.

4-Methyl-1-phenylpentan-1-one (S9)

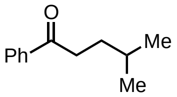

Following **GP1**, benzoyl chloride (0.4 mL, 3.56 mmol) gave **S9** (590 mg, 94%) as an oil. ^{1}H NMR (400 MHz, $CDCl_3$) δ 7.96 (2H, d, *J* = 7.7 Hz), 7.55 (1H, t, *J* = 7.3 Hz), 7.46 (2H, t, *J* = 7.6 Hz), 3.02–2.91 (2H, m), 1.72–1.56 (3H, m), 0.95 (6H, d, *J* = 6.0 Hz); ^{13}C NMR (101 MHz, $CDCl_3$) δ 200.9, 137.2, 133.0, 128.7, 128.2, 36.8, 33.4, 28.0, 22.6. Data in accordance with the literature.[6]

1-(4-Methoxyphenyl)-4-methylpentan-1-one (S10)

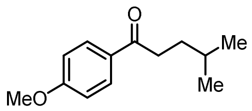

Following **GP1**, 4-methoxybenzoyl chloride (0.65 mL, 2.90 mmol) gave **S10** (413 mg, 69%) as an oil. ^{1}H NMR (400 MHz, $CDCl_3$) δ 7.95 (2H, d, *J* = 8.8 Hz), 6.94 (2H, t, *J* = 5.8 Hz), 3.87 (3H, s), 2.90 (2H, dd, *J* = 14.8, 7.4 Hz), 1.68–1.57 (3H, m), 0.94 (6H, d, *J* = 6.1 Hz); ^{13}C NMR (101 MHz, $CDCl_3$) 198.7, 163.6, 130.2, 113.5, 53.2, 36.4, 33.9, 28.5, 22.3. Data in accordance with the literature.[7]

4-(4-Methylpentanoyl)benzonitrile (S11)

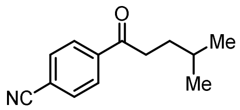

Following **GP1**, 4-cyanobenzoyl chloride (250 mg, 1.50 mmol) gave **S11** (273 mg, 91%) as an oil. ^{1}H NMR (400 MHz, $CDCl_3$) δ 8.06–8.02 (2H, m), 7.79–7.75 (2H, m), 3.02–2.93 (2H, m,), 1.66–1.60 (3H, m), 0.96–0.93 (6H, m); ^{13}C NMR (101 MHz, $CDCl_3$) δ 199.2, 132.5, 128.5, 126.2, 36.9, 32.8, 27.8, 22.4. Data in accordance with the literature.[8]

4-Methyl-1-(naphthalen-2-yl)pentan-1-one (S12)

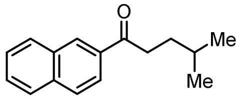

Following **GP1**, 2-napthoyl chloride (500 mg, 2.60 mmol) gave **S12** (367 mg, 63%) as an oil. ^{1}H NMR (400 MHz, $CDCl_3$) δ 8.48 (1H, s), 8.04 (1H, dd, *J* = 8.6, 1.6 Hz), 7.98 (1H, d, *J* = 8.0 Hz), 7.92–7.86 (2H, m), 7.58 (2H, ddd, *J* = 15.0, 13.6, 6.7 Hz), 3.11 (2H, dd, *J* = 9.5, 5.4 Hz), 1.75–1.65 (3H, m), 0.98 (6H, d, *J* = 6.1 Hz); ^{13}C NMR (101 MHz, $CDCl_3$) δ 200.8, 135.5, 134.4, 132.6, 129.6, 129.6, 128.4, 128.3, 127.8, 126.7, 124.0, 36.7, 33.4, 27.9, 22.5. This compound was known in literature,[9] but no spectroscopic data was provided.

1-(Furan-2-yl)-4-methylpentan-1-one (S13)

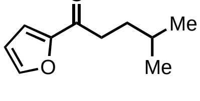

Following **GP1**, furan-2-carbonyl chloride (0.65 mL, 3.83 mmol) gave **S13** (374 mg, 59%) as an oil. ^{1}H NMR (400 MHz, $CDCl_3$) δ 7.57 (1H, dd, *J* = 1.7, 0.7 Hz), 7.17 (1H, dd, *J* = 3.5, 0.7

Hz), 6.52 (1H, dd, *J* = 3.5, 1.7 Hz), 2.84–2.79 (2H, m), 1.67–1.56 (4H, m), 0.95–0.92 (6H, d, *J* = 6.4 Hz). Data in accordance with the literature.[10]

1-(2-Chloropyridin-4-yl)-4-methylpentan-1-one (S14)

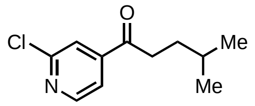

Following **GP1**, 2-chloroisonicotinoyl chloride (200 mg, 1.14 mmol) gave **S14** (120 mg, 50%) as an oil. ^{1}H NMR (500 MHz, $CDCl_3$) δ 8.57 (1H, d, *J* = 5.1 Hz), 7.75 (1H, d, *J* = 0.5 Hz), 7.65 (1H, dd, *J* = 5.1, 0.9 Hz), 2.96–2.91 (2H, m), 1.68–1.59 (3H, m), 0.95 (6H, d, *J* = 6.1 Hz); ^{13}C NMR (101 MHz, $CDCl_3$) δ 198.4, 152.9, 150.9, 145.8, 122.4, 119.9, 37.1, 32.5, 27.7, 22.3; HRMS (APCI) Found MH^+ 212.0837, $C_{11}H_{15}ONCl$ requires 212.0837.

2,2,6-Trimethylheptan-3-one (S15)

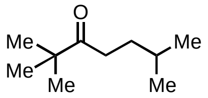

Following **GP1,** pivaloyl chloride (2.4 g, 20 mmol) gave **S15** (2.0 g, 65%) as an oil. ^{1}H NMR (500 MHz, Chloroform-*d*) δ 2.44 (2H, t), 1.54–1.45 (1H, m), 1.44–1.37 (2H, m), 1.11 (9H, s), 0.86 (6H, d, *J* = 6.6 Hz); ^{13}C NMR (126 MHz, $CDCl_3$) δ 216.1, 44.2, 34.4, 32.8, 27.7, 26.4, 22.4. This compound was known in the literature,[11] but spectroscopic data was not provided.

1-((3*r*,5*r*,7*r*)-Adamantan-1-yl)-4-methylpentan-1-one (S16)

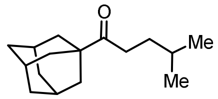

Following **GP1**, (3*r*,5*r*,7*r*)-adamantane-1-carbonyl chloride (250 mg, 1.25 mmol) gave **S16** (230 mg, 78%) as an oil. FT-IR ν_{max} (film)/cm^{-1} 2903, 2849, 1697, 1451, 1164, 754; ^{1}H NMR (400 MHz, $CDCl_3$) δ 2.46–2.39 (2H, m), 2.05–2.01 (3H, m), 1.80 (6H, d, *J* = 2.7 Hz), 1.78–1.65 (6H, m), 1.51 (1H, m), 1.45–1.38 (2H, m), 0.88 (6H, d, *J* = 6.5 Hz); ^{13}C NMR (101 MHz, $CDCl_3$) δ 216.0, 46.4, 38.3, 36.6, 33.9, 32.6, 28.0, 27.8, 22.5; HRMS (ASAP) Found MH^+ 235.2047, $C_{16}H_{27}O$ requires 235.2056.

5-Methyl-1-phenylhexan-2-one (S17)

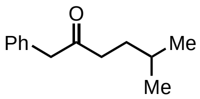

Following **GP1**, phenylacetyl chloride (0.45 mL, 3.23 mmol) gave **S17** (558 mg, 91%) as an oil. ^{1}H NMR (400 MHz, $CDCl_3$) δ 7.35 (2H, t, *J* = 7.4 Hz), 7.29 (1H, d, *J* = 7.4 Hz), 7.22 (2H, d, *J* = 7.5 Hz), 3.71 (2H, s), 2.49–2.44 (2H, m), 1.56–1.40 (3H, m), 0.85 (6H, d, *J* = 6.1 Hz); ^{13}C NMR (101 MHz, $CDCl_3$) δ 208.9, 134.5, 192.9, 128.8, 127.1, 50.3, 40.2, 32.7, 22.7, 22.4. Data in accordance with the literature.[12]

1-Cyclohexyl-4-methylpentan-1-one (S18)

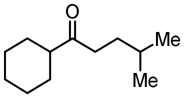

Following **GP1**, cyclohexanecarbonyl chloride (2.1 g, 15 mmol) gave **S19** (2.4 g, 88%) as an oil. FT-IR ν_{max} (film)/cm^{-1} 2933, 2859, 1717, 1451, 1164, 754;;^{1}H NMR (400 MHz, $CDCl_3$) δ 2.37 (2H, t, *J* = 7.6 Hz), 2.34– 2.24 (1H, m), 1.81–1.68 (2H, m), 1.65–1.58 (1H, m), 1.54–1.35 (4H, m), 1.35–1.00 (6H, m), 0.83 (6H, d, *J* = 7.6 Hz); ^{13}C NMR (101 MHz ,$CDCl_3$) δ 214.7, 50.9, 38.8, 32.7, 28.7, 27.9, 26.0, 25.6, 22.5; HRMS (ESI) Found MNa^+ 205.1564 $C_{12}H_{22}NaO$ requires 205.1568.

Ethyl 8-Methyl-5-oxononanoate (S19)

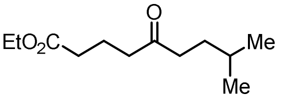

Following **GP1**, ethyl 5-chloro-5-oxopentanoate (500 mg, 2.8 mmol) gave **S19** (469 mg, 78%) as an oil. FT-IR ν_{max} (film)/cm^{-1} 3340, 2969, 2930, 1466, 1378, 1340, 1160, 1106, 950, 815, 688; ^{1}H NMR (400 MHz, $CDCl_3$) δ 4.12 (2H, q, *J* = 7.1 Hz), 2.48 (2H, t, *J* = 7.2 Hz), 2.41–2.36 (2H, m), 2.31 (2H, t, *J* = 7.2 Hz), 1.92–1.84 (2H, m), 1.58–1.48 (1H, m), 1.49–1.39 (2H, m), 1.25 (3H, t, *J* = 7.1 Hz), 0.88 (6H, d, *J* = 6.4 Hz); ^{13}C NMR (101 MHz, $CDCl_3$) δ 210.6, 173.2, 60.3, 41.5, 40.9, 33.4, 32.6, 27.7, 22.3, 18.9, 14.2; HRMS (ESI) Found MNa^+ 237.1454, $C_{12}H_{22}O_3Na$ requires 237.1461.

10-Bromo-2-methyldecan-5-one (S20)

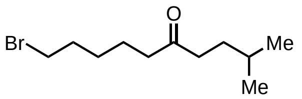

Following **GP1**, 6-bromohexanoyl chloride (450 mg, 2.1 mmol) gave **S20** (430 mg, 82%) as an oil. FT-IR ν_{max} (film)/cm^{-1} 2953, 2867, 1711, 1464, 1366, 1253, 1169, 755; ^{1}H NMR (500 MHz, $CDCl_3$) δ 3.40 (2H, t, *J* = 6.8 Hz), 2.47–2.35 (4H, m), 1.92–1.82 (2H, m), 1.64–1.56 (2H, m), 1.55–1.48 (1H, m), 1.48–1.39 (4H, m), 0.89 (6H, d, *J* = 6.5 Hz); ^{13}C NMR (126 MHz, $CDCl_3$) δ 211.2, 42.4, 40.9, 33.6, 32.7, 32.6, 27.8, 27.7, 22.9, 22.3; HRMS (ASAP) Found MH$^+$ 249.0840, $C_{11}H_{22}BrO$ requires 249.0849.

4,8-Dimethyl-1-phenylnonan-1-one (S21)

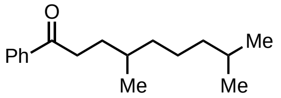

Following **GP1** but using (2,6-dimethylheptyl)magnesium bromide, benzoyl chloride (0.41 mL, 3.5 mmol) gave **S21** (781 mg, 90%) as an oil.[a] HRMS (ASAP) Found MH$^+$ 247.2047, $C_{17}H_{27}O$ requires 247.2056.

5,9-Dimethyl-1-phenyldecan-2-one (S22)

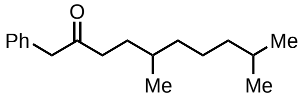

Following **GP1** but using (2,6-dimethylheptyl)magnesium bromide, phenyl acetyl chloride (500 mg, 3.25 mmol) gave **S22** (365 mg, 43%) as an oil. FT-IR ν_{max} (film)/cm^{-1} 2954, 2926, 2868, 1709, 1454, 1215, 752, 698; ^{1}H NMR (400 MHz, $CDCl_3$) δ 7.33 (2H, t, *J* = 7.3 Hz), 7.30–7.24 (1H, m), 7.21 (2H, d, *J* = 7.2 Hz), 3.69 (2H, s), 2.49–2.40 (2H, m), 1.63–1.54 (2H, m), 1.53–1.45 (1H, m), 1.40–1.30 (2H, m), 1.27–1.15 (3H, m), 1.13–1.06 (2H, m), 0.85 (6H, d, *J* = 6.6 Hz), 0.80 (3H, d, *J* = 6.2 Hz); ^{13}C NMR (101 MHz, $CDCl_3$) δ 209.0, 134.5, 129.5, 128.8, 127.1, 50.3, 39.9, 39.4, 37.1, 32.5, 30.9, 26.6, 24.8, 22.8, 22.7, 19.5; HRMS (ASAP) Found MH$^+$ 261.2203, $C_{18}H_{29}O$ requires 261.2213.

[a] In this case **S21** was directly used in the next step without chromatographic purification.

General Procedure for Synthesis of Ketones S23–26 – GP2

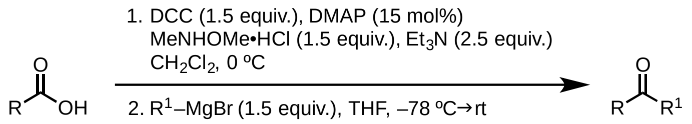

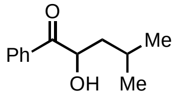

S23

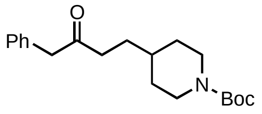

S24

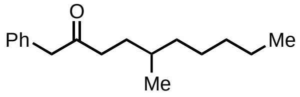

S25

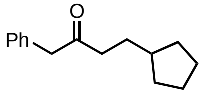

S26

A solution of carboxylic acid (1.0 equiv.) in CH_2Cl_2 (0.1 M) was cooled to 0 °C and treated with N,O-dimethylhydroxylamine hydrochloride (1.5 equiv.), DCC (1.5 equiv.), DMAP (15 mol%) and Et_3N (2.5 equiv.). The mixture was stirred overnight at room temperature, filtered through celite and diluted with HCl 0.5 N. The layers were separated and the aqueous layer was extracted with CH_2Cl_2 (x 3). The combined organic layers were dried ($MgSO_4$), filtered and evaporated. The crude was purified by column chromatography on silica gel to give the corresponding Weinreib amides. A solution of the Weinreib amide (1.0 equiv.) in THF (0.1 M) was cooled to –78 °C and treated with the Grignard reagent (1.5 equiv.) by dropwise. The reaction was allowed to warm to room temperature overnight. The mixture was diluted with NH_4Cl_{sat} and EtOAc. The layers were separated and the aqueous layer was extracted with EtOAc (x 3). The combined organic layers were dried ($MgSO_4$), filtered and evaporated. The crude was purified by column chromatography on silica gel.

3-Hydroxy-5-methyl-1-phenylhexan-2-one (S23)

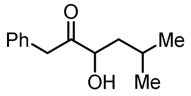

Following **GP2**, 2-hydroxy-4-methylpentanoic acid (500 mg, 3.1 mmol) gave **S23** (175 mg, 57%) as an oil. ^{1}H NMR (500 MHz, $CDCl_3$) δ 7.30–7.20 (3H, m), 7.14 (2H, d, J = 7.2 Hz), 4.22 (1H, dd, J = 10.2, 2.9 Hz), 3.76 (1H, d, J = 15.8 Hz), 3.70 (1H, d, J = 15.8 Hz), 1.92–1.82 (1H, m), 1.59–1.47 (1H, m), 1.45–1.30 (1H, m), 0.90 (6H, d, J = 6.7 Hz); ^{13}C NMR (126 MHz, $CDCl_3$) δ 210.4, 133.3, 129.6, 128.9, 127.4, 74.9, 44.9, 42.8, 24.9, 23.7, 21.4; HRMS (ESI) Found MNa^+ 229.1212 $C_{13}H_{18}NaO_2$ requires 229.1204.

***tert*-Butyl 4-(3-Oxo-4-phenylbutyl)piperidine-1-carboxylate (S24)**

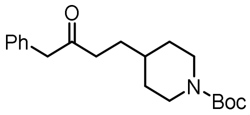

Following **GP2**, 3-(1-(*tert*-butoxycarbonyl)piperidin-4-yl)propanoic acid (230 mg, 0.77 mmol) gave **S24** (150 mg, 59%) as an oil. FT-IR ν_{max} (film)/cm^{-1} 2253, 1375, 1038, 918, 737, 562, 555, 540; ^{1}H NMR (500 MHz, $CDCl_3$) δ 7.38–7.28 (3H, m), 7.21 (2H, t, *J* = 8.5 Hz), 4.03 (2H, s), 3.68 (2H, br s), 2.59 (2H, br s), 2.47 (2H, t, *J* = 7.5 Hz), 1.62–1.42 (7H, m), 1.44 (9H, s); ^{13}C NMR (126 MHz, $CDCl_3$) δ 208.2, 155.0, 134.4, 130.5, 129.3, 127.0, 79.1, 60.3, 38.7, 35.2, 29.9, 28.35, 21.0, 14.3; HRMS (ESI) Found MNa$^+$ 354.2040, $C_{20}H_{29}O_3NNa$ requires 354.2040.

5-Methyl-1-phenyldecan-2-one (S25)

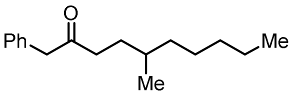

Following **GP2**, 4-methylnonanoic acid (316 mg, 2.0 mmol) gave **S25** (173 mg, 37%) as an oil. FT-IR ν_{max} (film)/cm^{-1} 2954, 2926, 2868, 1709, 1454, 1215, 752, 698; ^{1}H NMR (400 MHz, $CDCl_3$,) δ 7.26–7.21 (2H, m), 7.21–7.15 (1H, m), 7.15–7.11 (2H, m), 3.60 (2H, s), 2.48–2.17 (2H, m), 1.58-1.39 (1H, m), 1.35–0.94 (10H, m), 0.79 (3H, t, *J* = 6.9 Hz), 0.72 (3H, d, *J* = 6.3 Hz); ^{13}C NMR (101 MHz, $CDCl_3$,) δ 208.9, 134.5, 129.5, 128.8, 127.1, 68.1 50.2, 39.9, 36.5, 32.4, 30.8, 29.3, 23.1, 19.5, 14.2; HRMS (ESI) Found MNa$^+$ 269.3845, $C_{17}H_{26}NaO$ requires 269.3838.

4-Cyclopentyl-1-phenylbutan-2-one (S26)

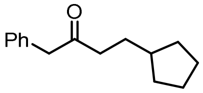

Following **GP2**, 3-cyclopentylpropanoic acid (500 mg, 3.5 mmol) gave **S26** (151 mg, 20%) as an oil. FT-IR ν_{max} (film)/cm^{-1} 1710, 1417, 1359, 1220, 1091, 902, 702; ^{1}H NMR ($CDCl_3$, 400 MHz) δ 7.26 (2H, d, *J* = 7.5 Hz), 7.23–7.16 (1H, m), 7.13 (2H, d, *J* = 7.5 Hz), 3.61 (2H, s), 2.38 (2H, t, *J* = 7.6 Hz), 1.66–155 (3H, m), 1.55–1.44 (4H, m), 1.46–1.35 (2H, m), 0.94 (2H, br s); ^{13}C NMR ($CDCl_3$, 126 MHz) δ 209.1, 134.7, 129.8, 129.1, 127.3, 50.5, 41.7, 39.9, 32.8, 30.3, 25.5; HRMS (ESI) Found MNa$^+$ 239.1406, $C_{15}H_{20}NaO$ requires 239.1412.

General Procedure for the Synthesis of Ketones S27–30 – GP3

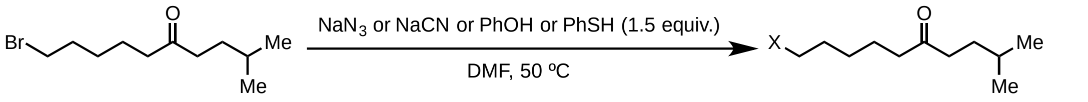

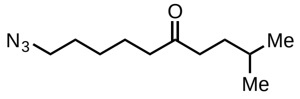

S27

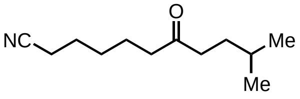

S28

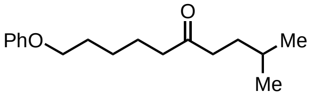

S29

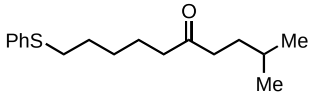

S30

A solution of **S20** (1.0 equiv.) in DMF (0.1M) was treated with the appropriate nucleophile (1.5 equiv.), warmed to 50 °C and stirred at the same temperature overnight. The mixture was diluted with H_2O (0.1 M) and EtOAc. The layers were separated and the aqueous layer was extracted with EtOAc (x 3). The combined organic layers were dried ($MgSO_4$), filtered and evaporated. The crude was purified by column chromatography on silica gel.

10-Azido-2-methyldecan-5-one (S27)

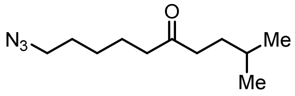

Following **GP3**, **S20** (248 mg, 1.0 mmol) gave **S27** (93 mg, 44%) as an oil. FT-IR ν_{max} (film)/cm^{-1} 1749, 1710, 1418, 1359, 1220, 1091; ^{1}H NMR (400 MHz, $CDCl_3$) δ 3.25 (2H, t, *J* = 6.9 Hz), 2.41 (2H, t, *J* = 7.5 Hz), 2.37 (2H, d, *J* = 7.5 Hz), 1.60–1.54 (4H, m), 1.53–1.25 (5H, m), 0.87 (6H, d, *J* = 6.4 Hz); ^{13}C NMR (101 MHz, $CDCl_3$) δ 211.2, 51.3, 42.5, 41.0, 32.7, 28.8, 27.8, 26.4, 23.3, 22.4; HRMS (ESI) Found MNa^+ 234.1577, $C_{11}H_{21}N_3NaO$ requires 234.1582.

10-Methyl-7-oxoundecanenitrile (S28)

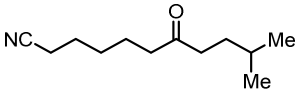

Following **GP3**, **S20** (248 mg, 1.0 mmol) gave **S28** (101 mg, 51%) as an oil. ^{1}H NMR (500 MHz, $CDCl_3$) δ 2.38 (2H, t, *J* = 7.3 Hz), 2.36–2.32 (2H, m), 2.30 (2H, t, *J* = 7.1 Hz), 1.62 (2H, m), 1.54 (2H, m), 1.47 (1H, m), 1.43–1.34 (4H, m), 0.83 (6H, d, *J* = 6.6 Hz); ^{13}C NMR (126 MHz, $CDCl_3$) δ 210.6, 119.4, 41.8, 40.6, 32.3, 27.9, 27.4, 25.0, 22.5, 22.1, 16.7; HRMS (ESI) Found MNa^+ 218.1815, $C_{12}H_{21}NNaO$ requires 218.1521.

2-Methyl-10-phenoxydecan-5-one (S29)

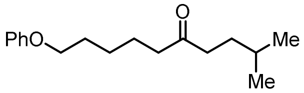

Following **GP3** but adding K_2CO_3 (2.0 equiv.), **S20** (200 mg, 0.8 mmol) gave **S29** (208 mg, 99%) as an oil. FT-IR ν_{max} (film)/cm^{-1} 2959, 2942, 2253, 1715, 1736, 1418, 1291, 1034, 918, 794, 758, 695; ^{1}H NMR (400 MHz, $CDCl_3$) δ 7.42–7.22 (2H, m), 6.99–6.88 (3H, m), 3.98 (2H, t, *J* = 6.4 Hz), 2.47 (2H, t, *J* = 7.4 Hz), 2.42 (2H, t, *J* = 7.2 Hz), 1.82 (2H, q, *J* = 7.1 Hz), 1.67 (2H, q, *J* = 7.4 Hz), 1.60–1.43 (5H, m), 0.91 (6H, d, *J* = 6.4 Hz); ^{13}C NMR (101 MHz, $CDCl_3$) δ 211.4, 159.0, 129.4, 120.5, 114.4, 67.5, 42.5, 40.8, 32.6, 29.1, 27.7, 25.7, 23.5, 22.3; HRMS (ESI) Found MNa^+ 285.1859, $C_{17}H_{26}NaO$ requires 285.1830.

2-Methyl-10-(phenylthio)decan-5-one (S30)

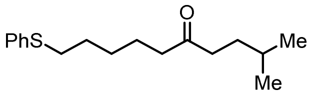

Following **GP3** but adding NaOH (2.0 equiv.), **S20** (200 mg, 0.8 mmol) gave **S30** (160 mg, 72%) as an oil. FT-IR ν_{max} (film)/cm^{-1} 2956, 1710, 1579, 1438, 1264, 1023, 805, 733, 702, 689; ^{1}H NMR (500 MHz, $CDCl_3$) δ 7.45–7.38 (2H, m), 7.25–7.19 (3H, m), 2.83 (2H, t, *J* = 7.3 Hz), 2.34-2.20 (4H, m), 1.57 (2H, q, *J* = 7.5 Hz), 1.50 (2H, q, *J* = 8 Hz), 1.47–1.40 (1H, m), 1.40–1.28 (4H, m), 0.80 (6H, d, *J* = 6.5 Hz); ^{13}C NMR (126 MHz, $CDCl_3$) δ 211.4, 137.1, 129.2, 127.6, 125.8, 42.6, 41.0, 33.5, 32.8, 29.0, 28.4, 27.8, 23.4, 22.4; HRMS (ESI) Found MK^+ 317.1334, $C_{17}H_{26}KOS$ requires 317.1341.

(*R*)-5-((3*R*,5*R*,8*R*,9*S*,10*S*,13*R*,14*S*,17*R*)-3-hydroxy-10,13-dimethylhexadecahydro-1*H*-cyclopenta[*a*]phenanthren-17-yl)hexan-2-one (S31)

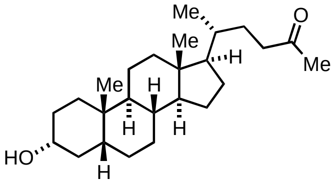

A solution of lithocolic acid (0.5 g, 1.3 mmol, 1.0 equiv.) in THF (15 mL), cooled to 0 ºC and treated with MeLi (4.2 mL, 6.5 mmol, 5.0 equiv., 1.6 M in diethyl ether) by dropwise. The reaction mixture was stirred for 8 h at room temperature, and then quenched with TMSCl (6 mL). The crude was diluted with EtOAc (20 mL), washed with HCl 1M (20 mL) and water (20 mL). The organic layer was dried ($MgSO_4$), filtered and evaporated. The crude was purified by column chromatography on silica gel, eluting with CH_2Cl_2–MeOH (95:5), to give **S31** as an oil (290 mg, 60%). ^{1}H NMR (400 MHz, $CDCl_3$) δ 3.64–3.49 (1H, m), 2.46 – 2.21 (3H, m), 2.09 (3H, s), 1.90 (1H, m), 1.83–1.55 (6H, m), 1.56–1.41 (2H, m), 1.34 (7H, d, *J* = 3.8 Hz), 1.28–1.11 (5H, m), 1.11–0.95 (5H, m), 0.86 (3H, s), 0.84 (3H, d, *J* = 6.5 Hz), 0.58 (3H, s);^{13}C NMR (101 MHz, $CDCl_3$) δ 209.6, 71.3, 56.2, 55.7, 42.4, 41.8, 40.4, 40.1, 39.9, 36.1, 35.5, 35.1, 345.0, 34.3, 30.2, 29.6, 29.5, 27.91, 26.9, 26.1, 23.9, 23.1, 20.5, 18.1, 11.7. Data in accordance with the literature.[13]

2.2 Synthesis of Oximes

General Procedure for the Synthesis of Oximes 1a–u and 6a–w – GP4

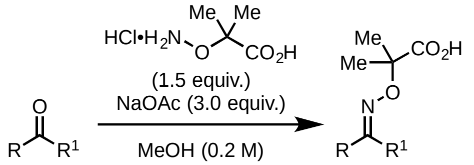

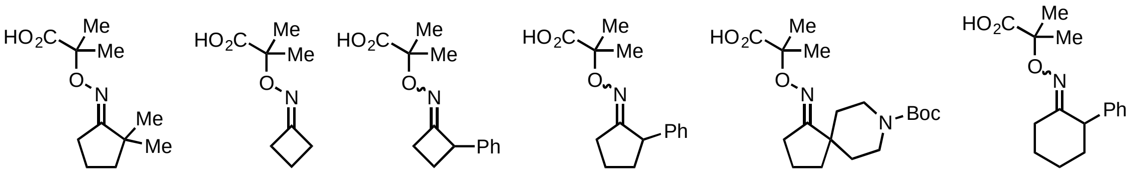

1a **1b** **1c** **1d** **1e** **1f**

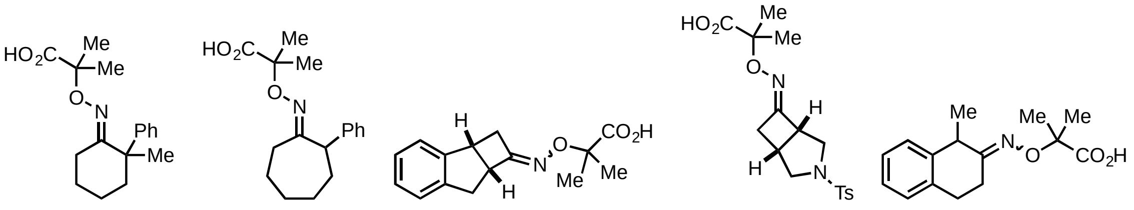

1g **1h** **1i** **1j** **1k**

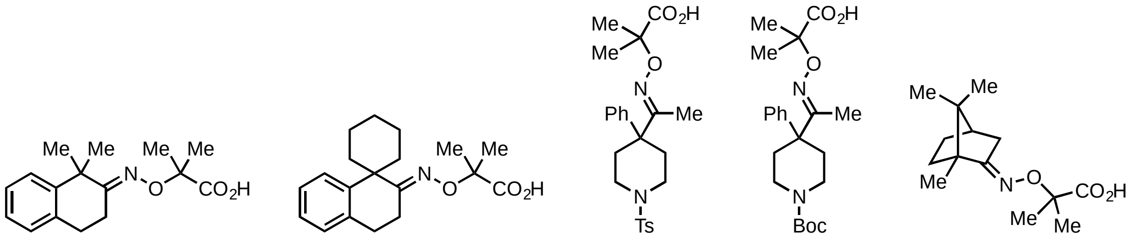

1l **1m** **1n** **1o** **1p**

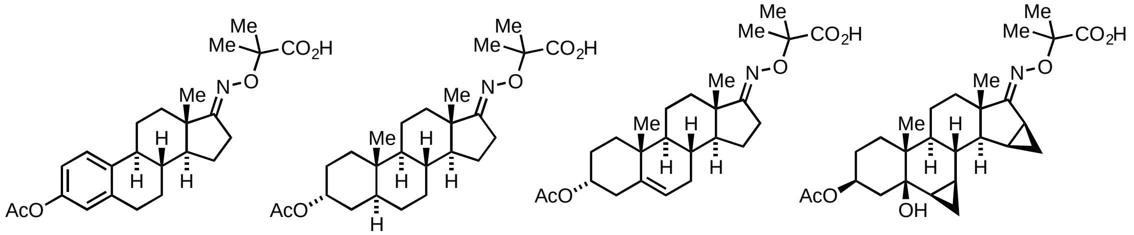

1q **1r** **1s** **1t**

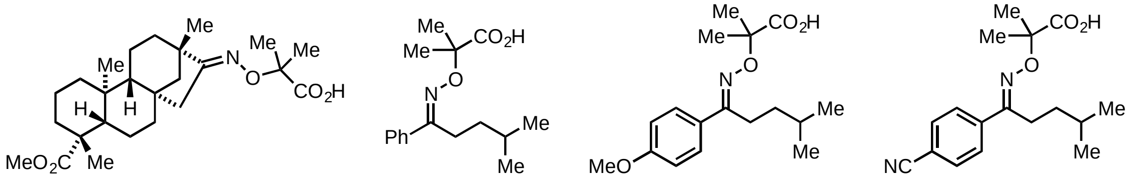

1u **6a** **6b** **6c**

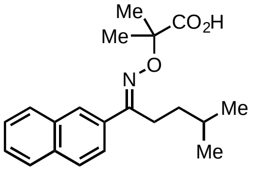

6d

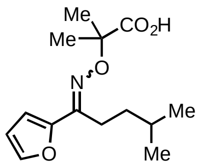

6e

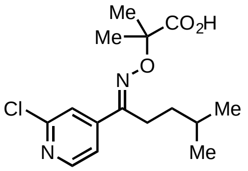

6f

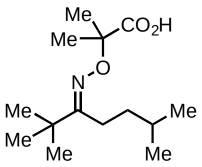

6g

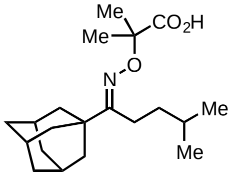

6h

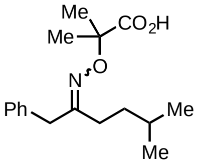

6i

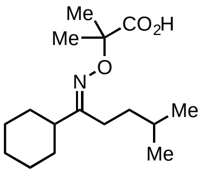

6j

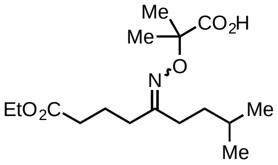

6k

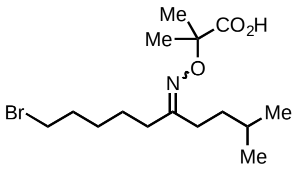

6l

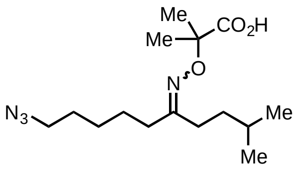

6m

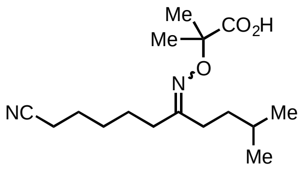

6n

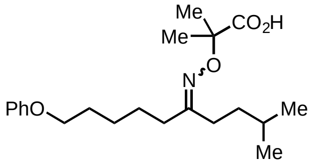

6o

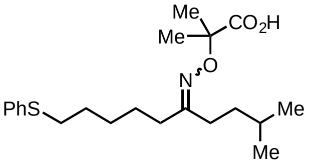

6p

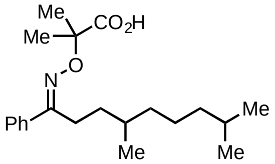

6q

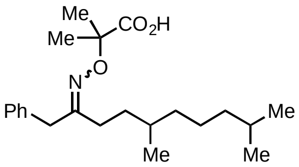

6r

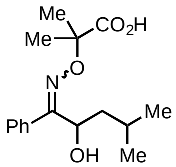

6s

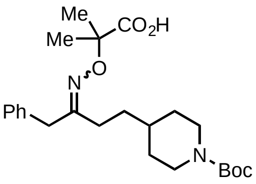

6t

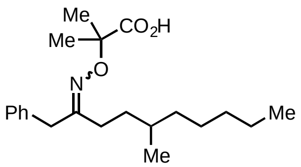

6u

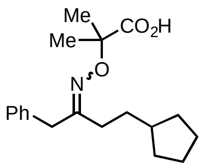

6v

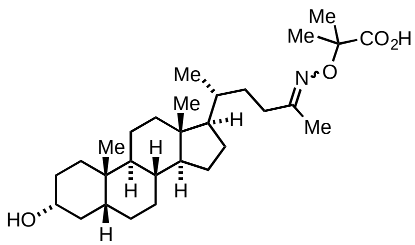

6w

A solution of the ketone (1.0 equiv.) in MeOH (0.2 M) was treated with 1-carboxy-1-methylethoxyammonium chloride (1.5 equiv.), anhydrous NaOAc (3 equiv.) and heated to reflux until complete by TLC analysis (1-6 h). The mixture was allowed to cool to room temperature and an aqueous K_2CO_3 solution was added. The solution was extracted with Et_2O and the organic layer washed with aqueous K_2CO_3 solution (x 2). The combined aqueous extractions were then acidified with conc. HCl solution (30% H_2O) and extracted with CH_2Cl_2 (x 3). The combined organic fractions were dried ($MgSO_4$), filtered and evaporated.

2-(((2,2-Dimethylcyclopentylidene)amino)oxy)-2-methylpropanoic Acid (1a)

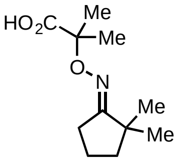

Following **GP4**, 2,2-dimethylcyclopentan-1-one (0.22 mL, 1.79 mmol) gave **1a** (381 mg, quant.) as an oil. FT-IR ν_{max} (film)/cm^{-1} 2960, 1714, 1169, 969, 927, 881; ^{1}H NMR (500 MHz, $CDCl_3$) δ 2.54 (2H, t, *J* = 7.5 Hz), 1.82–1.74 (2H, m), 1.64 (2H, t, *J* = 6.9 Hz), 1.49 (6H, s), 1.16 (6H, s); ^{13}C NMR (126 MHz, $CDCl_3$) δ 176.6, 175.1, 81.1, 42.8, 40.7, 27.7, 26.4, 24.3, 20.8; HRMS (ESI) Found MH$^+$ 214.1434, $C_{11}H_{20}O_3N$ requires 214.1438.

2-((Cyclobutylideneamino)oxy)-2-methylpropanoic Acid (1b)

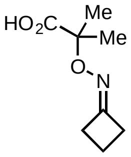

Following **GP4**, cyclobutanone (210 mg, 3 mmol) gave **1b** (180 mg, 35%) as an oil. FT-IR ν_{max} (film)/cm^{-1} 2993, 1715, 1171, 974, 755; ^{1}H NMR (500 MHz, $CDCl_3$) δ 2.95 (4H, dd, *J* = 12.5, 7.8 Hz), 2.03 (2H, p, *J* = 8.1 Hz), 1.50 (6H, s); ^{13}C NMR ($CDCl_3$, 126 MHz) δ 178.2, 162.2, 81.2, 32.2, 31.7, 24.6, 14.9.

2-Methyl-2-(((2-phenylcyclobutylidene)amino)oxy)propanoic Acid (1c)

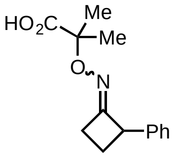

Following **GP4**, 2-phenylcyclobutan-1-one (146 mg, 0.73 mmol) gave **1c** (54 mg, 30%) as an oil. dr: 2.4:1. FT-IR ν_{max} (film)/cm^{-1} 2360, 1735, 1264, 895, 732, 703, 568; ^{1}H NMR (500 MHz, $CDCl_3$, *E*:*Z* isomers) δ 7.36–7.11 (5H, m), 3.05–2.86 (1H, m), 2.56–2.39 (2H, m), 2.09–2.01 (1H, m), 1.50 (4.3H, s), 1.29 (0.8H, s), 1.26 (0.8H, s); ^{13}C NMR (126 MHz, $CDCl_3$, *E*:*Z* isomers)[b] δ 179.6^M, 179.2^m, 162.0^M, 161.2^m, 140.3^M, 139.9^m, 128.5, 128.4, 127.4, 127.0, 126.7, 80.9^M, 80.7^m, 50.1^m, 49.4^M, 29.5^m, 28.9^M, 24.3, 24.2, 24.1; HRMS (APCI) Found M-H$^+$ 246.1136, $C_{14}H_{17}O_3N$ requires 246.1130.

---

[b] In this case not all ^{13}C NMR signals could be assigned between the major (M) and the minor component owing to partial overlap.

2-Methyl-2-(((2-phenylcyclopentylidene)amino)oxy)propanoic Acid (1d)

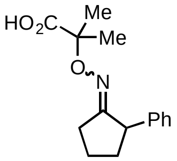

Following **GP4**, 2-phenylcyclopentan-1-one (197 mg, 1.2 mmol) gave **1d** (159 mg, 51%) as an oil. dr 1:1. FT-IR ν_{max} (film)/cm^{-1} 3358, 2970, 1379, 1265, 1160, 1127, 949, 815, 734, 703, 561; ^{1}H NMR (500 MHz, $CDCl_3$, *E*:*Z* isomers) δ 7.32 (2H, t, *J* = 7.5 Hz), 7.27–7.20 (1H, m), 7.19 (2H, d, *J* = 7.4 Hz), 3.79 (1H, t, *J* = 8.1 Hz), 2.87–2.47 (2H, m), 2.35–2.21 (1H, m), 2.07–1.72 (3H, m), 1.48 (1.5H, s), 1.46 (1.5H, s); ^{13}C NMR (126 MHz, $CDCl_3$, *E*:*Z* isomers) δ 175.5, 175.5, 171.8, 141.0, 129.0, 128.2, 127.3, 81.8, 50.2, 35.2, 29.2, 24.9, 24.8, 23.1; HRMS (APCI) Found [M-H]$^-$ 260.1292, $C_{15}H_{19}O_3N$ requires 246.1287.

2-(((8-(*tert*-Butoxycarbonyl)-8-azaspiro[4.5]decan-1-ylidene)amino)oxy)-2-methylpropanoic Acid (1e)

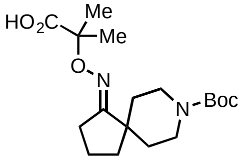

Following **GP4**, *tert*-butyl 1-oxo-8-azaspiro[4.5]decane-8-carboxylate (100 mg, 0.4 mmol) gave **1e** (120 mg, 85%) as a solid. FT-IR ν_{max} (film)/cm^{-1} 2935, 1693, 1426, 1366, 1282, 1249, 1170, 904, 727; ^{1}H NMR (400 MHz, $CDCl_3$) δ 3.66–3.53 (2H, m), 3.37–3.25 (2H, m), 2.54 (2H, t, *J* = 7.0 Hz), 1.79–1.56 (6H, m), 1.47–1.44 (2H, m), 1.46 (6H, s), 1.43 (9H, s); ^{13}C NMR (101 MHz, $CDCl_3$) δ 178.3, 170.8, 155.0, 81.2, 79.5, 44.3, 40.4, 37.8, 34.3, 28.5, 27.7, 24.3, 20.5; HRMS (ESI) Found MK+ 393.1785, $C_{18}H_{30}O_5N_2K$ requires 393.1786.

2-Methyl-2-(((2-phenylcyclohexylidene)amino)oxy)propanoic Acid (1f)

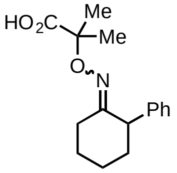

Following **GP4**, 2-phenylcyclohexan-1-one (500 mg, 2.87 mmol) gave **1f** (552 mg, 70%) as an oil. dr 1.5:1. FT-IR ν_{max} (film)/cm^{-1} 3396 (br.), 2978, 2934, 1579, 1402, 1359, 1163, 958, 753, 698; ^{1}H NMR (500 MHz, $CDCl_3$, *E*/*Z* isomers) δ 7.34–7.14 (5H, m), 4.74 (0.4H, br s), 3.56 (0.6H, dd, *J* = 8.4, 6.7 Hz), 2.82–2.71 (0.6H, m), 2.39–2.22 (1.4H, m), 2.16–1.88 (2H,

m), 1.84–1.66 (2H, m), 1.61–1.41 (2H, m), 1.32 (1.2H, s), 1.30 (1.8H, s); ^{13}C NMR (101 MHz, $CDCl_3$) δ 180.7, 180.6, 162.6, 162.3, 141.3, 140.1, 128.6, 128.5, 128.3, 127.4, 126.5, 126.1, 82.3, 82.3, 47.2, 37.7, 33.5, 26.7, 25.9, 25.5, 25.4, 25.1, 24.9, 24.5, 23.8; HRMS (ESI) Found MK^+ 314.1153, $C_{16}H_{21}O_3NK$ requires 314.1153.

(*E*)-2-Methyl-2-(((2-methyl-2-phenylcyclohexylidene)amino)oxy)propanoic Acid (1g)

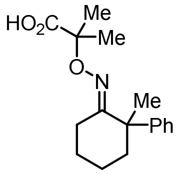

Following **GP4**, 2-methyl-2-phenylcyclohexan-1-one (967 mg, 5.1 mmol) gave **1g** (1.47 g, quant.) as an oil. FT-IR ν_{max} (film)/cm^{-1} 3019, 2940, 1713, 1446, 1215, 970, 920, 874, 877; ^{1}H NMR (400 MHz, $CDCl_3$) δ 7.37–7.31 (2H, m), 7.25–7.19 (3H, m), 3.28–3.20 (1H, m), 2.65–2.57 (1H, m), 1.83–1.74 (1H, m), 1.72–1.56 (3H, m), 1.60 (3H, s), 1.58 (3H, s), 1.56–1.39 (2H, m), 1.32 (3H, s); ^{13}C NMR (101 MHz, $CDCl_3$) δ 176.9, 167.6, 144.6, 128.9, 126.4, 126.2, 81.2, 47.2, 38.3, 29.9, 26.6, 24.5, 24.2, 23.4, 21.9; HRMS (ESI) Found MNa^+ 312.1570, $C_{17}H_{23}O_3NNa$ requires 312.1570

(*E*)-2-Methyl-2-(((2-phenylcycloheptylidene)amino)oxy)propanoic Acid (1h)

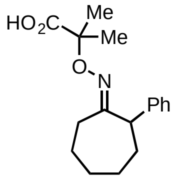

Following **GP4**, 2-phenylcycloheptanone (300 mg, 1.6 mmol) gave **1h** (351 mg, 76%) as an oil. ^{1}H NMR (500 MHz, $CDCl_3$) δ 7.34–7.30 (2H, m), 7.25–7.19 (3H, m), 3.79 (1H, dd, J = 10.7, 5.4 Hz), 2.93 (1H, ddd, J = 14.2, 6.7, 2.6 Hz), 2.33–2.25 (1H, m), 2.16 (1H, ddd, J = 14.3, 11.9, 2.7 Hz), 2.00–1.87 (3H, m), 1.58 (2H, dt, J = 13.3, 4.1 Hz), 1.47 (6H, s), 1.41–1.35 (2H, m); ^{13}C NMR (126 MHz, $CDCl_3$) δ 175.9, 168.1, 141.6, 128.6, 127.2, 126.8, 81.2, 49.4, 32.2, 30.7, 27.4, 26.9, 25.4, 24.4, 24.3; HRMS (APCI) Found MH^+ 290.1754, $C_{17}H_{24}O_3N$ requires 290.1751.

2-Methyl-2-(((2,2a,7,7a-tetrahydro-1H-cyclobuta[a]inden-1-ylidene)amino)oxy) propanoic Acid (1i)

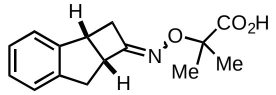

Following **GP4**, **S1** (400 mg, 2.5 mmol) gave **1i** (537 mg, 83%) as an oil. dr 1.4:1. FT-IR ν_{max} (film)/cm^{-1} 2970, 1264, 732, 703, 564; ^{1}H NMR ($CDCl_3$, 400 MHz, *E*:*Z* isomers) δ 7.33–7.18 (4H, m), 4.08–3.87 (2H, m), 3.57 (0.4H, d, *J* = 16.8 Hz), 3.48–3.38 (1H, m), 3.32–3.22 (1.6H. m), 2.85 (0.6H, dt, *J* = 17.8, 3.3 Hz), 2.74 (0.4H, dt, *J* = 16.8, 3.3 Hz), 1.59 (1.3H, s), 1.56 (1.3H, s), 1.51 (1.7H, s), 1.50 (1.7H, s); ^{13}C NMR ($CDCl_3$, 126 MHz, *E*:*Z* isomers); δ 179.6^m, 179.5^M, 163.0^M, 161.6^m, 144.7^m, 144.6^M, 143.2^m, 143.1^M, 127.0^M, 127.0^m, 126.9 ^{M}, 126.9^M, 124.9^M, 124.6^m, 124.8^M, 124.7^m, 80.5^M, 80.5^m, 47.4^M, 47.1^m, 40.5 ^{M}, 39.6^m, 39.6^m, 39.4^M, 37.0^M, 34.7^m, 24.1^M, 23.5^m, 24.0^m, 23.8^M; HRMS (ESI) Found MH$^+$ 259.1214, $C_{15}H_{17}O_3N$ requires 259.1208.

2-Methyl-2-(((3-tosyl-3-azabicyclo[3.2.0]heptan-6-ylidene)amino)oxy)propanoic Acid (1j)

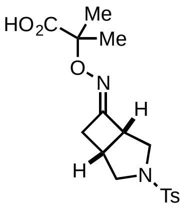

Following **GP4**, 3-tosyl-3-azabicyclo[3.2.0]heptan-6-one (100 mg, 0.38 mmol) gave **1j** (120 mg, 86%) as a solid. FT-IR ν_{max} (film)/cm^{-1} 3334, 2930, 1264, 733, 703, 570; ^{1}H NMR (500 MHz, $CDCl_3$) δ 7.70 (2H, d, *J* = 8.0 Hz), 7.34 (2H, d, *J* = 8.0 Hz), 3.96 (1H, d, *J* = 9.9 Hz), 3.66 (1H, s), 3.57 (1H, d, *J* = 10.0 Hz), 3.11 (1H, dd, *J* = 17.5, 8.6 Hz), 2.91–2.84 (1H, m), 2.76–2.69 (2H, m), 2.66 (1H, dd, *J* = 9.8, 7.5 Hz), 2.44 (3H, s), 1.52 (3H, s), 1.50 (3H, s); ^{13}C NMR (126 MHz, $CDCl_3$) δ 175.8, 159.0, 144.0, 131.7, 129.7, 128.1, 81.3, 50.2, 47.7, 35.7, 32.3, 31.0, 24.1, 21.6; HRMS (ASAP) Found MH$^+$ 367.1322, $C_{17}H_{23}O_5N_2S$ requires 367.1322.

2-Methyl-2-(((1-methyl-3,4-dihydronaphthalen-2(1H)-ylidene)amino)oxy)propanoic Acid (1k)

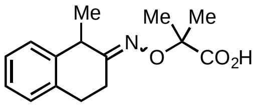

Following **GP4**, 1-methyl-3,4-dihydronaphthalen-2(1H)-one (145 mg, 0.9 mmol) gave **1k** (106 mg, 46%) as an oil. dr 1.8:1. FT-IR ν_{max} (film)/cm^{-1} 3327, 2970, 2830, 2360, 1466, 1379, 1161, 1128, 1029, 951, 817; ^{1}H NMR (500 MHz, $CDCl_3$, *E*:*Z* isomers) δ 7.22–6.98 (4H, m), 4.23 (0.3H, q, *J* = 7.2 Hz), 3.56 (0.7H, q, *J* = 7.0 Hz), 2.99–2.38 (4H, m), 1.43 (6H, s), 1.38 (2H, d, *J* = 7.1 Hz), 1.35 (1H, d, *J* = 7.2 Hz); ^{13}C NMR (126 MHz, $CDCl_3$, *E*:*Z* isomers)[b] δ 178.6^{M}, 178.5^{m}, 163.6 ^{M}, 163.4^{m}, 157.03, 139.1^{m}, 139.1^{M}, 137.1^{M}, 135.6^{m}, 128.2^{m}, 127.4^{m}, 128.1^{M}, 126.6^{M}, 126.7^{m}, 126.4^{M}, 126.0 ^{M}, 126.0^{m}, 80.8, 80.5, 80.4, 38.9, 33.4, 28.8, 27.2, 26.8, 24.0, 24.0, 20.9^{M}, 20.8^{m}, 17.9; HRMS (APCI) Found MH^{+} 260.1292, $C_{15}H_{19}O_3N$ requires 260.1287.

2-(((1,1-Dimethyl-3,4-dihydronaphthalen-2(1H)-ylidene)amino)oxy)-2-methylpropanoic Acid (1l)

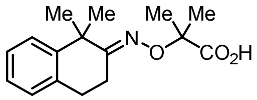

Following **GP4**, **S2** (270 mg, 1.26 mmol) gave **1l** (347 mg, quant) as a solid. FT-IR ν_{max} (film)/cm^{-1} 2982, 1712, 1171, 967, 922, 895, 757; ^{1}H NMR (400 MHz, $CDCl_3$) δ 7.41 (1H, d, *J* = 7.7 Hz), 7.31–7.26 (1H, m), 7.21 (1H, td, *J* = 7.3, 1.2 Hz), 7.17 (1H, d, *J* = 6.6 Hz), 2.99 (2H, t, *J* = 6.6 Hz), 2.92–2.87 (2H, m), 1.56 (6H, s), 1.55 (6H, s); ^{13}C NMR (126 MHz, $CDCl_3$) δ 175.7, 168.7, 142.8, 135.8, 128.4, 127.1, 126.5, 125.1, 81.5, 41.4, 27.9, 27.6, 24.4, 22.9; HRMS (APCI) Found MH^{+} 276.1584, $C_{16}H_{22}O_3N$ requires 276.1594.

2-(((3',4'-Dihydro-2'H-spiro[cyclohexane-1,1'-naphthalen]-2'-ylidene)amino)oxy)-2-methylpropanoic Acid (1m)

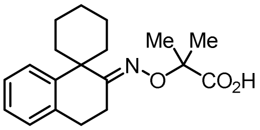

Following **GP4**, **S3** (460 mg, 2.15 mmol) gave **1m** (481 mg, 71%) as a solid. FT-IR ν_{max} (film)/cm^{-1} 2926, 1712, 1169, 966, 928, 874, 752; ^{1}H NMR (400 MHz, $CDCl_3$) δ 7.43 (1H, d, *J* = 7.8 Hz), 7.26–7.21 (1H, m), 7.16 (1H, td, *J* = 7.4, 1.0 Hz), 7.11 (1H, d, *J* = 7.2 Hz), 3.01

(2H, t, J = 7.0 Hz), 2.86 (2H, t, J = 7.0 Hz), 2.16–2.10 (2H, m), 1.86–1.77 (2H, m), 1.76–1.61 (5H, m), 1.54 (6H, s), 1.39–1.29 (1H, m); ^{13}C NMR (126 MHz, $CDCl_3$) δ 178.4, 165.3, 144.6, 136.5, 128.6, 126.6, 126.1, 125.4, 81.1, 45.5, 35.1, 29.0, 26.2, 24.3, 23.1, 21.7; HRMS (APCI) Found MH^+ 316.1901, $C_{19}H_{26}O_3N$ requires 316.1907.

2-Methyl-2-(((1-(4-phenyl-1-tosylpiperidin-4-yl)ethylidene)amino)oxy)propanoic Acid (1n)

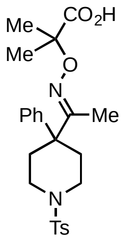

Following **GP4**, **S4** (432 mg, 1.2 mmol) gave **1n** (478 mg, 87%) as a solid. FT-IR ν_{max} (film)/cm^{-1} 3022, 1715, 1446, 1352, 1214, 1118, 971, 924, 753, 667, 659, 587, 573; ^{1}H NMR (400 MHz, $CDCl_3$) δ 7.67 (2H, d, J = 7.6 Hz,), 7.39–7.29 (4H, m), 7.29–7.18 (3H, m), 3.60 (2H, d, J = 11.0 Hz), 2.67 (2H, t, J = 11.2 Hz), 2.45 (3H, s), 2.38–2.28 (2H, m), 2.12–2.01 (2H, m), 1.49 (3H, s), 1.40 (6H, s); ^{13}C NMR (101 MHz, $CDCl_3$) δ 177.8, 159.6, 143.7, 143.5, 133.2, 129.7, 128.8, 127.9, 127.1, 126.0, 81.1, 46.8, 43.2, 32.9, 24.0, 21.5, 12.2; HRMS (ESI neg) Found M-H^+ 457.1803, $C_{24}H_{29}O_5N_2S$ requires 457.1797.

2-(((1-(1-(*tert*-Butoxycarbonyl)-4-phenylpiperidin-4-yl)ethylidene)amino)oxy)-2-methylpropanoic Acid (1o)

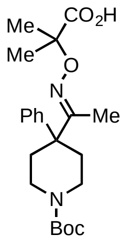

Following **GP4**, **S5** (432 mg, 1.2 mmol) gave **1o** (494 mg, quant.) as a solid. FT-IR ν_{max} (film)/cm^{-1} 3015, 1682, 1435, 1355, 1215, 1173, 696, 859, 751, 700, 573; ^{1}H NMR (400 MHz, $CDCl_3$) δ 9.68–9.33 (1H, br. s), 7.36–7.26 (4H, m), 7.23 (1H, t, J = 6.9 Hz), 3.99–3.69 (2H, br. s), 3.27–3.06 (1H, br. s), 2.32–2.19 (2H, m), 1.97–1.76 (2H, m), 1.61 (6H, s), 1.60 (3H, s), 1.46 (9H, s); ^{13}C NMR (101 MHz, $CDCl_3$) δ 178.6, 159.5, 155.1, 144.3, 128.7, 126.8, 126.2, 81.2, 79.6, 47.5, 40.8, 33.4, 28.5, 24.2, 12.0; HRMS (ESI) found MNa^+ 427.2203, $C_{22}H_{32}O_5N_2Na$ requires 427.2203.

2-Methyl-2-((((1*S*,4*R*,*E*)-1,7,7-trimethylbicyclo[2.2.1]heptan-2-ylidene)amino)oxy) propanoic Acid (1p)

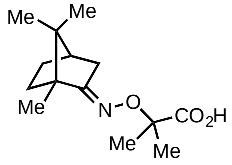

Following **GP4**, (*D*)-camphor (400 mg, 2.6 mmol) gave **1p** (256 mg, 39%) as an oil. dr 1:1. FT-IR ν_{max} (film)/cm^{-1} 2958, 1714, 1174, 968, 921, 885, 757; ^{1}H NMR (500 MHz, $CDCl_3$, *E*:*Z* isomers) δ 2.56–2.53 (1H, m), 2.08 (0.5H, s), 2.04 (0.5H, s), 1.98–1.93 (1H, m), 1.91–1.82 (1H, m), 1.76 (1H, td, *J* = 12.4, 3.9 Hz), 1.49 (3H, s), 1.47 (3H, s), 1.45–1.39 (1H, m), 1.29–1.22 (1H, m), 1.02 (3H, s), 0.94 (3H, s), 0.79 (3H, s); ^{13}C NMR (126 MHz, $CDCl_3$, *E*:*Z* isomers) δ 175.7, 174.3, 81.0, 52.8, 48.5, 43.5, 34.0, 32.4, 27.1, 24.5, 24.3, 19.4, 18.4, 11.0; HRMS (APCI) Found MH$^+$ 254.1747, $C_{14}H_{24}O_3N$ requires 254.1751.

2-((((8*R*,9*S*,13*S*,14*S*,*E*)-3-Acetoxy-13-methyl-6,7,8,9,11,12,13,14,15,16-decahydro-17*H*-cyclopenta[*a*]phenanthren-17-ylidene)amino)oxy)-2-methylpropanoic Acid (1q)

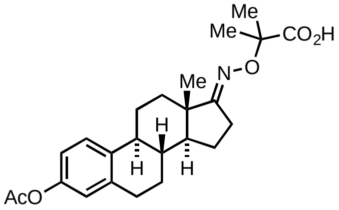

Following **GP4**, estrone (420 mg, 1.56 mmol) gave **1q** after acetylation[c] (432 mg, 67%), as a solid. FT-IR ν_{max} (film)/cm^{-1} 2979, 2359, 2253, 1746, 1470, 1127, 903, 724, 650; ^{1}H NMR (400 MHz, $CDCl_3$) δ 7.29 (1H, d, *J* = 8.5 Hz), 6.85 (1H, dd, *J* = 8.5, 2.6 Hz), 6.80 (1H, d, *J* = 2.5 Hz), 2.93–2.83 (2H, m), 2.67–2.48 (2H, m), 2.44–2.35 (1H, m), 2.29 (3H, s), 2.00–1.89 (2H, m), 1.68–1.38 (8H, m), 1.50 (6H, d, *J* = 5.7 Hz), 0.97 (3H, s); ^{13}C NMR (101 MHz, $CDCl_3$) δ 176.3, 174.2, 169.9, 148.5, 138.0, 137.4, 126.4, 121.6, 118.8, 81.1, 45.0, 44.1, 37.7, 33.9, 29.4, 27.0, 26.2, 25.9, 24.4, 24.4, 22.9, 21.2, 17.2; HRMS (APCI) Found MH$^+$ 414.2284, $C_{24}H_{32}O_5N$ requires 414.2275.

---

[c] Experimental procedure for the acetylation: A suspension of the crude in H_2O (2.0 mL) was cooled to 0 ºC and treated with NaOH (82 mg, 2.04 mmol, 3.0 equiv.) and Ac_2O (0.18 mL, 2.04 mmol, 3.0 equiv.). The mixture was allowed to warm to room temperature overnight. The mixture was diluted with 1M HCl and EtOAc. The layers were separated and the aqueous layer was extracted with EtOAc (3 x 5mL). The combined organic layers were dried ($MgSO_4$), filtered and evaporated. Purification by column chromatography on silica gel gave **1q**.

2-((((3*R*,8*R*,9*S*,10*S*,13*S*,14*S*,*E*)-3-Acetoxy-10,13-dimethylhexadecahydro-17H-cyclopenta[a]phenanthren-17-ylidene)amino)oxy)-2-methylpropanoic Acid (1r)

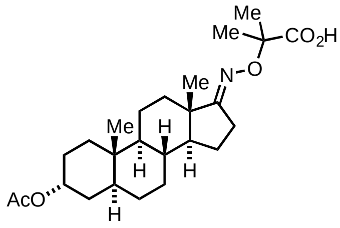

Following **GP4**, **S6** (200 mg, 0.6 mmol) gave **1r** (211 mg, 65%) as a solid. FT-IR ν_{max} (film)/cm^{-1} 2945, 1724, 1443, 1355, 1245, 1026, 875, 754; ^{1}H NMR (400 MHz, $CDCl_3$) δ 4.74–4.61 (1H, m), 2.60–2.39 (2H, m), 2.02 (3H, s), 1.94–1.87 (1H, m), 1.87–1.77 (2H, m), 1.77–1.68 (2H, m), 1.67–1.57 (2H, m), 1.55–1.42 (2H, m), 1.48 (3H, s), 1.46 (3H, s), 1.41–1.26 (6H, m), 1.25–1.13 (2H, m), 1.08–0.94 (2H, m), 0.91 (3H, s), 0.85 (3H, s), 0.78–0.69 (1H, m); ^{13}C NMR (126 MHz, $CDCl_3$) δ 175.5, 175.0, 170.7, 81.2, 73.5, 54.3, 45.0, 44.6, 36.7, 35.6, 34.8, 33.9, 33.8, 31.4, 28.3, 27.4, 26.2, 24.4, 24.4, 23.1, 21.5, 20.6, 17.2, 12.2; HRMS (ASAP) Found MH$^+$ 434.2886, $C_{25}H_{40}O_5N$ requires 434.2901.

2-((((3*R*,8*R*,9*S*,10*R*,13*S*,14*S*,*E*)-3-acetoxy-10,13-dimethyl-1,2,3,4,7,8,9,10,11,12,13, 14,15,16-tetradecahydro-17H-cyclopenta[a]phenanthren-17-ylidene)amino)oxy)-2-methylpropanoic Acid (1s)

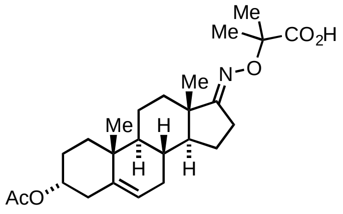

Following **GP4** but running the reaction overnight at room temperature, (3R,8R,9S,10R,13S,14S)-10,13-dimethyl-17-oxo-2,3,4,7,8,9,10,11,12,13,14,15,16,17-tetra decahydro-1H-cyclopenta[a]phenanthren-3-yl acetate (400 mg, 1.2 mmol) gave **1s** (436 mg, 84%) as a solid. FT-IR ν_{max} (film)/cm^{-1} 2947, 1725, 1374, 1251, 1032, 754; ^{1}H NMR (400 MHz, $CDCl_3$) δ 9.86 (1H, br s), 5.32 (1H, d, *J* = 4.9 Hz), 4.54 (1H, dt, *J* = 10.3, 5.5 Hz), 2.59–2.35 (2H, m), 2.35–2.17 (2H, m), 2.05–1.95 (1H, m), 1.99 (3H, s), 1.90 (1H, d, *J* = 11.7 Hz), 1.86–1.71 (3H, m), 1.64–1.46 (4H, m), 1.44 (3H, s), 1.43 (3H, s), 1.47–1.26 (3H, m), 1.20–1.03 (2H, m), 0.99 (3H, s), 1.04–0.93 (1H, m), 0.87 (3H, s); ^{13}C NMR (101 MHz, $CDCl_3$) δ 177.7, 173.0, 170.6, 139.8, 122.0, 80.8, 77.5, 77.1, 76.8, 73.8, 53.9, 50.1, 44.3, 38.0, 36.89, 36.7, 33.8, 31.3, 31.2, 27.6, 26.0, 24.3, 24.2, 23.2, 21.4, 20.4, 19.3, 16.9; HRMS (APCI) found MH$^+$ 432.2739, $C_{25}H_{38}O_5N$ requires 432.2744.

2-((((2S,4a*R*,4b*S*,6a*S*,7a*S*,8a*S*,8b*S*,8c*R*,8d*R*,9a*R*,9b*R*,*E*)-2-Acetoxy-9b-hydroxy-4a,6a-dimethyloctadecahydro-7H-cyclopropa[4,5]cyclopenta[1,2-a]cyclopropa[l]phenanthren-7-ylidene)amino)oxy)-2-methylpropanoic Acid (1t)

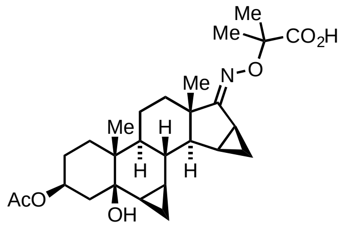

Following **GP4**, **S7** (210 mg, 0.44 mmol) gave **1t** (260 mg, quant.) as a solid. FT-IR ν_{max} (film)/cm^{-1} 3018, 1730, 1443, 1352, 1215, 1144, 1040, 753, 668, 567; ^{13}C NMR (126 MHz, $CDCl_3$) δ 175.5, 173.8, 169.9, 81.3, 72.9, 70.5, 54.5, 41.4, 40.8, 40.4, 37.2, 33.3, 24.6, 24.4, 24.4, 22.7, 22.6, 21.7, 21.5, 18.9, 16.4, 16.3, 14.9, 14.4, 11.4; HRMS (ASAP) Found MH$^+$ 474.2843, $C_{27}H_{40}O_6N$ requires 474.2850.

2-((((4*R*,6a*S*,9*R*,11a*R*,11b*S*,*Z*)-4-(Methoxycarbonyl)-4,9,11b-trimethyldodecahydro-6a,9-methanocyclohepta[a]naphthalen-8(7H)-ylidene)amino)oxy)-2-methylpropanoic Acid (1u)

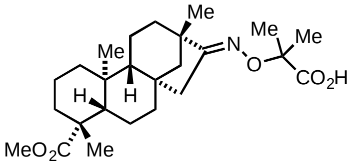

Following **GP4**, **S8** (332 mg, 1.0 mmol) gave **1u** (210 mg, 48%) as a solid; FT-IR ν_{max} (film)/cm^{-1} 1710, 1265, 1223, 735, 703, 545; ^{1}H NMR (400 MHz, $CDCl_3$) δ 3.65 (3H, s), 2.94 (1H, dd, *J* = 19.0, 3.3 Hz), 2.24–2.13 (1H, m), 1.96 (1H, d, *J* = 19.0 Hz), 1.91–1.76 (2H, m), 1.76–1.54 (5H, m), 1.49 (6H, s), 1.49–1.36 (3H, m), 1.29–1.21 (3H, m), 1.18 (3H, s), 1.10 (3H, s), 1.09–0.96 (2H, m), 0.92–0.80 (2H, m), 0.72 (3H, s); ^{13}C NMR (101 MHz, $CDCl_3$) δ 178.0, 176.3, 174.3, 81.6, 57.5, 56.5, 55.1, 51.8, 45.0, 44.2, 41.4, 41.1, 40.3, 40.0, 38.4, 38.4, 38.3, 29.3, 24.9, 24.8, 22.5, 22.1, 20.8, 19.4, 13.6; HRMS (APCI) Found MH$^+$ 434.2899, $C_{25}H_{39}NO_5$ requires 434.2906.

2-((((8*R*,9*S*,13*S*,14*S*,*E*)-3-Methoxy-13-methyl-6,7,8,9,11,12,13,14,15,16-decahydro-17*H*-cyclopenta[*a*]phenanthren-17-ylidene)amino)oxy)-2-methylpropanoic Acid (1v)

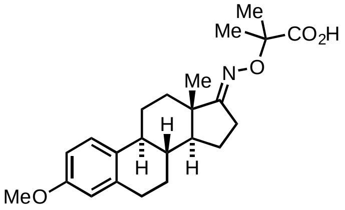

Following **GP4**, mestrone (70 mg, 0.25 mmol) gave **1v** (93 mg, quant.) as a solid. FT-IR ν_{max} (film)/cm^{-1} 2929, 1716, 1499, 1169, 919, 753; ^{1}H NMR (400 MHz, $CDCl_3$) δ 7.20 (1H, d, *J* = 8.6 Hz), 6.72 (1H, dd, *J* = 8.6, 2.6 Hz), 6.64 (1H, d, *J* = 2.4 Hz), 3.78 (3H, s), 2.93–2.85 (2H, m), 2.67–2.47 (2H, m), 2.43–2.35 (1H, m), 2.29 (1H, m), 2.08 (1H, m), 2.00–1.91 (1H, m), 1.69–1.39 (5H, m), 1.51 (6H, d, *J* = 5.3 Hz), 0.97 (3H, s); ^{13}C NMR (101 MHz, $CDCl_3$) δ 176.0, 174.5, 157.6, 137.7, 132.0, 126.3, 113.9, 111.6, 81.16, 55.2, 52.8, 45.1, 43.9, 38.1, 33.9, 29.7, 27.2, 26.3, 26.1, 24.4, 24.4, 22.9, 17.3; HRMS (ESI) Found MH$^+$ 386.2322, $C_{23}H_{32}O_4N$ requires 386.2326.

(*E*)-2-Methyl-2-(((4-methyl-1-phenylpentylidene)amino)oxy)propanoic Acid (6a)

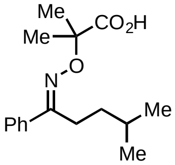

Following **GP4**, **S9** (654 mg, 3.70 mmol) gave **6a** (890 mg, 87%) as a solid. FT-IR ν_{max} (film)/cm^{-1} 2955, 2869, 1714, 1468, 1295, 1169, 978, 920, 903, 764, 692; ^{1}H NMR (400 MHz, $CDCl_3$) δ 7.63–7.58 (2H, m), 7.41–7.35 (3H, m), 2.83–2.75 (2H, m), 1.66–1.56 (1H, m), 1.60 (6H, s), 1.47–1.39 (2H, m), 0.94 (6H, d, *J* = 6.6 Hz); ^{13}C NMR (126 MHz, $CDCl_3$) δ 177.3, 161.4, 135.0, 129.7, 128.6, 126.5, 81.6, 35.4, 28.3, 24.9, 24.3, 22.3; HRMS (APCI) Found MH$^+$ 278.1746, $C_{16}H_{24}O_3N$ requires 278.1751.

(*E*)-2-(((1-(4-Methoxyphenyl)-4-methylpentylidene)amino)oxy)-2-methylpropanoic Acid (6b)

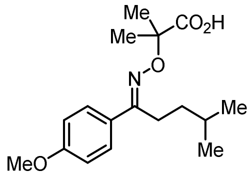

Following **GP4**, **S10** (413 mg, 2.0 mmol) gave **6b** (436 mg, 71%) as a solid. FT-IR ν_{max} (film)/cm^{-1} 2955, 1713, 1607, 1513, 1466, 1297, 1249, 1174, 977, 830; ^{1}H NMR (500 MHz, $CDCl_3$) δ 7.56 (2H, d, *J* = 8.5 Hz), 6.90 (2H, d, *J* = 8.5 Hz), 3.83 (3H, s), 2.79–2.74 (2H, m), 1.66–1.56 (1H, m), 1.58 (6H, s), 1.42 (2H, m), 0.94 (6H, d, *J* = 6.6 Hz); ^{13}C NMR (126 MHz, $CDCl_3$) δ 177.4, 161.2, 161.0, 127.9, 127.4, 114.1, 81.6, 55.5, 35.6, 28.4, 24.9, 24.5, 22.5; HRMS (APCI) Found MH$^+$ 308.1853, $C_{17}H_{26}O_4N$ requires 308.1856.

(*E*)-2-(((1-(4-Cyanophenyl)-4-methylpentylidene)amino)oxy)-2-methylpropanoic Acid (6c)

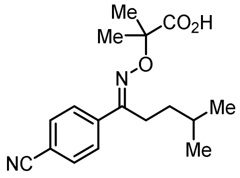

Following **GP4**, **S11** (273 mg, 1.36 mmol) gave **6c** (387 mg, 94%) as a solid. FT-IR ν_{max} (film)/cm^{-1} 2955, 1716, 1612, 1302, 1250, 974, 821; ^{1}H NMR (500 MHz, $CDCl_3$) δ 7.72 (2H, d, *J* = 8.1 Hz), 7.65 (2H, d, *J* = 8.1 Hz), 2.80–2.75 (2H, m), 1.62 (6H, s), 1.64–1.56 (1H, m), 1.42–1.40 (2H, m), 0.94 (6H, d, *J* = 6.6 Hz); ^{13}C NMR (126 MHz, $CDCl_3$) δ 176.5, 158.9, 139.5, 132.3, 126.9, 118.5, 112.9, 82.0, 35.2, 28.3, 24.4, 24.2, 22.3; HRMS (APCI) MH$^+$ 303.1691, $C_{17}H_{23}O_3N_2$ requires 303.1703.

(*E*)-2-Methyl-2-(((4-methyl-1-(naphthalen-2-yl)pentylidene)amino)oxy)propanoic Acid (6d)

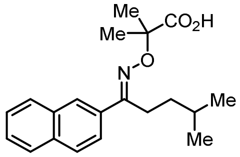

Following **GP4**, **S12** (367 mg, 1.62 mmol) gave **6d** (491 mg, 93%) as a solid. FT-IR ν_{max} (film)/cm^{-1} 2956, 1715, 1467, 1170, 980, 750; ^{1}H NMR (400 MHz, $CDCl_3$) δ 8.01 (1H, br s),

7.90–7.82 (3H, m), 7.80–7.77 (1H, m), 7.55–7.51 (2H, m), 2.98–2.89 (2H, m), 1.73–1.62 (1H, m), 1.64 (6H, s), 1.55–1.46 (2H, m), 0.97 (6H, d, *J* = 6.6 Hz); ^{13}C NMR (126 MHz, $CDCl_3$) δ 175.6, 161.0, 133.9, 133.1, 132.4, 128.6, 128.3, 127.7, 126.9, 126.5, 126.3, 123.7, 81.9, 35.5, 28.3, 24.8, 24.4, 22.4; HRMS (APCI) Found MH^+ 328.1913, $C_{20}H_{26}O_3N$ requires 328.1907.

2-(((1-(Furan-2-yl)-4-methylpentylidene)amino)oxy)-2-methylpropanoic Acid (6e)

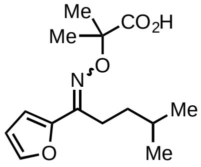

Following **GP4**, **S13** (374 mg, 2.25 mmol) gave **6e** (507 mg, 84%) as an oil. dr1:1. FT-IR ν_{max} (film)/cm^{-1} 2955, 1714, 1468, 1383, 1365, 1161, 979, 751; ^{1}H NMR (400 MHz, $CDCl_3$, *E*:*Z* isomers) δ 7.51 (0.5H, dd, *J* = 1.7, 0.5 Hz), 7.50–7.49 (0.5H, m), 7.36–7.35 (0.5H, m), 6.72 (0.5H, dd, *J* = 3.5, 0.5 Hz), 6.55 (0.5H, dd, *J* = 3.5, 1.8 Hz), 6.47 (0.5H, dd, *J* = 3.5, 1.8 Hz), 2.73–2.66 (2H, m), 1.62 (3H, s), 1.58 (3H, s), 1.66–1.43 (3H, m), 0.95 (3H, d, *J* = 6.6 Hz), 0.94 (3H, d, *J* = 6.6 Hz); ^{13}C NMR (126 MHz, $CDCl_3$, *E*:*Z* isomers) δ 176.4, 153.5, 149.4, 148.9, 145.1, 144.3, 143.2, 119.3, 112.2, 111.5, 111.4, 81.9, 81.8, 36.3, 35.7, 31.0, 30.0, 28.2, 27.7, 27.1, 24.5, 24.4, 24.3, 22.4, 22.3; HRMS (APCI) Found MH^+ 268.1544, $C_{14}H_{22}O_4N$ requires 268.1543.

2-(((1-(2-Chloropyridin-4-yl)-4-methylpentylidene)amino)oxy)-2-methylpropanoic Acid (6f)

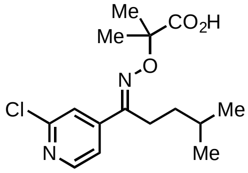

Following **GP4**, **S14** (128 mg, 0.57 mmol) gave **6f** (130 mg, 74%) as an oil. FT-IR ν_{max} (film)/cm^{-1} 2958, 1720, 1588, 1379, 1215, 1168, 986, 755; ^{1}H NMR (500 MHz, $CDCl_3$) δ 8.38 (1H, d, *J* = 5.2 Hz), 7.51 (1H, s), 7.41 (1H, d, *J* = 5.2 Hz), 2.76–2.70 (2H, m), 1.62 (6H, s), 1.66–1.56 (1H, m), 1.42–1.38 (2H, m), 0.95 (6H, d, *J* = 6.6 Hz); ^{13}C NMR (126 MHz, $CDCl_3$) δ 176.1, 157.0, 152.2, 150.0, 146.0, 121.1, 119.3, 82.3, 35.0, 28.2, 24.4, 22.3; HRMS (APCI) Found MH^+ 313.1305, $C_{15}H_{22}O_3N_2Cl$ requires 313.1313.

(*E*)-2-Methyl-2-(((2,2,6-trimethylheptan-3-ylidene)amino)oxy)propanoic Acid (6g)

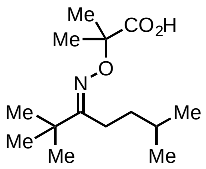

Following **GP4**, **S15** (300 mg, 1.9 mmol) gave **6g** (155 mg, 31%) as a solid. FT-IR ν_{max} (film)/cm^{-1} 2958, 2870, 1716, 1468, 1364, 1175, 974, 898, 758; ^{1}H NMR (400 MHz, $CDCl_3$) δ 11.50 (1H, br s), 2.34–2.27 (2H, m), 1.64–1.54 (1H, m), 1.50 (6H, s), 1.43–1.35 (2H, m), 1.16 (9H, s), 0.93 (6H, d, *J* = 6.6 Hz); ^{13}C NMR (101 MHz, $CDCl_3$) δ 175.6, 172.1, 81.1, 38.2, 35.3, 28.9, 27.6, 24.6, 24.4, 22.2; HRMS (APCI) Found MH^{+} 258.2059, $C_{14}H_{28}O_3N$ requires 258.2064.

2-((((*E*)-1-((3*r*,5*r*,7*r*)-Adamantan-1-yl)-4-methylpentylidene)amino)oxy)-2-methyl propanoic Acid (6h)

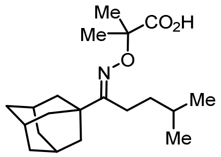

Following **GP4**, **S16** (230 mg, 0.98 mmol) gave **6h** (230 mg, 62%) as a solid. FT-IR ν_{max} (film)/cm^{-1} 2903, 2849, 1711, 1451, 1291, 1173, 970, 918, 900; ^{1}H NMR (400 MHz, $CDCl_3$) δ 2.31–2.25 (2H, m), 2.06 (3H, br s), 1.81–1.64 (12H, m), 1.62–1.52 (1H, m), 1.50 (6H, s), 1.39–1.31 (2H, m), 0.92 (6H, d, *J* = 6.6 Hz); ^{13}C NMR (126 MHz, $CDCl_3$) δ 175.6, 172.1, 81.1, 40.1, 39.3, 38.6, 36.5, 36.4, 35.3, 28.9, 27.9, 27.8, 24.4, 23.5, 22.2; HRMS (APCI) Found MH^{+} 336.2524, $C_{20}H_{34}O_3N$ requires 336.2533.

2-Methyl-2-(((5-methyl-1-phenylhexan-2-ylidene)amino)oxy)propanoic Acid (6i)

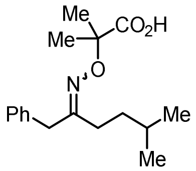

Following **GP4**, **S17** (600 mg, 3.16 mmol) gave **6i** (920 mg, quant.) as a solid. dr 1:1. FT-IR ν_{max} (film)/cm^{-1} 2955 (br.), 2869, 1714, 1468, 1453, 1296, 1169, 983, 968, 908, 733, 700; ^{1}H NMR (400 MHz, $CDCl_3$, *E*:*Z* isomers) δ 7.35–7.28 (2H, m), 7.28–7.23 (1H, m), 7.22–7.17 (2H, m), 3.73 (1H, s), 3.52 (1H, s), 2.30–2.25 (1H, m), 2.24–2.19 (1H, m), 1.52 (6H, s), 1.55–1.42 (1H, m), 1.42–1.33 (1H, m), 1.33–1.25 (1H, m), 0.86 (3H, d, *J* = 6.3 Hz), 0.84 (3h, d, *J* = 6.3 Hz); ^{13}C NMR (101 MHz, $CDCl_3$, *E*:*Z* isomers) δ 177.2, 177.2, 163.7, 162.3, 136.2,

136.1, 129.0, 128.7, 127.0, 126.7, 81.2, 81.0, 40.6, 34.8, 34.5, 34.4, 31.9, 28.1, 27.5, 26.0, 24.3, 24.2, 22.3, 22.2; HRMS (APCI) Found MH^+ 292.1894, $C_{17}H_{26}O_3N$ requires 292.1907.

(*E*)-2-(((1-Cyclohexyl-4-methylpentylidene)amino)oxy)-2-methylpropanoic Acid (6j)

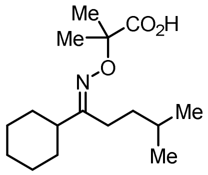

Following **GP4**, **S18** (200 mg, 1.12 mmol) gave **6j** (250 mg, 79%) as a solid. FT-IR ν_{max} (film)/cm^{-1} 2928, 2854, 1715, 1468, 1450, 1171, 975, 927, 755; ^{1}H NMR (400 MHz, $CDCl_3$) δ 2.34–2.25 (2H, m), 2.26–2.13 (1H, m), 1.81 (4H, m), 1.54 (1H, dd, *J* = 16.6, 10.0 Hz), 1.48 (6H, s), 1.42–1.13 (8H, m), 0.91 (6H, d, *J* = 6.6 Hz); ^{13}C NMR (101 MHz, $CDCl_3$) δ 175.9, 169.7, 81.1, 44.0, 35.0, 30.4, 28.6, 26.2, 26.1, 26.0, 24.5, 22.4.

2-(((1-Ethoxy-8-methyl-1-oxononan-5-ylidene)amino)oxy)-2-methylpropanoic Acid (6k)

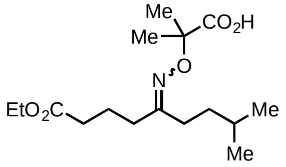

Following **GP4**, **S19** (513 mg, 2.4 mmol) gave **6k** (461 mg, 61%) as an oil. dr 1:1. FT-IR ν_{max} (film)/cm^{-1} 2955 (br.), 1732, 1714, 1170 (br.), 972; ^{1}H NMR (400 MHz, $CDCl_3$, *E*:*Z* isomers) δ 4.17–4.11 (2H, m), 2.41–2.22 (6H, m), 1.91–1.79 (2H, m), 1.58–1.48 (1H, m), 1.50 (6H, s), 1.43–1.32 (2H, m), 1.26 (1.5H, t, *J* = 7.1 Hz), 1.25 (1.5H, t, *J* = 7.1 Hz), 0.91 (3H, d, *J* = 6.5 Hz), 0.90 (3H, d, *J* = 6.6 Hz); ^{13}C NMR (126 MHz, $CDCl_3$, *E*:*Z* isomers) δ 176.2, 176.1, 173.2, 173.1, 164.4, 163.9, 81.2, 81.0, 60.6, 60.5, 34.8, 34.6, 33.8, 33.5, 33.3, 32.2, 28.2, 27.7, 27.6, 26.6, 24.3, 24.2, 22.3, 22.3, 21.2, 21.1, 14.2; HRMS (APCI) Found MH^+ 316.2103, $C_{16}H_{30}O_5N$ requires 316.2118.

2-(((10-Bromo-2-methyldecan-5-ylidene)amino)oxy)-2-methylpropanoic Acid (6l)

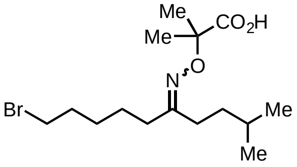

Following **GP4**, **S20** (300 mg, 1.2 mmol) gave **6l** (276 mg, 66%) as an oil. dr 1:1. FT-IR ν_{max} (film)/cm^{-1} 2955, 2867, 1716, 1467, 1362, 1170, 975, 755, 667; ^{1}H NMR (400 MHz, $CDCl_3$,

E:*Z* isomers) δ 3.42 (1H, t, *J* = 6.7 Hz), 3.41 (1H, t, *J* = 6.7 Hz), 2.41–2.31 (2H, m), 2.29–2.23 (2H, m), 1.92–1.85 (2H, m), 1.60–1.45 (5H, m), 1.49 (6H, s), 1.44–1.32 (2H, m), 0.92 (3H, d, *J* = 6.6 Hz), 0.91 (3H, d, *J* = 6.6 Hz); ^{13}C NMR (101 MHz, $CDCl_3$, *E*:*Z* isomers) δ 175.8, 175.7, 165.3, 165.2, 81.1, 53.4, 34.8, 34.6, 34.1, 33.6, 33.4, 32.4, 32.4, 32.2, 28.3, 28.2, 28.1, 27.7, 27.7, 26.7, 25.0, 24.8, 24.4, 24.3, 22.3, 22.3; HRMS (ASAP) Found MH^+ 350.1311, $C_{15}H_{29}O_3NBr$ requires 350.1325.

2-(((10-Azido-2-methyldecan-5-ylidene)amino)oxy)-2-methylpropanoic Acid (6m)

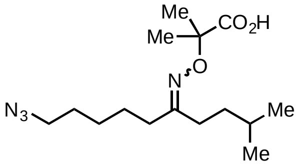

Following **GP4**, **S27** (93 mg, 0.44 mmol) gave **6m** (128 mg, 93%) as a yellow oil. dr 1:1. ^{1}H NMR (500 MHz, $CDCl_3$, *E*:*Z* isomers) δ 3.27 (2H, t, *J* = 6.9 Hz), 2.33–2.27 (2H, m), 2.12 (2H, q, *J* = 7.1 Hz), 1.68–1.54 (2H, m), 1.55–1.45 (3H, m), 1.40 (6H, s), 1.43–1.24 (4H, m), 0.90 (3H, d, *J* = 6.6 Hz), 0.87 (3H, d, *J* = 6.6 Hz); ^{13}C NMR (126 MHz, $CDCl_3$, *E*:*Z* isomers) δ 181.5, 159.9, 159.8, 81.7, 81.6, 51.3, 51.2, 47.6, 44.4, 35.5, 34.8, 33.9, 32.2, 28.6, 28.4, 28.2, 27.8, 27.7, 26.7, 26.4, 26.0, 25.3, 25.1, 25.0, 24.5, 22.4, 22.3; HRMS (ESI) Found MNa^+ 335.2023, $C_{15}H_{28}N_4NaO_3$ requires 335.2059.

2-(((10-Cyano-2-methyldecan-5-ylidene)amino)oxy)-2-methylpropanoic Acid (6n)

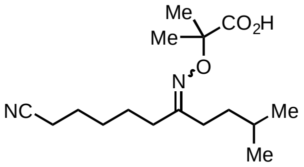

Following **GP4**, **S28** (90 mg, 0.46 mmol) gave **6n** (63 mg, 46%) as a yellow oil. dr 1:1. FT-IR ν_{max} (film)/cm^{-1} 3321, 2941, 2831, 1449, 1022, 668, 572; ^{1}H NMR (400 MHz, $CDCl_3$, *E*:*Z* isomers) δ 2.41–2.30 (4H, m), 2.27–2.19 (2H, m), 1.74–1.63 (2H, m), 1.61–1.43 (5H, m), 1.50 (6H, s), 1.44–1.30 (2H, m), 0.92 (3H, d, *J* = 6.5 Hz), 0.90 (3H, d, *J* = 6.5 Hz); ^{13}C NMR (101 MHz, $CDCl_3$, *E*:*Z* isomers) δ 176.7, 176.6, 164.4, 119.8, 119.7, 81.1, 81.0, 35.0, 34.7, 34.0, 32.5, 28.7, 28.3, 28.2, 27.8, 26.8, 25.2, 25.1, 25.0, 24.4, 24.3, 22.4, 22.3, 17.3, 17.2; HRMS (ESI) Found MNa^+ 297.2167, $C_{16}H_{28}N_2O_3$ requires 297.2178.

2-Methyl-2-(((2-methyl-10-phenoxydecan-5-ylidene)amino)oxy)propanoic Acid (6o)

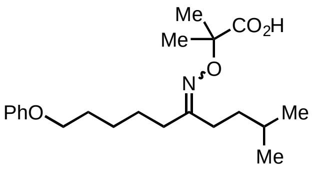

Following **GP4**, **S29** (200 mg, 0.76 mmol) gave **6o** (140 mg, 51%) as an yellow oil. dr 1.25:1. ^{1}H NMR (400 MHz, $CDCl_3$, *E*:*Z* isomers) δ 7.32–7.12 (2H, m), 6.91–6.83 (1H, m), 6.81 (2H, d, *J* = 8.6 Hz), 3.95–3.82 (2H, m), 2.38–2.24 (2H, m), 2.18 (2H, dt, *J* = 11.2, 7.6 Hz), 1.79–1.68 (2H, m), 1.56–1.37 (5H, m), 1.42 (3H, s), 1.38–1.24 (2H, m), 1.41 (3H, s), 0.84 (3H, d, *J* = 6.3 Hz), 0.83 (3H, d, *J* = 6.3 Hz); ^{13}C NMR (101 MHz, $CDCl_3$, *E*:*Z* isomers) δ 158.8, 129.2, 129.2, 129.2, 120.4, 120.3, 114.3, 114.27, 80.8, 80.7, 67.4, 67.3, 36.7, 34.7, 34.4, 34.0, 32.2, 28.8, 28.7, 28.4, 28.1, 28.0, 27.5, 26.4, 26.0, 25.5, 25.4, 25.3, 24.1, 22.5, 22.2, 22.1.

2-Methyl-2-(((2-methyl-10-(phenylthio)decan-5-ylidene)amino)oxy)propanoic Acid (6p)

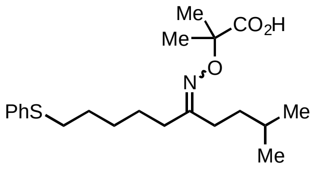

Following **GP4**, **S30** (140 mg, 0.5 mmol) gave **6p** (110 mg, 58%) as a pale yellow oil. dr 1.25:1. FT-IR ν_{max} (film)/cm^{-1} 1264, 734, 703, 545; ^{1}H NMR (400 MHz, $CDCl_3$, *E*:*Z* isomers) δ 7.28–7.15 (5H, m), 2.98–2.77 (2H, m), 2.31–2.21 (2H, m), 2.17–2.07 (2H, m), 1.72–1.58 (2H, m), 1.42 (3H, s), 1.40 (3H, s), 1.50–1.22 (7H, m), 0.83 (3H, d, *J* = 6.7 Hz), 0.82 (3H, d, *J* = 6.7 Hz); ^{13}C NMR (101 MHz, $CDCl_3$, *E*:*Z* isomers) δ 177.5, 177.4, 164.3, 164.2, 136.7, 129.1, 129.0, 127.5, 127.1, 125.8, 125.7, 80.8, 80.7, 34.9, 34.6, 34.1, 33.4, 32.3, 28.8, 28.7c, 28.3, 28.2, 281.1, 27.6, 26.5, 25.5, 25.2, 24.3, 22.4, 22.2; HRMS (APCI) Found M-H$^+$ 378.2097, $C_{21}H_{32}O_3NS$ requires 378.2110.

(*E*)-2-(((4,8-Dimethyl-1-phenylnonylidene)amino)oxy)-2-methylpropanoic Acid (6q)

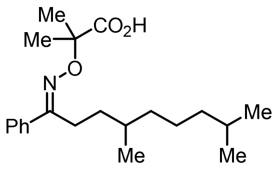

Following **GP4**, **S21** (400 mg, 1.63 mmol) gave **6q** (430 mg, 76%) as an oil. FT-IR νmax (film)/cm^{-1} 3345, 2969, 1466, 1378, 1345, 1304, 1160, 1127, 1107, 950; ^{1}H NMR (400 MHz,

$CDCl_3$) δ 7.56–7.52 (2H, m), 7.37–7.29 (3H, m), 2.78–2.67 (2H, m), 1.53 (6H, s), 1.51–1.37 (5H, m), 1.33–1.15 (5H, m), 0.87 (3H d, *J* = 6.5 Hz), 0.79 (6H, d, *J* = 6.6 Hz); ^{13}C NMR (126 MHz, $CDCl_3$) δ 165.9, 161.2, 141.0, 135.1, 132.9, 129.6, 128.6, 126.4, 81.8, 39.2, 36.7, 33.3, 33.0, 28.0, 24.7, 24.5, 24.5, 24.4, 22.7, 22.6, 19.5.

2-(((5,9-Dimethyl-1-phenyldecan-2-ylidene)amino)oxy)-2-methylpropanoic Acid (6r)

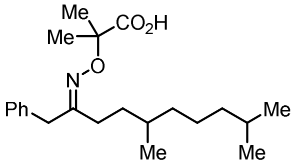

Following **GP4**, **S22** (200 mg, 0.76 mmol) gave **6r** (262 mg, 95%) as an oil. dr:1:1. FT-IR ν_{max} (film)/cm^{-1} 2953, 2925, 2868, 1715, 1467, 1453, 1378, 1363, 1169, 971, 735, 699; ^{1}H NMR (400 MHz, $CDCl_3$, *E*:*Z* isomers) δ 7.35–7.24 (3H, m), 7.22–7.16 (2H, m), 3.76 (0.5H, d, *J* = 14.1 Hz), 3.70 (0.5H, d, *J* = 14.1 Hz), 3.52 (1H, s), 2.37–2.08 (2H, m), 1.51 (6H, s), 1.44–1.03 (8H, m), 0.89–0.82 (9H, m); ^{13}C NMR (126 MHz, $CDCl_3$, *E*:*Z* isomers) δ 176.1, 176.0, 164.6, 163.2, 136.1, 135.9, 129.2, 129.1, 128.9, 128.9, 127.2, 126.9, 81.5, 81.3, 40.8, 39.4, 39.4, 37.0, 36.8, 34.7, 33.2, 33.0, 32.6, 32.5, 31.8, 28.1, 28.1, 27.7, 25.9, 24.8, 24.8, 24.5, 24.4, 22.8, 22.7, 22.7, 19.5, 19.4; HRMS (APCI) Found MH^+ 360.2544, $C_{22}H_{34}NO_3$ requires 360.2539.

2-(((3-Hydroxy-5-methyl-1-phenylhexan-2-ylidene)amino)oxy)-2-methylpropanoic Acid (6s)

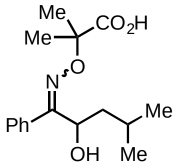

Following **GP4**, **S23** (103 mg, 0.5 mmol) gave **6s** (137 mg, 89%) as an oil. dr 4:1. FT-IR ν_{max} (film)/cm^{-1} 1710, 1418, 1359, 1220, 1091, 581; ^{1}H NMR (400 MHz, $CDCl_3$, *E*:*Z* isomers) δ 7.22–7.06 (5H, m), 4.76–4.69 (0.2H, m), 4.16 (0.8H, br t, *J* = 6.2 Hz), 3.82 (0.8H, d, *J* = 14.3 Hz), 3.52 (0.8H, d, *J* = 14.3 Hz), 3.51 (0.4H, br s), 1.70–1.54 (1H, m), 1.44 (6H, br s), 1.36–1.30 (1.6H, m), 1.28–1.12 (0.4H), 0.77 (2.4H, d, *J* = 6.7 Hz), 0.71 (2.4H, d, *J* = 6.2 Hz), 0.82–0.67 (1.2H, m); ^{13}C NMR (101 MHz, $CDCl_3$, *E*:*Z* isomers) δ 179.0^{M}, 178.9^{m}, 164.0^{m}, 161.1^{M}, 136.8^{m}, 136.4^{M}, 131.0, 129.2, 128.7, 128.6, 128.3, 127.1, 126.8, 126.6, 81.5^{M}, 81.3^{m}, 70.8^{M}, 67.8^{m}, 45.8, 44.0^{M}, 43.4^{m}, 37.1^{m}, 31.8^{M}, 24.6^{m}, 24.5^{M}, 24.1^{M}, 24.0^{M}, 23.6^{M}, 23.2^{m}, 22.0^{M},21.5^{m}; HRMS (ESI) Found MH^+ 307.1784, $C_{17}H_{25}NO_4$ requires 307.1784.

2-(((4-(1-(*tert*-Butoxycarbonyl)piperidin-4-yl)-1-phenylbutan-2-ylidene)amino)oxy)-2-methylpropanoic Acid (6t)

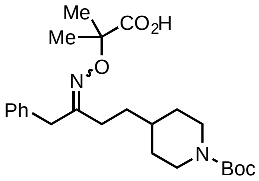

Following **GP4**, **S24** (200 mg, 0.6 mmol) gave **6t** (220 mg, 85%) as an oil. dr 1:1. ^{1}H NMR (400 MHz, $CDCl_3$, *E*:*Z* isomers) δ 7.36–7.15 (5H, m), 4.04 (2H, br s), 3.73 (1H, s), 3.50 (1H, s), 2.62 (2H, br s, *J* = 11.9 Hz), 2.30 (1H, t, *J* = 7.5 Hz), 2.21 (1H, t, *J* = 7.5 Hz), 1.66–1.59 (2H, m), 1.55 (3H, s), 1.55 (3H, s), 1.46 (4.5H, s), 1.45 (4.5H, s), 1.49–1.26 (3H, m), 1.11–0.91 (2H, m); ^{13}C NMR (101 MHz, $CDCl_3$, *E*:*Z* isomers) δ 177.7, 177.6, 162.4, 161.0, 155.1, 136.4, 130.7, 129.1, 129.1, 128.8, 127.1,126.8, 81.2, 81.1, 79.5, 40.7, 35.9, 35.3, 34.5, 32.4, 32.3, 31.9, 31.0, 28.6, 25.3, 24.3, 24.3.

2-Methyl-2-(((5-methyl-1-phenyldecan-2-ylidene)amino)oxy)propanoic Acid (6u)

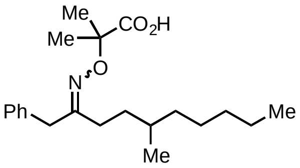

Following **GP4**, **S25** (144 mg, 0.62 mmol) gave **6u** (179 mg, 54%) as an pale yellow oil. dr 1.8:1. FT-IR ν_{max} (film)/cm^{-1} 3344, 2359, 1649, 1264, 1016, 733, 703; ^{1}H NMR (400 MHz , $CDCl_3$, *E:Z* isomers) δ 7.34–7.09 (5H, m), 3.69 (0.4H, d, *J* = 13.2 Hz), 3.63 (0.4H, d, *J* = 14.0 Hz), 3.45 (1.2H, s), 2.19 (1.2H, t, *J* = 7.7 Hz), 2.12–1.53 (0.8H, m), 2.02–1.53 (2H, m), 1.46 (6H, s), 1.41–0.96 (9H, m), 0.88–0.69 (6H, m); ^{13}C NMR (101 MHz, $CDCl_3$, *E:Z* isomers) δ 176.6, 164.2, 162.8, 136.0, 135.9, 129.0, 129.0, 128.7, 127.0, 126.8, 81.3, 81.1, 40.7, 36.4, 36.1, 34.5, 33.0, 32.8, 32.3, 32.4, 31.7, 29.2, 29.1, 25.7, 24.3, 23.0, 19.4, 19.3, 14.1; HRMS (ESI) Found MNa$^+$ 370.2362, $C_{21}H_{33}NaO_3$ requires 370.2358.

2-(((4-Cyclopentyl-1-phenylbutan-2-ylidene)amino)oxy)-2-methylpropanoic Acid (6v)

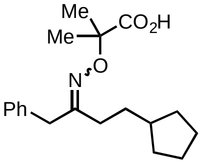

Following **GP4**, **S26** (86 mg, 0.4 mmol) gave **6v** (41 mg, 37%) as a colourless oil. dr 1.3:1. FT-IR ν_{max} (film)/cm^{-1} 1708, 1359, 12221, 582; ^{1}H NMR (500 MHz, $CDCl_3$, *E*:*Z* isomers) δ

7.37–7.08 (5H, m), 3.73 (0.9H, s), 3.52 (1.1H, s), 2.31–2.26 (1.1H, m), 2.24–2.18 (0.9H, m), 1.79–1.64 (4H, m), 1.61–1.38 (7H, m), 1.53 (6H, s); ^{13}C NMR (126 MHz, $CDCl_3$, *E*:*Z* isomers) δ 176.8^{M}, 176.7^{m}, 163.8^{M}, 162.5^{m}, 136.1^{m}, 136.0^{M}, 129.0 ^{M}, 128.7^{m}, 127.0^{M},126.7^{m}, 81.3^{m}, 81.1^{M}, 40.6, 40.1, 39.6, 34.5, 33.2, 32.4, 32.2, 31.9, 29.7, 27.4, 25.1, 24.3^{m}, 24.3^{M}; HRMS (ESI) Found MNa^+ 340.1891, $C_{19}H_{27}NaO_3$ requires 340.1889.

2-((((5-((5*R*,8*R*,9*S*,10*S*,13*R*,14*S*,17*R*)-3-Hydroxy-10,13-dimethylhexadecahydro-1H-cyclopenta[a]phenanthren-17-yl)hexan-2-ylidene)amino)oxy)-2-methylpropanoic Acid (6w)

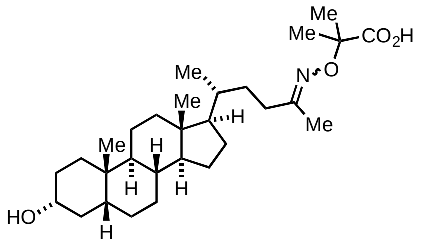

Following **GP4**, **S31** (150 mg, 0.4 mmol) gave **6w** (120 mg, 63%) as a solid. dr: 4:1. FT-IR ν_{max} (film)/cm^{-1} 3314, 2942, 2831, 1448, 1418, 606, 546; ^{1}H NMR (400 MHz, $CDCl_3$, *E*:*Z* isomers) δ 3.73–3.57 (0.8H, m), 3.52–3.44 (0.2H, m), 2.08 (0.7H, s), 2.40-2.08 (2H, m), 2.00–1.92 (1H, m), 1.89 (2.3H, s), 1.86–1.45 (8H, m), 1.50 (6H, s), 1.43–1.32 (7H, m), 1.28–0.96 (10H, m), 0.91 (3H, s), 0.63 (0.7H, s), 0.62 (2.3H, s); ^{13}C NMR (101 MHz, $CDCl_3$, *E*:*Z* isomers) δ 177.0^{M}, 176.3^{m}, 162.6 ^{m}, 162.0 ^{M}, 81.0 ^{M}, 80.9 ^{m}, 72.0, 56.6, 56.1^{M}, 55.8^{m}, 43.0, 42.2, 40.5, 40.3, 36.5, 35.9, 35.8, 35.4, 35.3, 34.7, 32.9, 32.2, 31.6, 30.6, 28.4^{M}, 28.3^{m},27.3, 26.5^{M}, 26.5^{m}, 24.5, 24.3, 23.5, 20.9 , 20.4, 18.6^{m}, 18.4^{M}, 14.7, 12.1; HRMS (APCI) Found MH^+ 474.3589, $C_{29}H_{49}NO_4$ requires 474.3583.

3 Reaction Optimizations

All reactions were optimised using Fuzumi's acridinium **2** as the photocatalyst.

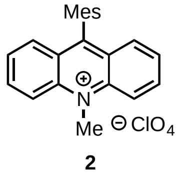

3.1 Cascade Ring-Opening-Fluorination

General Procedure for the Reaction Optimization – GP5

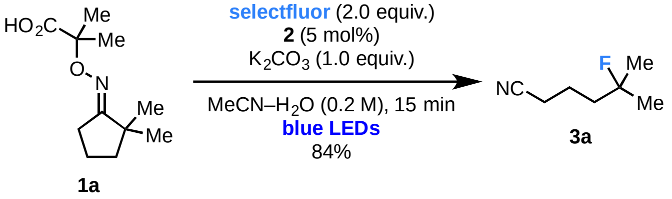

A dry tube equipped with a stirring bar was charged with **1a** (21 mg, 0.1 mmol, 1.0 equiv.), **2** (2 mg, 0.005 mmol, 5 mol%), the base (0.1 mmol, 1.0 equiv.), and the F-source (0.2 mmol, 2.0 equiv.). The reaction vessel was sealed, evacuated and back-filled with nitrogen three times, then sealed with parafilm. The solvent (0.5 mL) was added, the blue LEDs were switched on and the reaction was stirred under irradiation for the given amount time. H_2O (1 mL), EtOAc (1 mL) and 1,3-Dinitrobenzene (4 mg, 0.025 mmol, 0.5 equiv.) were added. The layers were separated and the aqueous layer was extracted with EtOAc (2 x 2 mL). The combined organic layers were dried ($MgSO_4$), filtered and evaporated. $CDCl_3$ (0.4 mL) was added and the mixture was analysed by ^{1}H NMR spectroscopy to determine the NMR yield.

The optimum reaction conditions identified by this optimisation study were:

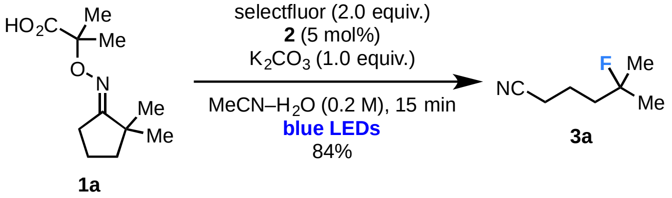

The following Table reports all experiments performed.

Entry	F-source	Base	Solvent	Time	Yield (%)
**1**	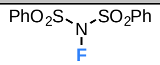	Cs_2CO_3	MeCN	1h	–
**2**	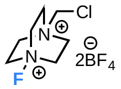	Cs_2CO_3	MeCN	1h	13%
**3**	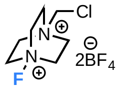	Cs_2CO_3	MeCN–H_2O (1:1)	1h	67
**4**	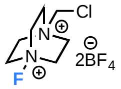	K_2CO_3	MeCN–H_2O (1:1)	1h	70%
**5**	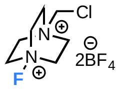	Et_3N	MeCN–H_2O (1:1)	1h	8%
**6**	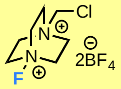	K_2CO_3	MeCN–H_2O (1:1)	15 min	84%
			Control Experiments		
**7**[a]	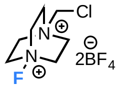	K_2CO_3	MeCN–H_2O (1:1)	1h	–
**8**[b]	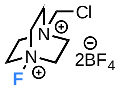	K_2CO_3	MeCN–H_2O (1:1)	1h	–
**9**	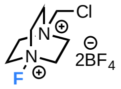	–	MeCN–H_2O (1:1)	1h	8%

[a] the reaction was run without light.

[b] the reaction was run without **2**.

3.2 Cascade Ring-Opening-Chlorination

General Procedure for the Reaction Optimization – GP6

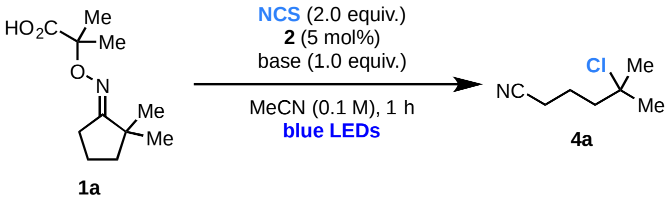

A dry tube equipped with a stirring bar was charged with **1a** (21 mg, 0.1 mmol, 1.0 equiv.), **2** (2 mg, 0.005 mmol, 5 mol%), the base (0.1 mmol, 1.0 equiv.), and NCS (27 mg, 0.2 mmol, 2.0 equiv.). The reaction vessel was sealed, evacuated and back-filled with nitrogen three times, then sealed with parafilm. MeCN (1.0 mL) was added, the blue LEDs were switched on and the reaction was stirred under irradiation for 1 h. H_2O (1 mL), EtOAc (1 mL) and 1,3-Dinitrobenzene (4 mg, 0.025 mmol, 0.5 equiv.) were added. The layers were separated and the aqueous layer was extracted with EtOAc (2 x 2 mL). The combined organic layers were dried ($MgSO_4$), filtered and evaporated. $CDCl_3$ (0.4 mL) was added and the mixture was analysed by ^1H NMR spectroscopy to determine the NMR yield.

The optimum reaction conditions identified by this optimisation study were:

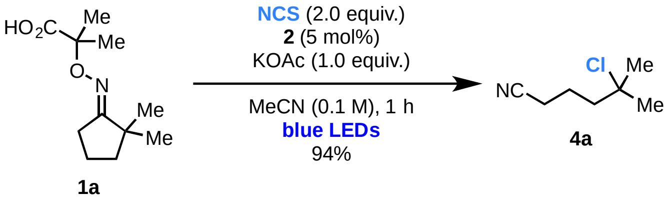

The following Table reports all experiments performed.

Entry	Base	Solvent	Time	Yield (%)
**1**	Cs_2CO_3	MeCN	1 h	65
**2**	K_2CO_3	MeCN	1 h	84
**3**	KOAc	MeCN	1 h	94
		Control Experiments		
**4**	KOAc	MeCN	1 h	–
**5**	KOAc	MeCN	1 h	–
**6**	–	MeCN	1 h	–

[a] the reaction was run without light.

[b] the reaction was run without **2**.

3.3 Cascade Ring Opening-Azidation

General Procedure for the Reaction Optimization – GP7

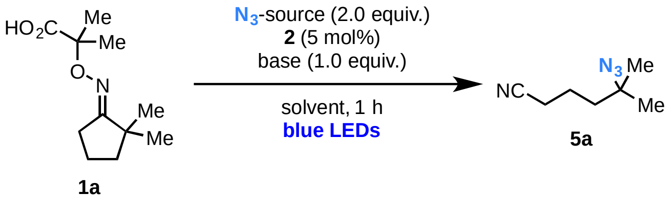

A dry tube equipped with a stirring bar was charged with **1a** (21 mg, 0.1 mmol, 1.0 equiv.), **2** (2 mg, 0.005 mmol, 5 mol%), the base (0.1 mmol, 1.0 equiv.), and the azide source (0.2 mmol, 2.0 equiv.). The reaction vessel was sealed, evacuated and back-filled with nitrogen three times, then sealed with parafilm. The solvent (0.2 mL, 0.1 M) was added, the blue LEDs were switched on and the reaction was stirred under irradiation. H_2O (1 mL), EtOAc (1 mL) and 1,3-Dinitrobenzene (4 mg, 0.025 mmol, 0.5 equiv.) were added. The layers were separated and the aqueous layer was extracted with EtOAc (2 x 2 mL). The combined organic layers were dried ($MgSO_4$), filtered and evaporated. $CDCl_3$ (0.4 mL) was added and the mixture was analysed by ^{1}H NMR spectroscopy to determine the NMR yield.

The optimum reaction conditions identified by this optimisation study were:

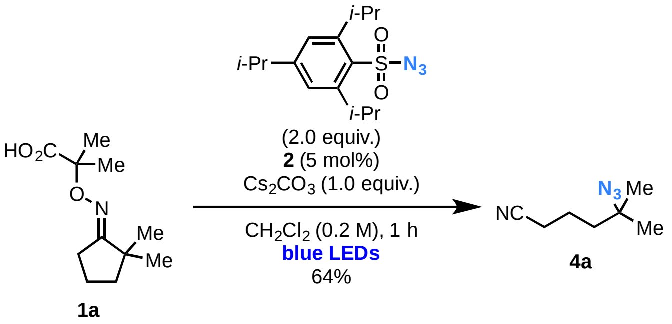

The following Table reports all experiments performed.

Entry	N_3-source	Base	Solvent	Yield (%)
**1**	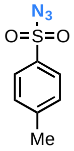	Cs_2CO_3	toluene	–
**2**	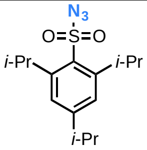	Cs_2CO_3	toluene	45%
**3**	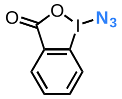	Cs_2CO_3	toluene	traces
**4**	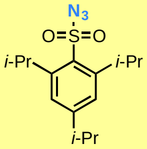	Cs_2CO_3	CH_2Cl_2	64%
Control Experiments				
**5**	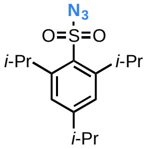<br>(4.0)	Cs_2CO_3	CH_2Cl_2	–
**6**	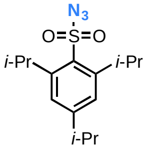<br>(4.0)	Cs_2CO_3	CH_2Cl_2	–
**7**	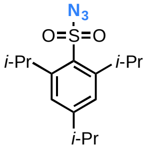<br>(4.0)	–	CH_2Cl_2	–

[a] the reaction was run without light.

[b] the reaction was run without **2**.

3.4 Cascade 1,5-H Abstraction-Chlorination

General Procedure for the Reaction Optimization – GP8

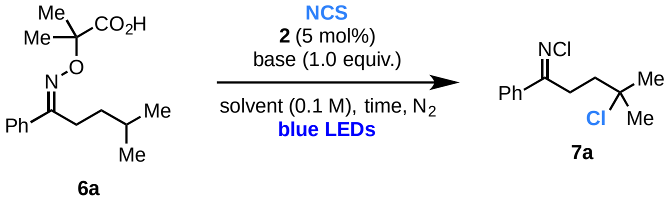

A dry tube equipped with a stirring bar was charged with **6a** (28 mg, 0.1 mmol, 1.0 equiv.), **2** (2 mg, 0.005 mmol, 5 mol%), the base (0.1 mmol, 1.0 equiv.), and NCS (54 mg, 0.4 mmol, 4 equiv.). The reaction vessel was sealed, evacuated and back-filled with nitrogen three times. The solvent (1 mL) was added, the blue LEDs were switched on and the reaction was stirred under irradiation, under a constant flow of nitrogen for the given amount of time. H_2O (1 mL), EtOAc (1 mL) and 1,3-Dinitrobenzene (4 mg, 0.025 mmol, 0.5 equiv.) were added. The layers were separated and the aqueous layer was extracted with EtOAc (2 x 2 mL). The combined organic layers were dried ($MgSO_4$), filtered and evaporated. $CDCl_3$ (0.4 mL) was added and the mixture was analysed by ^{1}H NMR spectroscopy to determine the NMR yield.

The optimum reaction conditions identified by this optimisation study were:

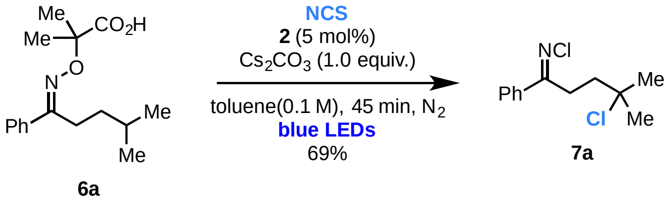

The following Table reports all experiments performed.

Entry	Base	Solvent	Time	Yield (%)
**1**	K_2CO_3	toluene	16 h	41
**2**	Cs_2CO_3	CH_2Cl_2	16 h	41
**3**	Cs_2CO_3	toluene	16 h	57
**4**	Cs_2CO_3	Toluene	45 min	69
Control Experiments				
**5**	Cs_2CO_3	toluene	1 h	–
**6**	Cs_2CO_3	toluene	1 h	–
**7**	–	toluene	1 h	–

[a] the reaction was run without light.

[b] the reaction was run without **2**.

3.5 Cascade 1,5-H Abstraction-Fluorination

General Procedure for the Reaction Optimization – GP8

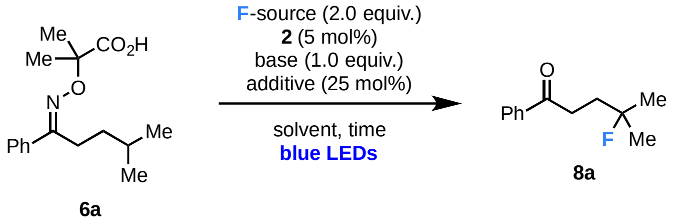

A dry tube equipped with a stirring bar was charged with **6a** (14 mg, 0.05 mmol, 1.0 equiv.), **2** (2 mg, 0.005 mmol, 5 mol%), the base (0.05 mmol, 1.0 equiv.), the F-source (0.1 mmol, 2.0 equiv.) and the additive (0.0125 mmol, 25 mol%). The reaction vessel was sealed, evacuated and back-filled with nitrogen three times. The solvent was added, the blue LEDs were switched on and the reaction was stirred under irradiation, under a constant flow of nitrogen. H_2O (1 mL), EtOAc (1 mL) and 1,3-Dinitrobenzene (4 mg, 0.025 mmol, 0.5 equiv.) were added. The layers were separated and the aqueous layer was extracted with EtOAc (2 x 2 mL). The combined organic layers were dried ($MgSO_4$), filtered and evaporated. $CDCl_3$ (0.4 mL) was added and the mixture was analysed by ^1H NMR spectroscopy to determine the NMR yield.

The optimum reaction conditions identified by this optimisation study were:

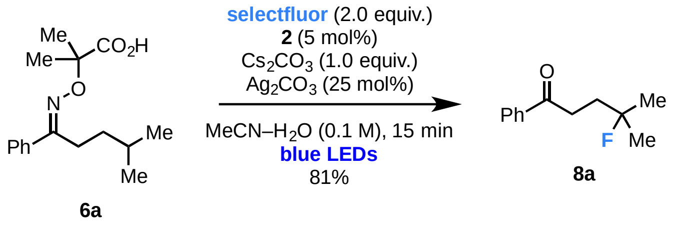

The following Table reports all experiments performed.

Entry	F-Source	Base	Additive (equiv.)	Solvent (M)	Time	Yield (%)
**1**	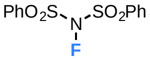	Cs_2CO_3	–	CH_2Cl_2 (0.1 M)	16 h	–
**2**	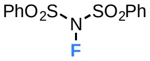	Cs_2CO_3	–	CH_2Cl_2 (0.1 M)	1 h	–
**3**	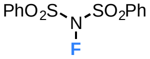	K_2CO_3	–	CH_2Cl_2 (0.1 M)	1 h	–
**4**	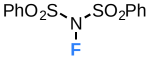	KH_2PO_4	–	CH_2Cl_2 (0.1 M)	1 h	–
**5**	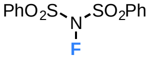	CsF	–	CH_2Cl_2 (0.1 M)	1 h	–
**6**	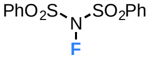	Cs_2CO_3	–	HFIP (0.1M)	1 h	–
**7**	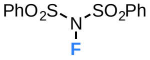	Cs_2CO_3	–	toluene (0.1M)	1 h	–
**8**	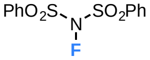	Cs_2CO_3	–	MeCN (0.1M)	1 h	–
**9**	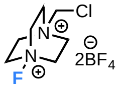	Cs_2CO_3	–	CH_2Cl_2 (0.1 M)	16 h	–
**10**	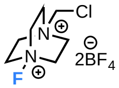	Cs_2CO_3	–	MeCN (0.1M)	1 h	–
**11**	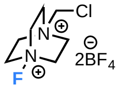	Cs_2CO_3	–	MeCN–H_2O 1:1 (0.1M)	1 h	17
**12**	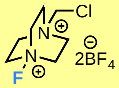	Cs_2CO_3	Ag_2CO_3 (0.25)	MeCN–H_2O 1:1 (0.1M)	15 min	81

Entry	F-Source	Base	Additive (equiv.)	Solvent (M)	Time	Yield (%)
**13**	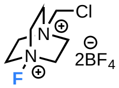	Cs_2CO_3	Ag_2CO_3 (0.25)	MeCN–H_2O 1:1 (0.05M)	15 min	65
**14**	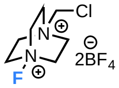	Cs_2CO_3	Ag_2NO_3 (0.25)	MeCN–H_2O 1:1 (0.05M)	15 min	60
**15**	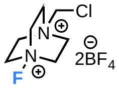	Cs_2CO_3	$AgSbF_6$ (0.25)	MeCN–H_2O 1:1 (0.05M)	15 min	20
**16**	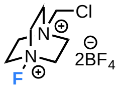	Cs_2CO_3	$Ag(phen)_2OTf$ (0.25)	MeCN–H_2O 1:1 (0.05M)	15 min	39
**17**	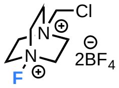	Cs_2CO_3	$AgBF_4$ (0.25)	MeCN–H_2O 1:1 (0.05M)	15 min	32
**18**	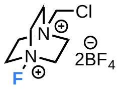	Cs_2CO_3	AgO (0.25)	MeCN–H_2O 1:1 (0.05M)	15 min	19
**19**	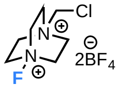	NaH_2PO_4	Ag_2CO_3 (0.25)	MeCN–H_2O 1:1 (0.1M)	15 min	40
**20**	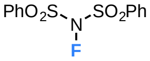	Cs_2CO_3	Ag_2CO_3 (0.25)	MeCN–H_2O 1:1 (0.1M)	15 min	11
Control Experiments						
**21**[a]	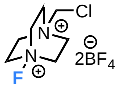	Cs_2CO_3	Ag_2CO_3 (0.25)	MeCN–H_2O 1:1 (0.1M)	15 min	34

Entry	F-Source	Base	Additive (equiv.)	Solvent (M)	Time	Yield (%)
**22**[b]	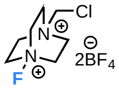	Cs_2CO_3	Ag_2CO_3 (0.25)	MeCN-H_2O 1:1 (0.1M)	15 min	–
**23**	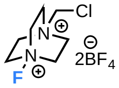	–	Ag_2CO_3 (0.25)	MeCN-H_2O 1:1 (0.1M)	15 min	2

[a] the reaction was run without **2**.

[b] the reaction was run in the dark.

4 Picture of Reaction Set-Up

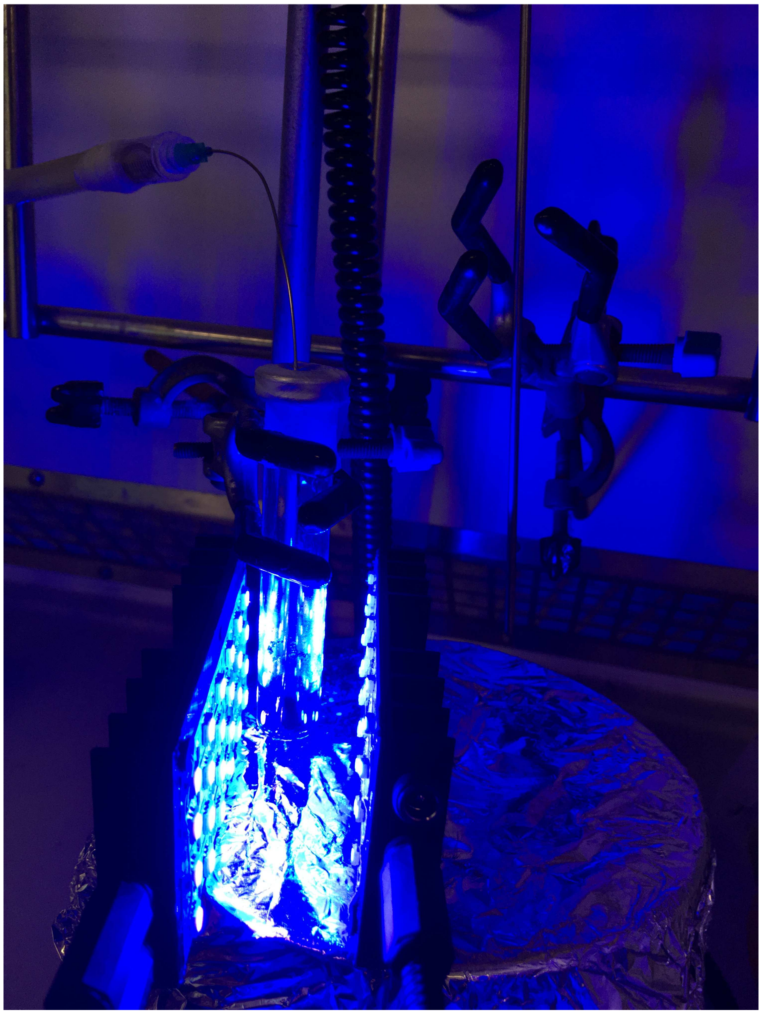

5 Reaction Products

5.1 Cascade Ring-Opening-Fluorination

General Procedure for the Reaction Optimization – GP9

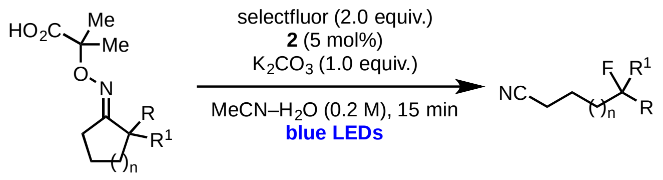

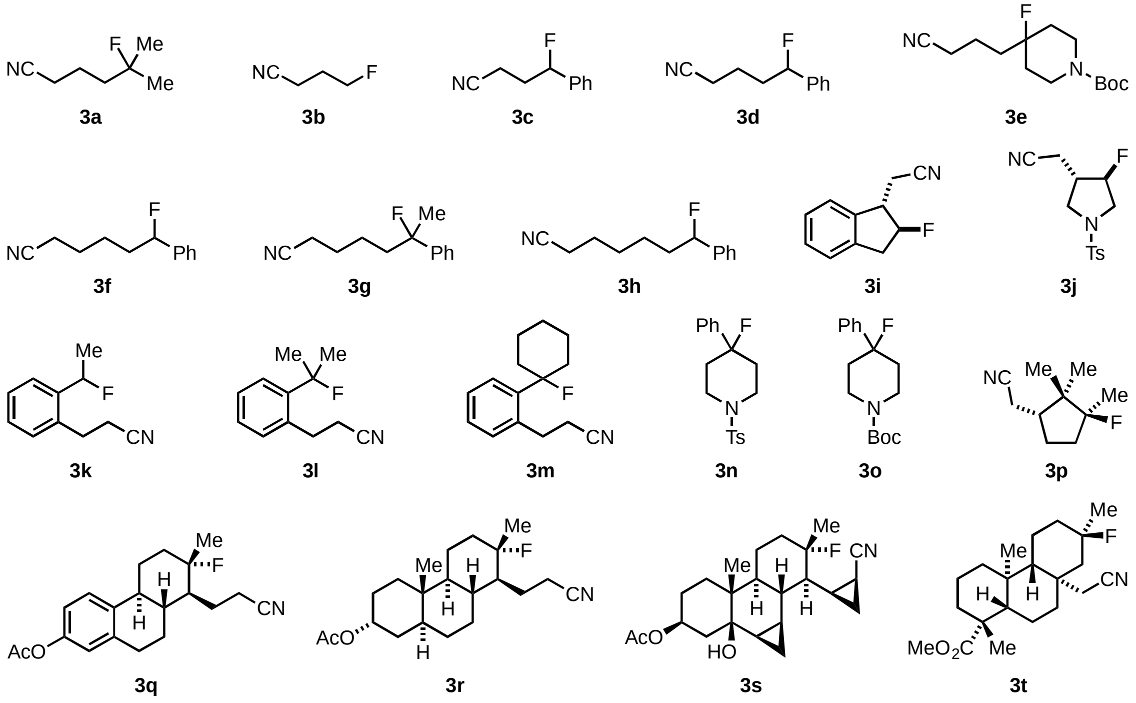

A dry tube equipped with a stirring bar was charged with the oxime (1.0 equiv.), **2** (5 mol%), K_2CO_3 (0.1 mmol, 1.0 equiv.) and selectfluor (0.2 mmol, 2.0 equiv.). The tube was sealed, evacuated and back-filled with nitrogen three times. MeCN–H_2O (1:1, 0.2 M) was added, the blue LEDs were switched on and the reaction was stirred under irradiation for 15 min. The mixture was dilute with H_2O (1 mL) and EtOAc (1 mL). The layers were separated and the aqueous layer was extracted with EtOAc (3 x 5 mL). The combined organic layers were dried ($MgSO_4$), filtered and evaporated. The residue was purified by column chromatography on silica gel.

5-Fluoro-5-methylhexanenitrile (3a)

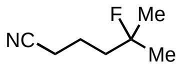

Following **GP9**, **1a** (19 mg, 0.1 mmol) gave **3a** (11 mg, 84%) as an oil. ^{1}H NMR (400 MHz, $CDCl_3$) δ 2.40 (2H, t, *J* = 6.4 Hz), 1.86–1.69 (4H, m), 1.37 (6H, d, *J* = 21.3 Hz); ^{19}F NMR (376 MHz, $CDCl_3$) δ –140.0. Data in accordance with the literature.[14]

4-Fluoro-4-phenylbutanenitrile (3b)

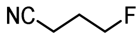

Following **GP9**, **1b** (17 mg, 0.1 mmol) gave **3b** (76%) as an oil.[d] ^{1}H NMR (500 MHz, $CDCl_3$) δ 4.52 (2H, dt, *J* = 46.9, 5.5 Hz), 2.49 (2H, t, *J* = 7.1 Hz), 2.07–1.98 (2H, m); ^{13}C NMR (126 MHz, $CDCl_3$) δ 118.6, 81.2 (d, *J* = 167.3 Hz), 47.4, 26.2 (H, d, *J* = 20.5 Hz), 13.2 (H, d, *J* = 5.3 Hz); ^{19}F NMR ($CDCl_3$, 376 MHz) δ –222.8.

4-Fluoro-4-phenylbutanenitrile (3c)

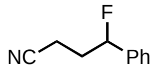

Following **GP9**, **1c** (12 mg, 0.05 mmol) gave **3c** (6 mg, 68%). ^{1}H NMR (500 MHz, $CDCl_3$) δ 7.47–7.22 (5H, m), 5.51 (1H, ddd, *J* = 47.6, 8.6, 3.9 Hz), 2.55–2.05 (4H, m); ^{13}C NMR (126 MHz, $CDCl_3$) δ 138.3, 129.1, 128.9, 125.4, 119.0, 92.2 (d, *J* = 173.5 Hz), 33.6, 13.5; ^{19}F NMR (471 MHz, $CDCl_3$) δ –179.6. Data in accordance with the literature.[15]

5-Fluoro-5-phenylpentanenitrile (3d)

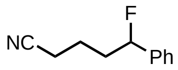

Following **GP9**, **1d** (13 mg, 0.05 mmol) gave **3d** (6 mg, 68%) as an oil. ^{1}H NMR (500 MHz, $CDCl_3$) δ 7.43 – 7.30 (5H, m), 5.49 (1H, ddd, *J* = 47.8, 8.1, 4.2 Hz), 2.49–2.34 (2H, m), 2.18–1.95 (2H, m), 1.94–1.72 (2H, m); δ ^{13}C NMR (100 MHz, CDCl3) δ 16.9, 21.2 (d, *J* = 3.8 Hz), 35.8 (d, *J* = 23.8 Hz), 93.5 (d, *J* = 172.2 Hz), 119.2, 125.2 (d, *J* = 6.7 Hz), 128.5 (d, *J* =1.9 Hz), 128.6, 139.4 (d, *J* = 20.0 Hz); ^{19}F NMR (471 MHz, $CDCl_3$) δ –176.9. Data in accordance with the literature.[16]

---

[d] As **3b** is volatile, the reaction was performed in CD_3CN–D_2O and after 15 min under blue LEDs irradiation, a solution of 1,3-$(MeO)_3$-C_6H_3 in $CDCl_3$ was added and the yield calculated by crude ^{1}H NMR analysis.

tert-Butyl 4-(3-Cyanopropyl)-4-fluoropiperidine-1-carboxylate (3e)

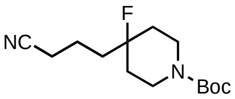

Following **GP9**, **1e** (35 mg, 0.1 mmol) gave **3e** (10 mg, 35%) as an oil. FT-IR ν_{max} (film)/cm^{-1} 3013, 2947, 1689, 1427, 1352, 1244, 997, 908, 732; ^{1}H NMR (400 MHz, $CDCl_3$) δ 3.94 (2H, br s), 3.06 (2H, br s), 2.40 (2H, t, *J* = 6.7 Hz), 1.87–1.75 (5H, m), 1.75–1.68 (1H, m), 1.54–1.42 (2H, m), 1.46 (9H, s); ^{13}C NMR (126 MHz, $CDCl_3$) δ 154.7, 119.3, 93.4 (d, *J* = 172.2 Hz), 79.7, 39.0 (d, *J* = 22.3 Hz), 34.5 (d, *J* = 13.9 Hz), 29.7, 28.4, 19.0, 17.4; ^{19}F NMR (376 MHz, $CDCl_3$) δ –164.2; HRMS (ASAP) Found MNa^{+} 293.1625, $C_{14}H_{23}O_2N_2FNa$ requires 293.1636.

6-Fluoro-6-phenylhexanenitrile (3f)

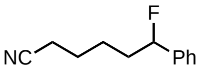

Following **GP9**, **1f** (28 mg, 0.1 mmol) gave **3f** (12 mg, 61%) as an oil. FT-IR ν_{max} (film)/cm^{-1} 2253, 1375, 1038, 917, 735; ^{1}H NMR (400 MHz, $CDCl_3$) δ 7.42–7.34 (3H, m), 7.32 (2H, d, *J* = 7.8 Hz), 5.44 (1H, ddd, *J* = 47.7, 8.1, 4.6 Hz), 2.36 (2H, t, *J* = 7.0 Hz), 2.08–1.78 (2H, m), 1.78–1.53 (4H, m); ^{13}C NMR (101 MHz, $CDCl_3$) δ 139.9 (d, *J* = 19.9 Hz), 128.6, 128.4 (d, *J* = 1.8 Hz), 125.4 (d, *J* = 6.9 Hz), 119.5, 94.1 (d, *J* = 171.4 Hz), 36.4 (d, *J* = 23.9 Hz), 25.3, 24.4 (d, *J* = 4.1 Hz), 17.1; ^{19}F NMR (376 MHz, $CDCl_3$) δ –175.4; HRMS (ESI) Found MNa^{+} 214.0998, $C_{12}H_{14}NFNa$ requires 214.1002.

6-Fluoro-6-phenylheptanenitrile (3g)

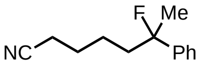

Following **GP9**, **1g** (29 mg, 0.1 mmol) gave **3g** (13 mg, 63%) as an oil. FT-IR ν_{max} (film)/cm^{-1} 3019, 1352, 1214, 1028, 864, 550; ^{1}H NMR (400 MHz, $CDCl_3$) δ 7.40–7.34 (2H, m), 7.33–7.27 (3H, m), 2.28 (2H, t, *J* = 7.1 Hz), 2.01–1.96 (1H, m), 1.96–1.89 (1H, m), 1.66 (3H, d, *J* = 21.5 Hz), 1.60–1.45 (2H, m), 1.38– 1.24 (2H, m); ^{13}C NMR (101 MHz, $CDCl_3$) δ 144.3 (d, *J* = 22.0 Hz), 128.3, 127.3, 123.9 (d, *J* = 9.9 Hz), 119.6, 97.4 (d, *J* = 173.1 Hz) 41.2 (d, *J* = 24.1 Hz), 27.9 (d, *J* = 25.3 Hz), 25.5, 23.0 (d, *J* = 3.7 Hz), 17.08; ^{19}F NMR (376 MHz, $CDCl_3$) δ –149.6; HRMS (ESI) Found MNa^{+} 228.1156, $C_{13}H_{16}NFNa$ requires 228.1159.

7-Fluoro-7-phenylheptanenitrile (3h)

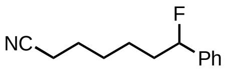

Following **GP9**, **1h** (29 mg, 0.1 mmol) gave **3h** (15 mg, 72%) as an oil. FT-IR ν_{max} (film)/cm^{-1} 2950, 2400, 1450, 904, 725, 649; ^{1}H NMR (400 MHz, CD_3Cl) δ 7.41–7.36 (2H, m), 7.35–7.30 (3H, m), 5.43 (1H, ddd, *J* = 47.8, 8.1, 4.7 Hz), 2.34 (2H, t, *J* = 7.0 Hz), 2.06–1.75 (3H, m), 1.71–1.63 (2H, m), 1.55–1.38 (5H, m); ^{13}C NMR (126 MHz, $CDCl_3$) δ 140.2 (d, *J* = 19.7 Hz), 128.5, 128.3 (d, *J* = 2.1 Hz), 125.4 (d, *J* = 6.9 Hz), 119.6, 94.3 (d, *J* = 170.9 Hz), 36.9 (d, *J* = 23.8 Hz), 29.7, 28.4, 25.3, 24.3 (d, *J* = 4.4 Hz), 17.1; ^{19}F NMR (376 MHz, $CDCl_3$) δ –175.2; HRMS (ESI) Found MNa^+ 228.1160, $C_{13}H_{16}NFNa$ requires 228.1159.

2-(2-Fluoro-2,3-dihydro-1H-inden-1-yl)acetonitrile (3i)

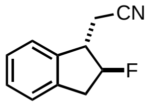

Following **GP9**, **1i** (26 mg, 0.1 mmol) gave **3i** (11 mg, 62%) as an oil. dr: 4:1. FT-IR ν_{max} (film)/cm^{-1} 2360, 1474, 1433, 1072, 870, 661; ^{1}H NMR (400 MHz, $CDCl_3$, diastereomers) δ 7.33–7.22 (4H, m), 5.55–5.37 (0.2H, m), 5.20 (0.8H, ddt, *J* = 52.4, 6.3, 3.8 Hz), 3.63 (0.8H, dtd, *J* = 22.0, 6.6, 3.8 Hz), 3.58–3.50 (0.2H, m), 3.43–3.28 (1H, m), 3.27–3.09 (1H, m), 2.86–2.73 (0.4H, m), 2.67 (1.6H, d, *J* = 6.7 Hz); ^{13}C NMR (101 MHz, $CDCl_3$, diastereomers) δ 139.9^m, 139.9^m, 139.5^M, 139.1^M, 128.8^M, 128.5^m, 127.9^M, 127.6^m, 125.4^M, 125.4^m, 124.2^M, 123.7^m, 117.9^M, 97.6 (d, *J* = 183.9 Hz)^M, 94.9 (H, d, *J* = 184.4 Hz)^m, 47.8 (d, *J* = 24.0 Hz)^M, 46.0 (d, *J* = 19.4 Hz)^m, 38.9 (d, *J* = 22.8 Hz)^m, 38.2 (d, *J* = 22.6 Hz)^M, 20.4 (d, *J* = 6.5 Hz)^M, 16.3 (d, *J* = 13.6 Hz)^m; ^{19}F NMR (376 MHz, $CDCl_3$) δ –176.88^M, –193.86^m. HRMS (ESI) Found MNa^+ 176.081, $C_{11}H_{10}NFNa$ requires 176.0876.

2-(4-Fluoro-1-tosylpyrrolidin-3-yl)acetonitrile (3j)

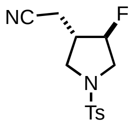

Following **GP9**, **1j** (19 mg, 0.1 mmol) gave **3j** (12 mg, 44%) as an oil. dr: 3.4:1. FT-IR ν_{max} (film)/cm^{-1} 2360, 1264, 1017, 908, 730, 704, 561; ^{1}H NMR (500 MHz, $CDCl_3$, diastereomers) δ 7.65 (1.4H, d, *J* = 8.3 Hz) & 7.64 (0.6H, d, *J* = 8.3 Hz), 7.29 (1.4H, d, *J* = 8.3 Hz) & 7.29 (0.6H, d, *J* = 8.3 Hz), 5.08–4.94 (0.3H, m) & 4.88 (0.7H, ddt, *J* = 6.8, 4.3, 2.0 Hz), 3.69 (0.3H, dd, *J* = 9.6, 7.4 Hz), 3.65–3.55 (0.7H, m), 3.47–3.34 (0.7H, m), 3.25–3.19

(1.7H, m), 2.95–2.88 (0.3H, m), 2.67–2.57 (0.7H, m), 2.49–2.39 (0.3H, m), 2.40–2.36 (0.3H, M), 2.38 (3H, s), 2.27 (1.7H, dd, J = 7.7, 4.7 Hz); ^{13}C NMR (126 MHz, $CDCl_3$, diastereomers) δ 144.7^{M}, 144.6^{m}, 133.6 ^{m}, 133.1^{M}, 130.4^{M}, 130.3 ^{m}, 128.0^{M}, 127.9^{m}, 117.4^{M}, 117.0^{m}, 94.15 (d, J = 184.8 Hz)^{M}, 92.3 (d, J = 184.2 Hz)^{m}, 54.4 (d, J = 23.1 Hz)^{m}, 52.6 (d, J = 24.8 Hz)^{M}, 50.4^{M}, 50.0^{m}, 41.8 (d, J = 23.2 Hz)^{M}, 40.8 (d, J = 19.4 Hz)^{m}, 22.0, 18.6 (d, J = 8.4 Hz)^{M}, 15.0 (d, J = 9.0 Hz)^{m}; ^{19}F NMR (376 MHz, $CDCl_3$) δ –174.5^{M}, –192.4^{m}; HRMS (ESI) Found MNa^+ 305.0730, $C_{13}H_{15}O_2N_2FNaS$ requires 305.0730.

3-(2-(1-Fluoroethyl)phenyl)propanenitrile (3k)

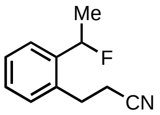

Following **GP9**, **1k** (26 mg, 0.1 mmol) gave **3k** (8 mg, 46%) as an oil. FT-IR ν_{max} (film)/cm^{-1} 2360, 1264, 907, 729, 704, 650; ^{1}H NMR ($CDCl_3$, 500 MHz) δ 7.43 (1H, d, J = 6.5 Hz), 7.37–7.30 (2H, m), 7.27 (1H, d, J = 6.3 Hz), 5.83 (1H, dq, J = 47.2, 6.4 Hz), 3.12–2.97 (2H, m), 2.71–2.57 (2H, m), 1.71 (3H, dd, J = 23.6, 6.4 Hz); ^{13}C NMR ($CDCl_3$, 126 MHz) δ 138.8 (d, J = 18.4 Hz), 136.2 (d, J = 3.2 Hz), 130.3, 129.4 (d, J = 2.3 Hz), 128.1, 126.6 (d, J = 6.8 Hz), 119.5, 88.9 (d, J = 166.1 Hz), 28.7, 22.2 (d, J = 25.4 Hz), 19.6 (d, J = 3.0 Hz); ^{19}F NMR (376 MHz, $CDCl_3$) δ –163.6 (s); HRMS (APCI) Found MK^+ 216.0585, $C_{11}H_{12}NFK$ requires 216.0591.

3-(2-(2-Fluoropropan-2-yl)phenyl)propanenitrile (3l)

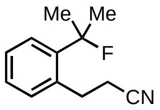

Following **GP9**, **1l** (28 mg, 0.1 mmol) gave **3l** (13 mg, 70%) as an oil. ^{1}H NMR (400 MHz, $CDCl_3$) δ 7.35 (1H, d, J = 7.6 Hz), 7.29–7.24 (1H, m), 7.21–7.17 (2H, m), 3.13–3.07 (2H, m), 2.72–2.66 (2H, m), 1.44 (6H, s); ^{19}F NMR (376 MHz, $CDCl_3$) δ –134.10 (s); HRMS (ESI) Found MNH_4^+ 209.1448, $C_{12}H_{18}FN_2$ requires 209.1454. Data in accordance with the literature.[17]

3-(2-(1-Fluorocyclohexyl)phenyl)propanenitrile (3m)

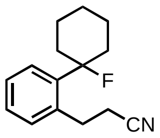

Following **GP9**, **1m** (32 mg, 0.1 mmol) gave **3m** (13 mg, 56%) as an oil. ^{1}H NMR (400 MHz, $CDCl_3$) δ 7.37–7.29 (2H, m), 7.07–7.11 (2H, m), 3.10 (2H, m), 2.56 (2H, t, *J* = 7.5 Hz), 1.83–1.60 (10H, m); ^{19}F NMR (376 MHz, $CDCl_3$) δ –157.7. Data in accordance with the literature.[17]

4-Fluoro-4-phenyl-1-tosylpiperidine (3n)

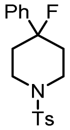

Following **GP9**, **1n** (46 mg, 0.1 mmol) gave **3n** (20 mg, 60%) as a solid. FT-IR ν_{max} (film)/cm^{-1} 3382, 2952, 1175, 954, 650, 633; ^{1}H NMR (400 MHz, $CDCl_3$) δ 7.69 (2H, d, *J* = 7.9 Hz), 7.40–7.28 (7H, m), 3.82 (2H, dd, *J* = 11.4, 3.9 Hz), 2.69 (2H, t, *J* = 11.6 Hz), 2.46 (3H, s), 2.25 (1H, td, *J* = 13.6, 4.8 Hz), 2.15 (1H, td, *J* = 13.6, 4.8 Hz), 2.03 (2H, t, *J* = 11.8 Hz); ^{13}C NMR (126 MHz, $CDCl_3$) δ 143.8, 143.27 (d, *J* = 21.2 Hz), 133.1, 129.8, 128.5, 128.0, 127.7, 123.7 (d, *J* = 9.3 Hz), 93.2 (d, *J* = 175.1 Hz), 42.3 (d, *J* = 1.2 Hz), 36.2 (d, *J* = 22.5 Hz), 21.57; ^{19}F NMR (376 MHz, $CDCl_3$) δ –162.4; HRMS (ESI) Found MNa$^+$ 356.1078, $C_{18}H_{20}O_2NFNaS$ requires 356.1091.

***tert*-Butyl 4-Fluoro-4-phenylpiperidine-1-carboxylate (3o)**

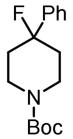

Following **GP9**, **1o** (40 mg, 0.1 mmol) gave **3o** (18 mg, 64%) as an oil. FT-IR ν_{max} (film)/cm^{-1} 3314, 2343, 1022, 667, 620; ^{1}H NMR (500 MHz, $CDCl_3$) δ 7.41–7.35 (4H, m), 7.34–7.28 (1H, m), 4.12 (2H, br s), 3.18 (2H, br s), 2.07–1.89 (4H, m), 1.49 (9H, s); ^{13}C NMR (126 MHz, $CDCl_3$) δ 154.8, 144.1 (d, *J* = 21.4 Hz), 128.4 (d, *J* = 0.7 Hz), 127.7 (d, *J* = 1.1 Hz), 123.9 (d, *J* = 9.2 Hz), 94.3 (d, *J* = 174.5 Hz), 79.7, 36.9, 36.6, 28.5; ^{19}F NMR (376 MHz, $CDCl_3$) δ –162.7; HRMS (ESI) Found MNa$^+$ 302.1515, $C_{16}H_{22}O_2NFNa$ requires 302.1513.

2-((1*R*,3*S*)-3-Fluoro-2,2,3-trimethylcyclopentyl)acetonitrile (3p)

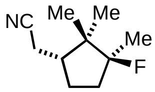

Following **GP9**, **1p** (25 mg, 0.1 mmol) gave **3p** (12 mg, 68%) as an oil. dr: 4:1.

Data for major diastereomer: ^{1}H NMR (500 MHz, $CDCl_3$) δ 2.43–2.34 (2H, m), 2.25–2.20 (1H, m), 2.19–2.10 (1H, m), 2.03–1.79 (2H, m), 1.46–1.36 (1H, m), 1.30 (3H, d, *J* = 22.2 Hz), 1.02 (3H, d, *J* = 1.4 Hz), 0.70 (3H, s); ^{13}C NMR (126 MHz, $CDCl_3$) 119.4, 96.6 (d, *J* = 171.5 Hz), 43.2, 34.9 (d, *J* = 24.0 Hz), 26.5, 19.1, 19.0, 18.77 (d, *J* = 26.4 Hz), 18.31 (d, *J* = 5.8 Hz), 17.8; ^{19}F NMR (376 MHz, $CDCl_3$) δ –143.1; HRMS (ESI) Found MH^+ 170.1335, $C_{10}H_{17}NF$ requires 170.1340. Data in accordance with literature.[17]

(4b*S*,7*S*,8*S*,8a*R*)-8-(2-Cyanoethyl)-7-fluoro-7-methyl-4b,5,6,7,8,8a,9,10-octahydro phenanthren-2-yl Acetate (3q)

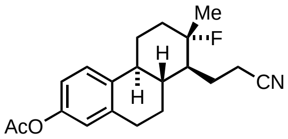

Following **GP9**, **1q** (41 mg, 0.1 mmol) gave **3q** (24 mg, 72%) as an oil. dr 1.3:1. ^{1}H NMR (400 MHz, $CDCl_3$) δ 7.28 (1H, d, *J* = 8.5 Hz), 6.85 (1H, d, *J* = 8.5 Hz), 6.80 (1H, s), 2.62–2.54 (2H, m), 2.44–2.36 (2H, m), 2.28 (3H, s), 2.14–2.05 (2H, m), 1.99–1.86 (4H, m), 1.66–1.58 (2H, m), 1.57–1.41 (1H, m), 1.32 (3H, d, *J* = 22.4 Hz), 1.23–1.09 (2H, m); ^{19}F NMR (376 MHz, $CDCl_3$) δ -130.2, -159.5; HRMS (ESI) Found MH^+ 170.1335, $C_{10}H_{17}NF$ requires 170.1340. Data in accordance with the literature.[17]

(2*R*,4a*S*,4b*S*,7*S*,8*S*,8a*R*)-8-(2-Cyanoethyl)-7-fluoro-4a,7-dimethyltetradecahydro phenanthren-2-yl Acetate (3r)

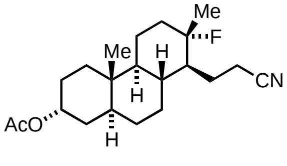

Following **GP9**, **1r** (43 mg, 0.1 mmol) gave **3r** (18 mg, 52%) as an oil. dr 3:1. FT-IR ν_{max} (film)/cm^{-1} 2995, 1266, 1174, 1130, 986, 951; ^{1}H NMR (400 MHz, $CDCl_3$) δ 4.68–4.56 (1H, m), 2.51–2.32^{M} (1.6H, m), 2.30–2.19^{m} (0.4H, m), 1.89–1.81 (2H, m), 1.81–1.73 (2H, m), 1.72–1.65 (2H, m), 1.61–1.52 (2H, m), 1.49–1.37 (m, 2H), 1.37–1.06 (12H, m), 1.04–0.88 (3H, m), 0.73^{m} (0.8H, s), 0.69^{M} (2.2H, s); ^{13}C NMR (126 MHz, $CDCl_3$)^{b} δ 170.7^{m}, 170.7^{M},

120.3^{m}, 120.2^{M}, 98.4^{M} (d, J = 169.7 Hz), 96.2^{m} (d, J = 170.5 Hz), 73.5^{m}, 73.3^{M}, 52.7^{M}, 52.5^{m}, 50.6^{M} (d, J = 17.0 Hz), 49.0^{m} (d, J = 21.0 Hz), 43.9^{m}, 43.9^{M}, 39.4 (d, J = 9.3 Hz), 39.1^{M} (d, J = 21.1 Hz), 38.4^{m} (d, J = 22.4 Hz), 36.6^{M}, 36.6^{m}, 35.6 (d, J = 16.4 Hz), 35.5, 34.9, 33.7^{m}, 33.6^{M}, 31.4^{M}, 31.2^{m}, 28.3^{M}, 28.2^{m}, 27.3^{M}, 27.3^{m}, 25.9^{m} (d, J = 24.9 Hz), 24.4^{M}, 23.1^{m}, 22.6^{M} (d, J = 12.4 Hz), 21.5^{m}, 21.4^{M}, 20.2^{M}, 20.2^{m}, 19.3^{M} (d, J = 25.4 Hz), 17.5^{M} (d, J = 6.5 Hz), 14.7^{m} (d, J = 5.6 Hz), 12.1^{m}, 12.0^{M}; ^{19}F NMR (376 MHz, $CDCl_3$) δ –129.5^{M}, –159.6^{m}; HRMS (ESI) Found MNa^+ 372.2301, $C_{21}H_{32}O_2NFNa$ requires 372.2309.

(1b*R*,3*S*,5a*R*,5b*S*,8*S*,9*S*,9a*R*)-9-((1*S*)-2-Cyanocyclopropyl)-8-fluoro-1b-hydroxy-5a,8-dimethyltetradecahydro-1H-cyclopropa[l]phenanthren-3-yl Acetate (3s)

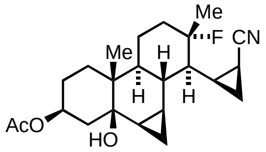

Following **GP9**, **1s** (47 mg, 0.1 mmol) gave **3s** (20 mg, 51%) as an oil. dr 3:1.

Data for the major isomer: FT-IR ν_{max} (film)/cm^{-1} 2925, 1728, 1249, 905, 729, 649; ^{1}H NMR (400 MHz, $CDCl_3$) δ 5.08–5.02 (1H, m), 2.21 (1H, dd, J = 14.3, 4.3 Hz), 2.07 (3H, s), 1.98–1.92 (2H, m), 1.80–1.70 (1H, m), 1.52–1.42 (2H, m), 1.46 (3H, d, J = 21.5 Hz), 1.42–1.37 (2H, m), 1.23–1.14 (1H, m), 1.13–1.06 (2H, m), 1.03–0.97 (1H, m), 0.95 (3H, s), 0.91–0.77 (3H, m), 0.74–0.64 (2H, m); ^{13}C NMR (126 MHz, $CDCl_3$) δ 170.1, 121.7, 96.2 (d, J = 171.9 Hz), 71.9, 70.8, 53.4, 50.3 (d, J = 20.7 Hz), 41.7, 40.7, 39.1, 38.7 (d, J = 21.9 Hz), 29.7, 26.8 (d, J = 25.5 Hz), 25.1, 22.1, 21.5, 20.3 (d, J = 2.6 Hz), 17.7, 14.2, 13.7, 10.3, 3.1; ^{19}F NMR (376 MHz, $CDCl_3$) δ –161.5; HRMS (ESI) Found MNa^+ 412.2247, $C_{23}H_{32}O_3NFNa$ requires 412.2258.

Methyl (1*R*,4as,4br,7*R*,8ar)-8a-(Cyanomethyl)-7-fluoro-1,4a,7-trimethyltetradecahydro phenanthrene-1-carboxylate (3t)

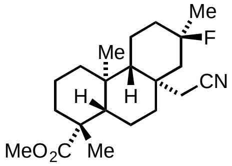

Following **GP9**, **1t** (43 mg, 0.1 mmol) gave **3t** (11 mg, 32%) as an oil. FT-IR ν_{max} (film)/cm^{-1} 2970, 2923, 1711, 1360, 1260, 1220, 1090, 1023, 917, 917, 802, 731; ^{1}H NMR (500 MHz, $CDCl_3$) δ 3.65 (3H, s), 2.90 (1H, d, J = 15.2 Hz), 2.65 (1H, d, J = 0.4 Hz), 2.28 (1H, ddd, J = 15.1, 9.0, 2.5 Hz), 2.21–2.12 (2H, m, J = 6.5 Hz), 2.08–1.98 (2H, m), 1.92–1.36 (7H, m),

1.32 (3H, d, *J* = 21.3 Hz), 1.18 (3H, s), 1.14–0.80 (6H, m), 0.62 (3H, s); ^{13}C NMR (126 MHz, $CDCl_3$) δ 177.6, 119.6, 95.3, 57.33, 56.9, 51.5, 49.5 (d, *J* = 20.3 Hz), 43.9, 40.2, 39.9, 38.1, 38.1 (d, *J* = 23.2 Hz), 37.6, 36.7, 28.7, 28.2 (d, *J* = 25.2 Hz), 21.7 (d, *J* = 9.1 Hz), 19.28, 19.1, 17.3, 14.1; ^{19}F NMR (471 MHz, $CDCl_3$) δ –144.87; HRMS (ESI) Found MNa^+ 372.2309, $C_{21}H_{32}O_2NFNa$ requires 372.2315.

5.2 Cascade Ring-Opening-Chlorination

General Procedure for the Reaction Optimization – GP10

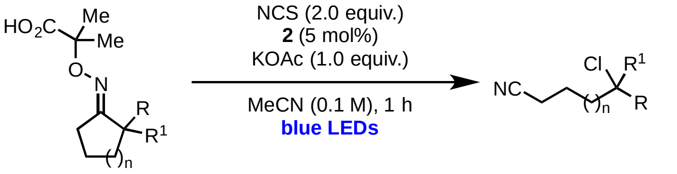

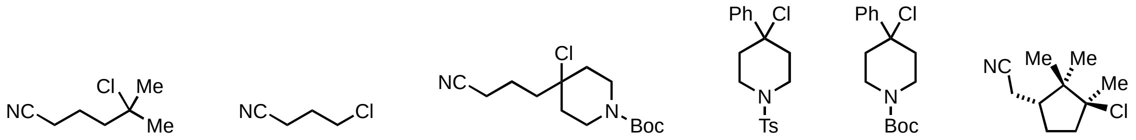

4a **4b** **4c** **4d** **4e** **4f**

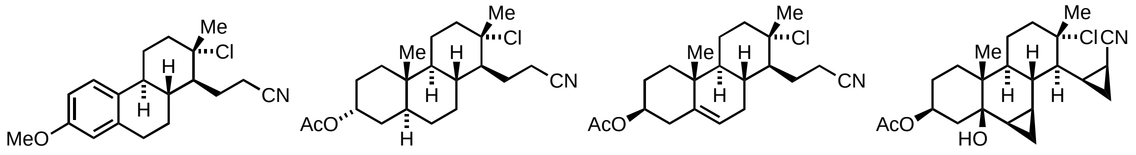

4g **4h** **4i** **4j**

A dry tube equipped with a stirring bar was charged with the oxime (1.0 equiv.), **2** (5 mol%), KOAc (0.1 mmol, 1.0 equiv.) and NCS (0.2 mmol, 2.0 equiv.). The tube was sealed, evacuated and back-filled with nitrogen three times. MeCN (0.1 M) was added, the blue LEDs were switched on and the reaction was stirred under irradiation for 1 h. The mixture was dilute with H_2O (1 mL) and EtOAc (1 mL). The layers were separated and the aqueous layer was extracted with EtOAc (3 x 5 mL). The combined organic layers were dried ($MgSO_4$), filtered and evaporated. The residue was purified by column chromatography on silica gel.

5-Chloro-5-methylhexanenitrile (4a)

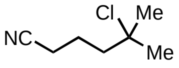

Following **GP10**, **1a** (21 mg, 0.1 mmol) gave **4a** (14 mg, 94%) as an oil. FT-IR ν_{max} (film)/cm^{-1} 2924, 1372, 957; ^{1}H NMR (400 MHz, $CDCl_3$) δ 2.43–2.38 (2H, m), 1.92–1.84 (4H, m), 1.60 (6H, s); ^{13}C NMR (126 MHz, $CDCl_3$) δ 119.4, 69.6, 44.6, 32.5, 21.3, 17.2; HRMS (ASAP) Found MH^+ 146.0727, $C_7H_{13}NCl$ requires 146.07321.

4-Chlorobutanenitrile (4b)

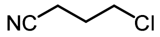

Following **GP10**, **1b** (17 mg, 0.1 mmol) gave **4b** (6 mg, 59%) as an oil. ^{1}H NMR (500 MHz, CD_3CN) δ 3.73–3.66 (2H, m), 2.61–2.55 (4H, m); ^{13}C NMR (126 MHz, CD_3CN) δ 119.6, 44.2, 28.7, 15.2. Data in accordance with a commercially available sample [CAS: 628-20-6].

***tert*-Butyl 4-Chloro-4-(3-cyanopropyl)piperidine-1-carboxylate (4c)**

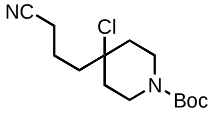

Following **GP10**, **1e** (35 mg, 0.1 mmol) gave **4c** (14 mg, 50%) as an oil. FT-IR ν_{max} (film)/cm^{-1} 3055, 2986, 1264, 908,731, 704; ^{1}H NMR (400 MHz, $CDCl_3$) δ 3.92 (2H, br s), 3.07 (2H, br s), 2.36 (2H, t, *J* = 6.1 Hz), 1.88–1.81 (5H, m), 1.79 (1H, br s), 1.62–1.52 (2H, m), 1.39 (9H, s); ^{13}C NMR (126 MHz, $CDCl_3$) δ 154.9, 119.6, 80.2, 72.3, 44.6, 40.6, 39.8, 28.8, 20.3, 17.7; HRMS (ESI) Found MNa^+ 309.1340, $C_{14}H_{23}O_2N_2ClNa$ requires 309.1340.

4-Chloro-4-phenyl-1-tosylpiperidine (4d)

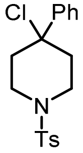

Following **GP10**, **1n** (46 mg, 0.1 mmol) gave **4d** (25 mg, 71%) as a solid. FT-IR ν_{max} (film)/cm^{-1} 2950, 2250, 1711, 1541, 1374, 1346, 1248, 1163, 1045, 904, 723, 648; ^{1}H NMR (400 MHz, $CDCl_3$) δ 7.70 (2H, d, *J* = 7.8 Hz), 7.50 (2H, d, *J* = 7.7 Hz), 7.40–7.29 (5H, m), 3.86–3.81 (2H, m), 2.96–2.86 (2H, m), 2.46 (3H, s), 2.37–2.28 (4H, m); ^{13}C NMR (101 MHz, $CDCl_3$) δ 144.5, 143.8, 133.3, 129.8, 128.6, 127.7, 125.3, 125.0, 70.9, 42.7, 38.9, 21.6; HRMS (ESI) Found MNa^+ 372.0783, $C_{18}H_{20}O_2NClNaS$ required 372.0795.

***tert*-Butyl 4-Chloro-4-phenylpiperidine-1-carboxylate (4e)**

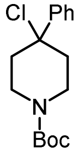

Following **GP10**, **1o** (40 mg, 0.1 mmol) gave **4e** (19 mg, 66%) as an oil. FT-IR ν_{max} (film)/cm^{-1} 3320, 2295, 1113, 954, 830, 679; ^{1}H NMR (400 MHz, $CDCl_3$) δ 7.59–7.51 (2H, m), 7.39 (2H, t, *J* = 7.5 Hz), 7.31 (1H, t, *J* = 7.4 Hz), 4.10 (2H, br. s), 3.35 (2H, br.s), 2.33–

2.26 (2H, m), 2.18–2.07 (2H, m), 1.47 (9H, s); ^{13}C NMR (126 MHz, $CDCl_3$) δ 145.1, 128.5, 128.1, 125.4, 71.9, 28.5.

2-((1*R*,3*S*)-3-Chloro-2,2,3-trimethylcyclopentyl)acetonitrile (4f)

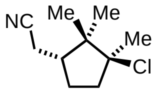

Following **GP10**, **1p** (25 mg, 0.1 mmol) gave **4f** (13 mg, 69%) as an oil. dr: 4:1.

Data for major diastereomer: FT-IR ν_{max} (film)/cm^{-1} 2950, 2400, 2310, 1475, 903, 724; ^{1}H NMR (500 MHz, $CDCl_3$) δ 2.66–2.58 (1H, m), 2.41–2.34 (1H, m), 2.29–2.19 (3H, m), 2.11–2.03 (1H, m), 1.58 (3H, s), 1.49–1.41 (1H, m), 1.13 (3H, s), 0.80 (3H, s); ^{13}C NMR (126 MHz, $CDCl_3$) δ 119.3, 86.0, 48.8, 43.3, 40.2, 26.6, 25.1, 22.1, 18.7, 18.3; HRMS (APCI) Found MH^+ 186.1040, $C_{10}H_{17}NCl$ requires 186.1044.

3-((1*S*,2*S*,4a*S*,10a*R*)-2-Chloro-7-methoxy-2-methyl-1,2,3,4,4a,9,10,10a-octahydrophenanthren-1-yl)propanenitrile (4g)

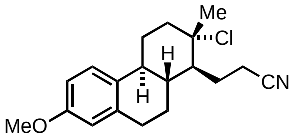

Following **GP10**, **1v** (39 mg, **0.1** mmol) gave **4g** (17 mg, 53%) as an oil. FT-IR ν_{max} (film)/cm^{-1} 2963, 1201, 1145, 957, 811, 752; ^{1}H NMR (400 MHz, $CDCl_3$) δ 7.21 (1H, d, *J* = 8.6 Hz), 6.73 (1H, d, *J* = 8.3 Hz), 6.63 (1H, s), 3.78 (3H, s, 2.94–2.84 (2H, m), 2.65–2.49 (1H, m), 2.47–2.27 (3H, m), 2.26–2.08 (2H, m), 2.07–1.97 (1H, m), 1.88–1.72 (3H, m), 1.69 (3H, s), 1.73–1.61 (1H, m), 1.58–1.38 (2H, m); ^{13}C NMR (126 MHz, $CDCl_3$) δ 157.7, 137.4, 131.4, 126.6, 119.7, 113.4, 112.0, 75.1, 55.2, 51.8, 42.7, 42.7, 41.6, 32.0, 30.2, 26.8, 26.7, 25.5, 18.2; HRMS (ASAP) Found MH^+ 318.1619, $C_{19}H_{25}ONCl$ requires 318.1619.

(2*R*,4a*S*,4b*S*,7*S*,8*S*,8a*R*)-7-Chloro-8-(2-cyanoethyl)-4a,7-dimethyltetradecahydro phenanthren-2-yl Acetate (4h)

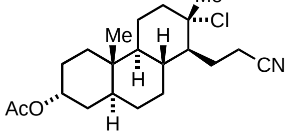

Following **GP10**, **1r** (43 mg, 0.1 mmol) gave **4h** (31 mg, 87%) as an oil. FT-IR ν_{max} (film)/cm^{-1} 2972, 2925, 1295, 1154, 1130, 952, 815; ^{1}H NMR (400 MHz, $CDCl_3$) δ 4.75–4.63 (1H, m), 2.58–2.46 (1H, m), 2.38–2.29 (1H, m), 2.10–2.03 (2H, m), 2.02 (3H, s), 1.90–

1.74 (3H, m), 1.70–1.57 (4H, m), 1.61 (3H, s), 1.52–1.13 (8H, m), 1.07–0.93 (3H, m), 0.82 (3H, s); ^{13}C NMR (126 MHz, $CDCl_3$) δ 170.7, 119.7, 92.9, 75.5, 73.4, 52.9, 52.6, 44.0, 42.5, 38.3, 36.5, 35.7, 33.7, 31.9, 31.3, 28.3, 27.3, 25.9, 21.5, 21.0, 18.0, 12.1; HRMS (ESI) Found MNa^+ 388.2001, $C_{21}H_{32}O_2NClNa$ requires 388.2014.

(2*S*,4a*R*,4b*S*,7*S*,8*S*,8a*R*)-7-Chloro-8-(2-cyanoethyl)-4a,7-dimethyl-1,2,3,4,4a,4b,5,6,7,8,8a,9-dodecahydrophenanthren-2-yl Acetate (4i)

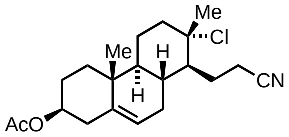

Following **GP10**, **1s** (43 mg, 0.1 mmol) gave **4i** (23 mg, 62%) as an oil. dr 4:1. FT-IR ν_{max} (film)/cm^{-1} 2969, 2929, 1466, 1378, 1306, 1160, 1128, 950, 816; ^{1}H NMR (500 MHz, $CDCl_3$) δ 5.38 (1H, d, *J* = 5.2 Hz), 4.64–4.57 (1H, m), 2.60–2.52 (1H, m), 2.44–2.36 (1H, m), 2.36–2.27 (2H, m), 2.24–2.15 (1H, m), 2.14–2.04 (2H, m), 2.04 (3H, s), 1.92–1.84 (2H, m), 1.80–1.65 (4H, m), 1.62 (3H, s), 1.65–1.58 (2H, m), 1.17–1.05 (4H, m), 1.03 (3H, s), 0.91–0.80 (2H, m); ^{13}C NMR (126 MHz, $CDCl_3$) δ 170.5, 139.5, 121.3, 119.7, 75.3, 73.5, 48.7, 44.0, 37.6, 37.0, 36.9, 36.7, 32.3, 29.7, 27.5, 26.6, 23.4, 22.7, 21.4, 19.2, 18.7; HRMS (ASAP neg) Found M-H^+ 362.1883, $C_{21}H_{29}ClNO_2$ requires 362.1892.

(1a*S*,1b*R*,3*S*,5a*R*,5b*S*,8*S*,9*S*,9a*R*,9b*S*)-8-Chloro-9-((1*S*)-2-cyanocyclopropyl)-1b-hydroxy-5a,8-dimethyltetradecahydro-1H-cyclopropa[l]phenanthren-3-yl Acetate (4j)

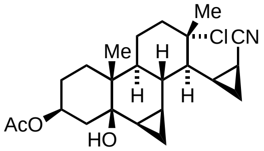

Following **GP10**, **1t** (47 mg, 0.1 mmol) gave **4j** (16 mg, 40%) as an oil. dr 3:1. FT-IR ν_{max} (film)/cm^{-1} 1710, 1357, 12220, 917, 731, 532; ^{1}H NMR (400 MHz, $CDCl_3$) δ 5.04 (1H, s), 2.24–2.14 (2H, m), 2.10–2.02 (1H, m), 2.07 (3H, s), 1.98–1.87 (2H, m), 1.69–1.58 (4H, m), 1.61 (3H, s), 1.52–1.39 (2H, m), 1.38–1.16 (2H, m), 1.16–0.95 (4H, m), 0.92 (3H, s), 0.91–0.77 (2H, m), 0.74–0.64 (1H, m), 0.64–0.58 (1H, m); ^{13}C NMR (101 MHz , $CDCl_3$) δ 170.2, 121.4, 75.0, 72.0, 70.8, 53.1, 45.4, 42.4, 42.0, 40.7, 29.8, 25.3, 25.0, 24.6, 21.8, 21.6, 17.7, 14.9, 12.2, 10.2, 4.0, 3.7; HRMS (ESI) Found MK^+ 444.1702, $C_{23}H_{32}O_3NClK$ requires 444.1702.

5.3 Cascade Ring Opening-Azidation

General Procedure for the Reaction Optimization – GP11

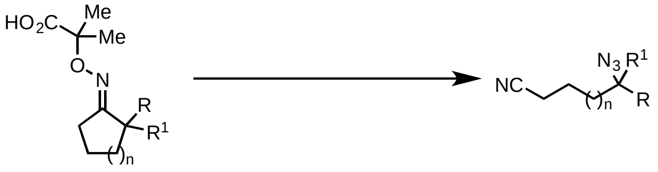

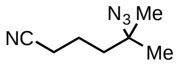

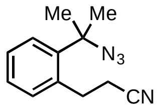

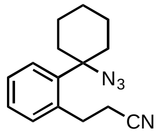

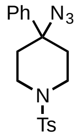

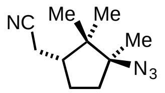

5a **5b** **5c** **5d** **5e**

A dry tube equipped with a stirring bar was charged with the oxime (1.0 equiv.), **2** (5 mol%), Cs_2CO_3 (0.1 mmol, 1.0 equiv.) and 2,4,6-triisopropylbenzenesulfonyl azide (0.2 mmol, 2.0 equiv.). The tube was sealed, evacuated and back-filled with nitrogen three times. CH_2Cl_2 (0.5 M) was added, the blue LEDs were switched on and the reaction was stirred under irradiation for 1 h. The mixture was dilute with H_2O (1 mL) and EtOAc (1 mL). The layers were separated and the aqueous layer was extracted with EtOAc (3 x 5 mL). The combined organic layers were dried ($MgSO_4$), filtered and evaporated. The residue was purified by column chromatography on silica gel.

5-Azido-5-methylhexanenitrile (5a)

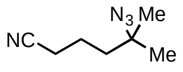

Following **GP11**, **1a** (21 mg, 0.1 mmol) gave **5a** (10 mg, 64%) as an oil. ^{1}H NMR (400 MHz, $CDCl_3$) δ 2.45 (2H, t, J = 7.5 Hz), 1.78 – 1.76 (2H, m), 1.52 (2H, t, J = 6.8 Hz), 1.07 (6H, s); ^{13}C NMR (126 MHz, $CDCl_3$) δ 117.7, 64.7, 34.7, 29.5, 26.7, 25.1; HRMS (ESI) found MH^+ 153.1132, $C_7H_{13}N_4$ requires 153.1135.

3-(2-(2-Azidopropan-2-yl)phenyl)propanenitrile (5b)

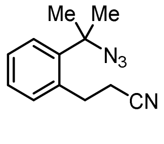

Following **GP11**, **1l** (28 mg, 0.1 mmol) gave **5b** (11 mg, 52%) as an oil. ^{1}H NMR (400 MHz, $CDCl_3$) δ 7.43 (1H, d, J = 7.9 Hz), 7.29–7.24 (2H, m), 7.10 (1H, d, J = 7.1 Hz), 2.88 (2H, t, J = 6.9 Hz), 2.59 (2H, t, J = 6.2 Hz), 1.60 (6H, s); ^{13}C NMR (101 MHz, $CDCl_3$) δ 154.8, 136.2, 127.4, 126.8, 126.0, 125.6, 124.1, 66.8, 29.9, 24.8, 23.5.

3-(2-(1-Azidocyclohexyl)phenyl)propanenitrile (5c)

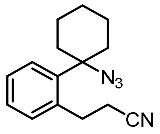

Following **GP11**, **1m** (32 mg, 0.1 mmol) gave **5c** (16 mg, 63%) as an oil. FT-IR ν_{max} (film)/cm^{-1} 2947, 2830, 1451, 1022, 757; ^{1}H NMR (500 MHz, $CDCl_3$) δ 7.30 (1H, d, *J* = 7.9 Hz), 7.26–7.23 (2H, m), 7.22–7.17 (1H, m), 3.33–3.26 (2H, m), 2.60–2.54 (2H, m), 1.76–1.59 (10H, m); ^{13}C NMR (126 MHz, $CDCl_3$) δ 141.9, 137.5, 131.5, 128.2, 127.2, 126.4, 119.4, 66.7, 35.9, 29.7, 25.2, 22.3, 19.5; HRMS (ESI) Found MNa$^+$ 277.1414, $C_{15}H_{18}N_4Na$ requires 277.1424.

4-Azido-4-phenyl-1-tosylpiperidine (5d)

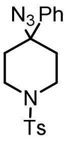

Following **GP11** but using HFIP as the solvent, **1n** (46 mg, 0.1 mmol) gave **5d** (23 mg, 64%) as a solid. FT-IR ν_{max} (film)/cm^{-1} 2979, 2334, 1161, 730, 620; ^{1}H NMR (400 MHz, $CDCl_3$) δ 7.68 (2H, d, *J* = 8.2 Hz), 7.42–7.39 (3H, m), 7.37–7.27 (4H, m), 3.77–3.67 (2H, m), 2.79–2.67 (2H, m), 2.45 (3H, s), 2.30–2.22 (2H, m), 2.21–2.08 (2H, m); ^{13}C NMR (126 MHz, $CDCl_3$) δ 143.6, 142.6, 133.1, 129.8, 129.0, 128.3, 127.7, 125.2, 81.7, 42.1, 32.9, 21.6; HRMS (ESI) Found MNa$^+$ 379.1199, $C_{18}H_{20}O_2N_4NaS$ requires 379.1199.

2-(3-Azido-2,2,3-trimethylcyclopentyl)acetonitrile (5e)

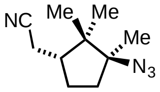

Following **GP11**, **1p** (25 mg, 0.1 mmol) gave **5e** (17 mg, 86%) as an oil. FT-IR ν_{max} (film)/cm^{-1} 2966, 2102, 1266, 1089, 757; ^{1}H NMR (500 MHz, $CDCl_3$) δ 2.38–2.28 (2H, m), 2.23–2.16 (1H, m), 2.16–2.06 (1H, m), 1.95 (1H, ddd, *J* = 14.0, 9.7, 4.0 Hz), 1.81 (1H, ddd, *J* = 14.4, 11.8, 6.2 Hz), 1.47–1.37 (1H, m), 1.30 (3H, s), 0.97 (3H, s), 0.73 (3H, s); ^{13}C NMR (126 MHz, $CDCl_3$) δ 119.3, 75.4, 47.0, 43.3, 33.8, 26.5, 20.2, 18.3, 18.0, 17.9; HRMS (ESI): Found MNa$^+$ 215.1266, $C_{10}H_{16}N_4Na$ requires 215.1267.

5.4 Cascade 1,5-H Abstraction-Chlorination

General Procedure for the Reaction Optimization – GP12

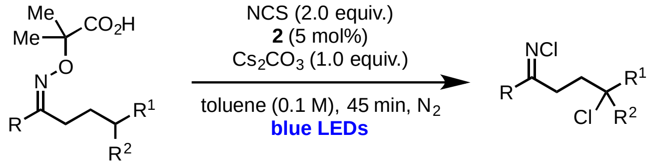

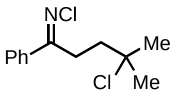

7a

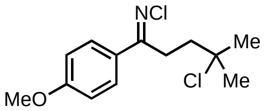

7b

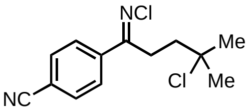

7c

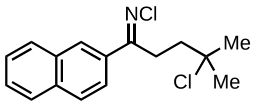

7d

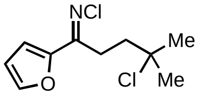

7e

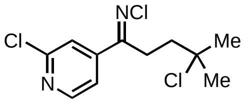

7f

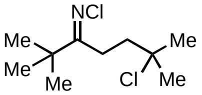

7g

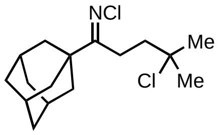

7h

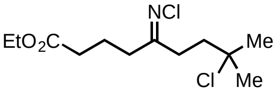

7i

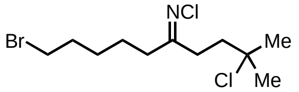

7j

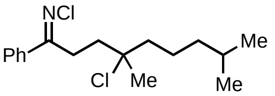

7k

A dry tube equipped with a stirring bar was charged with the oxime (1.0 equiv.), **2** (5 mol%), Cs_2CO_3 (0.1 mmol, 1.0 equiv.) and NCS (0.2 mmol, 2.0 equiv.). The tube was sealed, evacuated and back-filled with nitrogen three times. Toluene (0.1 M) was added, the blue LEDs were switched on and the reaction was stirred under irradiation for 45 min. The mixture was dilute with H_2O (1 mL) and EtOAc (1 mL). The layers were separated and the aqueous layer was extracted with EtOAc (3 x 5 mL). The combined organic layers were dried ($MgSO_4$), filtered and evaporated. The residue was purified by column chromatography on silica gel.

N,4-Dichloro-4-methyl-1-phenylpentan-1-imine (7a)

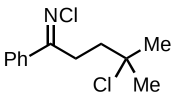

Following **GP12**, **6a** (27 mg, 0.1 mmol) gave **7a** (17 mg, 69%) as an oil. FT-IR ν_{max} (film)/cm^{-1} 2962, 1652, 1444, 1295, 978, 898, 763, 663; ^{1}H NMR (400 MHz, $CDCl_3$) δ 7.77–7.73 (2H, m), 7.48–7.40 (3H, m), 3.34–3.26 (2H, m), 2.06–1.98 (2H, m), 1.67 (6H, s); ^{13}C NMR (101 MHz, $CDCl_3$) δ 179.6, 136.0, 131.2, 129.0, 127.3, 70.1, 41.2, 32.4, 29.9; HRMS (APCI) Found MH^+ 244.0646, $C_{12}H_{16}NCl_2$ required 244.0654.

N,4-Dichloro-1-(4-methoxyphenyl)-4-methylpentan-1-imine (7b)

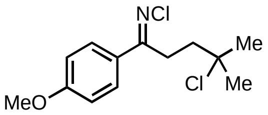

Following **GP12**, **6b** (31 mg, 0.1 mmol) gave **7b** (19 mg, 70%) as an oil. FT-IR ν_{max} (film)/cm^{-1} 2955, 1651, 1603, 1473, 1292, 979, 679; ^{1}H NMR (400 MHz, $CDCl_3$) δ 7.74 (2H, d, J = 9.4 Hz), 6.93 (2H, d, J = 9.4 Hz), 3.85 (3H, s), 3.30–3.22 (2H, m), 2.04–1.97 (2H, m), 1.67 (6H, s); ^{13}C NMR (101 MHz, $CDCl_3$) δ 178.4, 161.9, 128.8, 128.2, 114.1, 70.1, 55.4, 41.2, 32.3, 29.8; HRMS (APCI) Found MH$^+$ 274.0756, $C_{13}H_{18}ONCl_2$ requires 274.0760.

4-(4-Chloro-1-(chloroimino)-4-methylpentyl)benzonitrile (7c)

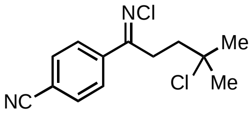

Following **GP12**, **6c** (30 mg, 0.1 mmol) gave **7c** (13 mg, 49%) as an oil. FT-IR ν_{max} (film)/cm^{-1} 2956, 2879, 2229, 1650, 1279, 970, 781; ^{1}H NMR (500 MHz, $CDCl_3$) δ 7.88 (2H, d, J = 8.5 Hz), 7.73 (2H, d, J = 8.5 Hz), 3.33–3.28 (2H, m), 2.01–1.97 (2H, m), 1.67 (6H, s); ^{13}C NMR (126 MHz, $CDCl_3$) δ 178.2, 139.8, 132.7, 127.9, 118.2, 114.8, 69.9, 40.9, 32.4, 29.9; HRMS (APCI) Found MH$^+$ 269.0601, $C_{13}H_{15}N_2Cl_2$ requires 269.0607.

N,4-Dichloro-4-methyl-1-(naphthalen-2-yl)pentan-1-imine (7d)

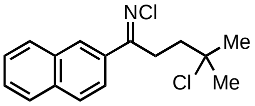

Following **GP12**, **6d** (33 mg, 0.1 mmol) gave **7d** (19 mg, 65%) as an oil. FT-IR ν_{max} (film)/cm^{-1} 2967, 1656, 1442, 1153, 970, 754, 675; ^{1}H NMR (500 MHz, $CDCl_3$) δ 8.22 (1H, br s), 7.94–7.90 (2H, m), 7.89–7.85 (2H, m), 7.55 (2H, tdd, J = 8.3, 5.1, 1.4 Hz), 3.49–3.36 (2H, m), 2.14–2.04 (2H, m), 1.70 (6H, s); ^{13}C NMR (126 MHz, $CDCl_3$) δ 179.2, 134.4, 133.0, 132.9, 129.1, 128.6, 127.8, 127.7, 127.7, 126.8, 123.7, 70.2, 41.2, 32.3, 29.9; HRMS (APCI) Found MH$^+$ 294.0799, $C_{16}H_{18}NCl_2$ requires 294.0811.

N,4-Dichloro-1-(furan-2-yl)-4-methylpentan-1-imine (7e)

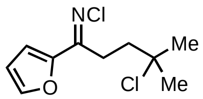

Following **GP12**, **6e** (27 mg, 0.1 mmol) gave **7e** (15 mg, 66%) as an oil. FT-IR ν_{max} (film)/cm^{-1} 2955, 1661, 1399, 1170, 970, 668; ^{1}H NMR (500 MHz, $CDCl_3$) δ 7.85 (1H, d, *J* = 3.7 Hz), 7.57 (1H, d, *J* = 1.6 Hz), 6.62 (1H, dd, *J* = 3.7, 1.7 Hz), 3.18–3.13 (2H, m), 2.15–2.09 (2H, m), 1.64 (6H, s); ^{13}C NMR (126 MHz, $CDCl_3$) δ 166.8, 146.4, 144.1, 120.3, 112.4, 69.9, 42.6, 33.5, 32.4; HRMS (ESI) Found MH$^+$ 234.0449, $C_{10}H_{14}ONCl_2$ requires 234.0447.

***N*,4-Dichloro-1-(2-chloropyridin-4-yl)-4-methylpentan-1-imine (7f)**

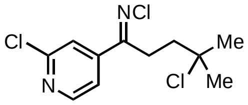

Following **GP12**, **6f** (31 mg, 0.1 mmol) gave **7f** (13 mg, 45%) as an oil. ^{1}H NMR (400 MHz, $CDCl_3$) δ 8.49 (1H, dd, *J* = 5.2, 0.8 Hz), 7.71–7.66 (1H, m), 7.55 (1H, dd, *J* = 5.2, 1.5 Hz), 3.31–3.22 (2H, m), 2.02–1.94 (2H, m), 1.67 (6H, s); ^{13}C NMR (101 MHz, $CDCl_3$) δ 176.9, 152.7, 150.5, 145.5, 121.9, 119.7, 69.6, 40.6, 32.2, 29.5.

***N*,6-dichloro-2,2,6-trimethylheptan-3-imine (7g)**

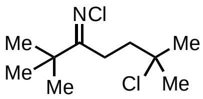

Following **GP12**, **6g** (27 mg, 0.1 mmol) gave **7g** (13 mg, 58%) as an oil. FT-IR ν_{max} (film)/cm^{-1} 2986, 2870, 1642, 1474, 1215, 968, 672; ^{1}H NMR (400 MHz, $CDCl_3$) δ 2.83–2.75 (2H, m), 2.08–2.01 (2H, m), 1.65 (6H, s), 1.16 (9H, s); ^{13}C NMR (126 MHz, $CDCl_3$) δ 188.9, 70.7, 43.8, 40.8, 32.6, 29.3, 28.2; HRMS (APCI) Found MH$^+$ 224.0964, $C_{10}H_{20}NCl_2$ requires 224.0967.

1-((3r,5r,7r)-Adamantan-1-yl)-N,4-dichloro-4-methylpentan-1-imine (7h)

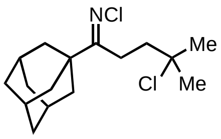

Following **GP12**, **6h** (33 mg, 0.1 mmol) gave **7h** (27 mg, 91%) as an oil. FT-IR ν_{max} (film)/cm^{-1} 2895, 2850, 1646, 1295, 969, 920, 670; ^{1}H NMR (500 MHz, $CDCl_3$) δ 2.78–2.74

(2H, m), 2.02–1.98 (2H, m), 1.87–1.85 (5H, m), 1.84–1.78 (2H, m), 1.72 (8H, m), 1.65 (6H, s); ^{13}C NMR (126 MHz, $CDCl_3$) δ 188.3, 70.4, 45.6, 40.4, 39.9, 39.5, 36.9, 36.4, 32.2, 28.4, 28.1, 28.0; HRMS (ESI) Found MH^+ 268.1817, $C_{16}H_{27}NCl$ requires 268.1827.

Ethyl-8-chloro-5-(chloroimino)-8-methylnonanoate (7i)

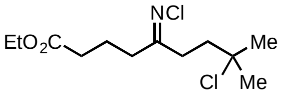

Following **GP12**, **6k** (32 mg, 0.1 mmol) gave **7i** (10 mg, 36%) as an oil. dr: 1.5:1. FT-IR ν_{max} (film)/cm^{-1} 2923, 2849, 1734, 1372, 1185, 730; ^{1}H NMR (400 MHz, $CDCl_3$) δ 4.14–4.01 (2H, m), 2.65–2.60 (1H, m), 2.59–2.53 (1H, m), 2.42 (1H, t, *J* = 7.2 Hz), 2.36–2.30 (2H, m), 2.25 (1H, t, *J* = 7.2 Hz), 2.01–1.95 (1H, m), 1.91–1.85 (1H, m), 1.82 (1H, t, *J* = 7.3 Hz), 1.53 (6H, s), 1.46–1.36 (1H, m), 1.16–1.10 (3H, m); δ ^{13}C NMR (126 MHz, $CDCl_3$) δ 182.7, 172.6, 69.8, 60.6, 41.1, 35.0, 33.7, 32.4, 22.3, 20.7, 14.3; HRMS (APCI) Found MH^+ 282.1013, $C_{12}H_{22}O_2NCl_2$ requires 282.1022.

10-Bromo-N,2-dichloro-2-methyldecan-5-imine (7j)

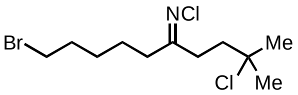

Following **GP12**, **6l** (35 mg, 0.1 mmol) gave **7j** (17 mg, 53%) as an oil. dr 1:1. FT-IR ν_{max} (film)/cm^{-1} 2925, 2854, 1711, 1461, 1371, 907, 731, 668; ^{1}H NMR (500 MHz, $CDCl_3$) δ 3.45–3.38 (2H, m), 2.79–2.75 (0.5H, m), 2.70–2.65 (1H, m), 2.62–2.57 (1H, m), 2.48 (0.5H, t, *J* = 7.6 Hz), 2.43 (0.5H, t, *J* = 7.3 Hz), 2.41–2.37 (0.5H, m), 2.06–2.01 (1H, m), 1.98–1.82 (3H, m), 1.70–1.57 (2H, m), 1.55 (6H, s), 1.53–1.39 (2H, m); ^{13}C NMR (126 MHz, $CDCl_3$) δ 183.2, 183.1, 69.9, 69.7, 42.4, 41.2, 41.0, 40.4, 38.6, 35.6, 34.6, 33.6, 33.5, 33.4, 32.7, 32.6, 32.5, 32.3, 32.2, 31.6, 28.1, 27.8, 27.7, 27.7, 25.0, 24.5, 22.9, 22.4; HRMS (APCI) Found MH^+ 316.0223, $C_{11}H_{21}NBrCl_2$ requires 316.0229.

***N*,4-Dichloro-4,8-dimethyl-1-phenylnonan-1-imine (7k)**

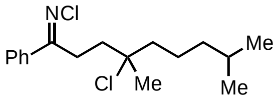

Following **GP12**, **6q** (35 mg, 0.1 mmol) gave **7k** (11 mg, 34%) as an oil. FT-IR ν_{max} (film)/cm^{-1} 2950, 2250, 903, 723, 650; ^{1}H NMR (500 MHz, $CDCl_3$) δ 7.62–7.32 (5H, m), 3.31–3.22 (2H, m), 2.09–1.94 (2H, m), 1.88–1.73 (2H, m), 1.61 (3H, s), 1.51–1.40 (2H, m),

1.24–1.16 (3H, m), 0.89 (6H, d, *J* = 6.6 Hz); ^{13}C NMR (126 MHz, $CDCl_3$) δ 132.9, 131.1, 129.6, 128.8, 128.4, 127.1, 73.9, 44.2, 39.2, 39.1, 39.0, 29.7, 29.5, 28.0, 24.7, 22.6; HRMS (APCI) Found MH^+ 314.1440, $C_{17}H_{26}NCl_2$ requires 314.1437.

5.5 Cascade 1,5-H Abstraction-Fluorination

General Procedure for the Reaction Optimization – GP13

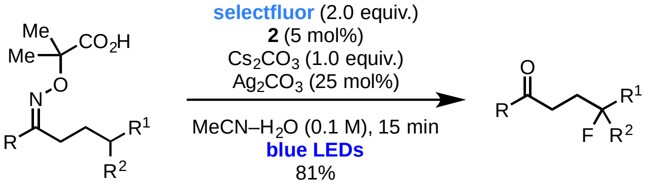

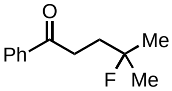

8a

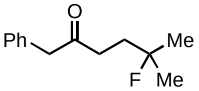

8b

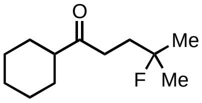

8c

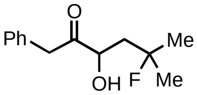

8d

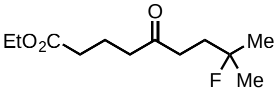

8e

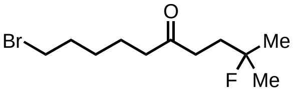

8f

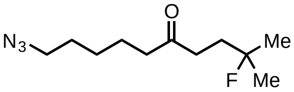

8g

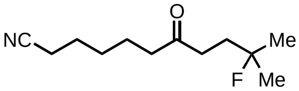

8h

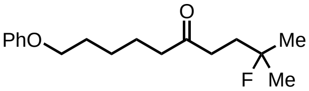

8i

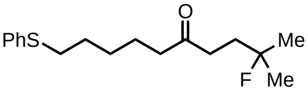

8j

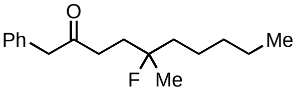

8k

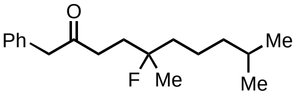

8l

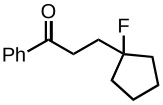

8m

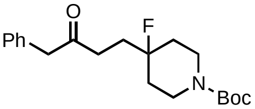

8n

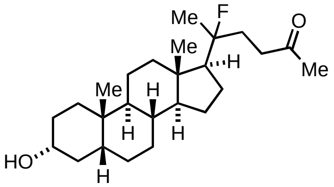

8o

A dry tube equipped with a stirring bar was charged with the oxime (1.0 equiv.), **2** (5 mol%), Cs_2CO_3 (0.1 mmol, 1.0 equiv.), Ag_2CO_3 (25 mol%) and selectfluor (0.2 mmol, 2.0 equiv.). The tube was sealed, evacuated and back-filled with nitrogen three times. $CH_3CN–H_2O$ (1:1, 0.1 M) was added, the blue LEDs were switched on and the reaction was stirred under irradiation for 15 min. The mixture was dilute with H_2O (1 mL) and EtOAc (1 mL). The layers were separated and the aqueous layer was extracted with EtOAc (3 x 5 mL). The combined organic layers were dried ($MgSO_4$), filtered and evaporated. The residue was purified by column chromatography on silica gel.

4-Fluoro-4-methyl-1-phenylpentan-1-one (8a)

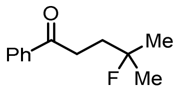

Following **GP13**, **6a** (28 mg, 0.1 mmol) gave **8a** (16 mg, 81%) as an oil. ^{1}H NMR (500 MHz, $CDCl_3$) δ 8.01–7.95 (2H, m), 7.64–7.29 (3H, m), 3.13 (2H, t, *J* = 7.7 Hz), 2.08 (2H, dt, *J* = 21.3, 7.7 Hz), 1.41 (6H, d, *J* = 21.3 Hz); ^{19}F NMR (376 MHz, $CDCl_3$) δ –140.9. Data in accordance with the literature.[18]

4-Fluoro-4-methyl-1-phenylpentan-1-one (8b)

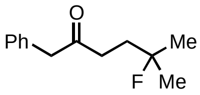

Following **GP13**, **6i** (29 mg, 0.1 mmol) gave **8b** (18 mg, 88%) as an oil. FT-IR ν_{max} (film)/cm^{-1} 2292, 2253, 1440, 1374, 1271, 1038, 918, 735, 703; ^{1}H NMR (400 MHz, $CDCl_3$) δ 7.35–7.31 (2H, m), 7.30–7.24 (1H, m), 7.21 (2H, d, *J* = 7.6 Hz), 3.72 (2H, s), 2.60 (2H, t, *J* = 7.7 Hz), 1.88 (2H, dt, *J* = 21.3, 7.7 Hz), 1.30 (6H, d, *J* = 21.3 Hz); ^{13}C NMR ($CDCl_3$, 101 MHz) δ 207.8, 134.3, 129.5, 128.9, 127.2, 95.0 (d, *J* = 165.8 Hz), 50.3, 36.6 (d, *J* = 3.7 Hz), 34.7 (d, *J* = 22.7 Hz), 26.7 (d, *J* = 24.7 Hz); ^{19}F NMR (376 MHz, $CDCl_3$) δ –140.8; HRMS (ESI) Found MNa$^+$ 231.1156, $C_{13}H_{17}FNaO$ requires 231.1161.

1-Cyclohexyl-4-fluoro-4-methylpentan-1-one (8c)

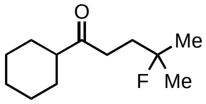

Following **GP13**, **6j** (28 mg, 0.1 mmol) gave **8c** (17 mg, 74%) as an oil. FT-IR ν_{max} (film)/cm^{-1} 1710, 1418, 1359, 1220, 1092, 533; ^{1}H NMR (500 MHz, $CDCl_3$) δ 2.60–2.55 (2H, m), 2.36–2.18 (1H, m), 1.93–1.64 (8H, m), 1.34 (6H, d, *J* = 21.1 Hz), 1.37–1.16 (4H, m); ^{13}C NMR (126 MHz, $CDCl_3$) δ 213.5, 95.2 (d, *J* = 165.8 Hz), 51.0, 34.9 (d, *J* = 3.6 Hz), 34.6 (d, *J* = 22.7 Hz), 28.6, 26.7 (d, *J* = 24.7 Hz), 25.8, 25.7; ^{19}F NMR (376 MHz, $CDCl_3$) δ –140.83; HRMS (ESI) Found MNa$^+$ 223.1469, $C_{12}H_{21}FNaO$ requires 223.1474.

5-Fluoro-3-hydroxy-5-methyl-1-phenylhexan-2-one (8d)

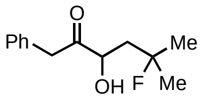

Following **GP13**, **6s** (32 mg, 0.1 mmol) gave **8d** (14 mg, 57%) as an oil. FT-IR ν_{max} (film)/cm^{-1} 1710, 1421, 1360, 1221, 1092, 903, 587; ^{1}H NMR (500 MHz, $CDCl_3$) δ 7.35–7.32 (2H, m), 7.31–7.26 (1H, m), 7.23–7.21 (2H, m), 4.52–4.39 (1H, br s, OH), 3.83 (1H, d, *J* = 15.7 Hz) , 3.76 (1H, d, *J* = 15.7 Hz), 3.44–3.33 (1H, m), 2.26–2.18 (1H, m), 1.84 (1H, m), 1.44 (3H, d, *J* = 20.9 Hz), 1.43 (3H, d, *J* = 22.0 Hz); ^{13}C NMR (126 MHz, $CDCl_3$) δ 209.6, 133.4, 129.7, 128.9, 127.4, 95.9 (d, *J* = 165.2 Hz), 73.6 (d, *J* = 3.9 Hz), 44.9 (d, *J* = 1.4 Hz), 44.5 (d, *J* = 21.9 Hz), 27.6 (d, *J* = 24.3 Hz), 27.2 (d, *J* = 24.4 Hz); ^{19}F NMR (376 MHz, $CDCl_3$) δ –135.1 HRMS (ESI) Found MNa^{+} 247.1105, $C_{13}H_{17}FNaO_2$ requires 247.1110.

Ethyl 8-fluoro-8-methyl-5-oxononanoate (8e)

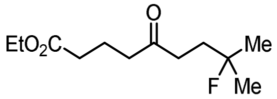

Following **GP13**, **6k** (33 mg, 0.1 mmol) gave **8e** (17 mg, 71%) as an oil. FT-IR ν_{max} (film)/cm^{-1} 1710, 1418, 1359, 1220, 1091, 1062, 531; ^{1}H NMR (400 MHz, $CDCl_3$) δ 4.13 (2H, q, *J* = 7.1 Hz), 2.60–2.47 (4H, m), 2.33 (2H, t, *J* = 7.2 Hz), 1.97–1.81 (4H, m), 1.34 (6H, d, *J* = 21.3 Hz), 1.25 (3H, t, *J* = 7.1 Hz); ^{13}C NMR (126 MHz, $CDCl_3$) δ 209.5, 173.3, 95.0 (d, *J* = 166.1 Hz), 60.5, 41.8, 37.3 (d, *J* = 3.7 Hz), 34.7 (d, *J* = 22.8 Hz), 33.5, 26.8 (d, *J* = 24.6 Hz), 19.1, 14.4; ^{19}F NMR (376 MHz, $CDCl_3$) δ –140.9; HRMS (ESI) Found MNa^{+} 255.1367, $C_{12}H_{21}FNaO$ requires 255.1372.

10-Bromo-2-fluoro-2-methyldecan-5-one (8f)

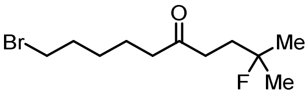

Following **GP13**, **6l** (37 mg, 0.1 mmol) gave **8f** (17 mg, 59%) as an oil. FT-IR ν_{max} (film)/cm^{-1} 3327, 2969, 2942, 2831, 1449, 1414, 1380, 1023, 951, 816, 669; ^{1}H NMR (500 MHz, $CDCl_3$) δ 3.34 (2H, t, *J* = 6.7 Hz), 2.51–2.45 (2H, m), 2.39 (2H, t, *J* = 7.4 Hz), 1.94–1.81 (2H, m), 1.54 (2H, dt, *J* = 21.3, 7.4 Hz), 1.42–1.33 (4H, m), 1.27 (6H, d, *J* = 21.3 Hz); ^{13}C NMR (126 MHz, $CDCl_3$) δ 210.1, 95.0 (d, *J* = 165.9 Hz), 42.7, 37.3 (d, *J* = 3.6 Hz), 34.8 (d, *J* = 22.8 Hz), 33.8, 32.7, 27.8, 26.8 (d, *J* = 24.7 Hz), 23.0; ^{19}F NMR (376 MHz, $CDCl_3$) –140.9; HRMS (ESI) Found MNa^{+} 289.0574, $C_{11}H_{20}BrFNaO$ requires 289.0579.

10-Azido-2-fluoro-2-methyldecan-5-one (8g)

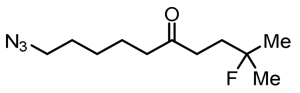

Following **GP13**, **6m** (31 mg, 0.1 mmol) gave **8g** (20 mg, 79%) as an oil. FT-IR ν_{max} (film)/cm^{-1} 3398, 1704, 1640, 1421, 1364, 1223, 669-533 (bs); ^{1}H NMR (400 MHz, $CDCl_3$) δ 3.27 (2H, t, *J* = 6.8 Hz), 2.54 (2H, t, *J* = 7.4 Hz), 2.46 (2H, t, *J* = 7.3 Hz), 1.90 (2H, dt, *J* = 21.2, 7.4 Hz), 1.65–1.57 (4H, m), 1.43–1.23 (2H, m), 1.34 (6H, d, *J* = 21.2 Hz); ^{13}C NMR (126 MHz, $CDCl_3$) δ 210.1, 95.0 (d, *J* = 166.1 Hz), 51.4, 42.7, 37.3 (d, *J* = 3.6 Hz), 34.8 (d, *J* = 22.8 Hz), 28.9, 26.9, 26.7 (d, *J* = 24.7 Hz), 23.4; ^{19}F NMR (376 MHz, $CDCl_3$) δ –140.9; HRMS (ESI) Found MNa$^+$ 252.1483, $C_{11}H_{20}FN_3NaO$ requires 252.1488.

10-Fluoro-10-methyl-7-oxoundecanenitrile (8h)

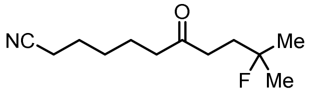

Following **GP13**, **6n** (30 mg, 0.1 mmol) gave **8h** (14 mg, 66%) as an oil. FT-IR ν_{max} (film)/cm^{-1} 3321, 2941, 2831, 1449, 1375, 1022, 917, 668; ^{1}H NMR (500 MHz, $CDCl_3$) δ 2.54 (2H, t, *J* = 7.6 Hz), 2.47 (2H, t, *J* = 7.2 Hz), 2.35 (2H, t, *J* = 7.1 Hz), 1.90 (2H, dt, *J* = 21.3, 7.5 Hz), 1.67 (2H, p, *J* = 7.7 Hz), 1.62 (2H, p, *J* = 7.7 Hz), 1.50–1.41 (2H, m), 1.34 (6H, d, *J* = 21.3 Hz); ^{13}C NMR (126 MHz, $CDCl_3$) δ 209.9, 119.8, 95.0 (d, *J* = 166.0 Hz), 42.4, 37.3 (d, *J* = 3.6 Hz), 34.7 (d, *J* = 22.8 Hz), 28.3, 26.8 (d, *J* = 24.7 Hz), 25.4, 22.9, 17.2; ^{19}F NMR (376 MHz, $CDCl_3$) δ –140.9; HRMS (ESI) Found MNa$^+$ 236.1429, $C_{12}H_{20}FNaNO$ requires 236.1427.

2-Fluoro-2-methyl-10-phenoxydecan-5-one (8i)

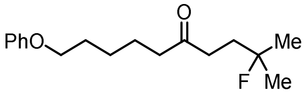

Following **GP13**, **6o** (38 mg, 0.1 mmol) gave **8i** (21 mg, 68%) as an oil. ^{1}H NMR (500 MHz, $CDCl_3$) δ 7.21 (2H, d, *J* = 7.3 Hz), 6.93 (1H, t, *J* = 7.2 Hz), 6.89 (2H, d, *J* = 7.8 Hz), 3.96 (2H, t, *J* = 6.3 Hz), 2.55 (2H, t, *J* = 7.7 Hz), 2.47 (2H, t, *J* = 7.3 Hz), 1.90 (2H, dt, *J* = 19.3, 7.3 Hz), 1.84–1.62 (4H, m), 1.52 – 1.41 (2H, m), 1.34 (6H, d, *J* = 21.3 Hz); ^{13}C NMR (126 MHz, $CDCl_3$) δ 210.1, 158.9, 129.3, 120.5, 114.4, 94.7 (d, *J* = 166.1 Hz), 67.4, 42.6, 37.0 (d, *J* = 3.7 Hz), 34.5 (d, *J* = 22.9 Hz), 29.0, 26.6 (d, *J* = 24.7 Hz), 25.6, 23.5; ^{19}F NMR (471 MHz, $CDCl_3$) δ –140.8.

2-Fluoro-2-methyl-10-(phenylthio)decan-5-one (8j)

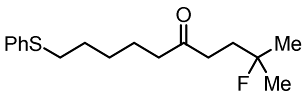

Following **GP13**, **6p** (38 mg, 0.1 mmol) gave **8j** (15 mg, 52%) as an oil. FT-IR ν_{max} (film)/cm^{-1} 1710, 1436, 1357, 12220, 531; ^{1}H NMR (500 MHz, $CDCl_3$) δ 7.61 (2H, d, *J* = 7.6 Hz), 7.54–7.47 (3H, m), 2.78 (2H, t, *J* = 7.5 Hz), 2.52 (2H, *J* = 7.5 Hz), 2.43 (2H, t, *J* = 7.2 Hz), 1.88 (2H, dt, *J* = 21.3, 7.5 Hz), 1.67–1.54 (4H, m), 1.51–1.31 (2H, m), 1.33 (6H, d, *J* = 21.3 Hz); ^{13}C NMR (126 MHz , $CDCl_3$) δ 210.3, 144.3, 131.3, 129.6, 124.4, 95.2 (d, *J* = 166.0 Hz), 57.3, 42.7, 37.6 (d, *J* = 3.6 Hz), 35.0 (d, *J* = 22.9 Hz), 28.5, 27.1 (d, *J* = 24.7 Hz), 23.6, 22.3**;** ^{19}F NMR (471 MHz, $CDCl_3$) δ –140.9; HRMS (ESI) Found MNa$^+$ 319.1502, $C_{17}H_{25}FNaSO$ requires 319.1508.

5-Fluoro-5-methyl-1-phenyldecan-2-one (8k)

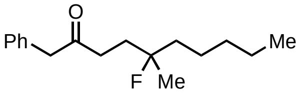

Following **GP13**, **6u** (35 mg, 0.1 mmol) gave **8k** (17 mg, 63%) as an oil. FT-IR ν_{max} (film)/cm^{-1} 2253, 1440, 1374, 1032, 918, 737; ^{1}H NMR (500 MHz, $CDCl_3$) δ 7.33 (1H, t, *J* = 7.2 Hz), 7.29–7.25 (1H, m), 7.21 (2H, d, *J* = 6.9 Hz), 3.64 (2H, s), 2.50 (2H, t, *J* = 7.7 Hz), 1.94–1.64 (2H, m), 1.61–1.37 (2H, m), 1.33–1.22 (6H, m), 1.16 (3H, d, *J* = 21.7 Hz), 0.87–0.76 (3H, m); ^{13}C NMR (126 MHz , $CDCl_3$) δ 207.6, 134.1, 129.3, 128.8, 128.6, 126.9, 96.6 (d, *J* = 168.0 Hz), 95.7 (d, *J* = 188.1 Hz), 50.1, 39.4 (d, *J* = 22.6 Hz), 36.1 (H, d, *J* = 4.0 Hz), 32.9, 32.8, 25.7 (H, d, *J* = 6.0 Hz), 23.9 (d, *J* = 24.9 Hz), 22.90, 13.85; ^{19}F NMR (376 MHz , $CDCl_3$) δ –146.0; HRMS (ESI) Found MNa$^+$ 287.3748, $C_{17}H_{25}FNaO$ requires 287.3742.

5-Fluoro-5,9-dimethyl-1-phenyldecan-2-one (8l)

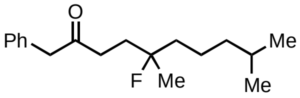

Following **GP13**, **6r** (37 mg, 0.1 mmol) gave **8l** (16 mg, 59%) as an oil. FT-IR ν_{max} (film)/cm^{-1} 2253, 1443, 1375, 1029, 918, 737, 586; ^{1}H NMR (500 MHz, $CDCl_3$) δ 7.35–7.31 (2H, m), 7.30–7.24 (1H, m), 7.21 (2H, d, *J* = 7.2 Hz), 3.72 (2H, s), 2.58 (2H, t, *J* = 7.8 Hz), 1.99–1.72 (2H, m), 1.52–1.42 (1H, m), 1.35–1.27 (2H, m), 1.24 (3H, d, *J* = 21.7 Hz), 1.19–1.09 (2H, m), 0.86 (6H, d, *J* = 6.6 Hz), 0.90–0.82 (2H, m); ^{13}C NMR (126 MHz, $CDCl_3$) δ 207.9, 134.3, 129.5, 129.0 (d, *J* = 19.3 Hz), 127.2, 96.9 (d, *J* = 168.0 Hz), 50.3, 40.2 (d, *J* = 22.6 Hz), 39.4, 36.3 (d, *J* = 4.0 Hz), 33.1 (d, *J* = 22.9 Hz), 28.0, 24.1 (d, *J* = 24.9 Hz), 22.7 (d,

J = 4.6 Hz), 21.6 (d, J = 6.1 Hz); ^{19}F NMR (376 MHz, $CDCl_3$) δ –146.5; HRMS (ESI) Found MNa^+ 301.1952, $C_{18}H_{27}FNaO$ requires 301.1944.

4-(1-Fluorocyclopentyl)-1-phenylbutan-2-one (8m)

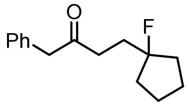

Following **GP13**, **6v** (41 mg, 0.13 mmol) gave **8m** (25 mg, 82%) as an oil. FT-IR ν_{max} (film)/cm^{-1} 2943, 2293, 2252, 1712, 1443, 1374, 1223, 1038, 917, 736; ^{1}H NMR (500 MHz, $CDCl_3$) δ 7.35–7.31 (2H, m), 7.30–7.24 (1H, m), 7.21 (2H, d, J = 7.6 Hz), 3.72 (2H, s), 2.64 (2H, t, J = 7.5 Hz), 1.98 (2H, dt, J = 22.5, 7.5 Hz), 1.92–1.73 (4H, m), 1.65–1.44 (4H, m); ^{13}C NMR (126 MHz, $CDCl_3$) δ 208.3, 134.6, 129.8, 129.1, 127.5, 106.7 (d, J = 172.9 Hz), 50.9, 37.9 (d, J = 23.7 Hz), 37.6 (d, J = 2.8 Hz), 32.6 (d, J = 24.1 Hz), 24.2; ^{19}F NMR (376 MHz, $CDCl_3$) δ –144.5; HRMS (ESI) Found MNa^+ 257.1312, $C_{15}H_{19}FNaO$ requires 257.1318.

***tert*-Butyl 4-fluoro-4-(3-oxo-4-phenylbutyl)piperidine-1-carboxylate (8n)**

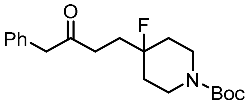

Following **GP13**, **6t** (22 mg, 0.05 mmol) gave **8n** (15 mg, 82%) as an oil. dr: 3:1. FT-IR ν_{max} (film)/cm^{-1} 2970, 1708, 1418, 1362, 1221, 1160, 1129, 1093, 951, 816; ^{1}H NMR (500 MHz, $CDCl_3$, diastereomers and rotamers) δ 7.46–7.26 (3.5H, m), 7.20 (1.5H, d, J = 7.5 Hz), 4.17–3.77 (2.5H, m), 3.71 & 3.69 (1.5H, s), 3.02 (1.5H, br t, J = 12.1 Hz), 2.78–2.66 (0.5H, m), 2.60 (1.5H, t, J = 7.6 Hz), 2.46 (0.5H, t, J = 7.5 Hz), 1.87 (1.5H, dt, J = 21.4, 7.7 Hz), 1.76–1.64 (1.5H, m), 1.64–1.58 (0.5H, m), 1.57–1.46 (2.5H, m), 1.45 & 1.44 (9H, s); ^{13}C NMR (126 MHz, $CDCl_3$, diastereomers and rotamers) δ 208.3m, 207.4^{M}, 154.8^{m}, 154.7^{M}, 134.2^{m}, 134.0^{M}, 129.4, 128.8, 128.8, 127.2^{M}, 127.1^{m}, 95.9 (d, J = 188.1 Hz)^{m}, 93.3 (d, J = 172.1 Hz)^{M}, 79.6^{M}, 79.2^{m}, 50.3^{m}, 50.3^{M}, 44.8^{m} (br s), 39.6^{M} (br s), 38.8, 35.3, 35.1 (d, J = 3.5 Hz), 34.6 (br s) 33.5 (d, J = 22.2 Hz), 30.1, 28.5^{m}, 28.4^{M}; ^{19}F NMR (376 MHz ,$CDCl_3$) δ –164.54^{M}, –164.81^{m}; HRMS (ESI) Found MNa^+ 372.1945, $C_{20}H_{28}FNNaO_3$ requires 372.1951.

5-Fluoro-5-((5*R*,8*R*,9*S*,10*S*,13*S*,14*S*,17*S*)-3-hydroxy-10,13-dimethylhexadecahydro-1H-cyclopenta[a]phenanthren-17-yl)hexan-2-one (8o)

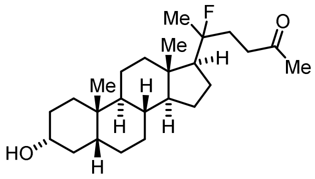

Following **GP13**, **6w** (32 mg, 0.07 mmol) gave **8o** (19 mg, 71%) as an oil. dr: 1.3:1. FT-IR ν_{max} (film)/cm^{-1} 3313, 2942, 2831, 1448, 1418, 1381, 1113, 1023, 951; ^{1}H NMR (500 MHz, $CDCl_3$, diastereomers) δ 3.67–3.56 (1H, m), 2.59–2.52 (1H, m), 2.52–2.45 (1H, m), 2.17 (1.3H, s), 2.16 (1.7H, s), 2.09–1.96 (2H, m), 1.92–1.69 (4H, m), 1.69–1.44 (7H, m), 1.45–1.34 (4H, m), 1.35 (3H, d, *J* = 21.7 Hz), 1.31–1.23 (4H, m), 1.21–0.93 (4H, m), 0.92 (3H, s), 0.78 (3H, d, *J* = 3.1 Hz); ^{13}C NMR (126 MHz, $CDCl_3$, diastereomers) δ 208.3, 208.2, 98.9 (d, *J* = 172.9 Hz)^{M}, 98.3 (d, *J* = 173.5 Hz)^{m}, 71.8, 57.8 (d, *J* = 20.1 Hz), 56.7, 43.0, 42.8, 42.0, 40.4 (d, *J* = 4.0 Hz), 40.3, 38.2 (d, *J* = 4.9 Hz)^{m}, 38.0 (d, *J* = 6.0 Hz)^{M}, 36.4, 35.3 (d, *J* = 4.2 Hz), 34.6, 34.0 (d, *J* = 23.9 Hz)^{M}, 33.1 (d, *J* = 24.3 Hz)^{m}, 30.5 (d, *J* = 1.2 Hz), 30.0 (d, *J* = 6.2 Hz), 27.1, 26.3 (d, *J* = 3.0 Hz), 24.1 (d, *J* = 25.4 Hz)^{m}, 23.7, 23.6, 23.3, 23.2 (d, *J* = 5.5 Hz)^{m}, 23.2 (d, *J* = 25.4 Hz)^{M}, 22.6 (d, *J* = 4.0 Hz)^{M}, 20.6, 20.6, 13.2 (H, d, *J* = 4.0 Hz), 13.0 (d, *J* = 4.1 Hz); ^{19}F NMR (376 MHz, $CDCl_3$) δ –154.3^{M}, –154.5^{m}; HRMS (ESI) Found MNa^{+} 415.2983, $C_{25}H_{41}FNNaO_2$ requires 415.2988.

6 Mechanistic Considerations

6.1 Emission Quenching Experiments

Emission intensities were recorded using a Steady State emission spectra were recorded on an Edinburgh Instrument FP920 Phosphorescence Lifetime Spectrometer equipped with a 5 watt microsecond pulsed xenon flash lamp and a 450 watt steady state xenon lamp and a red sensitive photomultiplier in peltier (air cooled) housing, (Hamamatsu R928P) spectrophotometer. The **2** solutions were excited at 436 nm and the emission intensity was collected at 505 nm.

Experimental procedures:

A screw-top quartz cuvette was charged with a 1.6 x 10^{-5} M solution of **2** in CH_2Cl_2 (2.0 mL) and the initial emission was collected then the appropriate amount of the quencher as a 1.6 x 10^{-2} M solution in CH_2Cl_2 was added. The sample was shaken for 1 min and then the emission of the sample was collected.

Quencher	Stern-Volmer Plot
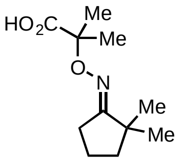 **1a**	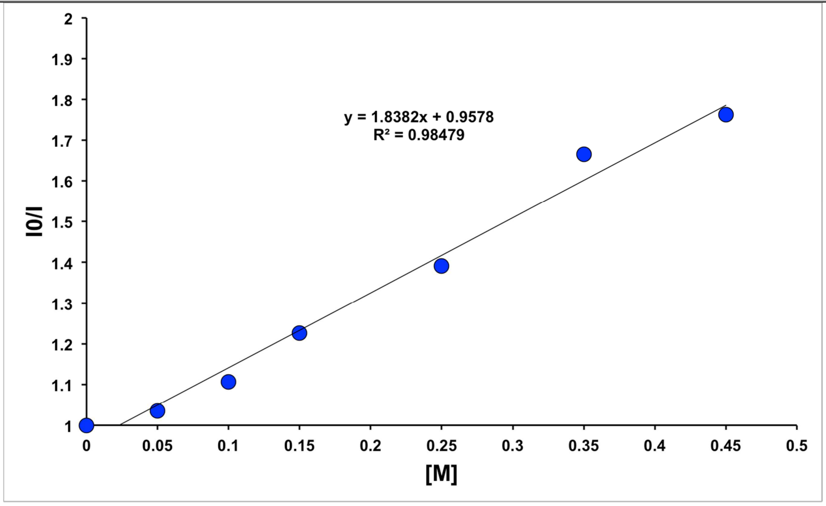

Quencher	Stern-Volmer Plot
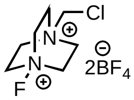 [e]	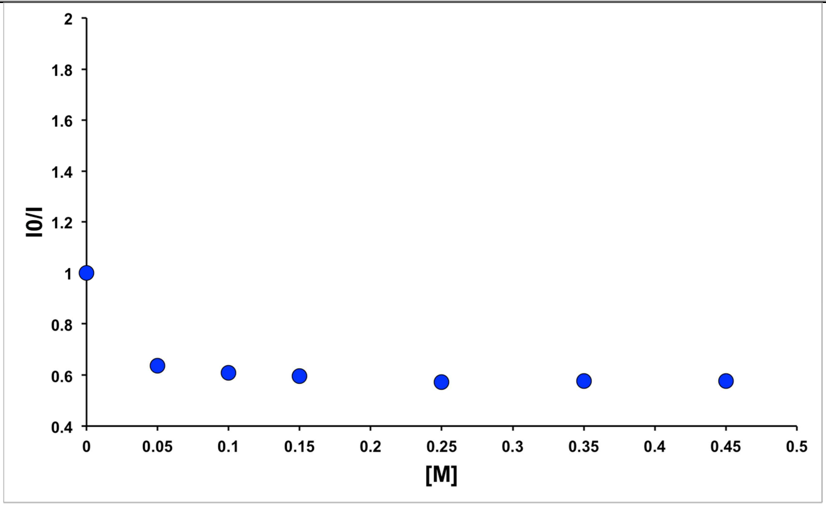
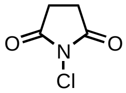	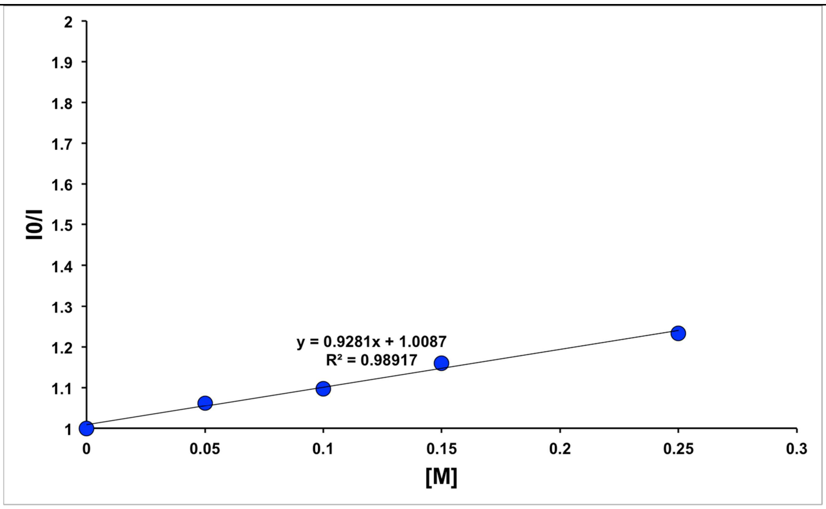
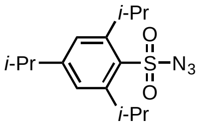	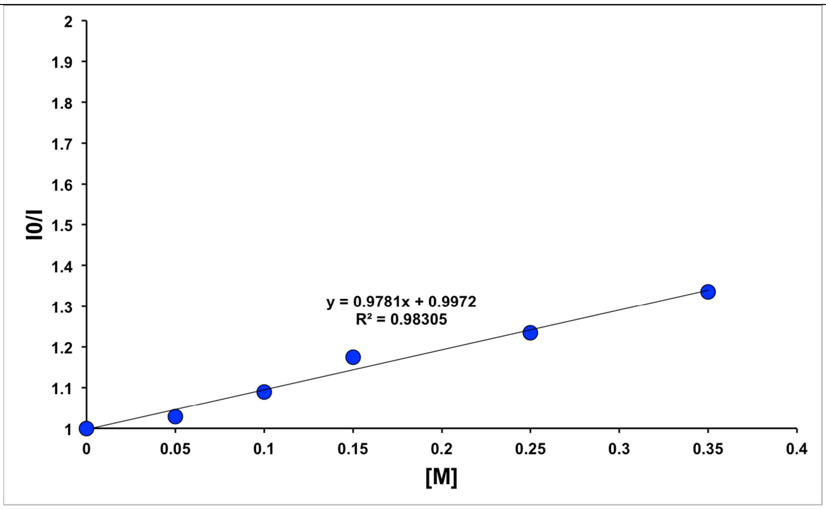

[e] In this case CH_3CN was used as the solvent.

Quencher	Stern-Volmer Plot
Ag_2CO_3[e]	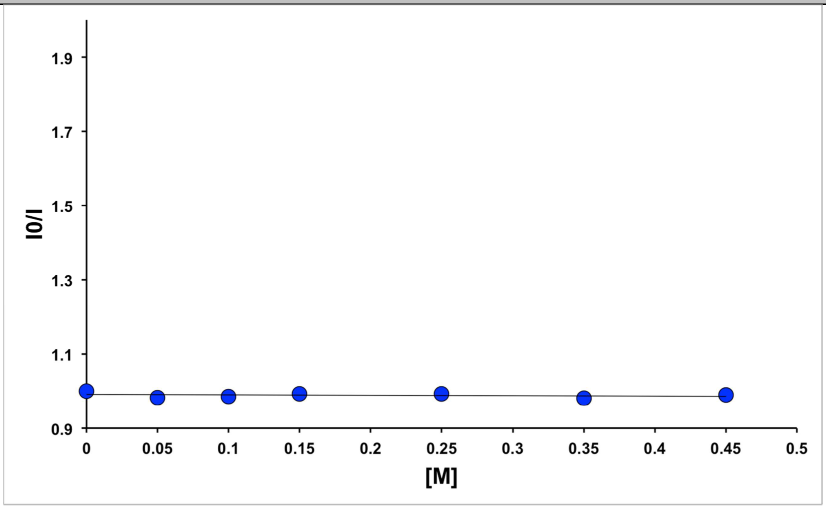

The quenching constants were obtained using the Stern-Volmer relationship:

$$\frac{I_o}{I} = 1 + k_{q\tau_0}[Quencher]$$

Substrate	k_q (M^{-1} s^{-1})
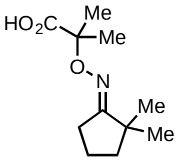 **1a**	3.1 x 10^8
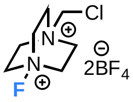	–
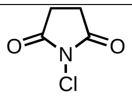	1.5 x 10^8
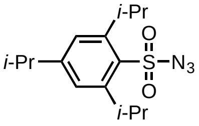	1.5 x 10^8
Ag_2CO_3	–

6.2 Quantum Yield Determination

The quantum yield determination was performed following the procedure reported by Yoon[19] and are the average of two runs.

Reaction	Quantum Yield (Φ)
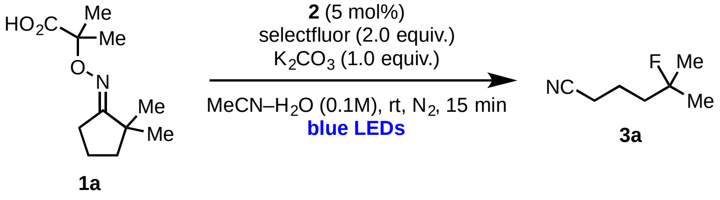	2.8
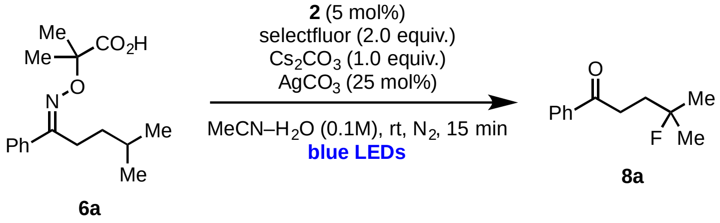	4.8

6.3 Ring-Opening: DFT Reactivity Scales

The following radical ring-openings were not found experimentally successful:

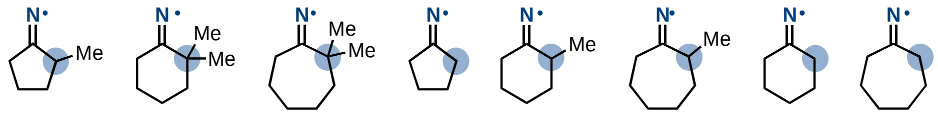

We have performed DFT studies aimed at determine the reaction parameters and reported them graphically in the following scales.

• **ΔG‡ (kcal mol^{-1})**

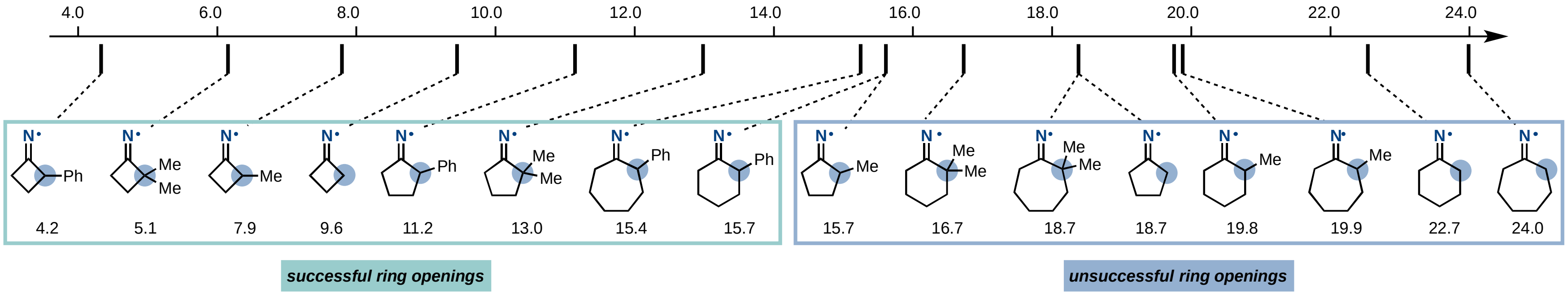

• **ΔG° (kcal mol^{-1})**

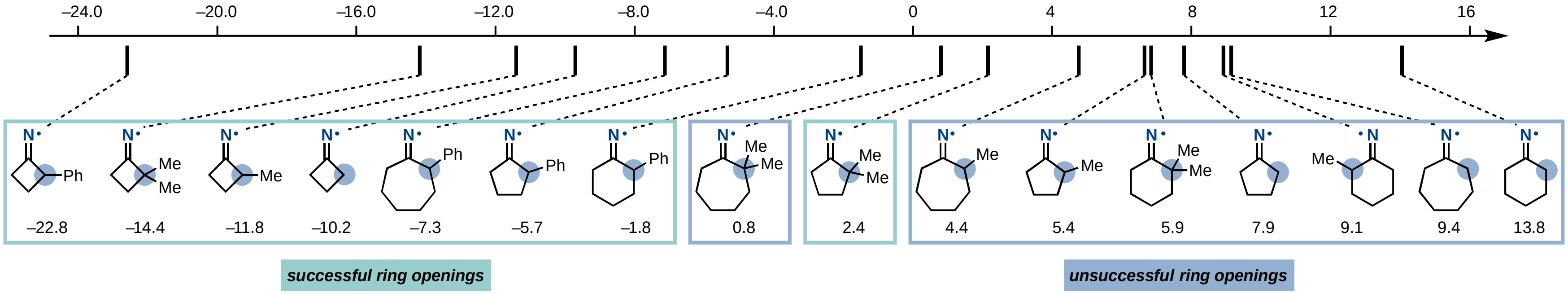

6.4 1,5-H Abstraction: DFT Reactivity Scales

The following 1,5-H abstraction were not found experimentally successful:

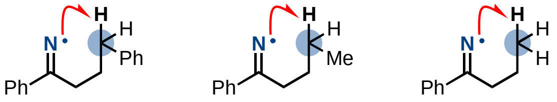

We have performed DFT studies aimed at determine the reaction parameters and reported them graphically in the following scales.

• ΔG‡ (kcal mol^{-1})

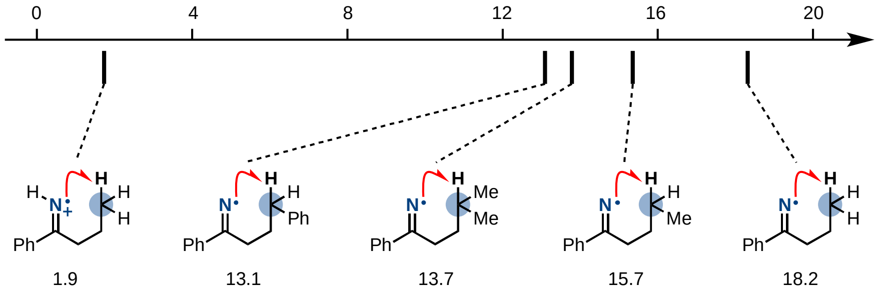

• ΔG° (kcal mol^{-1})

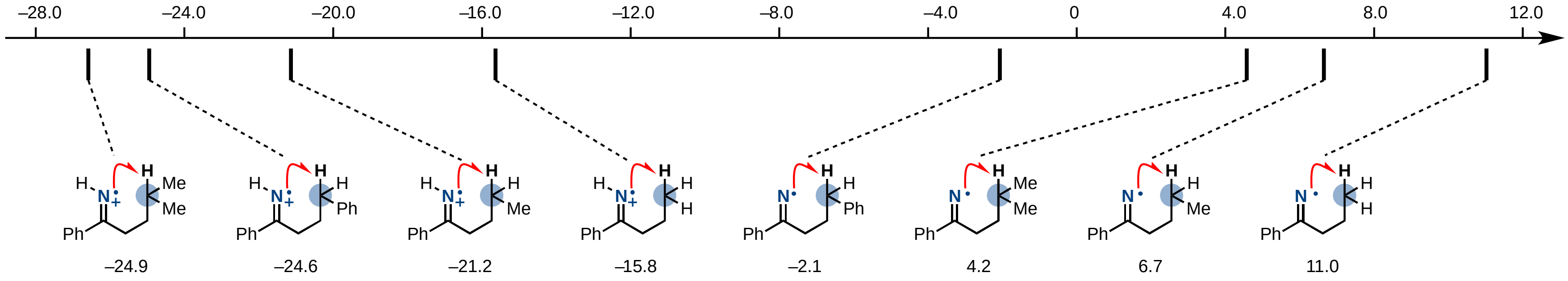

6.5 Role of Ag(I) in the 1,5-H Abstraction Fluorination

We have performed preliminary mechanistic studies to understand the role of Ag(I) in the 1,5-H abstraction fluorination cascade. We propose that the Ag(I) species acts at as dual co-catalyst facilitating both the radical fluorination and the final SET reduction.

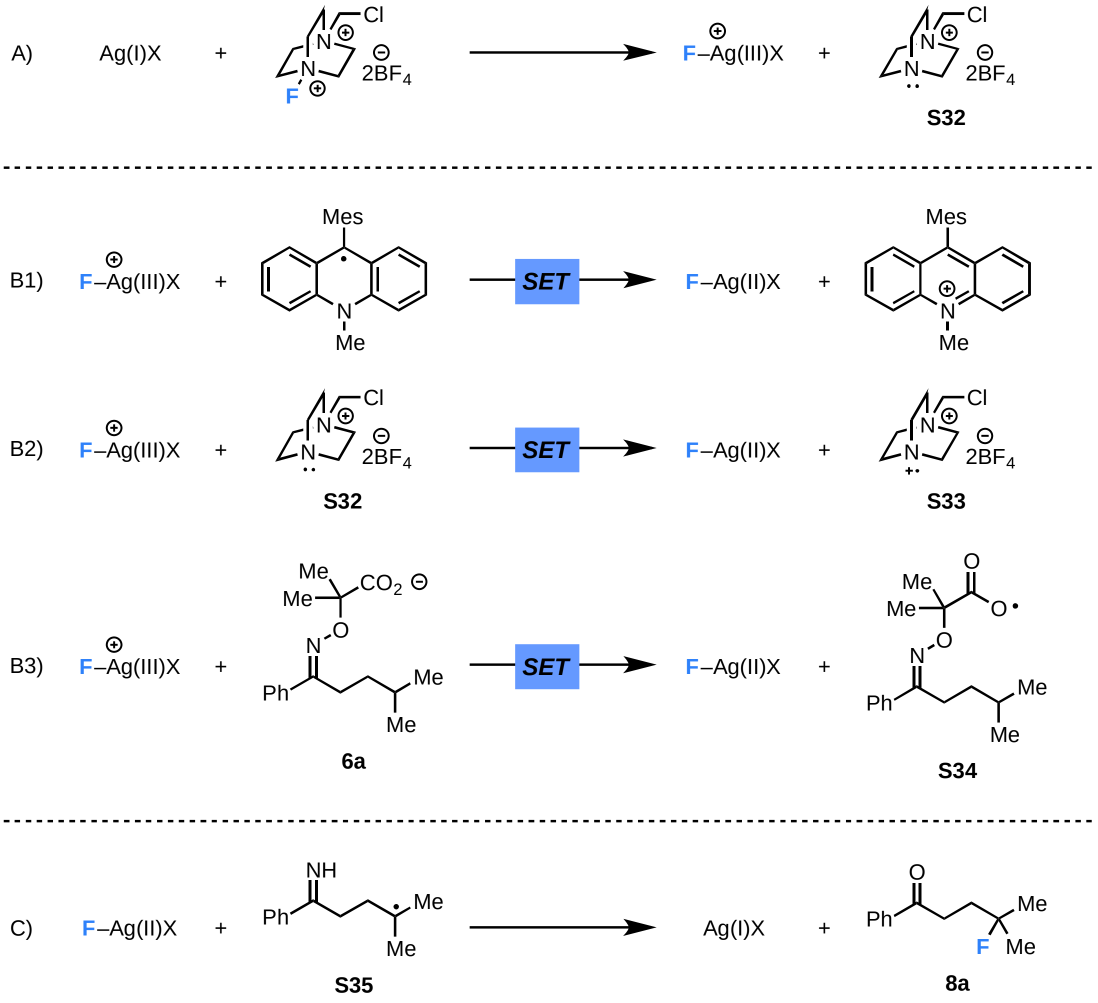

A) Selectfluor is a strong oxidant (E^{red} = +0.25 V vs SCE)[20] that can provide to formation of Ag(III)-F species.[21]

B) Ag(III)-F species are known to be strong oxidants[21a] and we propose that they can close the photoredox cycle by direct SET with the reduced photoredox catalyst (**B1**) and can also sustain productive radical chain propagations by oxidation of the DABCO species **S32** (B2) and/or the deprotonated oxime **6a** (B3).

C) This SET would deliver a Ag(II)-F species which is a very powerful radical F-transfer agent.[21a, 21c, 21d] In this way, following 1,5-H abstraction, the C-radical **S35** would undergo F-transfer to give the product and regenerate the catalytically active Ag(I) species.

At this stage, the presence of multinuclear Ag-complexes[22] cannot be excluded.

In order to provide some evidence for this reactivity scenario we have run some control experiments with several Ag(I) sources.

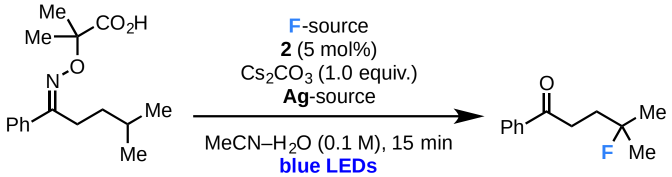

6a **8a**

Entry	Ag-source (equiv.)	F-source (equiv.)	Yield (%)
**1**	Ag_2CO_3 (0.25)	selectfluor (2.0)	81
**2**	AgF_2 (0.25)	selectfluor (2.0)	35
**3**	AgF_2 (0.5)	selectfluor (2.0)	47
**4**	AgF_2 (2.0)	–	15
**5**	AgF (0.25)	selectfluor (2.0)	45
**6**	AgF (2.0)	–	7

The successful formation of **8a** in the presence of AgF_2, with and without selectfluor, supports our proposed mechanistic picture.

The unsuccessful reaction outcome when using NFSI can be result by the fact that NFSI being a weaker oxidant than selectfluor,[21d] does not enable the efficient generation of the Ag(III)-F species to sustain the photoredox cycle and/or the productive radical chain pathways operating under our reaction conditions.

We have also evaluated the possibility of the 1,5-abstraction and fluorination to take place following oxidative fragmentation from the oxime. We feel this is not the case because when ketone **S10** was exposed to identical reaction conditions, **8a** could not be detected and **S10** was quantitatively recovered.

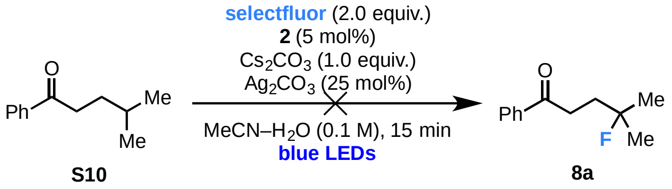

selectfluor (2.0 equiv.)
2 (5 mol%)
Cs2CO3 (1.0 equiv.)
Ag2CO3 (25 mol%)
MeCN–H2O (0.1 M), 15 min
blue LEDs
Ph
Me
Me
S10
F
8a

7 Computational Studies

7.1 Computational Methods

Density functional theory (DFT)[23] calculations were performed using Gaussian 09 (revision E.01)[24] and the Gaussview[25] was used to generate input geometries and visualize output structures. Geometry optimizations and frequency calculations for the ring-opening and 1,5-H atom abstraction reactions, B3LYP functional[26] was used with the UB3LYP/6-31+G(d,p) basis set.[27] All stationary points were characterized as minima or transitions states based on normal vibrational mode analysis. Thermal corrections were computed from unscaled frequencies, assuming a standard state of 298.15 K and 1 atm. Representative transition states were also linked to their corresponding minima through the intrinsic reaction coordinate (IRC)[28] calculations, which confirm the connection of transition structures with the reactants and products. For substrates having more than one conformations, low energy conformation of the transition state could possibly be different from the low energy ground state.[29] The structures described herein are the lowest energy-optimized conformers. Homolytic bond dissociation enthalpies (BDE) were calculated using (RO)B3P86/6-311G(d,p) for the determination of geometries, frequencies (scaled by a factor of 0.9806) and molecular energies.[30]

7.2 Activation Energy (ΔG‡) and Reaction Energy (ΔGº) for Ring-opening Reactions

DFT Method: UB3LYP/6-31+G(d,p) [values are in Kcal mol^{-1}]

No.	Ring-opening Reactions	ΔG≠	ΔG
1	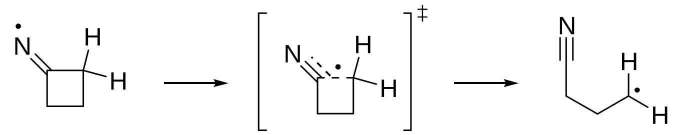	9.6	-10.2
2	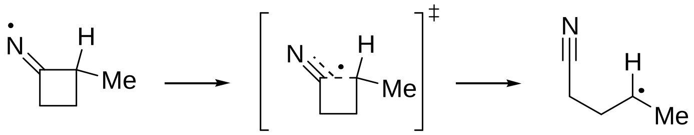	7.9	-11.8
3	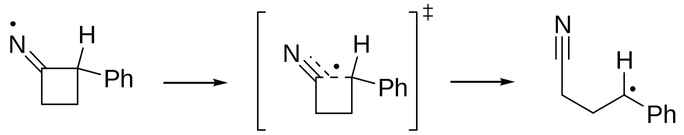	4.2	-22.8
4	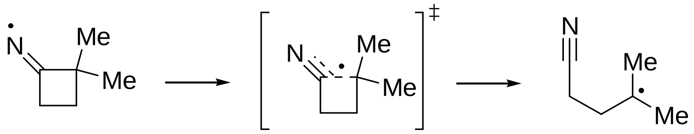	5.1	-14.4
5	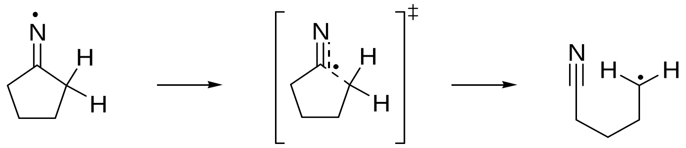	18.7	7.9
6	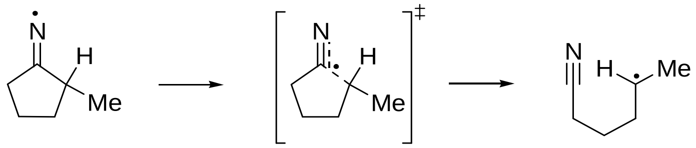	15.7	5.4
7	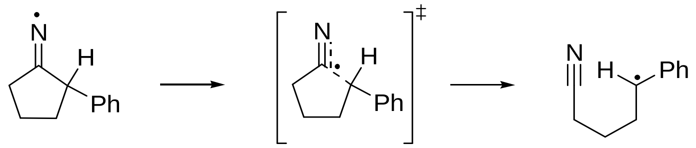	11.2	-5.7
8	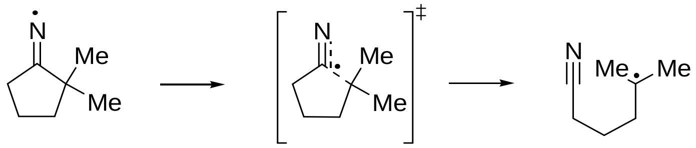	13.0	2.4
9	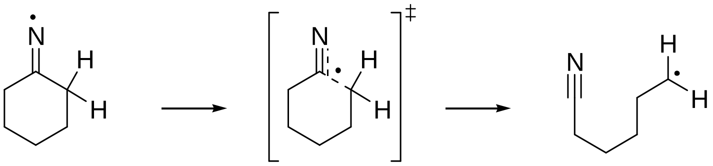	22.7	13.8

No.	Ring-opening Reactions	ΔG$^{\neq}$	ΔG
10	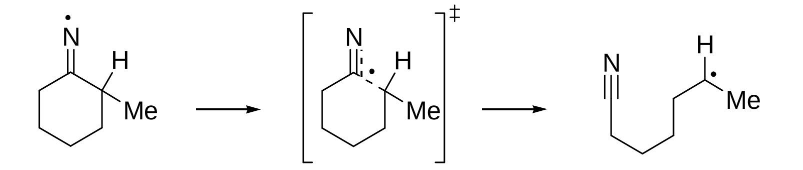	19.8	9.1
11	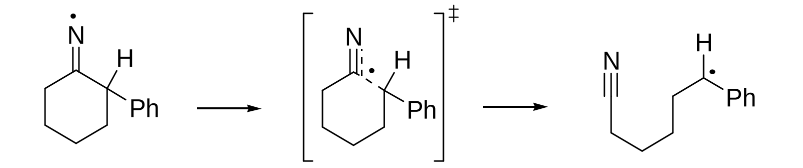	15.7	-1.8
12	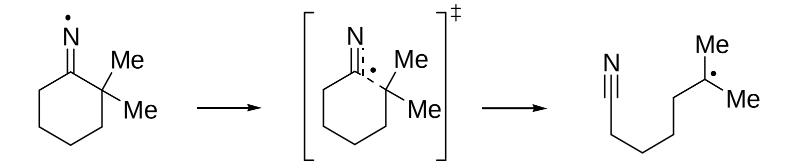	16.7	5.9
13	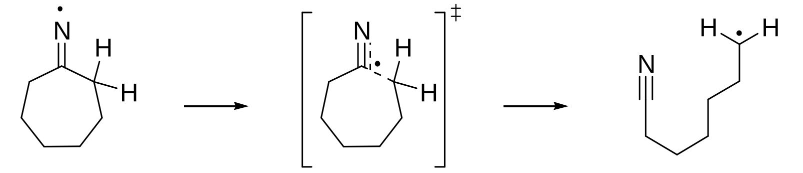	24.0	9.4
14	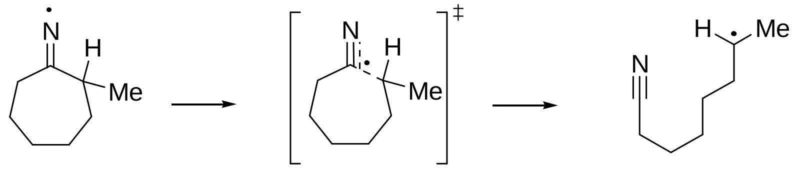	19.9	4.4
15	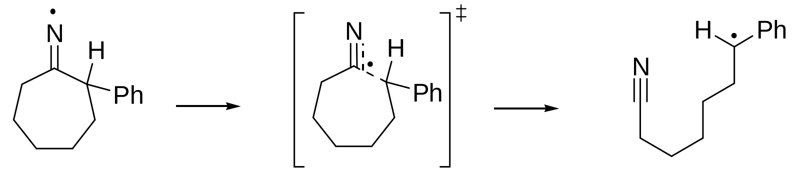	15.4	-7.3
16	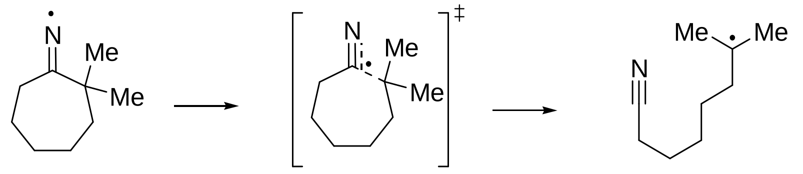	18.7	0.8

Computed Energies [values are in Hartree]

No.	Species	Total Electronic Energy	Sum of Electronic and Zero-point Energies	Gibbs Free Energy

No.	Species	Total Electronic Energy	Sum of Electronic and Zero-point Energies	Gibbs Free Energy
1	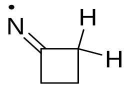	-210.7166215	-210.626489	-210.654033
2	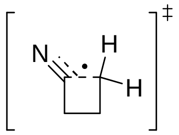	-210.6979534	-210.609980	-210.638747
3	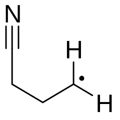	-210.7281878	-210.640406	-210.670367
4	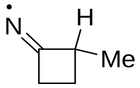	-250.0375918	-249.919134	-249.949627
5	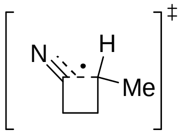	-250.0224139	-249.906168	-249.937034
6	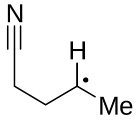	-250.051458	-249.935230	-249.968477
7	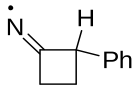	-441.7826685	-441.611084	-441.648001
8	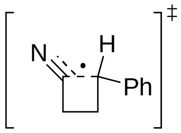	-441.7747906	-441.604834	-441.641311
9	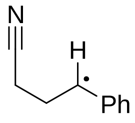	-441.8157272	-441.645108	-441.684279
10	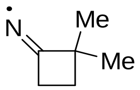	-289.3572314	-289.211182	-289.242115
11	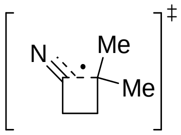	-289.345167	-289.201106	-289.234027

No.	Species	Total Electronic Energy	Sum of Electronic and Zero-point Energies	Gibbs Free Energy
12	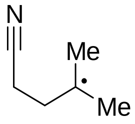	-289.3743336	-289.229876	-289.265095
13	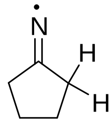	-250.0654743	-249.944731	-249.974536
14	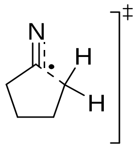	-250.0319367	-249.914602	-249.944672
15	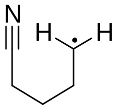	-250.0460994	-249.929775	-249.961967
16	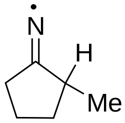	-289.3829979	-289.234136	-289.265863
17	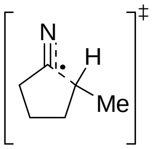	-289.3543375	-289.208676	-289.240877
18	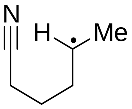	-289.3672346	-289.222321	-289.257209
19	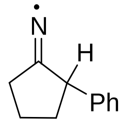	-481.1264788	-480.924730	-480.963168
20	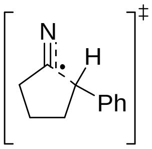	-481.1077488	-480.908192	-480.945302
21	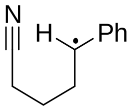	-481.1317662	-480.932389	-480.972301

No.	Species	Total Electronic Energy	Sum of Electronic and Zero-point Energies	Gibbs Free Energy
22	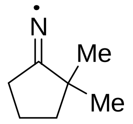	-328.701492	-328.524961	-328.558012
23	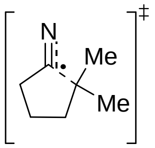	-328.6773023	-328.503613	-328.537241
24	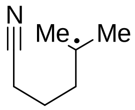	-328.6897892	-328.516962	-328.554184
25	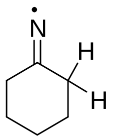	-289.3888477	-289.238559	-289.269385
26	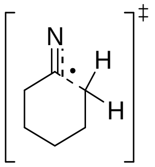	-289.347792	-289.201617	-289.233174
27	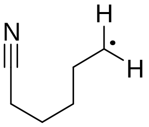	-289.358242	-289.213391	-289.247457
28	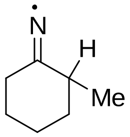	-328.7047815	-328.526316	-328.558864
29	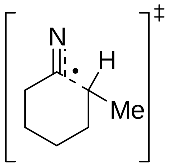	-328.6685384	-328.493878	-328.527386
30	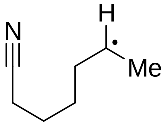	-328.6807265	-328.507405	-328.544424
31	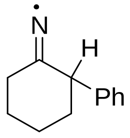	520.4502628	-520.219108	-520.257187

No.	Species	Total Electronic Energy	Sum of Electronic and Zero-point Energies	Gibbs Free Energy
32	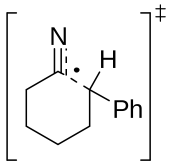	-520.4223635	-520.193732	-520.232150
33	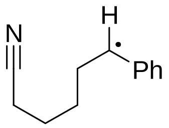	-520.4466126	-520.218597	-520.260097
34	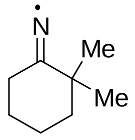	-368.0218584	-367.815756	-367.849542
35	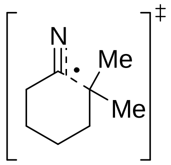	-367.9906659	-367.788018	-367.822917
36	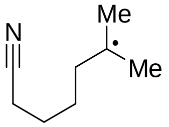	-368.0028887	-367.801238	-367.840073
37	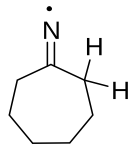	-328.6984517	-328.519696	-328.552846
38	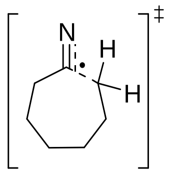	-328.656164	-328.481218	-328.514526
39	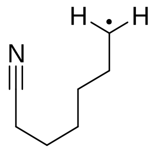	-328.6747067	-328.501421	-328.537823
40	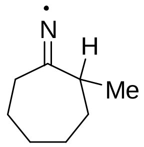	-368.0146288	-367.807675	-367.842371

No.	Species	Total Electronic Energy	Sum of Electronic and Zero-point Energies	Gibbs Free Energy
41	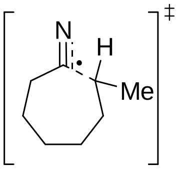	-367.9787141	-367.775556	-367.810646
42	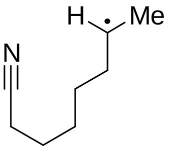	-367.9982529	-367.796579	-367.835389
43	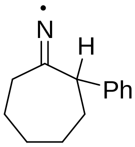	-559.7577878	-559.498148	-559.539089
44	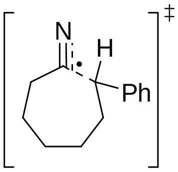	-559.7313942	-559.474511	-559.514609
45	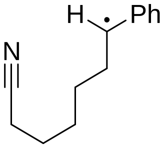	-559.7630951	-559.507007	-559.550721
46	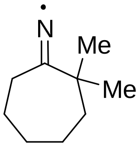	-407.3317484	-407.096827	-407.132171
47	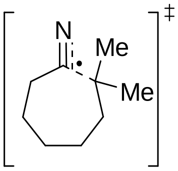	-407.2976989	-407.066051	-407.102373
48	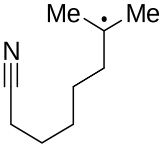	-407.3192837	-407.089364	-407.130858

Optimized Structures and Cartesian Coordinates

No.	Species	Optimized Structure
1	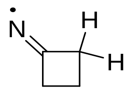	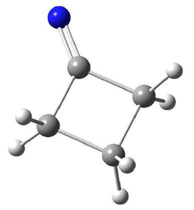

Cartesian Coordinates

C	-0.34827600	1.10851000	0.00015700
C	-1.44316200	-0.00000100	0.00012700
C	-0.34827200	-1.10851000	0.00005800
C	0.73483500	0.00000100	-0.00008700
H	-0.30905700	1.74209700	-0.89043200
H	-0.30887900	1.74181100	0.89094400
H	-2.08063400	0.00003700	-0.88695300
H	-2.08065300	-0.00004100	0.88719200
H	-0.30905200	-1.74201000	-0.89059300
H	-0.30887700	-1.74190000	0.89078200
N	1.97520000	0.00000100	-0.00035200

No.	Species	Optimized Structure
2	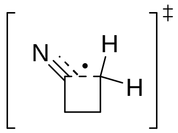	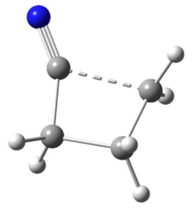

Cartesian Coordinates

C	0.88398000	-0.11220800	0.02056500
N	2.02656600	0.19840200	-0.09173600
C	-0.25301800	-1.10038400	0.10062300
H	-0.13631800	-1.91893300	-0.61746600
H	-0.27613200	-1.52580200	1.11009300
C	-1.41853100	-0.11599500	-0.15491300
H	-1.76692800	-0.17285000	-1.18977600
H	-2.27815900	-0.26631600	0.50509300
C	-0.64310700	1.16667600	0.10075200
H	-0.60923800	1.55624100	1.11579900
H	-0.53513300	1.91031700	-0.68375100

No.	Species	Optimized Structure
3	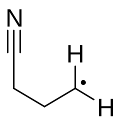	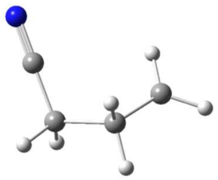

<table>
<tr><th>No.</th><th>Species</th><th>Optimized Structure</th></tr>
<tr><td colspan="3">Cartesian Coordinates
C 1.25982700 0.05564000 -0.04482200
N 2.16199600 -0.64965800 0.15041400
C 0.11157900 0.93691200 -0.28001900
H 0.35312200 1.92509900 0.12773500
H -0.01462100 1.05257900 -1.36252300
C -1.19934700 0.40714900 0.35509500
H -1.04879600 0.35506700 1.44740800
H -1.97645600 1.16411700 0.19579600
C -1.64336400 -0.91354200 -0.17577500
H -2.69722400 -1.16727700 -0.20822300
H -0.92216200 -1.69892700 -0.37996400</td></tr>
<tr><td>4</td><td>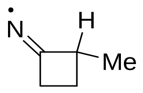</td><td>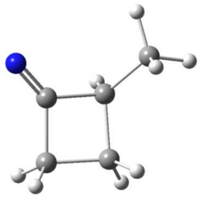</td></tr>
<tr><td colspan="3">Cartesian Coordinates
C -1.44383900 0.65950500 -0.11555500
C -0.09920300 1.44114400 -0.08342000
C 0.64047100 0.17279500 0.44249600
C -0.63976700 -0.64522600 0.09400300
H -2.09276400 0.86133100 0.74259100
H -2.03638700 0.69575600 -1.03276700
H -0.06407600 2.32420800 0.55898700
H 0.23839100 1.71929400 -1.08641500
H 0.73356400 0.20323900 1.53576200
N -0.87466200 -1.85138600 -0.06992700
C 1.95422700 -0.25468000 -0.19626000
H 2.74447200 0.46828300 0.03723800
H 2.26856700 -1.23610200 0.17192400
H 1.85952800 -0.31753400 -1.28541100</td></tr>
<tr><td>5</td><td>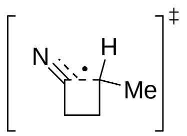</td><td>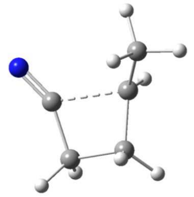</td></tr>
<tr><td colspan="3">Cartesian Coordinates
C 0.98709100 0.56152100 0.03839800
N 1.27609100 1.71373300 -0.05091500
C 1.27324100 -0.91464700 -0.08715900</td></tr>
</table>

No.	Species	Optimized Structure
H	1.88379400 -1.14857400 -0.96551800	
H	1.81148600 -1.24703100 0.80752800	
C	-0.19312300 -1.40340900 -0.10858300	
H	-0.53706900 -1.57070900 -1.13433500	
H	-0.37469900 -2.31660400 0.46780200	
C	-0.81454300 -0.13975200 0.47414100	
H	-0.79835500 -0.07986800 1.56372400	
C	-1.94710300 0.57687600 -0.19621400	
H	-1.80312100 0.63031600 -1.28048200	
H	-2.90023100 0.05594400 -0.01257800	
H	-2.04781700 1.59686600 0.18677400	
6	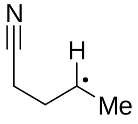	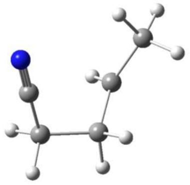
Cartesian Coordinates		
C	-1.57570100 -0.28660300 0.05132600	
N	-2.02488400 -1.35473900 -0.03335700	
C	-0.99389400 1.05577800 0.13870100	
H	-1.63009100 1.74265100 -0.43275100	
H	-1.02828000 1.37794400 1.18563600	
C	0.47275500 1.12170200 -0.39072300	
H	0.47396000 0.82450200 -1.44600600	
H	0.75658800 2.18303300 -0.35508100	
C	1.44651600 0.29448600 0.38632100	
H	1.68168900 0.61038000 1.40107100	
C	1.93825400 -1.03105500 -0.09255300	
H	2.82315600 -1.35774500 0.46259200	
H	1.17500400 -1.81957600 0.02001900	
H	2.19457500 -1.00386300 -1.16041500	
7	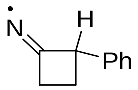	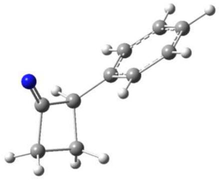
Cartesian Coordinates		
C	3.07951800 -0.12077500 -0.48137600	
C	1.88876400 -0.91600800 -1.08624300	
C	1.04155500 -0.65307300 0.20490800	
C	2.10835900 0.42549300 0.58868100	

No.	Species	Optimized Structure

H	3.84628900	-0.75536800	-0.02556000
H	3.56053700	0.63720900	-1.10392500
H	2.06739300	-1.96444200	-1.33580200
H	1.45714600	-0.41088700	-1.95416200
H	1.16329400	-1.48882100	0.90430400
N	2.12073400	1.40063000	1.35394200
C	-0.41398000	-0.28354700	0.09942100
C	-1.39682900	-1.16041900	0.58012500
C	-0.82278500	0.91514600	-0.50587200
C	-2.75494900	-0.85642000	0.45149700
H	-1.09712600	-2.08862500	1.06074900
C	-2.17771500	1.22267100	-0.63364200
H	-0.07755000	1.62116900	-0.86136200
C	-3.14942700	0.33648400	-0.15757200
H	-3.50085200	-1.54863000	0.83159100
H	-2.47556700	2.15825200	-1.09831100
H	-4.20376700	0.57841700	-0.25468000

No.	Species	Optimized Structure
8	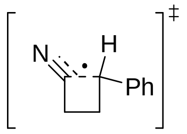	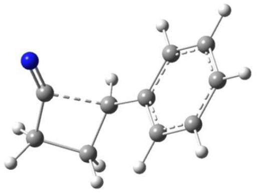

Cartesian Coordinates

C	-2.19396000	0.71196500	0.51760500
N	-2.03720300	1.65788700	1.23916800
C	-3.07401000	-0.43746000	0.06661600
H	-3.53127000	-0.97453600	0.90325400
H	-3.87179600	-0.05207200	-0.57762800
C	-1.96452300	-1.17886200	-0.71363500
H	-1.57018300	-2.01905100	-0.13850300
H	-2.25610500	-1.55127300	-1.70022900
C	-0.99548500	0.00543800	-0.75389000
C	0.42229800	-0.05743100	-0.38449100
C	1.28001900	0.99154700	-0.77978900
C	0.96956400	-1.10278800	0.38761900
C	2.62733900	0.99191500	-0.42853500
H	0.87499500	1.81280500	-1.36518000
C	2.31946100	-1.10360600	0.73597300
H	0.33968600	-1.92086100	0.72192000
C	3.15535900	-0.05811400	0.33083500
H	3.26709900	1.81015400	-0.74618900
H	2.72016900	-1.92117100	1.32838000

No.	Species	Optimized Structure
H	4.20591400 -0.06025200 0.60581900	
H	-1.19446800 0.69541300 -1.57566600	
9	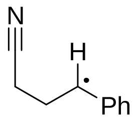	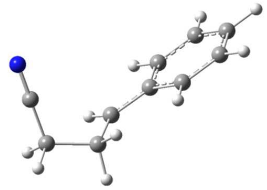

Cartesian Coordinates

C	3.21013300	0.02037500	0.80568500
N	3.30254200	0.10539600	1.96049500
C	3.09590600	-0.06722700	-0.65381000
H	3.86622100	0.57625500	-1.09477800
H	3.32867400	-1.09574000	-0.95486400
C	1.69683400	0.33922500	-1.18370900
H	1.48448700	1.36642800	-0.87428100
H	1.77637200	0.35845400	-2.28299700
C	0.61313400	-0.60285900	-0.76348800
H	0.85083200	-1.66419200	-0.82383400
C	-0.69974000	-0.26195000	-0.35807300
C	-1.62343400	-1.30217200	-0.03109000
C	-1.17517500	1.07985700	-0.25439800
C	-2.91999800	-1.02009400	0.37095900
H	-1.29034200	-2.33444800	-0.09875600
C	-2.47637400	1.35060300	0.14914500
H	-0.51504100	1.90764000	-0.49060500
C	-3.36088000	0.30929000	0.46528900
H	-3.59640600	-1.83422200	0.61523800
H	-2.80921200	2.38223800	0.22109500
H	-4.37581000	0.52952700	0.78126300

No.	Species	Optimized Structure
10	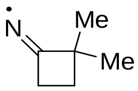	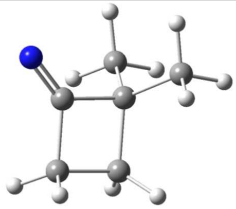

Cartesian Coordinates

C	-1.57352100	0.78346400	-0.00464800
C	-0.18249800	1.47831500	-0.02640600
C	0.54847300	0.09317100	-0.00202700
C	-0.86863900	-0.59137400	0.00428300
H	-2.17880700	0.95477300	0.88997000
H	-2.19853000	0.93476000	-0.88919200

No.	Species	Optimized Structure

H	0.02283100	2.10421600	0.84628700
H	0.01079900	2.06438500	-0.92901900
N	-1.24563500	-1.77060200	0.00956100
C	1.35805800	-0.23257100	-1.25892500
H	2.25414900	0.39893600	-1.29994000
H	1.67363400	-1.28075600	-1.25784000
H	0.77656600	-0.05597600	-2.16964300
C	1.33913500	-0.18946300	1.27792100
H	2.23497500	0.44293000	1.31062600
H	0.74420400	0.01888300	2.17315900
H	1.65357200	-1.23718800	1.31747400

No.	Species	Optimized Structure
11	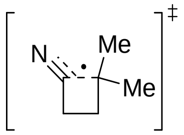	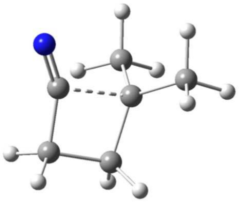

Cartesian Coordinates

C	-1.16623900	-0.52631000	0.05267800
N	-1.51315000	-1.66578400	0.11081300
C	-1.44623900	0.95430300	-0.04669300
H	-2.18811700	1.17704100	-0.82060000
H	-1.83507000	1.31530100	0.91169900
C	-0.00134200	1.42529200	-0.33812800
H	0.12770100	1.71235600	-1.38608200
H	0.32618000	2.26226000	0.28759800
C	0.70142400	0.09986000	-0.02130900
C	1.44535600	-0.60194700	-1.12649000
H	0.90326800	-0.55894500	-2.07609000
H	2.42932700	-0.13025100	-1.28177400
H	1.61538800	-1.65416200	-0.87788100
C	1.28068900	-0.05161300	1.36358000
H	1.44015800	-1.10549700	1.61160100
H	2.25575600	0.45866300	1.42166000
H	0.63557900	0.38621200	2.13234900

No.	Species	Optimized Structure
12	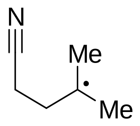	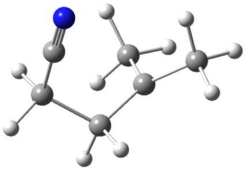

Cartesian Coordinates

C	-1.88934600	-0.05595400	0.24374600

No.	Species	Optimized Structure
N	-2.50265700 -1.00265100 0.52239800	
C	-1.10614000 1.12695500 -0.12279700	
H	-1.75151100 1.79721500 -0.70338500	
H	-0.83606000 1.65828300 0.79681000	
C	0.17993500 0.78702800 -0.94346900	
H	-0.12691500 0.29026000 -1.87055500	
H	0.61518000 1.75717100 -1.22417600	
C	1.19643200 -0.04131100 -0.21475200	
C	1.24947800 -1.52532200 -0.41057100	
H	0.58813300 -2.06103400 0.29231800	
H	0.93434000 -1.81559300 -1.41905400	
H	2.26236800 -1.91275800 -0.24278300	
C	1.93823700 0.56471600 0.93790500	
H	2.94205800 0.13282300 1.03775600	
H	2.04673200 1.65097600 0.83209300	
H	1.43269800 0.38453900 1.90381800	
13	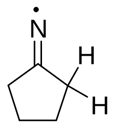	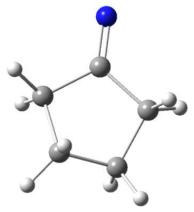
Cartesian Coordinates		
C	1.33616000 -0.73204200 0.23931900	
C	1.33619500 0.73203500 -0.23919200	
C	-0.06933300 1.24624100 0.12832000	
C	-0.96921300 -0.00000300 -0.00004600	
C	-0.06932400 -1.24623300 -0.12837600	
H	2.13254800 -1.33095800 -0.21307700	
H	1.47381600 -0.76853200 1.32719600	
H	1.47399300 0.76852200 -1.32705100	
H	2.13252900 1.33094300 0.21330600	
H	-0.10453500 1.58175500 1.17219900	
H	-0.43085200 2.06680400 -0.49598400	
H	-0.43091100 -2.06684000 0.49583100	
H	-0.10439900 -1.58167900 -1.17228200	
N	-2.21844300 0.00000000 -0.00004100	
14	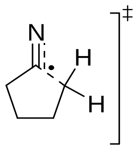	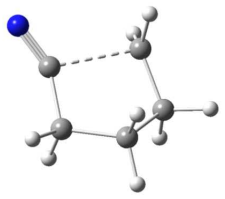

No.	Species	Optimized Structure

Cartesian Coordinates

C	-1.50503500	0.53307300	0.20469000
C	-1.15626400	-0.87203800	-0.32690600
C	0.22996800	-1.24285200	0.21528800
C	1.19371000	-0.11618600	0.03246800
C	-0.34467300	1.44845800	-0.05480400
H	-2.42072100	0.89983300	-0.28220100
H	-1.71376700	0.47818700	1.28000700
H	-1.12665100	-0.85442100	-1.42318200
H	-1.90111400	-1.61796300	-0.03048300
H	0.17367100	-1.45082200	1.29158700
H	0.64787400	-2.13097400	-0.26930200
H	-0.00577400	2.13789900	0.71145300
H	-0.15653600	1.76579100	-1.07753200
N	2.28525400	0.32424900	-0.08925300

No.	Species	Optimized Structure
15	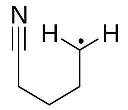	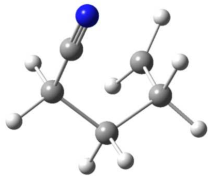

Cartesian Coordinates

C	1.66413400	-0.13227700	-0.15038700
N	2.46579600	-0.89669500	0.19998800
C	0.64243300	0.83196900	-0.57280600
H	1.13516300	1.79753700	-0.73416200
H	0.24550400	0.50412300	-1.54083500
C	-0.50581600	0.98052700	0.45028000
H	-0.09049500	1.30198100	1.41169700
H	-1.15818800	1.78873900	0.09737500
C	-1.33637500	-0.30280900	0.64720300
H	-2.03883800	-0.11257000	1.47865000
H	-0.68640600	-1.11557700	0.99419600
C	-2.09506500	-0.72941600	-0.56560400
H	-2.62947100	0.00268500	-1.16612200
H	-2.25371100	-1.77801700	-0.79283600

No.	Species	Optimized Structure
16	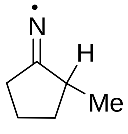	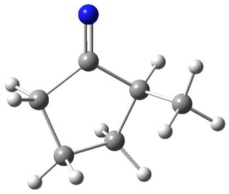

Cartesian Coordinates

No.	Species	Optimized Structure

C	-0.53294800	-1.28638400	-0.58702900
C	-1.52506700	-0.65903500	0.41112300
C	-1.32357000	0.85632500	0.22942000
C	0.16634800	0.99405500	-0.13837800
C	0.73574100	-0.40362800	-0.50292100
H	-0.31603500	-2.33800900	-0.37167300
H	-0.95186700	-1.23915100	-1.59991500
H	-1.27037600	-0.95242800	1.43640400
H	-2.55984500	-0.96739800	0.23268900
H	-1.91436000	1.23141900	-0.61569200
H	-1.57805300	1.45943300	1.10431200
H	1.24596300	-0.33586500	-1.46911800
N	0.81959300	2.05819000	-0.14078000
C	1.74602900	-0.87839900	0.55541200
H	2.58317500	-0.17922900	0.63275300
H	1.28478800	-0.95961300	1.54590900
H	2.14026100	-1.86408900	0.28402300

No.	Species	Optimized Structure
17	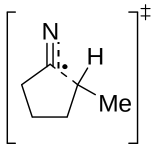	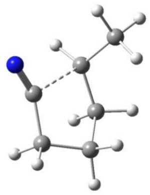

Cartesian Coordinates

C	-0.44715500	-1.41489800	0.47116900
C	0.77037100	-1.39740200	-0.47716600
C	1.64520800	-0.20319900	-0.07911800
C	0.80608700	1.02283300	0.09147800
C	-1.03939300	-0.02935700	0.55529900
H	-1.19204400	-2.14057100	0.11268500
H	-0.12446800	-1.74801400	1.46501800
H	0.43626700	-1.27617700	-1.51440100
H	1.33777600	-2.33245300	-0.42550800
H	2.14079400	-0.39759800	0.88098600
H	2.42627600	0.01334900	-0.81478200
H	-1.19736000	0.38056500	1.55074700
N	0.67284400	2.20061600	0.08185200
C	-2.01643500	0.43057500	-0.48750500
H	-2.10692000	1.52064500	-0.48810200
H	-1.72609700	0.11282200	-1.49527400
H	-3.01623100	0.01180800	-0.28927900

No.	Species	Optimized Structure
18	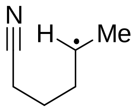	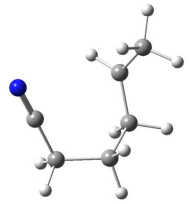

Cartesian Coordinates

C	1.65352100	0.62898100	0.05874500
N	1.78065100	1.78404600	0.04944600
C	1.53635100	-0.83350900	0.03657300
H	2.37777300	-1.21377600	-0.55473800
H	1.67569800	-1.20528500	1.05949700
C	0.20733600	-1.36100200	-0.54792800
H	0.02927000	-0.89258900	-1.52239600
H	0.33784400	-2.43445100	-0.73149500
C	-1.02785400	-1.16529900	0.37112400
H	-0.83653800	-1.68354200	1.32003800
H	-1.86237200	-1.69767400	-0.11224300
C	-1.42775600	0.25263000	0.63398300
H	-1.14810100	0.71263200	1.57802700
C	-2.02396300	1.11899600	-0.42604200
H	-2.71090400	1.85976200	0.00007800
H	-1.25593600	1.69196700	-0.97284200
H	-2.57709100	0.52984900	-1.16878900

No.	Species	Optimized Structure
19	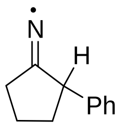	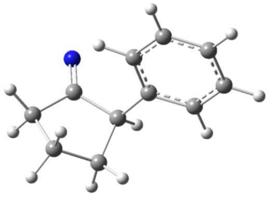

Cartesian Coordinates

C	1.47749400	1.56315600	0.23520600
C	2.16132400	0.71410700	1.32030400
C	2.77510000	-0.46194100	0.53835500
C	1.81986000	-0.66114400	-0.65596900
C	0.82480500	0.52782300	-0.72728000
H	0.74369600	2.26791400	0.63608700
H	2.23121600	2.14119400	-0.31436600
H	1.41530700	0.34624700	2.03399300
H	2.91066500	1.27463900	1.88751200
H	3.76363200	-0.20085800	0.14165000
H	2.88561600	-1.38312200	1.11647400
N	1.86110300	-1.61270000	-1.46317300

No.	Species	Optimized Structure

H	0.80773300	0.90641500	-1.75387400
C	-0.60389600	0.16827400	-0.33379900
C	-1.64309000	1.02380400	-0.73467400
C	-0.92965200	-0.95345400	0.44021200
C	-2.96550800	0.77415900	-0.36502500
H	-1.41400900	1.89200700	-1.34857800
C	-2.25330400	-1.20560500	0.81435700
H	-0.15447600	-1.65319400	0.73619800
C	-3.27598200	-0.34295700	0.41616600
H	-3.75277800	1.44693500	-0.69341800
H	-2.48278900	-2.08390300	1.41108700
H	-4.30443500	-0.54270500	0.70232700

No.	Species	Optimized Structure
20	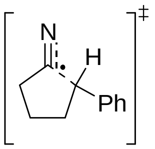	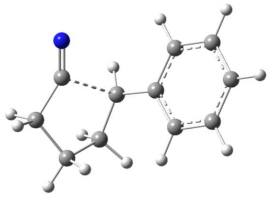

Cartesian Coordinates

C	1.66313600	-1.11445000	1.03978900
C	2.40854300	-1.26911500	-0.30145700
C	2.89890800	0.12708600	-0.69317000
C	1.82773200	1.14343900	-0.40485300
C	0.72966300	0.07968600	0.97049400
H	1.11906700	-2.03207900	1.29476100
H	2.39655500	-0.94763500	1.83734900
H	1.72752200	-1.64770700	-1.07155600
H	3.24105900	-1.97595800	-0.22476700
H	3.77615700	0.40862200	-0.09640200
H	3.18055300	0.20466100	-1.74753100
N	1.44332300	2.25112200	-0.62776200
C	-0.64042600	-0.03860700	0.47013600
C	-1.53765100	1.03624000	0.66122100
C	-1.11742900	-1.17976600	-0.21152900
C	-2.84787700	0.97271300	0.19727900
H	-1.18554200	1.93133800	1.16603100
C	-2.43214600	-1.24395800	-0.67159100
H	-0.46232400	-2.02957000	-0.37506800
C	-3.30447000	-0.16937700	-0.47185700
H	-3.51614600	1.81436200	0.35520500
H	-2.77748700	-2.13587900	-1.18689200
H	-4.32739200	-0.22045200	-0.83296800
H	0.83682100	0.80909500	1.76940900

No.	Species	Optimized Structure
21	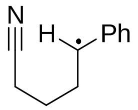	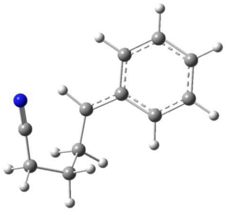

Cartesian Coordinates

C	2.92395600	1.13090300	-0.45395700
N	2.62368400	2.24973100	-0.54254000
C	3.34800500	-0.27092600	-0.35954600
H	4.01740800	-0.46281500	-1.20650100
H	3.95259500	-0.37976900	0.54979500
C	2.20442700	-1.30903800	-0.36118800
H	1.58798400	-1.17109200	-1.25577700
H	2.67833500	-2.29466300	-0.44520200
C	1.30695700	-1.30141700	0.90424300
H	1.95762100	-1.34177800	1.78839400
H	0.73637700	-2.23822500	0.90545900
C	0.37919100	-0.12945500	1.02345200
H	0.74738100	0.75555100	1.53522200
C	-0.93013200	-0.05817000	0.48364400
C	-1.69606900	1.13595300	0.64732700
C	-1.54803600	-1.12998200	-0.22799700
C	-2.98222900	1.24620800	0.14023500
H	-1.24801700	1.97214400	1.17730800
C	-2.83760800	-1.00901600	-0.73131200
H	-1.00724600	-2.05862700	-0.38057500
C	-3.56737900	0.17512000	-0.55351200
H	-3.53784700	2.16927800	0.27999100
H	-3.28260300	-1.84215200	-1.26859900
H	-4.57427700	0.26295200	-0.95007400

No.	Species	Optimized Structure
22	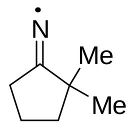	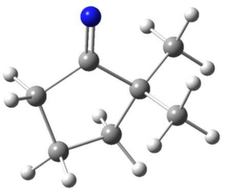

Cartesian Coordinates

C	-0.36822600	-1.34993300	-0.46971400
C	-1.72843200	-0.89559100	0.08735700
C	-1.72053300	0.62944100	-0.12259300
C	-0.23661200	1.02238100	0.00508800
C	0.65037800	-0.26442800	-0.03136500

No.	Species	Optimized Structure

H -0.07234300 -2.34657700 -0.12426100
H -0.41395900 -1.38474500 -1.56612600
H -1.80462400 -1.13080900 1.15521700
H -2.57364500 -1.37970400 -0.41158800
H -2.04789300 0.88563800 -1.13792400
H -2.34552100 1.19069200 0.57631100
N 0.19508600 2.18681900 0.12610800
C 1.17537500 -0.53519700 1.39518700
H 0.36097400 -0.64699700 2.11833500
H 1.76585300 -1.45899700 1.40518400
H 1.81153700 0.28773800 1.73345400
C 1.82511100 -0.11817700 -1.00827000
H 2.50354700 0.67892400 -0.69004200
H 2.39349000 -1.05455500 -1.05777300
H 1.47462000 0.12068900 -2.01767700

No.	Species	Optimized Structure
23	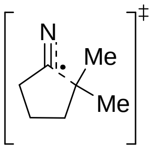	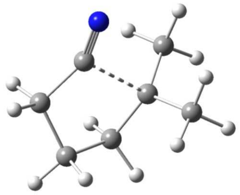

Cartesian Coordinates
C -0.07624700 -1.46859000 -0.35620600
C -1.48166400 -1.17568700 0.20172900
C -1.85465200 0.24658600 -0.23109700
C -0.70529000 1.17803600 0.00008800
C 0.85214300 -0.31386900 -0.03955800
H 0.31365000 -2.40803000 0.06444100
H -0.14077700 -1.60731000 -1.44288200
H -1.47448200 -1.23693300 1.29610600
H -2.21890600 -1.90075400 -0.15796300
H -2.08100400 0.26914100 -1.30508000
H -2.73068800 0.63640800 0.29675000
N -0.29810300 2.27741300 0.18680500
C 1.40109100 -0.24893600 1.36437500
H 1.76672800 0.75559400 1.59695400
H 0.65834200 -0.52275500 2.12046100
H 2.24866900 -0.94647800 1.46834600
C 1.79589700 0.15190300 -1.11612300
H 2.20054700 1.14131000 -0.88157100
H 2.64740500 -0.54301700 -1.20685300
H 1.30957600 0.20427600 -2.09559500

No.	Species	Optimized Structure
24	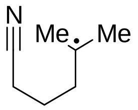	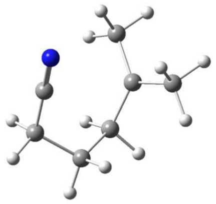

Cartesian Coordinates

C	-1.67467000	0.88024100	0.02692200
N	-1.55576400	2.00056500	0.31236200
C	-1.87441200	-0.53162400	-0.31863800
H	-2.88271900	-0.80515700	0.01412500
H	-1.86743500	-0.61987300	-1.41229500
C	-0.83996600	-1.49669500	0.30209600
H	-0.79769900	-1.33047300	1.38438100
H	-1.21847100	-2.51552100	0.15351300
C	0.58174400	-1.40269100	-0.30718900
H	0.51276600	-1.60958800	-1.38334600
H	1.15804700	-2.23617500	0.12991900
C	1.31092000	-0.11086900	-0.07924400
C	1.70759600	0.29051300	1.30918400
H	2.77045400	0.57297100	1.35492500
H	1.14247900	1.17019500	1.65675500
H	1.54623400	-0.51248900	2.03720400
C	1.66190600	0.81415200	-1.20298300
H	1.39389900	0.40043700	-2.18096100
H	1.15332400	1.78644200	-1.10175900
H	2.74076100	1.03711200	-1.21988200

No.	Species	Optimized Structure
25	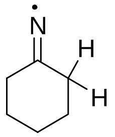	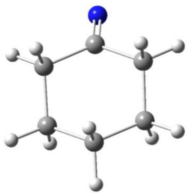

Cartesian Coordinates

C	-0.96314500	-1.26970600	-0.28430500
C	0.43209000	-1.29222600	0.37038500
C	1.19037300	0.00000000	0.06708800
C	0.43208900	1.29222600	0.37038400
C	-0.96314700	1.26970600	-0.28430400
C	-1.74515900	-0.00000100	0.08440200
H	0.32499700	-1.36523800	1.46208800
H	1.02121900	-2.15027700	0.03631400
H	-0.84742600	-1.32437900	-1.37501900

No.	Species	Optimized Structure
H -1.51935600 -2.16470600 0.01907300		
H 0.32499600 1.36523800 1.46208800		
H 1.02121800 2.15027700 0.03631300		
H -1.51935700 2.16470500 0.01907900		
H -0.84743000 1.32438100 -1.37501800		
H -1.95251500 -0.00000100 1.16454500		
H -2.71861000 -0.00000200 -0.42009400		
N 2.34480900 0.00000100 -0.42446700		
26	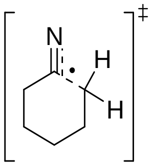	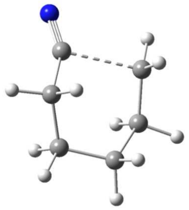
Cartesian Coordinates		
C 1.22603900 1.14795900 -0.31133100		
C -0.00593900 1.54599700 0.44553500		
C -1.44254400 -0.09736300 0.00691500		
C -0.61688000 -1.26315200 0.42581700		
C 0.74049000 -1.37327800 -0.29718800		
C 1.73459900 -0.25730300 0.05063000		
H -0.60031400 2.37474600 0.07550700		
H 1.03168100 1.20649200 -1.39073900		
H 2.03029500 1.87834600 -0.11266900		
H -0.45998000 -1.20516300 1.50964100		
H -1.21996100 -2.15766700 0.23153700		
H 1.17672100 -2.34379800 -0.03253000		
H 0.56642500 -1.39265300 -1.38051200		
H 1.96145400 -0.29419300 1.12602000		
H 2.67966600 -0.44863900 -0.47169800		
H 0.00185500 1.43495300 1.52968500		
N -2.42606200 0.39006000 -0.42950100		
27	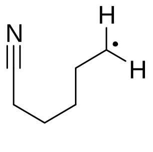	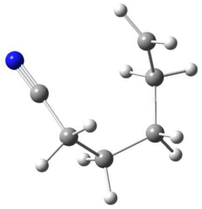
Cartesian Coordinates		
C 1.54880100 0.68577700 -0.47532900		
C 1.07189400 1.63012400 0.57869000		
C -1.72148300 0.19808700 -0.00295500		
C -1.04172500 -0.95062600 0.60534200		

No.	Species	Optimized Structure

C	0.13315900	-1.51341000	-0.23466800
C	1.49231500	-0.81105200	-0.07887600
H	0.39811700	2.44791100	0.35151600
H	0.99407800	0.84500900	-1.40795500
H	2.60319100	0.91486700	-0.71104900
H	-0.70062200	-0.66315000	1.60650400
H	-1.80165300	-1.72992500	0.73930000
H	0.26125500	-2.56164600	0.06028600
H	-0.16145200	-1.52421300	-1.29081100
H	1.84046100	-0.91845400	0.95804700
H	2.20918000	-1.36481400	-0.69826200
N	-2.28973200	1.07781300	-0.50610600
H	1.48780500	1.57632400	1.58194700

No.	Species	Optimized Structure
28	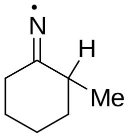	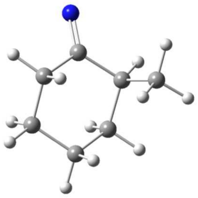

Cartesian Coordinates

C	0.42017200	1.22476400	-0.86585600
C	-0.91895400	0.62591500	-0.37186700
C	-0.74467000	-0.88766900	-0.15354300
C	0.46196700	-1.32616300	0.67701000
C	1.75486300	-0.68716000	0.13501100
C	1.62276600	0.83789500	0.01022300
H	-1.67017600	0.75008000	-1.15889000
H	0.60155600	0.87441500	-1.89060700
H	0.32168000	2.31641000	-0.92069900
H	0.29724100	-1.00514200	1.71436200
H	0.52131600	-2.41769200	0.67717700
H	2.59221000	-0.94902800	0.79281500
H	1.97998100	-1.11721000	-0.85017300
H	1.52018600	1.28008100	1.01037400
H	2.53945300	1.25933800	-0.41904600
N	-1.53765100	-1.71705000	-0.66033600
C	-1.45443800	1.30665700	0.90277000
H	-2.41187700	0.86946400	1.20199200
H	-0.76514600	1.21876000	1.74831500
H	-1.61309800	2.37443800	0.71424200

No.	Species	Optimized Structure
29	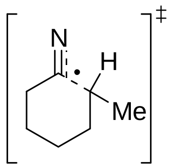	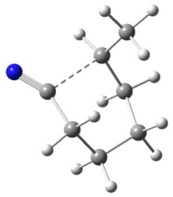

Cartesian Coordinates

C	1.03052500	0.91974000	-0.87889200
C	-0.36191300	1.23719500	-0.39271300
C	-1.27203900	-0.76402300	-0.11749600
C	-0.23863200	-1.44388200	0.71408800
C	1.17286100	-1.44908600	0.09589800
C	1.82183500	-0.06227800	0.00378400
H	-1.06139300	1.53290000	-1.17061700
H	0.97279900	0.51706700	-1.89806500
H	1.60385600	1.86152000	-0.95627200
H	-0.21359900	-0.96242800	1.69906100
H	-0.58865700	-2.47129500	0.86649800
H	1.80094100	-2.10563000	0.70986500
H	1.12415700	-1.90422500	-0.90168500
H	1.94416000	0.35210100	1.01339300
H	2.83361600	-0.16935000	-0.40537200
N	-2.30992700	-0.77330100	-0.68601000
C	-0.55688500	1.94628100	0.91878100
H	-1.61266800	1.96592100	1.20618300
H	0.01424300	1.49826700	1.73900000
H	-0.22247700	2.99457900	0.83938700

No.	Species	Optimized Structure
30	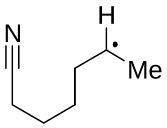	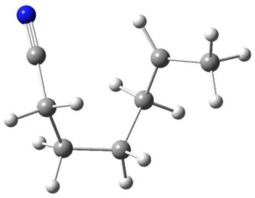

Cartesian Coordinates

C	1.09136000	-0.44895100	-0.98233900
C	1.43036800	0.78954900	-0.20743000
C	-1.80305300	0.92971800	0.06938000
C	-1.59137600	-0.27781100	0.87404800
C	-1.08136400	-1.50071400	0.06856200
C	0.43631100	-1.59349600	-0.15882000
H	0.90522000	1.71380100	-0.42684200
H	0.43973100	-0.19992800	-1.82883800
H	2.01801700	-0.86044000	-1.41647100

No.	Species	Optimized Structure

H	-0.89916800	-0.03511800	1.68866900
H	-2.55605600	-0.51949600	1.33628500
H	-1.39265300	-2.39547600	0.62067800
H	-1.60580000	-1.53462900	-0.89380900
H	0.94326600	-1.67598300	0.81209000
H	0.62131000	-2.54295900	-0.67761200
N	-2.00893400	1.87466500	-0.57463400
C	2.63133300	0.83429500	0.68063000
H	2.60215300	1.68681800	1.36700200
H	2.74015300	-0.07999000	1.28073300
H	3.56489300	0.92520400	0.09636900

No.	Species	Optimized Structure
31	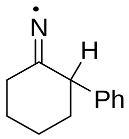	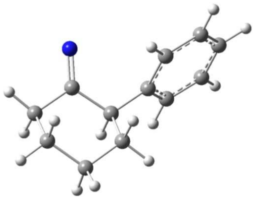

Cartesian Coordinates

C	3.42748500	-0.09703300	-0.38197100
C	2.82129800	0.84712900	0.67180300
C	1.31555400	1.01497500	0.45090300
C	0.52157100	-0.30450200	0.38414900
C	1.17481300	-1.22999800	-0.67799300
C	2.67653600	-1.43444900	-0.43445500
H	2.96574000	0.42362200	1.67583400
H	3.29976500	1.82980500	0.65513700
H	3.38318300	0.38864900	-1.36582100
H	4.48843100	-0.25880700	-0.15696900
H	0.67692700	-0.77940600	1.36431700
H	0.64848500	-2.19135700	-0.66852700
H	1.01534500	-0.79477900	-1.67303700
H	2.82221500	-1.97217700	0.51370700
H	3.09568000	-2.07222400	-1.22168000
N	0.80691700	2.15405500	0.32227300
C	-0.97361300	-0.14949000	0.17461800
C	-1.86764500	-0.74413700	1.07482100
C	-1.49761400	0.53380300	-0.93352300
C	-3.24958700	-0.66201600	0.87944400
H	-1.48033700	-1.27514000	1.94151800
C	-2.87662100	0.61943100	-1.13156600
H	-0.82631500	1.01654200	-1.63702700
C	-3.75870700	0.02066800	-0.22666600
H	-3.92383200	-1.12821900	1.59238700

No.	Species	Optimized Structure
H	-3.26296400 1.15928600 -1.99146400	
H	-4.83156000 0.08953000 -0.38167100	
32	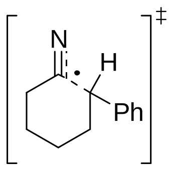	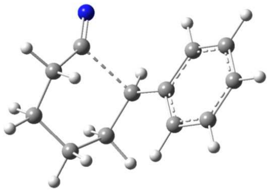

Cartesian Coordinates

C	1.44922300	-0.92908700	1.35699100
C	0.47989500	0.20824200	1.08695200
C	1.50349200	1.42595400	-0.27450000
C	1.97606500	0.48245000	-1.33298900
C	2.86599700	-0.64083100	-0.77216100
C	2.10078100	-1.60989300	0.13506000
H	2.25017400	-0.53777300	1.99568700
H	0.93379400	-1.69762800	1.95534700
H	1.09838900	0.05397000	-1.83196800
H	2.52212600	1.07800500	-2.07223600
H	3.29405000	-1.19322400	-1.61709000
H	3.70794000	-0.19456700	-0.22751800
H	1.34737600	-2.13158000	-0.46565200
H	2.78849400	-2.38185700	0.50117700
N	1.49802800	2.55586300	0.10359800
C	-0.85239900	0.00802600	0.50945600
C	-1.30916600	-1.22693800	-0.00318800
C	-1.75020700	1.10263200	0.47392000
C	-2.59684300	-1.35802400	-0.52538800
H	-0.66844100	-2.10071200	0.03171900
C	-3.02995000	0.97128800	-0.05425600
H	-1.41657700	2.06438700	0.85339300
C	-3.46273900	-0.26174000	-0.55979800
H	-2.92607300	-2.32291100	-0.90109200
H	-3.69511000	1.83000200	-0.07172200
H	-4.46309600	-0.36588900	-0.96943100
H	0.49586800	0.97627400	1.85360200

No.	Species	Optimized Structure
33	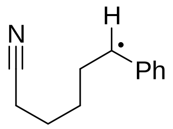	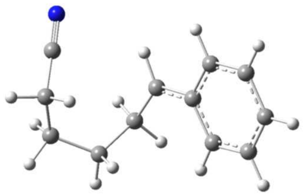

Cartesian Coordinates

No.	Species	Optimized Structure

C	0.95442700	1.08303800	-1.20525800
C	0.12628500	-0.13620300	-0.92087200
C	2.93739000	-1.35045000	0.22307500
C	2.78128700	-0.26737400	1.19872500
C	2.88863700	1.15718200	0.59373900
C	1.59495800	1.77146600	0.03460800
H	0.57176600	-1.10831100	-1.11090600
H	1.74810700	0.82363400	-1.91630200
H	0.33173900	1.83710300	-1.70622600
H	1.82243600	-0.39878700	1.71385200
H	3.56706600	-0.40766600	1.95052000
H	3.24459500	1.81399400	1.39610200
H	3.66994500	1.16040400	-0.17547500
H	0.84896700	1.83349100	0.83709100
H	1.83384300	2.80632000	-0.24303800
N	3.09257300	-2.20627900	-0.54738500
C	-1.20433200	-0.12230700	-0.42861700
C	-1.92938300	1.07743900	-0.15893300
C	-1.88260400	-1.35586500	-0.18578900
C	-3.23328800	1.03673400	0.32017700
H	-1.46010600	2.03999500	-0.33576900
C	-3.18402800	-1.38488800	0.29352100
H	-1.35726000	-2.28598800	-0.38694600
C	-3.87318800	-0.18932100	0.55260900
H	-3.76153400	1.96642100	0.51409100
H	-3.67221400	-2.34001400	0.46654900
H	-4.89233200	-0.21335100	0.92625200

34	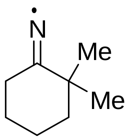	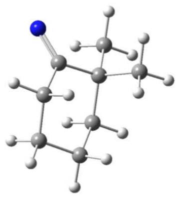

Cartesian Coordinates

C	-0.10789900	-1.30189200	-0.66649700
C	0.85126000	-0.29532200	0.02733300
C	0.17357500	1.09881500	0.00819900
C	-1.28137500	1.17225000	0.48240000
C	-2.15169800	0.13855200	-0.25379000
C	-1.55468300	-1.27099300	-0.15043000
H	-0.11339000	-1.08217000	-1.74301800
H	0.31456300	-2.30944500	-0.55691300
H	-1.30263700	0.97476500	1.56231400

No.	Species	Optimized Structure

H -1.65166100 2.18948500 0.33027000
H -3.16804900 0.15951700 0.15757400
H -2.23178400 0.42615700 -1.31076400
H -1.59452800 -1.61275500 0.89242900
H -2.16097600 -1.97906000 -0.72791800
N 0.76299600 2.13023000 -0.39428900
C 1.11331000 -0.69778900 1.49720100
H 1.78207500 0.02206800 1.97998100
H 0.19825800 -0.75933500 2.09225700
H 1.59623800 -1.68150800 1.52683200
C 2.19096600 -0.25939300 -0.72673800
H 2.89226700 0.43672000 -0.25745800
H 2.64411500 -1.25739700 -0.72623500
H 2.05379400 0.05597700 -1.76539000

35	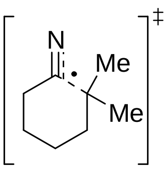	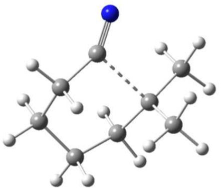

Cartesian Coordinates
C 0.04036300 -1.36785900 -0.69119400
C 0.99055300 -0.43753400 0.03601000
C -0.12355400 1.46180500 0.00411100
C -1.47066800 1.06274800 0.51211500
C -2.13399500 -0.07696600 -0.28149600
C -1.40293500 -1.41964900 -0.16546100
H 0.02224700 -1.09415900 -1.75447700
H 0.46786100 -2.38722400 -0.65263500
H -1.37518200 0.77784500 1.56632100
H -2.09495800 1.96264800 0.47751900
H -3.16231400 -0.18571600 0.08386900
H -2.20517500 0.21730300 -1.33661000
H -1.41525900 -1.75563400 0.87977000
H -1.95642400 -2.17735800 -0.73308800
N 0.58162400 2.32870300 -0.39284900
C 1.19612900 -0.67038400 1.51419000
H 1.74715800 0.15770100 1.97136300
H 0.26420400 -0.81061800 2.06894600
H 1.79765200 -1.58303700 1.66643300
C 2.25156300 -0.10708400 -0.71904800
H 2.03971100 0.21221100 -1.74328600
H 2.81472900 0.69214500 -0.22990500

No.	Species	Optimized Structure
H	2.89964400 -0.99749400 -0.76964300	
36	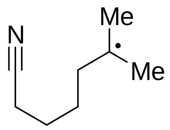	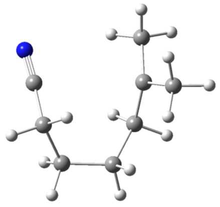
Cartesian Coordinates		
C	-0.77457100 0.85629900 -0.96010700	
C	-1.42507000 -0.09901300 0.00787200	
C	1.62163900 -1.25973300 0.23001100	
C	1.86580100 0.06863300 0.80075800	
C	1.71808500 1.23286500 -0.21343500	
C	0.30461300 1.81044000 -0.39808100	
H	-0.35062300 0.29139400 -1.80054300	
H	-1.56610700 1.49171500 -1.40519900	
H	1.19490700 0.21073800 1.65533100	
H	2.88827100 0.05468900 1.19739400	
H	2.36645100 2.04444300 0.13746800	
H	2.12206500 0.91343200 -1.18143900	
H	-0.03518300 2.23690000 0.55439600	
H	0.39939800 2.66093600 -1.08491600	
N	1.48588900 -2.31543800 -0.23608000	
C	-2.12677800 0.44676300 1.21695200	
H	-2.25544100 -0.32312700 1.98700700	
H	-1.60691200 1.29593000 1.67340000	
H	-3.14285400 0.80398900 0.96134400	
C	-1.90036500 -1.41726200 -0.52313600	
H	-2.14947300 -2.11409900 0.28509400	
H	-2.81670000 -1.29744600 -1.13212300	
H	-1.14914100 -1.89538000 -1.15966800	
37	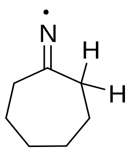	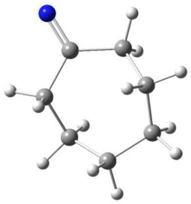
Cartesian Coordinates		
C	0.59185900 -1.53715800 -0.39779000	
C	1.80495900 -0.75580100 0.13460100	
C	1.80495200 0.75577700 -0.13467400	
C	-0.72161500 -1.28174300 0.36617200	
C	0.59190000 1.53714200 0.39781900	

No.	Species	Optimized Structure

C	-1.47549400	0.00001000	0.00001700
C	-0.72162400	1.28179200	-0.36607300
H	0.45212300	-1.34129900	-1.46963900
H	1.88163300	-0.92974400	1.21807500
H	1.88153800	0.92972000	-1.21815400
H	0.45222700	1.34125300	1.46966900
H	-0.50663000	-1.23868900	1.44322400
H	0.81265500	-2.60834700	-0.31467300
H	2.71617300	-1.18187900	-0.30495100
H	2.71620500	1.18184800	0.30480300
H	-1.41551800	-2.11551900	0.22452100
H	0.81271500	2.60832900	0.31472300
H	-1.41551300	2.11556000	-0.22430800
H	-0.50671000	1.23883100	-1.44314300
N	-2.73293200	-0.00002500	-0.00008200

No.	Species	Optimized Structure
38	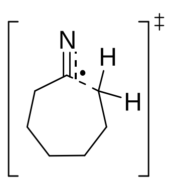	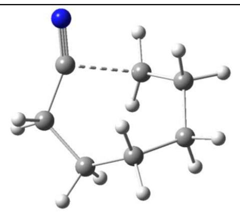

Cartesian Coordinates

C	0.19880600	1.62373300	0.45948800
C	1.19427100	1.09015600	-0.59542200
C	1.93389100	-0.19938100	-0.19162600
C	-1.27835800	1.23805100	0.25181500
C	1.08028100	-1.47481100	-0.10202200
C	-1.58823500	-0.16641800	-0.14656700
C	-0.05131800	-1.43439500	0.88280300
H	0.52612500	1.31271500	1.45786000
H	0.68030000	0.94098400	-1.55503300
H	2.42665300	-0.02822500	0.77663700
H	0.68147000	-1.73230400	-1.09247600
H	-1.71306500	1.85895700	-0.54101700
H	0.22504600	2.71973200	0.46846400
H	1.95253700	1.86130500	-0.77695800
H	2.74032400	-0.37876600	-0.91383900
H	-1.84818200	1.45178600	1.16417100
H	1.74830000	-2.31224400	0.16729900
H	-0.68795200	-2.31103400	0.94550600
H	0.09404300	-0.91216500	1.82790200
N	-2.25166000	-0.93319300	-0.75419100

No.	Species	Optimized Structure
39	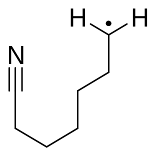	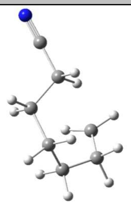

Cartesian Coordinates

C	-0.51336800	-1.09073000	-0.33162200
C	0.89595800	-1.49629000	0.14559000
C	2.09107500	-0.63098300	-0.30420500
C	-1.20392600	-0.03557700	0.56087600
C	2.24156500	0.77252600	0.32213300
C	-2.55958300	0.28415400	0.10612800
C	1.40238400	1.85104700	-0.27841500
H	-0.47033800	-0.71928000	-1.36246300
H	0.90022600	-1.58082600	1.24226400
H	2.07504900	-0.53395000	-1.39907700
H	2.07284500	0.71803000	1.40812500
H	-0.62167700	0.89408400	0.57449800
H	-1.15137900	-1.98140600	-0.35238400
H	1.08386000	-2.51067600	-0.22754600
H	2.99974000	-1.19888500	-0.06825200
H	-1.26660500	-0.39792000	1.59441600
H	3.30406000	1.06496500	0.22330300
H	1.23253400	2.78232600	0.25402100
H	1.13895000	1.82393900	-1.33238600
N	-3.63169900	0.53067300	-0.26820300

No.	Species	Optimized Structure
40	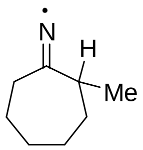	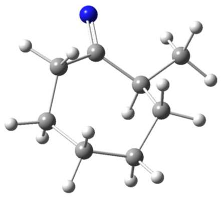

Cartesian Coordinates

C	-1.68754700	1.07009000	-0.43859100
C	-2.27904800	-0.25079100	0.07681100
C	-1.40506700	-1.49647700	-0.12225900
C	-0.48172400	1.58454000	0.37188200
C	0.01414600	-1.43297800	0.46862800
C	0.87979800	0.96565300	0.03325800
C	1.01160700	-0.53434200	-0.29966500
H	-1.42189200	0.98340100	-1.50087500
H	-2.50286800	-0.13499300	1.14749700

H	-1.32350200	-1.71359700	-1.19745300
H	-0.02282100	-1.14023700	1.52713400
H	-0.66639200	1.40798200	1.44073500
H	-2.46775800	1.83920400	-0.38570400
H	-3.24410800	-0.42317200	-0.41715000
H	-1.92797000	-2.35474200	0.31909100
H	-0.37669900	2.66672000	0.25276300
H	0.42692100	-2.44929600	0.45617100
H	0.76133200	-0.61082600	-1.36938000
N	1.88748200	1.71746100	0.02279300
C	2.46065400	-1.01680200	-0.12392500
H	2.55975900	-2.04700200	-0.48186000
H	3.16165800	-0.38780300	-0.67825100
H	2.75504600	-0.99122400	0.93089600

No.	Species	Optimized Structure
41	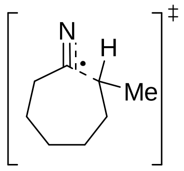	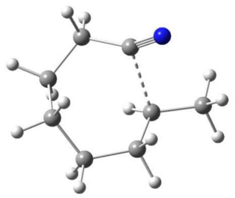

Cartesian Coordinates

C	-1.82164900	0.52903900	-0.70107400
C	-1.93999700	-0.53788000	0.40896200
C	-1.01689800	-1.75867300	0.23098500
C	-0.86233300	1.70224100	-0.40801600
C	0.49346700	-1.51882800	0.39348800
C	0.40287200	1.39085800	0.32214100
C	1.11780000	-0.52490900	-0.55071200
H	-1.53923700	0.04044900	-1.64038300
H	-1.76955200	-0.07815700	1.39212600
H	-1.20448600	-2.19116300	-0.76284000
H	0.72103800	-1.21867900	1.42571600
H	-1.37206000	2.44867900	0.21276900
H	-2.80418600	0.97845800	-0.88608500
H	-2.97232900	-0.90786700	0.42422800
H	-1.31592200	-2.52715200	0.95491400
H	-0.59234400	2.20469000	-1.34450600
H	1.00530300	-2.48842500	0.25506000
H	0.65618000	-0.44090300	-1.53584000
N	1.17984500	1.69918600	1.16045900
C	2.61191300	-0.38721500	-0.53356600
H	3.08316100	-1.30935900	-0.91009300
H	2.95330900	0.43984500	-1.16317000

No.	Species	Optimized Structure

H	2.98116700	-0.21251800	0.48164200

No.	Species	Optimized Structure
42	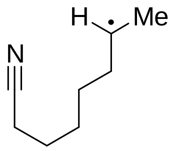	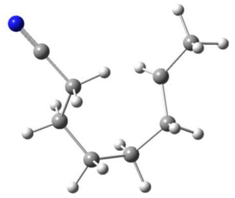

Cartesian Coordinates

C	-1.05390200	-1.26989200	-0.28113100
C	0.20316100	-2.01610000	0.20985400
C	1.57784900	-1.53114900	-0.29490700
C	-1.40976600	-0.01854600	0.55223700
C	2.10847200	-0.18436000	0.23903700
C	-2.63482700	0.63722300	0.08727100
C	1.59325700	1.04477500	-0.44122500
H	-0.93471500	-0.98142900	-1.33229300
H	0.20761600	-2.03986300	1.30956600
H	1.56817200	-1.50079200	-1.39375300
H	1.93209600	-0.11659700	1.32518700
H	-0.59430000	0.71358700	0.50676300
H	-1.91029900	-1.95256700	-0.24420000
H	0.10216300	-3.06201000	-0.10577000
H	2.30476100	-2.30732300	-0.02503300
H	-1.54746600	-0.29274200	1.60539000
H	3.21188300	-0.19769800	0.14402200
H	1.27215200	0.95915500	-1.47849500
N	-3.60522100	1.14984100	-0.29480300
C	1.89315000	2.40551400	0.09777100
H	2.95178300	2.68230600	-0.05645100
H	1.28907500	3.17989600	-0.38574400
H	1.71925900	2.46241000	1.18098800

No.	Species	Optimized Structure
43	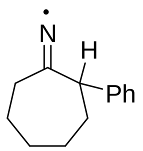	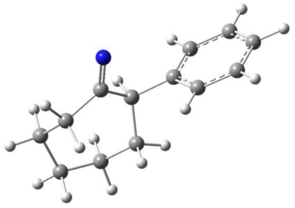

Cartesian Coordinates

C	-3.40495600	0.45755300	-0.41324300
C	-3.48658500	-0.90133200	0.29598300
C	-2.25260600	-1.80206100	0.15263600
C	-2.36938400	1.42689400	0.19183900
C	-0.91393200	-1.20846700	0.62753300

No.	Species	Optimized Structure

C	-0.92454500	1.23765000	-0.27671300
C	-0.27610200	-0.16596400	-0.33057000
H	-3.20908400	0.31942100	-1.48520500
H	-3.67963200	-0.72453300	1.36429200
H	-2.14522000	-2.10882200	-0.89823700
H	-1.01530600	-0.78701400	1.63644800
H	-2.38275100	1.33395800	1.28693100
H	-4.38708600	0.94104800	-0.34589000
H	-4.36007600	-1.44704600	-0.08363000
H	-2.43735300	-2.72502100	0.71707400
H	-2.64052600	2.46169900	-0.03602700
H	-0.19277300	-2.02814900	0.71794400
N	-0.28254900	2.25301600	-0.64767800
H	-0.46639800	-0.52206200	-1.35296800
C	1.23668700	-0.10715300	-0.15264000
C	2.08213100	-0.66371600	-1.12027500
C	1.81400000	0.44182800	1.00241400
C	3.46938200	-0.67387900	-0.94439600
H	1.65367300	-1.08769800	-2.02546100
C	3.19737900	0.43188000	1.18361800
H	1.18016400	0.89545900	1.75941600
C	4.03173200	-0.12728800	0.21025000
H	4.10676800	-1.10440100	-1.71163300
H	3.62535800	0.86695600	2.08239700
H	5.10888100	-0.13057100	0.34968200

No.	Species	Optimized Structure
44	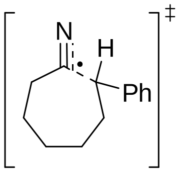	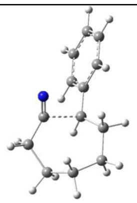

Cartesian Coordinates

C	3.13553800	-0.84082100	-0.62255900
C	3.37942500	0.57689400	-0.06323200
C	2.39818800	1.65284700	-0.56999500
C	2.17803900	-1.73826600	0.20059800
C	0.93878600	1.56738800	-0.08933300
C	1.09986400	-1.02155000	0.95538400
C	0.22011900	0.28193600	-0.44429400
H	2.77684900	-0.75825000	-1.65549000
H	3.38052400	0.55299900	1.03470300
H	2.41063700	1.63842000	-1.66977500
H	0.89366300	1.73006900	0.99405400

No.	Species	Optimized Structure

H	2.74404600	-2.29117800	0.95822000
H	4.08855500	-1.37840100	-0.68440800
H	4.38725000	0.89124600	-0.36060100
H	2.78587200	2.63677600	-0.27812900
H	1.70655000	-2.48344300	-0.45044300
H	0.38617900	2.40554100	-0.54258200
N	0.61365300	-0.88016400	2.03306400
C	-1.22790600	0.16447100	-0.29239700
C	-1.95023100	-0.72676200	-1.11590000
C	-1.94782100	0.90508400	0.66910300
C	-3.33083200	-0.86618200	-0.99315000
H	-1.41546200	-1.30838700	-1.86344600
C	-3.32855600	0.76475400	0.79152600
H	-1.41958000	1.57952200	1.33454100
C	-4.02845300	-0.11915400	-0.03754700
H	-3.86415100	-1.55475300	-1.64249200
H	-3.86191100	1.34203800	1.54150500
H	-5.10463300	-0.22619000	0.06193600
H	0.59306900	-0.21870500	-1.33826700

No.	Species	Optimized Structure
45	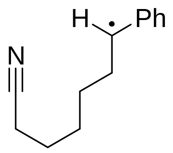	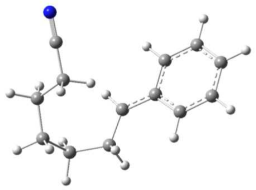

Cartesian Coordinates

C	3.04579600	0.16930400	0.15307200
C	3.06118000	-1.32375100	-0.23541300
C	2.02045600	-2.25460000	0.41992600
C	2.09894900	1.03894200	-0.70547100
C	0.54295400	-2.07957100	0.01203600
C	2.15780300	2.45586600	-0.33473900
C	-0.19406600	-0.98849800	0.73048300
H	2.78265800	0.28364300	1.21135700
H	2.99278200	-1.41629600	-1.32920100
H	2.10315200	-2.17692800	1.51319100
H	0.48005900	-1.94071000	-1.07788400
H	1.06008200	0.70642500	-0.60574400
H	4.05781800	0.57500800	0.04475300
H	4.04892400	-1.71956900	0.03115500
H	2.31527500	-3.28168100	0.17200800
H	2.36773000	0.95250600	-1.76546600
H	0.02485900	-3.03588100	0.20132200

No.	Species	Optimized Structure

N 2.21789900 3.57420400 -0.02520700
H 0.24149900 -0.61806300 1.65705800
C -1.45957400 -0.46650100 0.35870900
C -2.07260800 0.55107200 1.15071400
C -2.17418500 -0.91140000 -0.79351900
C -3.30603500 1.08660500 0.81044800
H -1.55139400 0.91019800 2.03450200
C -3.41070700 -0.36921900 -1.12340200
H -1.75092000 -1.69016700 -1.41988200
C -3.98764200 0.63238000 -0.32971000
H -3.74464300 1.86323100 1.43056200
H -3.93422900 -0.72781800 -2.00542900
H -4.95286000 1.05290100 -0.59465800

No.	Species	Optimized Structure
46	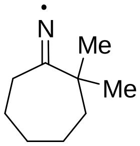	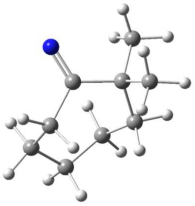

Cartesian Coordinates
C -2.16718700 0.79500300 -0.05978800
C -2.30519200 -0.73113600 0.03378300
C -1.09581800 -1.54367400 -0.46166700
C -0.91505600 1.38839500 0.62114600
C 0.15699600 -1.41067300 0.42880800
C 0.36922100 1.07782900 -0.15661200
C 1.13364500 -0.24693300 0.10586500
H -2.16414600 1.10563000 -1.11210900
H -2.50494700 -1.01107800 1.07859400
H -0.85861500 -1.26799000 -1.49819900
H -0.16554400 -1.33956400 1.47631300
H -0.82315800 1.01569200 1.64890300
H -3.05248800 1.25576400 0.39678900
H -3.19468700 -1.03018500 -0.53551400
H -1.38899900 -2.60014900 -0.49139500
H -1.01645500 2.47600200 0.67472900
H 0.74910900 -2.33246900 0.36596200
N 0.78749000 1.91631200 -0.99247500
C 2.05358400 -0.01046800 1.32629200
H 2.56810800 -0.94225600 1.58971300
H 2.80942600 0.74901200 1.10485400
H 1.48684600 0.31637800 2.20499900
C 1.99992800 -0.60438200 -1.11810000

No.	Species	Optimized Structure

H	1.38546300	-0.81170300	-1.99940700
H	2.67737600	0.21447200	-1.37436600
H	2.59955300	-1.49549400	-0.90090300

No.	Species	Optimized Structure
47	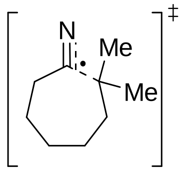	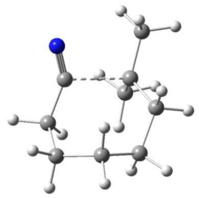

Cartesian Coordinates

C	2.33492600	0.43579900	0.23063800
C	1.97136600	-0.76918600	-0.65326000
C	0.91831800	-1.74518900	-0.08307500
C	1.13922700	1.18571900	0.85645900
C	-0.53281600	-1.45666100	-0.52513200
C	0.03383000	1.43266500	-0.12020800
C	-1.29486200	-0.29342700	0.08473700
H	2.97945400	0.10972500	1.05720000
H	1.61850800	-0.40412500	-1.62632200
H	0.99254100	-1.79599500	1.01189200
H	-0.53934700	-1.34781600	-1.61698100
H	1.46919200	2.16333900	1.22536900
H	2.92373400	1.14109000	-0.36673600
H	2.89949700	-1.31687100	-0.85701500
H	1.15758900	-2.75492000	-0.43709400
H	0.75786900	0.63309300	1.71520700
H	-1.13467200	-2.36012300	-0.31360700
N	-0.38605500	2.16718400	-0.95528700
C	-2.49391400	0.14041200	-0.72384500
H	-3.23399000	-0.67552900	-0.75072300
H	-2.97968700	1.01813300	-0.28800000
H	-2.22064400	0.38650800	-1.75266400
C	-1.57196000	-0.38201900	1.56913500
H	-0.72697400	-0.76118600	2.15097900
H	-1.87613300	0.58495600	1.98444000
H	-2.40924700	-1.07924800	1.73836600

No.	Species	Optimized Structure
48	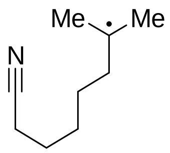	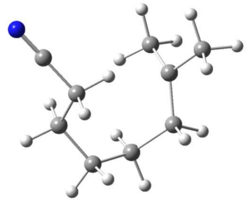

Cartesian Coordinates

No.	Species	Optimized Structure
C	-1.36538800 1.28048700 0.26919600	
C	-0.19196200 2.15545700 -0.21479700	
C	1.23403600 1.78306400 0.24515900	
C	-1.59510300 0.00835200 -0.57535100	
C	1.92606700 0.57957700 -0.43595000	
C	-2.73386800 -0.78413200 -0.10560100	
C	1.57058300 -0.81507000 0.01023800	
H	-1.21410900 0.99482300 1.31703800	
H	-0.21484100 2.22389600 -1.31252700	
H	1.23963500 1.65579700 1.33524700	
H	1.78263800 0.65933900 -1.52290100	
H	-0.69982600 -0.62655100 -0.55050100	
H	-2.28569800 1.87490300 0.24239200	
H	-0.38344100 3.17344300 0.14686500	
H	1.86434500 2.66015000 0.05182300	
H	-1.77434000 0.27804500 -1.62351400	
H	3.01675900 0.71083500 -0.28504000	
N	-3.63519500 -1.40809800 0.28033700	
C	1.84002900 -1.93372200 -0.95343400	
H	2.91657400 -2.18689600 -0.98698900	
H	1.31434000 -2.85306400 -0.67023500	
H	1.55072000 -1.67349400 -1.97857700	
C	1.57550500 -1.15367200 1.47168400	
H	1.08921600 -0.39170200 2.08912800	
H	1.07648700 -2.11013200 1.66332600	
H	2.60851800 -1.25475700 1.85522700	

7.3 Activation Energy ($\Delta G^{‡}$) and Reaction Energy (ΔG^{o}) for 1,5-H atom Abstraction

No.	1,5-H-atom abstraction	$\Delta G^{\neq}$	ΔG
1	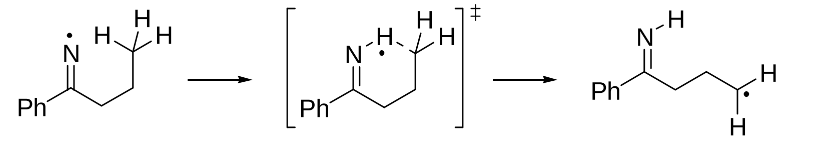	18.2	11.0
2	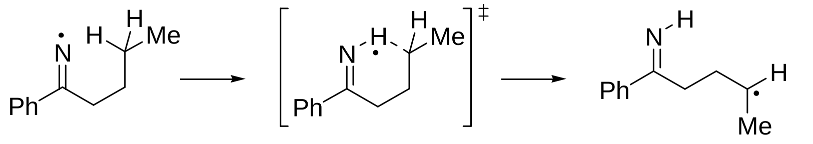	15.7	6.7
3	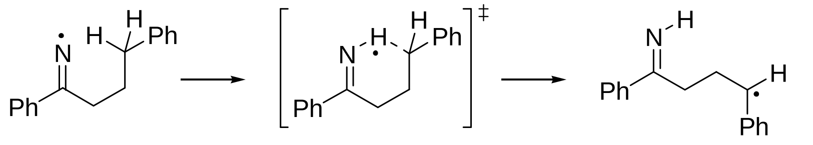	13.1	-2.1
4	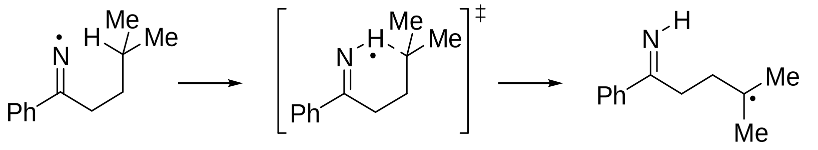	13.7	4.2
5	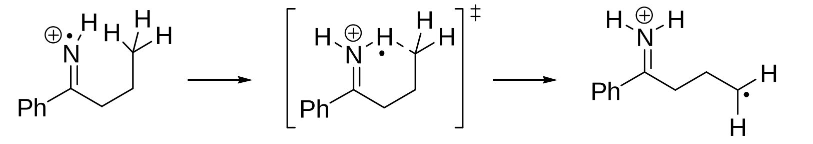	1.9	-15.8
6	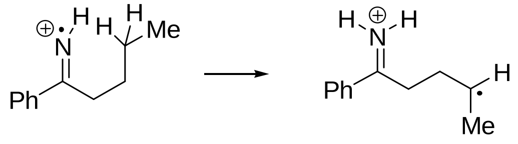		-21.2
7	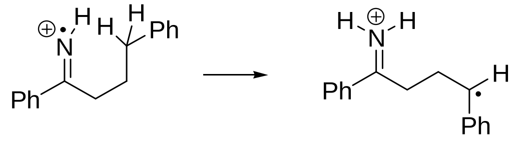		-24.6
8	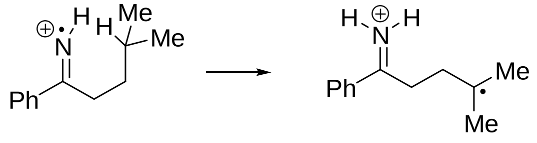		-24.9

DFT Method: UB3LYP/6-31+G(d,p) [values are in Kcal mol^{-1}]

Computed Energies [values are in Hartree]

No.	Species	Total Electronic Energy	Sum of Electronic and Zero-point Energies	Gibbs Free Energy
1	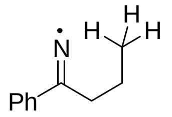	-443.0235649	-442.829472	-442.867708
2	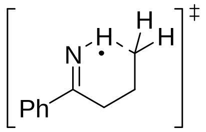	-442.9914959	-442.802530	-442.838714
3	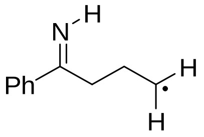	-443.0050384	-442.812436	-442.850257
4	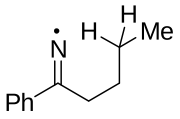	-482.3410005	-482.118455	-482.158831
5	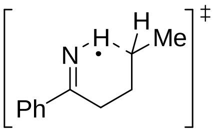	-482.3126879	-482.095479	-482.133745
6	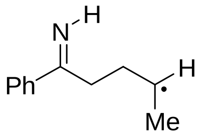	-482.3286596	-482.107689	-482.148180
7	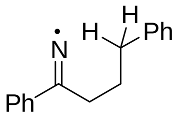	-674.0885445	-673.813030	-673.859863
8	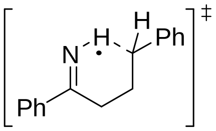	-674.0658605	-673.795136	-673.838936
9	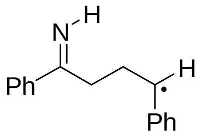	-674.0936418	-673.818234	-673.863262
10	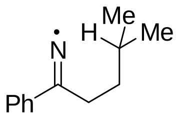	-521.6577733	-521.407302	-521.449929

No.	Species	Total Electronic Energy	Sum of Electronic and Zero-point Energies	Gibbs Free Energy
11	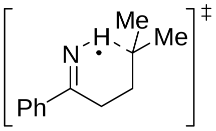	-521.6332498	-521.388149	-521.428127
12	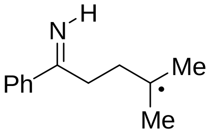	-521.6504017	-521.400957	-521.443224
13	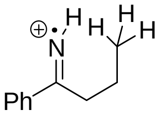	-443.3647098	-443.160177	-443.197973
14	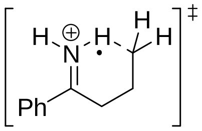	-443.3619859	-443.159096	-443.195650
15	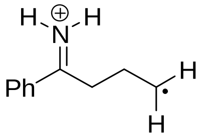	-443.3931927	-443.186642	-443.223797
16	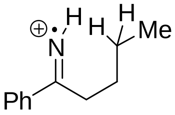	-482.6838595	-482.450670	-482.490647
17	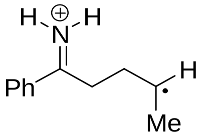	-482.7193286	-482.484544	-482.524409
18	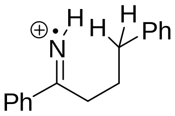	-674.4433058	-674.156379	-674.202150
19	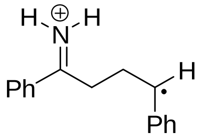	-674.4850309	-674.196189	-674.241345
20	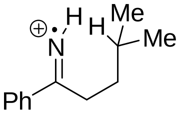	-522.0013441	-521.740222	-521.781792

No.	Species	Total Electronic Energy	Sum of Electronic and Zero-point Energies	Gibbs Free Energy
21	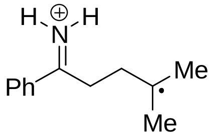	-522.0425908	-521.779580	-521.821416

Optimized Structures and Cartesian Coordinates

No.	Species	Optimized Structure
1	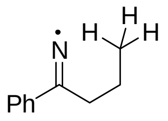	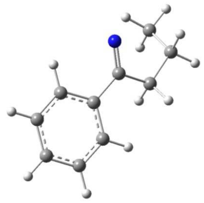

Cartesian Coordinates

C	-0.78190600	0.32911300	-0.38197000
N	-1.22495500	1.46294100	-0.71483600
C	-1.73567100	-0.86336000	-0.23137700
H	-1.48924400	-1.56949100	-1.03548900
H	-1.49289400	-1.37074500	0.71163800
C	-3.23390500	-0.53051700	-0.28085600
H	-3.77762000	-1.48062900	-0.35621900
H	-3.45212800	0.02826200	-1.19794700
C	-3.74677200	0.24831400	0.93665600
H	-3.26863000	1.22903800	1.01369100
C	0.68836000	0.12202400	-0.14910500
C	1.25203600	-1.16286000	-0.11793600
C	1.52726800	1.23584300	0.03524700
C	2.62427800	-1.33044600	0.08723800
H	0.62972100	-2.03991200	-0.25901900
C	2.89443300	1.06571800	0.24208600
H	1.09441400	2.23079400	0.02142800
C	3.44900000	-0.21877700	0.26819600
H	3.04552400	-2.33141100	0.10528300
H	3.52845900	1.93557600	0.38725700
H	4.51469400	-0.35053100	0.43108500
H	-4.82797200	0.40821100	0.86648500
H	-3.55234900	-0.30005800	1.86658800

No.	Species	Optimized Structure
2	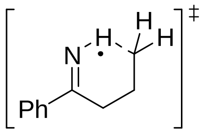	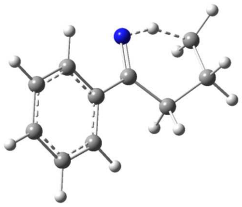

Cartesian Coordinates

C	-0.84535600	-0.22957000	-0.10754400
N	-1.34984100	-1.38635900	-0.29877100
C	-1.71374600	1.03016600	0.02814700
H	-1.23234400	1.74881600	0.69816600
H	-1.76952000	1.51037300	-0.95973900

No.	Species	Optimized Structure

C	-3.12447900	0.68800600	0.53497600
H	-3.75441700	1.58820100	0.46815400
H	-3.06527200	0.42613600	1.59855500
C	-3.71758200	-0.46824100	-0.23738400
H	-2.56771700	-1.24223200	-0.31011100
C	0.64591400	-0.08794800	-0.04138000
C	1.27667200	1.15726700	-0.19440700
C	1.44438100	-1.22851600	0.15885500
C	2.66988200	1.26022000	-0.15300900
H	0.68898100	2.05398700	-0.36087400
C	2.83232200	-1.12304800	0.21064900
H	0.95655400	-2.19038500	0.27467700
C	3.45164600	0.12232800	0.05335600
H	3.14107400	2.23052600	-0.28100200
H	3.43387300	-2.01267200	0.37413800
H	4.53413300	0.20337500	0.09235600
H	-4.47719800	-1.06489100	0.26854800
H	-3.95718700	-0.27069700	-1.28503100

No.	Species	Optimized Structure
3	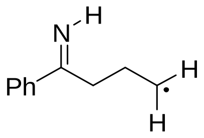 	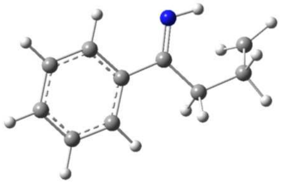

Cartesian Coordinates

C	-0.81147300	0.36985200	-0.11467500
N	-1.22181800	1.58367500	-0.10159600
C	-1.71984600	-0.85501900	-0.20172000
H	-1.34575700	-1.50202700	-1.00456500
H	-1.60698800	-1.43652700	0.72434100
C	-3.21081000	-0.57514100	-0.44507600
H	-3.69191900	-1.54949700	-0.64821000
H	-3.33861700	-0.00480800	-1.37662400
C	-3.91964800	0.10439700	0.67992000
C	0.66635300	0.12671000	-0.04225300
C	1.21121400	-1.16461300	0.05566400
C	1.54760400	1.22358400	-0.06536400
C	2.59400900	-1.35558700	0.13037100
H	0.56574100	-2.03572800	0.08132800
C	2.92526100	1.03323000	0.00482100
H	1.12533900	2.21954600	-0.13815800
C	3.45593900	-0.25849300	0.10357500
H	2.99366400	-2.36259600	0.20905300

No.	Species	Optimized Structure	
H	3.58903200	1.89301200	-0.01705100
H	4.53072500	-0.40622500	0.15897300
H	-4.87716400	0.58945600	0.51901700
H	-3.58018100	-0.01700400	1.70446500
H	-2.24276000	1.63316100	-0.13296600
4	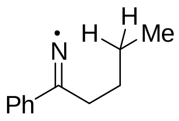	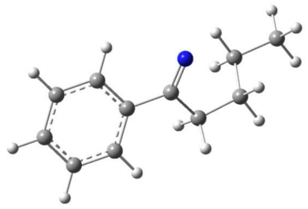	
Cartesian Coordinates			
C	0.26460400	-0.24322400	-0.48604400
N	0.72734400	-1.33799600	-0.91013600
C	1.18283300	0.97216300	-0.30194000
H	0.86949000	1.72033200	-1.04211300
H	0.97053300	1.40622800	0.68406200
C	2.68699400	0.70357800	-0.45047000
H	3.19318600	1.67760600	-0.48980900
H	2.87746900	0.21402500	-1.41329700
C	3.30276600	-0.13090500	0.68182800
H	2.81662400	-1.11231400	0.71703600
C	-1.19879100	-0.10454500	-0.17308000
C	-1.81067900	1.15461200	-0.07418700
C	-1.98190000	-1.25701400	0.01815100
C	-3.17635000	1.25926300	0.20322400
H	-1.23152300	2.06020200	-0.21886800
C	-3.34272700	-1.14955800	0.29744600
H	-1.51065700	-2.23226100	-0.04766800
C	-3.94592000	0.10955600	0.39019800
H	-3.63584700	2.24094700	0.27305900
H	-3.93339100	-2.04867200	0.44716100
H	-5.00637000	0.19239900	0.60969500
H	3.09379700	0.35843100	1.64388800
C	4.81609300	-0.31650000	0.52270200
H	5.33803200	0.64817500	0.51324600
H	5.23198000	-0.91162700	1.34275100
H	5.05371800	-0.83205400	-0.41516500

No.	Species	Optimized Structure
5	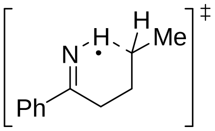	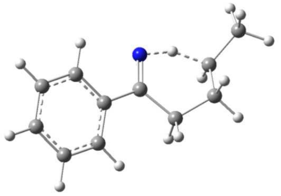

Cartesian Coordinates

C	-0.38902900	-0.06883400	-0.16040200
N	-0.98778400	-1.17168900	-0.39272400
C	-1.14998100	1.25820100	-0.01341300
H	-0.63018400	1.91278700	0.69264600
H	-1.13081600	1.77004800	-0.98660900
C	-2.59921900	1.02810700	0.44178700
H	-3.15122400	1.97971900	0.38731100
H	-2.59679200	0.72954500	1.49883500
C	-3.27486800	-0.05324300	-0.37746500
H	-2.20565100	-0.89848500	-0.43564700
C	1.10729600	-0.05746000	-0.05580000
C	1.84587400	1.13390500	-0.13948200
C	1.80111700	-1.26977700	0.11023600
C	3.24143500	1.11482900	-0.06284200
H	1.34167200	2.08437400	-0.27888600
C	3.19120500	-1.28665800	0.19664100
H	1.23025700	-2.19009800	0.17166300
C	3.91827900	-0.09368900	0.10910000
H	3.79636100	2.04570700	-0.13701700
H	3.71049900	-2.23106800	0.33275200
H	5.00242000	-0.10787900	0.17494300
H	-3.40259300	0.21724300	-1.43195000
C	-4.47136500	-0.75545500	0.22114500
H	-4.23706100	-1.17124800	1.20829000
H	-5.31877400	-0.06379400	0.35313600
H	-4.81809100	-1.57458700	-0.41743400

No.	Species	Optimized Structure
6	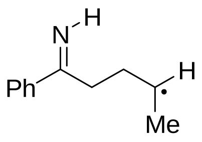	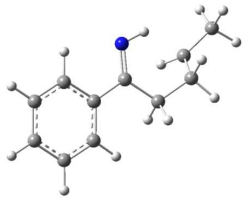

Cartesian Coordinates

C	-0.31013800	0.29523400	-0.19025100
N	-0.76772500	1.49162800	-0.22976300
C	-1.16730900	-0.96640700	-0.26678300

No.	Species	Optimized Structure

H	-0.74851000	-1.61523100	-1.04582900
H	-1.05053500	-1.52203500	0.67446100
C	-2.66190200	-0.75479200	-0.54791600
H	-3.10040300	-1.75103500	-0.74729700
H	-2.79107400	-0.20583600	-1.49450000
C	-3.43054600	-0.08067000	0.54533800
C	1.17296000	0.11422300	-0.06236400
C	1.76618100	-1.15185100	0.07596400
C	2.00926600	1.24597800	-0.07282800
C	3.15222600	-1.28428800	0.20161500
H	1.15620200	-2.04844100	0.09305700
C	3.39017300	1.11400000	0.04864000
H	1.54943200	2.22231900	-0.17693800
C	3.96919300	-0.15311100	0.18696500
H	3.58968600	-2.27260900	0.31062400
H	4.01862500	2.00012500	0.03605400
H	5.04647000	-0.25553200	0.28229500
H	-3.04011400	-0.13472000	1.55998600
C	-4.84863800	0.33974700	0.34206100
H	-5.53624800	-0.52515500	0.32248700
H	-5.19507800	1.00360300	1.14042000
H	-4.98494100	0.85888000	-0.61648600
H	-1.78822500	1.49589500	-0.29263600

No.	Species	Optimized Structure
7	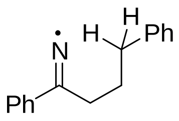	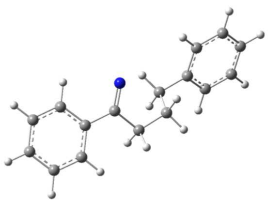

Cartesian Coordinates

C	-1.51634200	0.28988600	0.45079200
N	-0.99905900	1.43386900	0.58018300
C	-0.66065300	-0.97076400	0.63423800
H	-1.03359700	-1.47944400	1.53290200
H	-0.86601900	-1.64295500	-0.20925400
C	0.85010000	-0.73731500	0.77162900
H	1.30968300	-1.68131800	1.08934500
H	1.04165800	-0.01198100	1.57003300
C	1.53572100	-0.26397900	-0.52767800
H	1.08128800	0.67853500	-0.85012600
C	-2.97804300	0.14143000	0.13612900
C	-3.64251200	-1.08428700	0.29653300
C	-3.70662100	1.25375900	-0.32256000

No.	Species	Optimized Structure

C	-5.00613700	-1.19403800	0.01056600
H	-3.10715400	-1.95852900	0.65078200
C	-5.06522200	1.14091100	-0.60873800
H	-3.19503400	2.20127800	-0.45654100
C	-5.72107300	-0.08402600	-0.44252300
H	-5.50658600	-2.14887200	0.14280000
H	-5.61329900	2.00803700	-0.96560100
H	-6.77998500	-0.17159300	-0.66746200
H	1.34481700	-1.00027800	-1.31958300
C	3.02911600	-0.07565800	-0.36289500
C	3.54968300	1.14326400	0.09854800
C	3.92555200	-1.11908000	-0.63738200
C	4.92387500	1.31362900	0.28382500
H	2.87079300	1.96624400	0.30929400
C	5.30128900	-0.95408900	-0.45420700
H	3.54319600	-2.06891600	-1.00515900
C	5.80544900	0.26425600	0.00852700
H	5.30599800	2.26712500	0.63800800
H	5.97802600	-1.77432100	-0.67770900
H	6.87453800	0.39651000	0.14815800

No.	Species	Optimized Structure
8	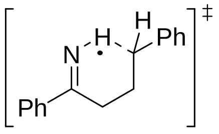	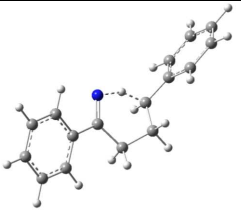

Cartesian Coordinates

C	1.29031300	0.20903400	-0.21096100
N	0.52231000	-0.68603000	-0.69207700
C	0.76300300	1.58809200	0.21993400
H	1.35644900	1.96749400	1.05709600
H	0.91719100	2.28573600	-0.61599600
C	-0.72244600	1.53142300	0.60173700
H	-1.09992000	2.55259900	0.75520800
H	-0.81556400	1.01588200	1.56387000
C	-1.52947700	0.80699800	-0.46519600
H	-0.69087100	-0.16549300	-0.68087800
C	2.75846600	-0.07343700	-0.08547700
C	3.69834000	0.96079800	0.04891600
C	3.21645300	-1.40247200	-0.12460200
C	5.06354800	0.67458000	0.13691200
H	3.37548400	1.99645800	0.07194900
C	4.57666300	-1.68673200	-0.02613200

No.	Species	Optimized Structure

H	2.48986500	-2.20108800	-0.23009600
C	5.50663000	-0.64880100	0.10345500
H	5.77785700	1.48730900	0.23237600
H	4.91367100	-2.71913100	-0.04977300
H	6.56710600	-0.87137800	0.17867600
H	-1.50276000	1.32879900	-1.42809700
C	-2.86801300	0.25562600	-0.17188900
C	-3.83459200	0.19055700	-1.19857400
C	-3.22171300	-0.26475800	1.09099400
C	-5.09656900	-0.35643400	-0.97429500
H	-3.58515200	0.58019900	-2.18253500
C	-4.48484400	-0.81180400	1.31600900
H	-2.50281500	-0.24984700	1.90416500
C	-5.43087300	-0.86043700	0.28710300
H	-5.82122400	-0.38784700	-1.78321800
H	-4.73099200	-1.20456300	2.29873400
H	-6.41383300	-1.28632500	0.46546500

No.	Species	Optimized Structure
9	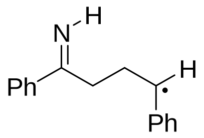	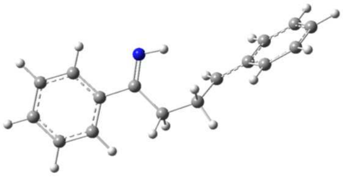

Cartesian Coordinates

C	-1.44999500	-0.01696400	-0.40819900
N	-0.90653500	-1.00768700	-1.01304400
C	-0.70684600	1.23235400	0.06050300
H	-1.19821500	2.10524800	-0.38723800
H	-0.84960900	1.33668200	1.14481700
C	0.79278900	1.28874600	-0.25857600
H	1.14051500	2.31010400	-0.03237700
H	0.94398800	1.17768300	-1.34343300
C	1.62340300	0.29523900	0.50102400
C	-2.92486200	-0.07819500	-0.14587200
C	-3.61307000	0.95020700	0.51968800
C	-3.65530300	-1.20176000	-0.57545100
C	-4.98908400	0.85999400	0.74940200
H	-3.08665700	1.83242100	0.86730000
C	-5.02601100	-1.29135500	-0.34722900
H	-3.12227900	-1.99462900	-1.08795900
C	-5.70045000	-0.25996500	0.31705000
H	-5.50130700	1.66644300	1.26632300
H	-5.57190900	-2.16693600	-0.68722600

No.	Species	Optimized Structure

H	-6.76974500	-0.33054200	0.49500700
H	1.13776800	-0.26075200	1.30062400
C	3.00604600	0.06317700	0.29302200
C	3.76529800	0.75371400	-0.69809300
C	3.69971000	-0.89333900	1.09388200
C	5.12074600	0.49865100	-0.86907900
H	3.28005100	1.49248500	-1.32816800
C	5.05270900	-1.14058000	0.91371900
H	3.14594100	-1.43506300	1.85647600
C	5.77715300	-0.44754400	-0.06925600
H	5.67530800	1.03953900	-1.63103200
H	5.55301400	-1.87581500	1.53780200
H	6.83604100	-0.64253400	-0.20891400
H	0.09944900	-0.87480900	-1.12990800

No.	Species	Optimized Structure
10	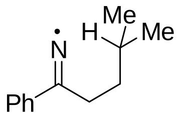	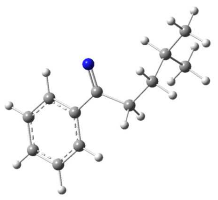

Cartesian Coordinates

C	0.04780100	0.40289900	-0.56054300
N	-0.38145200	1.57447200	-0.75029600
C	-0.88894400	-0.80406300	-0.70116400
H	-0.54739700	-1.36723900	-1.57989300
H	-0.72400000	-1.46007300	0.16151800
C	-2.38052800	-0.47815500	-0.86908100
H	-2.89626300	-1.41678900	-1.11740000
H	-2.50106600	0.18156000	-1.73655100
C	-3.08833900	0.16044600	0.34475000
H	-2.55882800	1.08918100	0.59292000
C	1.49161800	0.16886800	-0.21389000
C	2.08739300	-1.09290300	-0.36432400
C	2.27275900	1.23571200	0.26543800
C	3.43550300	-1.28241200	-0.04843900
H	1.50994300	-1.93296700	-0.73484600
C	3.61588400	1.04330200	0.58183100
H	1.81349900	2.21060400	0.39243900
C	4.20323200	-0.21703700	0.42545300
H	3.88274800	-2.26424400	-0.17366600
H	4.20492700	1.87609300	0.95494200
H	5.24981900	-0.36666700	0.67417100
C	-4.53179800	0.53092200	-0.03068800

No.	Species	Optimized Structure

H	-5.11047300	-0.36279100	-0.29904100
H	-5.04470100	1.01932700	0.80537900
H	-4.55767500	1.21604600	-0.88539300
C	-3.06994900	-0.74955600	1.58344300
H	-2.05293200	-0.96308200	1.92913300
H	-3.60275400	-0.27998300	2.41781100
H	-3.56247000	-1.70841000	1.37383600

No.	Species	Optimized Structure
11	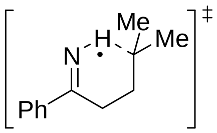	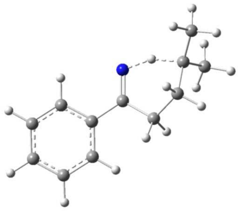

Cartesian Coordinates

C	0.08518900	-0.09675600	-0.01020900
N	0.68576700	-1.19078600	0.25419300
C	0.84606600	1.20607400	-0.30106800
H	0.29923300	1.79781800	-1.04174500
H	0.85870100	1.80476800	0.62046000
C	2.27495500	0.93532600	-0.79957200
H	2.84197700	1.88035900	-0.80530400
H	2.22052200	0.59650600	-1.84169100
C	2.99305900	-0.12497600	0.02841400
H	1.92492200	-0.92321300	0.19109600
C	-1.41536400	-0.06562200	-0.00645500
C	-2.13103500	1.14194600	0.03238400
C	-2.13486100	-1.27423800	-0.02326400
C	-3.52862800	1.14246300	0.05746900
H	-1.60641000	2.09129100	0.05578600
C	-3.52762400	-1.27249600	-0.00775100
H	-1.58168200	-2.20694200	-0.05057300
C	-4.23145100	-0.06322000	0.03401000
H	-4.06492200	2.08640600	0.09441300
H	-4.06719100	-2.21513500	-0.02869600
H	-5.31760600	-0.06269000	0.04715900
C	4.05786200	-0.91052300	-0.71179000
H	3.67516700	-1.32361800	-1.65152800
H	4.91957000	-0.26923100	-0.96019400
H	4.43682100	-1.74023000	-0.10489200
C	3.39826900	0.30730800	1.42722800
H	2.57188000	0.77293700	1.97471600
H	3.74924300	-0.54468300	2.01950700
H	4.22078800	1.03943700	1.38576300

No.	Species	Optimized Structure
12	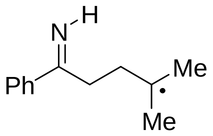	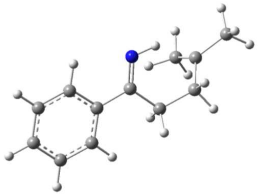

Cartesian Coordinates

C	-0.01988700	0.33594200	-0.31423000
N	-0.47368800	1.53358600	-0.26687900
C	-0.87422700	-0.90703000	-0.54892500
H	-0.42676200	-1.47482800	-1.37427100
H	-0.78828100	-1.55894500	0.33043800
C	-2.35544300	-0.66491000	-0.88339300
H	-2.78504200	-1.65392500	-1.13989800
H	-2.42631200	-0.08089200	-1.81176600
C	-3.21343600	-0.02180900	0.17520900
C	1.45489900	0.13496200	-0.12820200
C	2.04275200	-1.14095500	-0.10358200
C	2.28808900	1.25712400	0.03626800
C	3.42030800	-1.29234100	0.08057200
H	1.43497500	-2.03111500	-0.22400500
C	3.66062900	1.10669300	0.21674900
H	1.83236300	2.24078700	0.01976800
C	4.23424700	-0.17019600	0.24044700
H	3.85353000	-2.28835300	0.09833200
H	4.28669700	1.98596900	0.34022700
H	5.30494200	-0.28713000	0.38202300
C	-4.54033500	0.52496400	-0.25889800
H	-5.29669300	-0.27603100	-0.36378500
H	-4.94289200	1.23862300	0.46929900
H	-4.47996900	1.02620500	-1.23220700
C	-3.05025700	-0.41709800	1.61196300
H	-2.00467600	-0.40222700	1.93842600
H	-3.61382100	0.24981100	2.27306000
H	-3.42588700	-1.44127700	1.79791400
H	-1.49038900	1.54614800	-0.37927000

No.	Species	Optimized Structure
13	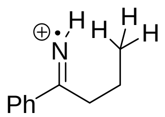	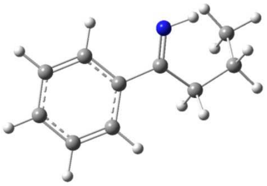

Cartesian Coordinates

No.	Species	Optimized Structure

C	0.75245700	-0.22014500	-0.36600400
N	1.07119800	-1.41169900	-0.76150800
C	1.73024700	0.92666900	-0.20254000
H	1.51127400	1.63748300	-1.01417800
H	1.48024800	1.44322400	0.73264500
C	3.22308500	0.56242800	-0.22386500
H	3.77653400	1.50728600	-0.22761200
H	3.48284700	0.07724600	-1.17432000
C	3.68181700	-0.29006800	0.96580900
H	3.18536700	-1.26725600	1.00048900
C	-0.68323700	-0.05283100	-0.11627300
C	-1.29833000	1.22503000	-0.12552500
C	-1.49059400	-1.22814300	0.08111600
C	-2.66229400	1.32690700	0.06666500
H	-0.70761700	2.11861600	-0.29106700
C	-2.85779200	-1.11216300	0.26862200
H	-0.99578000	-2.19117300	0.12737600
C	-3.44820700	0.15797500	0.26210600
H	-3.14288300	2.29970800	0.06717000
H	-3.46337400	-1.99579100	0.43827300
H	-4.51825600	0.25899300	0.41763000
H	4.75810700	-0.47431200	0.91244600
H	3.47961600	0.21734600	1.91515700
H	1.97261600	-1.52342600	-1.23411400

14	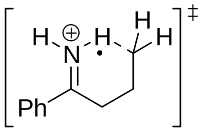	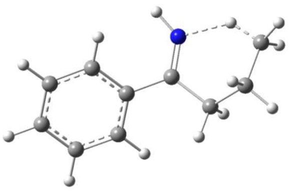

Cartesian Coordinates

C	0.79567300	0.12210200	-0.23517200
N	1.31392100	1.17918000	-0.73200200
C	1.70202600	-1.04935500	0.10352700
H	1.16148000	-1.73510600	0.75805100
H	1.90508400	-1.57899500	-0.83830100
C	3.02109100	-0.58986700	0.75393600
H	3.63141300	-1.48566900	0.93300300
H	2.81053900	-0.15660000	1.73788600
C	3.79198200	0.39245000	-0.10644600
H	3.04089600	1.18612800	-0.45611900
C	-0.67438600	0.05012700	-0.08566400
C	-1.34431700	-1.16305400	-0.34518600

No.	Species	Optimized Structure
C	-1.40982900 1.20172900 0.27862500	
C	-2.72877500 -1.22035000 -0.22909500	
H	-0.79145200 -2.04742000 -0.64340100	
C	-2.79261000 1.12627100 0.40840400	
H	-0.89866600 2.12856600 0.52090800	
C	-3.45346300 -0.07878700 0.14564900	
H	-3.24667700 -2.15379200 -0.42326000	
H	-3.35408800 2.00089000 0.71945500	
H	-4.53338600 -0.13401400 0.23981600	
H	4.57218700 0.94049500 0.42842300	
H	4.18331100 -0.04020900 -1.03212200	
H	0.87756100 2.07386700 -0.95179800	
15	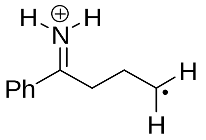	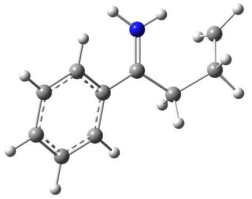
Cartesian Coordinates		
C	0.76374100 0.24244800 -0.08302200	
N	1.30353700 1.41173300 -0.32555300	
C	1.69364200 -0.93109500 0.11226300	
H	1.21661500 -1.63097500 0.80150600	
H	1.75934200 -1.44917800 -0.85687600	
C	3.11530600 -0.59998200 0.62007200	
H	3.58884400 -1.57067800 0.84737600	
H	3.05435500 -0.08271700 1.58581500	
C	3.98903200 0.15265600 -0.33165400	
C	-0.68456800 0.09451500 -0.02404700	
C	-1.27054400 -1.17675800 -0.21684300	
C	-1.52512800 1.20865400 0.20952200	
C	-2.65420100 -1.32026600 -0.20775800	
H	-0.65197600 -2.04700900 -0.40505100	
C	-2.90488700 1.05343400 0.23799000	
H	-1.10890900 2.18860700 0.42517300	
C	-3.47204600 -0.20910400 0.02091200	
H	-3.09522800 -2.29703300 -0.37560400	
H	-3.54030400 1.90927300 0.43955000	
H	-4.55100600 -0.32632100 0.04021200	
H	4.84494200 0.70360100 0.04565400	
H	3.98819400 -0.09842200 -1.39029700	
H	0.74518300 2.22994400 -0.53980800	
H	2.32311300 1.50177000 -0.36338300	

No.	Species	Optimized Structure
16	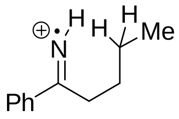	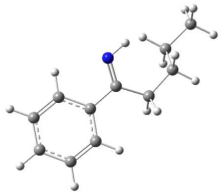

Cartesian Coordinates

C	-0.24277200	-0.13855800	0.43164800
N	-0.59758400	-1.30107900	0.88093600
C	-1.17858100	1.04127900	0.25841300
H	-0.86690300	1.79359400	0.99892500
H	-0.97317800	1.48062000	-0.72614300
C	-2.67966600	0.75741300	0.41268400
H	-3.18880300	1.72765900	0.38417900
H	-2.89167300	0.35460000	1.41339100
C	-3.27570300	-0.15379500	-0.67176700
C	1.18769100	-0.04005300	0.12426300
C	1.85228500	1.21089300	0.05897100
C	1.94040000	-1.25304300	-0.05384800
C	3.21128300	1.25023500	-0.18613500
H	1.30408800	2.13369700	0.20923600
C	3.30341400	-1.19966400	-0.29430400
H	1.40775700	-2.19677400	-0.04338600
C	3.94299900	0.04456500	-0.36076900
H	3.72848100	2.20232000	-0.24367300
H	3.86722300	-2.11325600	-0.44859600
H	5.00976300	0.09562300	-0.55762900
H	-3.05700700	0.27513800	-1.65872100
C	-4.78768800	-0.34675200	-0.51781400
H	-5.03865100	-0.80005100	0.44767700
H	-5.31562600	0.61084400	-0.58429800
H	-5.18106100	-0.99781000	-1.30350100
H	-2.78192300	-1.13747900	-0.65697500
H	-1.48136500	-1.35629500	1.39490300

No.	Species	Optimized Structure
17	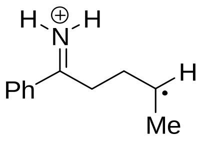	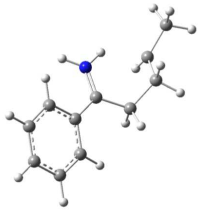

Cartesian Coordinates

C	0.27736900	0.13679400	-0.13533200
N	0.88198100	1.26577300	-0.41100200

C	1.14412000	-1.08398000	0.05749600
H	0.63058700	-1.76369600	0.74010400
H	1.19070500	-1.59858100	-0.91475200
C	2.57715000	-0.82287600	0.57346200
H	3.00192400	-1.81488100	0.80640400
H	2.53305000	-0.30144600	1.53960900
C	3.49639600	-0.10252600	-0.37041700
C	-1.17574900	0.07699300	-0.03993800
C	-1.84122500	-1.15785600	-0.20794900
C	-1.94185900	1.24127000	0.20303900
C	-3.23053100	-1.21779400	-0.16500600
H	-1.28041600	-2.06494200	-0.40331500
C	-3.32750300	1.16953400	0.26620400
H	-1.46176600	2.19586400	0.39881500
C	-3.97452200	-0.05805100	0.07387300
H	-3.73322800	-2.16739700	-0.31447900
H	-3.90529600	2.06356800	0.47532600
H	-5.05777800	-0.11004800	0.11990900
H	3.41652400	-0.35149900	-1.43033800
H	0.37162100	2.11519300	-0.62347400
H	1.90777200	1.28341600	-0.46476300
C	4.79067400	0.46188200	0.11358400
H	5.22871600	1.15533700	-0.61029800
H	4.68372400	0.97731500	1.07507000
H	5.53407600	-0.33894900	0.26909700

No.	Species	Optimized Structure
18	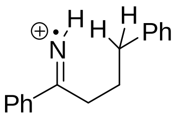	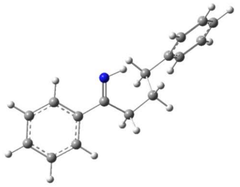

Cartesian Coordinates

C	-1.52257600	0.42295200	0.53113800
N	-1.13112900	1.64814600	0.40632500
C	-0.66509800	-0.70854600	1.08015100
H	-1.00697300	-0.91514800	2.10454400
H	-0.87150300	-1.61745500	0.50386100
C	0.85220700	-0.46772500	1.11603400
H	1.31463700	-1.32825100	1.61037000
H	1.09507900	0.39900500	1.74296800
C	1.47116900	-0.30388400	-0.29619900
C	-2.91804900	0.14434300	0.12293300
C	-3.57529000	-1.05663500	0.46925600

No.	Species	Optimized Structure

C	-3.63369500	1.13765700	-0.61164200
C	-4.89533900	-1.26190700	0.09495800
H	-3.06327000	-1.82086200	1.04254400
C	-4.95104400	0.92381300	-0.98898600
H	-3.11662200	2.05271100	-0.87484300
C	-5.58868400	-0.27279500	-0.63851600
H	-5.40106000	-2.18228800	0.36808300
H	-5.48391400	1.67971500	-1.55604500
H	-6.62044700	-0.44566100	-0.92883000
H	1.19199500	-1.16494400	-0.91256700
C	2.96259900	-0.14883000	-0.29144200
C	3.79721300	-1.14911200	-0.84942300
C	3.56948600	1.00510300	0.27608300
C	5.17638600	-1.01458500	-0.82623300
H	3.34421600	-2.02988400	-1.29484200
C	4.94691400	1.14409300	0.29708100
H	2.94336900	1.79172000	0.68737500
C	5.76056700	0.13349500	-0.25253500
H	5.80888200	-1.78760800	-1.25027600
H	5.40193400	2.03022300	0.72722400
H	6.84057300	0.24215500	-0.24070700
H	1.02415800	0.58427800	-0.77346100
H	-0.20375300	1.81065000	0.80436800

19	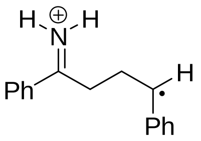	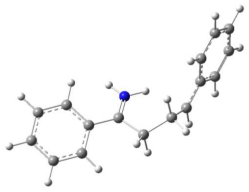

Cartesian Coordinates

C	1.30205100	0.22657700	-0.17223500
N	0.46065400	-0.65913100	-0.64499500
C	0.74740100	1.56579700	0.24755200
H	1.40469600	1.98608400	1.01112300
H	0.81986200	2.23173000	-0.62595400
C	-0.71213000	1.56106700	0.77194700
H	-0.88511700	2.57005100	1.17626200
H	-0.77945500	0.88443200	1.62985000
C	-1.74968700	1.24968000	-0.26859100
C	2.72420500	-0.08487900	-0.07854800
C	3.67290800	0.96068100	-0.04109700
C	3.17809400	-1.42401000	-0.04346200
C	5.03375000	0.67253100	-0.00173000

No.	Species	Optimized Structure

H	3.35345500	1.99614300	-0.07520300
C	4.53743000	-1.70357300	0.01738400
H	2.47493500	-2.25140400	-0.00786600
C	5.46771200	-0.65630900	0.02971900
H	5.75525600	1.48258300	0.00719600
H	4.87505000	-2.73339700	0.06630100
H	6.52941400	-0.87798500	0.07341700
H	0.76901200	-1.55476900	-1.00509300
H	-0.53937400	-0.43464800	-0.68726800
C	-2.91733200	0.46223600	-0.09049900
C	-3.23095200	-0.23093800	1.11933400
C	-3.84316000	0.33787600	-1.17409700
C	-4.38647200	-0.99278500	1.22520600
H	-2.57445300	-0.15211500	1.97984800
C	-4.99331400	-0.42606000	-1.05484000
H	-3.63723100	0.86494900	-2.10208100
C	-5.27451000	-1.10011500	0.14411200
H	-4.60769500	-1.50291500	2.15780700
H	-5.68139500	-0.49793000	-1.89145700
H	-6.17697700	-1.69545600	0.23731400
H	-1.66051300	1.79190400	-1.21016600

No.	Species	Optimized Structure
20	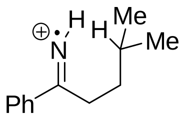	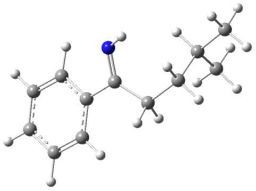

Cartesian Coordinates

C	0.05280500	0.24575900	-0.55736200
N	-0.28199500	1.47111300	-0.81817700
C	-0.89306700	-0.93602700	-0.64219900
H	-0.55201300	-1.54102500	-1.49636600
H	-0.72980700	-1.55363100	0.24802700
C	-2.38285100	-0.60811900	-0.82127000
H	-2.90450400	-1.56538600	-0.94340300
H	-2.53269400	-0.07734000	-1.77194000
C	-3.05250400	0.17914600	0.32663700
C	1.46254400	0.08236800	-0.19211300
C	2.11814600	-1.17107800	-0.29133400
C	2.20737600	1.24055600	0.21921600
C	3.46133700	-1.26628000	0.01920600
H	1.57658900	-2.05125100	-0.61821900
C	3.55453700	1.13238900	0.52274700

No.	Species	Optimized Structure

H	1.67990400	2.18057200	0.33181700
C	4.18541900	-0.11425800	0.42437200
H	3.97062300	-2.22195400	-0.04814300
H	4.11176300	2.00251600	0.85252600
H	5.23913300	-0.20887400	0.66938100
C	-4.50278600	0.51931800	-0.04715800
H	-4.55996400	1.08071900	-0.98621800
H	-5.09862700	-0.39353000	-0.16687200
H	-4.97608000	1.12311300	0.73276500
H	-2.51228100	1.13478500	0.45258100
C	-2.98817100	-0.56426500	1.66947900
H	-1.96213400	-0.73544900	2.01359600
H	-3.49610100	0.00926800	2.45039200
H	-3.48717100	-1.53876200	1.59857800
H	-1.12939100	1.62138300	-1.37258600

No.	Species	Optimized Structure
21	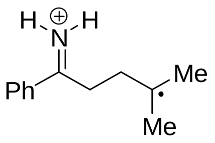	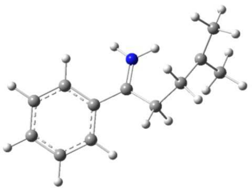

Cartesian Coordinates

C	0.02605900	0.13201100	0.07045200
N	-0.58634000	1.21101000	0.49156300
C	-0.84056900	-1.03560100	-0.32828400
H	-0.29382400	-1.64233400	-1.05208700
H	-0.96610100	-1.66231800	0.56658900
C	-2.22498600	-0.67234100	-0.92366700
H	-2.65666900	-1.62672000	-1.27204500
H	-2.07251800	-0.07411900	-1.82999600
C	-3.22146100	0.01416900	-0.01693400
C	1.48205900	0.07674400	0.01745200
C	2.14021300	-1.17315700	0.02039900
C	2.25763400	1.25925300	-0.01803800
C	3.53030400	-1.23449300	0.01836800
H	1.57203900	-2.09579600	0.05639700
C	3.64461400	1.18970900	-0.04042300
H	1.78515300	2.23531900	-0.08264700
C	4.28347200	-0.05683000	-0.01395800
H	4.02645800	-2.19897300	0.03932200
H	4.23022500	2.10161300	-0.08929800
H	5.36765200	-0.10804300	-0.02784600
H	-0.08314000	2.01989600	0.83737600

No.	Species	Optimized Structure		
H		-1.61585200	1.23389000	0.47684400
C		-4.27351100	0.85802300	-0.67504900
H		-4.76858300	1.52219000	0.04085700
H		-3.87298600	1.46264400	-1.49605700
H		-5.06662400	0.22463900	-1.11003100
C		-3.55367000	-0.60858100	1.30897600
H		-4.20331800	-1.49042000	1.17066700
H		-2.67459000	-0.95724800	1.86327600
H		-4.10388300	0.08526300	1.95198000

7.4 Bond Dissociation Enthalpies

DFT Methods:

Structure optimization: (RO)B3P86/6-311G(d,p)

Frequency calculations: (RO)B3P86/6-311G(d,p) scaled by a factor of 0.9806

No.	Reactions	Kcal mol^{-1}
1	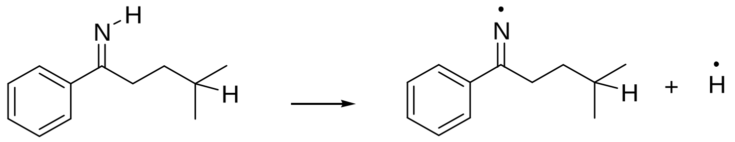	93.2
2	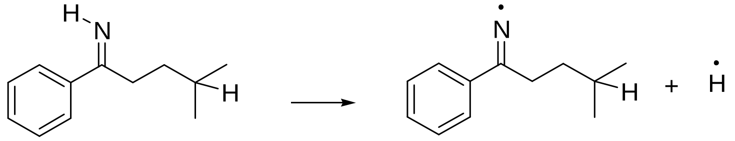	92.3
3	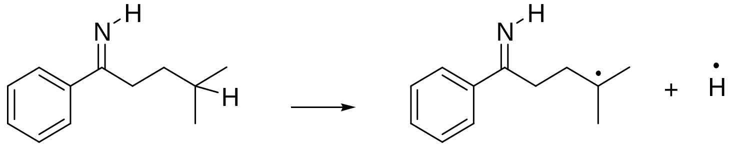	95.6
4	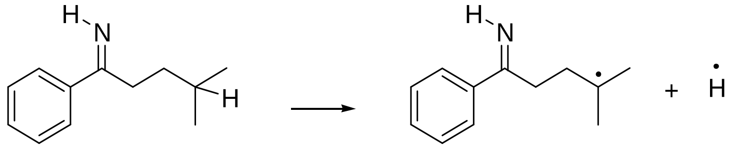	95.2
5	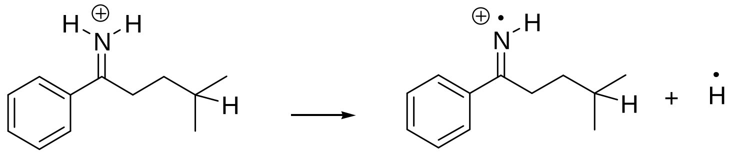	120.9
6	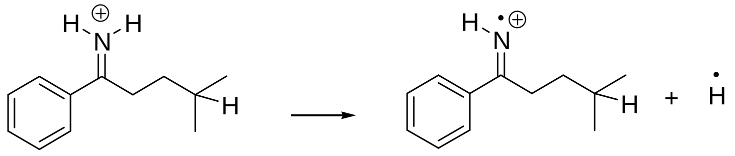	121.2
7	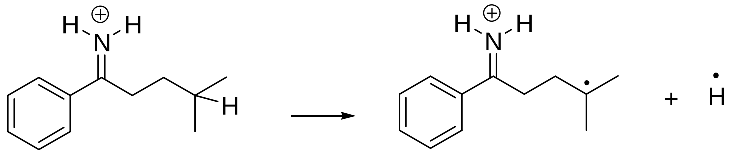	92.5
8	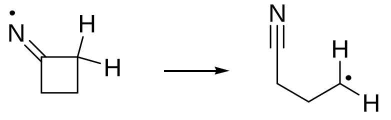	-6.8

No.	Reactions	Kcal mol^{-1}
9	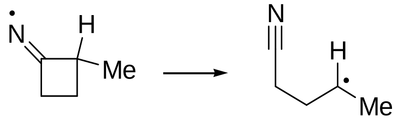	-8.3
10	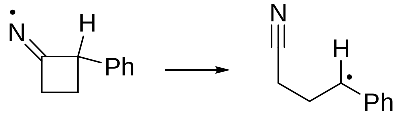	-18.7
11	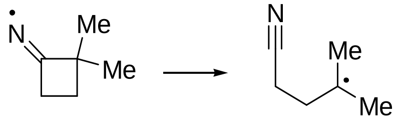	-10.2
12	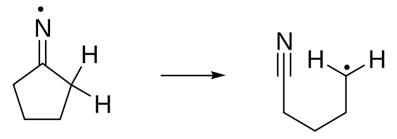	12.0
13	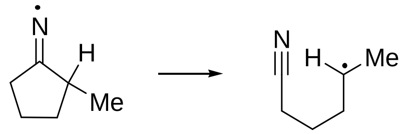	10.0
14	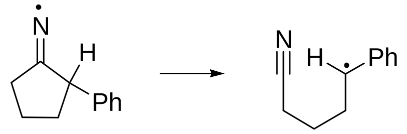	-0.9
15	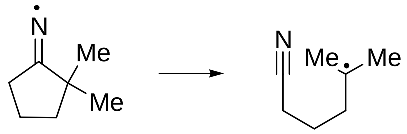	7.9
16	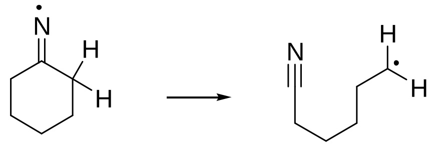	18.6
17	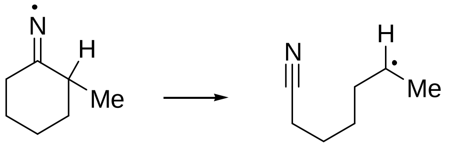	15.0
18	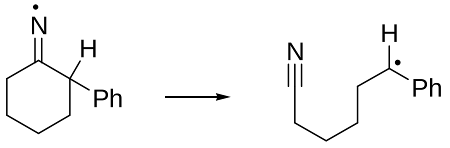	3.7

No.	Reactions	Kcal mol^{-1}
19	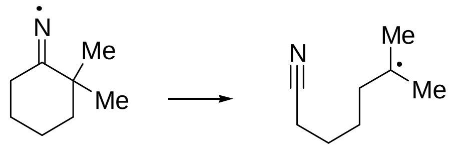	12.8
20	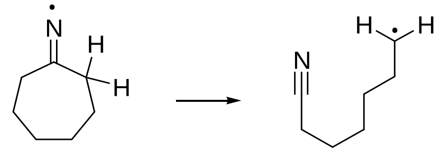	14.6
21	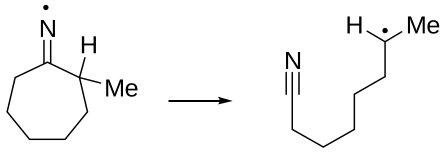	10.8
22	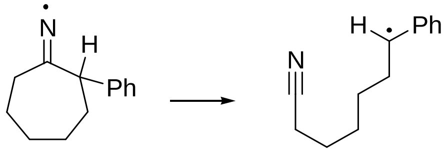	-1.2
23	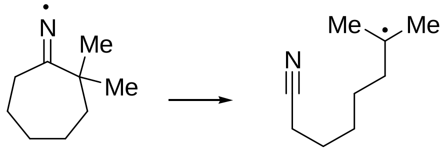	9.0
24	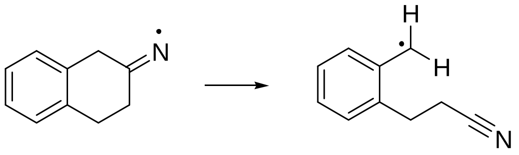	0.6
25	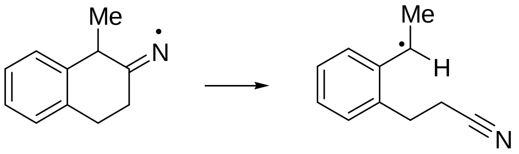	3.5
26	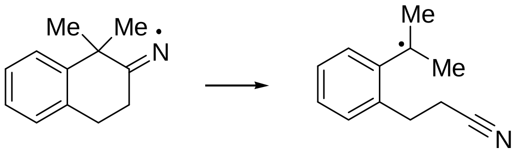	6.3

Computed Energies [values are in Hartree]

No.	Species	Total Electronic Energy	Sum of Electronic and Zero-point Energies	Sum of Electronic and Thermal Enthalpies	Gibbs Free Energy
1	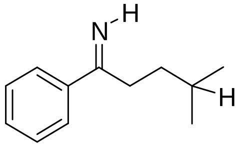	-524.1354695	-523.876405	-523.862063	-523.917814
2	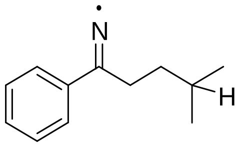	-523.4576186	-523.211808	-523.197320	-523.255216
3	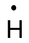	-0.5185156	-0.518516	-0.516155	-0.529170
4	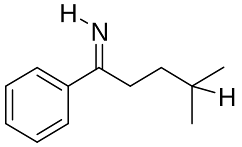	-524.1336755	-523.875000	-523.860488	-523.916784
5	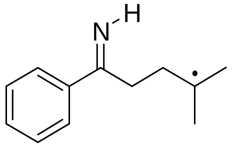	-523.4530609	-523.208598	-523.193598	-523.252112
6	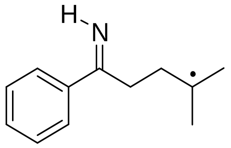	-523.4519284	-523.207782	-523.192637	-523.252061
7	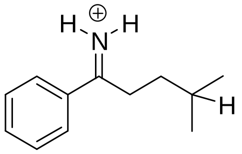	-524.5252933	-524.253414	-524.239606	-524.293228
8	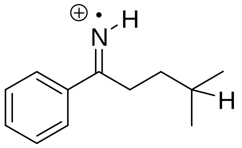	-523.8014873	-523.545696	-523.530734	-523.588419
9	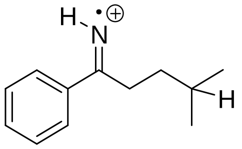	-523.8013617	-523.545175	-523.530245	-523.587838

No.	Species	Total Electronic Energy	Sum of Electronic and Zero-point Energies	Sum of Electronic and Thermal Enthalpies	Gibbs Free Energy
10	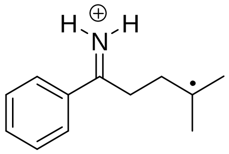	-523.8488571	-523.591406	-523.576081	-523.635315
11	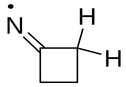	-211.4147367	-211.326279	-211.321084	-211.353832
12	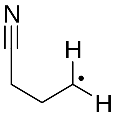	-211.4249726	-211.338956	-211.332400	-211.368226
13	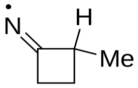	-250.8892424	-250.772918	-250.765575	-250.803372
14	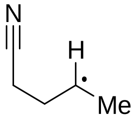	-250.9017145	-250.787437	-250.778761	-250.820570
15	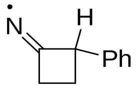	-443.2395939	-443.071085	-443.060712	-443.107319
16	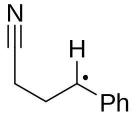	-443.2695386	-443.101734	-443.090543	-443.140367
17	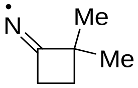	-290.3623497	-290.218973	-290.210169	-290.251145
18	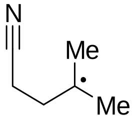	-290.3782924	-290.236530	-290.226381	-290.271731
19	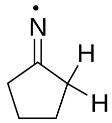	-250.9183288	-250.799789	-250.793126	-250.829630
20	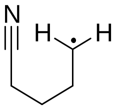	-250.8967484	-250.782526	-250.774077	-250.814675

No.	Species	Total Electronic Energy	Sum of Electronic and Zero-point Energies	Sum of Electronic and Thermal Enthalpies	Gibbs Free Energy
21	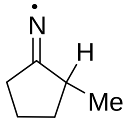	-290.3894344	-290.243322	-290.235243	-290.275064
22	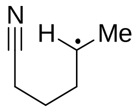	-290.3714698	-290.229160	-290.219294	-290.263671
23	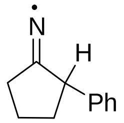	-482.7382544	-482.539866	-482.528750	-482.577114
24	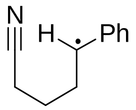	-482.7384872	-482.542664	-482.530216	-482.582598
25	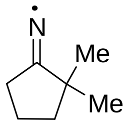	-329.8618548	-329.688780	-329.679266	-329.721985
26	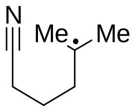	-329.8478144	-329.678085	-329.666715	-329.714781
27	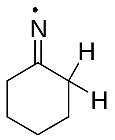	-290.3955297	-290.248013	-290.240449	-290.278884
28	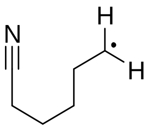	-290.3626814	-290.220452	-290.210740	-290.254345
29	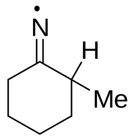	-329.8652279	-329.690137	-329.681118	-329.722760
30	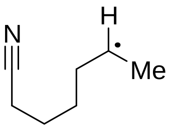	-329.8385404	-329.668335	-329.657144	-329.705004

No.	Species	Total Electronic Energy	Sum of Electronic and Zero-point Energies	Sum of Electronic and Thermal Enthalpies	Gibbs Free Energy
31	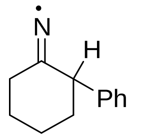	-522.2150475	-521.987578	-521.975551	-522.025364
32	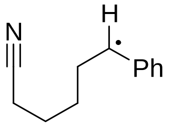	-522.2072852	-521.983313	-521.969633	-522.024513
33	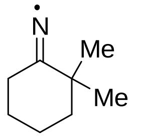	-369.3367271	-369.134686	-369.124306	-369.168603
34	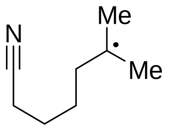	-369.3146039	-369.116407	-369.103968	-369.154482
35	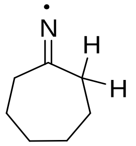	-329.8585988	-329.683115	-329.674234	-329.716155
36	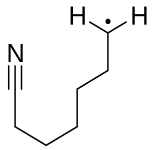	-329.832049	-329.661920	-329.650930	-329.698222
37	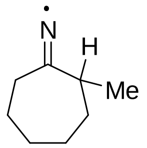	-369.3288049	-369.125761	-369.115516	-369.160455
38	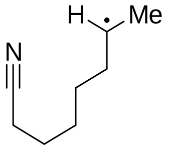	-369.3088806	-369.110835	-369.098369	-369.149581
39	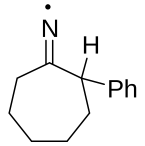	-561.676313	-561.421394	-561.407889	-561.461807

No.	Species	Total Electronic Energy	Sum of Electronic and Zero-point Energies	Sum of Electronic and Thermal Enthalpies	Gibbs Free Energy
40	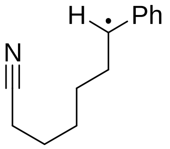	-561.6763643	-561.424871	-561.409748	-561.468555
41	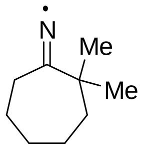	-408.8002562	-408.569941	-408.558383	-408.605425
42	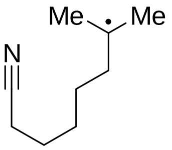	-408.7837159	-408.557974	-408.544051	-408.598914
43	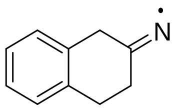	-443.2666946	-443.096752	-443.088263	-443.129741
44	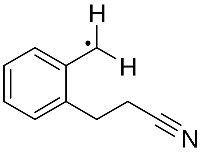	-443.2656329	-443.098393	-443.087258	-443.135158
45	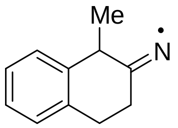	-482.7476837	-482.549714	-482.538412	-482.586467
46	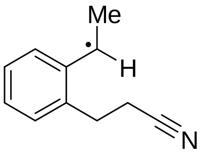	-482.7405199	-482.545878	-482.532913	-482.585587
47	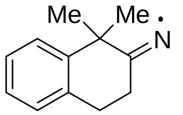	-522.2176861	-521.992566	-521.979893	-522.030121
48	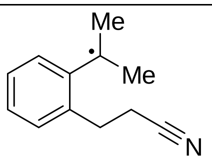	-522.2064323	-521.984148	-521.969853	-522.024947

Optimized Structures and Cartesian Coordinates

No.	Species	Optimized Structure
1	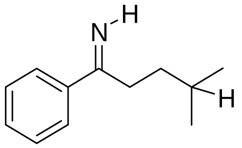	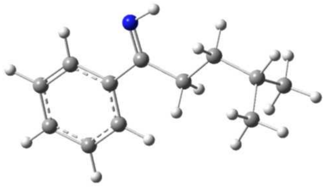

Cartesian Coordinates

C	-0.23464200	0.96528500	0.00002300
C	-1.55110900	0.26392400	0.00001000
C	-2.72640600	1.02315800	-0.00000800
C	-1.65650900	-1.12890200	0.00000800
C	-3.96677100	0.40757800	-0.00002700
H	-2.63081400	2.10204500	-0.00000800
C	-2.90200000	-1.74716900	-0.00001000
H	-0.76665100	-1.74691800	0.00002200
C	-4.05997600	-0.98190400	-0.00002700
H	-4.86766200	1.01135700	-0.00003400
H	-2.96491000	-2.82969400	-0.00001000
H	-5.03144000	-1.46383500	-0.00003900
N	-0.24231000	2.23954400	0.00003500
C	1.01218300	0.10401100	0.00003800
H	0.96129200	-0.55727700	0.87268700
H	0.96132400	-0.55723300	-0.87265900
H	0.70648900	2.61156700	0.00007000
C	2.32709200	0.87112200	0.00005500
H	2.36162600	1.52697600	-0.87879600
H	2.36165800	1.52693900	0.87892700
C	3.59467300	0.00258500	0.00001200
H	4.43756200	0.70531200	0.00007200
C	3.71844200	-0.85205000	1.25978400
C	3.71846700	-0.85187200	-1.25987500
H	4.68629700	-1.35973200	1.29092700
H	3.63110800	-0.24483100	2.16546300
H	2.94606700	-1.62608900	1.30000700
H	4.68629700	-1.35960300	-1.29104500
H	2.94605700	-1.62587200	-1.30025400
H	3.63120800	-0.24451800	-2.16546900

No.	Species	Optimized Structure
2	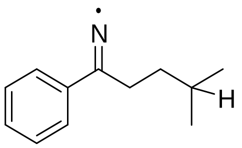	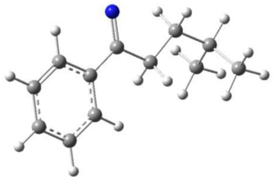

No.	Species	Optimized Structure
Cartesian Coordinates		
C 0.22703500 0.94627800 -0.00004000		
C 1.56453500 0.28031200 0.00003800		
C 2.72493000 1.06222600 -0.00012500		
C 1.68786600 -1.10941900 0.00025600		
C 3.97478800 0.46593400 -0.00010100		
H 2.62894000 2.14173100 -0.00027000		
C 2.94392300 -1.70580200 0.00028700		
H 0.80543800 -1.73753100 0.00040100		
C 4.08914000 -0.92166000 0.00010000		
H 4.86540300 1.08430700 -0.00023900		
H 3.02433000 -2.78702900 0.00044900		
H 5.06810500 -1.38761200 0.00012100		
N 0.13223000 2.19694500 -0.00012800		
C -1.02891200 0.08987900 -0.00007200		
H -0.97716100 -0.56494100 -0.87724200		
H -0.97740100 -0.56463200 0.87736000		
C -2.32455900 0.89321200 -0.00046400		
H -2.32987100 1.55364500 0.87273100		
H -2.32968300 1.55298300 -0.87416500		
C -3.60801400 0.05126700 -0.00026600		
C -3.75122600 -0.80130800 -1.25966900		
C -3.75178900 -0.79973200 1.26013400		
H -4.72872200 -1.29043300 -1.29029900		
H -3.65267500 -0.19542000 -2.16504500		
H -2.99480500 -1.59144400 -1.30201600		
H -4.72929900 -1.28881500 1.29094100		
H -2.99539400 -1.58983000 1.30381700		
H -3.65363700 -0.19270000 2.16478400		
H -4.43548300 0.77199000 -0.00089900		
3	Ḣ	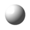
Cartesian Coordinates		
H 0.00000000 0.00000000 0.00000000		
4	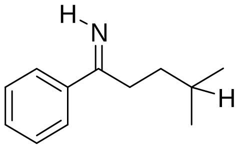	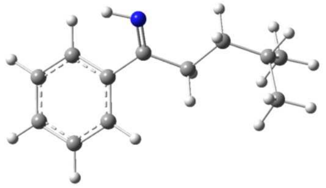
Cartesian Coordinates		
C -0.20901000 0.90359800 0.32241200		
C -1.53662000 0.25162600 0.11879800		
C -2.60257700 0.99511200 -0.39722600		
C -1.75584200 -1.09045500 0.43962000		

No.	Species	Optimized Structure

C	-3.85203900	0.41947200	-0.57643200
H	-2.44005800	2.02855400	-0.68545900
C	-3.01033300	-1.66373300	0.27496000
H	-0.94706300	-1.69023300	0.84003200
C	-4.06166300	-0.91202100	-0.23501500
H	-4.66250100	1.00913600	-0.99015000
H	-3.16631800	-2.70204800	0.54573600
H	-5.03836200	-1.36244800	-0.37122900
N	-0.06808400	2.11603600	0.68722800
C	1.01845000	0.06930800	0.05122500
H	1.00275900	-0.79223100	0.73047400
H	0.91497700	-0.35814400	-0.95364900
C	2.32908300	0.82916100	0.19424100
H	2.30243300	1.69760800	-0.47144900
H	2.38365000	1.24318000	1.20556000
C	3.59373400	0.00780500	-0.09153800
H	4.43584100	0.69639400	0.05567800
C	3.77993500	-1.14748200	0.89058400
C	3.66180600	-0.48454300	-1.53616800
H	4.74889800	-1.63329500	0.74444200
H	3.73319600	-0.80073200	1.92709000
H	3.01180900	-1.91662100	0.75970600
H	4.62637400	-0.95641900	-1.74398000
H	2.88679400	-1.22841300	-1.74676600
H	3.53421300	0.33993900	-2.24368600
H	-0.97960700	2.54643500	0.85428000

No.	Species	Optimized Structure
5	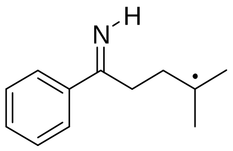	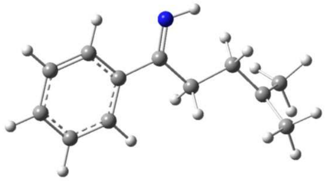

Cartesian Coordinates

C	-0.25125000	0.87112800	0.07712000
C	-1.60020100	0.23906300	0.00571100
C	-2.73732400	1.04740000	0.11191500
C	-1.77222600	-1.13662800	-0.16446700
C	-4.00585500	0.49559000	0.04993400
H	-2.59002300	2.11246200	0.24281800
C	-3.04604000	-1.69059300	-0.22692500
H	-0.91305000	-1.79099900	-0.25116300
C	-4.16579400	-0.87737500	-0.11998000
H	-4.87663600	1.13652500	0.13406900
H	-3.16104100	-2.76064900	-0.35941300

No.	Species	Optimized Structure

H	-5.15934000	-1.30926300	-0.16839600
N	-0.19494400	2.13537700	0.22749500
C	0.95178000	-0.04220700	-0.03729300
H	0.88472000	-0.79870300	0.75306500
H	0.87954700	-0.59771100	-0.97941000
C	2.31302300	0.65467900	0.03084100
H	2.38209900	1.38613800	-0.78205300
H	2.36732800	1.22686500	0.97178700
C	3.46668800	-0.29525000	-0.04980300
C	3.79306300	-1.13151400	1.13848200
C	4.50659000	-0.10328100	-1.09673000
H	4.47137500	-0.60440400	1.83093000
H	2.90371400	-1.39788900	1.71685800
H	4.30041300	-2.05933100	0.85531700
H	5.06740200	-1.02436700	-1.28350000
H	4.07318600	0.23016600	-2.04439500
H	5.24899600	0.65953300	-0.80496800
H	0.77118500	2.45791900	0.26315500

6	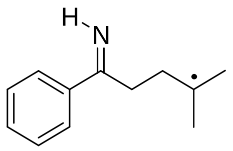	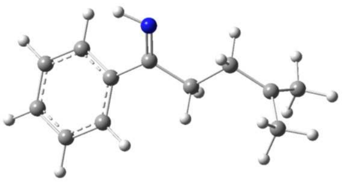

Cartesian Coordinates

C	-0.20784000	0.85995200	0.33617400
C	-1.54526400	0.23273200	0.12080700
C	-2.60520600	1.00589300	-0.36303400
C	-1.77819600	-1.11702500	0.39691900
C	-3.86218600	0.45093900	-0.55413400
H	-2.43269500	2.04656800	-0.61749100
C	-3.04011200	-1.66994500	0.22034300
H	-0.97368000	-1.73927000	0.77075900
C	-4.08539600	-0.88903400	-0.25719500
H	-4.66807400	1.06350300	-0.94274700
H	-3.20658400	-2.71513200	0.45610700
H	-5.06802900	-1.32322800	-0.40305000
N	-0.04428200	2.06065800	0.72852800
C	1.00742800	0.01519200	0.04368700
H	1.00170400	-0.85224900	0.71593400
H	0.90183100	-0.40148600	-0.96555100
C	2.34198400	0.76191500	0.18102500
H	2.31569800	1.62968700	-0.48638200
H	2.40236400	1.16561500	1.19660800

No.	Species	Optimized Structure

C	3.52715000	-0.09422300	-0.11816800
C	4.04771600	-1.01745900	0.92820800
C	3.92161600	-0.33189000	-1.53444200
H	5.11625200	-1.21806600	0.79279900
H	3.90292400	-0.61132700	1.93365800
H	3.54958100	-2.00236900	0.90745300
H	4.98997200	-0.55781100	-1.62282200
H	3.39111800	-1.19063300	-1.98101700
H	3.70409900	0.53519300	-2.16519100
H	-0.94667800	2.50411800	0.91010700

No.	Species	Optimized Structure
7	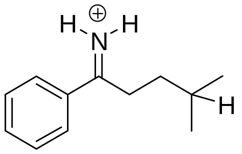	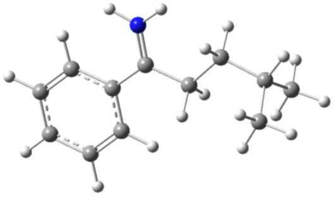

Cartesian Coordinates

C	0.24107200	0.87975000	-0.00001500
C	1.54814900	0.24802700	-0.00000300
C	2.73592200	1.00333700	-0.00002300
C	1.64088800	-1.15431600	0.00002800
C	3.96645300	0.37724900	-0.00001200
H	2.72379900	2.08793200	-0.00004900
C	2.87630500	-1.77739900	0.00004000
H	0.74738600	-1.76476700	0.00004500
C	4.03901200	-1.01472800	0.00001900
H	4.87312800	0.96938700	-0.00002800
H	2.93386100	-2.85877100	0.00006400
H	5.00640300	-1.50332000	0.00002700
N	0.12687400	2.17932000	-0.00003700
C	-1.02083100	0.07925300	-0.00000400
H	-0.97485300	-0.58361700	-0.87157000
H	-0.97486600	-0.58357100	0.87159900
H	-0.78948900	2.60909000	-0.00004700
C	-2.32880100	0.86412000	-0.00003700
H	-2.37202500	1.50991500	0.88676200
H	-2.37201100	1.50985500	-0.88688100
C	-3.58862000	-0.02056300	-0.00001400
H	-4.43041700	0.68086100	-0.00006200
C	-3.70049900	-0.87048100	-1.26329200
C	-3.70053800	-0.87035300	1.26334700
H	-4.66633400	-1.37864800	-1.29512000
H	-3.61867000	-0.26424700	-2.16985100
H	-2.93364800	-1.65104900	-1.30423800

No.	Species	Optimized Structure

H	-4.66637100	-1.37852400	1.29519200
H	-2.93368400	-1.65091100	1.30440300
H	-3.61874800	-0.26402500	2.16984700
H	0.92736000	2.79579300	-0.00004300

8	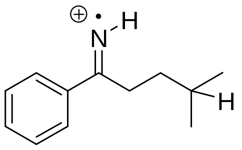	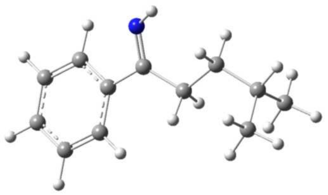

Cartesian Coordinates

C	0.22271100	0.80314000	-0.19521900
N	0.27179800	2.04421700	-0.51158700
C	-1.05205900	0.04249100	0.00936700
H	-1.00524900	-0.84671400	-0.63205800
H	-1.01808400	-0.33480600	1.03893000
C	-2.33123300	0.83062600	-0.25176800
H	-2.34027200	1.17297300	-1.29422800
C	1.54269700	0.19562900	-0.03951500
C	1.75070800	-1.18317200	-0.22763500
C	2.64804700	1.04994100	0.24263200
C	3.02391300	-1.69532200	-0.11899200
H	0.92255700	-1.83871200	-0.46469400
C	3.92000200	0.52411500	0.33865700
H	2.45722700	2.09890400	0.43127900
C	4.11116000	-0.84289000	0.15958100
H	3.19624100	-2.75652000	-0.25126000
H	4.75923500	1.16711800	0.57275300
H	5.10655900	-1.26414400	0.24424600
H	-2.34365400	1.72347900	0.38480400
C	-3.62029300	0.03163500	0.00246100
H	-4.43778800	0.72152600	-0.23502900
C	-3.77226000	-0.37294800	1.46677300
C	-3.75004900	-1.17049900	-0.93005200
H	-3.01072000	-1.94743200	-0.70683400
H	-4.73225400	-1.63462200	-0.81914100
H	-3.63900800	-0.88430100	-1.97983500
H	-3.67903500	0.48630100	2.13673200
H	-4.75414000	-0.81830100	1.63945100
H	-3.03080700	-1.12116000	1.76642400
H	-0.51346500	2.49041000	-0.98817600

No.	Species	Optimized Structure
9	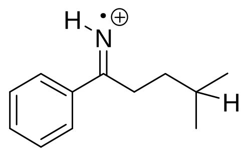	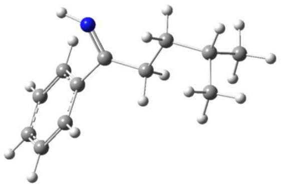

Cartesian Coordinates

C	-0.18070300	-0.69960000	-0.45672700
N	-0.20052000	-1.70283100	-1.22566700
C	1.05876400	-0.04496900	0.02166400
H	0.95763100	1.02922300	-0.18034400
H	1.02748100	-0.12013600	1.11815200
C	2.34608000	-0.62801800	-0.55147100
H	2.32741400	-0.51920000	-1.64090900
C	-1.54804400	-0.19760200	-0.16337500
C	-1.92009800	1.10188100	-0.55134900
C	-2.46615900	-1.05375100	0.48111300
C	-3.17556600	1.56246200	-0.21542400
H	-1.22494000	1.73583200	-1.08849700
C	-3.71542500	-0.57168200	0.82161300
H	-2.17038300	-2.05967200	0.75696500
C	-4.07357100	0.72838300	0.46688100
H	-3.46921800	2.57130700	-0.47913100
H	-4.41440200	-1.20440200	1.35439500
H	-5.06253200	1.09834100	0.71193700
H	2.36789600	-1.70400700	-0.34470000
C	3.62451300	0.01701900	0.00187100
H	4.44972500	-0.50001700	-0.50116500
C	3.78867300	-0.21139700	1.50275400
C	3.72830500	1.49822500	-0.35613800
H	2.97457700	2.10095600	0.16211800
H	4.70226300	1.89478300	-0.06162000
H	3.61795000	1.66294900	-1.43164100
H	3.71969300	-1.27197700	1.76087900
H	4.76404500	0.14483900	1.84074900
H	3.03734100	0.33106100	2.08708300
H	-1.00151000	-2.27576800	-1.49307200

No.	Species	Optimized Structure
10	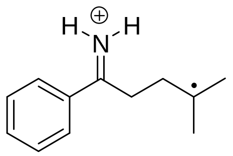	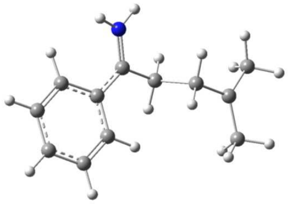

Cartesian Coordinates

No.	Species	Optimized Structure

C 0.23644700 1.14009100 0.21408200
N 0.25133300 2.40805000 -0.14986100
C -1.06505100 0.57391100 0.61546000
H -0.93130400 -0.27522500 1.28263900
H -1.65322500 1.32866600 1.14358700
C -1.90000700 0.11804600 -0.66614700
H -1.30257500 -0.60955900 -1.22105000
C 1.44263000 0.34726800 0.11778200
C 1.36913300 -1.05673500 0.12856500
C 2.70781000 0.95759100 0.01923600
C 2.51744500 -1.82021000 0.01484900
H 0.41174100 -1.55612500 0.20657000
C 3.85150400 0.18898100 -0.08037600
H 2.81386900 2.03565800 0.07425300
C 3.75885600 -1.20120900 -0.09066900
H 2.44700900 -2.90096600 0.01199900
H 4.82037400 0.66965200 -0.13761700
H 4.65769200 -1.80129500 -0.16963800
H -2.02774400 1.00527900 -1.29492400
C -3.20232400 -0.44778200 -0.27356700
H 1.06198900 2.83264200 -0.57671800
H -0.56063600 2.99498800 -0.02415800
C -3.32612900 -1.89761100 0.01876500
H -3.21519500 -2.10442000 1.09564500
H -4.31840000 -2.27077200 -0.25131600
H -2.57859800 -2.49455000 -0.50864000
C -4.31141700 0.46246000 0.10680900
H -4.22421800 1.44564400 -0.36205000
H -5.28177700 0.03716800 -0.16548000
H -4.35171400 0.61806700 1.19719000

11	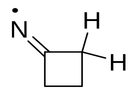	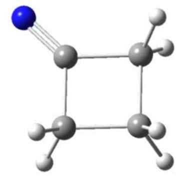

Cartesian Coordinates
C -0.34583300 1.10120300 0.00017500
C -1.43513400 -0.00000100 0.00012500
C -0.34583000 -1.10120300 0.00007900
C 0.73089400 0.00000100 -0.00008900
H -0.30574100 1.73353500 -0.88826700
H -0.30556300 1.73320100 0.88884900
H -2.07157200 0.00003500 -0.88464700

No.	Species	Optimized Structure

H	-2.07163700	-0.00004000	0.88485100
H	-0.30573500	-1.73344600	-0.88842700
H	-0.30556100	-1.73329200	0.88868800
N	1.96303200	0.00000100	-0.00039900

No.	Species	Optimized Structure
12	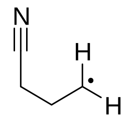	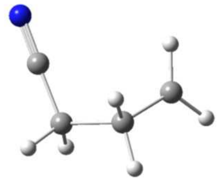

Cartesian Coordinates

C	1.24767000	0.06257500	-0.06287900
N	2.14180600	-0.64177600	0.11922000
C	0.10358000	0.93626900	-0.27044900
H	0.34636700	1.92247200	0.13767100
H	-0.04807300	1.05703900	-1.34686900
C	-1.18735500	0.39145400	0.39125000
H	-0.99758500	0.31215600	1.46832100
H	-1.96368700	1.14923000	0.25713400
C	-1.62943300	-0.91493000	-0.15399300
H	-2.47291100	-0.98623000	-0.82793000
H	-1.06351600	-1.81443800	0.05355700

No.	Species	Optimized Structure
13	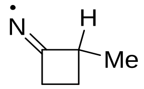	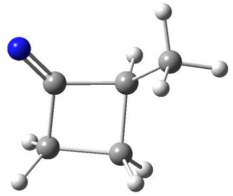

Cartesian Coordinates

C	-1.45645000	0.62244300	-0.08979800
C	-0.13502700	1.42543200	-0.11321700
C	0.63045800	0.19385900	0.43640100
C	-0.62114600	-0.65312500	0.10101000
H	-2.06649100	0.81806600	0.79521500
H	-2.08944600	0.63838000	-0.97681500
H	-0.09963000	2.33309800	0.48941800
H	0.17595700	1.66324100	-1.13263700
H	0.70837000	0.25088800	1.52784400
N	-0.81254600	-1.85518400	-0.08713900
C	1.94841600	-0.21507900	-0.18039100
H	2.72283400	0.52480600	0.04154200
H	2.27837800	-1.18115900	0.20765500
H	1.86034400	-0.30220700	-1.26627400

No.	Species	Optimized Structure
14	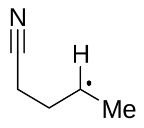	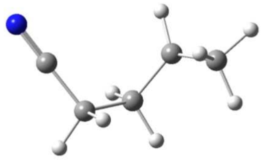

Cartesian Coordinates

C	0.72541600	-0.85272800	-0.52052700
C	-0.43054500	-0.96105400	0.50848400
C	-1.25802600	0.26852200	0.61929400
C	1.69876300	0.16661900	-0.16208900
H	1.24692400	-1.81144100	-0.60507900
H	0.32676800	-0.61626900	-1.51155200
H	0.00045400	-1.22364700	1.47856300
H	-1.05116000	-1.80729200	0.18950900
H	-0.96715200	1.02068800	1.34415400
N	2.46281600	0.97419500	0.14279800
C	-2.26562900	0.62086600	-0.41166200
H	-1.81881800	1.14498800	-1.27198300
H	-2.76366100	-0.26940700	-0.81100900
H	-3.03293900	1.28966500	-0.01319700

No.	Species	Optimized Structure
15	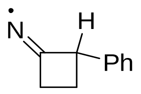	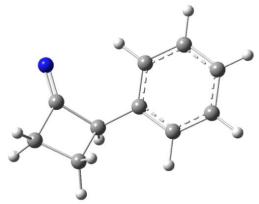

Cartesian Coordinates

C	3.02642700	-0.42512000	-0.39765500
C	1.87098900	-1.42831000	-0.18273600
C	1.05818900	-0.33757800	0.56748600
C	2.08688900	0.70197600	0.05764300
H	3.85460300	-0.54636100	0.30406400
H	3.42118600	-0.31224100	-1.40719200
H	2.10815300	-2.33112600	0.37946800
H	1.38948100	-1.70566700	-1.12140900
N	2.10053400	1.93028200	-0.02965300
H	1.21060200	-0.43078700	1.64998100
C	-0.39824900	-0.14389300	0.26475800
C	-1.23244900	-1.25809300	0.15629500
C	-0.94947300	1.12761400	0.11610200
C	-2.59100400	-1.10412500	-0.08800800
H	-0.81638300	-2.25472100	0.26593600
C	-2.30867300	1.28100300	-0.12954800

No.	Species	Optimized Structure

H	-0.30278200	1.99682400	0.17811300
C	-3.13383200	0.16753400	-0.23069600
H	-3.22576300	-1.97962000	-0.16962300
H	-2.72227800	2.27635600	-0.24817900
H	-4.19344000	0.28932700	-0.42542300

No.	Species	Optimized Structure
16	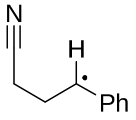	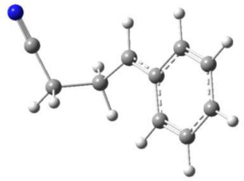

Cartesian Coordinates

C	2.52744900	0.92158900	0.32793300
C	1.63715900	0.80479700	-0.93374000
C	0.63712000	-0.29254300	-0.84633700
C	3.33680800	-0.26646100	0.54972300
H	3.19927900	1.78116000	0.23767900
H	1.90327700	1.08500500	1.21110300
H	2.28988700	0.64432900	-1.79713300
H	1.15331800	1.77400300	-1.07991500
N	3.97659400	-1.21325800	0.70118500
H	0.97749300	-1.29245400	-1.09606100
C	-0.69617400	-0.15448200	-0.39532100
C	-1.53201400	-1.29743500	-0.32699000
C	-1.25941300	1.08251100	0.00345300
C	-2.83831400	-1.20713300	0.10872900
H	-1.12389700	-2.25764700	-0.62489400
C	-2.56886400	1.16230200	0.43970700
H	-0.65986300	1.98505800	-0.03130200
C	-3.36907700	0.02313800	0.49661100
H	-3.45378400	-2.09903000	0.15023800
H	-2.97512400	2.12203800	0.74000700
H	-4.39481500	0.09264400	0.83937700

No.	Species	Optimized Structure
17	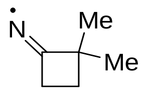	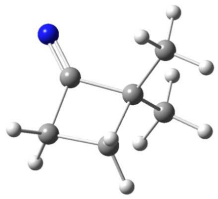

Cartesian Coordinates

C	-1.55554500	0.77120600	-0.05787500
C	-0.19347100	1.39796000	-0.43297600
C	0.54555200	0.08946900	-0.02629600
C	-0.85721900	-0.58749400	0.09014600

No.	Species	Optimized Structure

H	-1.95925500	1.11442600	0.89714800
H	-2.34694200	0.79421500	-0.80729100
H	0.09350300	2.29139000	0.12361300
H	-0.10915800	1.60609100	-1.50105800
N	-1.22447500	-1.75866000	0.17140200
C	1.45514200	-0.52826400	-1.07089500
H	2.35007400	0.08829100	-1.20369700
H	1.77014900	-1.52927800	-0.76633200
H	0.95183000	-0.61286400	-2.03679700
C	1.21426100	0.18063100	1.33849200
H	2.10148600	0.81912400	1.27671400
H	0.54382300	0.60656000	2.08971300
H	1.52349200	-0.80838700	1.68459900

No.	Species	Optimized Structure
18	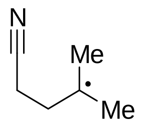	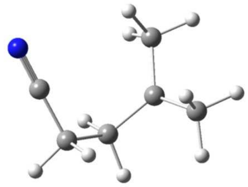

Cartesian Coordinates

C	1.09074800	-1.13032700	0.11681200
C	-0.18212300	-0.80125300	0.94000300
C	-1.18118000	0.03950000	0.21932800
C	1.84912900	0.05598700	-0.24581100
H	1.74672500	-1.79789900	0.68462900
H	0.81748600	-1.65730600	-0.80238500
H	0.12529600	-0.31639800	1.87099700
H	-0.62645100	-1.76779700	1.21035200
N	2.43620500	1.00935200	-0.52124000
C	-1.90220600	-0.54197500	-0.94672300
H	-1.37504500	-0.36180000	-1.89812700
H	-2.02806400	-1.62518000	-0.85162900
H	-2.89532300	-0.09567900	-1.06333500
C	-1.20630800	1.51309400	0.42698400
H	-0.47774800	2.03357300	-0.21443500
H	-2.18983800	1.93168400	0.19069200
H	-0.95883300	1.78117400	1.45836500

No.	Species	Optimized Structure
19	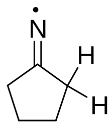	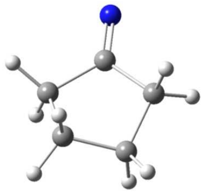

Cartesian Coordinates

<table>
<tr><th>No.</th><th>Species</th><th>Optimized Structure</th></tr>
<tr><td colspan="3">C -1.32670000 -0.72537100 -0.24318600
C -1.32667100 0.72546300 0.24308200
C 0.06755100 1.23641200 -0.12953200
C 0.96344200 -0.00012200 0.00001800
C 0.06743500 -1.23641200 0.12966900
H -2.12593300 -1.32620700 0.19546100
H -1.45362700 -0.75180600 -1.33048000
H -1.45380000 0.75190300 1.33035100
H -2.12580700 1.32634600 -0.19568700
H 0.09757400 1.56204400 -1.17461500
H 0.43214000 2.06038700 0.48413700
H 0.43192500 -2.06062800 -0.48373900
H 0.09725100 -1.56184600 1.17483500
N 2.20427600 -0.00000200 -0.00008200</td></tr>
<tr><td>20</td><td>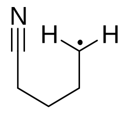
</td><td>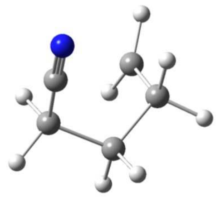</td></tr>
<tr><td colspan="3">Cartesian Coordinates
C 1.64103400 -0.12872600 -0.15305700
N 2.43064700 -0.89154900 0.19864600
C 0.62910700 0.83000000 -0.57163900
H 1.12111000 1.79145600 -0.74473800
H 0.22146500 0.49622100 -1.53156000
C -0.50209900 0.97751700 0.45516500
H -0.08087800 1.29720700 1.41213500
H -1.16159900 1.78131800 0.11206300
C -1.31293300 -0.30655900 0.64545800
H -2.01625300 -0.13752900 1.47754200
H -0.65226500 -1.11256400 0.98321500
C -2.05856300 -0.72495600 -0.56756600
H -2.58762600 0.01071700 -1.16420200
H -2.23775500 -1.76964400 -0.78514300</td></tr>
<tr><td>21</td><td>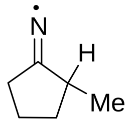
</td><td>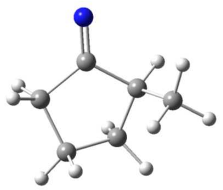</td></tr>
<tr><td colspan="3">Cartesian Coordinates
C -1.53189600 -0.58985400 0.41722000
C -0.58856200 -1.25051200 -0.59133300</td></tr>
</table>

No.	Species	Optimized Structure

C	0.71056500	-0.43271800	-0.50646200
C	0.21046400	0.98011800	-0.14128600
C	-1.27286700	0.90544000	0.22705200
H	-2.57882100	-0.85780700	0.26083400
H	-1.26864800	-0.89029500	1.43606000
H	-1.01367200	-1.16971600	-1.59712000
H	-0.41985700	-2.31161800	-0.39152700
H	1.22774300	-0.38767200	-1.46764600
H	-1.50264200	1.52674600	1.09290600
H	-1.84603700	1.29382500	-0.62168600
N	0.91024500	2.00407700	-0.13710800
C	1.68022500	-0.94593300	0.55661000
H	2.53851500	-0.27806900	0.64777300
H	1.20229400	-1.01003600	1.53827600
H	2.04183900	-1.94314100	0.29108000

No.	Species	Optimized Structure
22	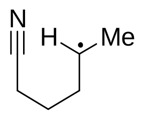	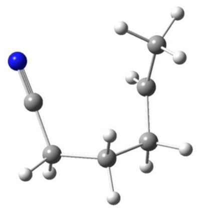

Cartesian Coordinates

C	1.59053700	0.62391200	0.07145600
N	1.68911600	1.77295800	0.05485500
C	1.50434300	-0.82940500	0.05045100
H	2.36122500	-1.19490100	-0.52402600
H	1.63128400	-1.19785500	1.07412100
C	0.20021100	-1.36857700	-0.55210600
H	0.03178200	-0.89751100	-1.52502500
H	0.33898000	-2.43795500	-0.73969300
C	-1.03370300	-1.17569700	0.34924700
H	-0.86651500	-1.71583700	1.28772500
H	-1.87368500	-1.67873900	-0.15203300
C	-1.39730700	0.23928900	0.63162800
H	-1.18233900	0.65509000	1.60925000
C	-1.89496900	1.15049400	-0.42876200
H	-2.67176400	1.82291200	-0.04972000
H	-1.09498500	1.79660000	-0.81965900
H	-2.31246000	0.59740400	-1.27640100

No.	Species	Optimized Structure
23	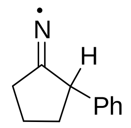	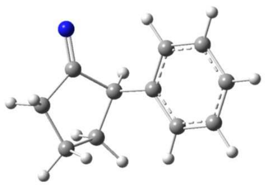

Cartesian Coordinates

C	2.14936000	-1.32862000	-0.62311900
C	1.68387300	-1.24033400	0.83215000
C	0.87874600	0.06192300	0.89666100
C	1.71028300	0.98114900	-0.02681700
C	2.54681700	0.11074700	-0.97002800
H	2.97218300	-2.03237100	-0.76320400
H	1.32392000	-1.65463700	-1.26170100
H	2.55305800	-1.14322900	1.49067800
H	1.12594400	-2.11409200	1.17235400
H	2.36863800	0.38501600	-2.01058500
H	3.60240300	0.30021700	-0.75645000
N	1.72394400	2.21924100	0.03368500
H	0.87453600	0.48880600	1.90263600
C	-0.56155000	-0.01073200	0.40726900
C	-1.27708900	1.17700600	0.23444400
C	-1.21660000	-1.21678100	0.16426600
C	-2.60655700	1.15897300	-0.15982500
H	-0.77267600	2.12405500	0.39738200
C	-2.54929000	-1.23722600	-0.23612900
H	-0.69558400	-2.15891700	0.28734100
C	-3.25023400	-0.05094600	-0.39823600
H	-3.14081700	2.09414500	-0.28616000
H	-3.03751500	-2.18780800	-0.42108700
H	-4.28825200	-0.06683200	-0.71081700

No.	Species	Optimized Structure
24	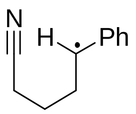	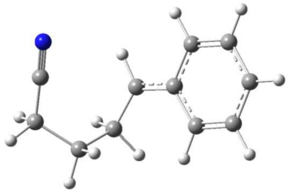

Cartesian Coordinates

C	2.78568900	1.14707400	-0.47638700
N	2.44541300	2.24790300	-0.51823700
C	3.25729400	-0.23037800	-0.43939900
H	3.86628800	-0.38894100	-1.33489900
H	3.93144700	-0.33136900	0.41838500
C	2.15478500	-1.29407800	-0.36931300

No.	Species	Optimized Structure

H	1.48222600	-1.17931900	-1.22372200
H	2.64474400	-2.26732500	-0.47442800
C	1.33871500	-1.28442300	0.93776000
H	2.03432500	-1.30306800	1.78433900
H	0.77869600	-2.22529600	0.98471200
C	0.40988700	-0.12805900	1.07734400
H	0.76003300	0.75012200	1.60819500
C	-0.88606400	-0.06319900	0.51212400
C	-1.65812000	1.11520800	0.66185500
C	-1.47031900	-1.13171500	-0.21052000
C	-2.92757600	1.21442500	0.12916100
H	-1.22779100	1.95220500	1.20141200
C	-2.74327600	-1.02274100	-0.74020800
H	-0.91629400	-2.05310900	-0.35042300
C	-3.48270900	0.14645100	-0.57593800
H	-3.49406700	2.13020000	0.25801400
H	-3.16790900	-1.85674100	-1.28864000
H	-4.47943000	0.22594600	-0.99415200

No.	Species	Optimized Structure
25	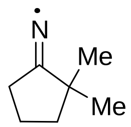	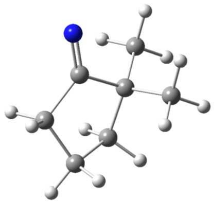

Cartesian Coordinates

C	-1.72328300	-0.87266600	0.07643700
C	-0.37984300	-1.32928800	-0.49235800
C	0.64129300	-0.27125100	-0.03256500
C	-0.22031100	1.01878000	0.00624300
C	-1.69873600	0.64520400	-0.11511100
H	-2.57570600	-1.34115400	-0.41980100
H	-1.79299200	-1.11979900	1.13986000
H	-0.42755400	-1.33364100	-1.58728300
H	-0.09381200	-2.33520200	-0.17355000
H	-2.31379800	1.20761300	0.58791200
H	-2.02413700	0.91376600	-1.12564900
N	0.23143100	2.16727200	0.12185600
C	1.12906900	-0.55813200	1.39199800
H	1.76586400	0.25439400	1.74759000
H	0.29980300	-0.66330300	2.09616700
H	1.70700800	-1.48734900	1.40850200
C	1.82655300	-0.12938700	-0.97939500
H	2.39484800	-1.06381200	-1.01911500
H	1.49571100	0.11167000	-1.99275100

No.	Species	Optimized Structure

H	2.49629700	0.66635200	-0.64637400

No.	Species	Optimized Structure
26	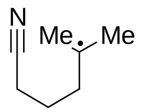	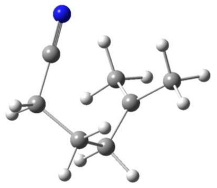

Cartesian Coordinates

C	-1.56762300	-0.93315800	-0.00768000
N	-1.36929500	-2.03612700	-0.28167500
C	-1.86536700	0.45365800	0.32098900
H	-2.88821900	0.65406200	-0.01281000
H	-1.86451900	0.55381900	1.41181600
C	-0.89944700	1.46750600	-0.30616100
H	-0.85811300	1.30209900	-1.38685900
H	-1.32472100	2.46519400	-0.15700700
C	0.51877900	1.42959400	0.28777700
H	0.45573000	1.64684300	1.36027500
H	1.07209500	2.26852800	-0.16279900
C	1.26328400	0.15547900	0.06933000
C	1.62420800	-0.26728500	-1.31190600
H	2.69230300	-0.51216300	-1.38788900
H	1.07928000	-1.17431600	-1.61005800
H	1.40853500	0.50660100	-2.05385800
C	1.66912700	-0.72703300	1.19557500
H	1.21272700	-1.72332300	1.10724500
H	2.75656900	-0.89106200	1.20843800
H	1.38562800	-0.31596800	2.16769500

No.	Species	Optimized Structure
27	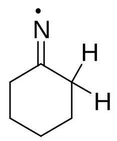	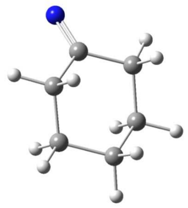

Cartesian Coordinates

C	0.95375500	1.26056500	-0.28550800
C	-0.42806900	1.28278000	0.37427600
C	-1.18168000	0.00000600	0.06928000
C	-0.42807600	-1.28277000	0.37428900
C	0.95373700	-1.26057100	-0.28551700
C	1.73192100	-0.00001000	0.08239400
H	-0.31417000	1.34986500	1.46368200
H	-1.02041400	2.13887500	0.04812600
H	0.82991900	1.30801100	-1.37366400

No.	Species	Optimized Structure

H	1.51111300	2.15564800	0.00677300
H	-0.31415700	-1.34983700	1.46369400
H	-1.02043400	-2.13886600	0.04816400
H	1.51108700	-2.15566800	0.00674000
H	0.82988200	-1.30799800	-1.37367300
H	1.93649200	-0.00001700	1.16096400
H	2.70439100	-0.00001200	-0.41873000
N	-2.32331900	0.00000100	-0.42962300

No.	Species	Optimized Structure
28	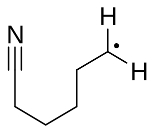	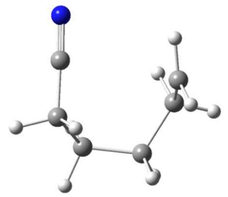

Cartesian Coordinates

C	1.47052900	0.72775100	-0.47829900
C	0.97850500	1.61810300	0.60327600
C	-1.68124700	0.16146500	-0.00617700
C	-0.99171500	-0.96130400	0.61246000
C	0.17747500	-1.51219200	-0.22343100
C	1.50357500	-0.76329100	-0.10287800
H	1.40971300	1.54670600	1.59637300
H	0.30532100	2.43933900	0.39954600
H	0.87690300	0.87092100	-1.38709400
H	2.49903900	1.02085000	-0.74566900
H	-0.64892300	-0.65449700	1.60530900
H	-1.73910600	-1.74714700	0.76254400
H	0.33998600	-2.54792000	0.08996800
H	-0.13139200	-1.55423800	-1.27274900
H	1.88578800	-0.86247800	0.92074500
H	2.22551100	-1.27762100	-0.74578100
N	-2.25222500	1.02041400	-0.52184300

No.	Species	Optimized Structure
29	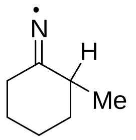	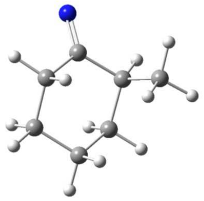

Cartesian Coordinates

C	-0.43375700	-1.20357400	-0.87066700
C	0.90411100	-0.63615700	-0.36945800
C	0.75683400	0.86949500	-0.15048600
C	-0.43145400	1.31747000	0.68235300
C	-1.72575100	0.71141400	0.13400100

No.	Species	Optimized Structure

C	-1.62066100	-0.80569600	0.00487100
H	1.65960700	-0.76971000	-1.14816800
H	-0.60750600	-0.83882000	-1.88953500
H	-0.35442300	-2.29394100	-0.93765800
H	-0.27290900	0.98096100	1.71372300
H	-0.46761100	2.40778900	0.69487300
H	-2.56195200	0.98691900	0.78371500
H	-1.93372300	1.14787400	-0.84997800
H	-1.52204300	-1.25242200	1.00131400
H	-2.54322500	-1.21083700	-0.42132500
N	1.55151300	1.68191500	-0.66050000
C	1.40510900	-1.32223300	0.90323500
H	2.36110200	-0.89988600	1.22008100
H	0.70385700	-1.22561600	1.73560900
H	1.55165400	-2.39003200	0.71776800

No.	Species	Optimized Structure
30	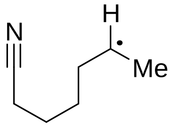	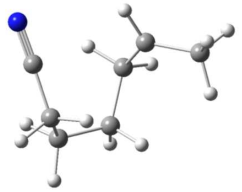

Cartesian Coordinates

C	1.03718000	-0.41304700	-1.00380600
C	1.38077600	0.79467800	-0.19858200
C	-1.74940400	0.91865400	0.07731400
C	-1.53033000	-0.26716800	0.89166900
C	-1.06333900	-1.49638400	0.09031000
C	0.43405700	-1.58173600	-0.19708200
H	0.89020700	1.73378500	-0.42288900
H	0.35231600	-0.14390900	-1.81417400
H	1.95156600	-0.79161500	-1.48668000
H	-0.80820400	-0.01979900	1.67594900
H	-2.47973100	-0.48888700	1.38959000
H	-1.35183500	-2.38361200	0.66220300
H	-1.62599500	-1.53959000	-0.84758000
H	0.97841700	-1.68513800	0.74922200
H	0.60543100	-2.51448000	-0.74499300
N	-1.95697700	1.84403200	-0.57863600
C	2.56940100	0.79939600	0.69173900
H	2.52810100	1.60607200	1.42773900
H	2.68692900	-0.14685100	1.23277900
H	3.50158800	0.93944400	0.11991100

No.	Species	Optimized Structure
31	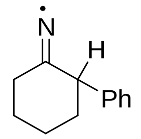	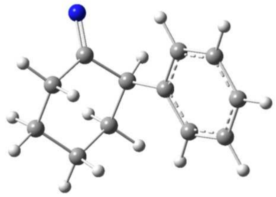

Cartesian Coordinates

C	-1.43782300	-1.10937600	-1.12295600
C	-0.62039000	0.18574000	-1.00400600
C	-1.41899100	1.18267300	-0.15568000
C	-1.90731000	0.67096100	1.18505000
C	-2.70864000	-0.61893000	0.99758100
C	-1.90512300	-1.66012300	0.22378300
H	-2.31714600	-0.88855800	-1.73826400
H	-0.86385100	-1.85870500	-1.67631200
H	-1.02486800	0.47299300	1.80510700
H	-2.49580500	1.44833200	1.67425300
H	-3.00315100	-1.01072300	1.97568400
H	-3.63425700	-0.39017500	0.45651900
H	-1.04316000	-1.96844800	0.82549300
H	-2.51055500	-2.55694000	0.06142400
N	-1.66793900	2.33031900	-0.57058400
C	0.78831100	0.03844700	-0.44215200
C	1.41337700	-1.19836500	-0.28642000
C	1.51505500	1.18928600	-0.12386800
C	2.72264800	-1.28441100	0.17643000
H	0.88573000	-2.11203400	-0.53248400
C	2.82111900	1.10591700	0.33776700
H	1.04532100	2.16028700	-0.24298100
C	3.43150700	-0.13386700	0.49219400
H	3.18721400	-2.25795400	0.28878900
H	3.36447600	2.01356600	0.57648400
H	4.45117900	-0.20106100	0.85431800
H	-0.52798900	0.62948500	-2.00027800

No.	Species	Optimized Structure
32	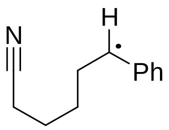	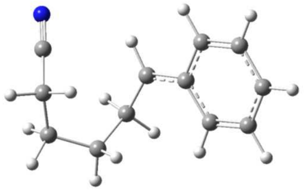

Cartesian Coordinates

C	0.97959100	1.05413700	-1.22076200
C	0.15752500	-0.15926300	-0.94610400
C	2.86524000	-1.32828900	0.23210600

No.	Species	Optimized Structure
C	2.67033200 -0.27449400 1.21556200	
C	2.81288500 1.14875300 0.64322400	
C	1.55582800 1.75881900 0.02828200	
H	1.80153700 0.79268900 -1.89458700	
H	0.36840000 1.79145700 -1.75710900	
H	1.68768600 -0.40888300 1.67890700	
H	3.41557400 -0.43426800 2.00113300	
H	3.12832800 1.79534100 1.46758500	
H	3.63075200 1.15926800 -0.08405700	
H	0.77484900 1.82394600 0.79393800	
H	1.80233200 2.79037700 -0.24608900	
N	3.04862000 -2.15741800 -0.54818000	
H	0.59324600 -1.13104500 -1.14894400	
C	-1.16640400 -0.13424600 -0.44403200	
C	-1.87243500 1.06599600 -0.18400600	
C	-1.84377400 -1.35192300 -0.18422600	
C	-3.16556900 1.04121300 0.30601600	
H	-1.39750900 2.02041500 -0.38070700	
C	-3.13410300 -1.36601200 0.30631100	
H	-1.32597400 -2.28534300 -0.38006300	
C	-3.80692500 -0.16987700 0.55738600	
H	-3.68505400 1.97498000 0.49307900	
H	-3.62707600 -2.31356400 0.49476900	
H	-4.82056900 -0.18233800 0.94085200	
33	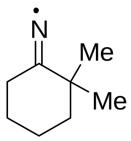	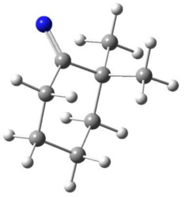
Cartesian Coordinates		
C	-0.10581500 -1.28601800 -0.67538900	
C	0.84408200 -0.29513000 0.02858500	
C	0.17470300 1.08987000 0.01360100	
C	-1.26766300 1.16167500 0.49107600	
C	-2.13258300 0.14426500 -0.25402800	
C	-1.54243700 -1.25858100 -0.15987000	
H	-0.10996100 -1.05068000 -1.74683000	
H	0.31712900 -2.29276000 -0.58013300	
H	-1.28709500 0.95128400 1.56673000	
H	-1.63262700 2.18042200 0.35185400	
H	-3.15030900 0.16460900 0.14757100	
H	-2.20274200 0.44073700 -1.30721700	
H	-1.58003000 -1.60545400 0.87944000	

No.	Species	Optimized Structure

H -2.14997700 -1.96168700 -0.73762400
N 0.76046900 2.11012100 -0.39612500
C 1.08815500 -0.70701600 1.48645400
H 1.74796400 0.00927000 1.98228500
H 0.16767300 -0.77589300 2.06925900
H 1.57287700 -1.68776600 1.51270600
C 2.17966700 -0.25139500 -0.70982400
H 2.87091900 0.44592400 -0.23194300
H 2.63730200 -1.24516100 -0.71175100
H 2.04693800 0.07028600 -1.74510200

No.	Species	Optimized Structure
34	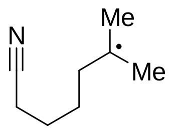	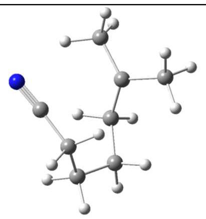

Cartesian Coordinates
C -0.68639600 0.85829200 -0.97876000
C -1.37926300 -0.03026900 0.00926000
C 1.46405900 -1.32161100 0.22859400
C 1.80289200 -0.03416300 0.81582900
C 1.77780100 1.13072000 -0.19091900
C 0.41574200 1.77736300 -0.43527500
H -0.27741500 0.24521100 -1.79032300
H -1.44555500 1.50403000 -1.45778000
H 1.12493400 0.15920400 1.65236200
H 2.80812300 -0.13589600 1.23786100
H 2.45726200 1.90081500 0.18667100
H 2.20289500 0.78509600 -1.13854000
H 0.07505000 2.25817200 0.48847100
H 0.57046900 2.59283600 -1.14944500
N 1.25638500 -2.34737800 -0.25601900
C -2.00684600 0.57206000 1.22048000
H -2.19212200 -0.18371000 1.98960300
H -1.40499200 1.36770000 1.66812400
H -2.98709600 1.02084500 0.98231200
C -1.95191600 -1.30808700 -0.49710800
H -2.25765200 -1.96665700 0.32064300
H -2.84961900 -1.12865800 -1.11380300
H -1.23541700 -1.85317400 -1.11662100

No.	Species	Optimized Structure
35	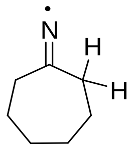	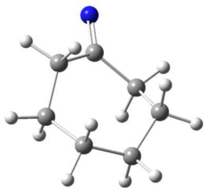

Cartesian Coordinates

C	0.58476800	-1.52277600	-0.39983700
C	1.79341800	-0.75145300	0.12999500
C	1.79311600	0.75190800	-0.12999900
C	-0.71328400	-1.26774200	0.37101600
C	0.58421500	1.52273200	0.39999000
C	-1.46694000	-0.00030400	-0.00058900
C	-0.71371400	1.26771600	-0.37109600
H	0.43920800	-1.31826500	-1.46728300
H	1.87064700	-0.93047100	1.21060100
H	1.87022900	0.93102400	-1.21059900
H	0.43862200	1.31776800	1.46734400
H	-0.48660800	-1.21083600	1.44326000
H	0.80376300	-2.59286000	-0.32853900
H	2.70246700	-1.17547800	-0.31046900
H	2.70202400	1.17624600	0.31045500
H	-1.40804600	-2.10117600	0.24860100
H	0.80296500	2.59289700	0.32913900
H	-1.40878200	2.10081800	-0.24813400
H	-0.48698600	1.21163900	-1.44336400
N	-2.71556700	-0.00025700	0.00030100

No.	Species	Optimized Structure
36	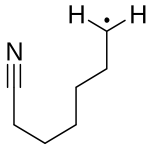	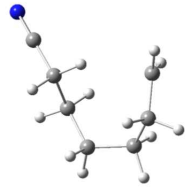

Cartesian Coordinates

C	0.49593500	1.08481100	-0.33228800
C	-0.90396100	1.48656400	0.14277000
C	-2.08393700	0.61406100	-0.29649200
C	1.18067000	0.03669700	0.55506300
C	-2.20074600	-0.78383300	0.32340000
C	2.52907000	-0.27221100	0.10919000
C	-1.34458400	-1.83257600	-0.28388200
H	0.45256700	0.71329000	-1.36117600
H	-0.90573400	1.57658200	1.23703000
H	-2.07580400	0.51942000	-1.38967500

No.	Species	Optimized Structure

H	-2.02756700	-0.72886100	1.40671300
H	0.60076100	-0.89453000	0.56057700
H	1.13323100	1.97342400	-0.35567400
H	-1.09851300	2.49628400	-0.23300000
H	-2.99802400	1.16583700	-0.05350700
H	1.23411500	0.39347800	1.58910500
H	-3.25386400	-1.10189300	0.23178500
H	-1.17481500	-2.77151500	0.23007400
H	-1.08678500	-1.78842100	-1.33632100
N	3.59510600	-0.50773800	-0.26178600

No.	Species	Optimized Structure
37	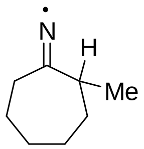	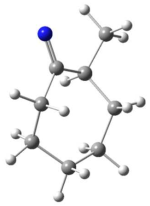

Cartesian Coordinates

C	-1.66426100	1.06304200	-0.45493400
C	-2.26346700	-0.23780100	0.07467300
C	-1.40230000	-1.48382600	-0.10408900
C	-0.47301800	1.57888900	0.35733300
C	0.00822900	-1.41571800	0.48079400
C	0.87931700	0.95580000	0.03926200
C	0.99419100	-0.53172600	-0.29953000
H	-1.38770100	0.95673000	-1.51051400
H	-2.48734600	-0.10475600	1.14130100
H	-1.32116700	-1.71659500	-1.17401100
H	-0.02552500	-1.10817600	1.53320300
H	-0.66749500	1.40942100	1.42384300
H	-2.43953200	1.83515900	-0.42640500
H	-3.22756100	-0.41046000	-0.41595500
H	-1.92869900	-2.33166200	0.34753100
H	-0.36032800	2.65786700	0.23459500
H	0.42165100	-2.42972300	0.48261000
H	0.72259200	-0.60247400	-1.36294500
N	1.89116100	1.68722600	0.05507700
C	2.43315600	-1.01818800	-0.14963300
H	2.52598500	-2.04377800	-0.51588700
H	3.12600400	-0.38514800	-0.70571900
H	2.73991600	-0.99982400	0.89956100

No.	Species	Optimized Structure
38	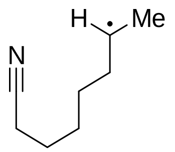	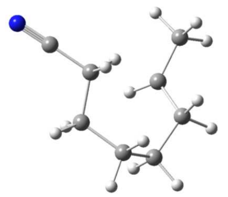

Cartesian Coordinates

C	1.01110300	1.28368500	-0.27968400
C	-0.24748900	2.00821100	0.20765400
C	-1.60273500	1.49632500	-0.29050900
C	1.37281000	0.04226100	0.54629800
C	-2.08659300	0.14133300	0.23744500
C	2.59716300	-0.59383800	0.08951000
C	-1.52381500	-1.05890700	-0.43754100
H	0.89735700	0.99692300	-1.33002000
H	-0.25064500	2.03487200	1.30535400
H	-1.59654300	1.46894100	-1.38753700
H	-1.91501500	0.07842400	1.32263800
H	0.56322100	-0.69565100	0.48982700
H	1.85882700	1.97377200	-0.24099200
H	-0.16579500	3.05318400	-0.10893700
H	-2.34868900	2.25001400	-0.01765700
H	1.49786900	0.31198300	1.60022800
H	-3.18655500	0.11317200	0.13609700
H	-1.21415200	-0.95964400	-1.47457900
N	3.56670100	-1.08954800	-0.28986900
C	-1.77618300	-2.42199900	0.09521000
H	-2.81053000	-2.74978300	-0.09928400
H	-1.11901700	-3.16753100	-0.35849500
H	-1.64281300	-2.46426400	1.18214500

No.	Species	Optimized Structure
39	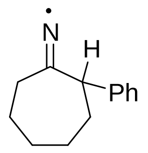	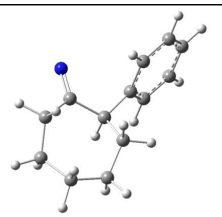

Cartesian Coordinates

C	-3.34014100	0.36363700	-0.56026000
C	-3.44777300	-0.91079200	0.27453500
C	-2.20286900	-1.79010500	0.29924600
C	-2.41042000	1.42340200	0.03793800
C	-0.90916800	-1.11811900	0.75870700
C	-0.92357700	1.25551900	-0.24288300
C	-0.29264900	-0.13952200	-0.26512400

No.	Species	Optimized Structure

H	-3.03622600	0.12630500	-1.58675200
H	-3.70967500	-0.62777200	1.30247500
H	-2.03625600	-2.21001500	-0.70140700
H	-1.05310700	-0.61610800	1.72289800
H	-2.53835700	1.43662900	1.12764700
H	-4.33690000	0.80840600	-0.64016100
H	-4.28690700	-1.50937500	-0.09616400
H	-2.39969800	-2.64617700	0.95354500
H	-2.68439800	2.41963700	-0.31471700
H	-0.16339100	-1.89760400	0.93734900
N	-0.24275300	2.27717500	-0.47106100
H	-0.52448700	-0.53658000	-1.26268800
C	1.21539700	-0.09581100	-0.14358400
C	2.01163500	-0.74103400	-1.08697300
C	1.83742300	0.52266200	0.94233800
C	3.39544200	-0.77335800	-0.95314700
H	1.54353200	-1.22055200	-1.94141300
C	3.21784100	0.48975800	1.08087100
H	1.23781300	1.04900100	1.67658700
C	4.00282200	-0.15944500	0.13383000
H	3.99766400	-1.27576200	-1.70216000
H	3.68434500	0.97949700	1.92858700
H	5.08155500	-0.18051100	0.24083900

No.	Species	Optimized Structure
40	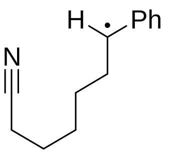	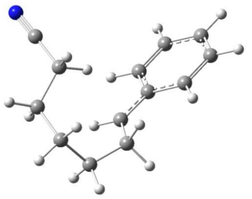

Cartesian Coordinates

C	3.00019700	0.22632400	0.14345700
C	3.07264100	-1.25609300	-0.23791600
C	2.06670900	-2.20870800	0.41534900
C	2.01723700	1.04470700	-0.70599300
C	0.59451500	-2.05497200	0.02020500
C	2.01949500	2.45276000	-0.34339200
C	-0.14170700	-0.97466400	0.73359300
H	2.74048900	0.33358600	1.20149700
H	3.00819900	-1.35370600	-1.32950600
H	2.15396300	-2.13430700	1.50650600
H	0.51986600	-1.92112400	-1.06827700
H	0.99512300	0.66799200	-0.59509800
H	3.99163600	0.67359900	0.02862300
H	4.07136500	-1.61668300	0.02858800

No.	Species	Optimized Structure
H	2.37933700 -3.22707200 0.16286400	
H	2.27865300 0.96349000 -1.76633100	
H	0.08575600 -3.01293500 0.21566800	
N	2.03634300 3.56383800 -0.03593400	
H	0.29414100 -0.59066300 1.65209900	
C	-1.41491600 -0.47694100 0.36373800	
C	-2.02804700 0.54307300 1.13236000	
C	-2.12409400 -0.95536100 -0.76465900	
C	-3.26484100 1.05225300 0.79086800	
H	-1.50375400 0.92839400 2.00094500	
C	-3.36391500 -0.44003800 -1.09652400	
H	-1.69412600 -1.74309800 -1.37249900	
C	-3.94384100 0.56553000 -0.32620100	
H	-3.70838000 1.83634500 1.39450500	
H	-3.88865900 -0.82467600 -1.96437800	
H	-4.91460700 0.96677400 -0.59297800	
41	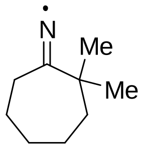	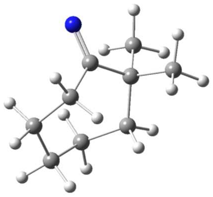
Cartesian Coordinates		
C	-2.15433500 0.78067200 -0.04825300	
C	-2.28040600 -0.73768900 0.03243000	
C	-1.07095300 -1.52897200 -0.46812000	
C	-0.90939500 1.36921100 0.62728100	
C	0.16535600 -1.39877700 0.42837000	
C	0.35583500 1.06431900 -0.16318000	
C	1.12922600 -0.23859800 0.10939200	
H	-2.15258100 1.09871700 -1.09632600	
H	-2.47509800 -1.02806500 1.07324600	
H	-0.82741400 -1.23012500 -1.49500800	
H	-0.16421800 -1.32570800 1.47199500	
H	-0.80835000 0.98903700 1.64970000	
H	-3.04039500 1.23251600 0.41026700	
H	-3.16713200 -1.03899000 -0.53550000	
H	-1.35488900 -2.58444100 -0.52264300	
H	-1.00953100 2.45449500 0.68810700	
H	0.76111100 -2.31652200 0.37276100	
N	0.74587600 1.88064400 -1.02161600	
C	2.03043300 0.01377700 1.32544700	
H	2.55391000 -0.90840700 1.59671000	
H	2.77626800 0.78059800 1.10489400	

No.	Species	Optimized Structure
H	1.45395100 0.33721600 2.19681300	
C	1.99725400 -0.58869900 -1.10093500	
H	1.38685000 -0.81890600 -1.97718700	
H	2.65125400 0.24378000 -1.36641200	
H	2.61704000 -1.46116300 -0.87469500	
42	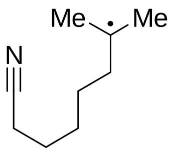	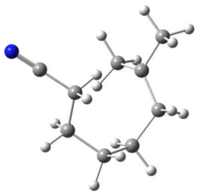
Cartesian Coordinates		
C	1.31870400 -1.29406000 0.26555000	
C	0.14486700 -2.15476600 -0.20994800	
C	-1.26558000 -1.75572500 0.23965100	
C	1.55055400 -0.03565600 -0.57987300	
C	-1.91381800 -0.54024400 -0.43721900	
C	2.68407400 0.74752100 -0.11936600	
C	-1.51463600 0.83051800 0.01386000	
H	1.17228900 -1.00377400 1.31094300	
H	0.16896600 -2.23216800 -1.30508900	
H	-1.27618900 -1.63129300 1.32833100	
H	-1.76830200 -0.61826200 -1.52248600	
H	0.65569300 0.60099600 -0.55398700	
H	2.23340300 -1.89353800 0.24082000	
H	0.32018500 -3.17092100 0.15829500	
H	-1.91532900 -2.61486300 0.04221800	
H	1.72261200 -0.30946800 -1.62620000	
H	-3.00679600 -0.63471900 -0.29468400	
N	3.58142000 1.36238000 0.26362700	
C	-1.77815900 1.95972300 -0.92418000	
H	-2.84377200 2.24640500 -0.91982600	
H	-1.21500700 2.85678400 -0.65007500	
H	-1.53081200 1.69991900 -1.95806000	
C	-1.46076900 1.14910200 1.46898100	
H	-0.98815900 0.36125400 2.06073300	
H	-0.91697200 2.07986000 1.65326200	
H	-2.47317400 1.28865700 1.88567700	
43	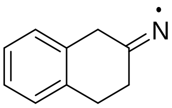	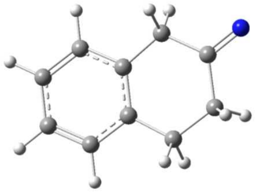

No.	Species	Optimized Structure

Cartesian Coordinates

C	2.89429700	0.44619500	-0.00005900
C	1.78049000	1.26592500	-0.00001400
C	0.48300300	0.73989900	0.00003600
C	0.31851400	-0.64212800	0.00004500
C	1.44971300	-1.46627600	-0.00000100
C	2.72727700	-0.93651600	-0.00005300
H	-0.55710900	2.36295300	0.87031200
H	3.88846600	0.87872800	-0.00010100
H	1.90764700	2.34469100	-0.00002500
C	-0.67201200	1.70914600	0.00010000
C	-1.03239300	-1.30519600	0.00012700
H	1.31391900	-2.54384500	0.00000000
H	3.58905800	-1.59430500	-0.00009100
C	-2.24401100	-0.38794600	-0.00002600
C	-2.09557300	1.13178500	-0.00007000
H	-1.11854900	-1.96023500	-0.87241500
H	-2.64594000	1.49429800	0.87025400
H	-1.11857200	-1.95996900	0.87286900
H	-2.64570600	1.49422300	-0.87057400
H	-0.55700800	2.36317400	-0.86993100
N	-3.38743300	-0.88700600	-0.00011600

No.	Species	Optimized Structure
44	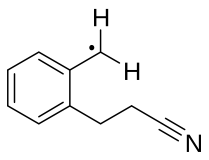	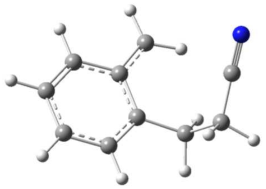

Cartesian Coordinates

C	2.42470800	-1.07481900	-0.50568800
C	1.19045000	-1.42830400	0.02933800
C	0.28409800	-0.47866100	0.48247300
C	0.62890900	0.90423800	0.40611200
C	1.89794100	1.23629300	-0.13694300
C	2.77419100	0.27258300	-0.58606400
H	-0.88074300	-1.90735400	1.53986800
H	3.10874000	-1.84214300	-0.84884100
H	0.92779300	-2.48006200	0.10401300
C	-1.03468600	-0.94973000	1.03486000
C	-0.21925000	1.93700300	0.84101200
H	2.16928400	2.28499800	-0.19656000
H	3.73412700	0.56318600	-0.99802400
C	-2.11765700	-1.18764200	-0.03826000
H	-2.97649600	-1.69703200	0.41063000

No.	Species	Optimized Structure

H	-1.41896600	-0.26106500	1.78978200
H	-1.72642100	-1.84605700	-0.81953200
C	-2.60948700	0.02670900	-0.67045600
N	-3.01994400	0.98745600	-1.15741900
H	-1.21145900	1.75938900	1.22855300
H	0.09844300	2.96793500	0.75373800

No.	Species	Optimized Structure
45	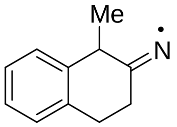	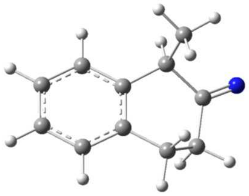

Cartesian Coordinates

C	3.02764800	0.35260100	-0.06446600
C	1.99660000	1.21924900	0.27495200
C	0.67062800	0.79456900	0.25530000
C	0.37654800	-0.52198200	-0.11960900
C	1.41456600	-1.38036300	-0.47537600
C	2.73509500	-0.95136800	-0.44506900
H	-0.84122600	1.54334800	1.58953900
H	4.05524200	0.69776800	-0.04365100
H	2.22087900	2.24405000	0.55478800
C	-0.46995000	1.72131500	0.57302600
C	-1.04722200	-1.02010200	-0.07611600
H	1.18305700	-2.39724600	-0.77804700
H	3.53236900	-1.63094500	-0.72440200
C	-2.05858700	0.06687500	-0.47714000
C	-1.60409400	1.51744500	-0.43055700
H	-1.16109000	-1.84070100	-0.78939500
H	-2.46419200	2.15452300	-0.21917400
H	-1.24265200	1.77480500	-1.43245500
H	-0.13082900	2.75996600	0.54412000
N	-3.21497700	-0.24397700	-0.81842000
C	-1.40408500	-1.55378200	1.32144800
H	-2.42638600	-1.93597000	1.33033000
H	-0.71948000	-2.35831300	1.59993100
H	-1.32373200	-0.77018500	2.07898500

No.	Species	Optimized Structure
46	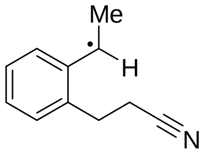	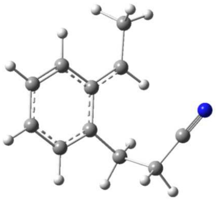

Cartesian Coordinates

No.	Species	Optimized Structure

C	2.41673100	-1.48514200	-0.40608300
C	1.17982200	-1.76644300	0.16485700
C	0.27669900	-0.76623200	0.50000800
C	0.62403900	0.59852600	0.26354700
C	1.89315500	0.85675600	-0.31457800
C	2.76685800	-0.15883900	-0.64519600
H	-0.89421900	-2.08547700	1.68389800
H	3.09883100	-2.28986900	-0.65418600
H	0.91110500	-2.80052500	0.36334500
C	-1.04446000	-1.17573200	1.09628700
C	-0.24386200	1.66836100	0.58437000
H	2.18003100	1.88392800	-0.50552000
H	3.72736000	0.07962000	-1.08866700
C	-2.13289300	-1.50546900	0.05306200
H	-2.99345900	-1.96476900	0.55026100
H	-1.42339600	-0.42250300	1.78989200
C	0.07120600	3.10005300	0.33687600
H	-0.71869600	3.74674500	0.71956000
H	1.01284000	3.40580300	0.80944100
H	0.17583500	3.31172600	-0.73560200
H	-1.74763000	-2.23749400	-0.66299900
C	-2.62144100	-0.35574500	-0.69250900
N	-3.03175000	0.55253000	-1.27176400
H	-1.22148300	1.44453800	0.98907400

No.	Species	Optimized Structure
47	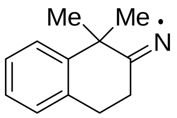	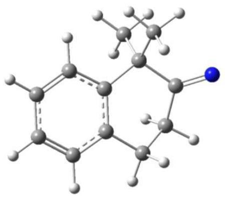

Cartesian Coordinates

C	3.18616800	0.19967900	0.08826000
C	2.24989000	1.21928500	0.12229500
C	0.87873500	0.95508100	0.08276300
C	0.43750700	-0.37305600	0.00554700
C	1.39515300	-1.39160000	-0.03695600
C	2.75278200	-1.11830300	0.00497100
H	-0.27963600	2.39389800	1.16839400
H	4.24518500	0.42973200	0.11929700
H	2.58066500	2.25210300	0.17766300
C	-0.09155900	2.10681800	0.12660100
C	-1.04276600	-0.76069000	-0.04434100
H	1.07157500	-2.42456300	-0.10510700
H	3.46947800	-1.93114200	-0.02957100

No.	Species	Optimized Structure

C -1.93444600 0.48242200 0.09358900
C -1.41711800 1.75608800 -0.53708500
H -2.15518000 2.54962600 -0.41618700
H -1.26676400 1.58683700 -1.60882000
H 0.35294600 2.98022800 -0.35957400
N -3.02334000 0.46373300 0.69843300
C -1.38830800 -1.75564700 1.07070300
H -2.45558900 -1.98470800 1.05119300
H -0.83415500 -2.68805100 0.94679100
H -1.14908900 -1.34079900 2.05156100
C -1.37046100 -1.38887000 -1.41349100
H -1.16016600 -0.70009100 -2.23481800
H -0.76454700 -2.28452200 -1.57090200
H -2.42480100 -1.67192000 -1.45609100

48	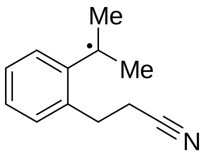	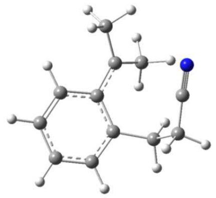

Cartesian Coordinates
C -3.00212100 -0.61473300 0.22392800
C -1.92918600 -1.30363200 -0.32388600
C -0.68242100 -0.71271900 -0.52312700
C -0.50024700 0.65882300 -0.18477300
C -1.59832700 1.32177500 0.41412000
C -2.82080300 0.70919000 0.60916800
H 0.00913100 -2.47964900 -1.48319800
H -3.95846400 -1.10663500 0.35786500
H -2.05384900 -2.34922800 -0.59258100
C 0.43848500 -1.63274100 -0.94127900
C 0.70489300 1.41390300 -0.41682100
H -1.48626500 2.36430100 0.68704400
H -3.63870000 1.26956600 1.04871900
C 1.20988700 -2.22478200 0.25866700
H 1.86107500 -3.03544200 -0.08447900
H 1.15127800 -1.16284400 -1.61518400
C 1.07759200 2.55100700 0.47473000
H 2.15447900 2.52028400 0.67417000
H 0.87399400 3.52628100 0.00780600
H 0.56603800 2.51941000 1.43694400
C 1.60410500 1.20910100 -1.59103300
H 1.10820500 0.70771800 -2.42397800
H 1.95172700 2.18252500 -1.95522400

No.	Species	Optimized Structure
H	2.51133200 0.64452600 -1.33140900	
H	0.50693800 -2.65703200 0.97693400	
C	2.04673800 -1.26333000 0.96046000	
N	2.73593100 -0.51642300 1.50509300	

8 NMR Spectra

S3 ^{1}H NMR (500 MHz, $CDCl_3$)

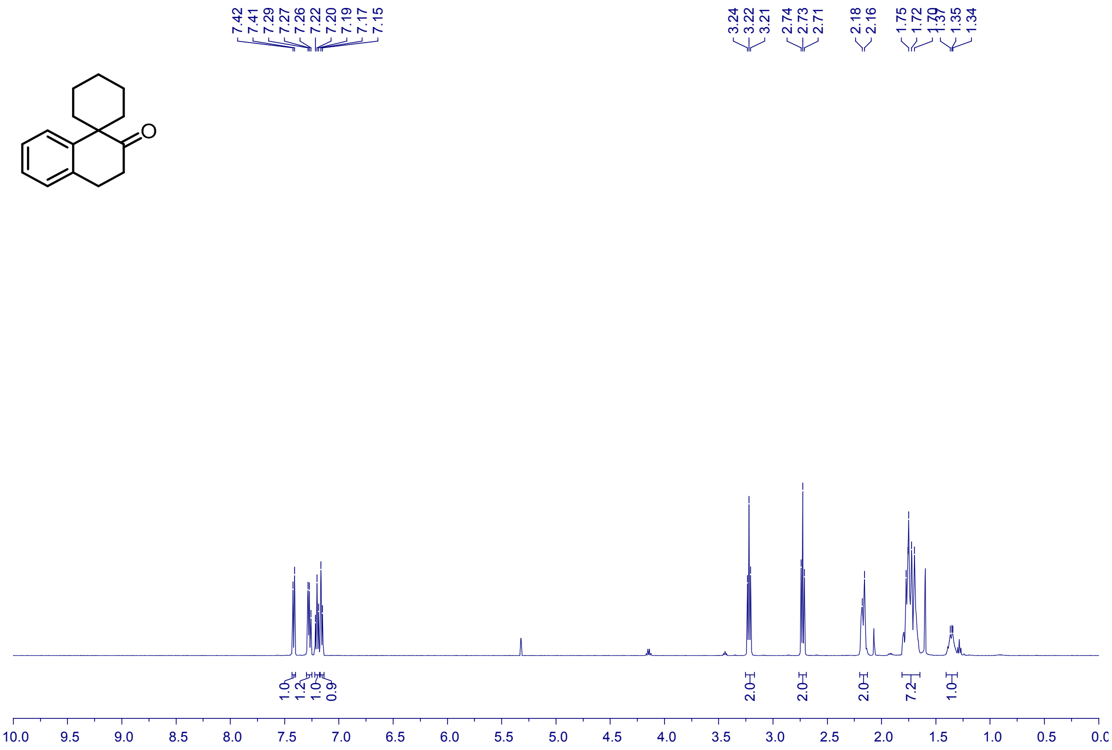

S3 ^{13}C NMR (126 MHz, $CDCl_3$)

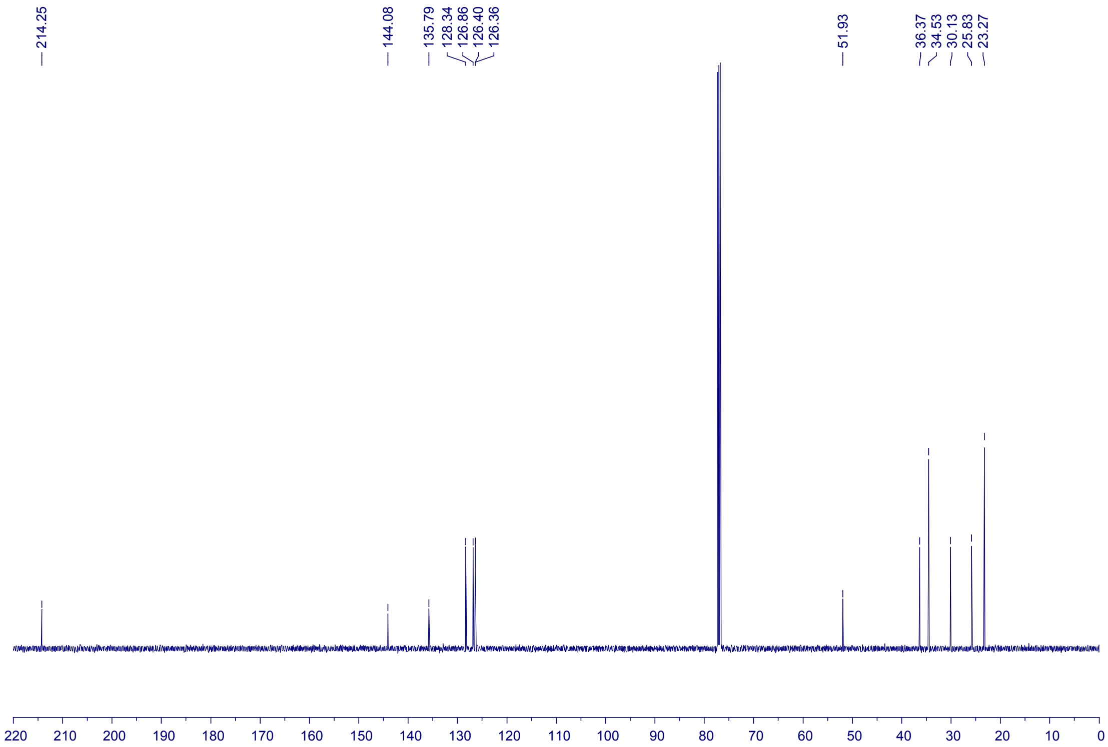

S4 ^{1}H NMR (400 MHz, $CDCl_3$)

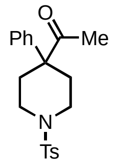

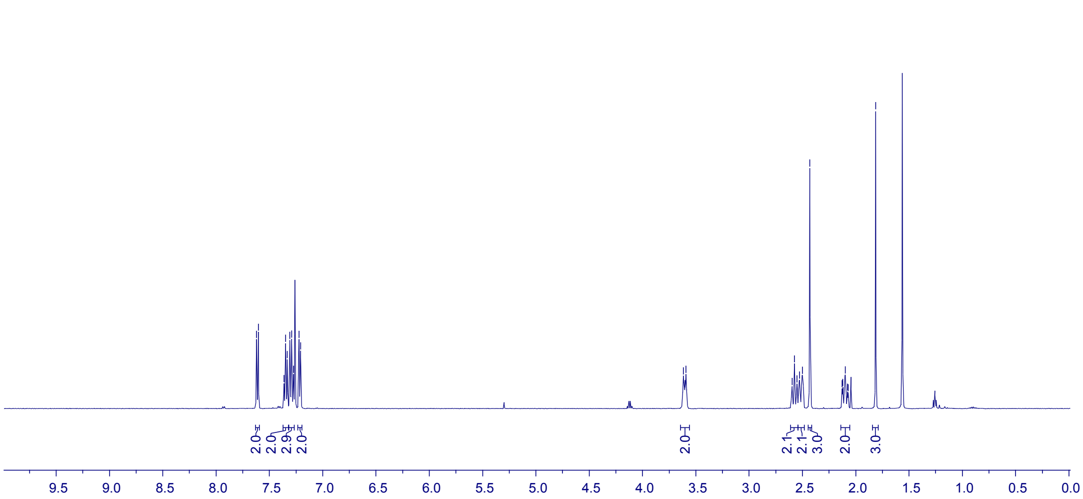

S4 ^{13}C NMR (126 MHz, $CDCl_3$)

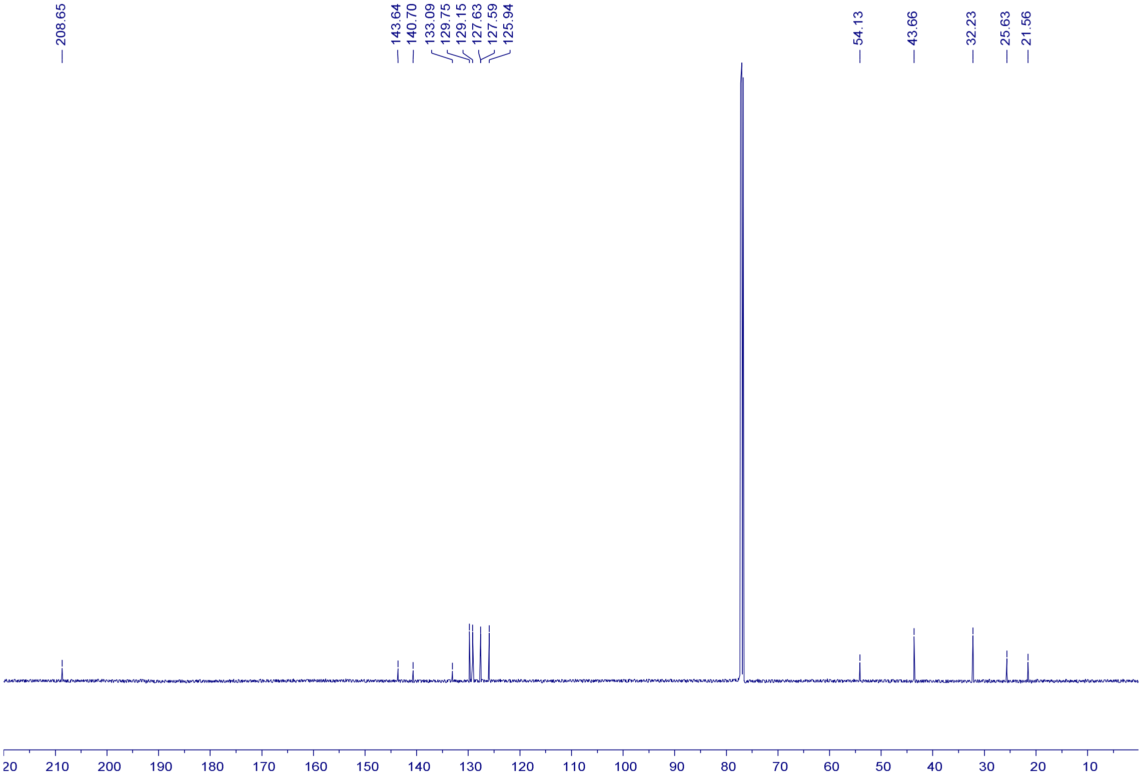

S5 ^{1}H NMR (400 MHz, $CDCl_3$)

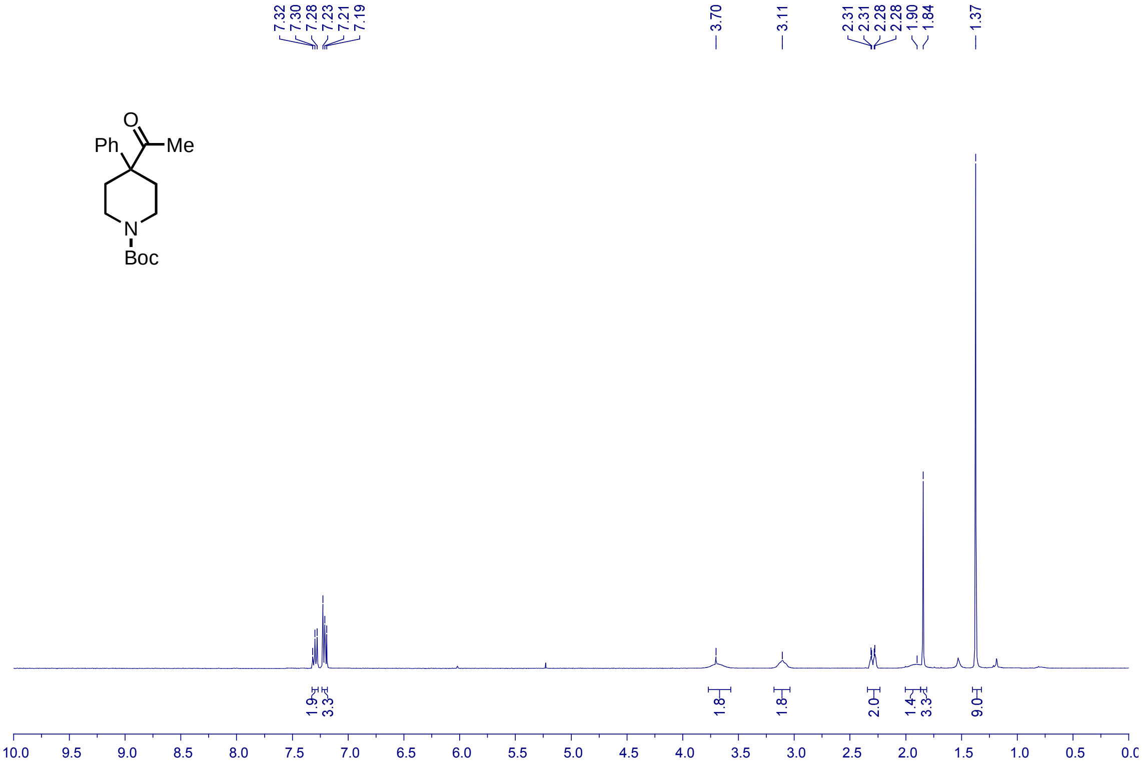

S5 ^{13}C NMR (101 MHz, $CDCl_3$)

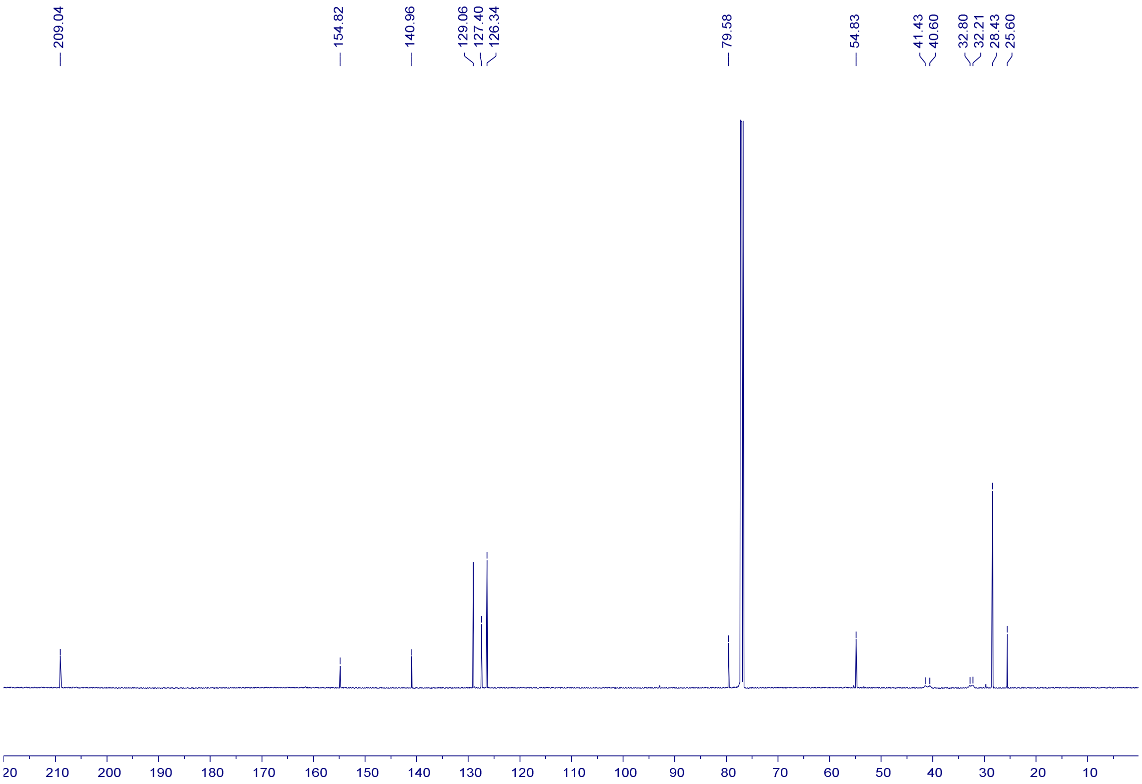

S7 ^{1}H NMR (500 MHz, CDCl$_3$)

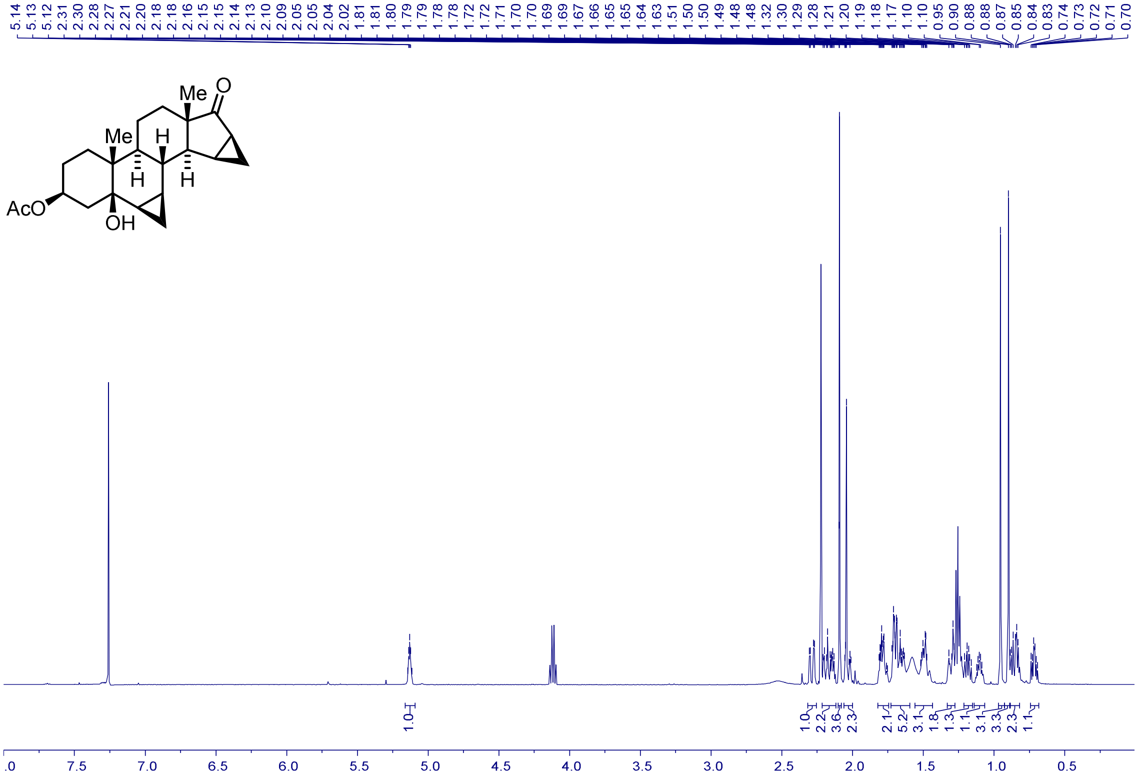

S7 ^{13}C NMR (126 MHz, CDCl$_3$)

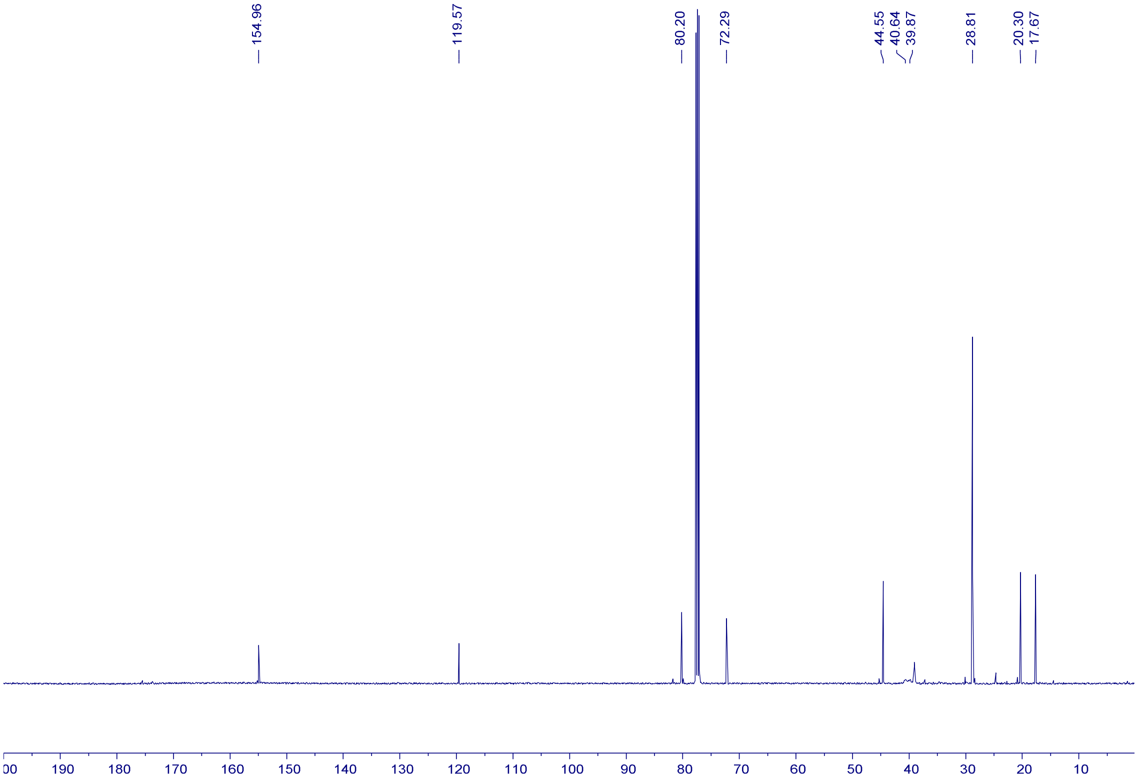

S12 ^{1}H NMR (400 MHz, $CDCl_3$)

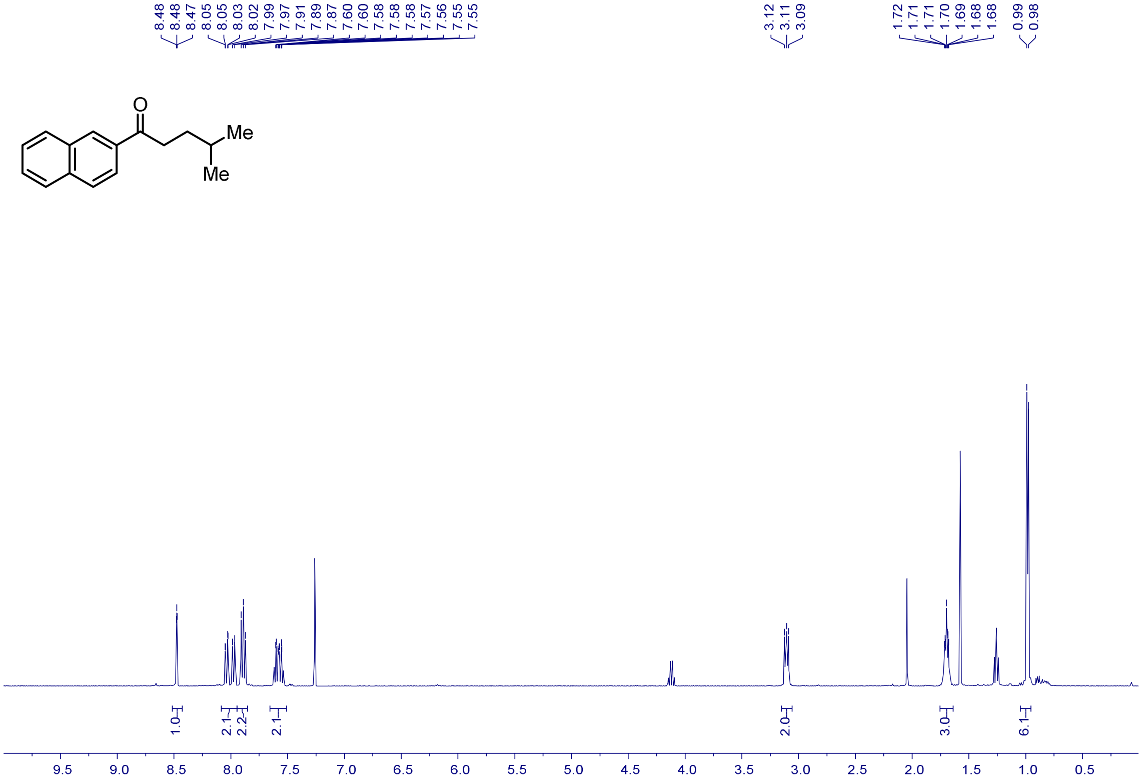

S12 ^{13}C NMR (101 MHz, $CDCl_3$)

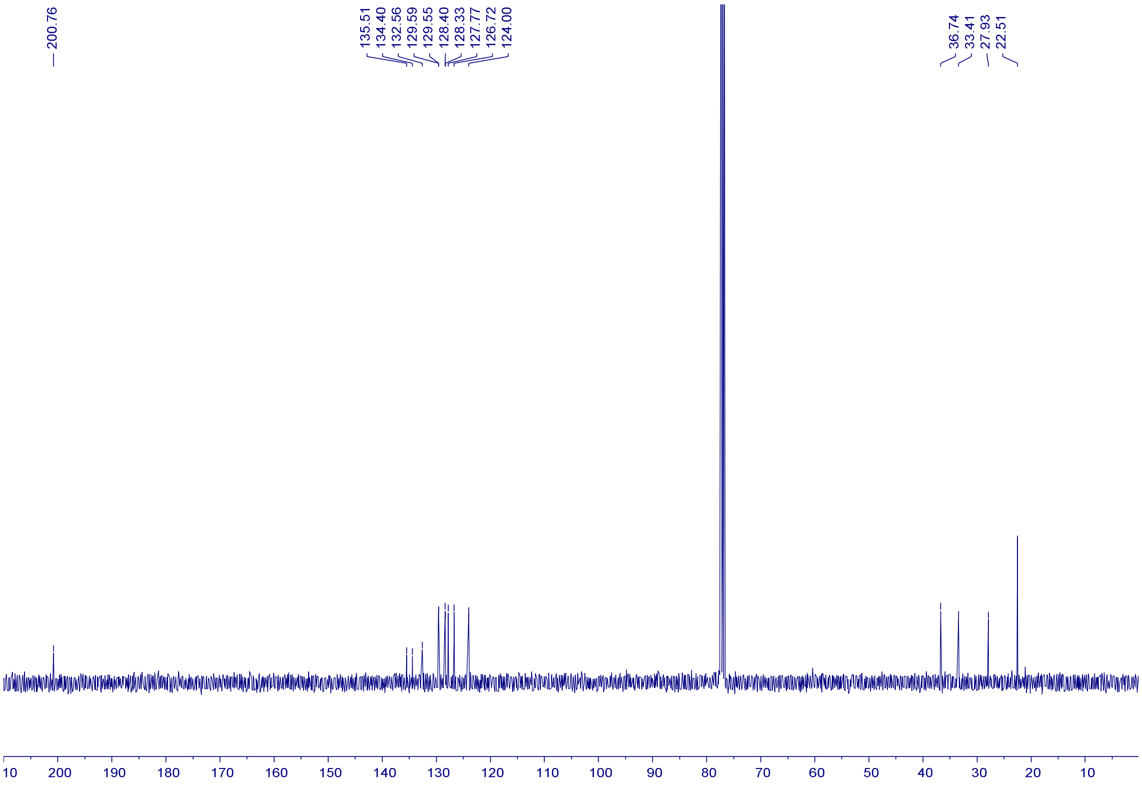

S14 ^{1}H NMR (500 MHz, $CDCl_3$)

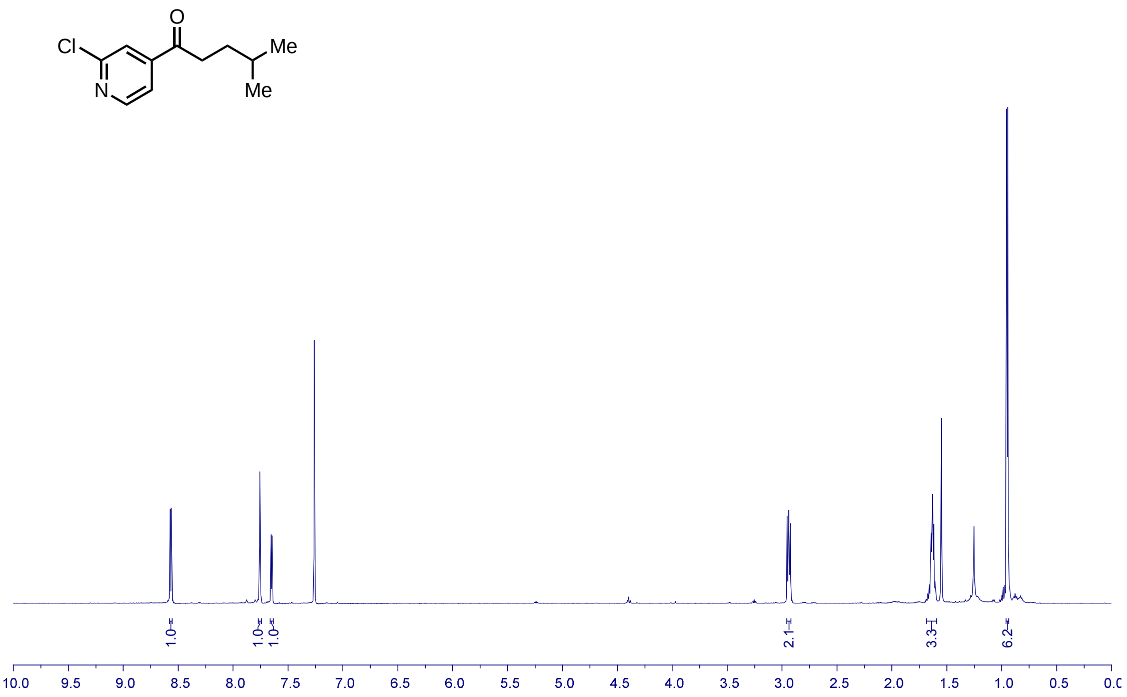

S14 ^{13}C NMR (101 MHz, $CDCl_3$)

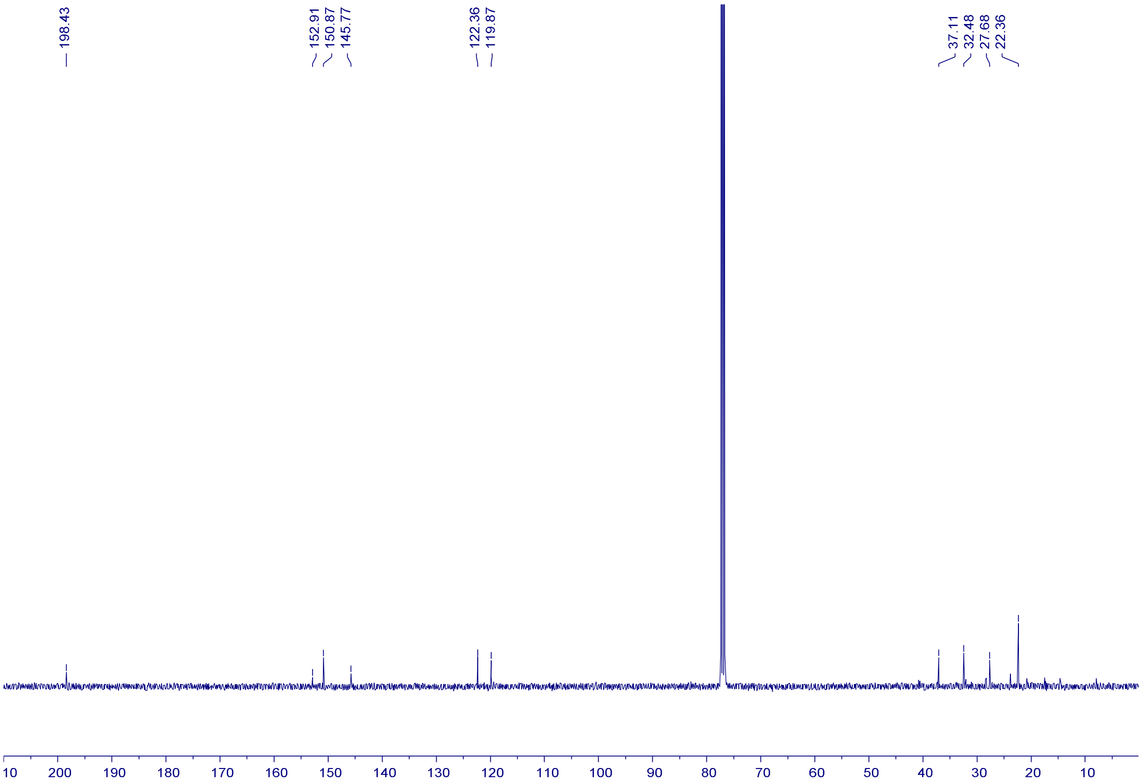

S15 ^{1}H NMR (500 MHz, $CDCl_3$)

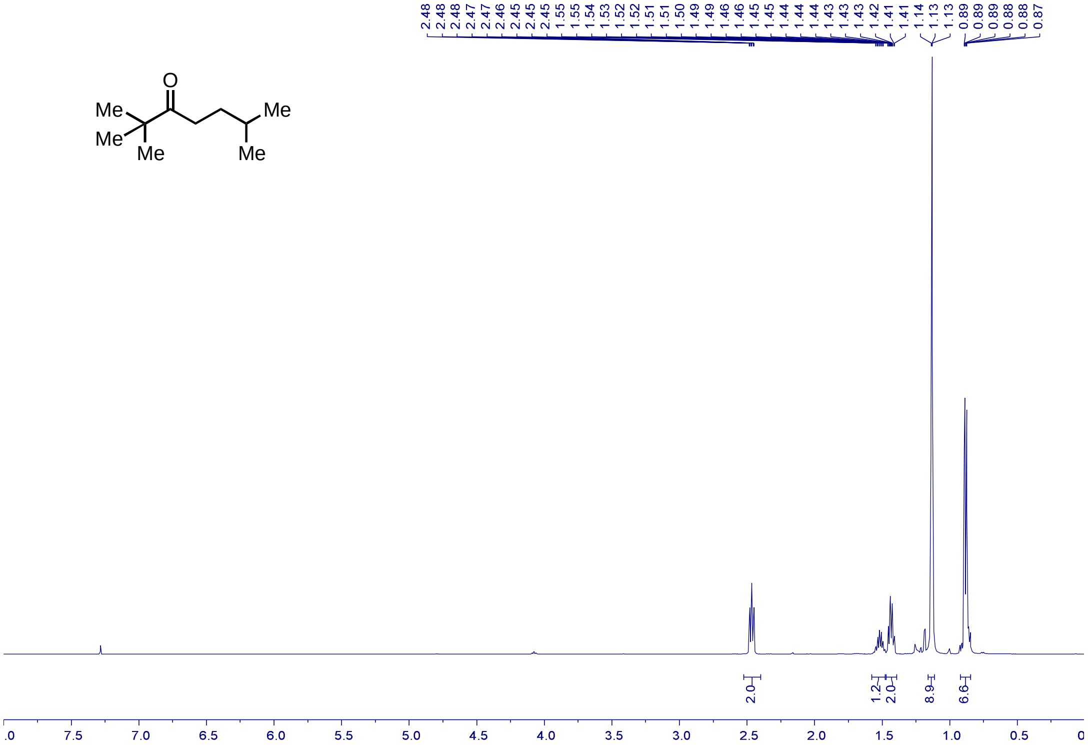

S15 ^{13}C NMR (126 MHz, $CDCl_3$)

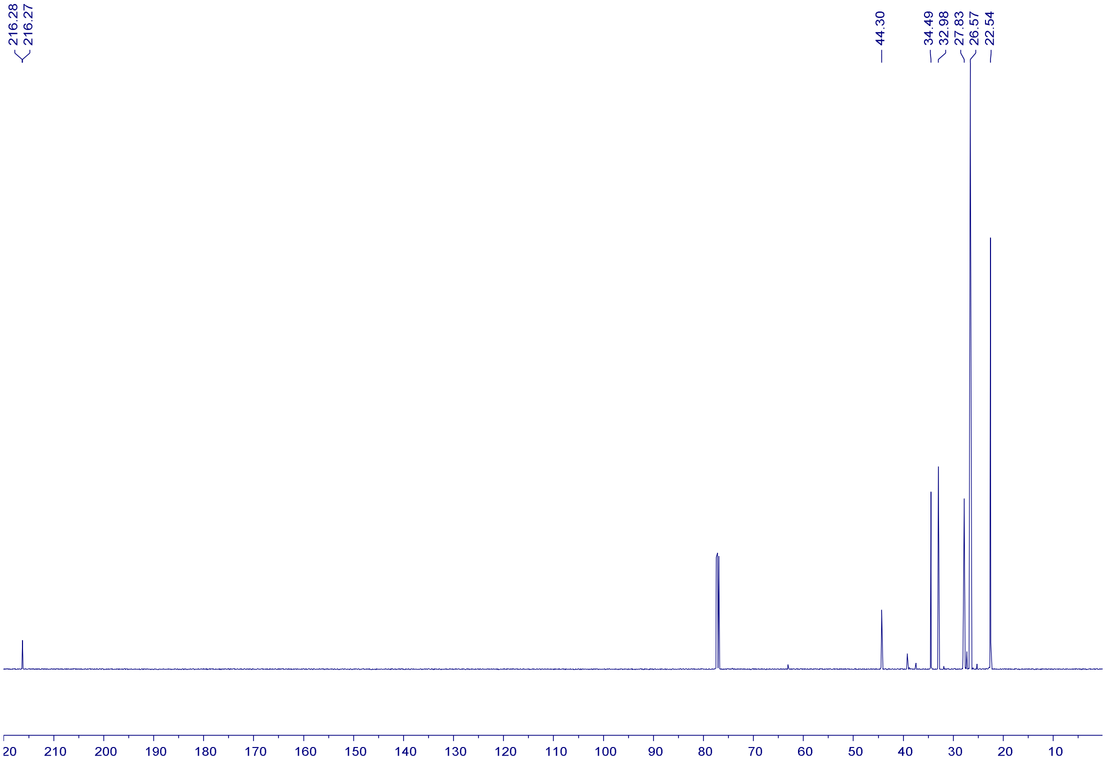

S16 ^{1}H NMR (400 MHz, $CDCl_3$)

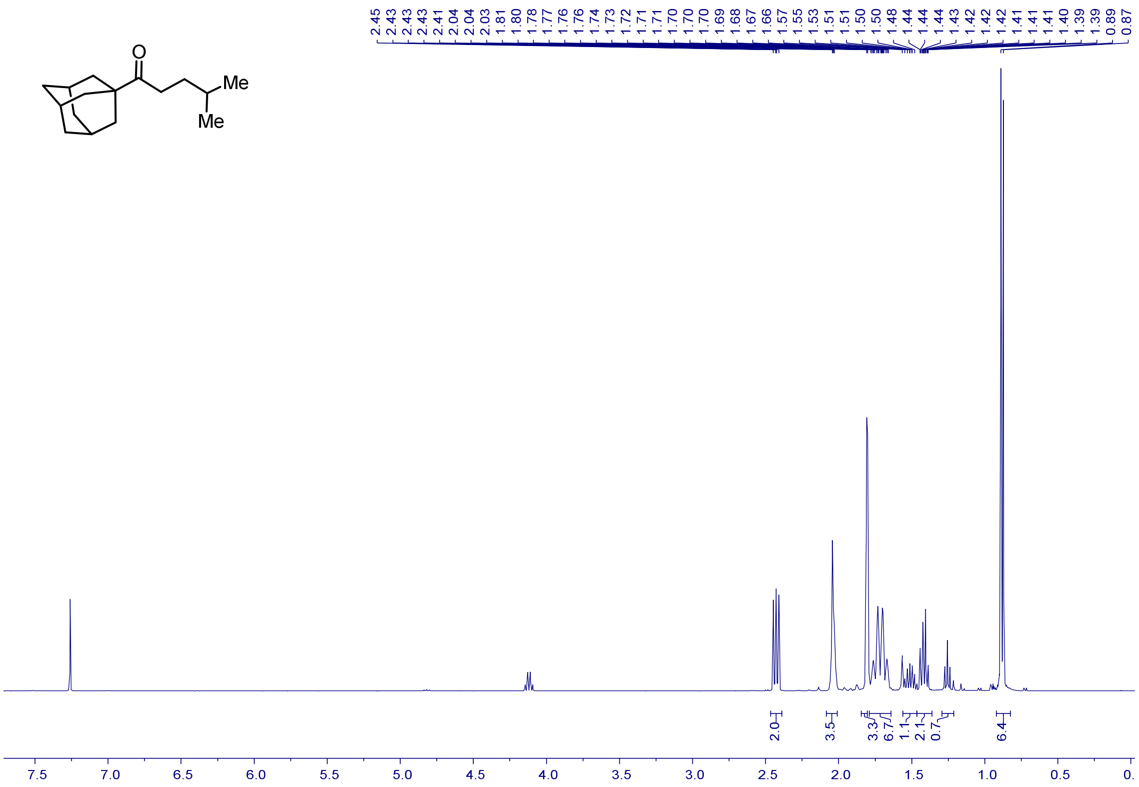

S16 ^{13}C NMR (101 MHz, $CDCl_3$)

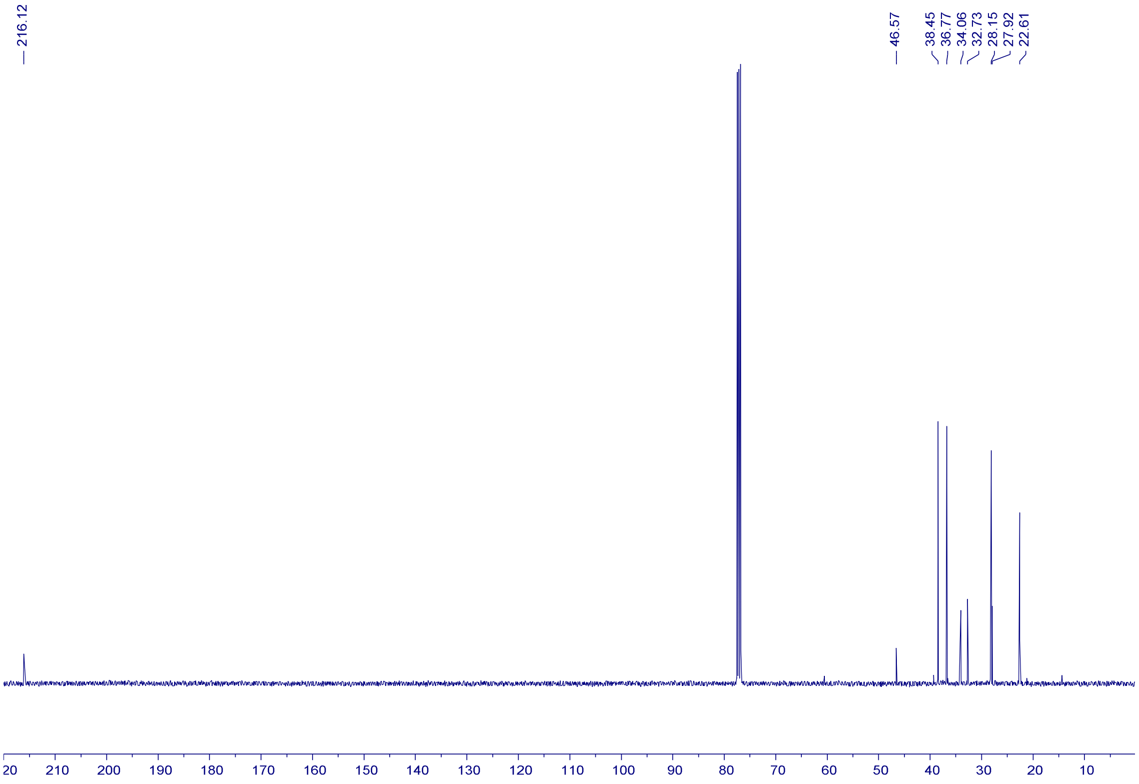

S18 ^{1}H NMR (400 MHz, $CDCl_3$)

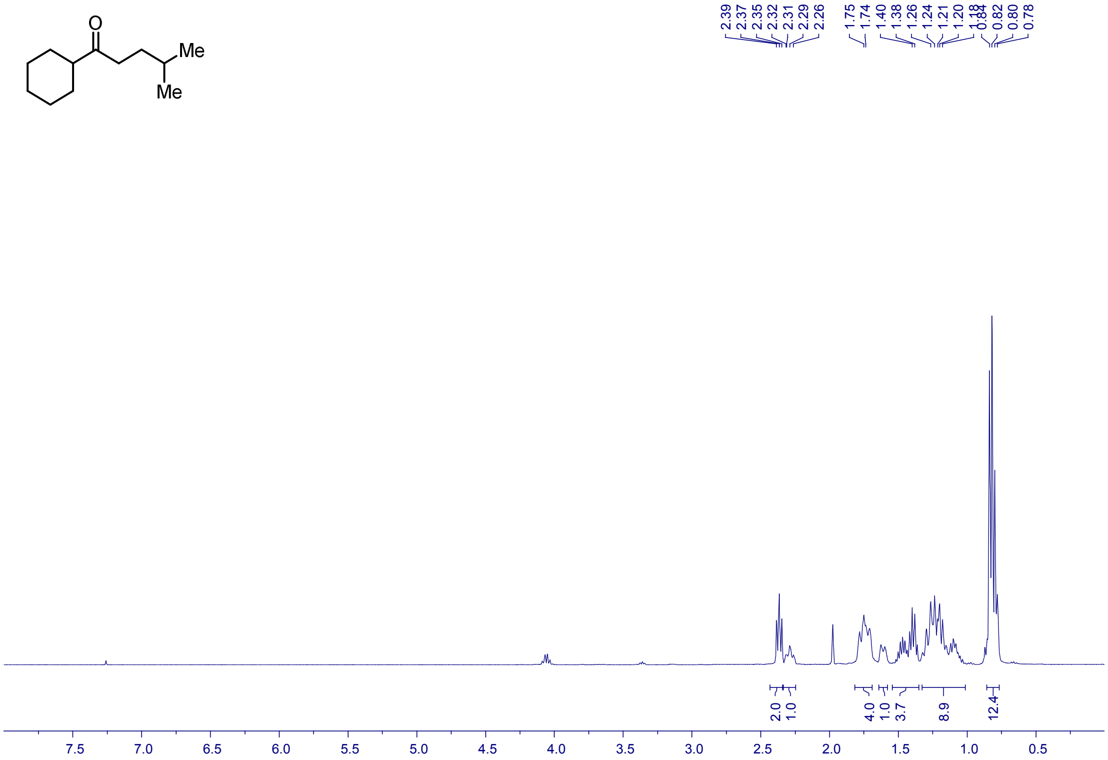

S18 ^{13}C NMR (101 MHz, $CDCl_3$)

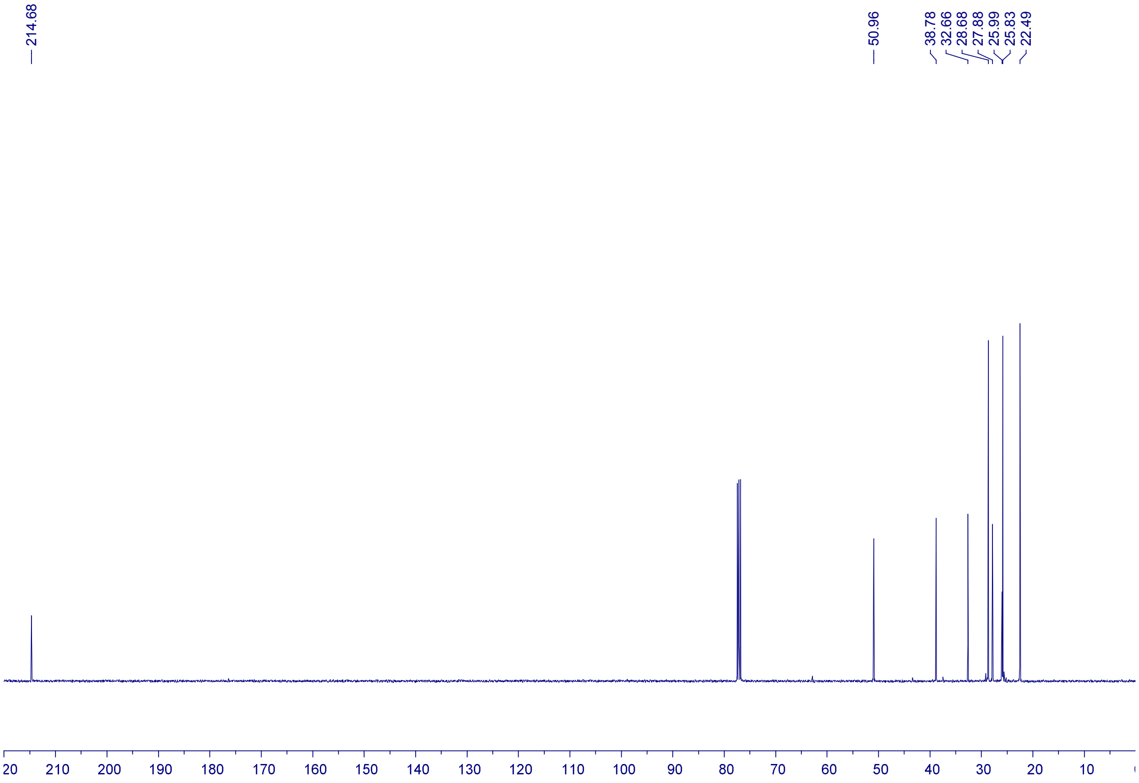

S19 ^{1}H NMR (400 MHz, $CDCl_3$)

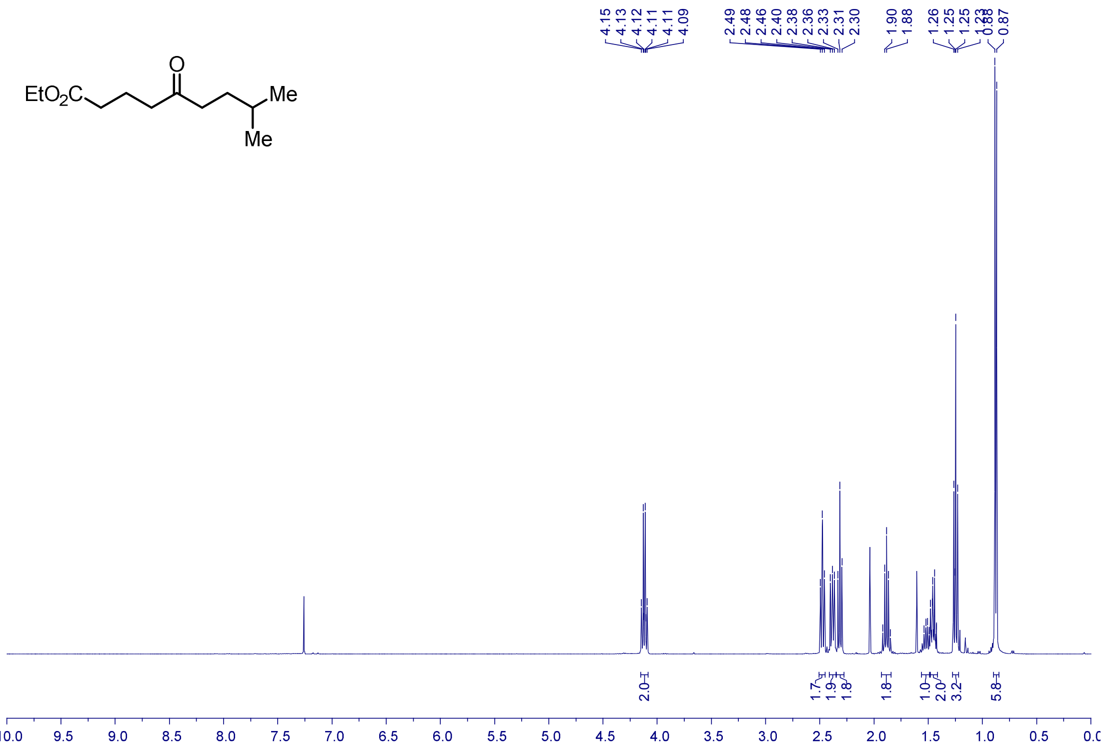

S19 ^{13}C NMR (101 MHz, $CDCl_3$)

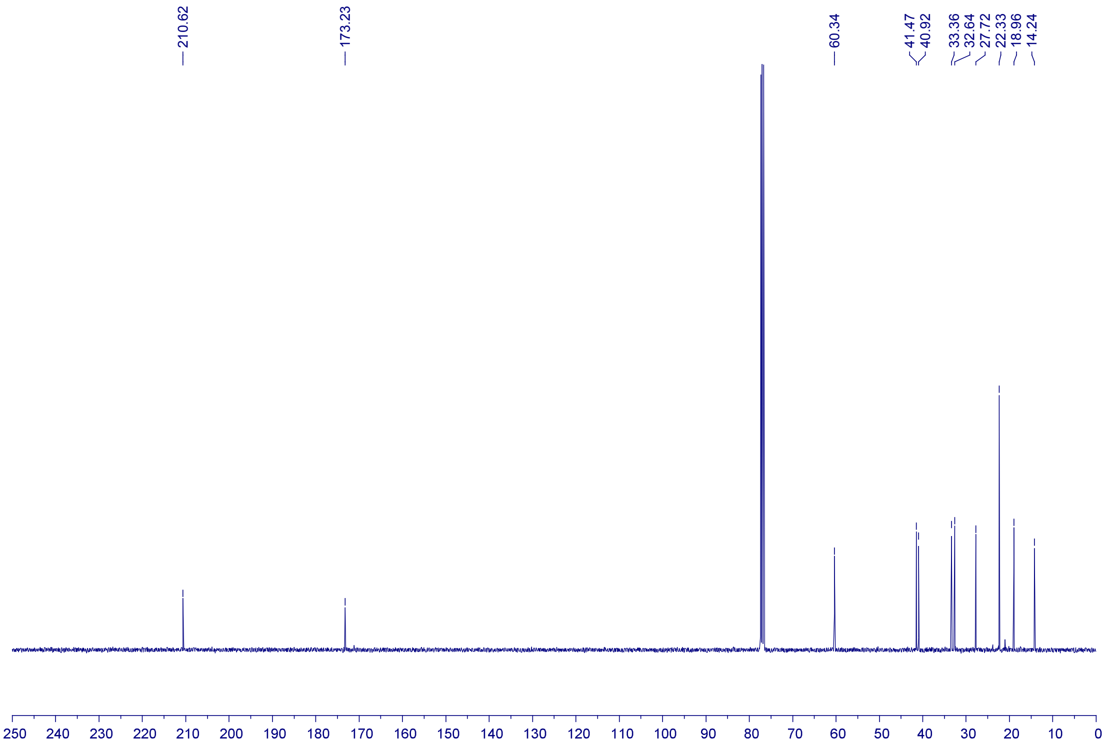

S20 ^{1}H NMR (500 MHz, $CDCl_3$)

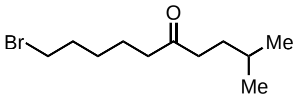

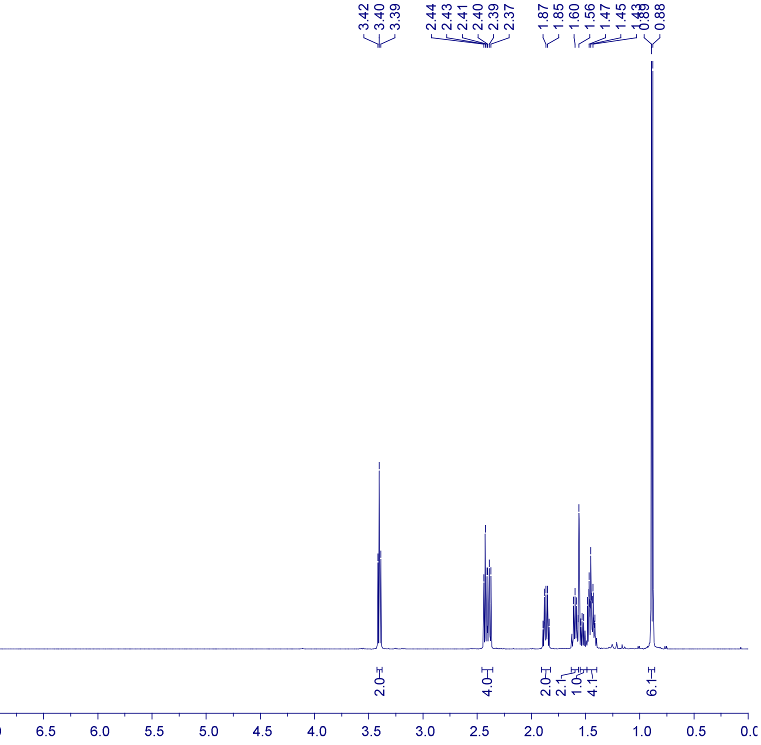

S20 ^{13}C NMR (126 MHz, $CDCl_3$)

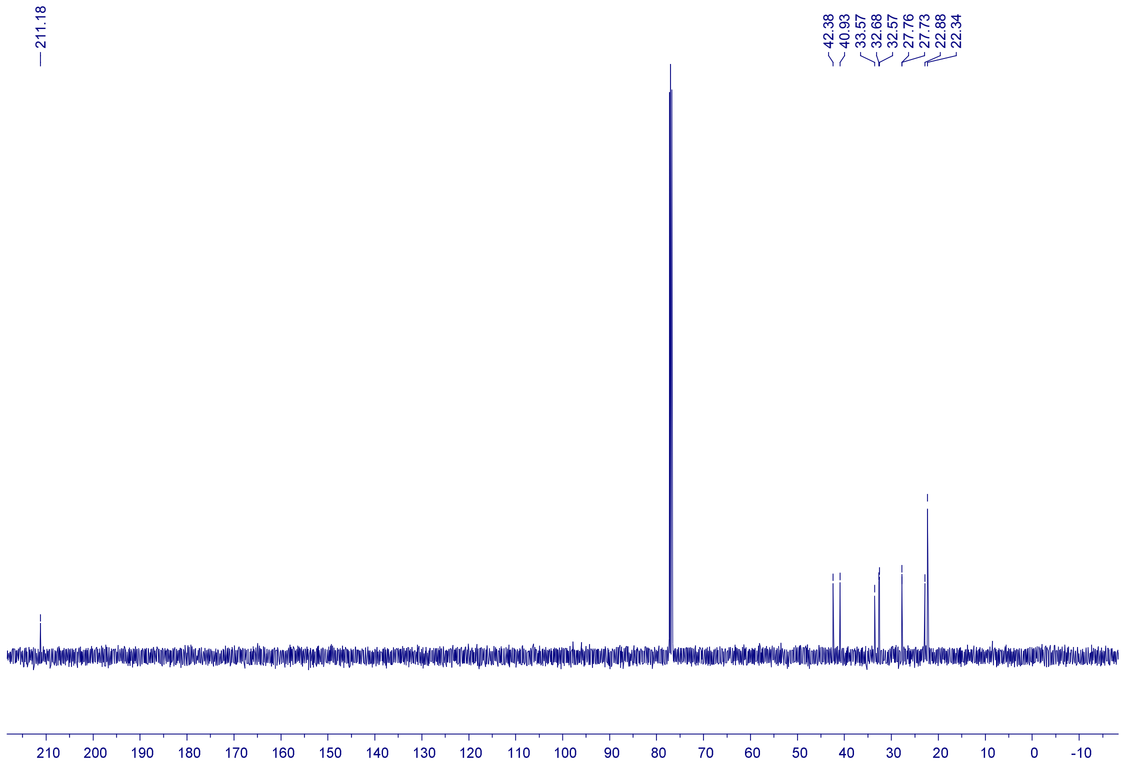

S22 ^{1}H NMR (400 MHz, $CDCl_3$)

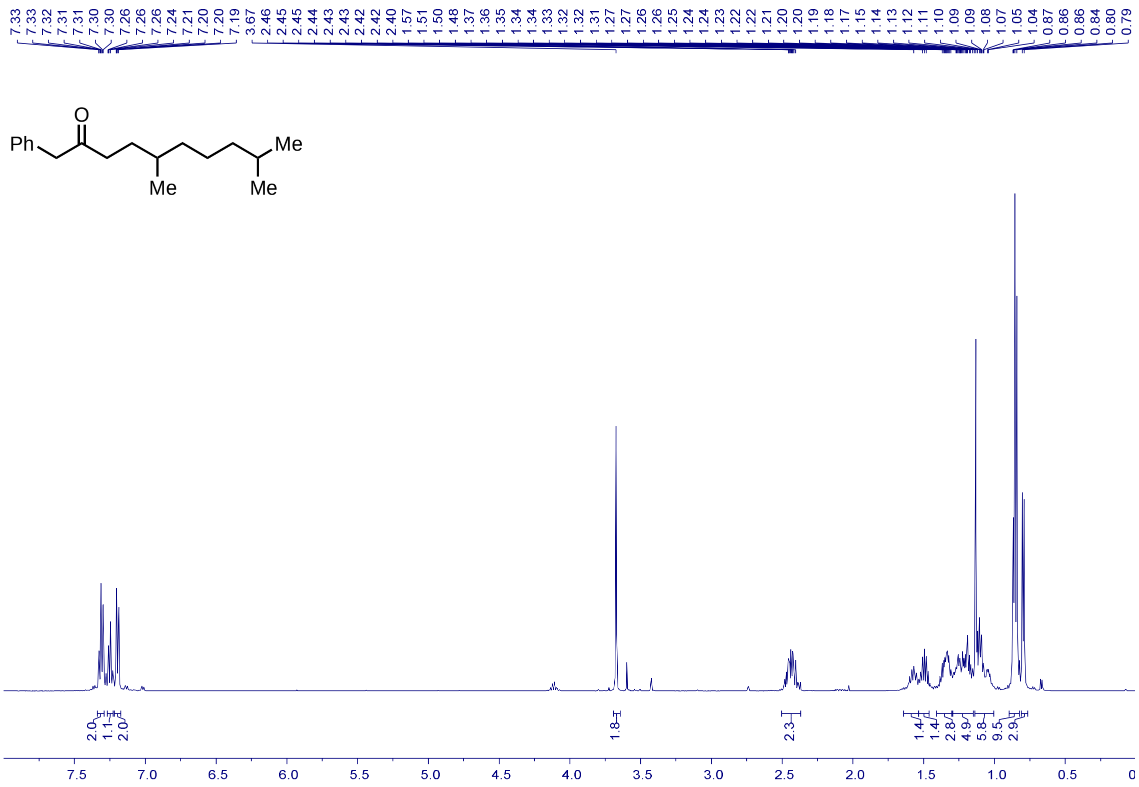

S22 ^{13}C NMR (101 MHz, $CDCl_3$)

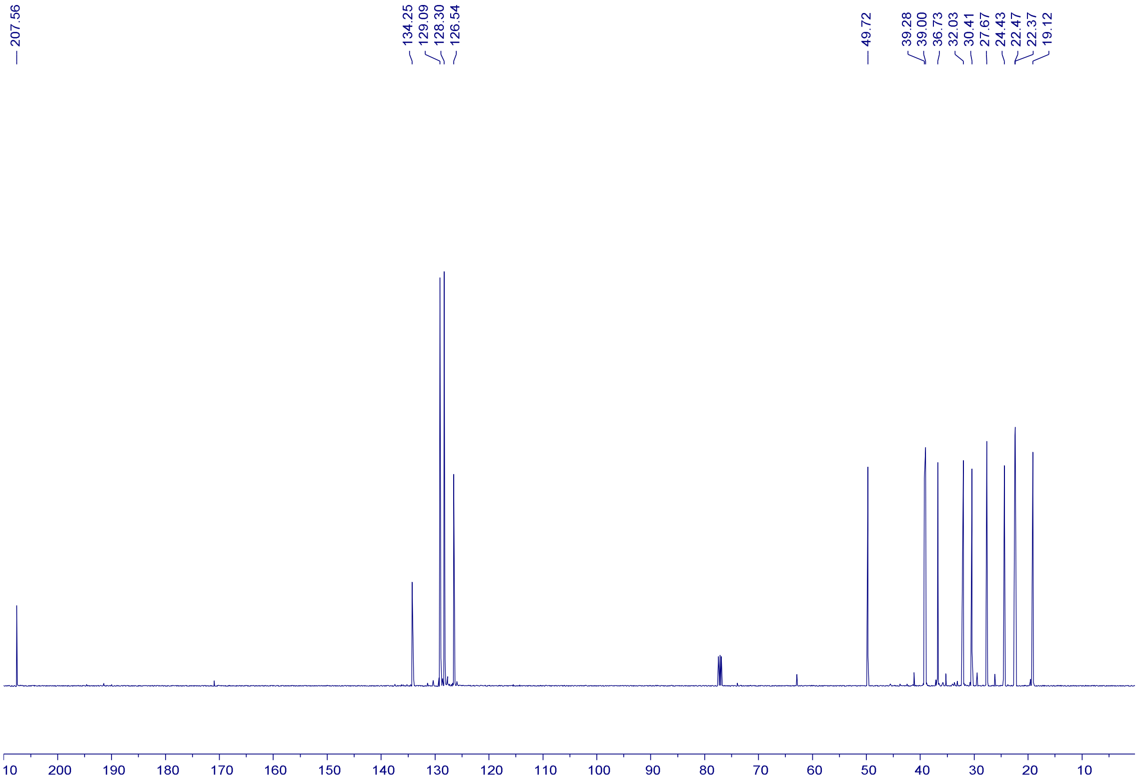

S23 ^{1}H NMR (500 MHz, $CDCl_3$)

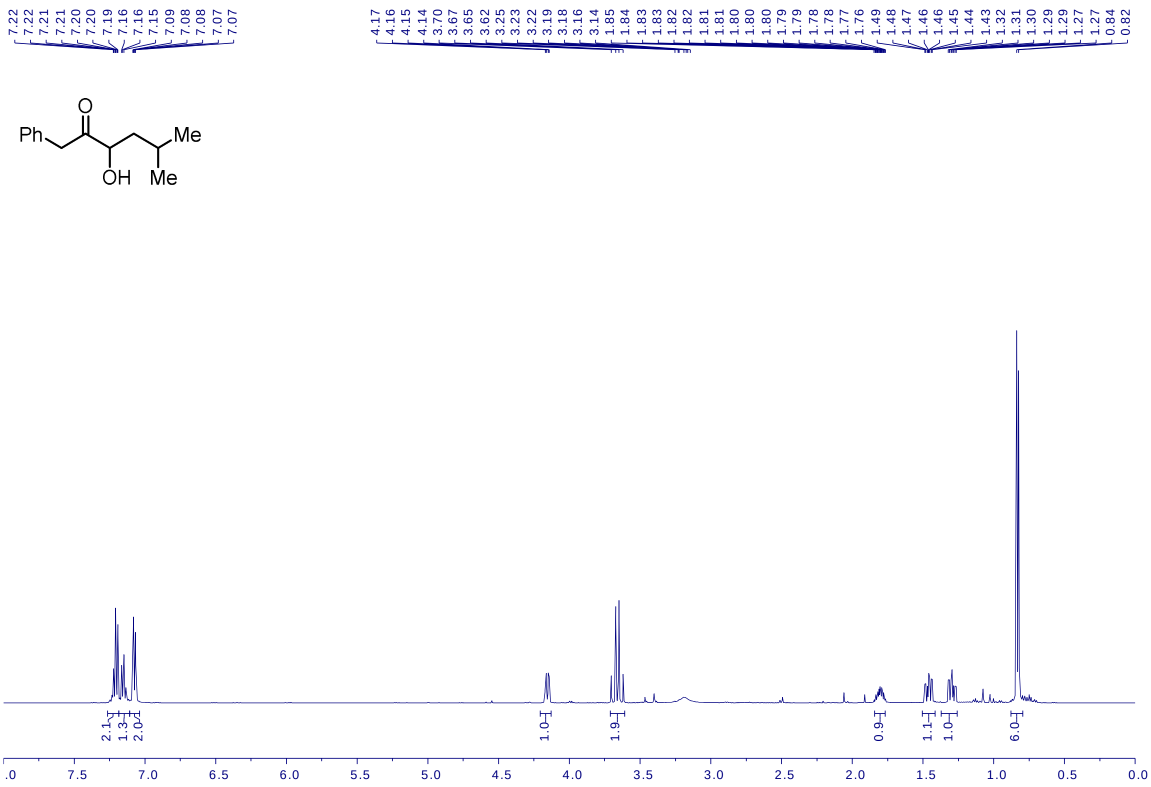

S23 ^{13}C NMR (126 MHz, $CDCl_3$)

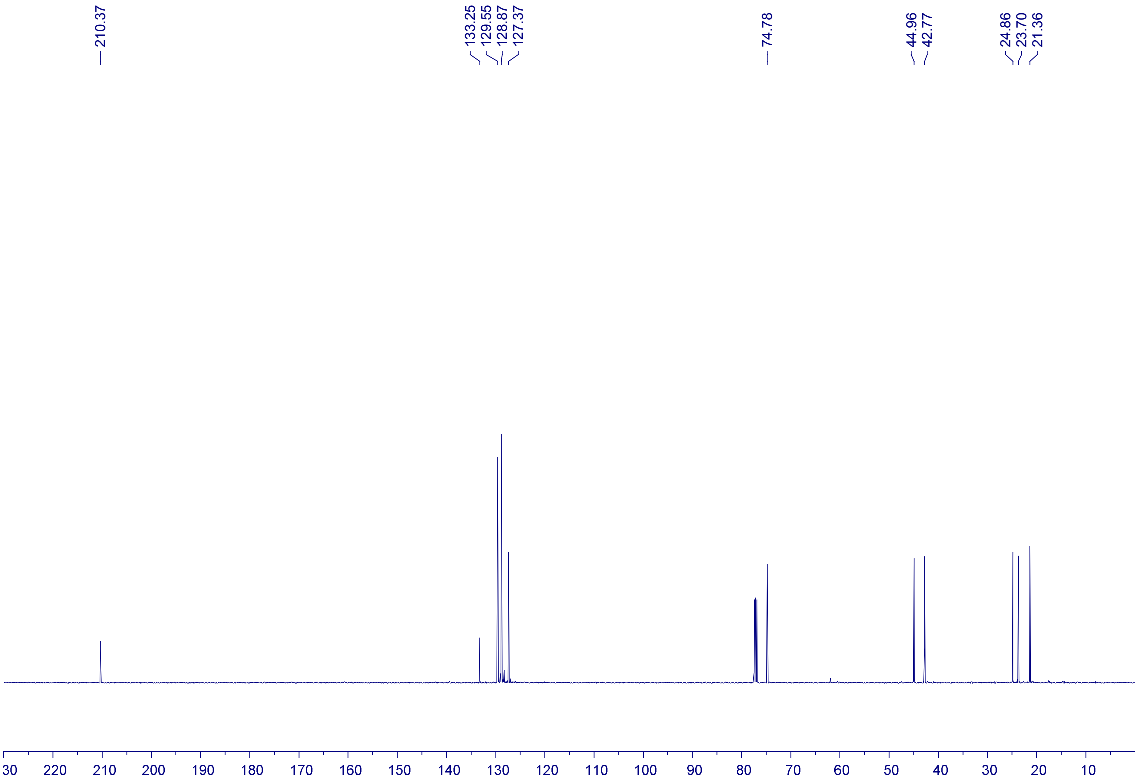

S24 ^{1}H NMR (500 MHz, $CDCl_3$)

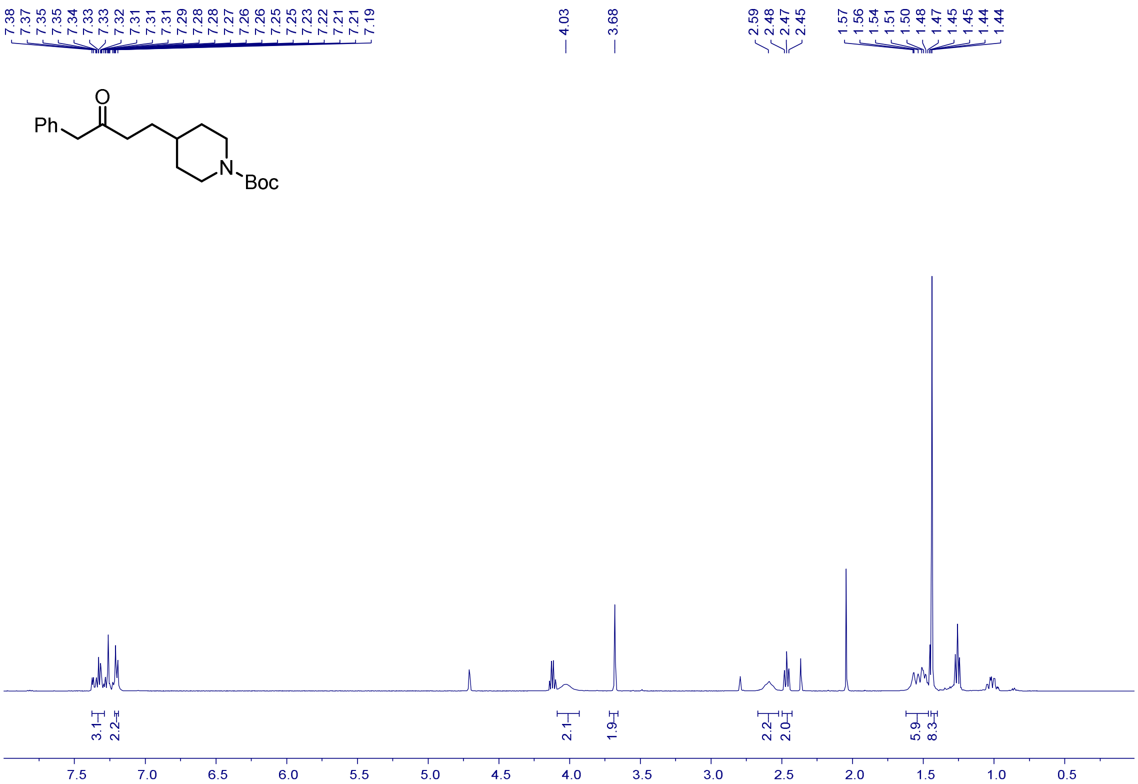

S24 ^{13}C NMR (126 MHz, $CDCl_3$)

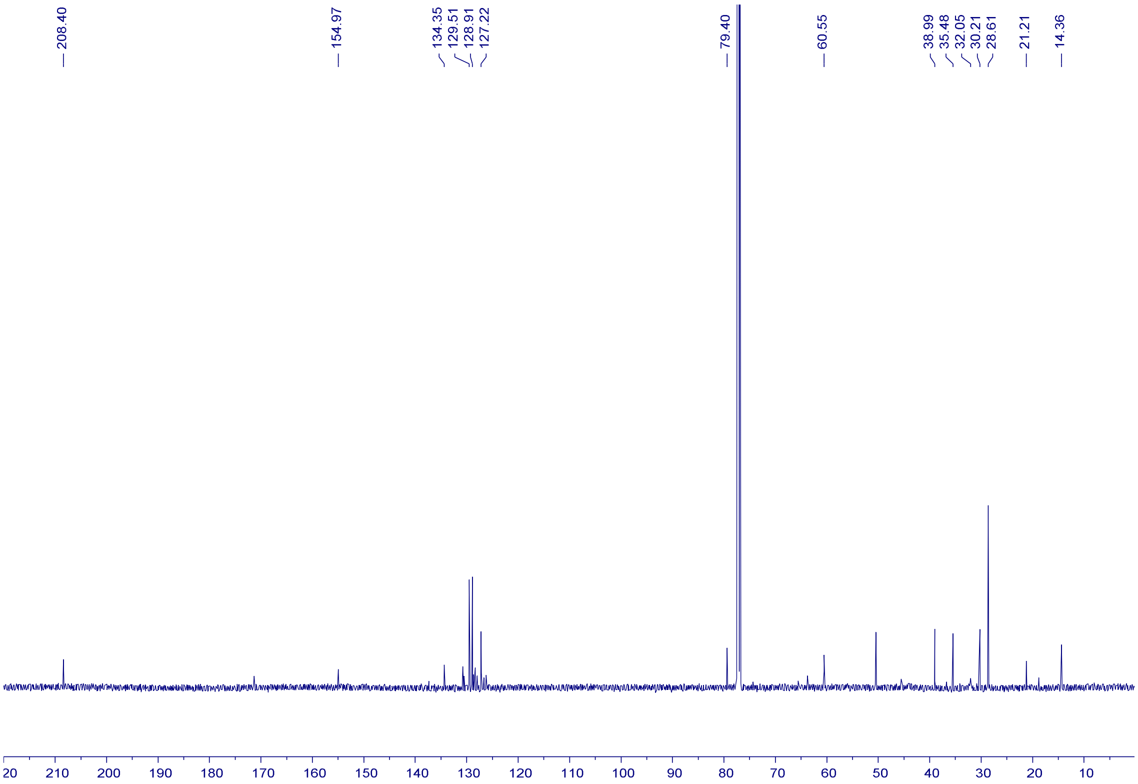

S25 ^{1}H NMR (400 MHz, $CDCl_3$)

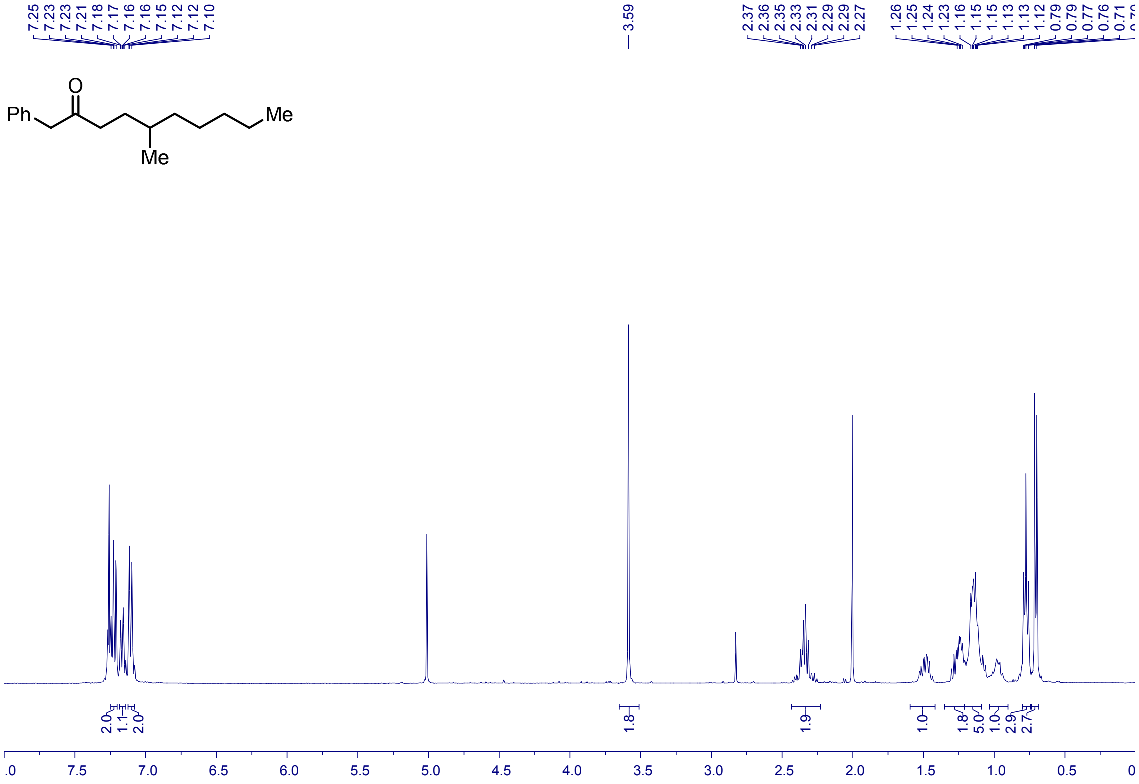

S25 ^{13}C NMR (101 MHz, $CDCl_3$)

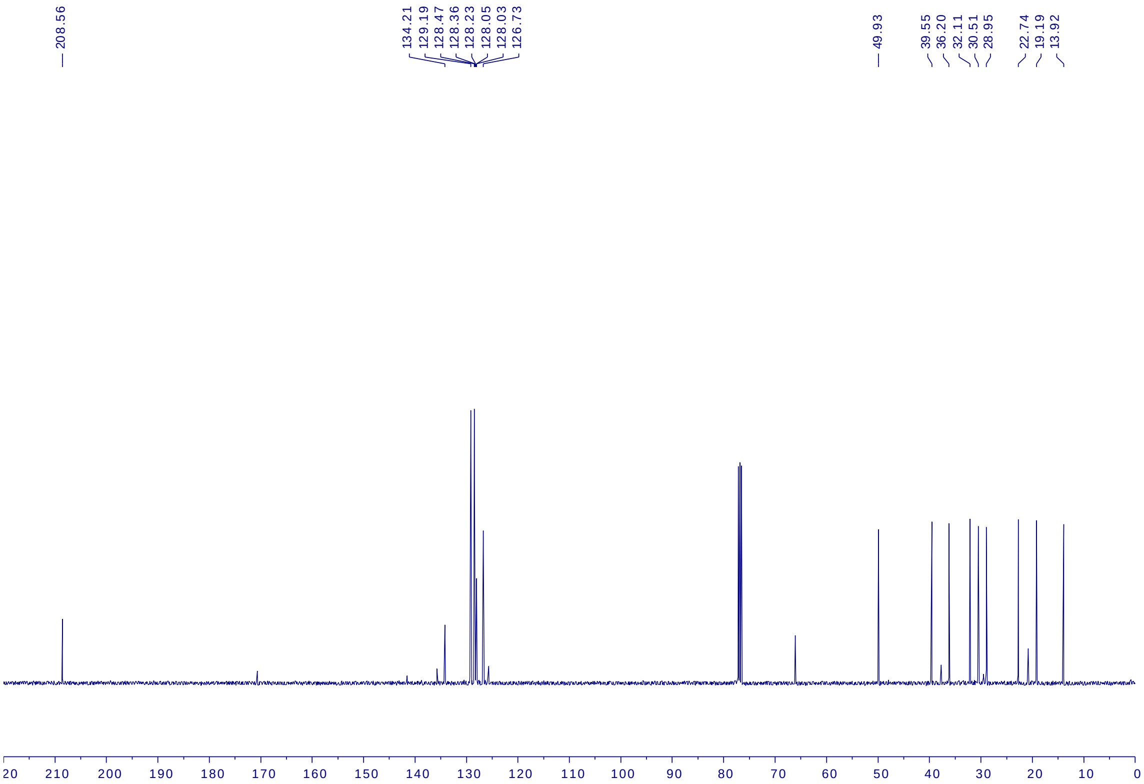

S26 ^{1}H NMR (400 MHz, $CDCl_3$)

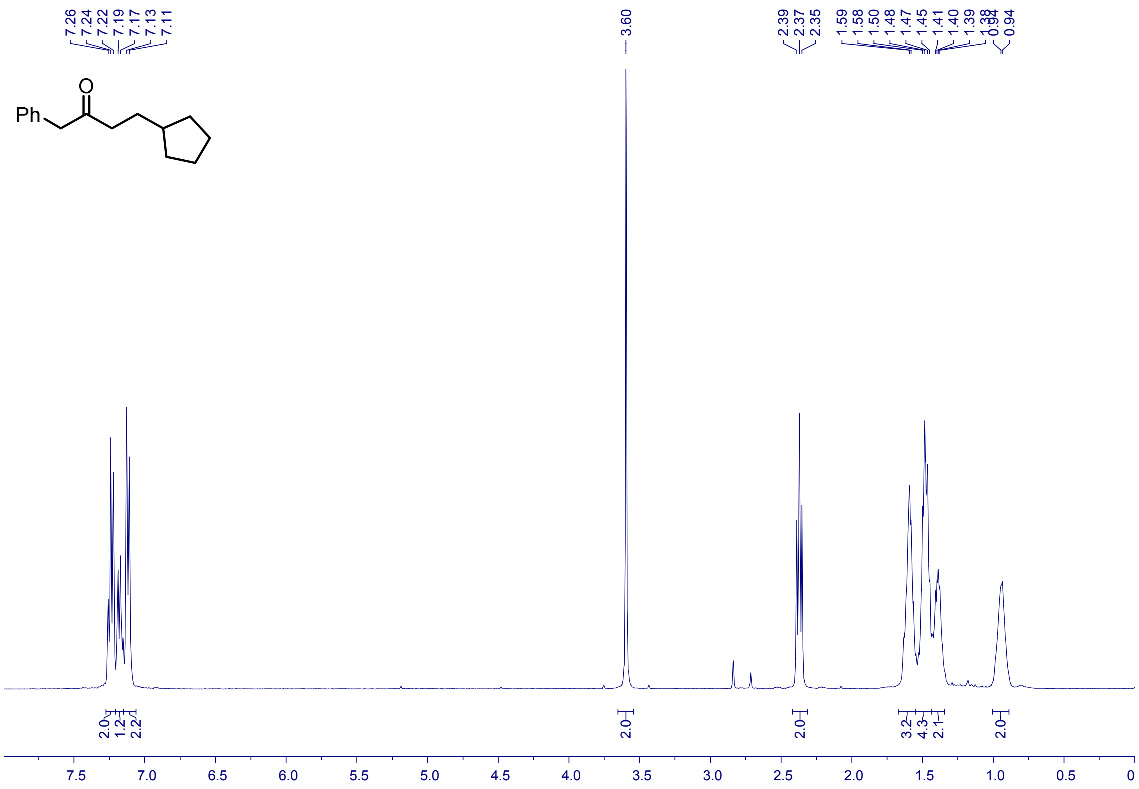

S26 ^{13}C NMR (126 MHz, $CDCl_3$)

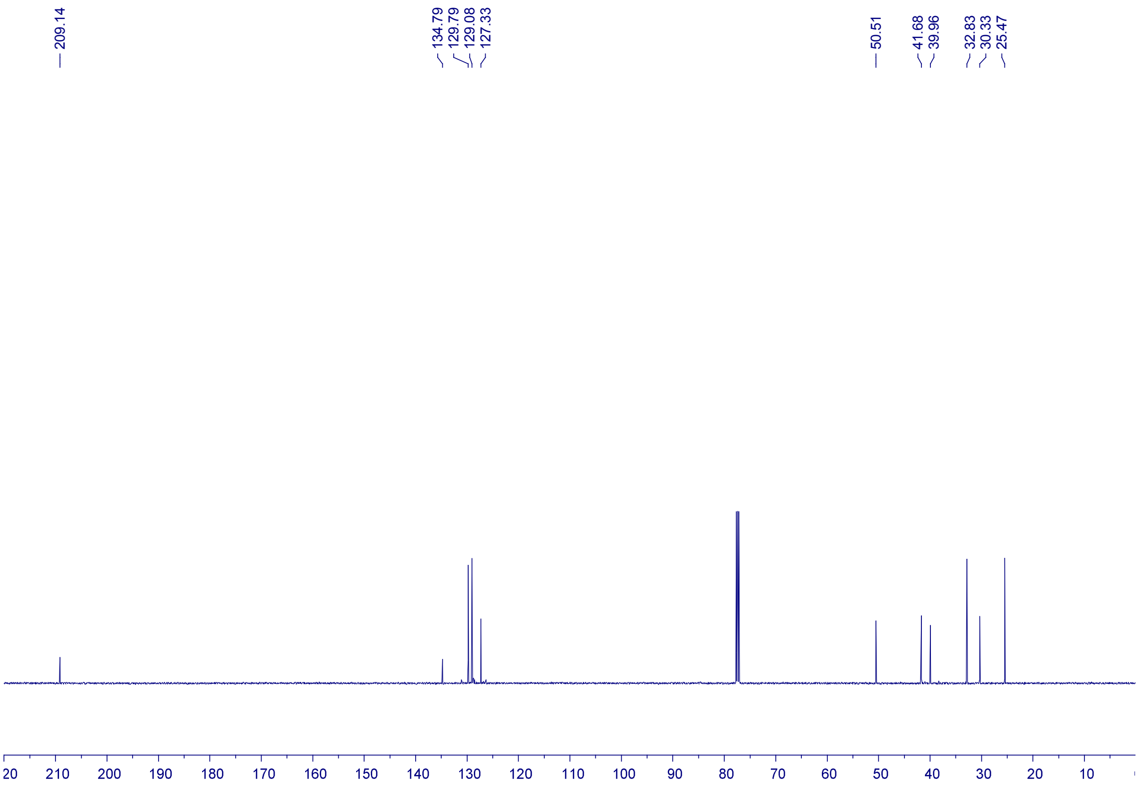

S27 ^{1}H NMR (400 MHz, $CDCl_3$)

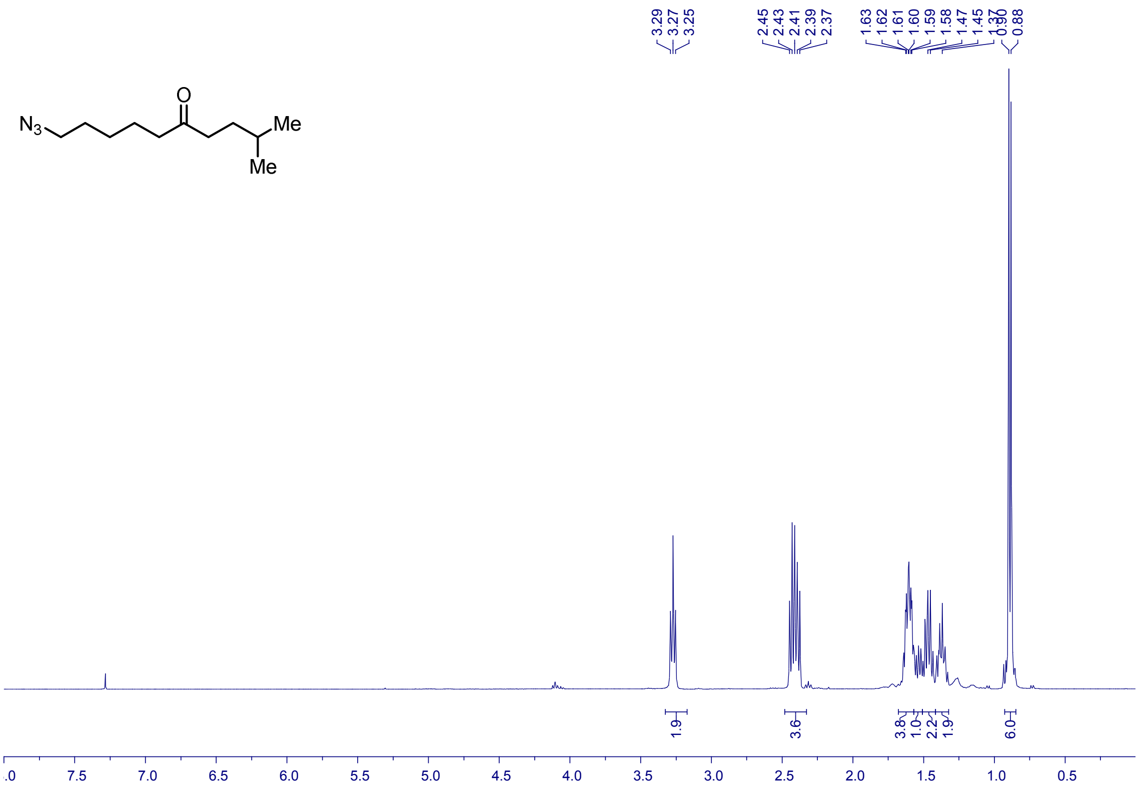

S27 ^{13}C NMR (101 MHz, $CDCl_3$)

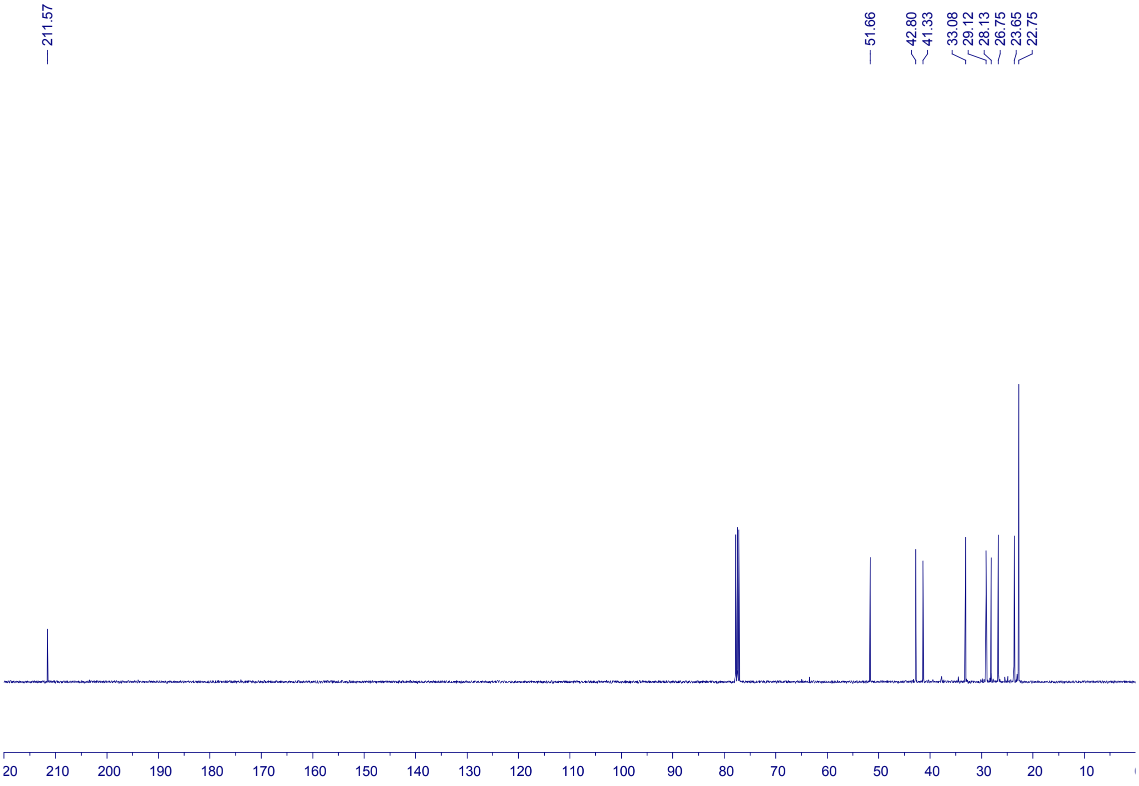

S28 ^{1}H NMR (500 MHz, $CDCl_3$)

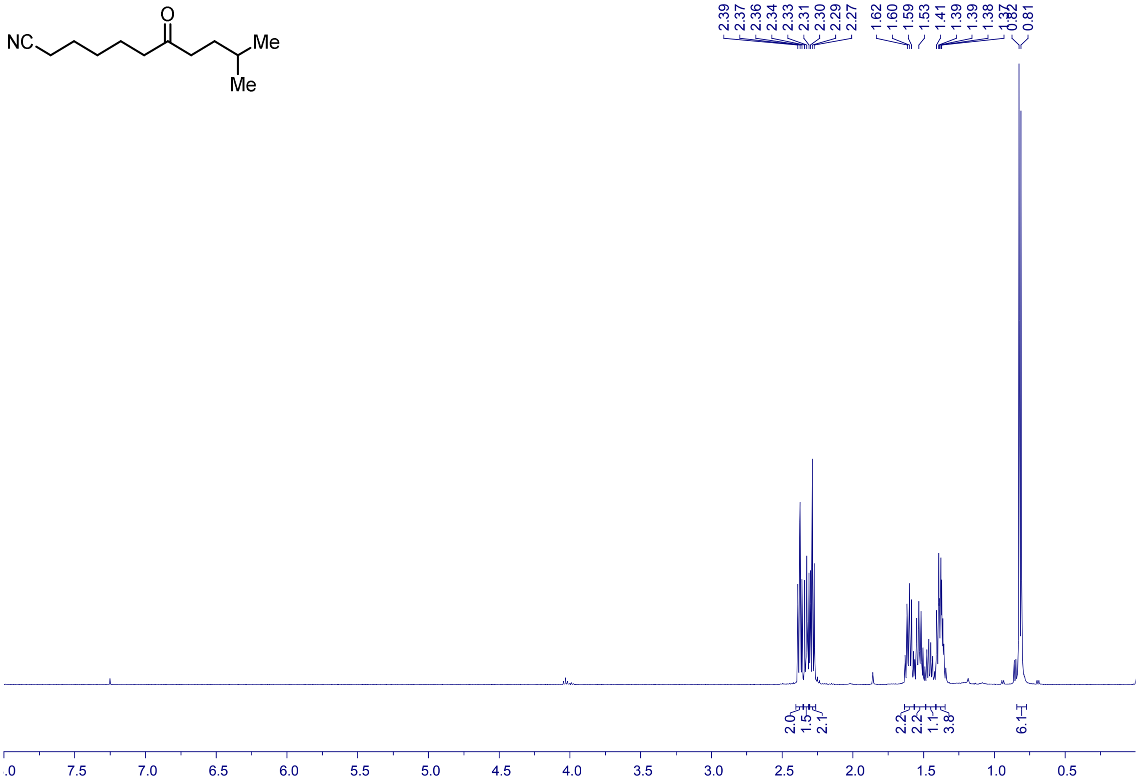

S28 ^{13}C NMR (126 MHz, $CDCl_3$)

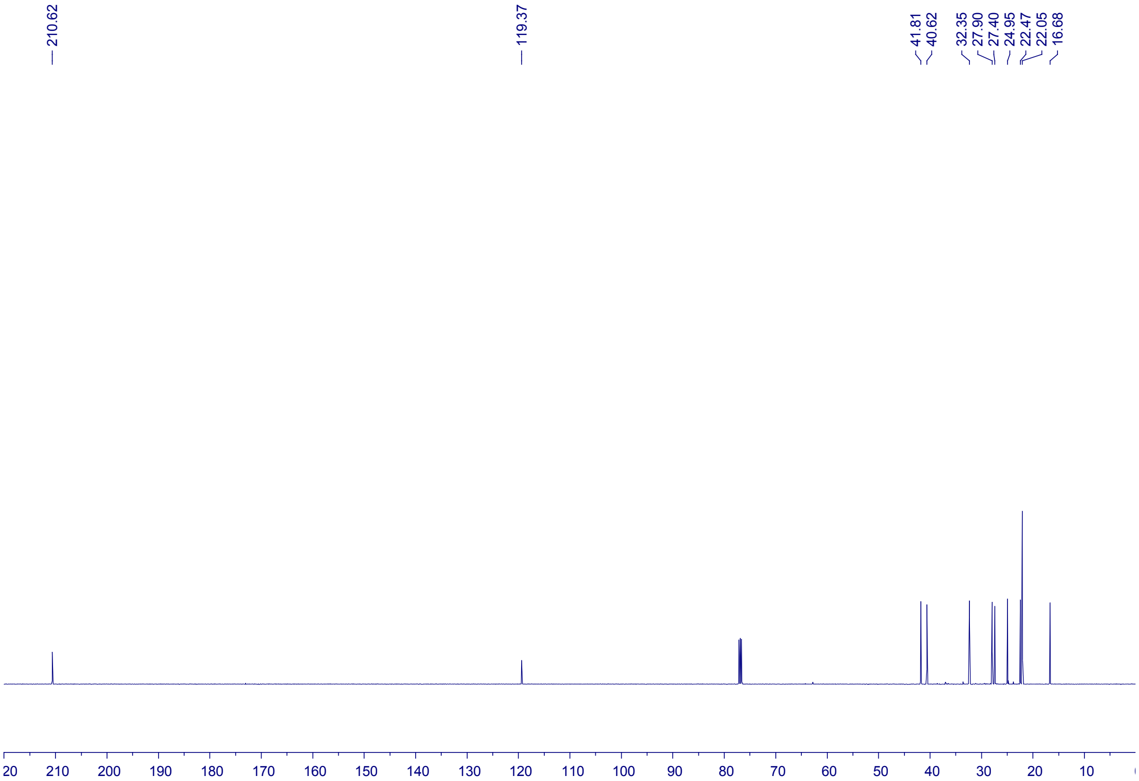

S29 ^{1}H NMR (400 MHz, $CDCl_3$)

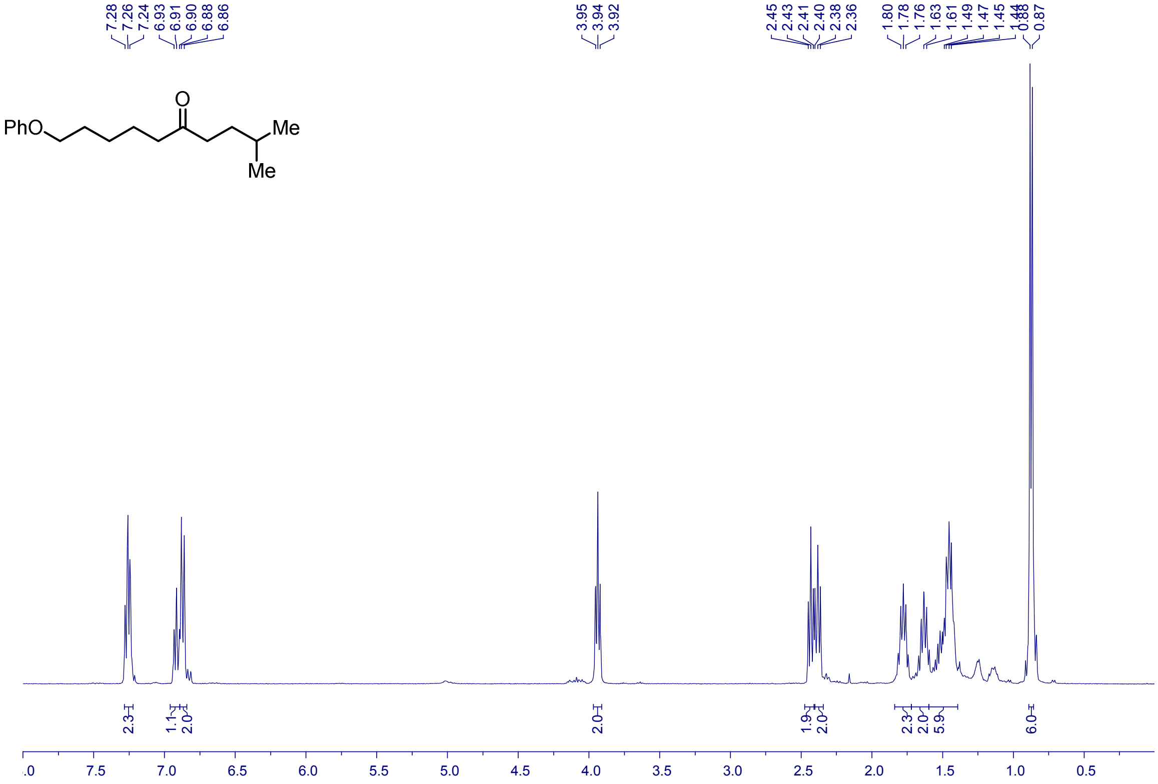

S29 ^{13}C NMR (101 MHz, $CDCl_3$)

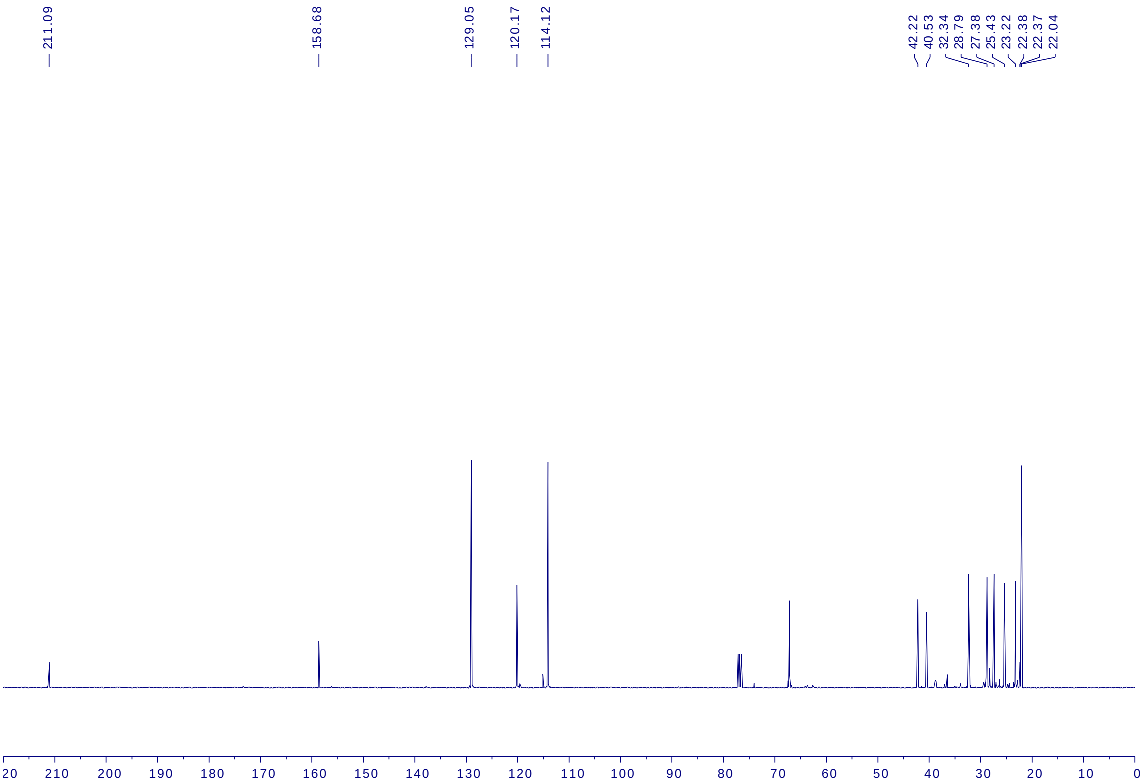

S30 ^{1}H NMR (500 MHz, $CDCl_3$)

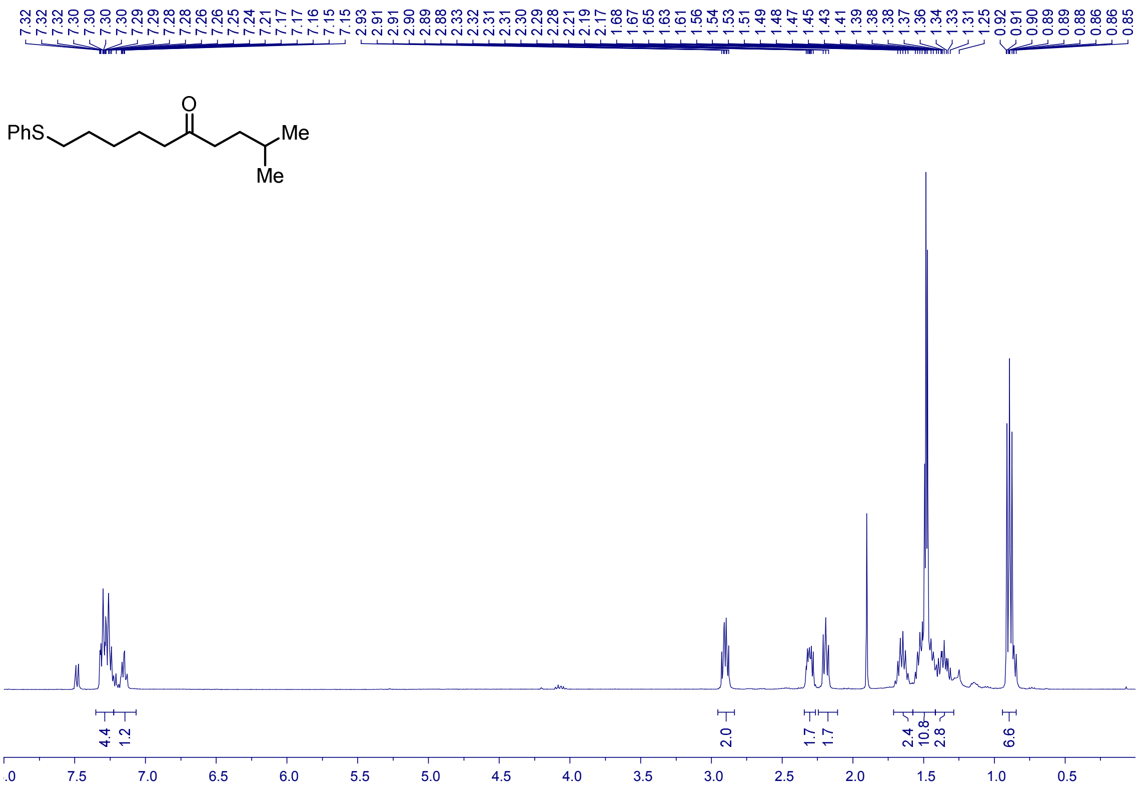

S30 ^{13}C NMR (126 MHz, $CDCl_3$)

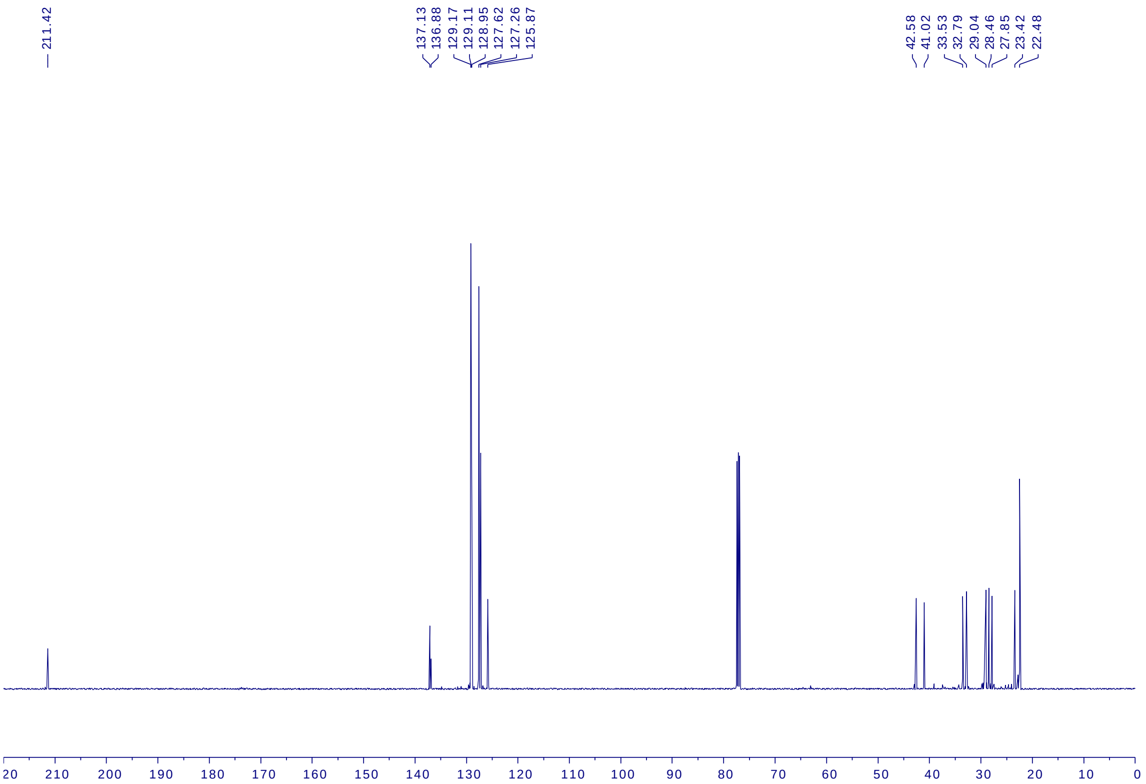

1a ^{1}H NMR (500 MHz, $CDCl_3$)

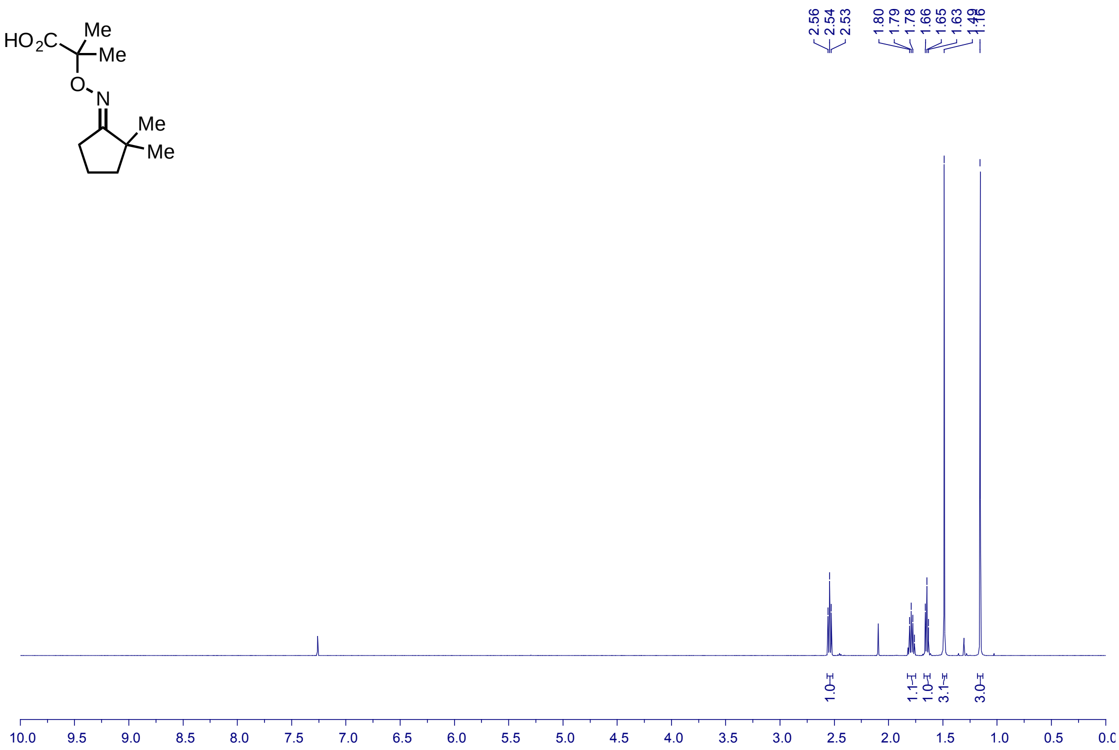

1a ^{13}C NMR (126 MHz, $CDCl_3$)

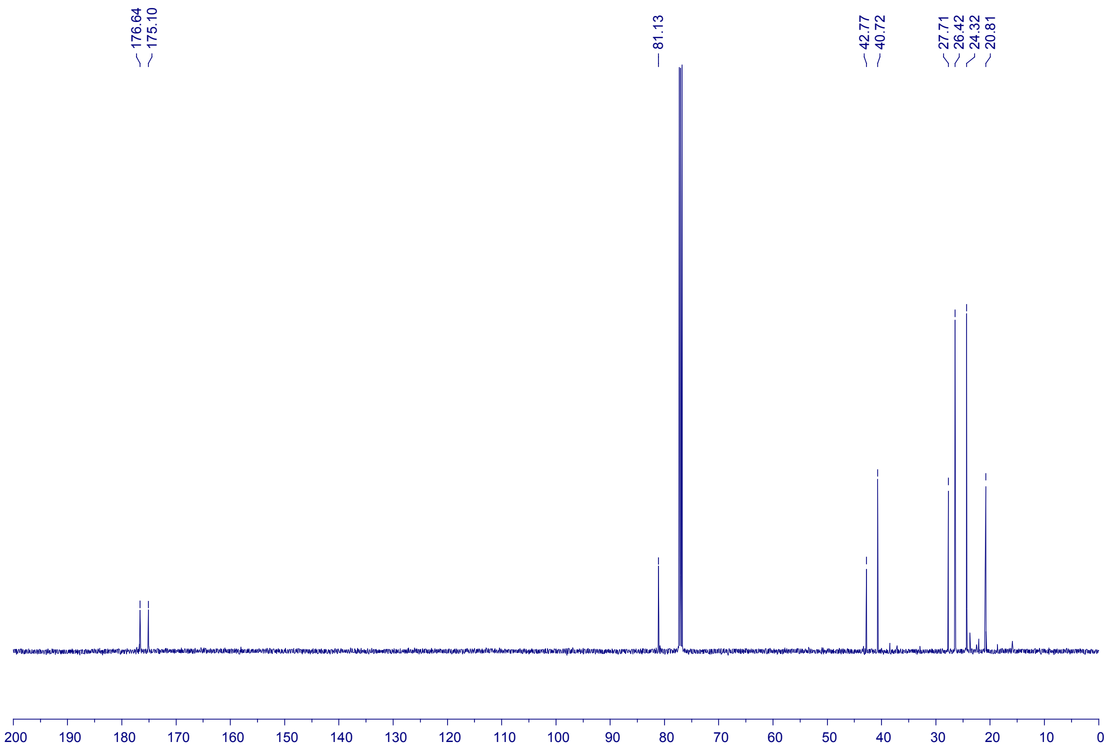

1b ^{1}H NMR (500 MHz, $CDCl_3$)

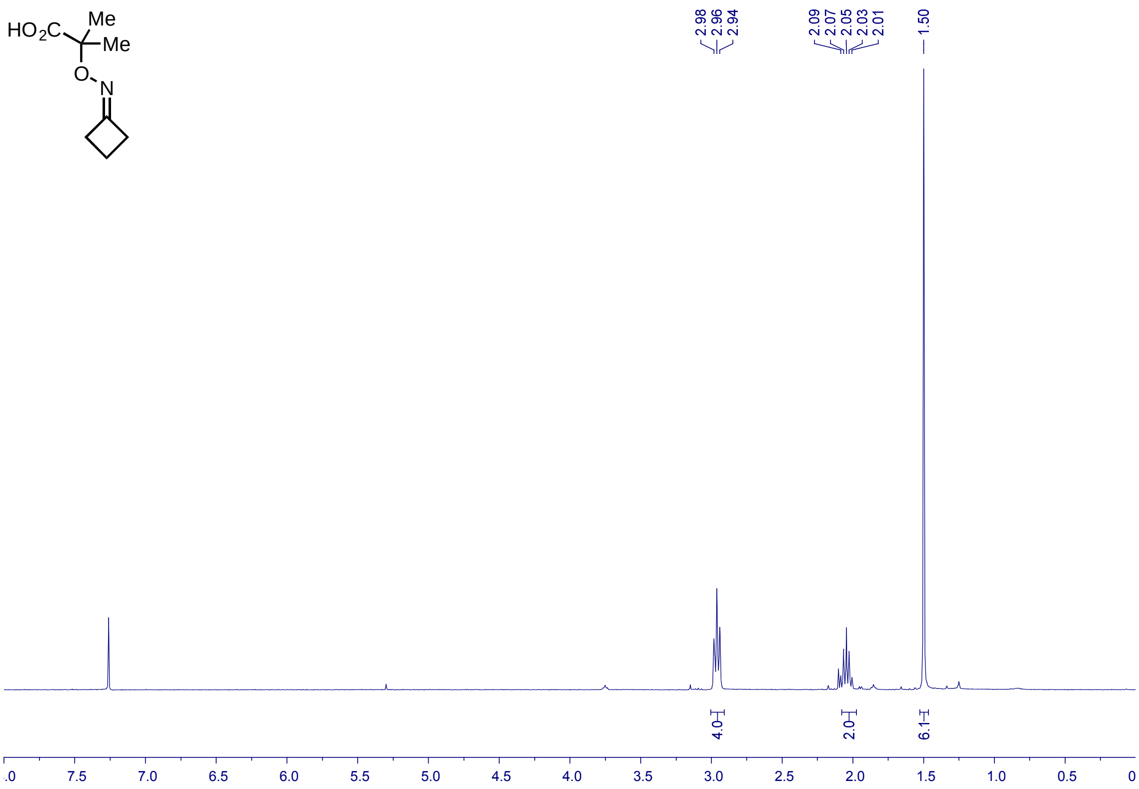

1b ^{13}C NMR (126 MHz, $CDCl_3$)

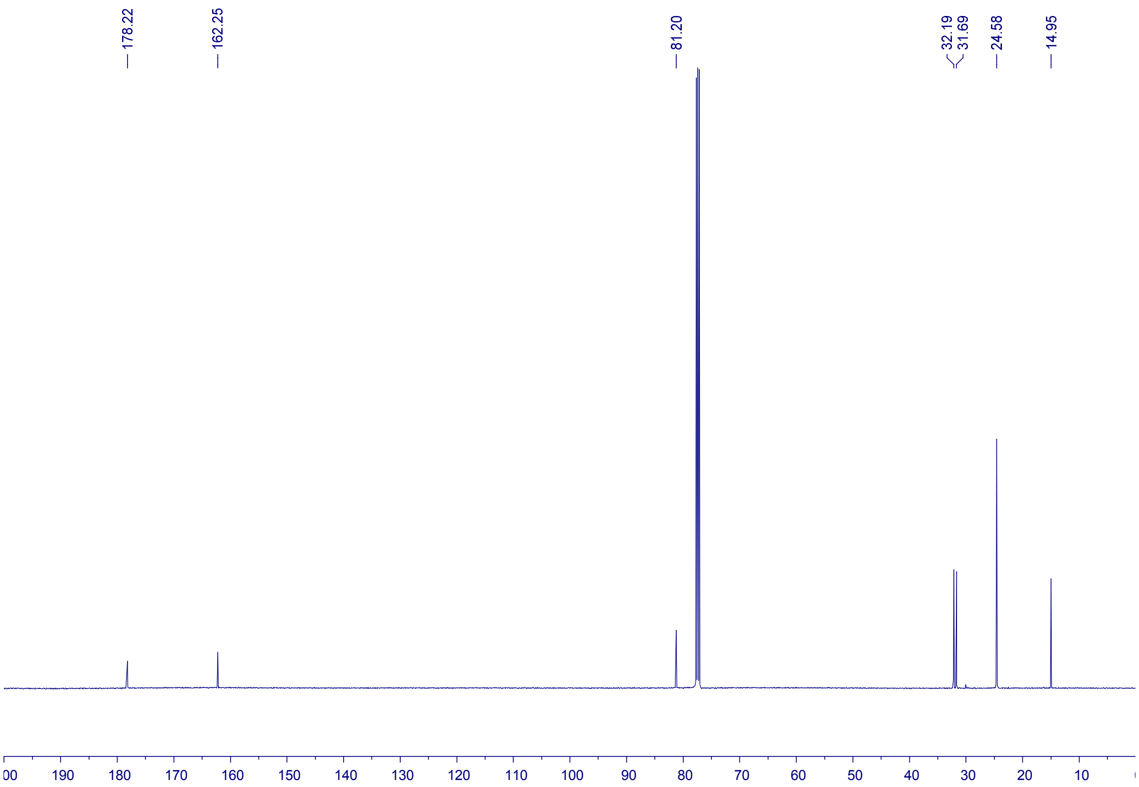

1c ^{1}H NMR (500 MHz, $CDCl_3$)

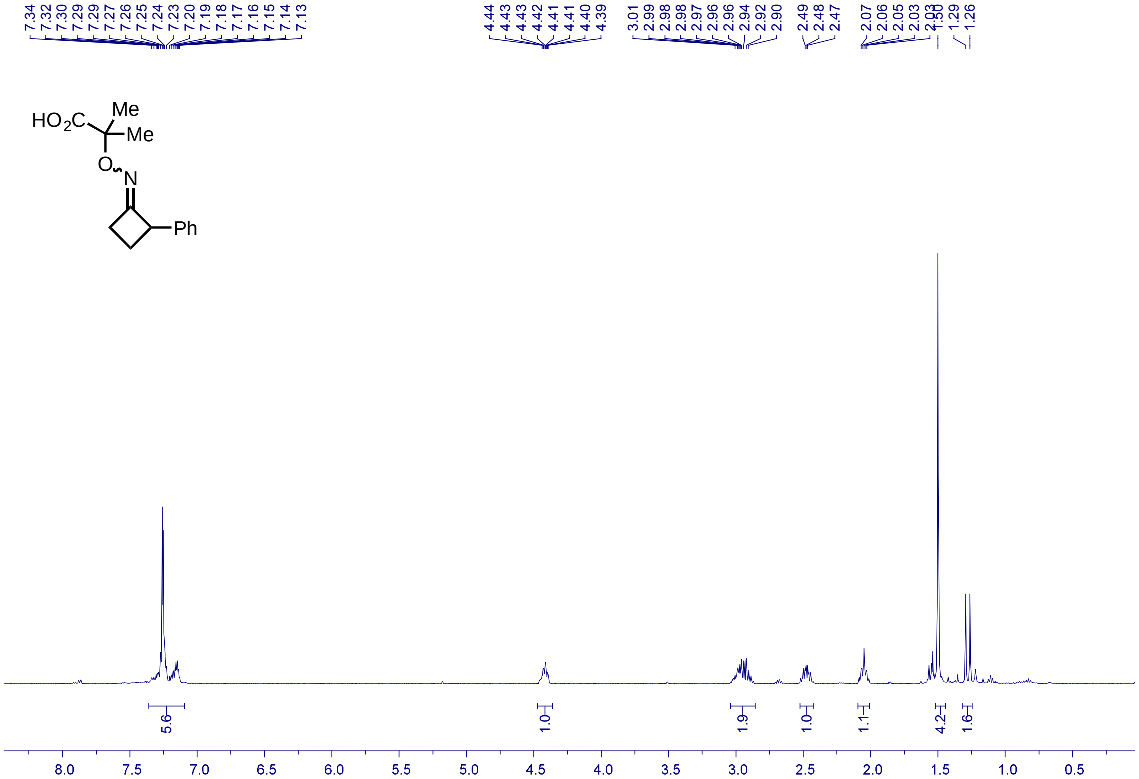

1c ^{13}C NMR (126 MHz, $CDCl_3$)

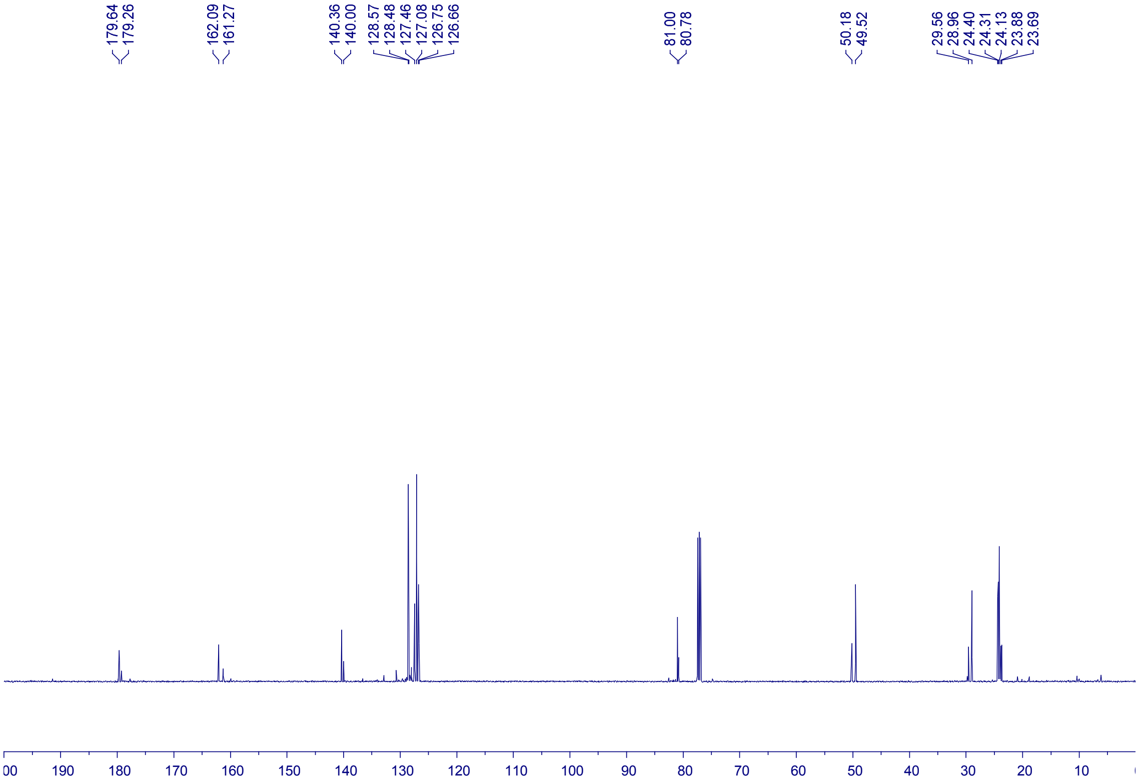

1d ^{1}H NMR (500 MHz, $CDCl_3$)

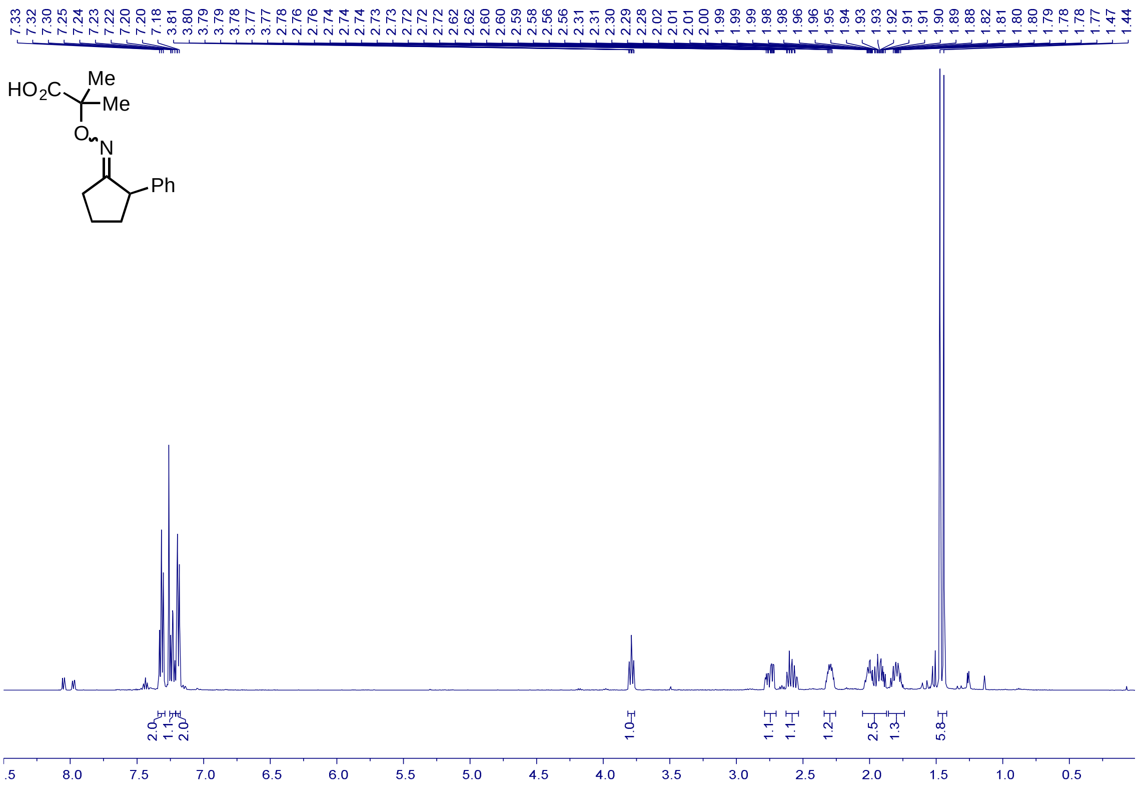

1d ^{13}C NMR (126 MHz, $CDCl_3$)

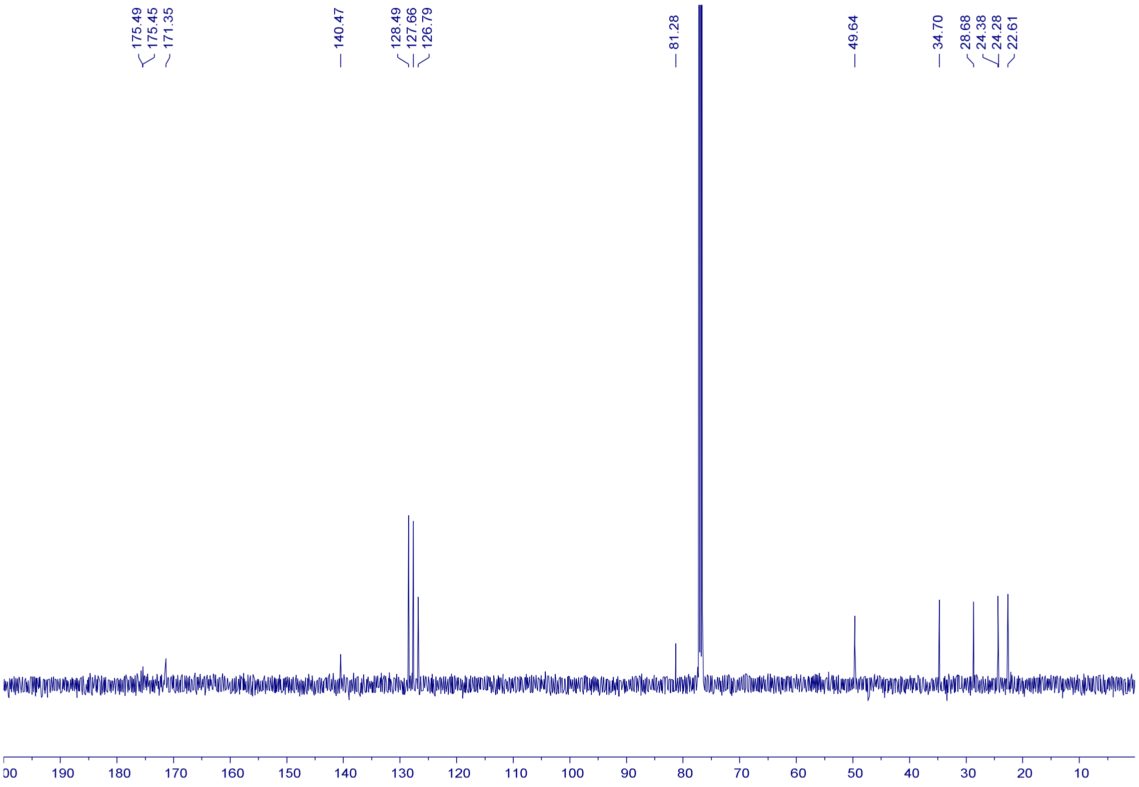

1e ^{1}H NMR (400 MHz, $CDCl_3$)

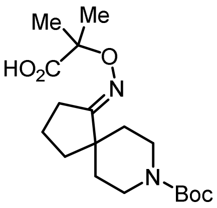

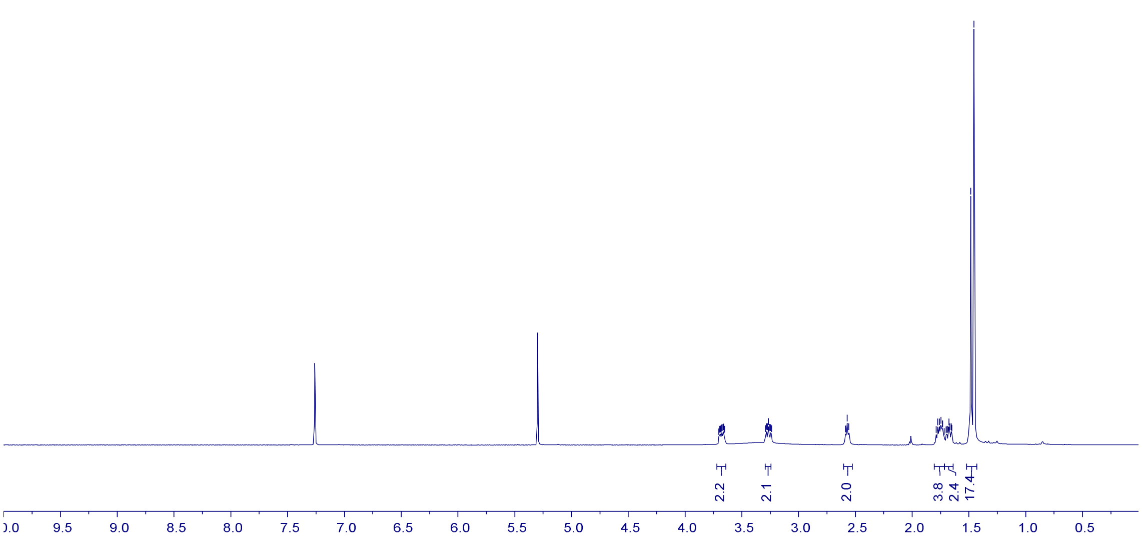

1e ^{13}C NMR (101 MHz, $CDCl_3$)

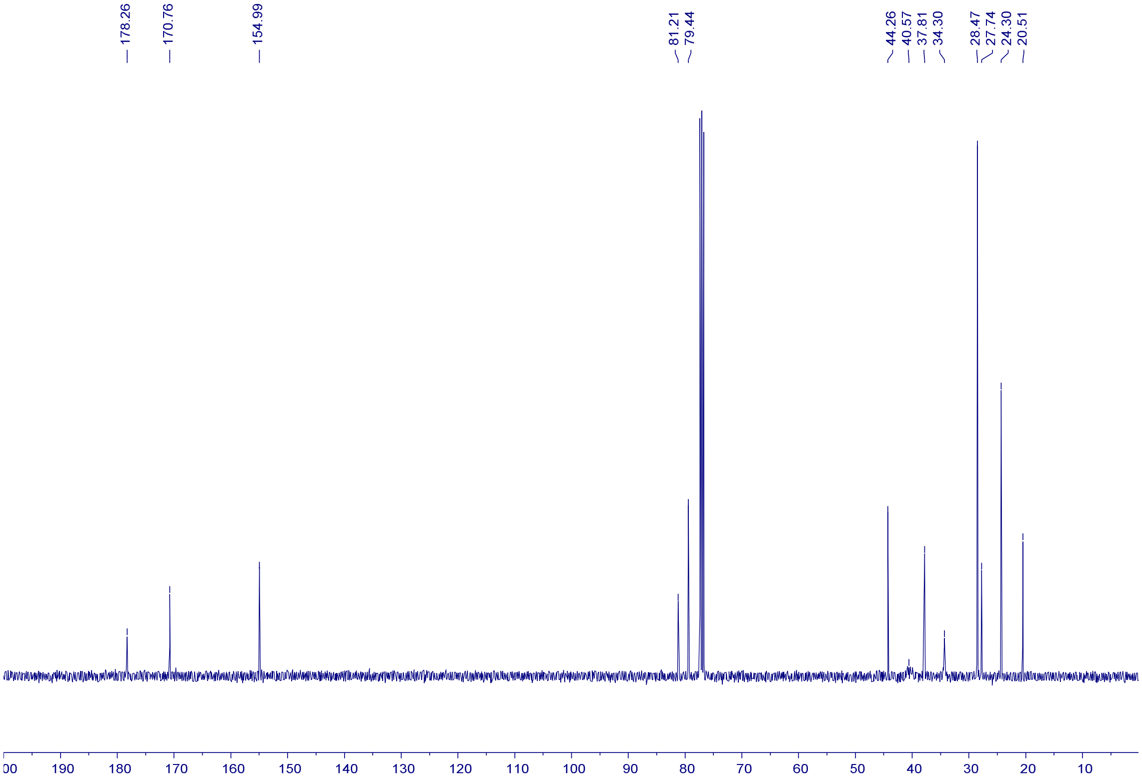

1f ^{1}H NMR (500 MHz, $CDCl_3$)

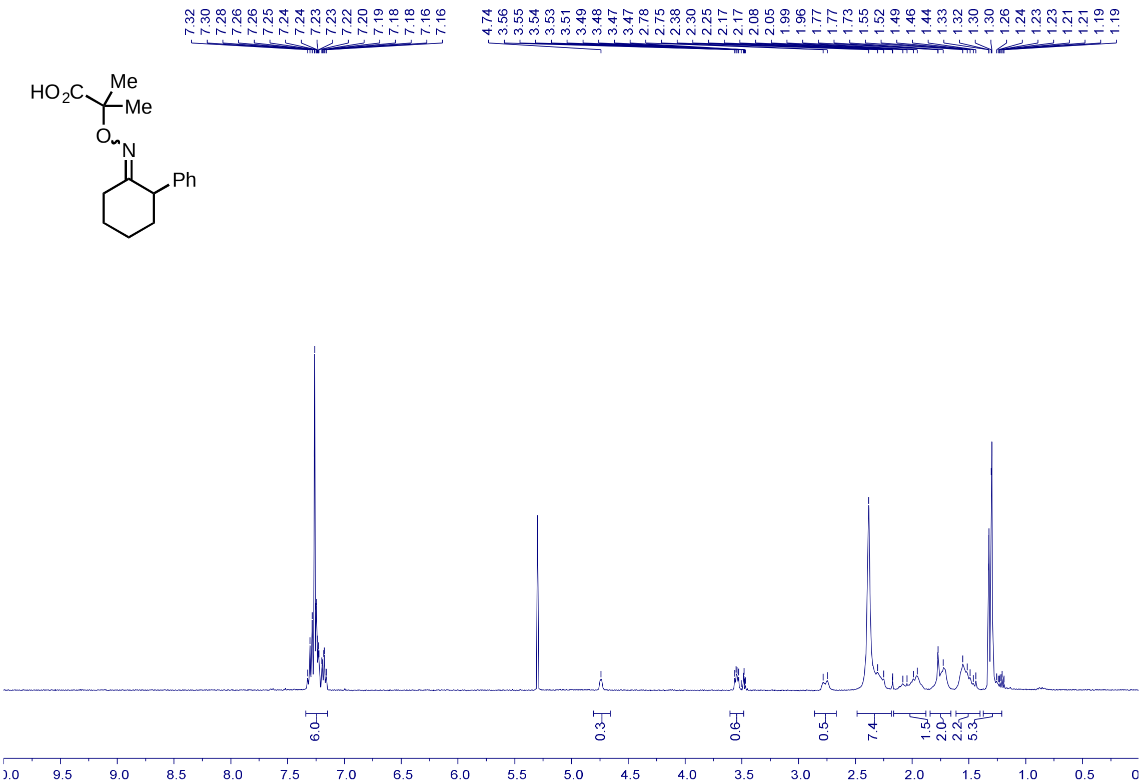

1f ^{13}C NMR (101 MHz, $CDCl_3$)

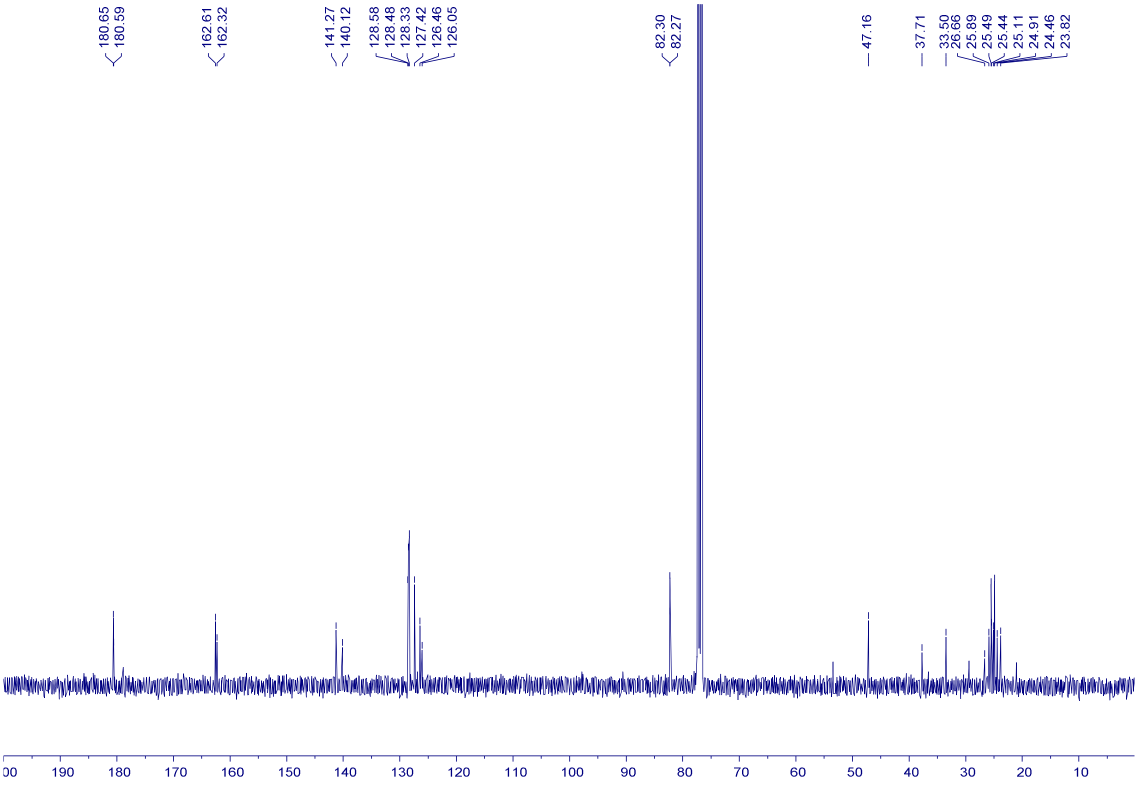

1g ^{1}H NMR (400 MHz, $CDCl_3$)

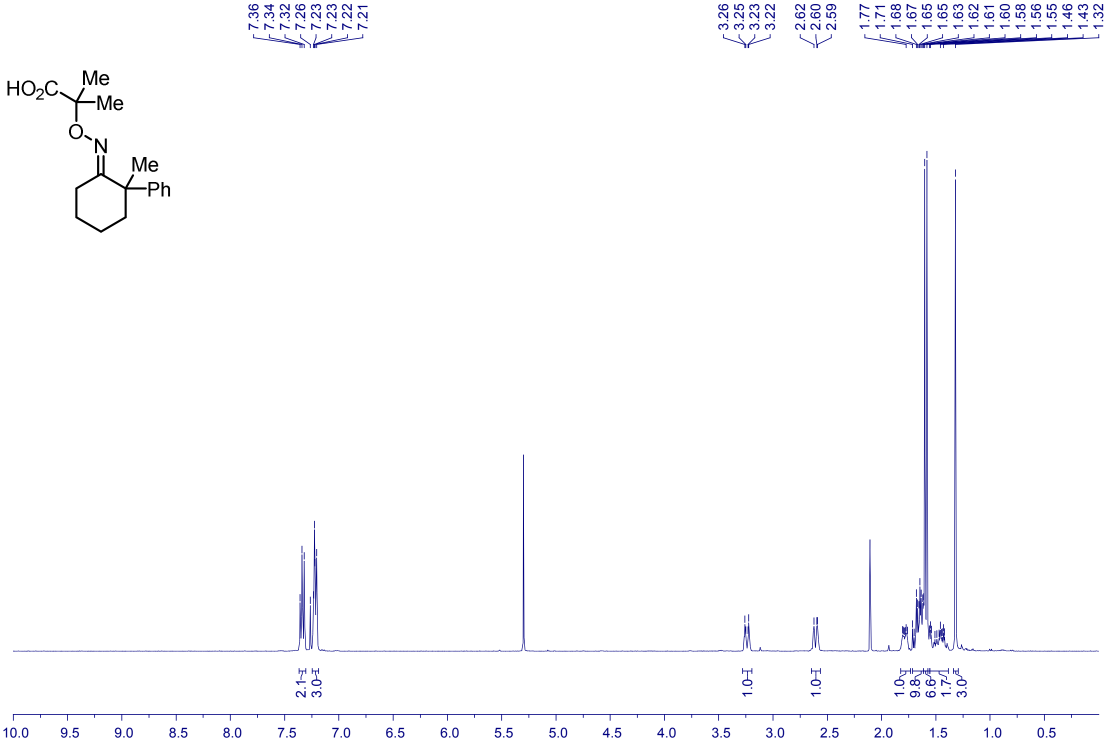

1g ^{13}C NMR (101 MHz, $CDCl_3$)

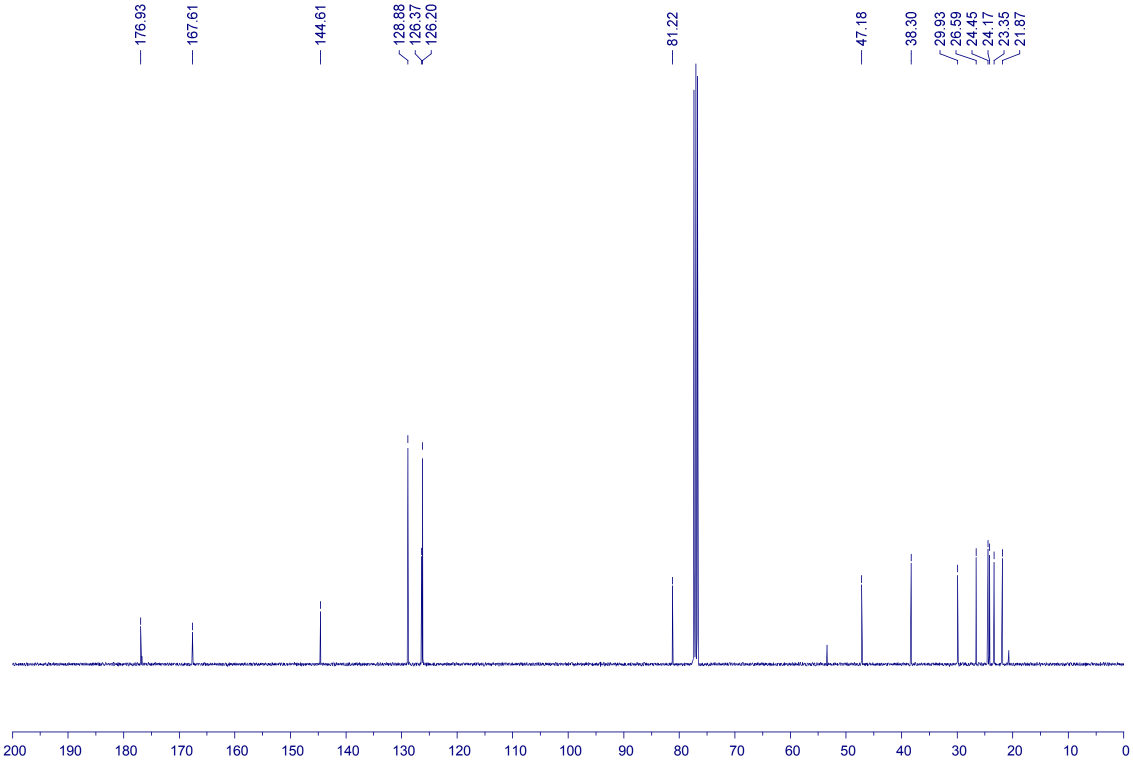

1h ^{1}H NMR (500 MHz, $CDCl_3$)

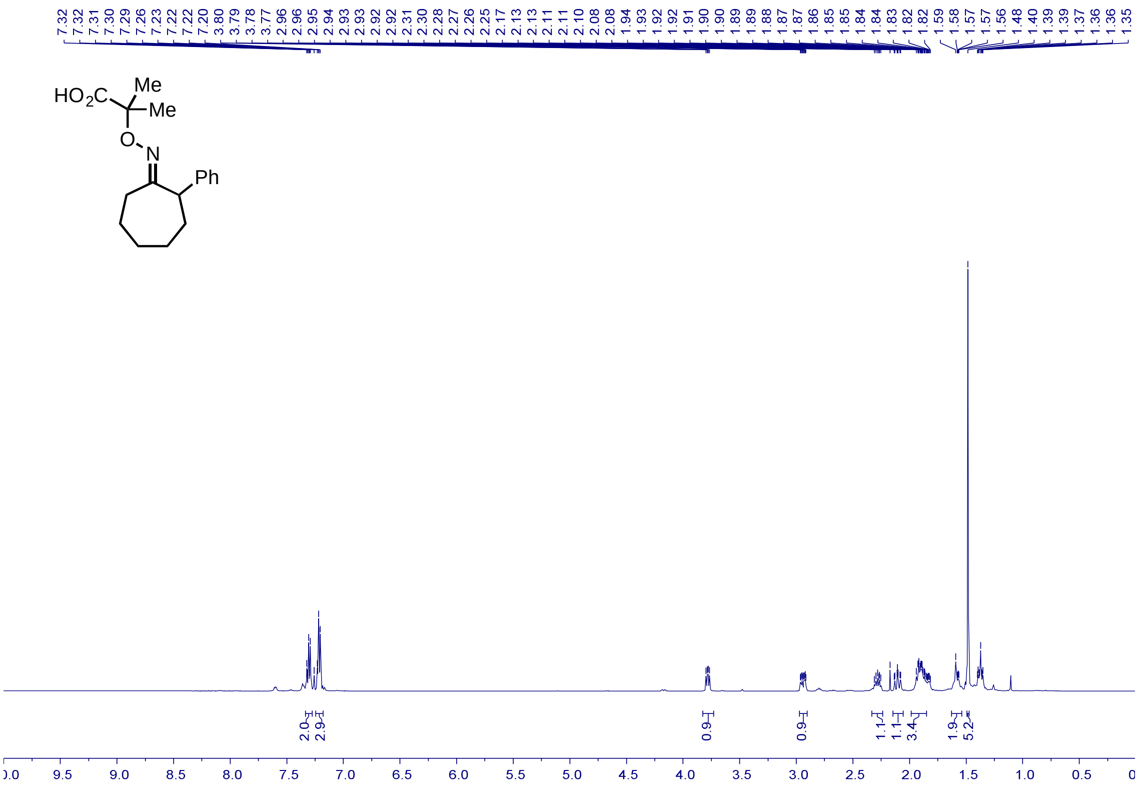

1h ^{13}C NMR (126 MHz, $CDCl_3$)

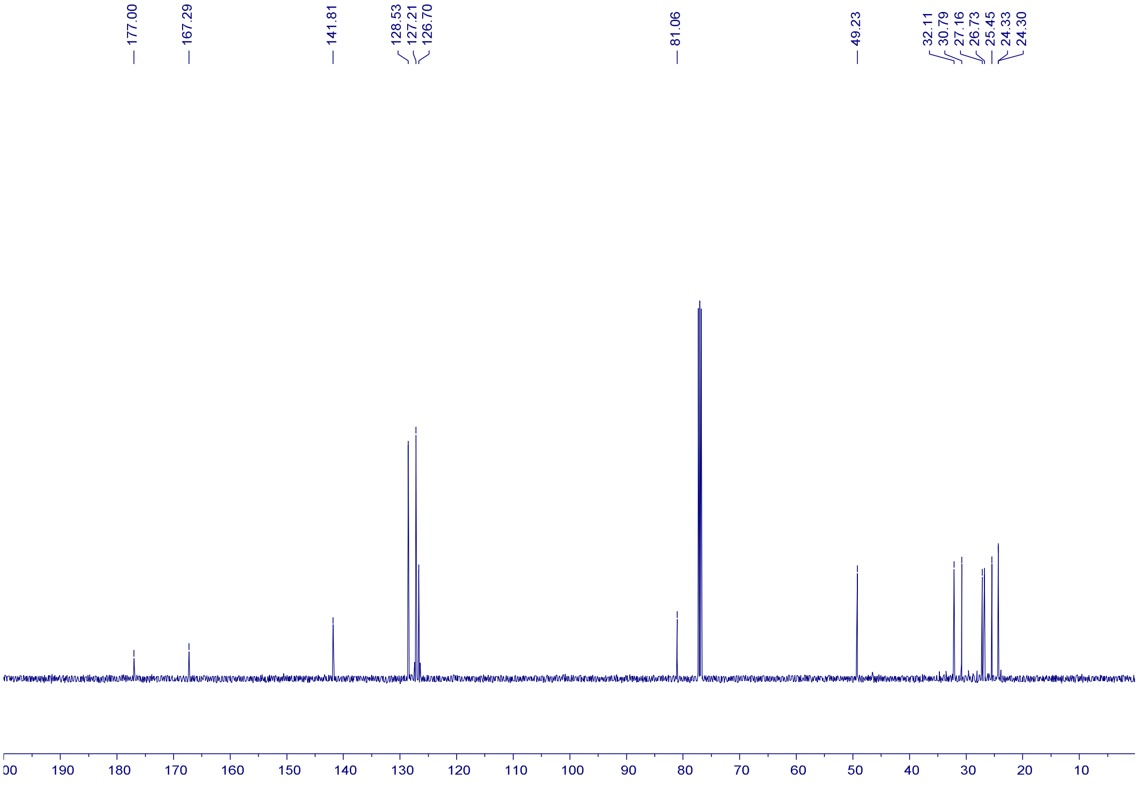

1i ^{1}H NMR ($CDCl_3$, 400 MHz)

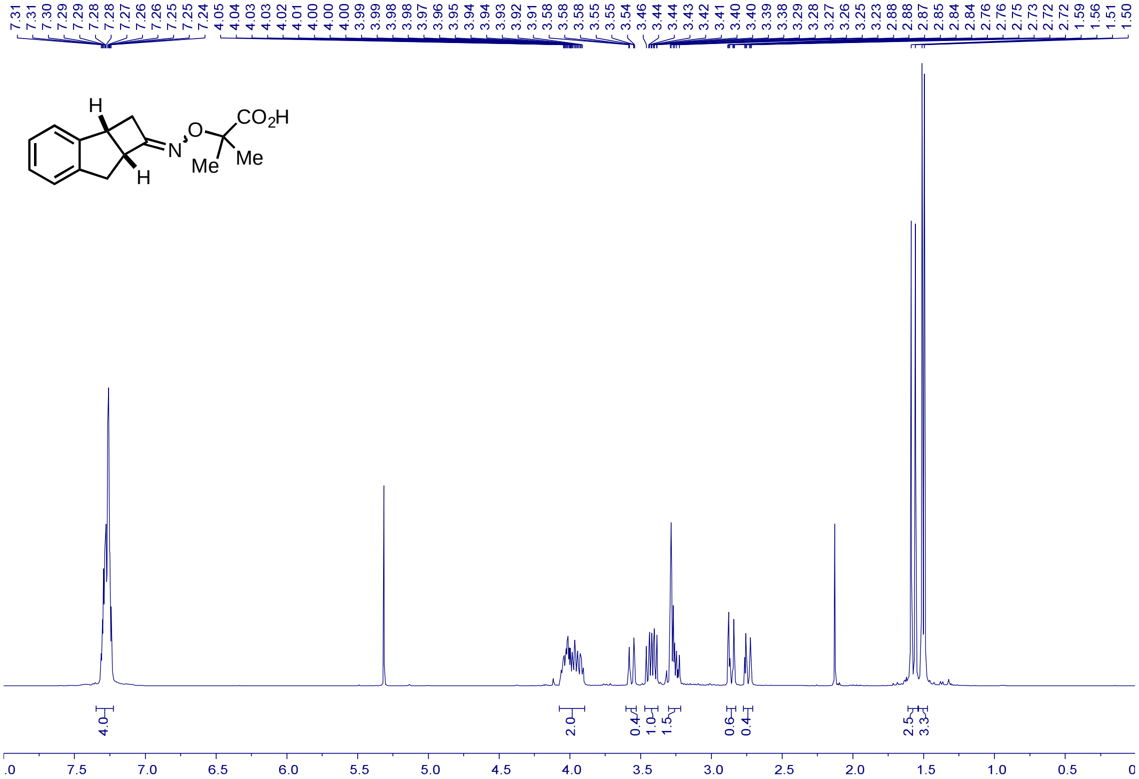

1i ^{13}C NMR ($CDCl_3$, 126 MHz)

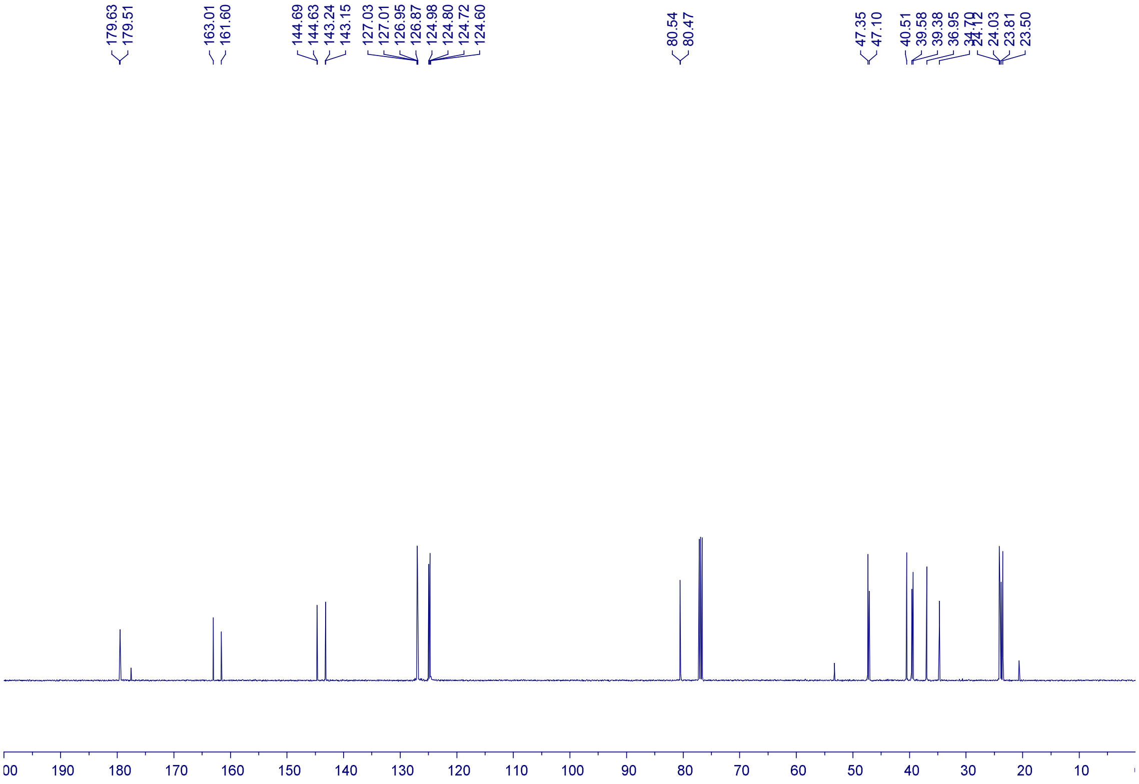

1k ^{1}H NMR (500 MHz, $CDCl_3$)

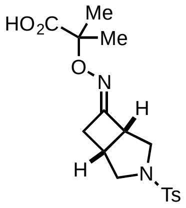

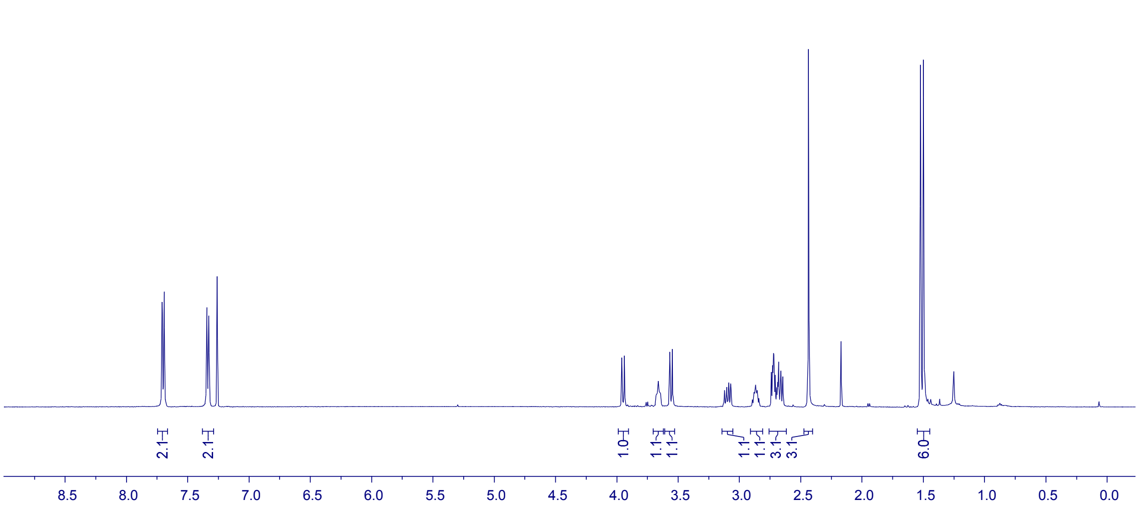

1k ^{13}C NMR (126 MHz, $CDCl_3$)

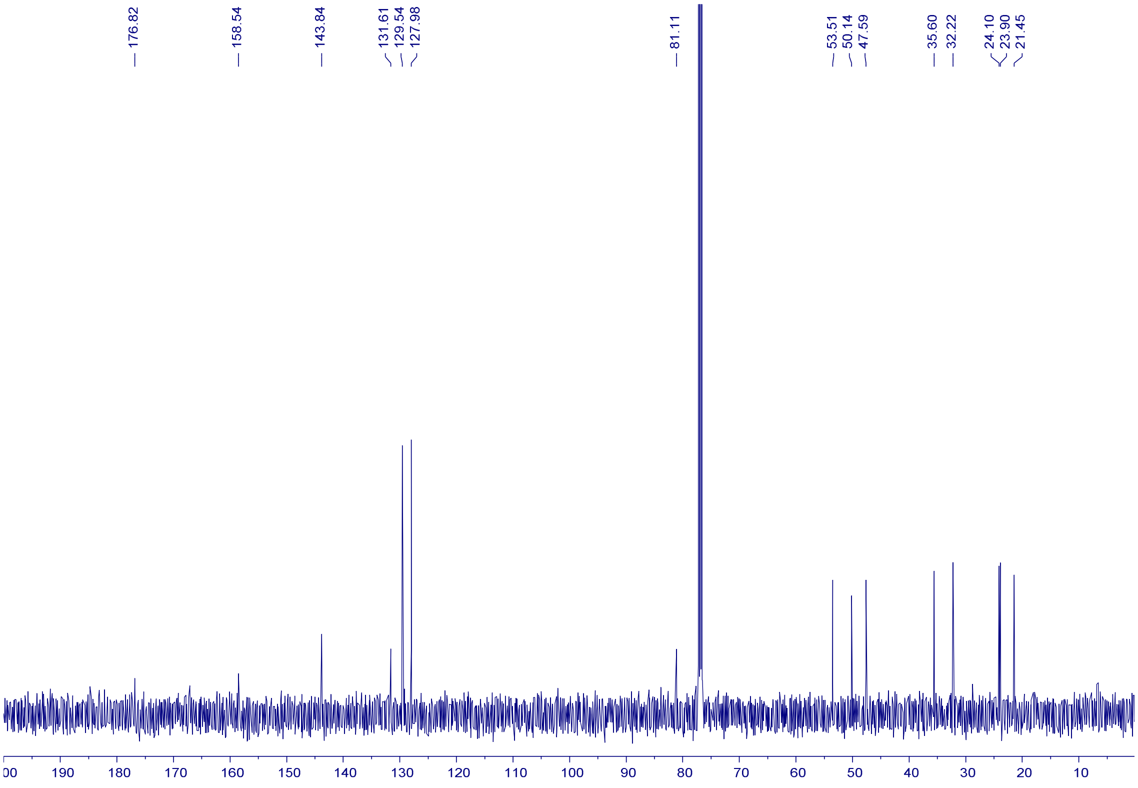

1k ^{1}H NMR ($CDCl_3$, 500 MHz)

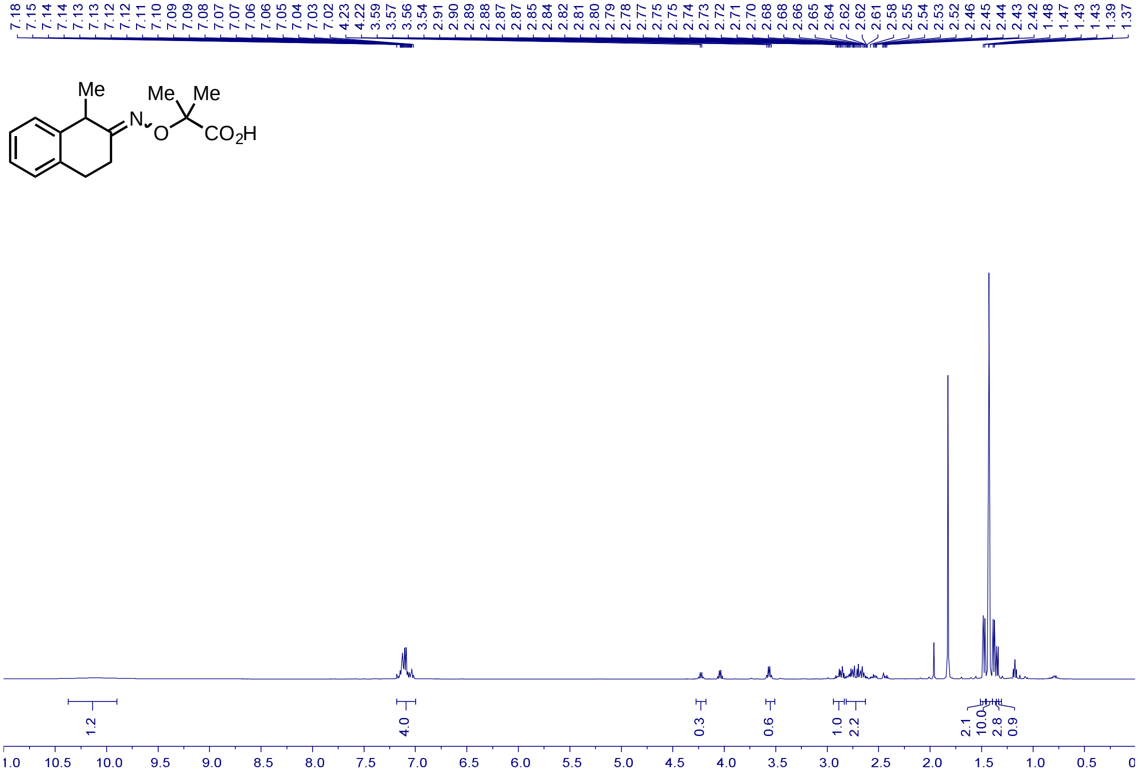

1k ^{13}C NMR ($CDCl_3$, 126 MHz)

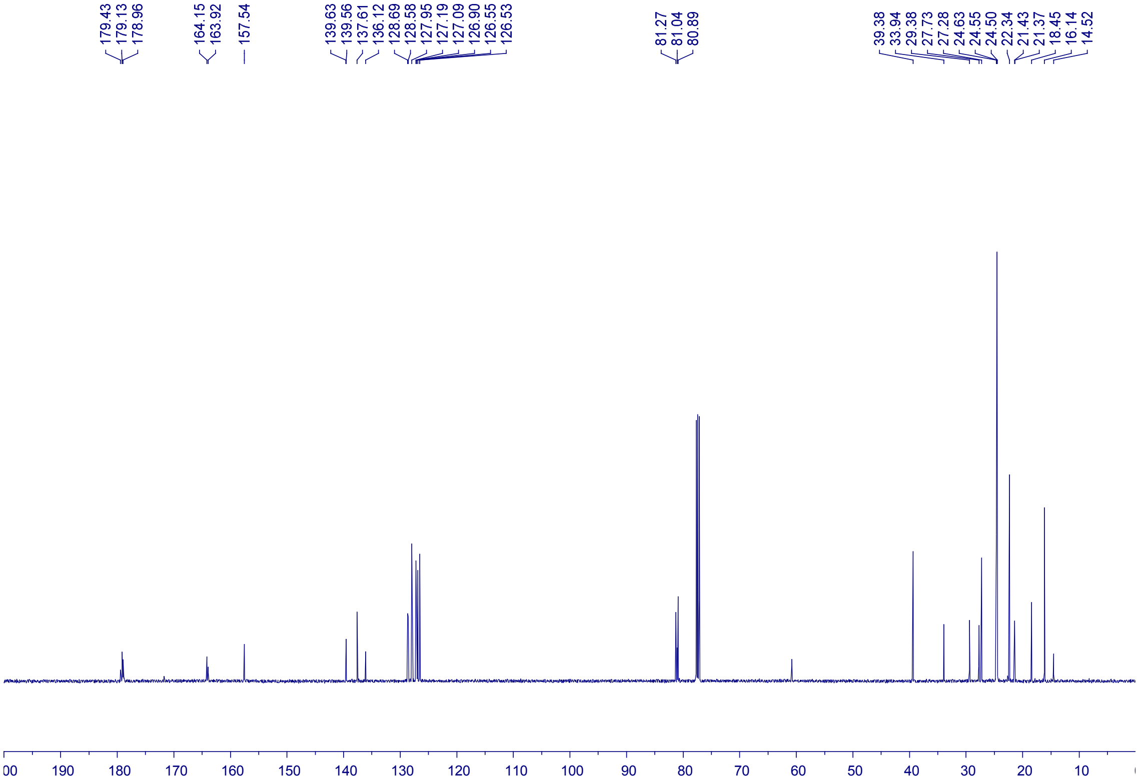

1l ^{1}H NMR (400 MHz, $CDCl_3$)

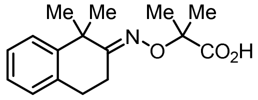

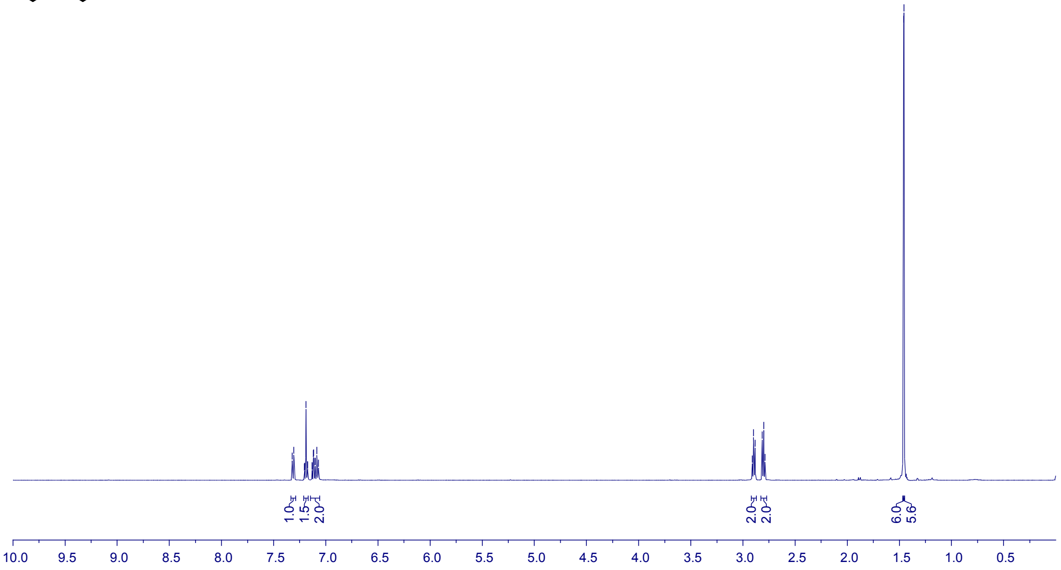

1l ^{13}C NMR (126 MHz, $CDCl_3$)

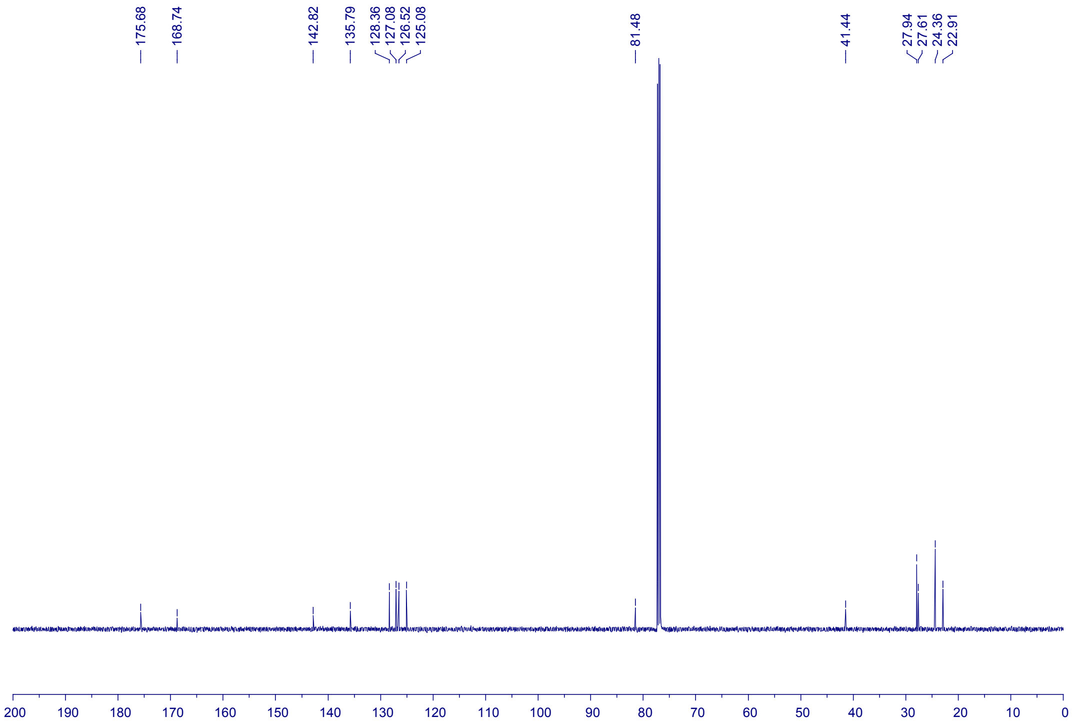

1m ^{1}H NMR (400 MHz, $CDCl_3$)

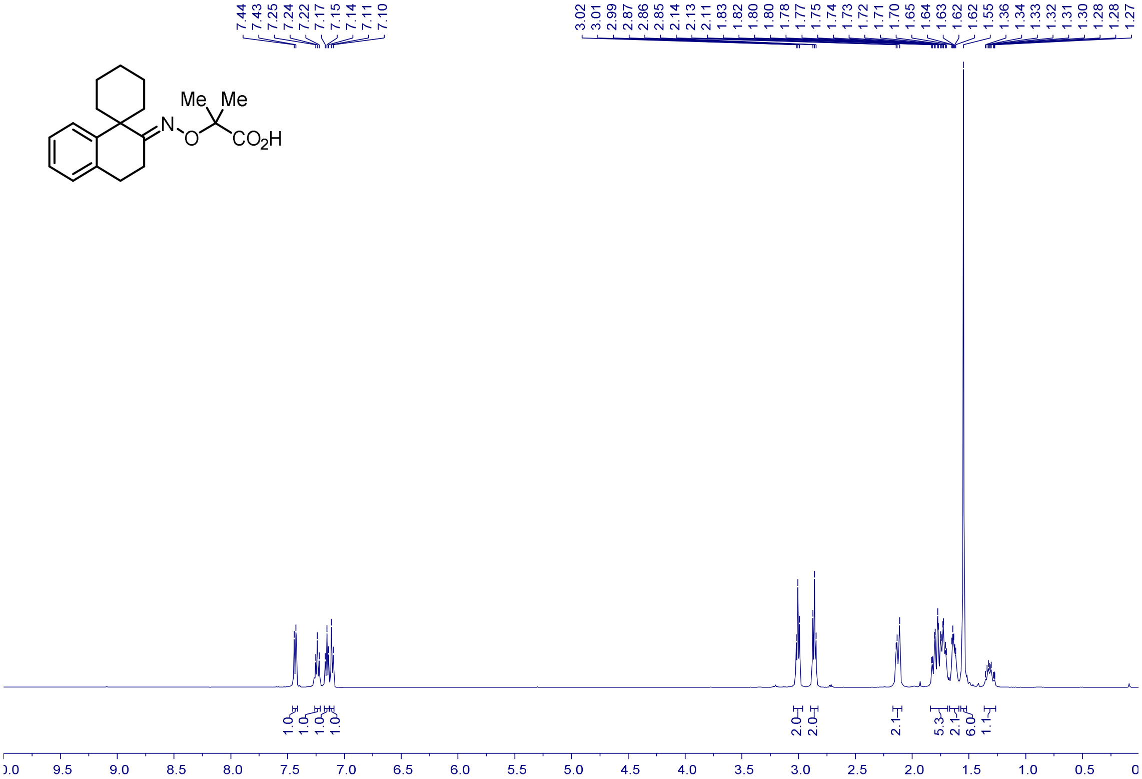

1m ^{13}C NMR (101 MHz, $CDCl_3$)

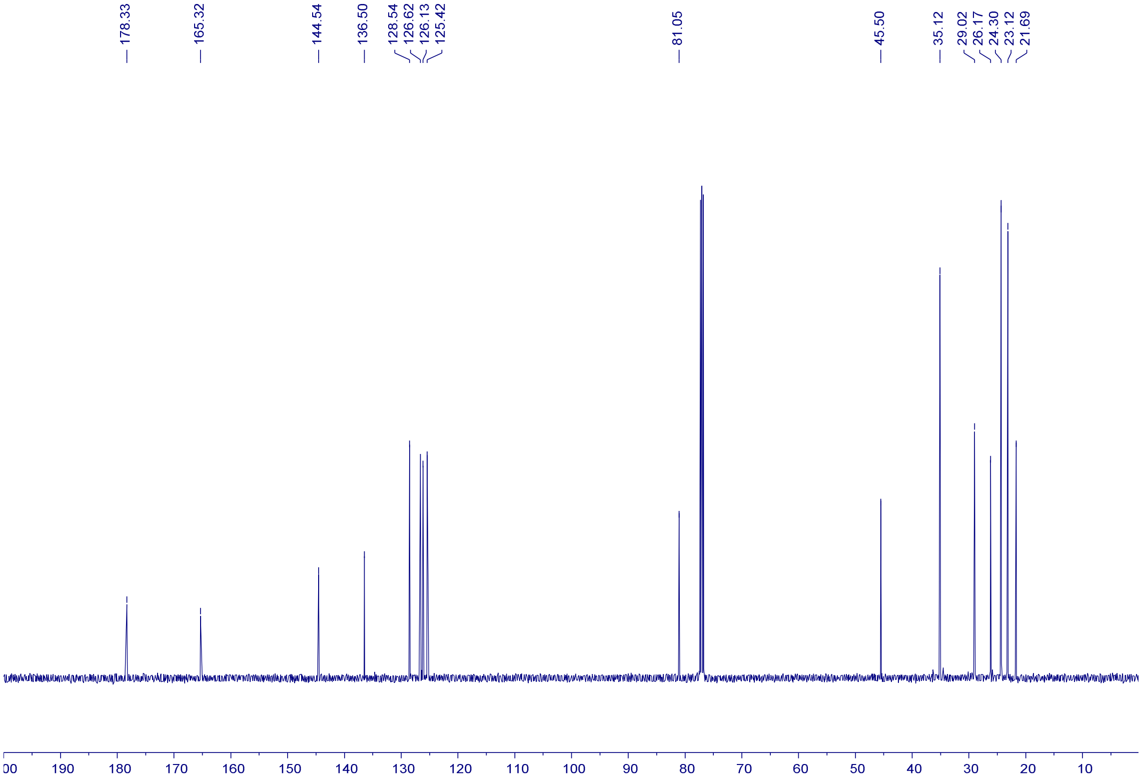

1n ^{1}H NMR (400 MHz, $CDCl_3$)

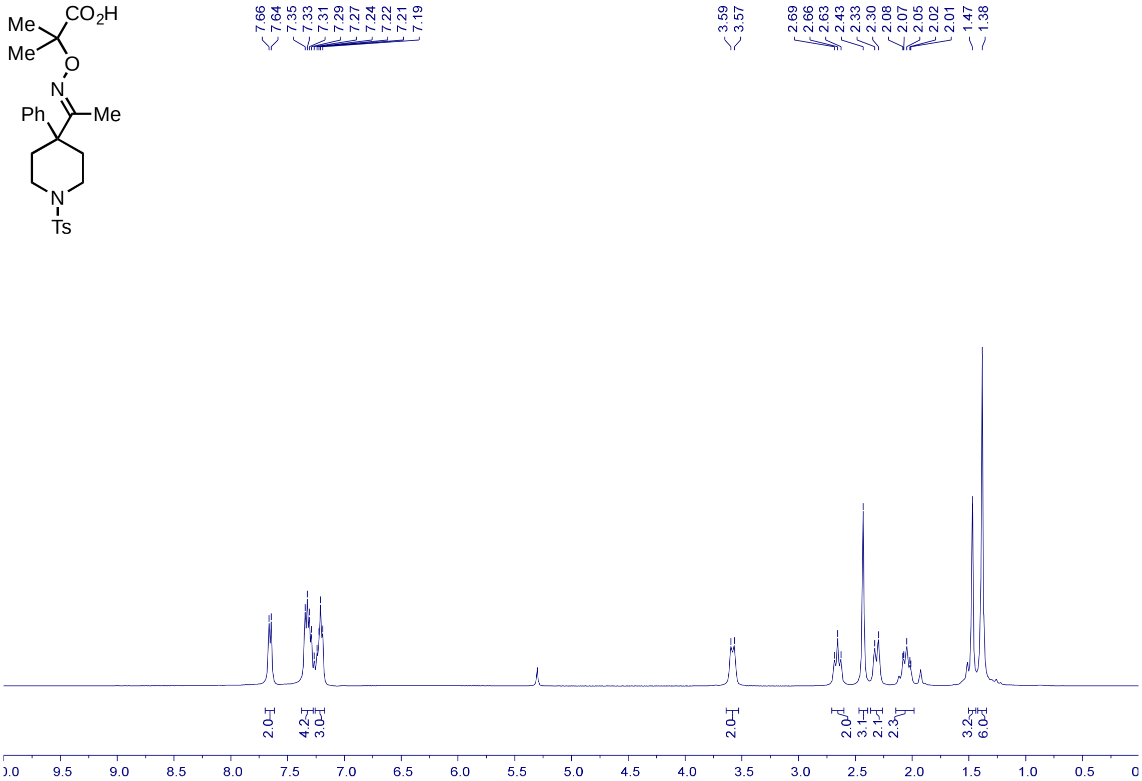

1n ^{13}C NMR (101 MHz, $CDCl_3$)

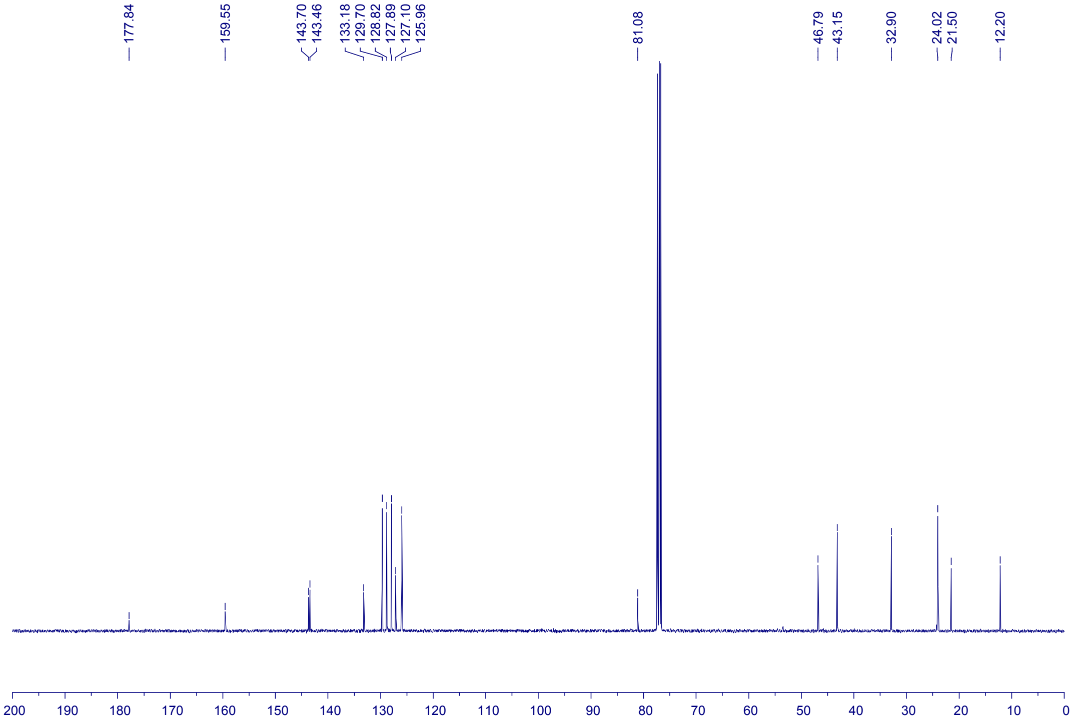

1o ^{1}H NMR (400 MHz, $CDCl_3$)

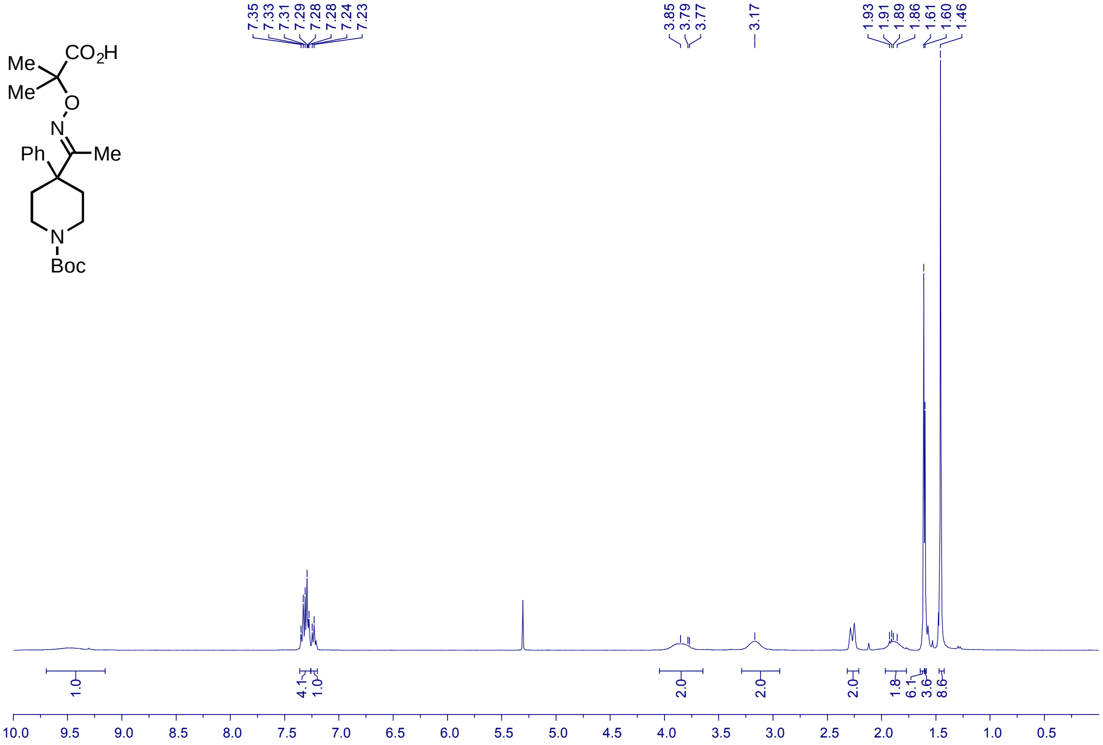

1o ^{13}C NMR (101 MHz, $CDCl_3$)

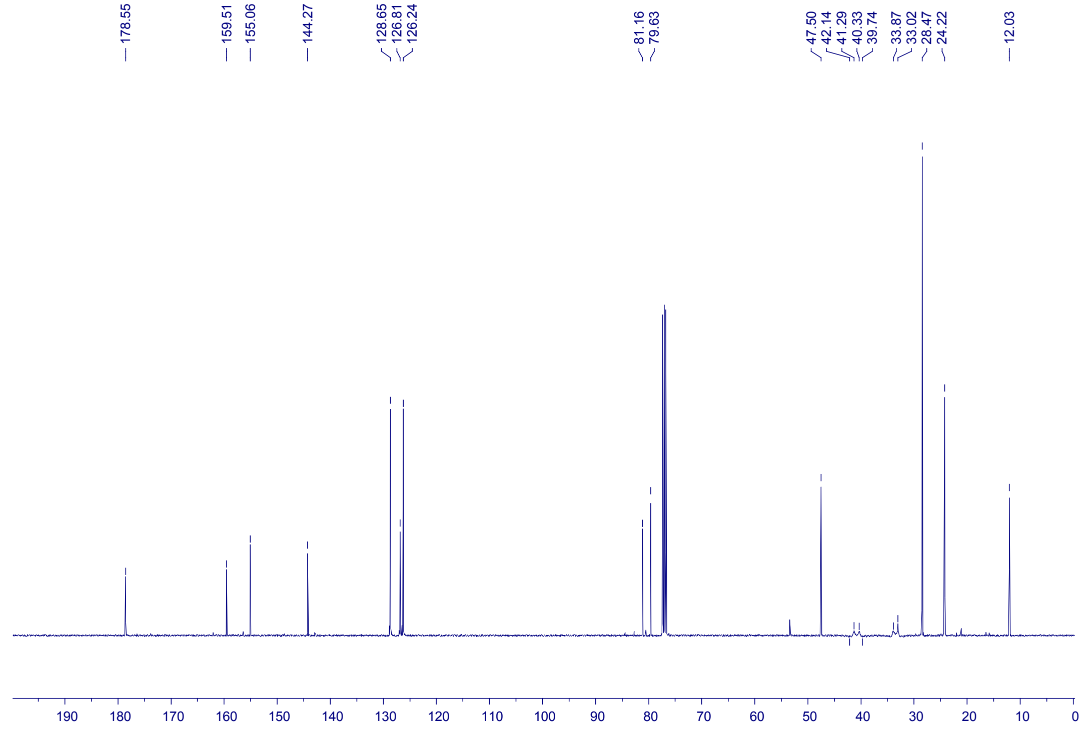

1p ^{1}H NMR (400 MHz, $CDCl_3$)

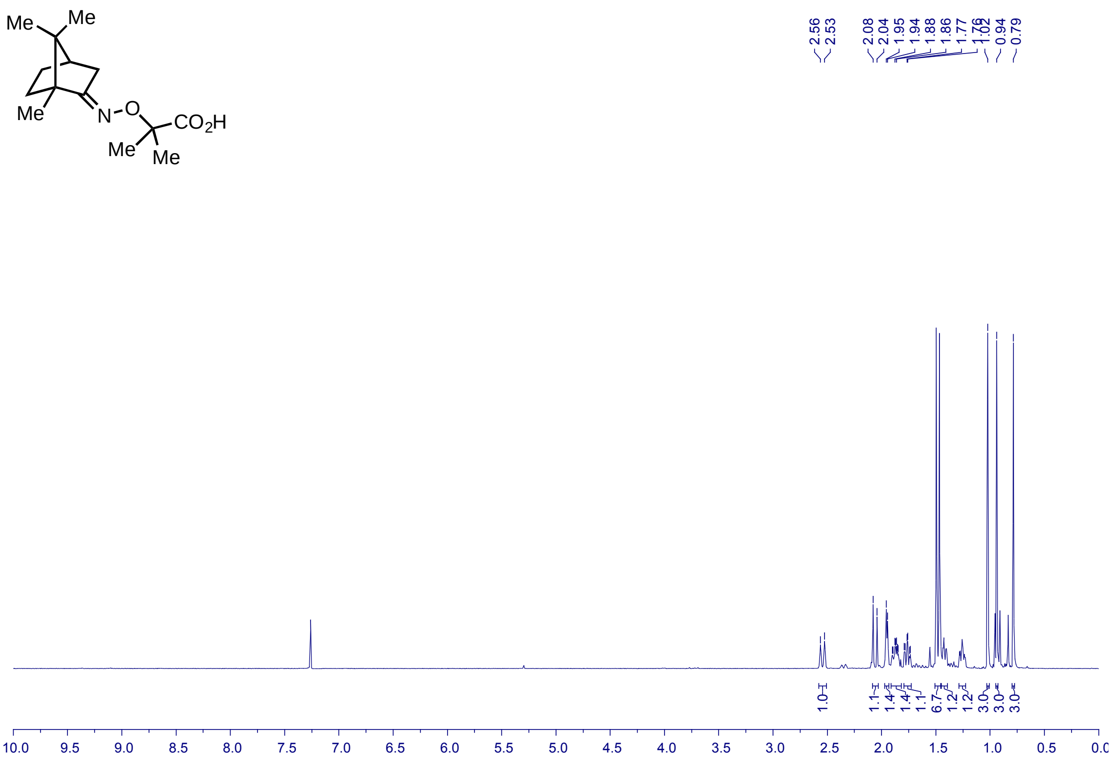

1p ^{13}C NMR (101 MHz, $CDCl_3$)

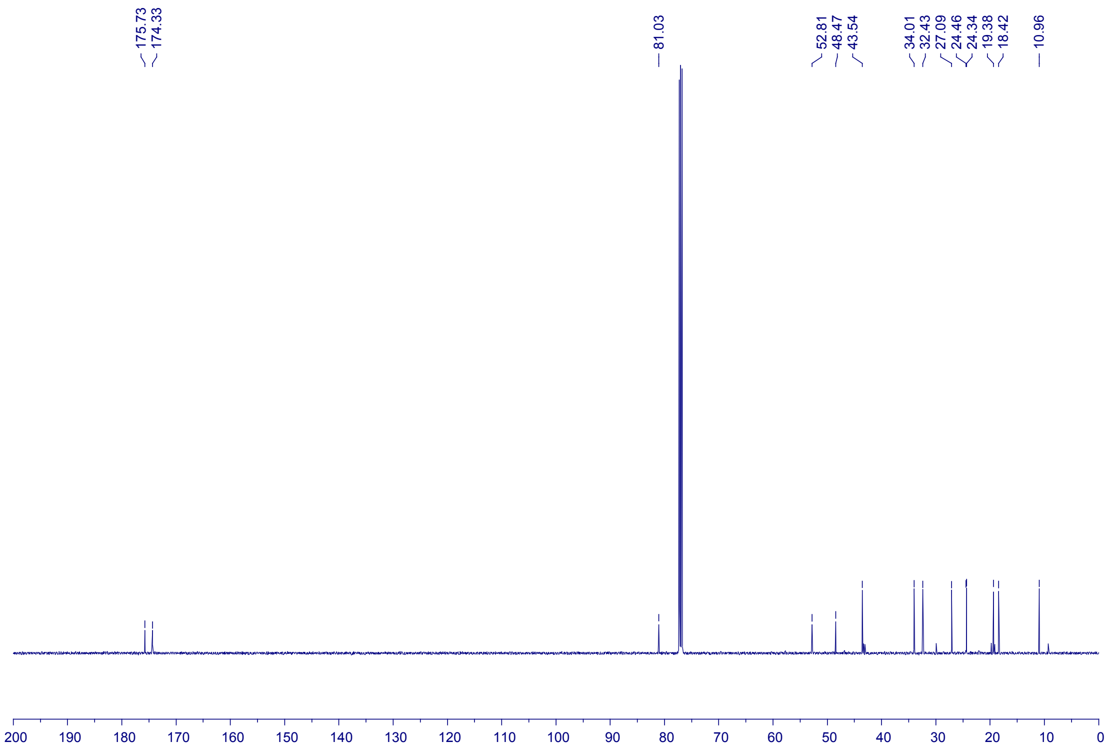

1q ^{1}H NMR (400 MHz, $CDCl_3$)

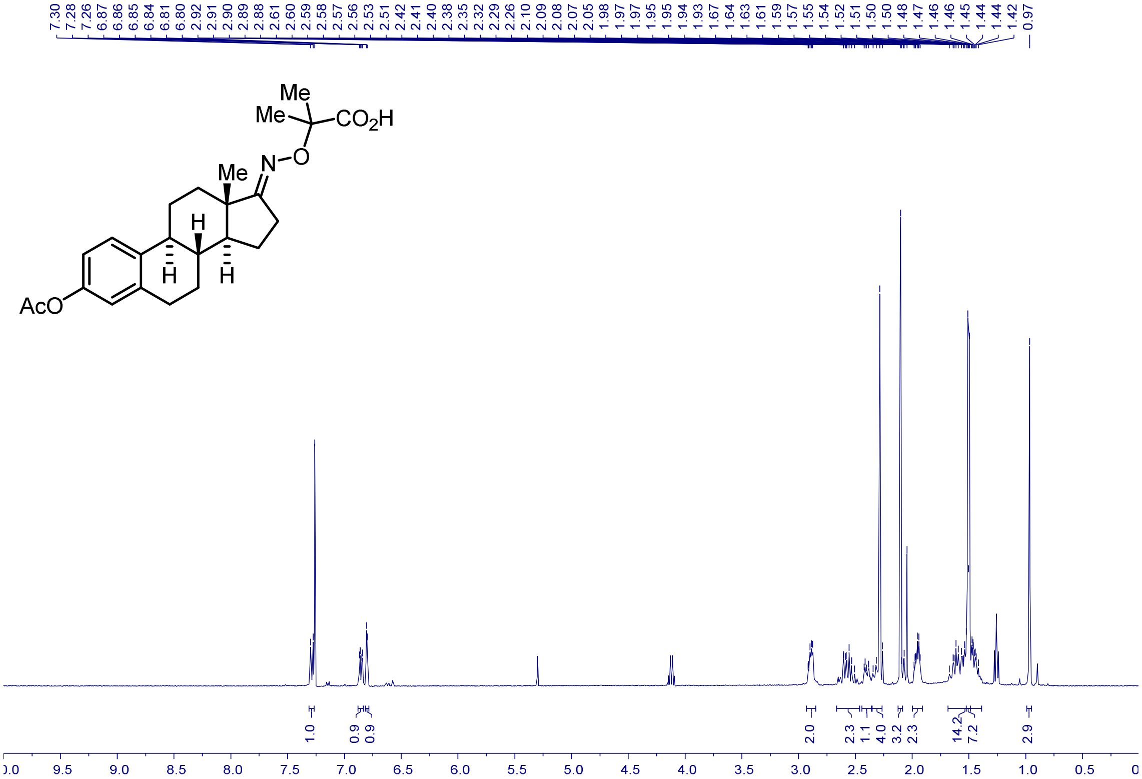

1q ^{13}C NMR (101 MHz, $CDCl_3$)

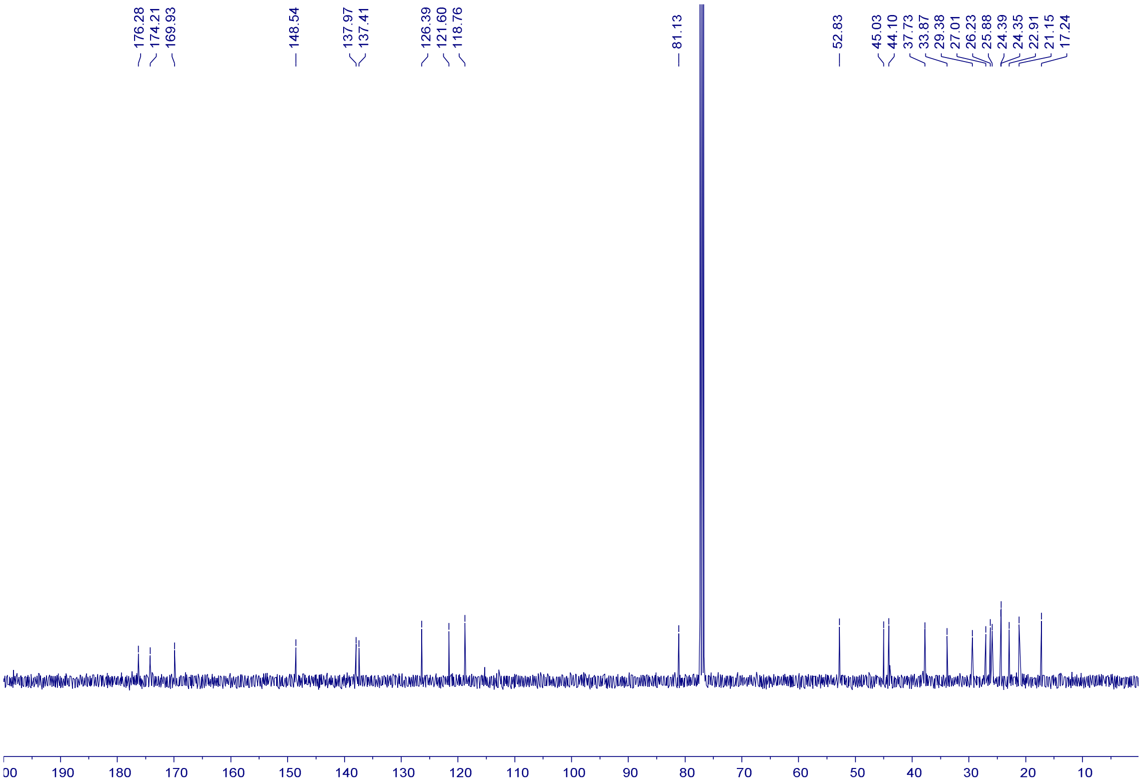

1r ^{1}H NMR (400 MHz, $CDCl_3$)

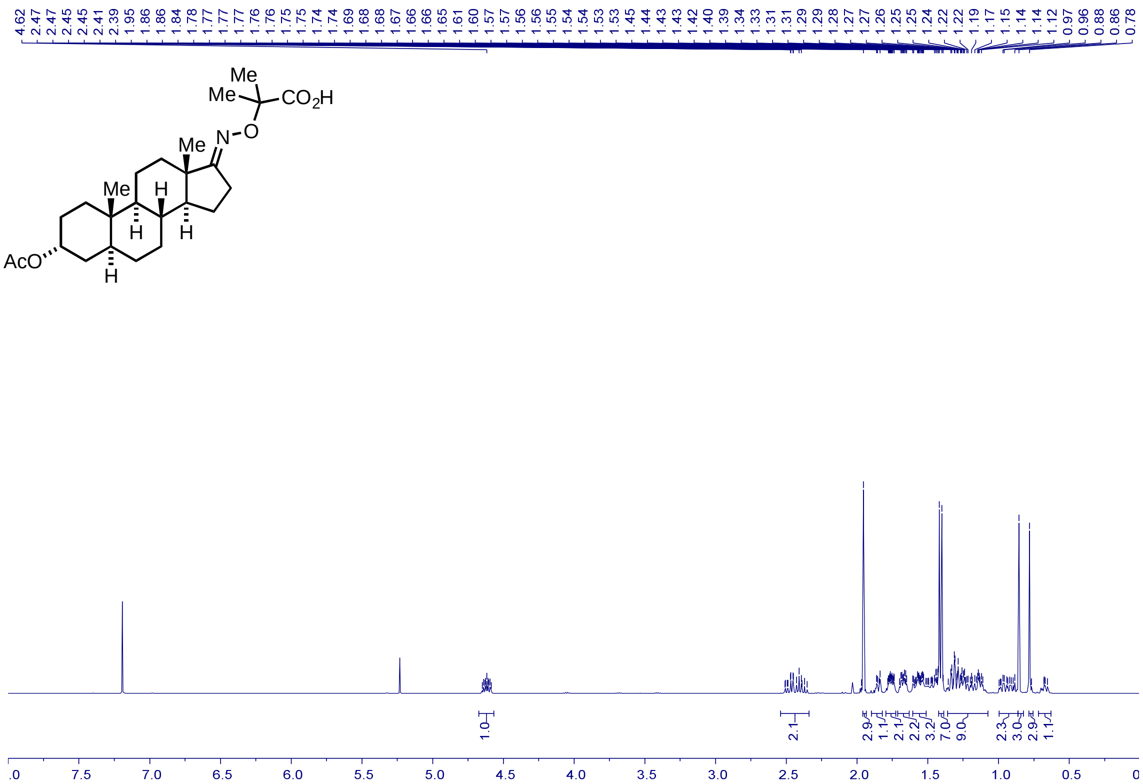

1r ^{13}C NMR (101 MHz, $CDCl_3$)

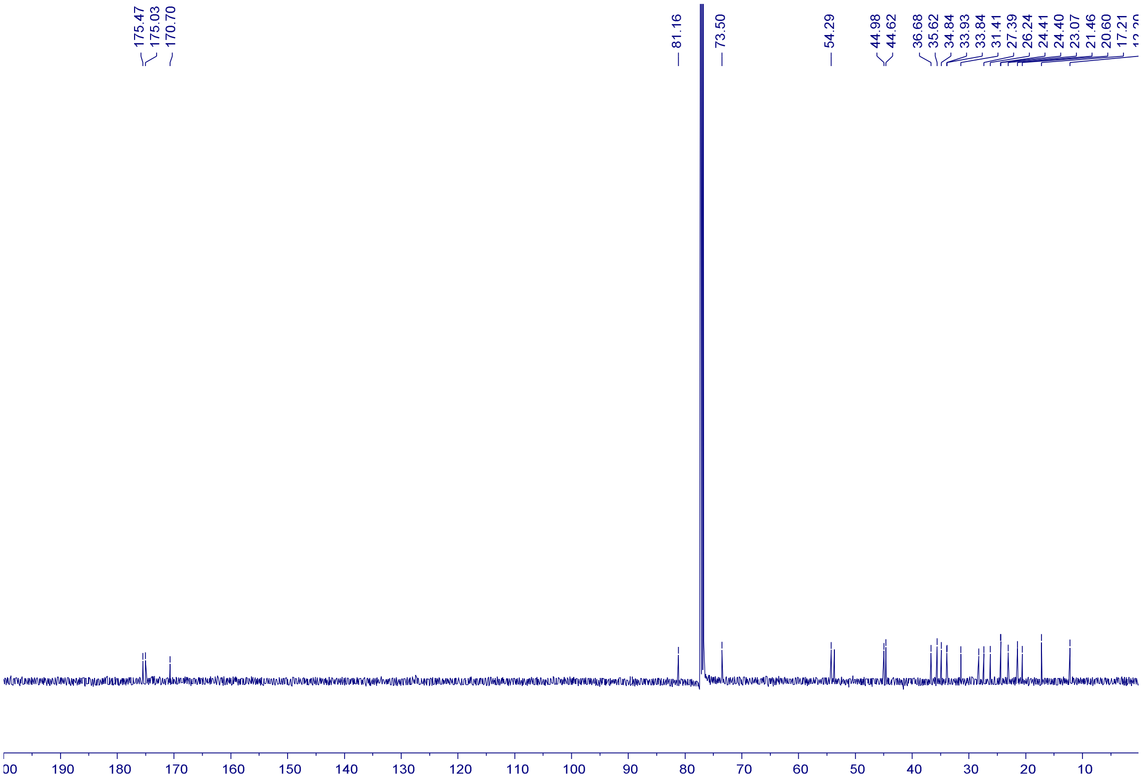

1s ^{1}H NMR (400 MHz, $CDCl_3$)

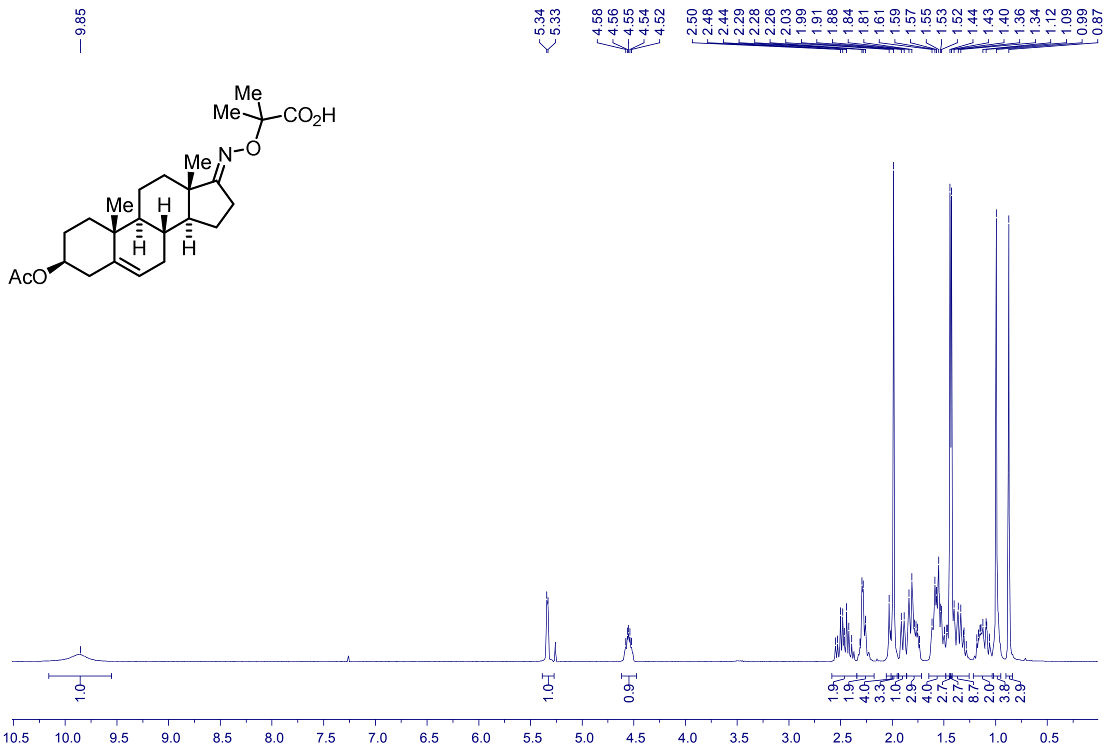

1s ^{13}C NMR (101 MHz, $CDCl_3$)

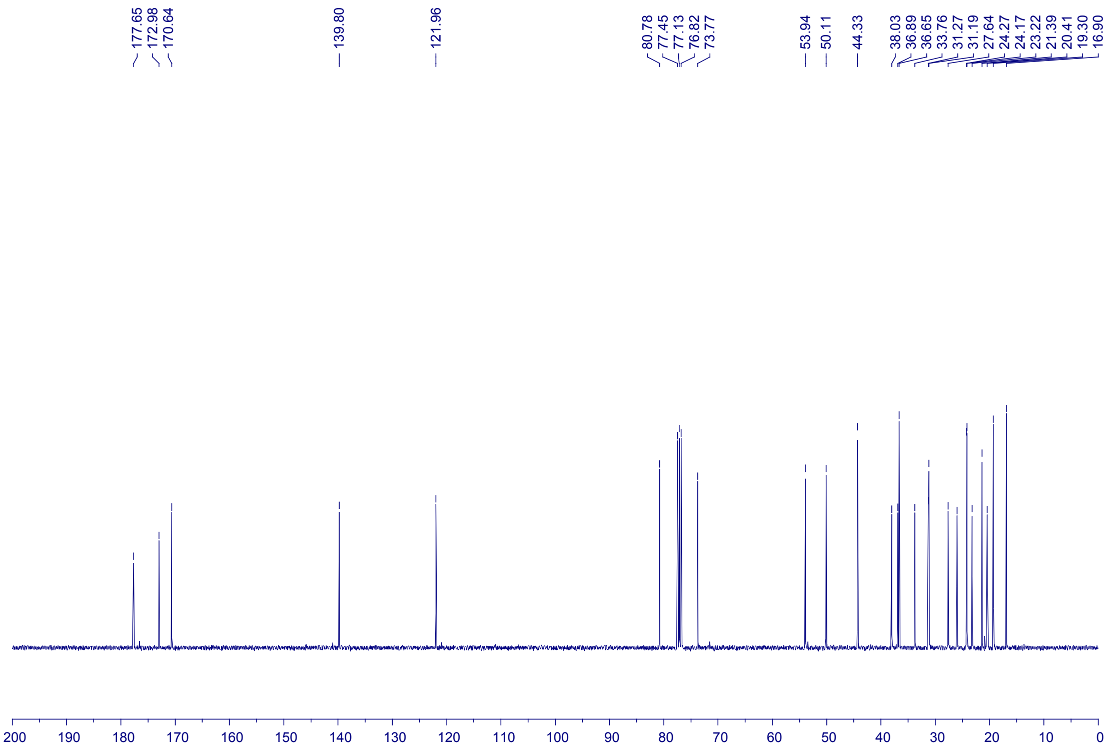

1t ^{1}H NMR (400 MHz, $CDCl_3$)

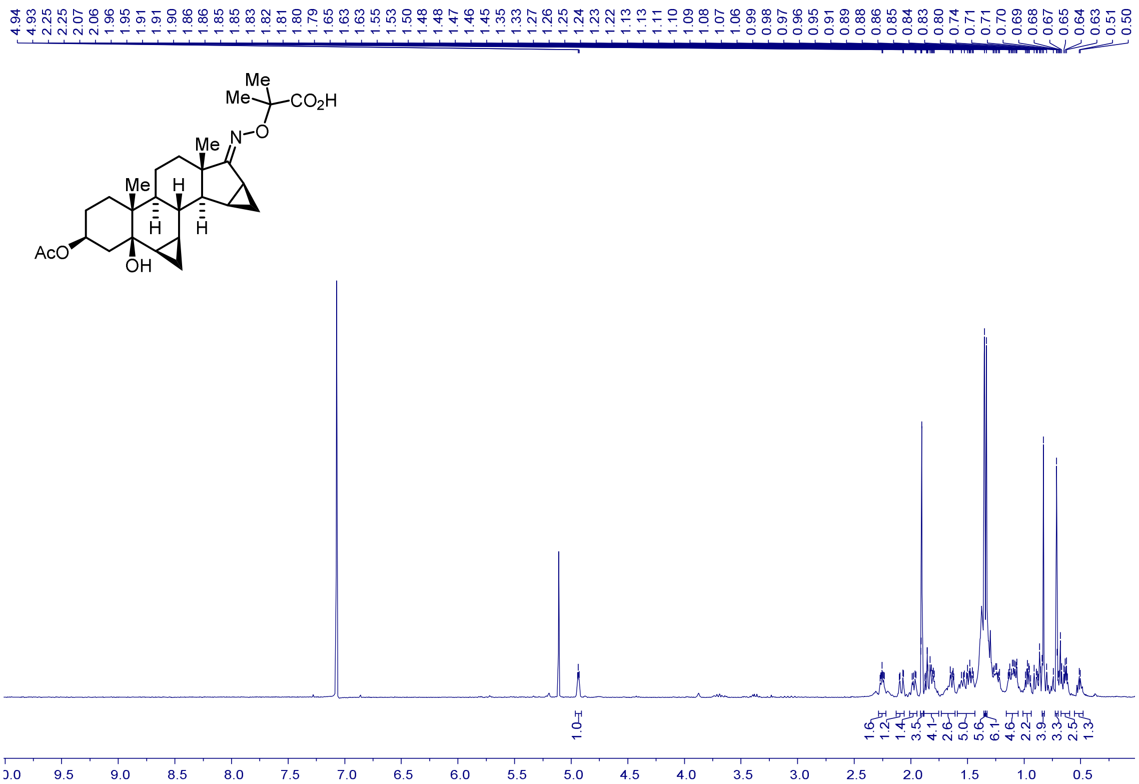

1t ^{13}C NMR (101 MHz, $CDCl_3$)

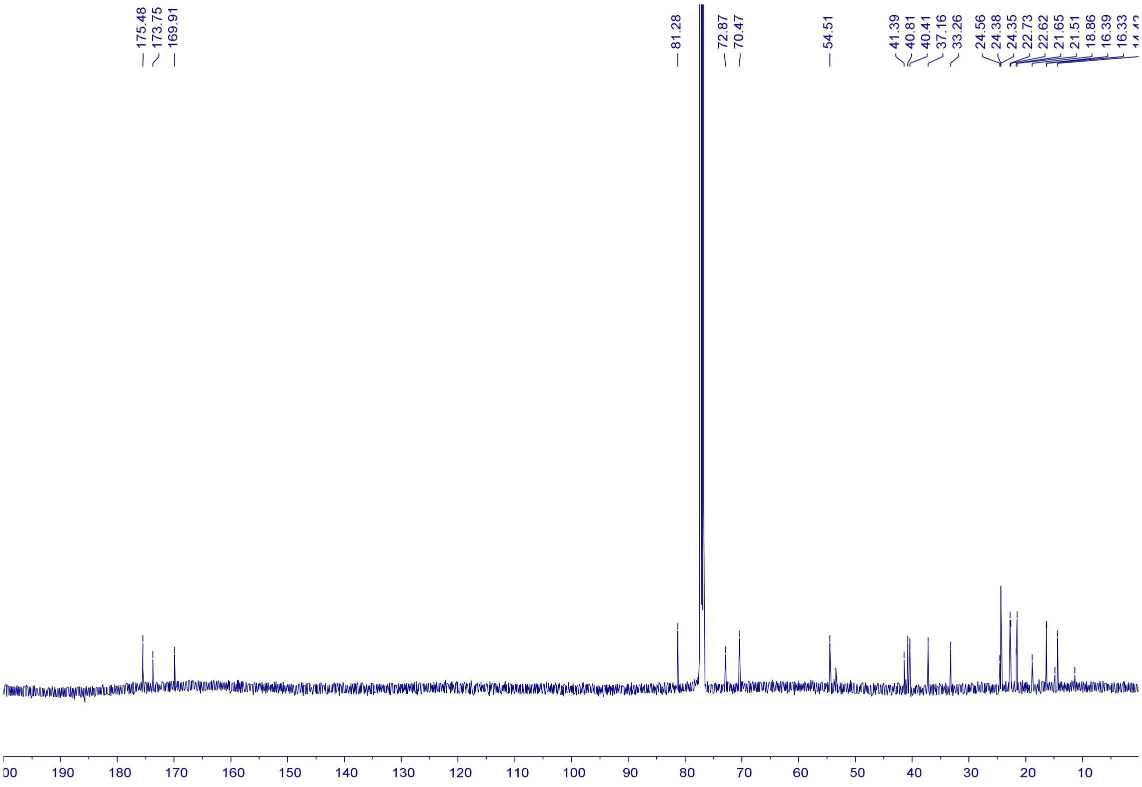

1u ^{1}H NMR (400 MHz, $CDCl_3$)

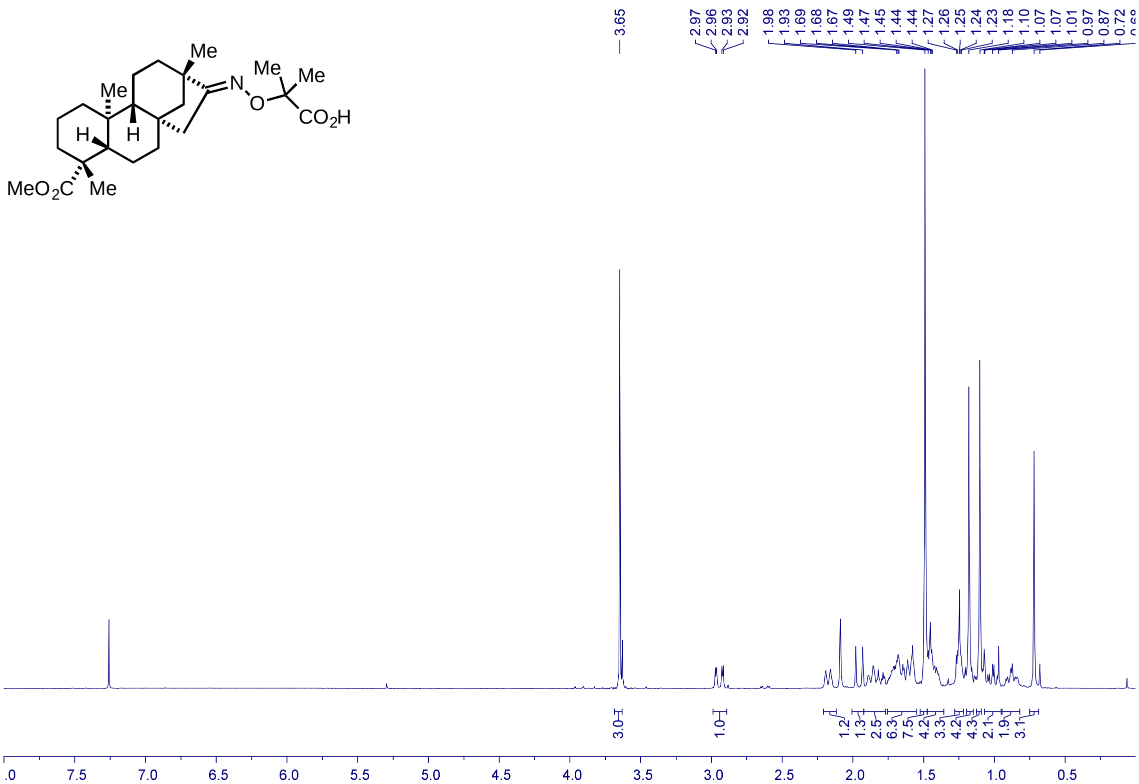

1u ^{13}C NMR (101 MHz, $CDCl_3$)

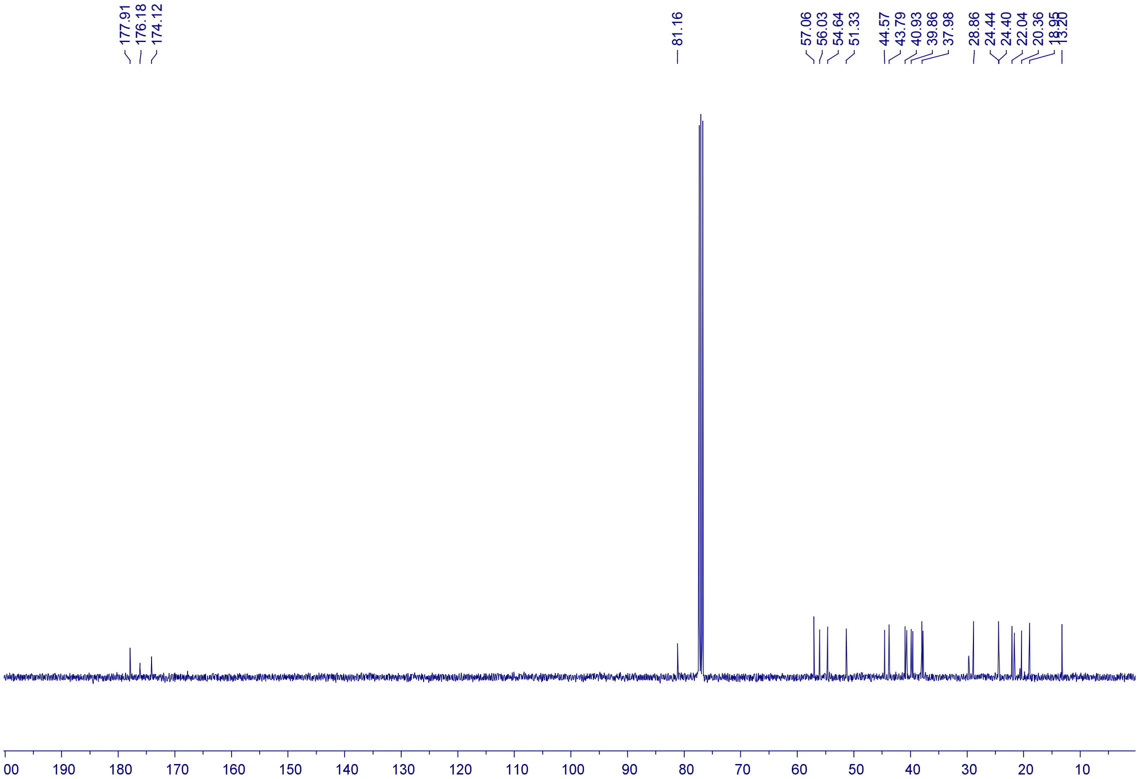

6a ^{1}H NMR (400 MHz, $CDCl_3$)

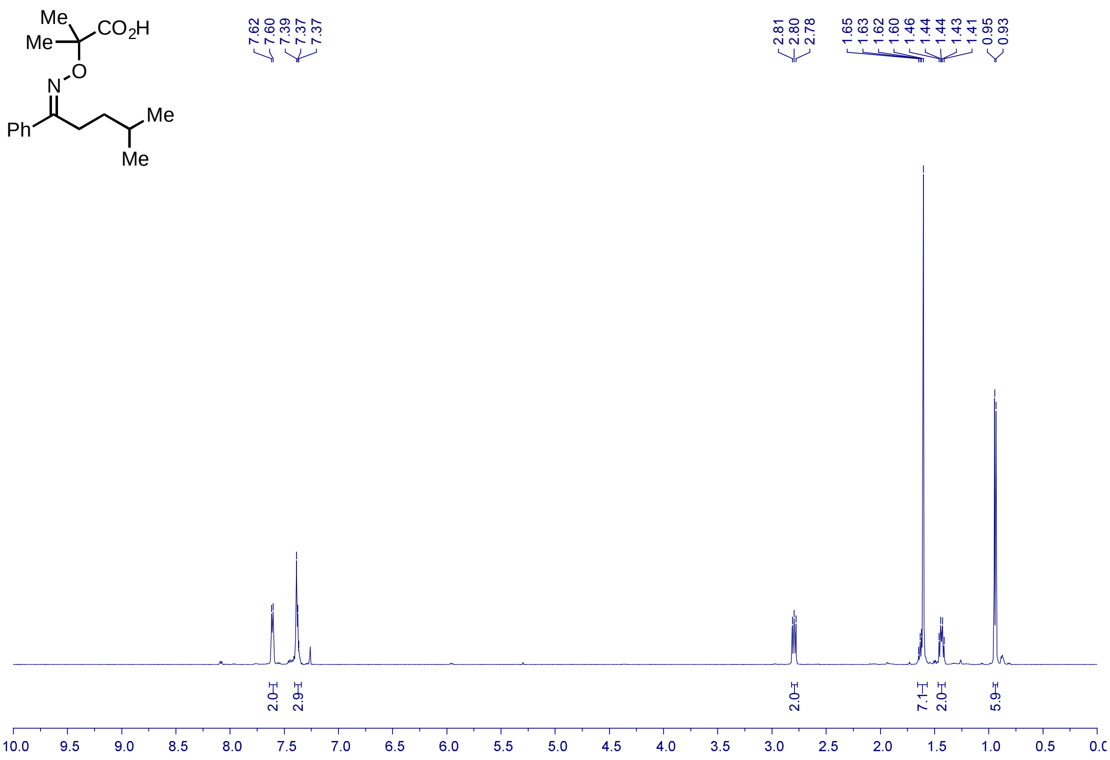

6a ^{13}C NMR (101 MHz, $CDCl_3$)

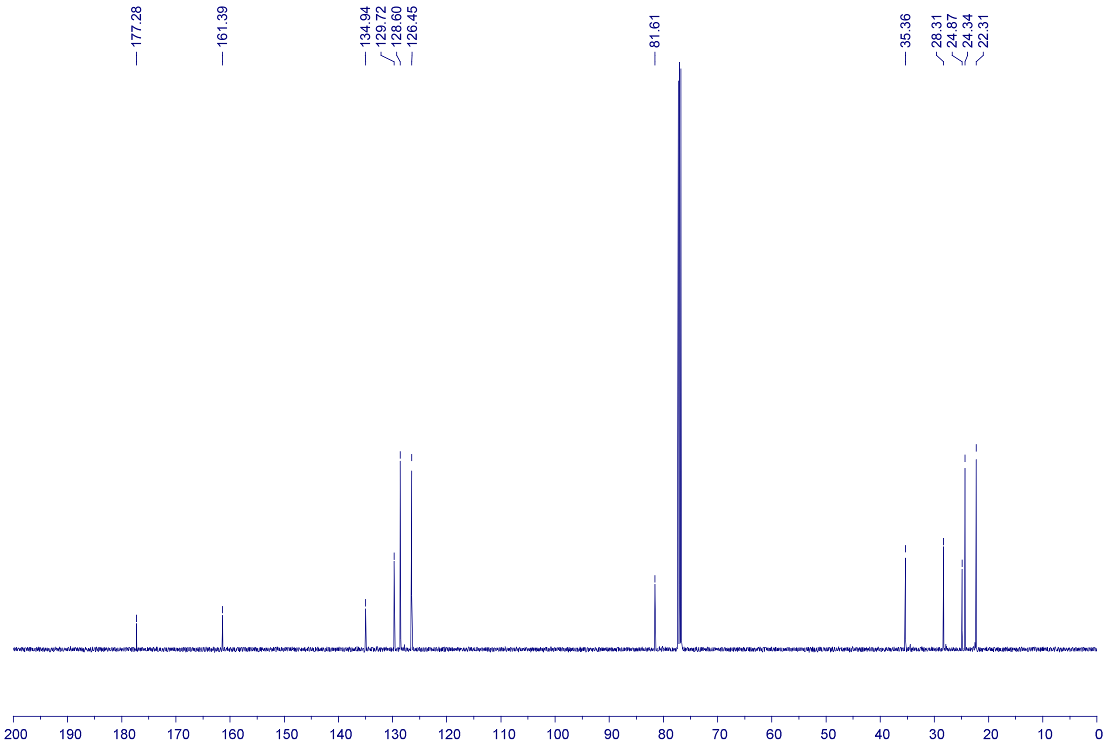

6b ^{1}H NMR (400 MHz, $CDCl_3$)

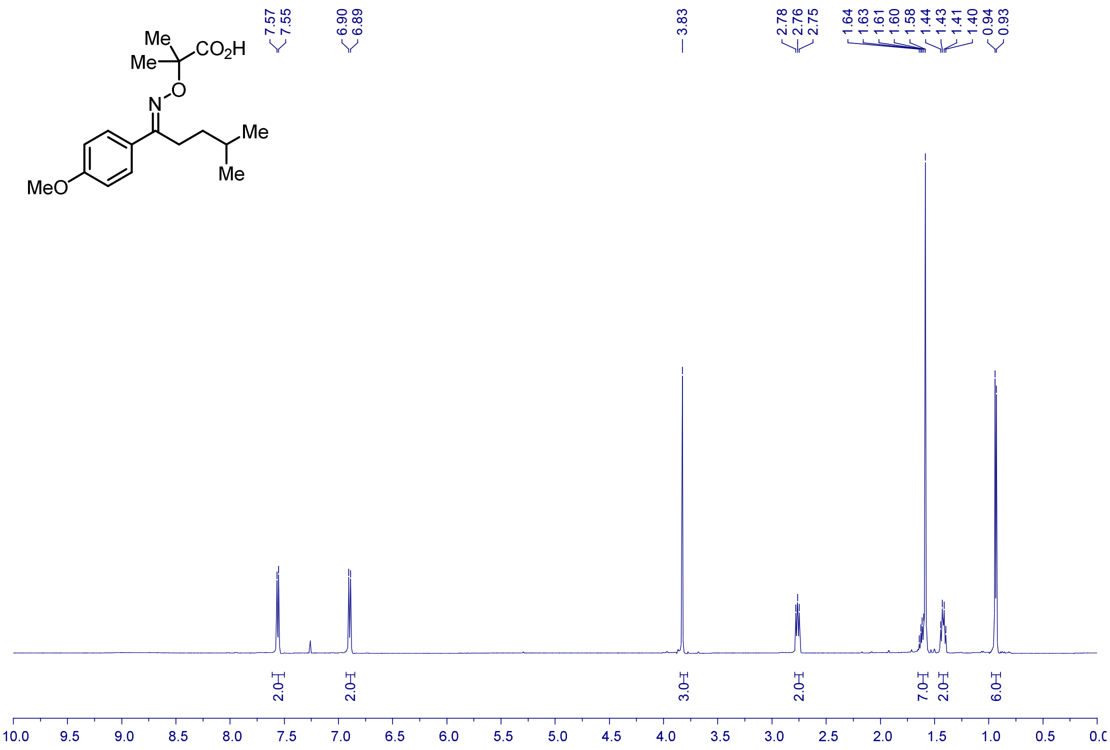

6b ^{13}C NMR (101 MHz, $CDCl_3$)

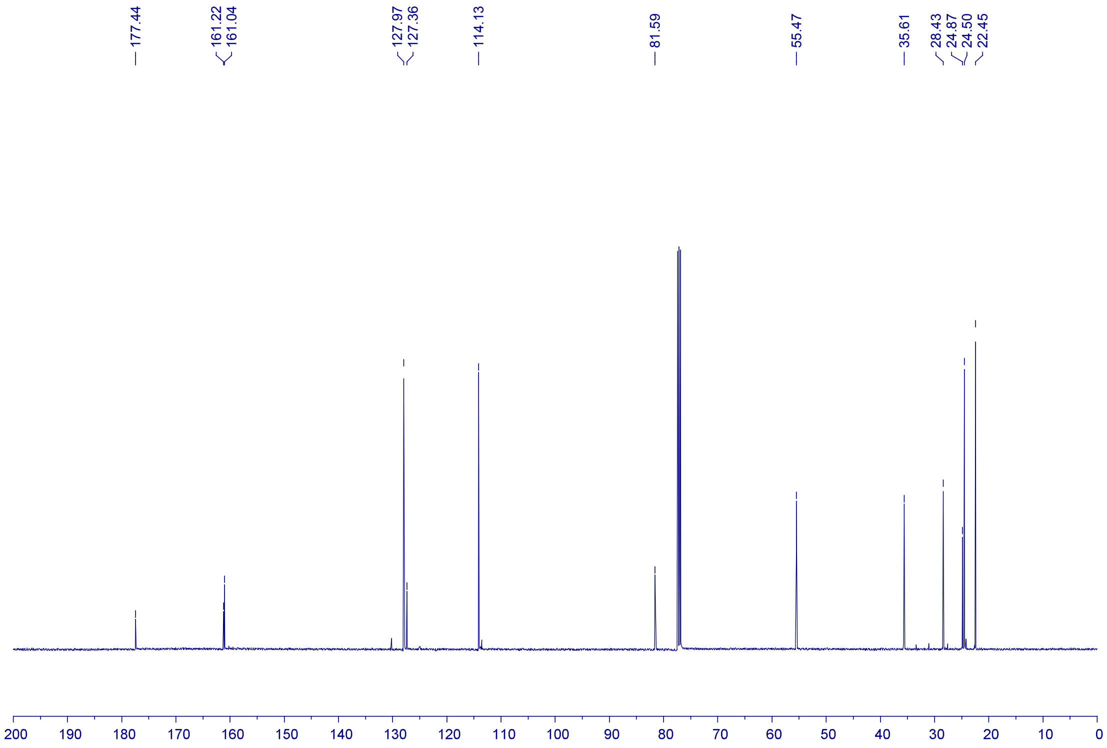

6c ^{1}H NMR (400 MHz, $CDCl_3$)

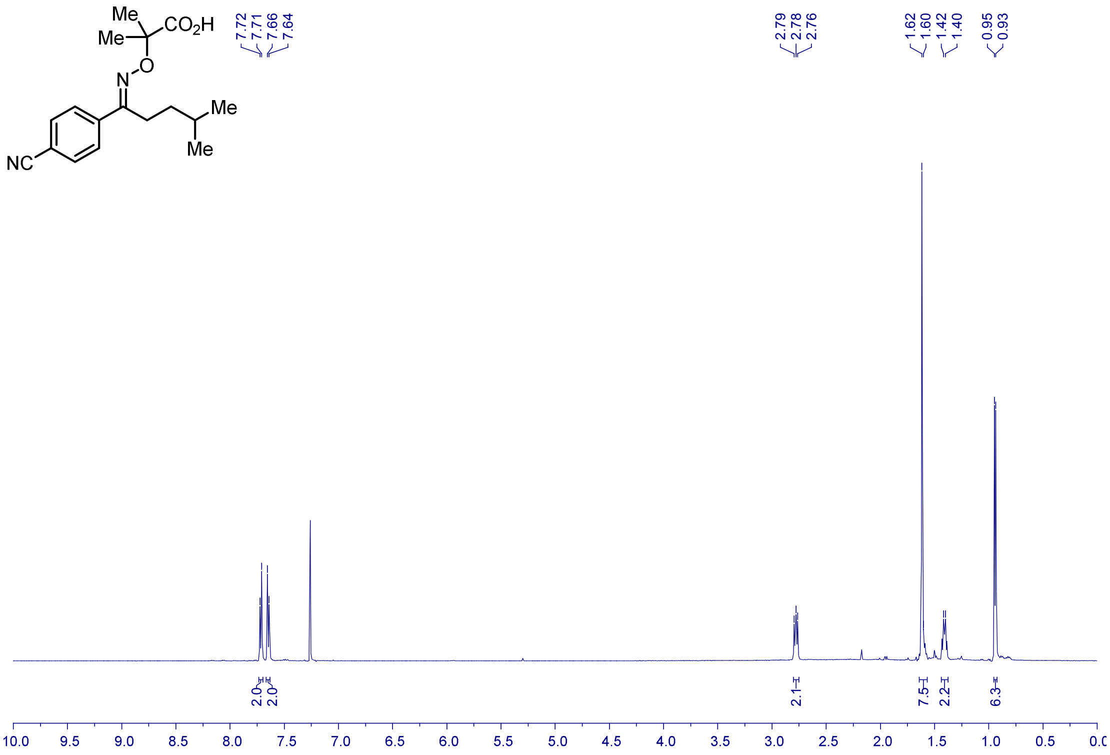

6c ^{13}C NMR (101 MHz, $CDCl_3$)

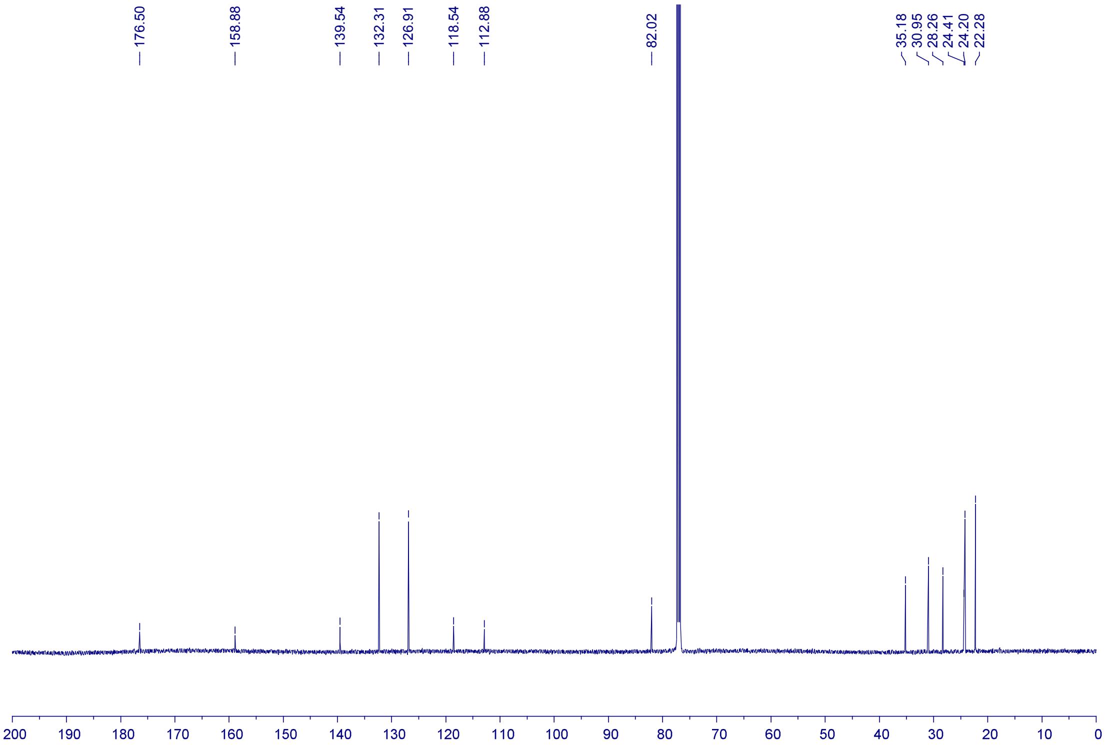

6d ^{1}H NMR (400 MHz, $CDCl_3$)

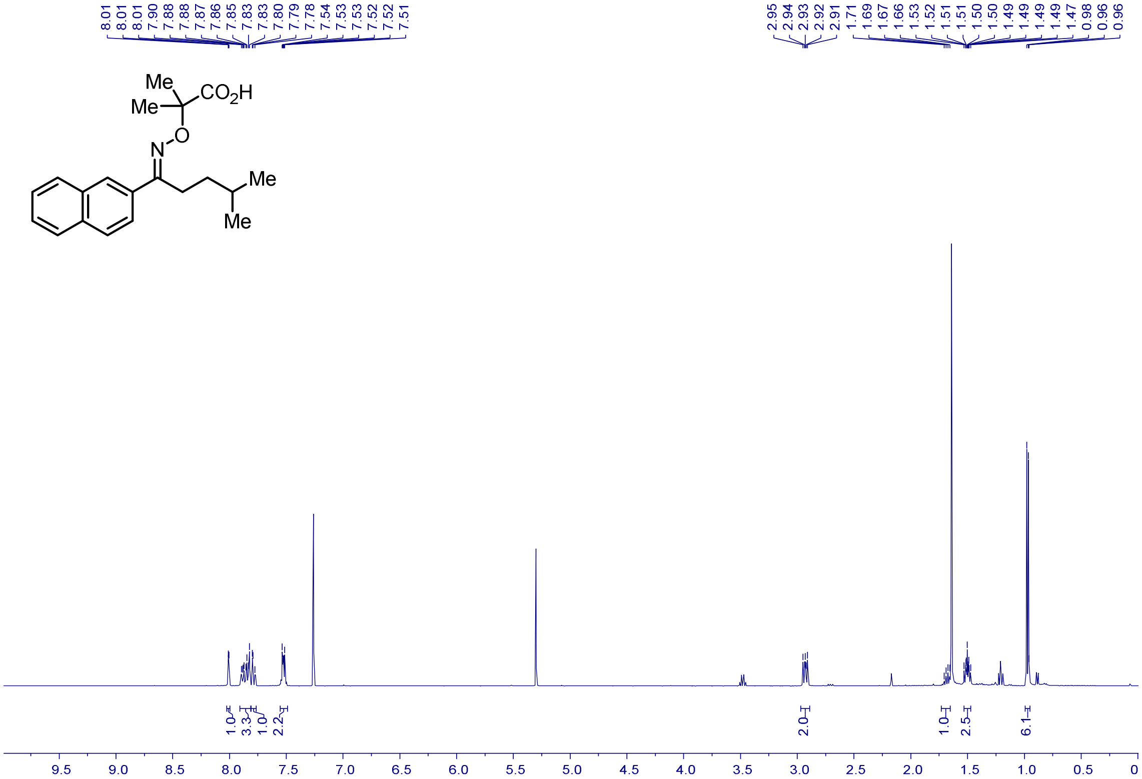

6d ^{13}C NMR (101 MHz, $CDCl_3$)

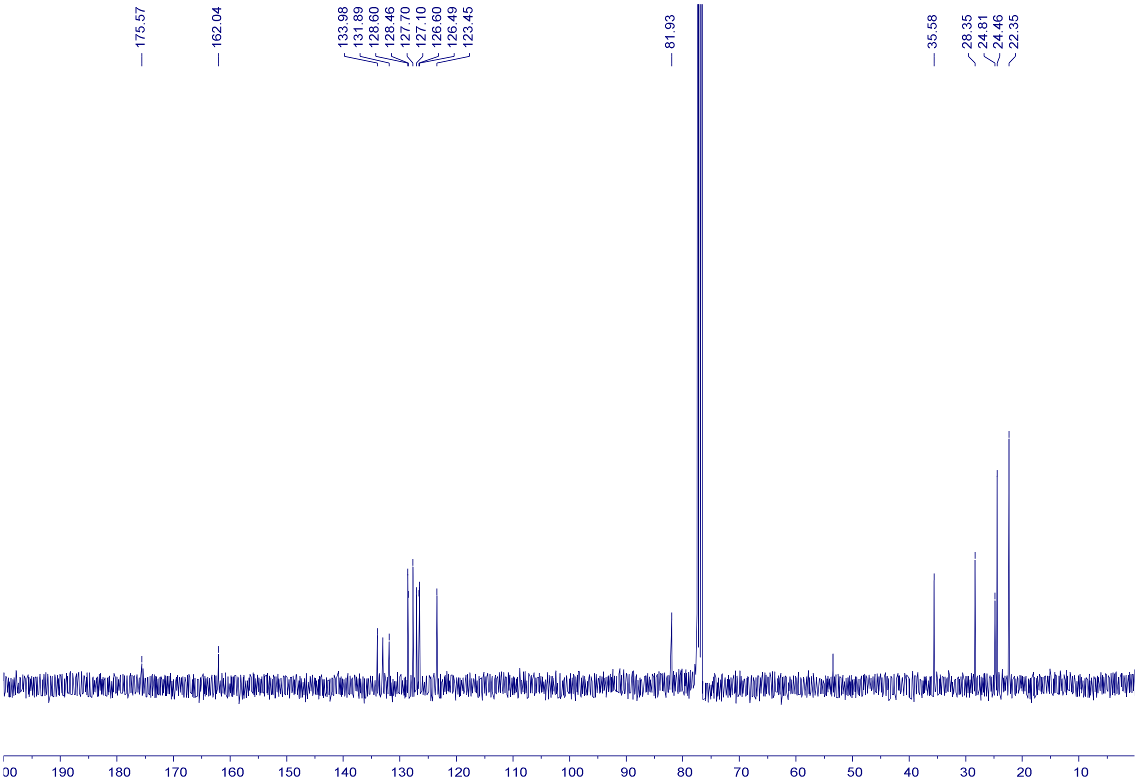

6e ^{1}H NMR (400 MHz, $CDCl_3$)

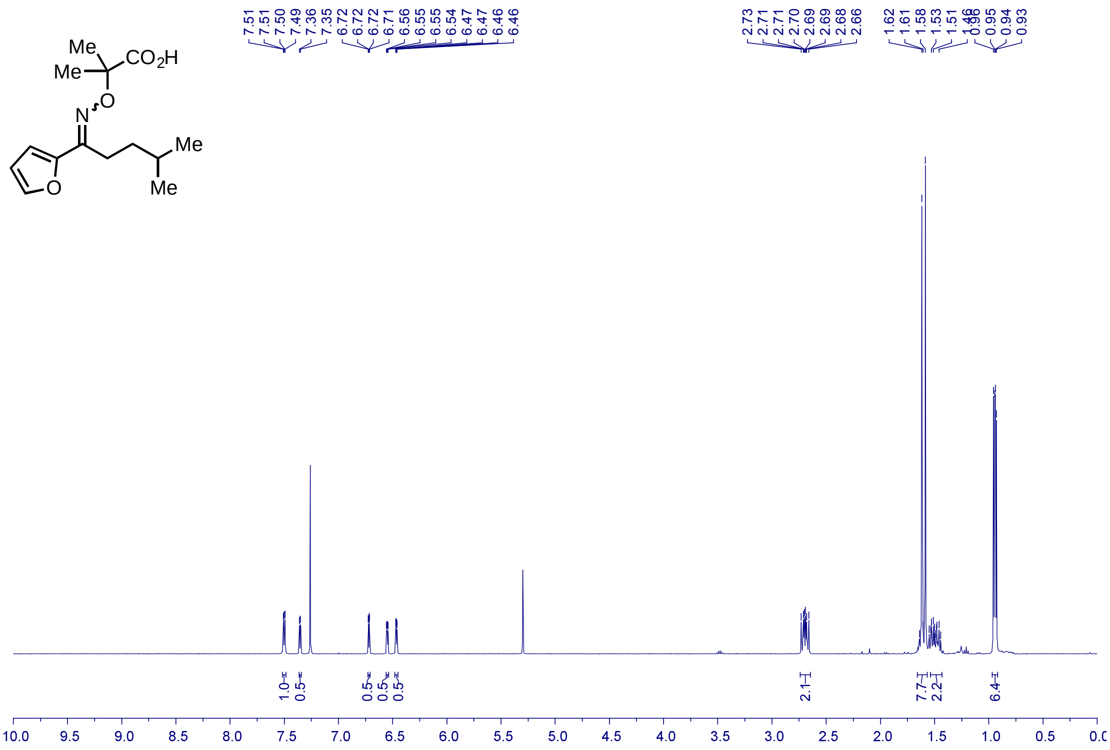

6e ^{13}C NMR (101 MHz, $CDCl_3$)

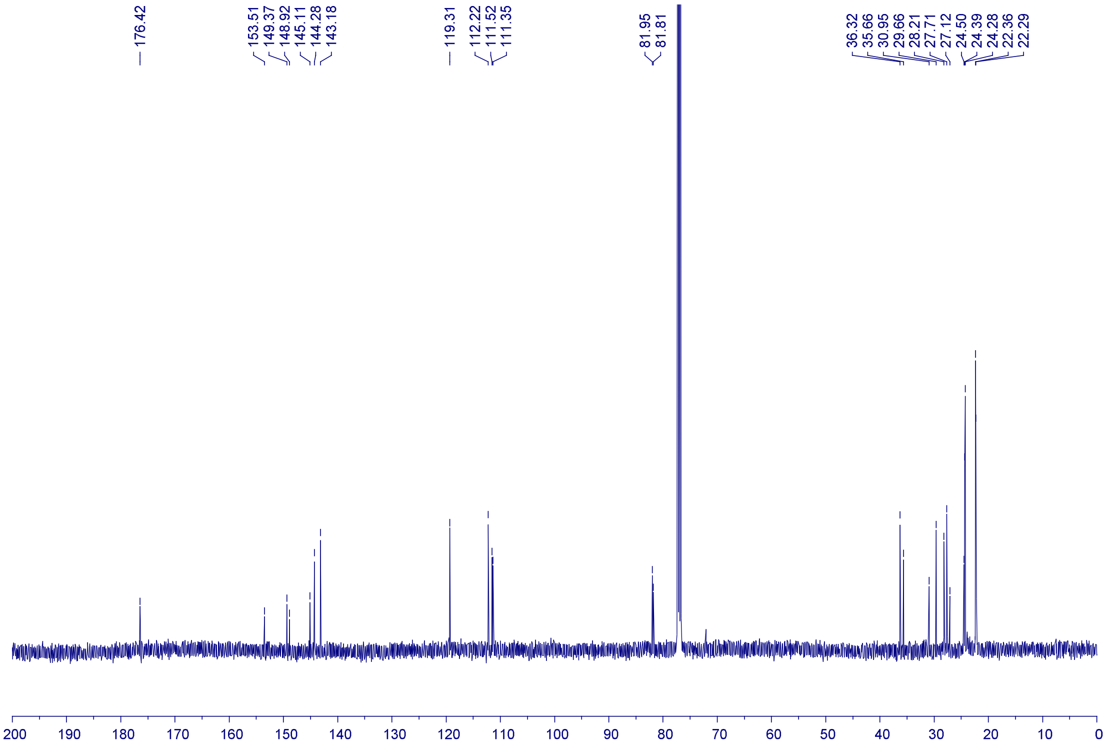

6f ^{1}H NMR (400 MHz, $CDCl_3$)

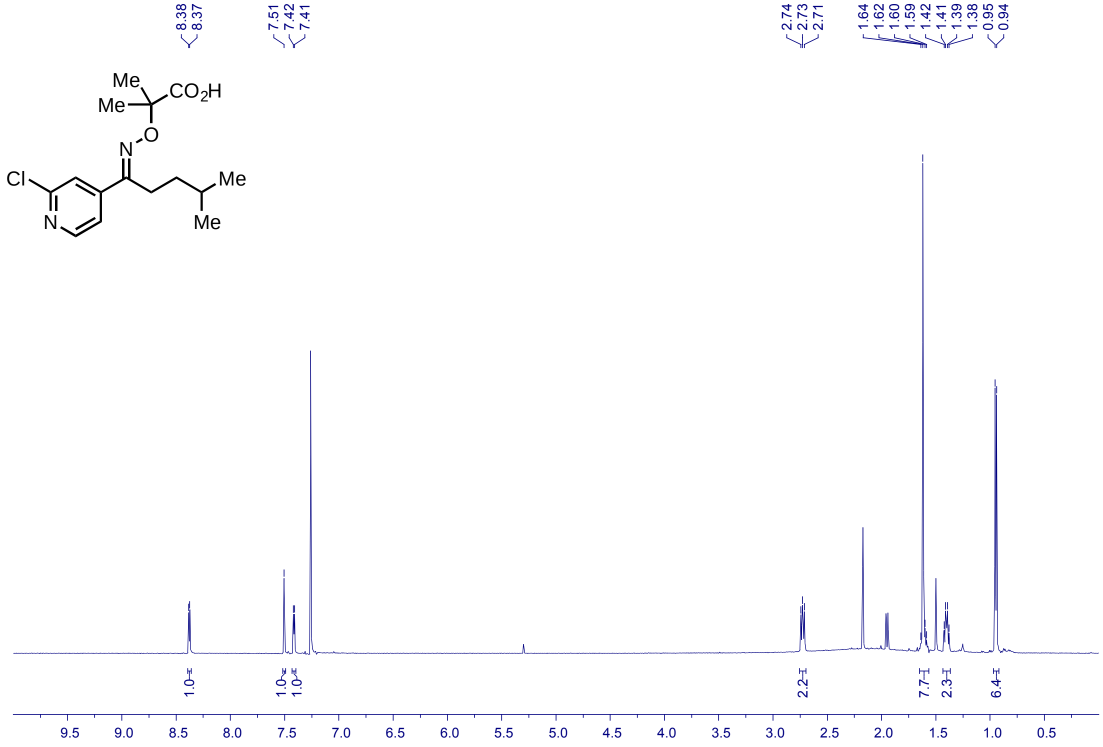

6f ^{13}C NMR (101 MHz, $CDCl_3$)

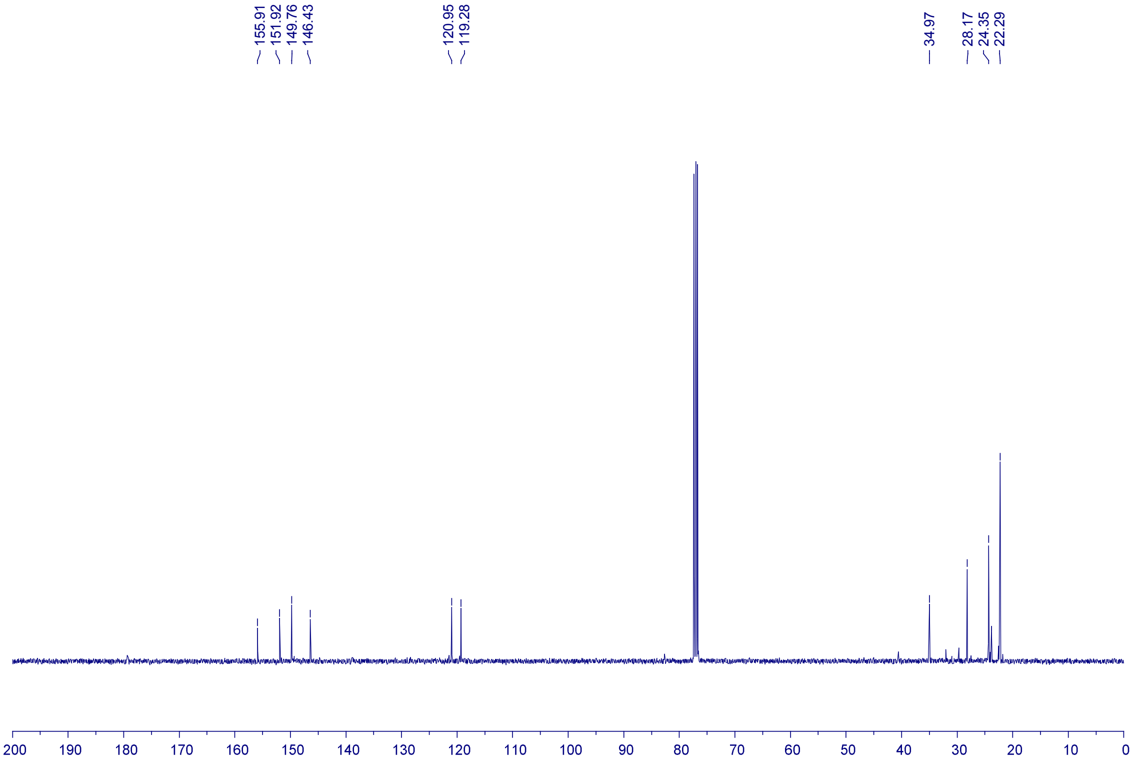

6g ^{1}H NMR (400 MHz, $CDCl_3$)

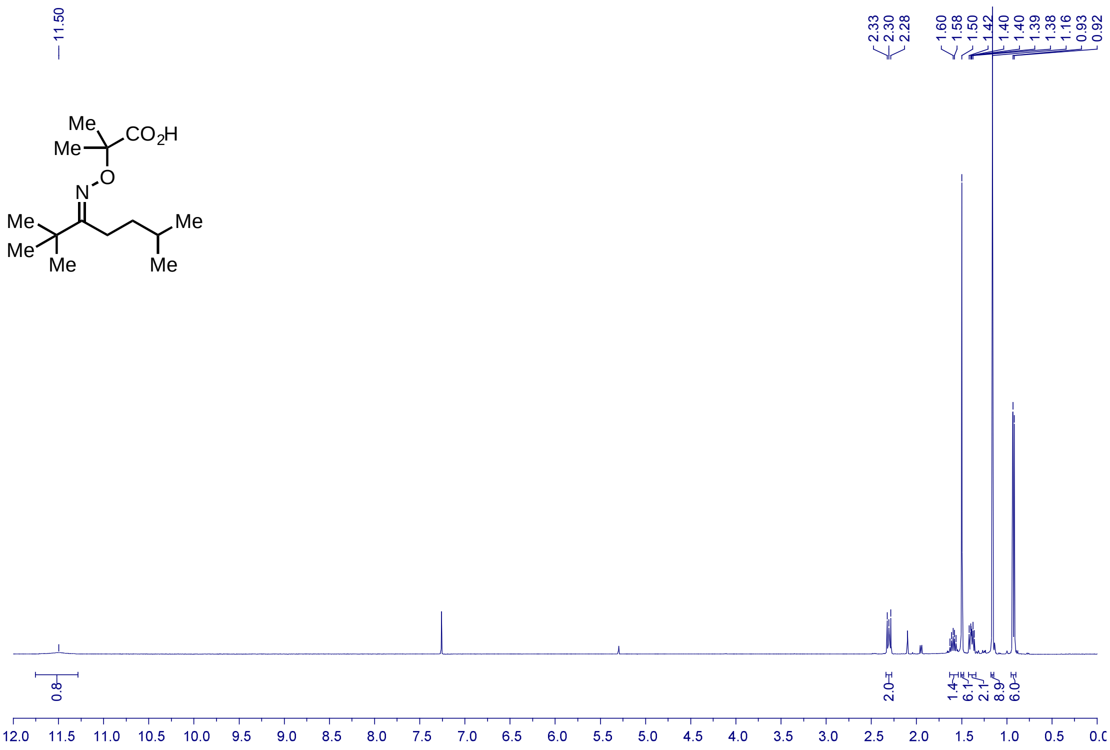

5g ^{13}C NMR (101 MHz, $CDCl_3$)

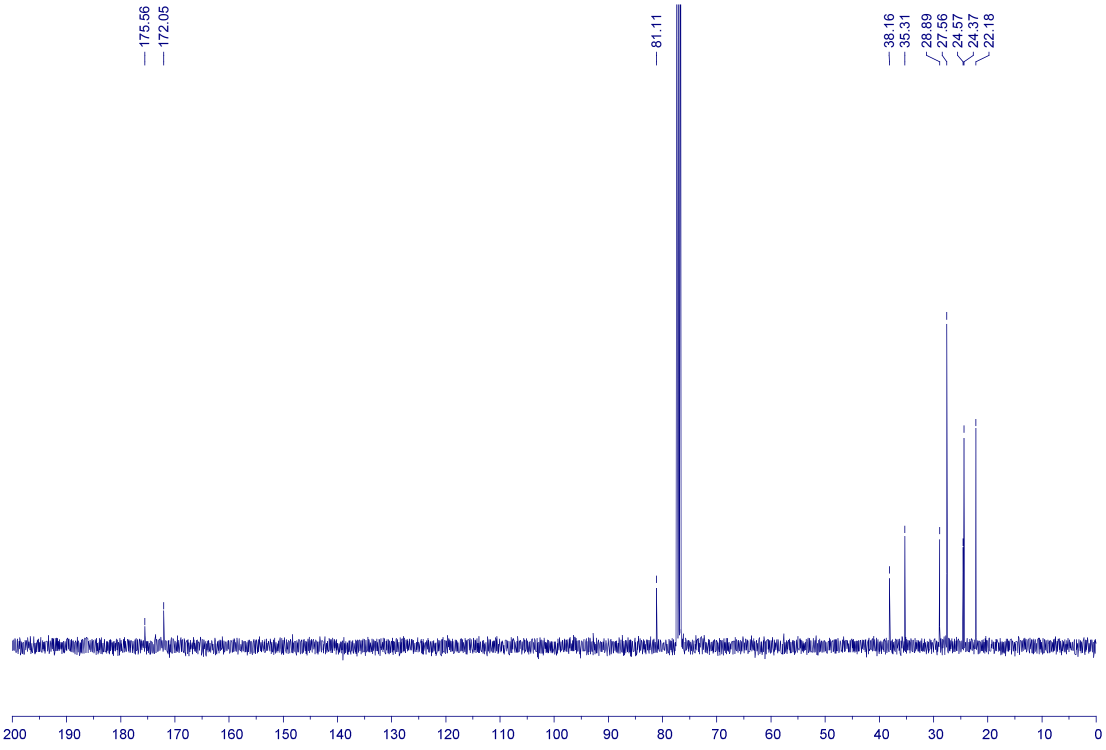

6h ^{1}H NMR (400 MHz, $CDCl_3$)

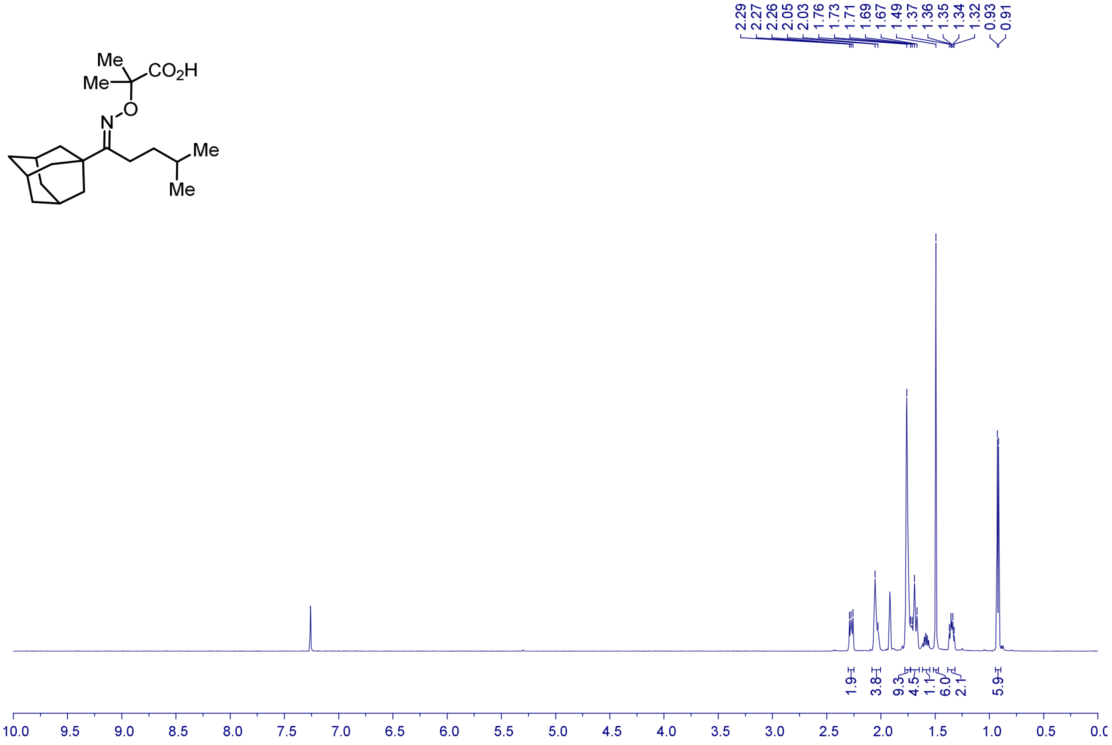

6h ^{13}C NMR (101 MHz, $CDCl_3$)

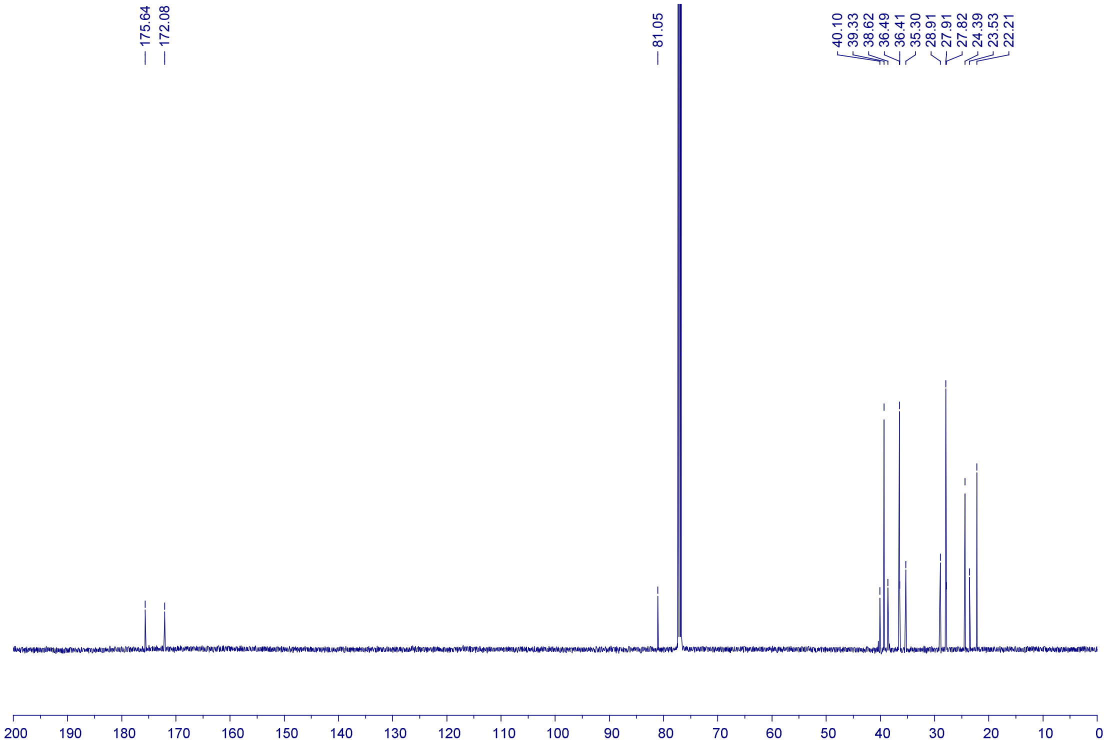

6i ^{1}H NMR (500 MHz, $CDCl_3$)

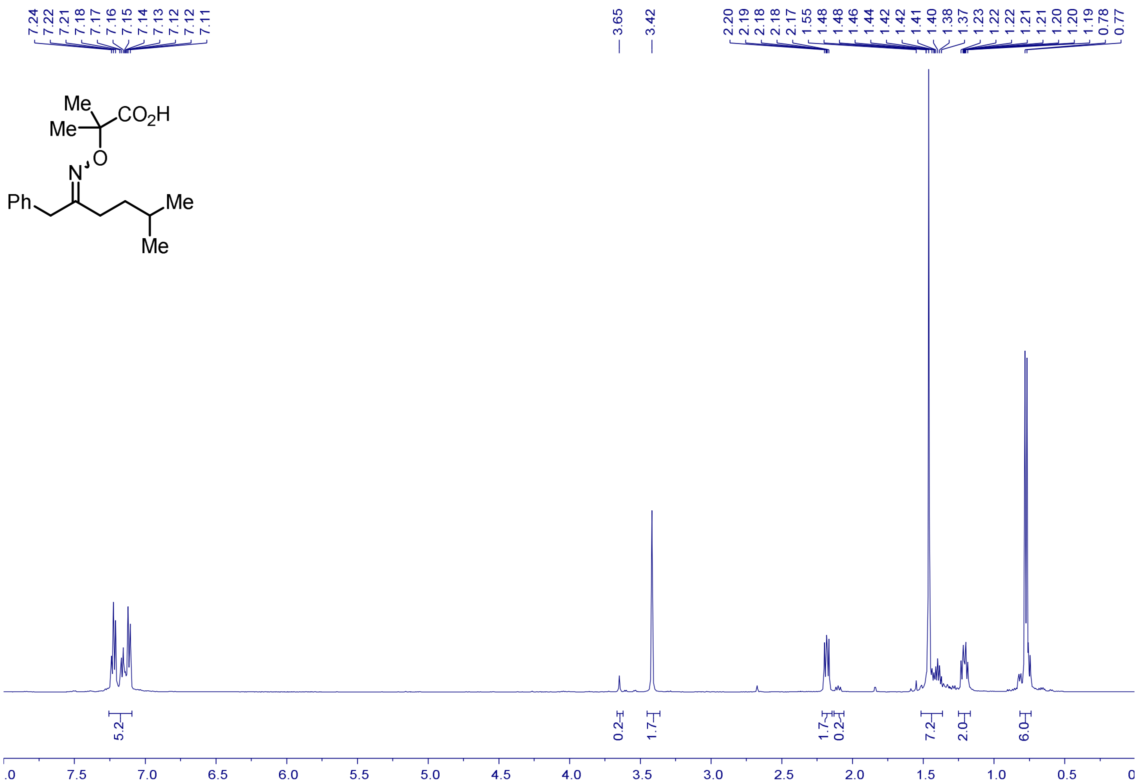

6i ^{13}C NMR (101 MHz, $CDCl_3$)

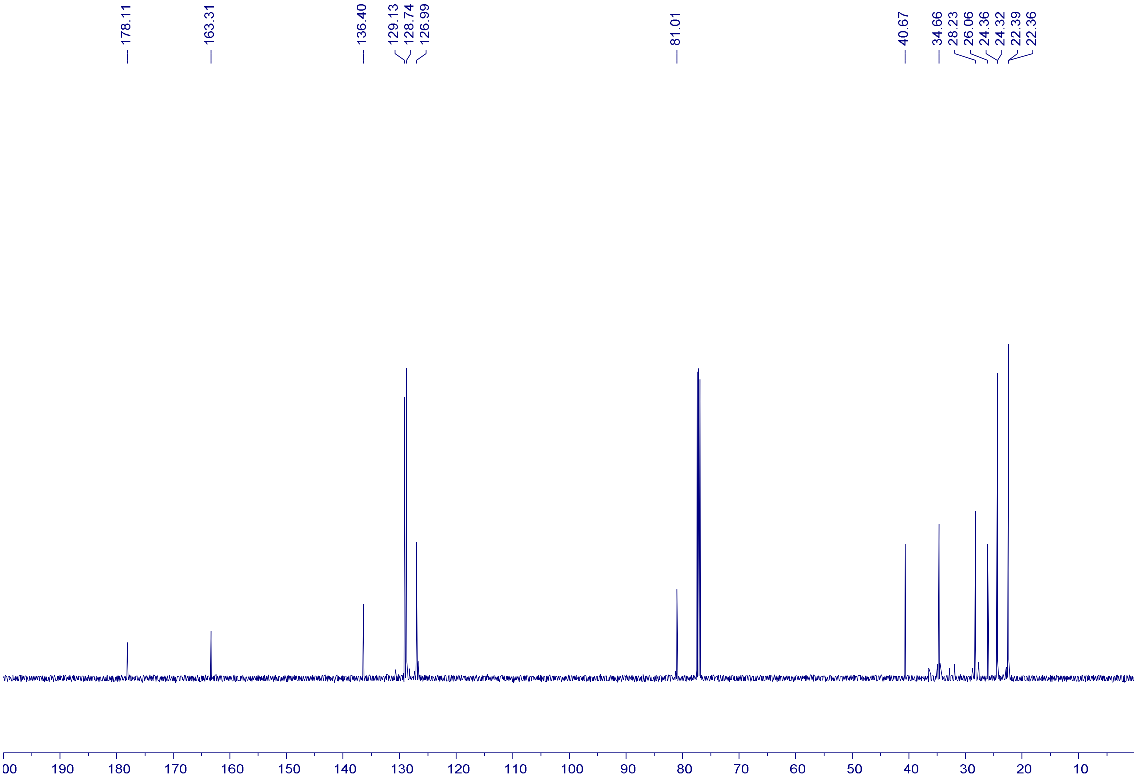

6j ^{1}H NMR (400 MHz, $CDCl_3$)

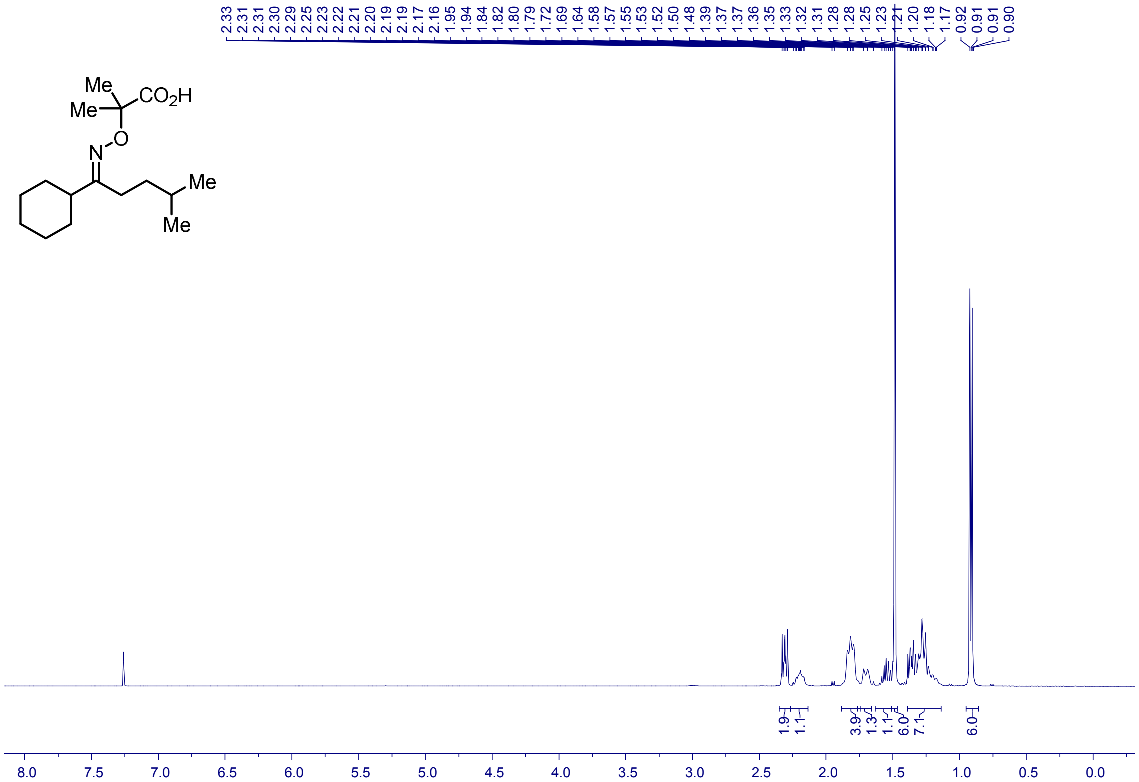

6j ^{13}C NMR (101 MHz, $CDCl_3$)

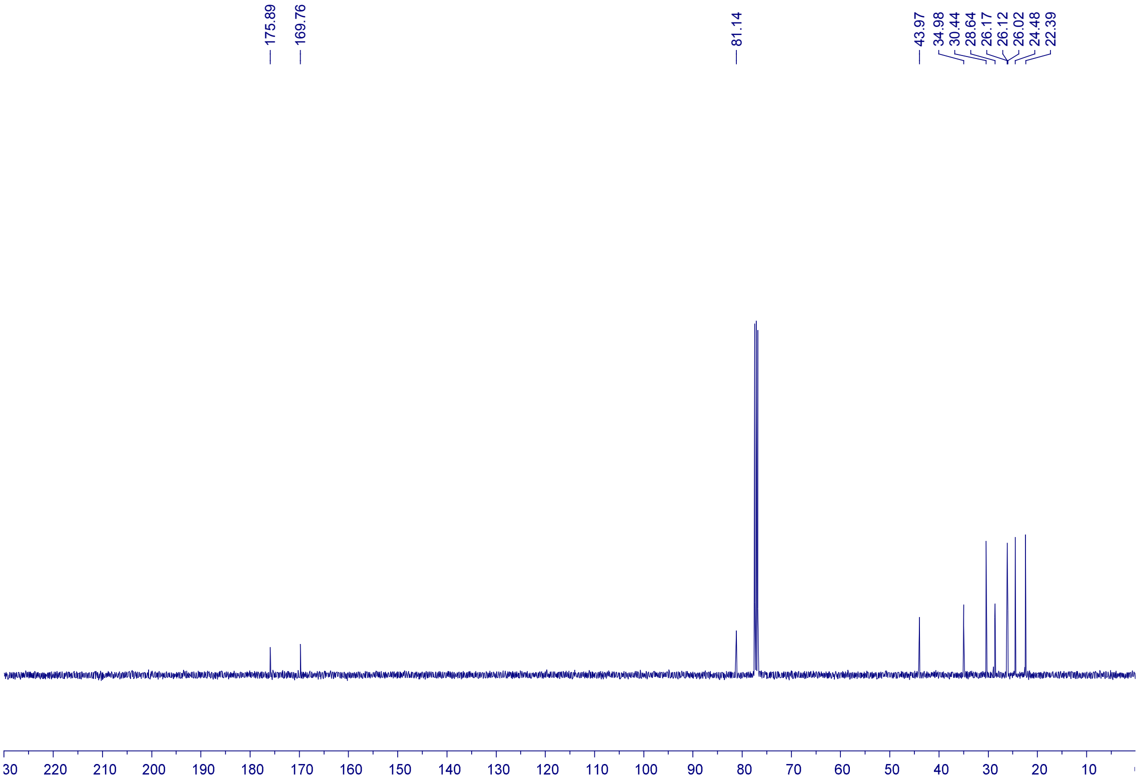

6k ^{1}H NMR (400 MHz, $CDCl_3$)

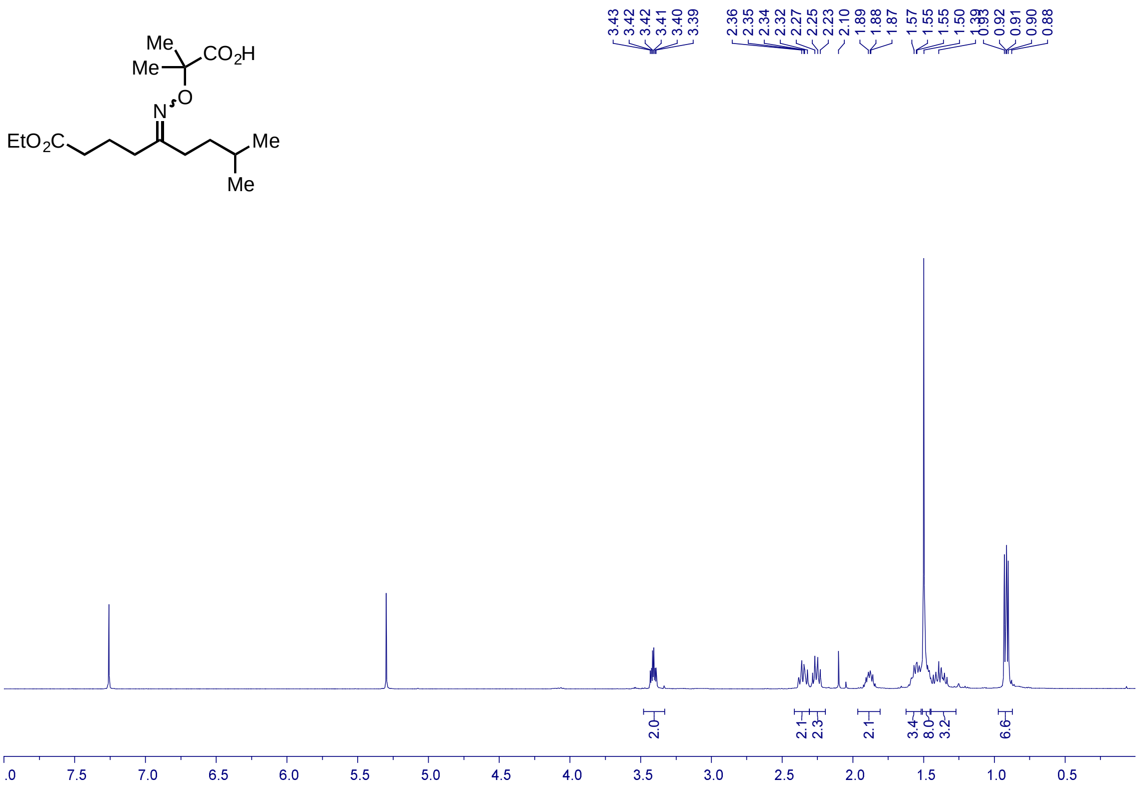

6k ^{13}C NMR (126 MHz, $CDCl_3$)

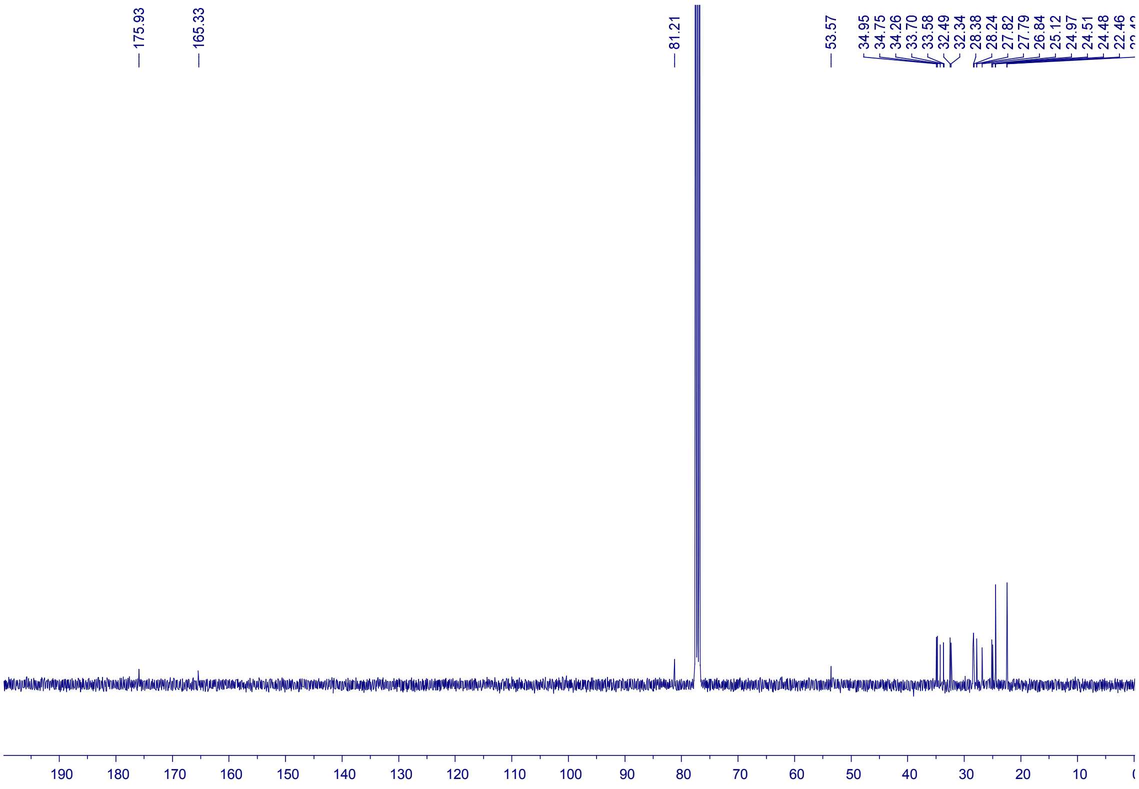

6l ^{1}H NMR (400 MHz, $CDCl_3$)

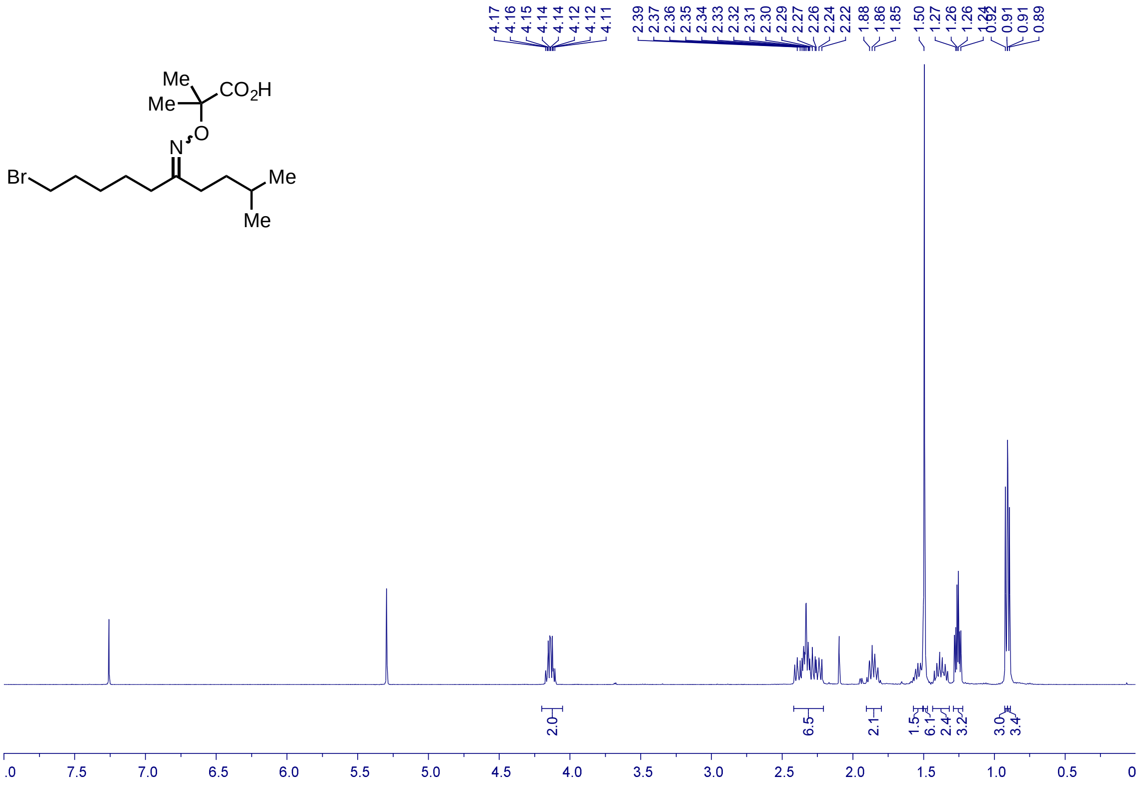

6l ^{13}C NMR (126 MHz, $CDCl_3$)

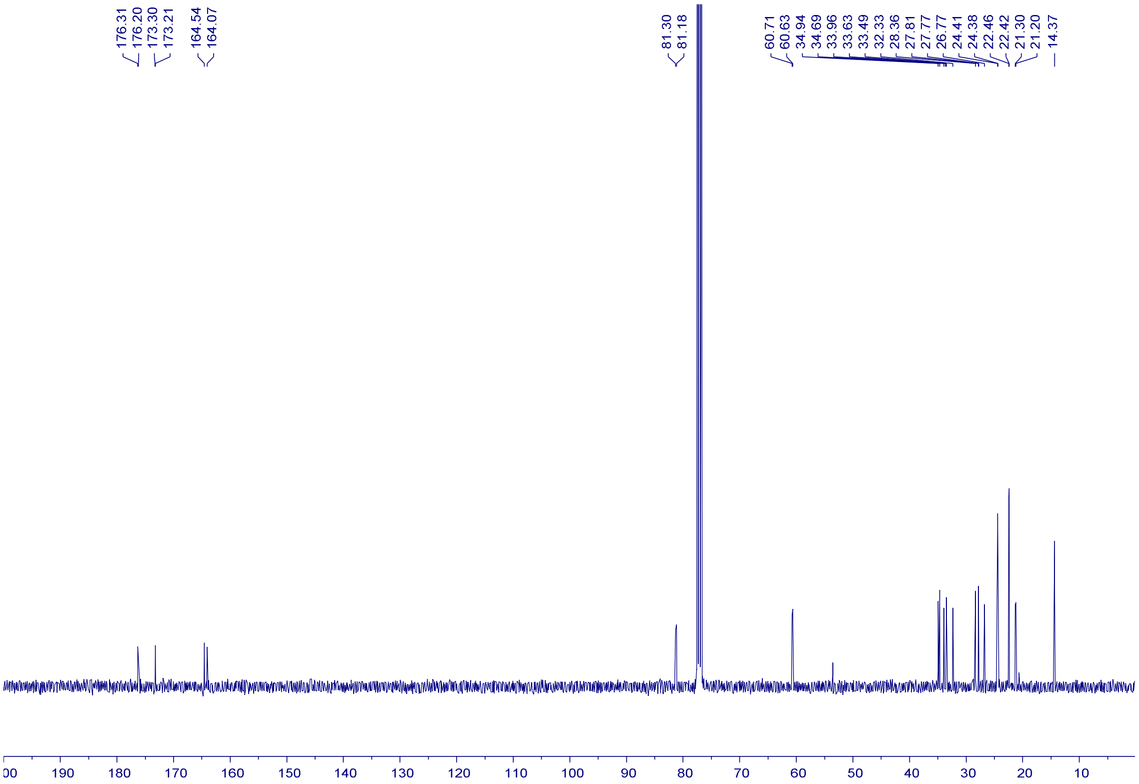

6m ^{1}H NMR (400 MHz, $CDCl_3$)

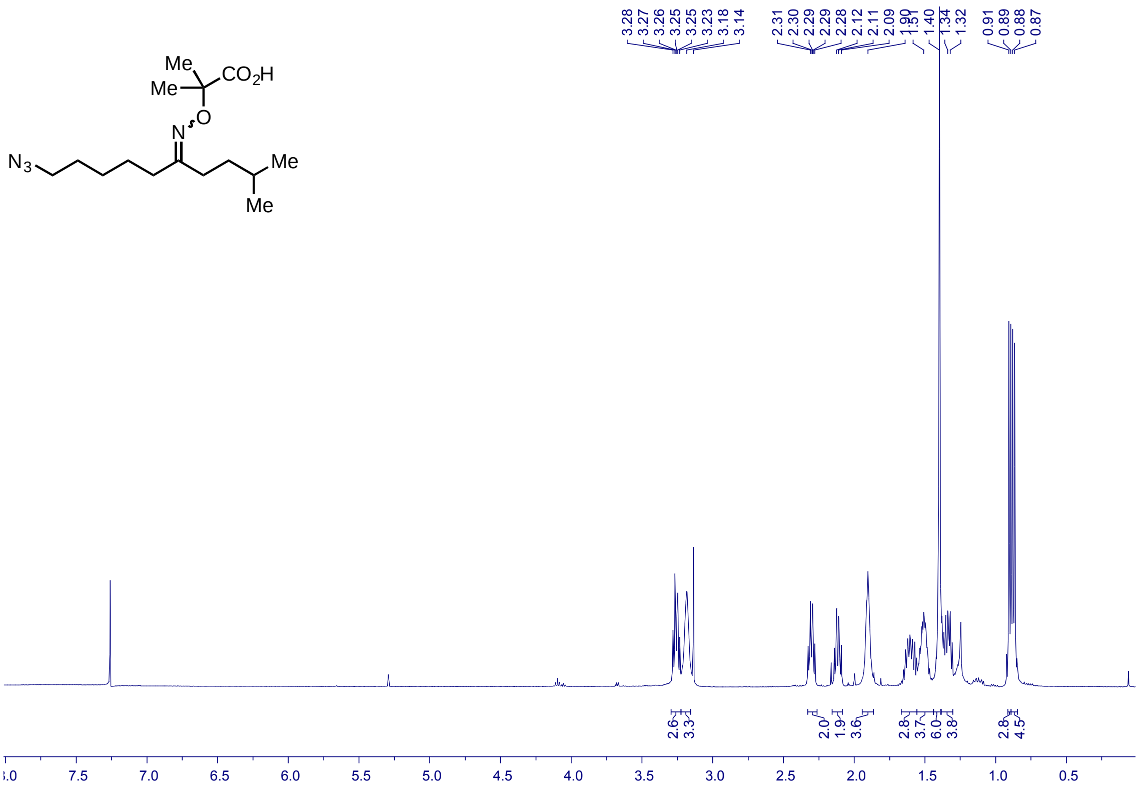

6m ^{13}C NMR (126 MHz, $CDCl_3$)

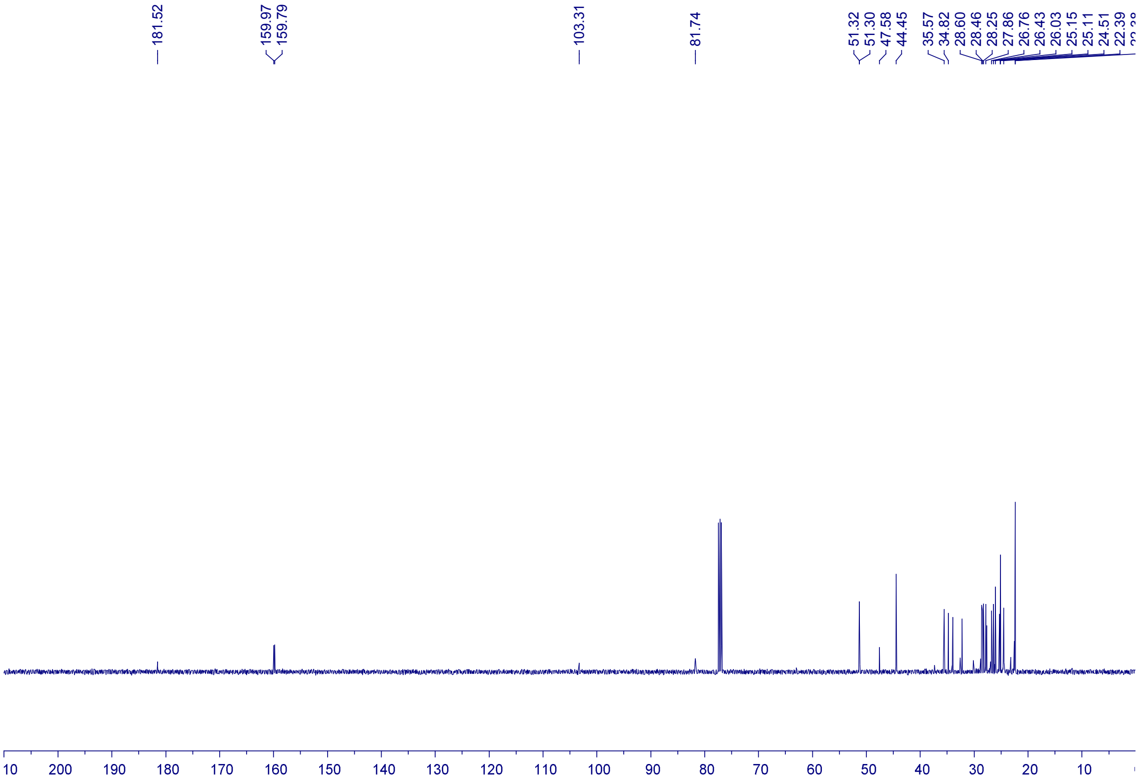

6n ^{1}H NMR (400 MHz, $CDCl_3$)

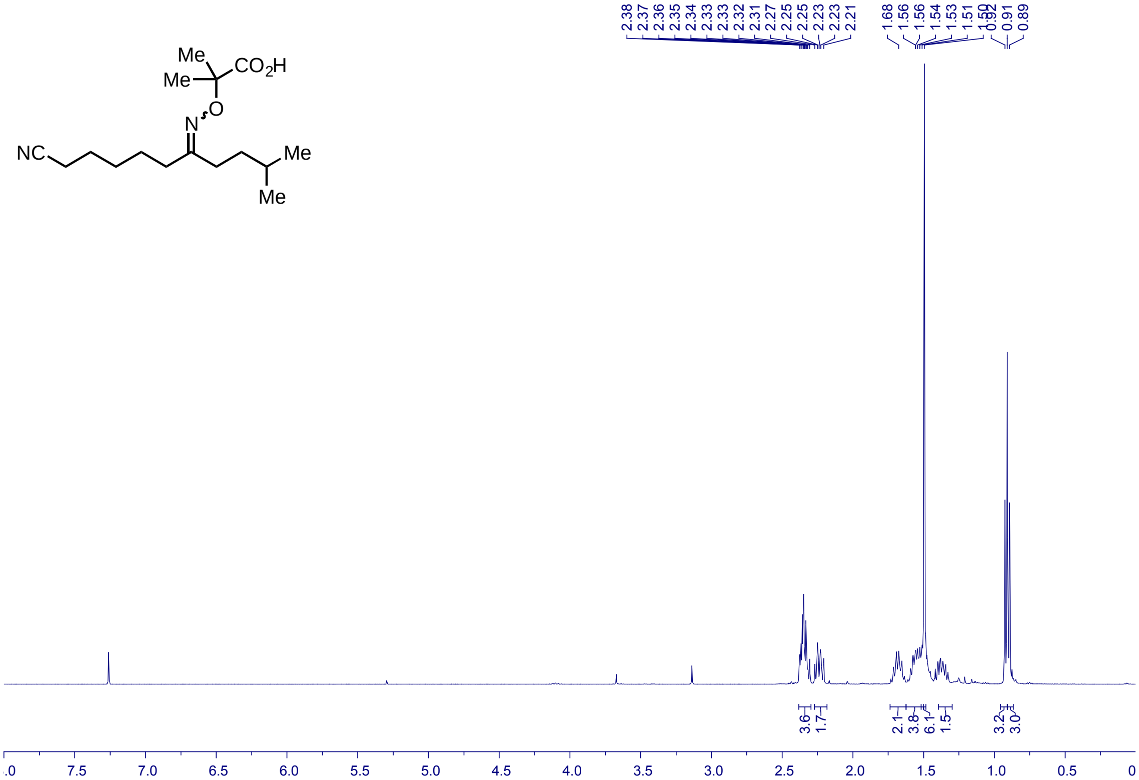

6n ^{13}C NMR (126 MHz, $CDCl_3$)

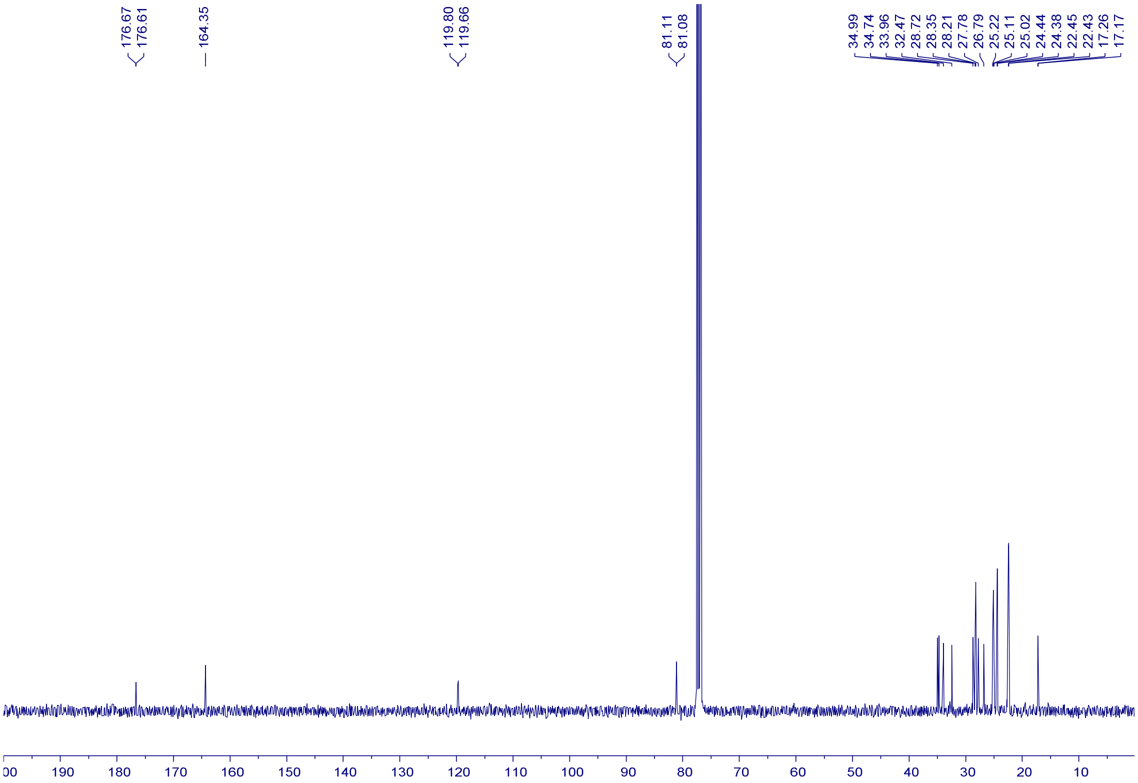

6o ^{1}H NMR (400 MHz, $CDCl_3$)

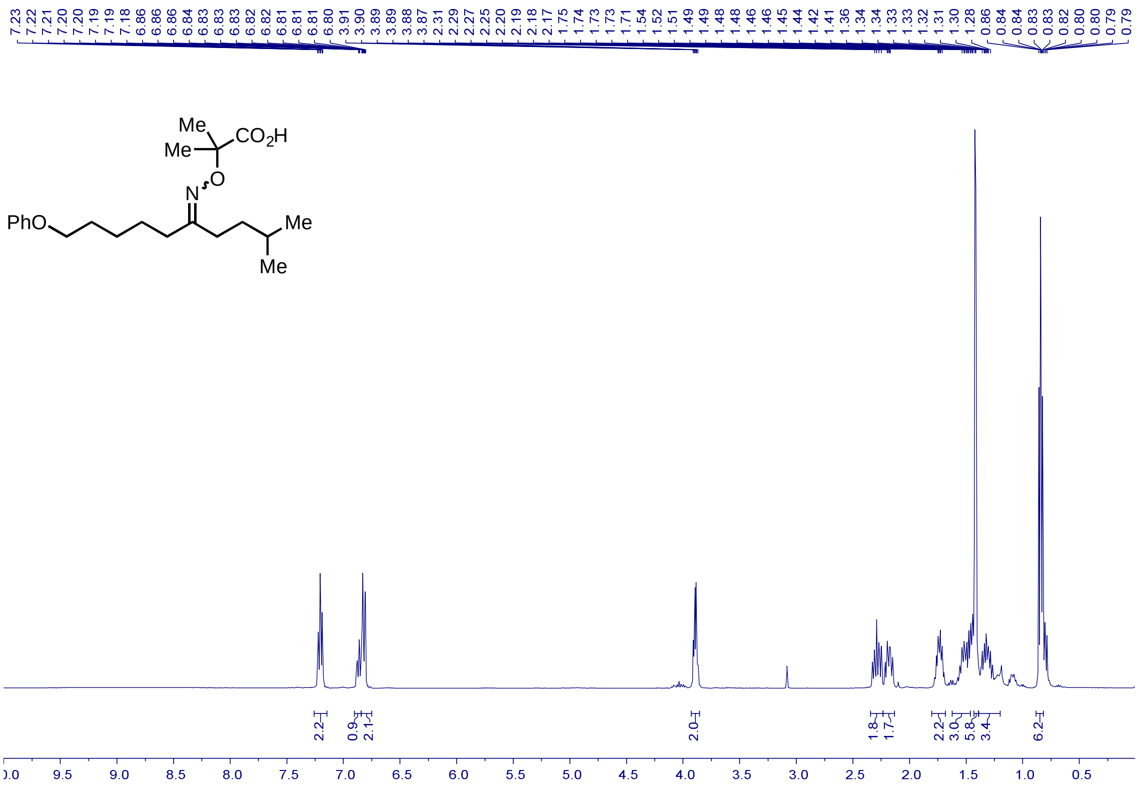

6o ^{13}C NMR (126 MHz, $CDCl_3$)

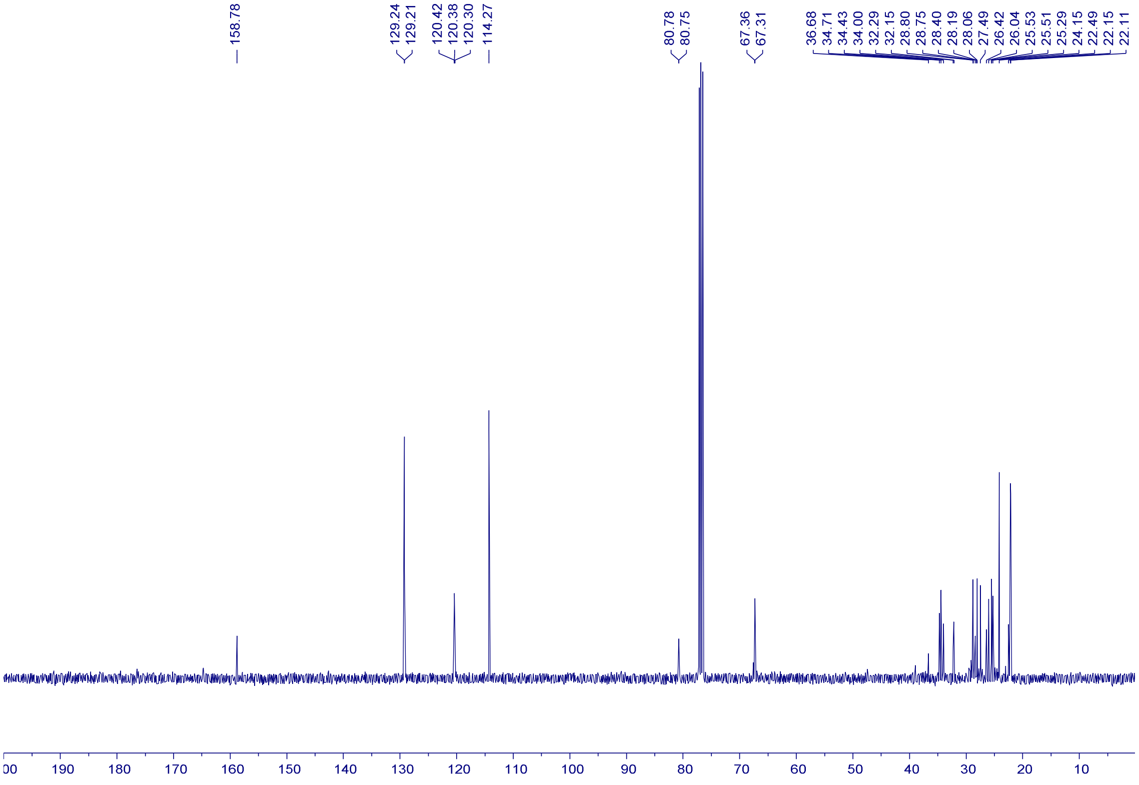

6p ^{1}H NMR (400 MHz, $CDCl_3$)

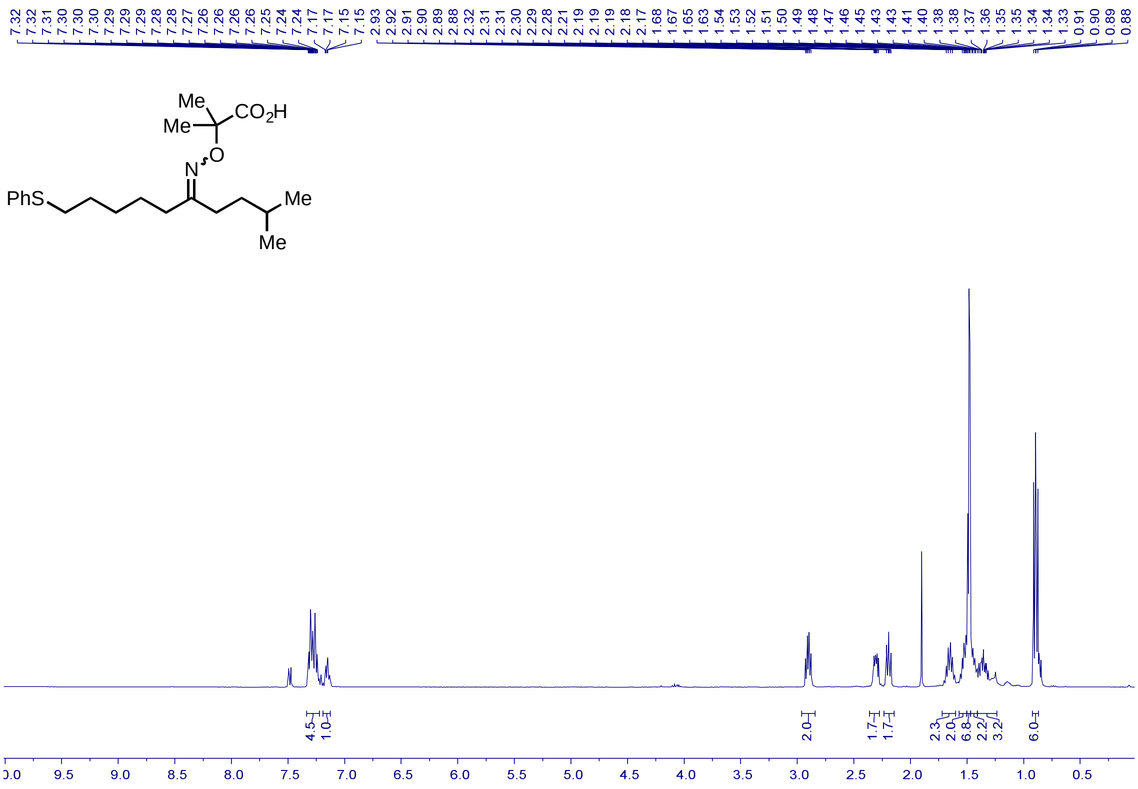

6p ^{13}C NMR (126 MHz, $CDCl_3$)

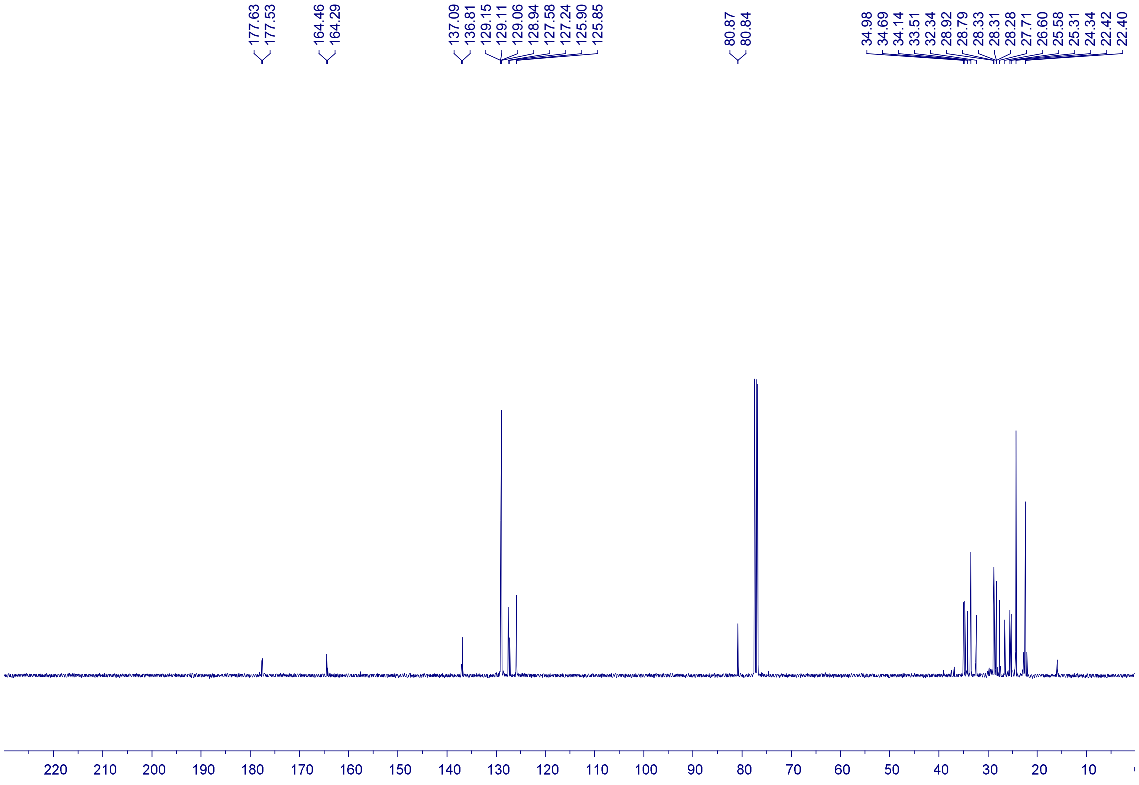

6q ^{1}H NMR (400 MHz, $CDCl_3$)

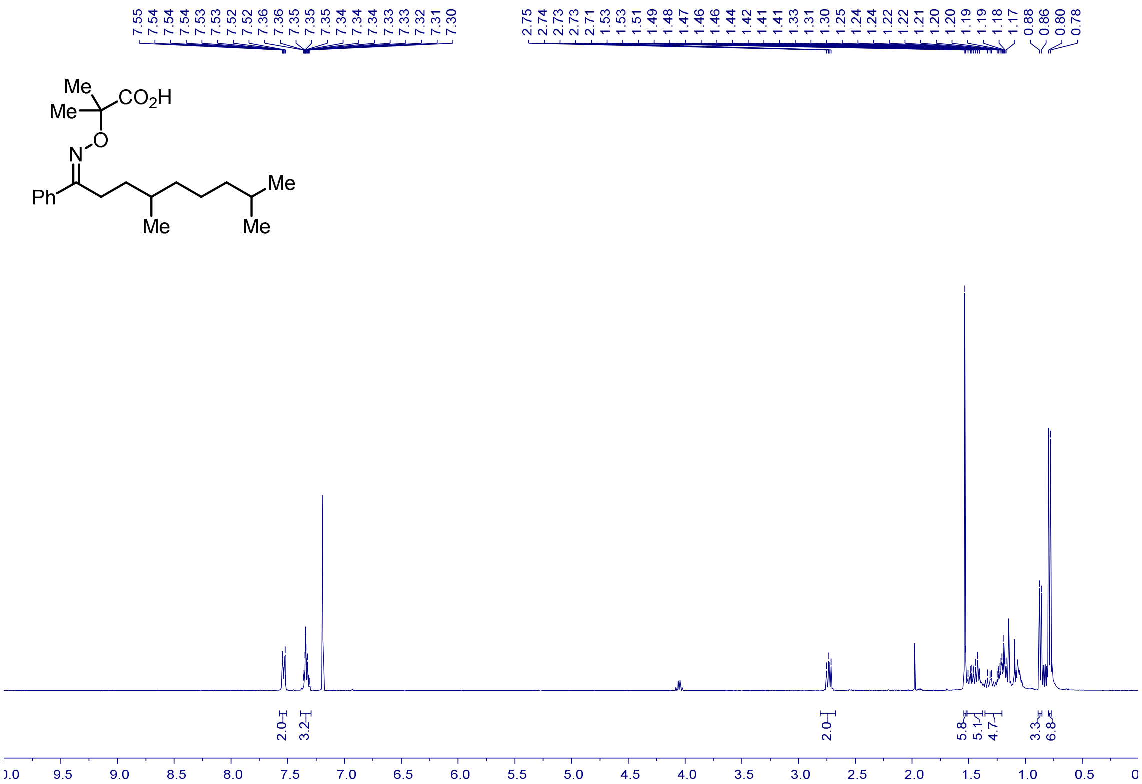

6q ^{13}C NMR (126 MHz, $CDCl_3$)

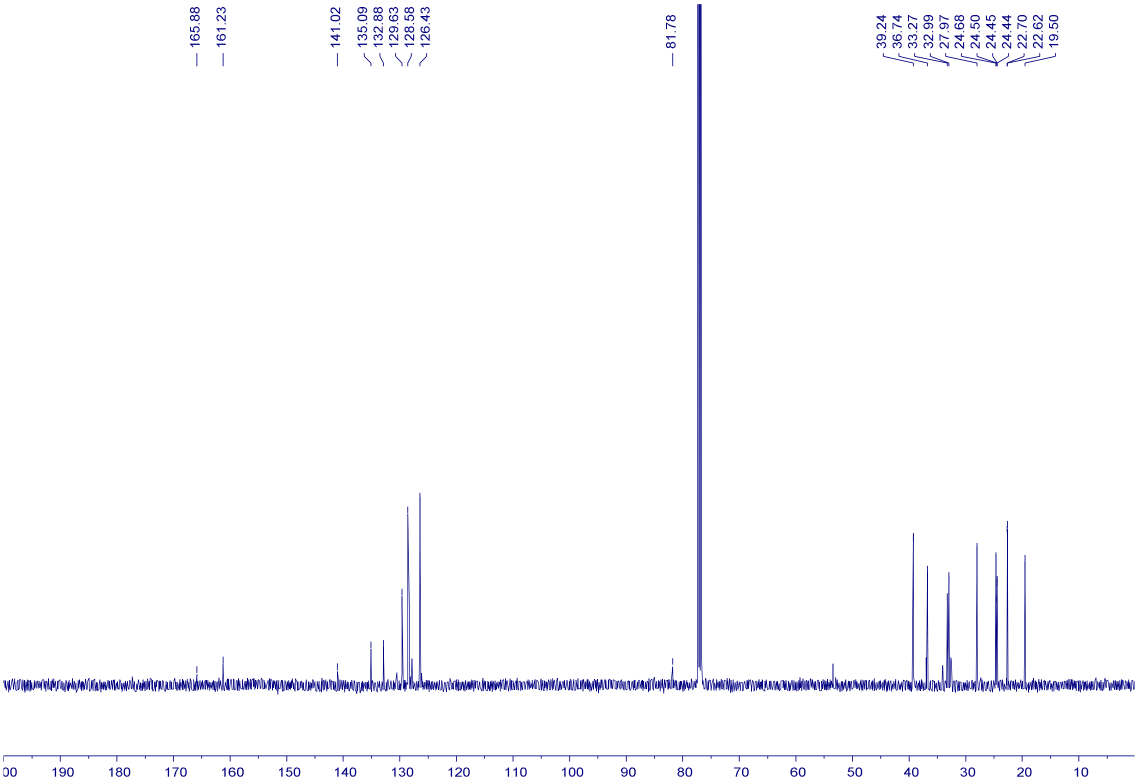

6r ^{1}H NMR (400 MHz, $CDCl_3$)

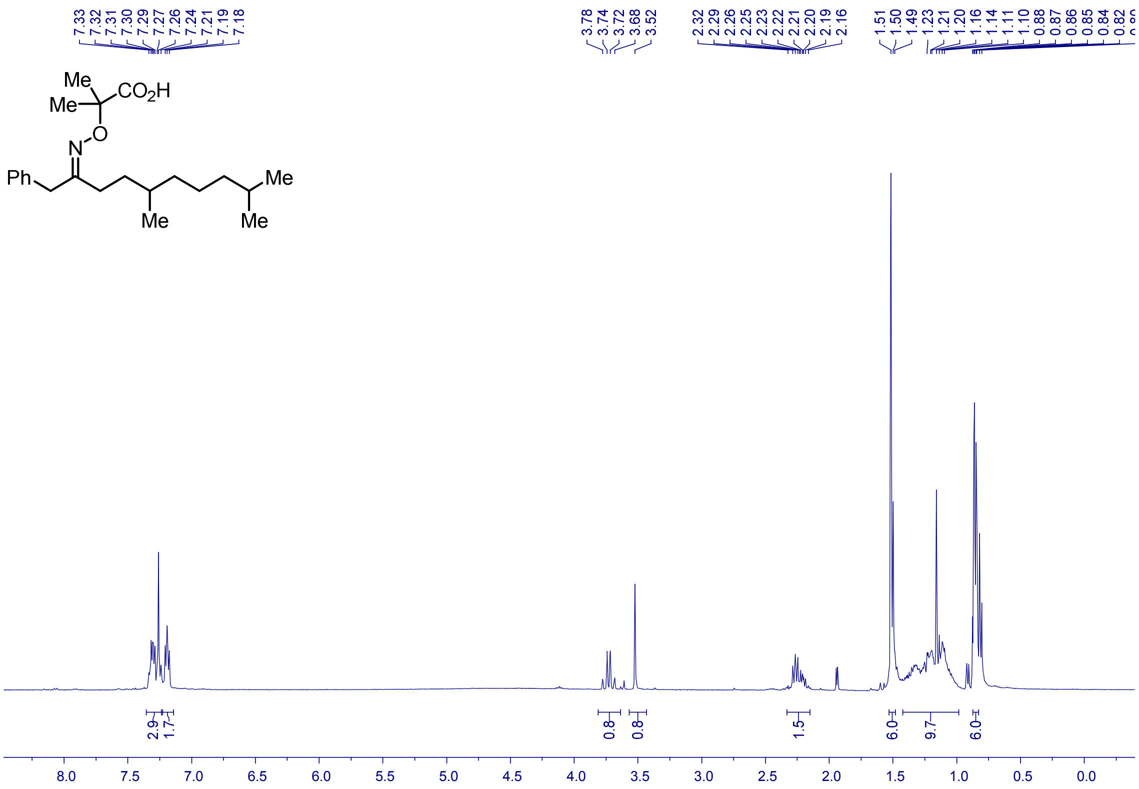

6r ^{13}C NMR (126 MHz, $CDCl_3$)

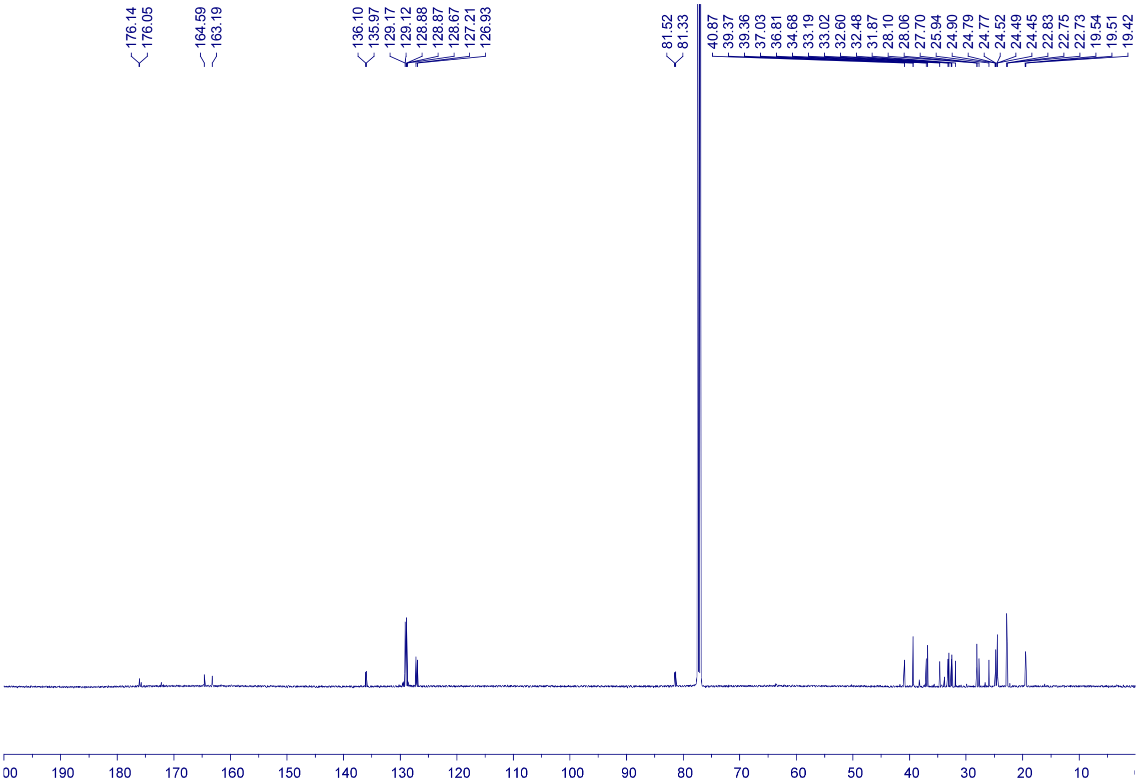

6s ^{1}H NMR (400 MHz, $CDCl_3$)

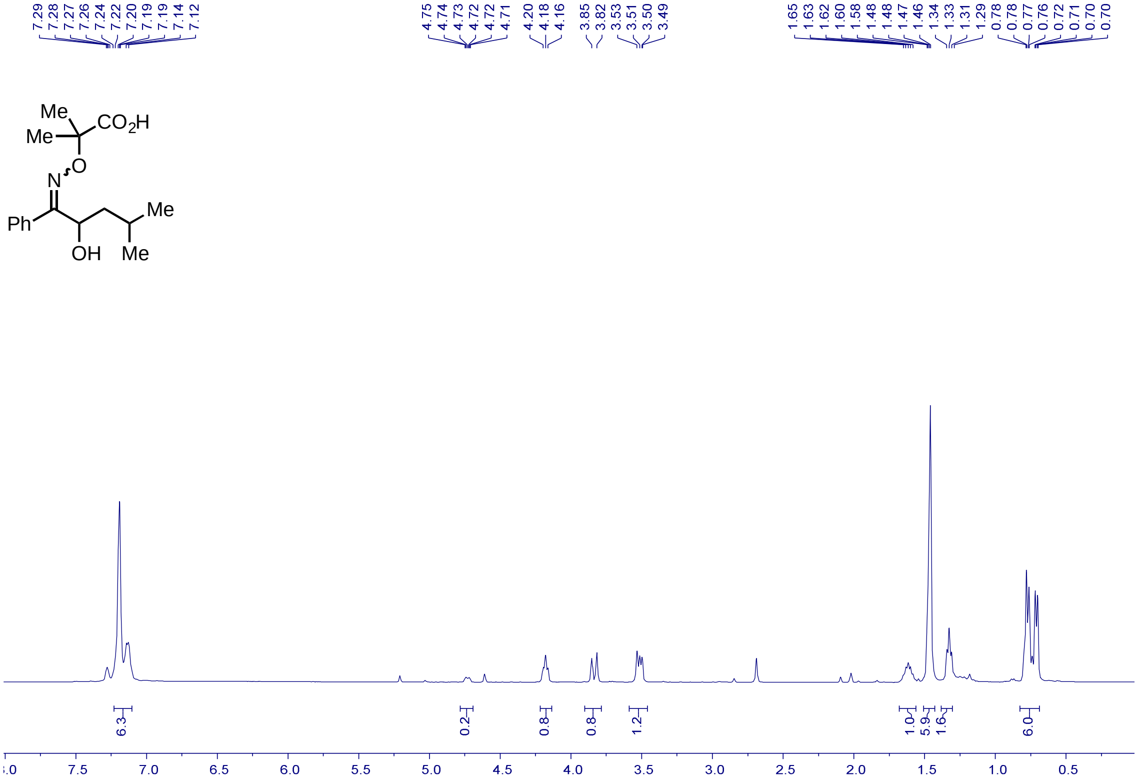

6s ^{13}C NMR (126 MHz, $CDCl_3$)

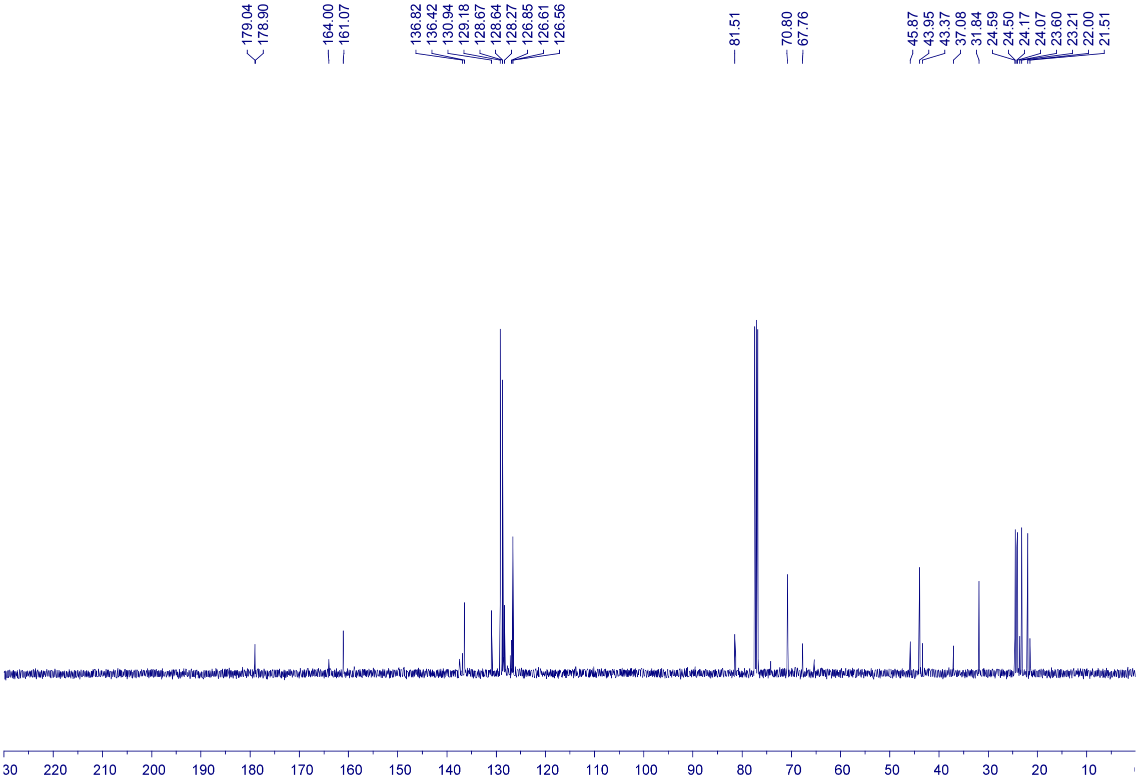

6t ^{1}H NMR (400 MHz, $CDCl_3$)

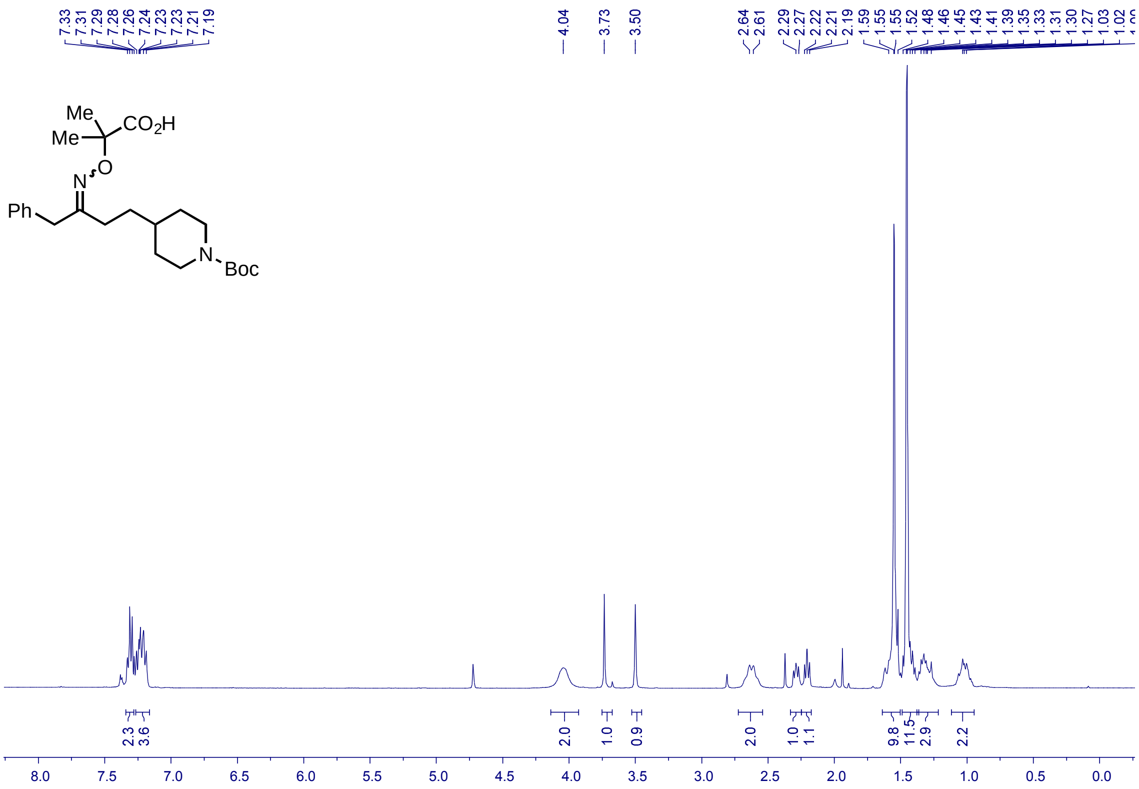

6t ^{13}C NMR (126 MHz, $CDCl_3$)

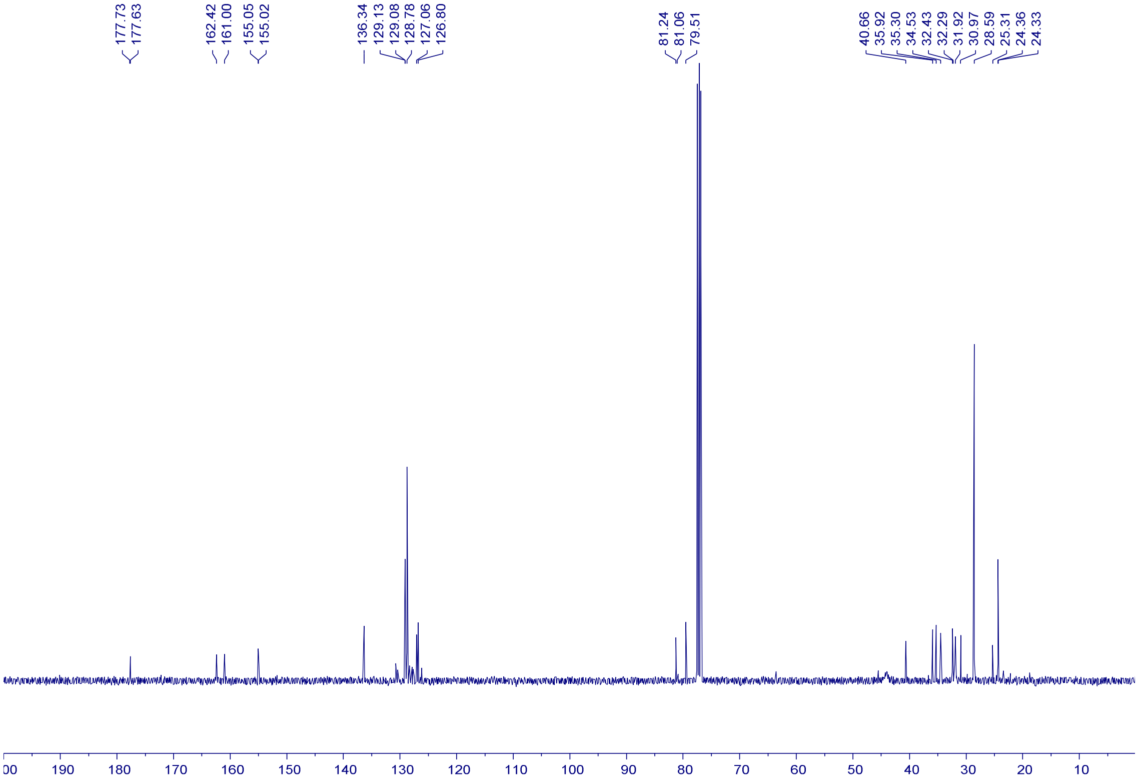

6u ^{1}H NMR (400 MHz, $CDCl_3$)

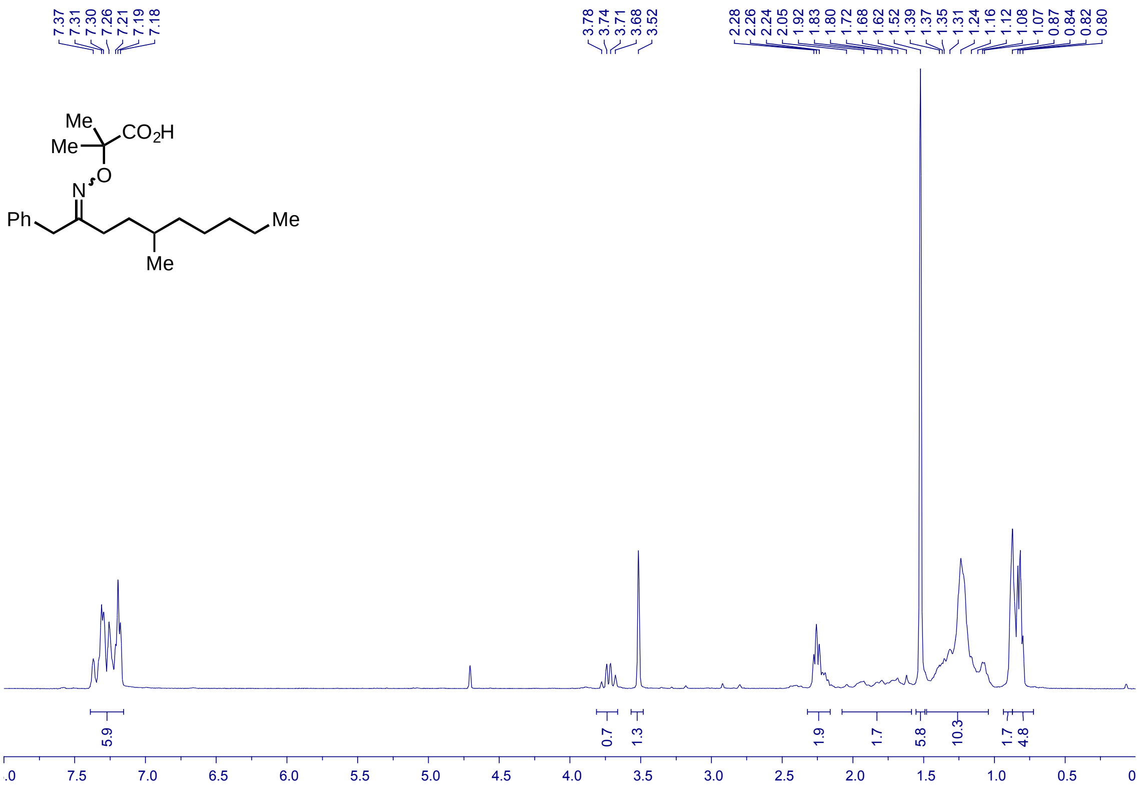

6u ^{13}C NMR (126 MHz, $CDCl_3$)

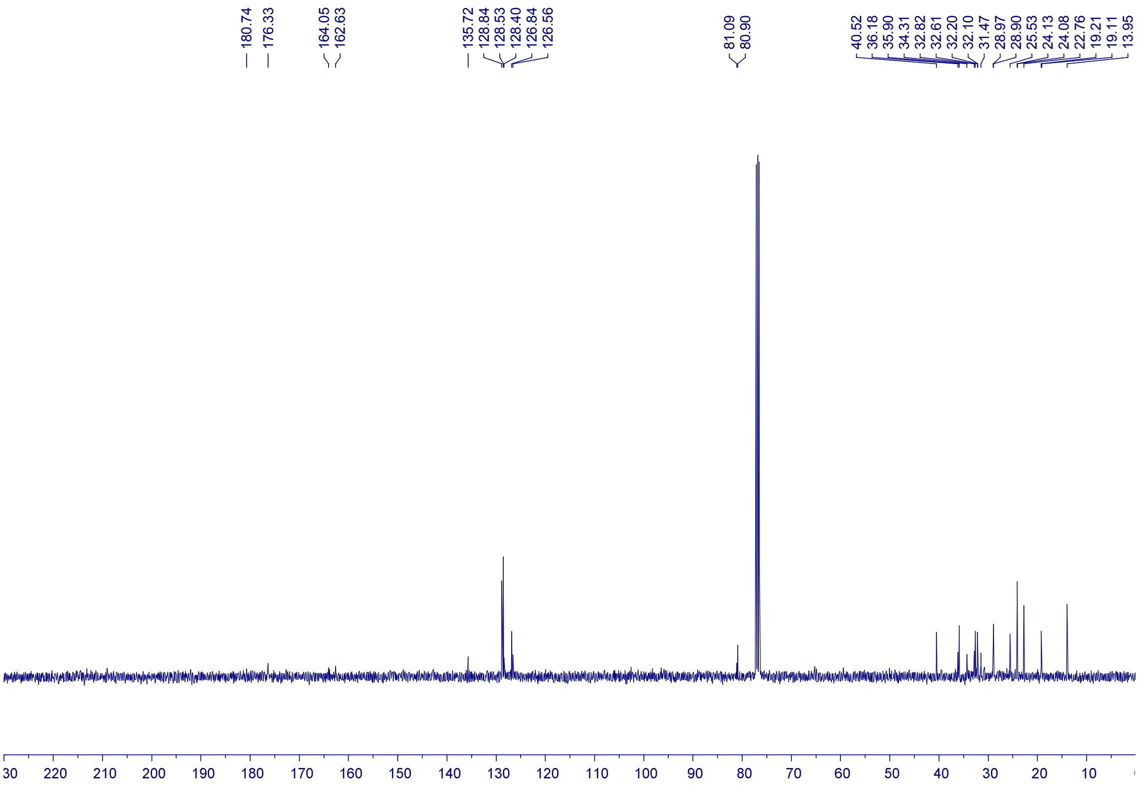

6v ^{1}H NMR (400 MHz, $CDCl_3$)

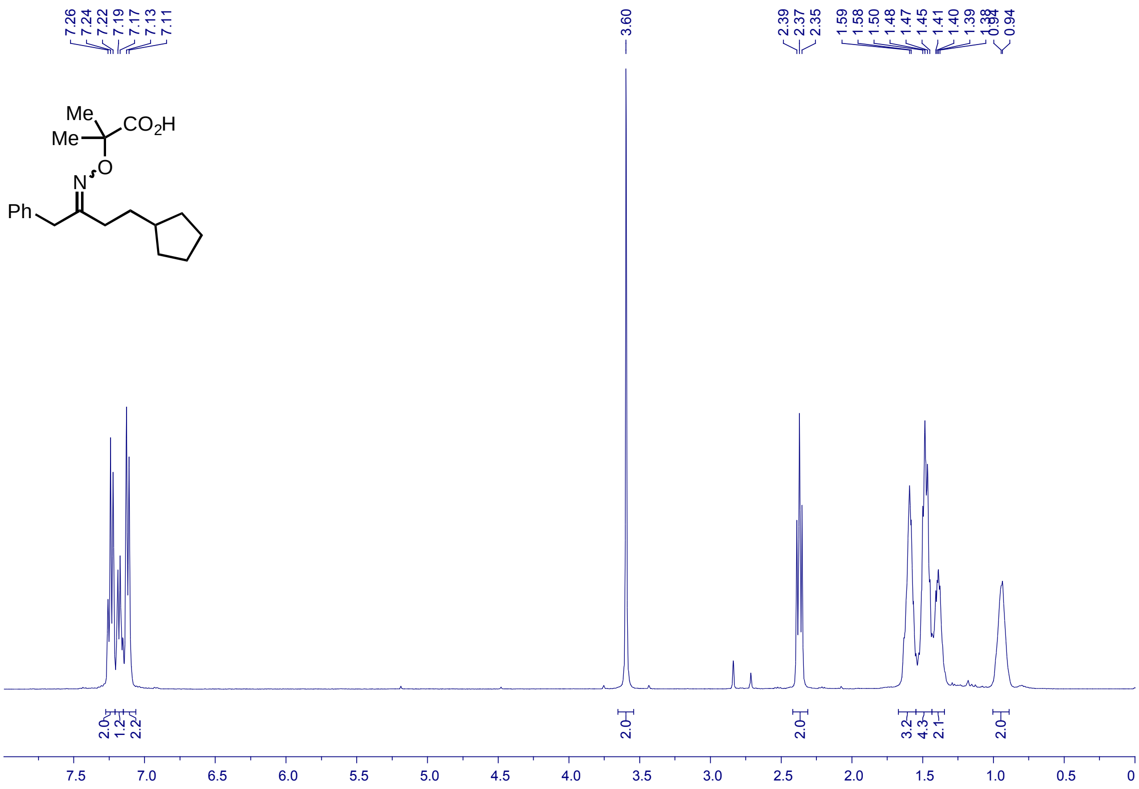

6v ^{13}C NMR (126 MHz, $CDCl_3$)

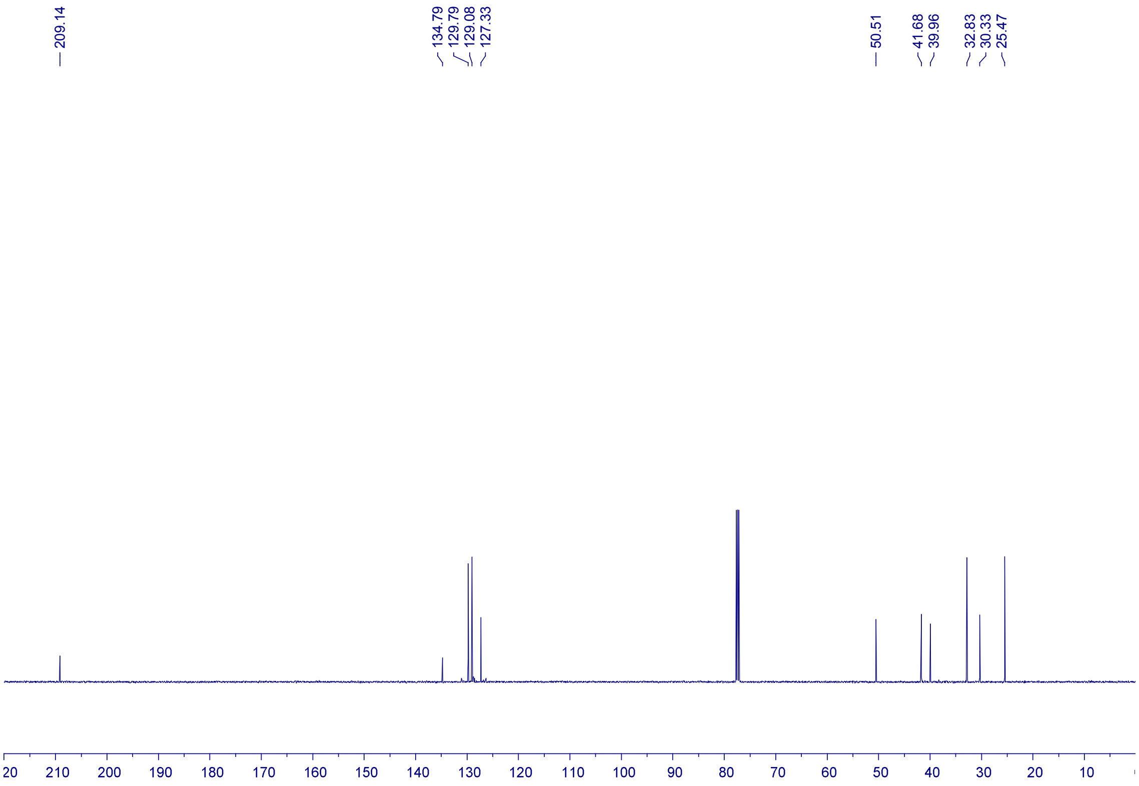

6w ^{1}H NMR (400 MHz, $CDCl_3$)

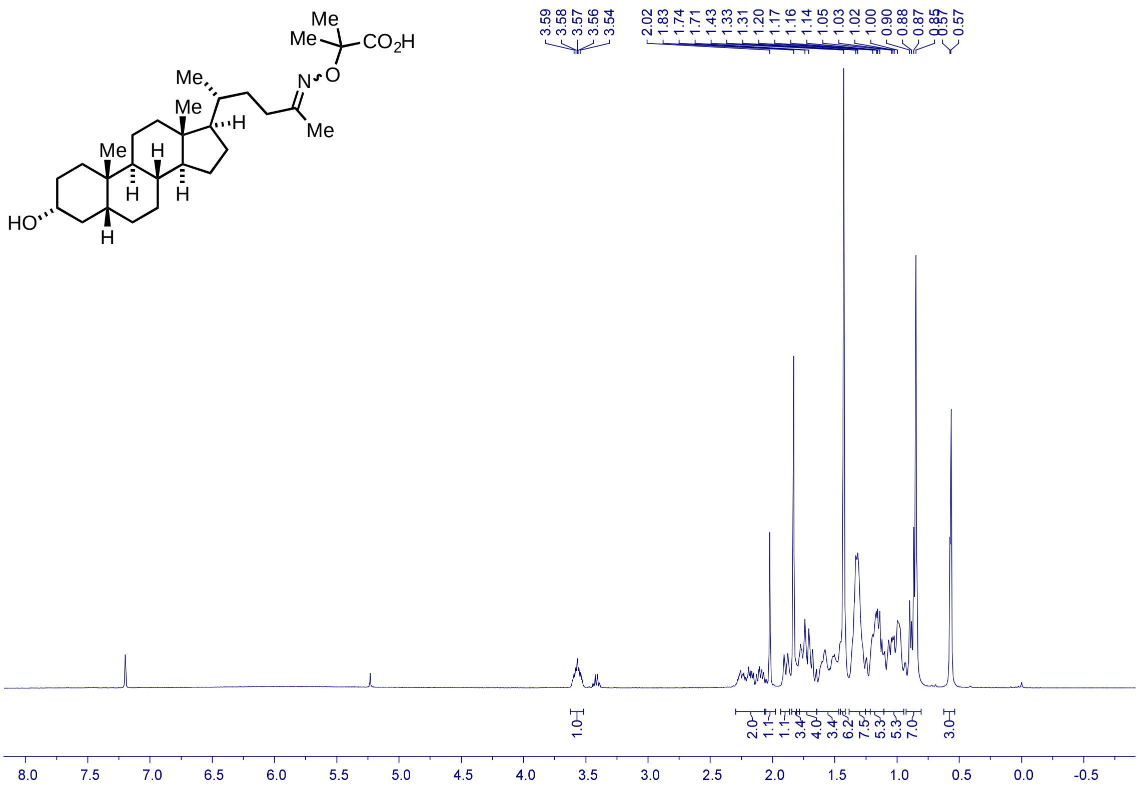

6w ^{13}C NMR (126 MHz, $CDCl_3$)

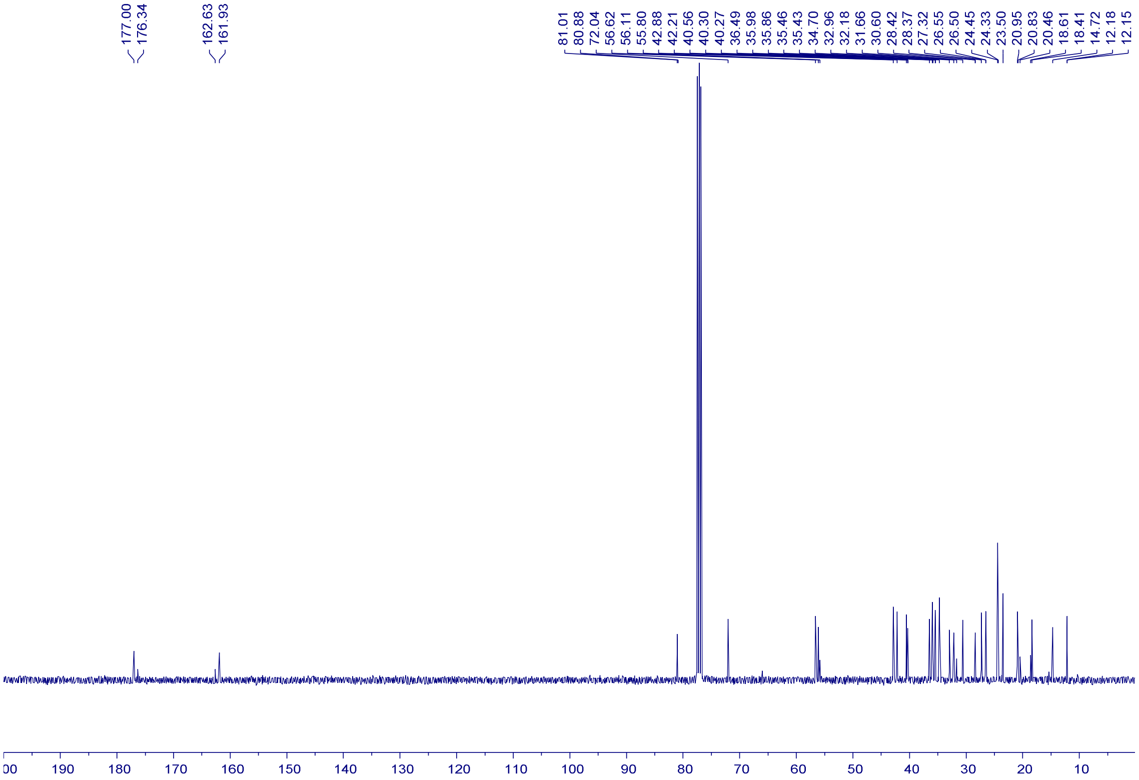

3b ^{1}H NMR (500 MHz, $CDCl_3$)[d]

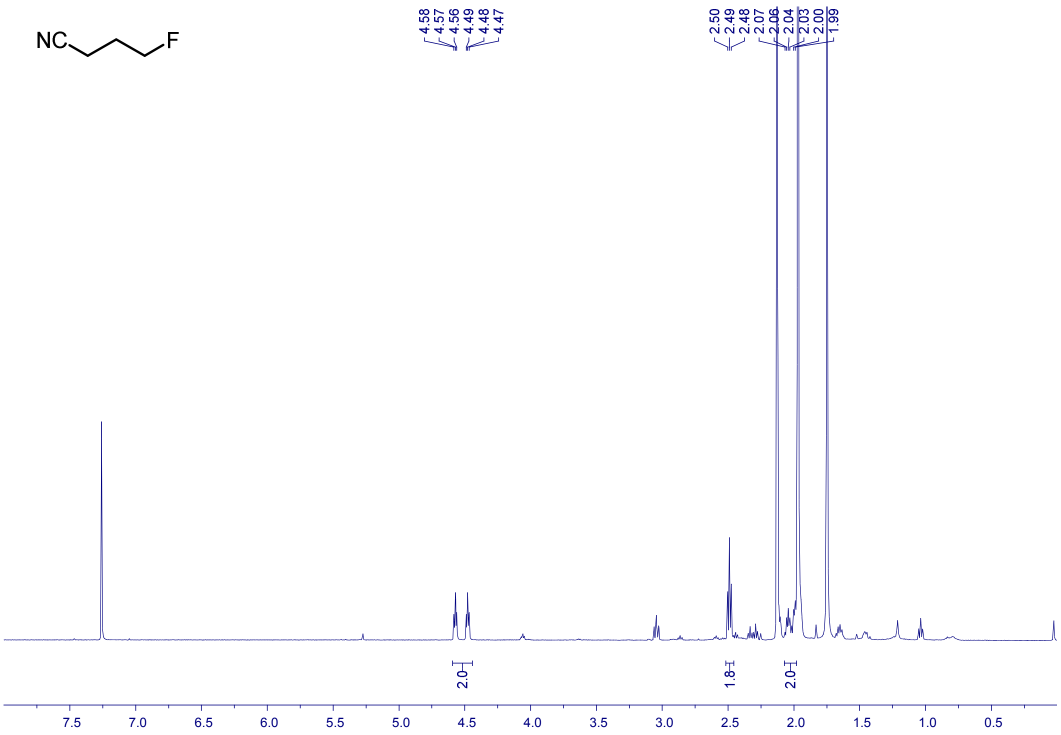

3b ^{13}C NMR (126 MHz, $CDCl_3$)

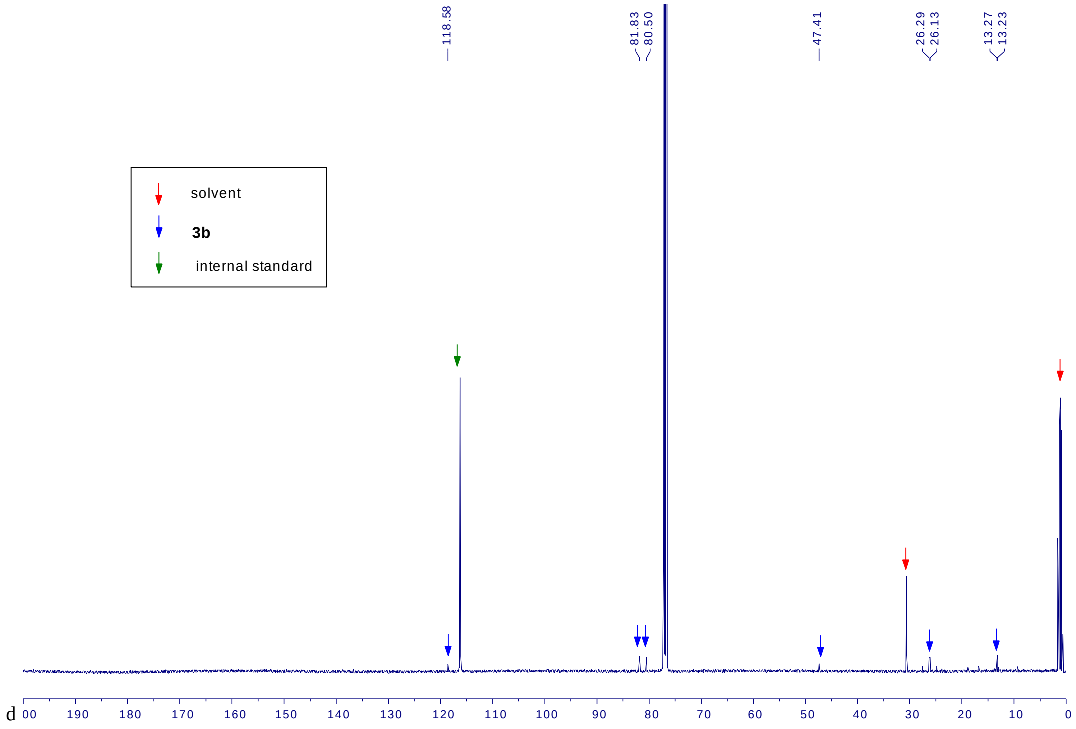

3b ^{19}F NMR (376 MHz, $CDCl_3$) [d]

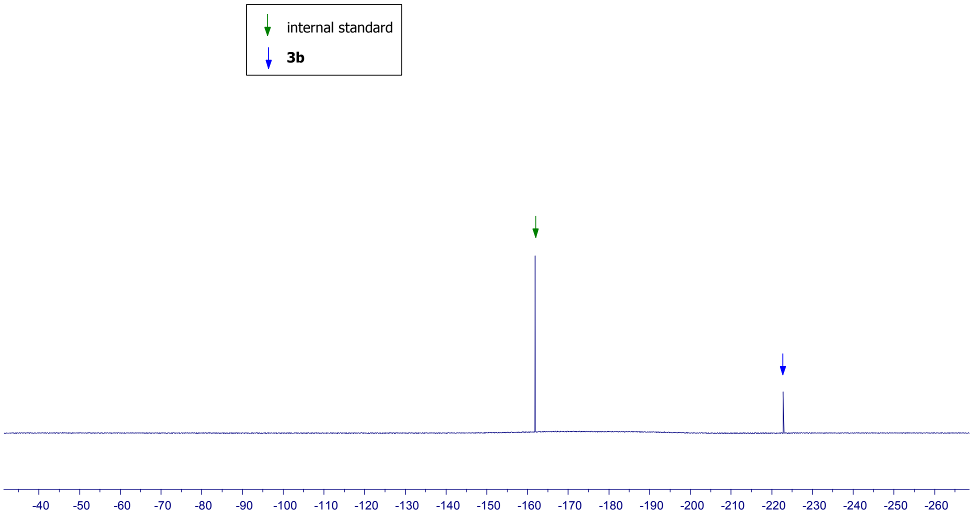

3e ^{1}H NMR (400 MHz, $CDCl_3$)

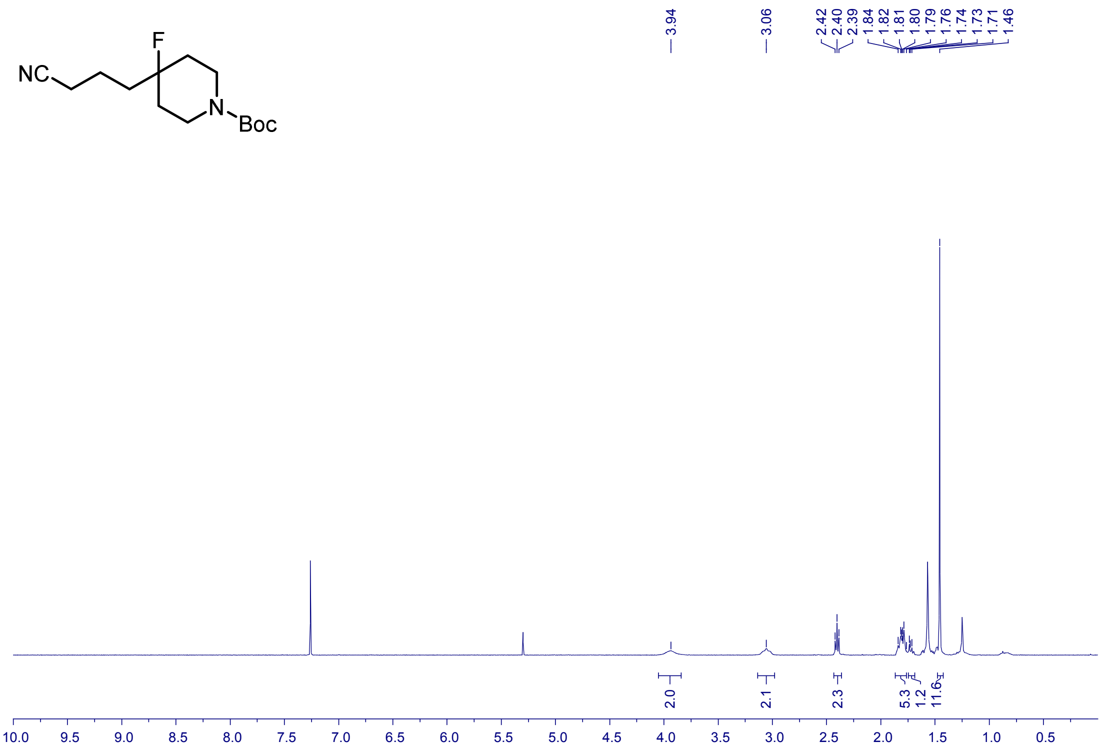

3e ^{13}C NMR (126 MHz, $CDCl_3$)

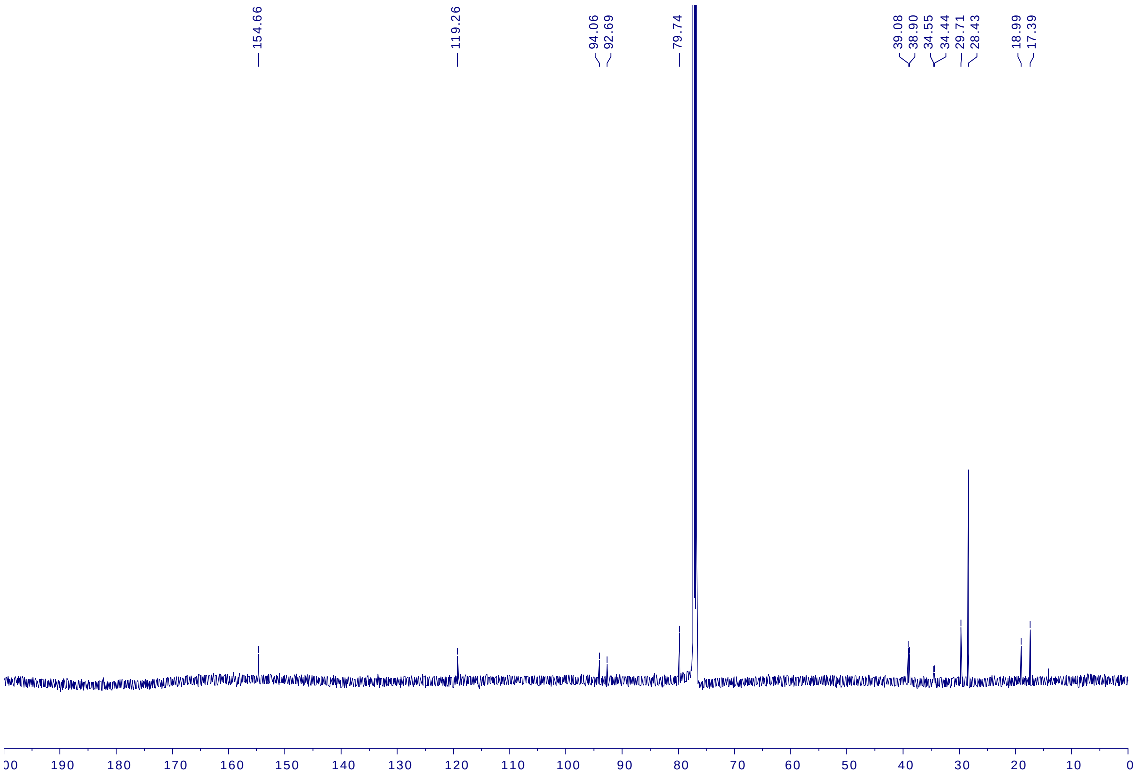

3e ^{19}F NMR (376 MHz, $CDCl_3$)

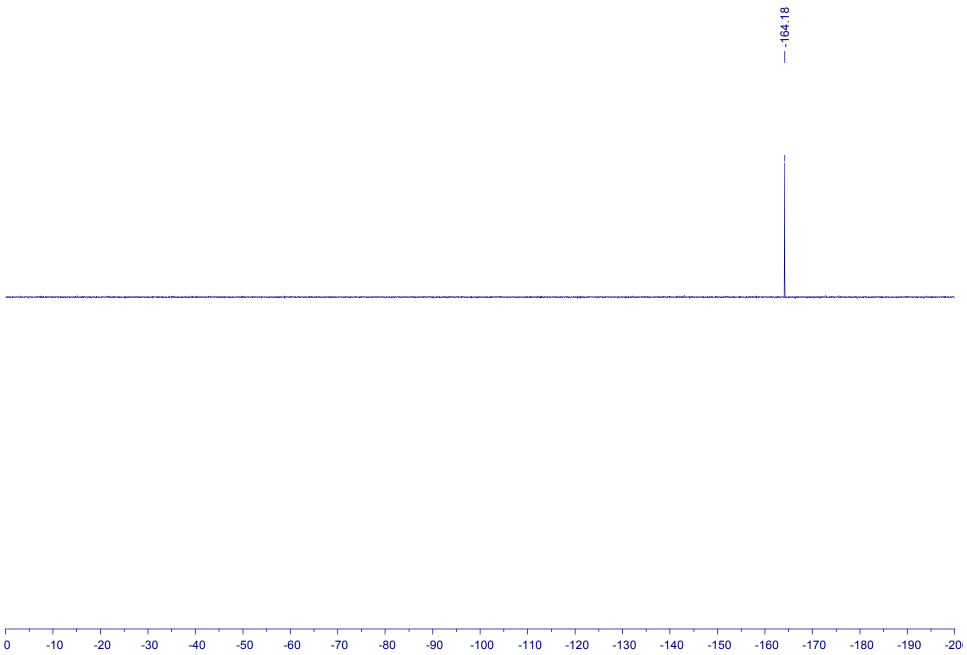

3f ^{1}H NMR (400 MHz, $CDCl_3$)

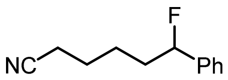

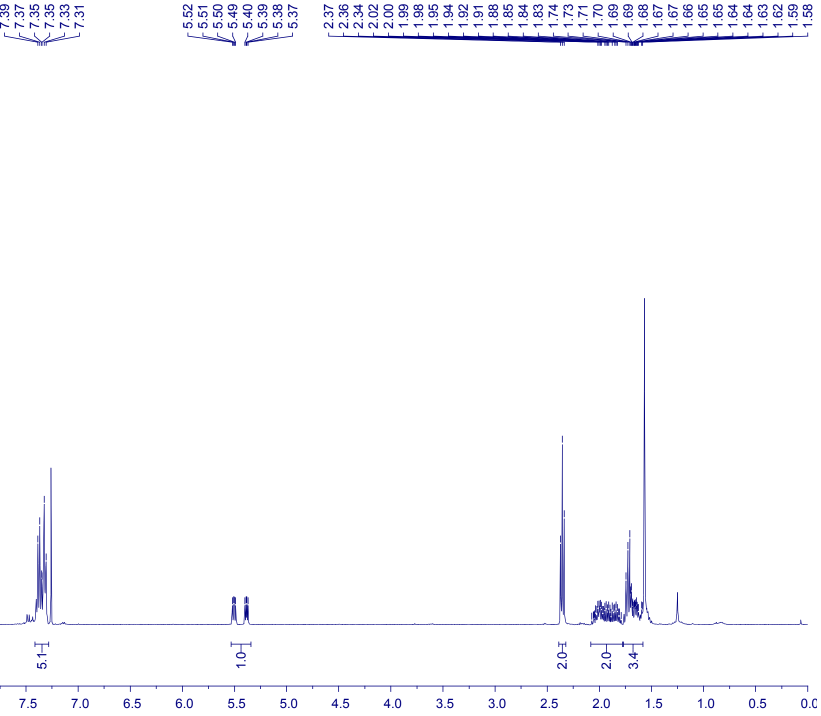

3f ^{13}C NMR (101 MHz, $CDCl_3$)

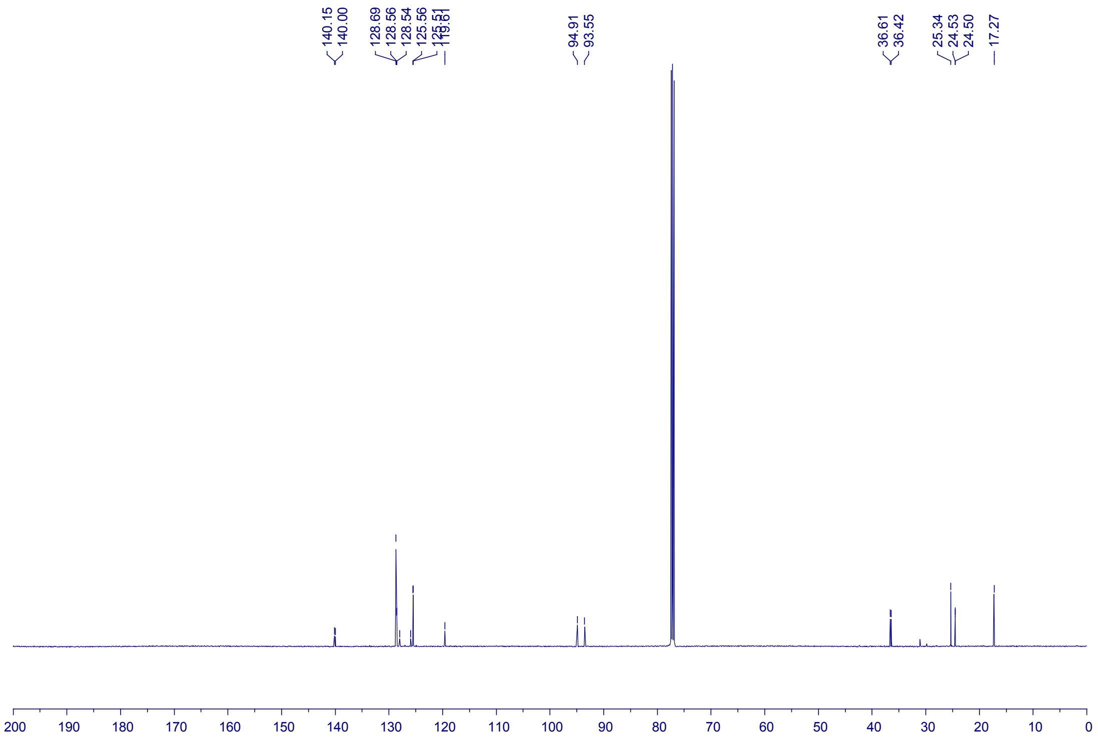

3f ^{19}F NMR (376 MHz, $CDCl_3$)

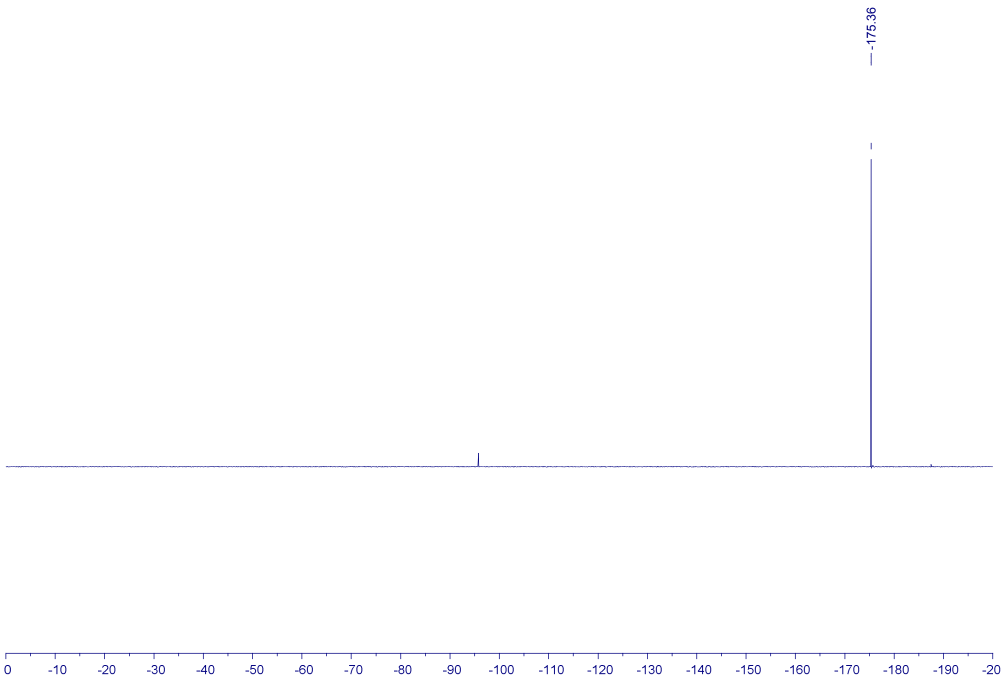

3g ^{1}H NMR (400 MHz, $CDCl_3$)

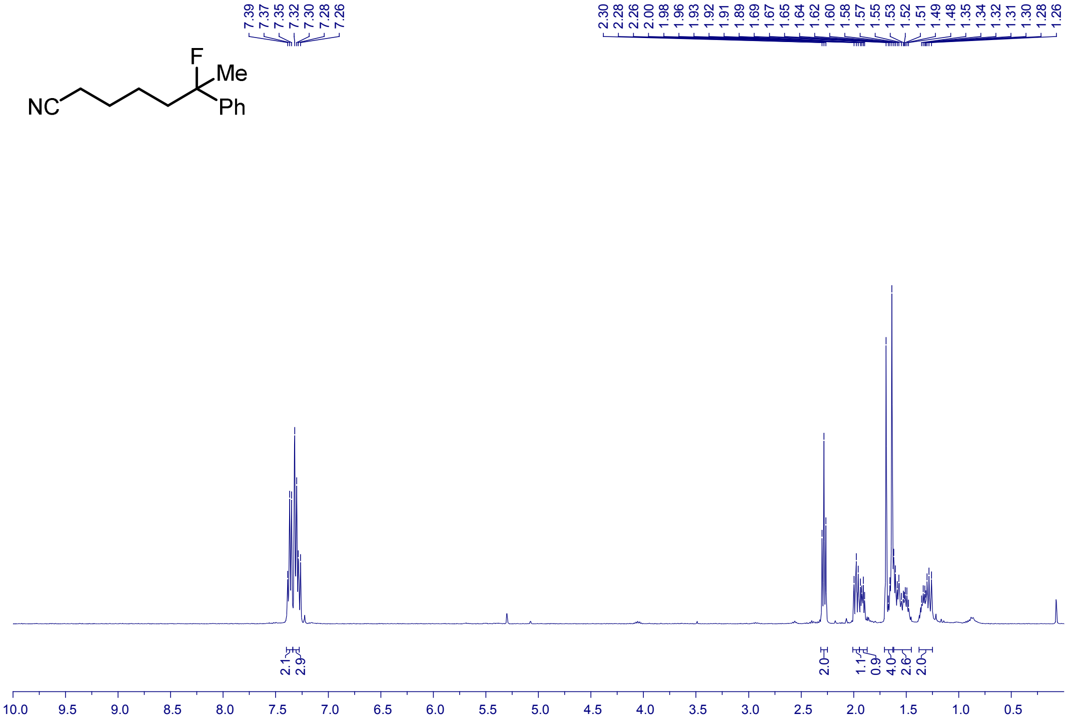

3g ^{13}C NMR (101 MHz, $CDCl_3$)

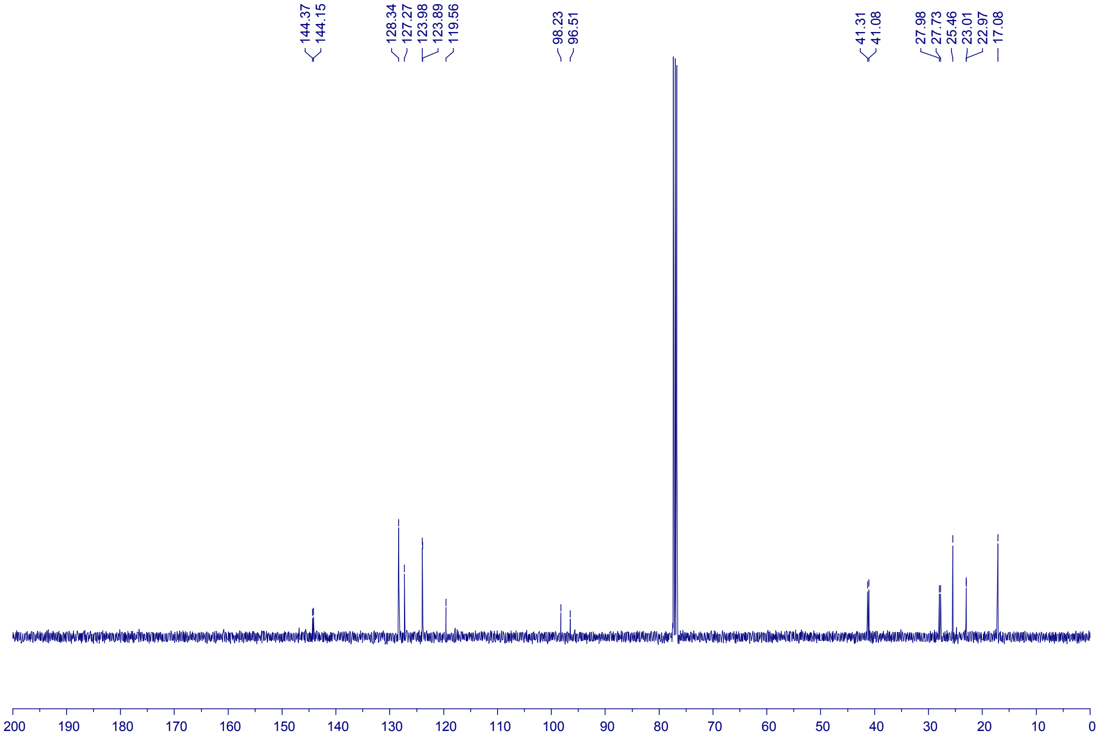

3g ^{19}F NMR (376 MHz, $CDCl_3$)

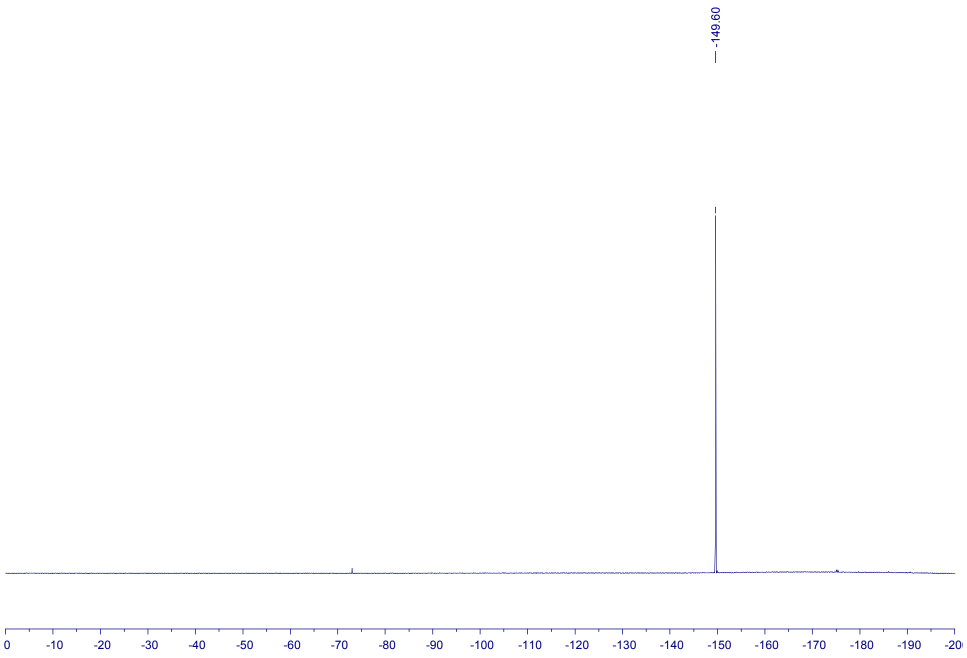

3h ^{1}H NMR (400 MHz, $CDCl_3$)

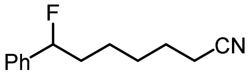

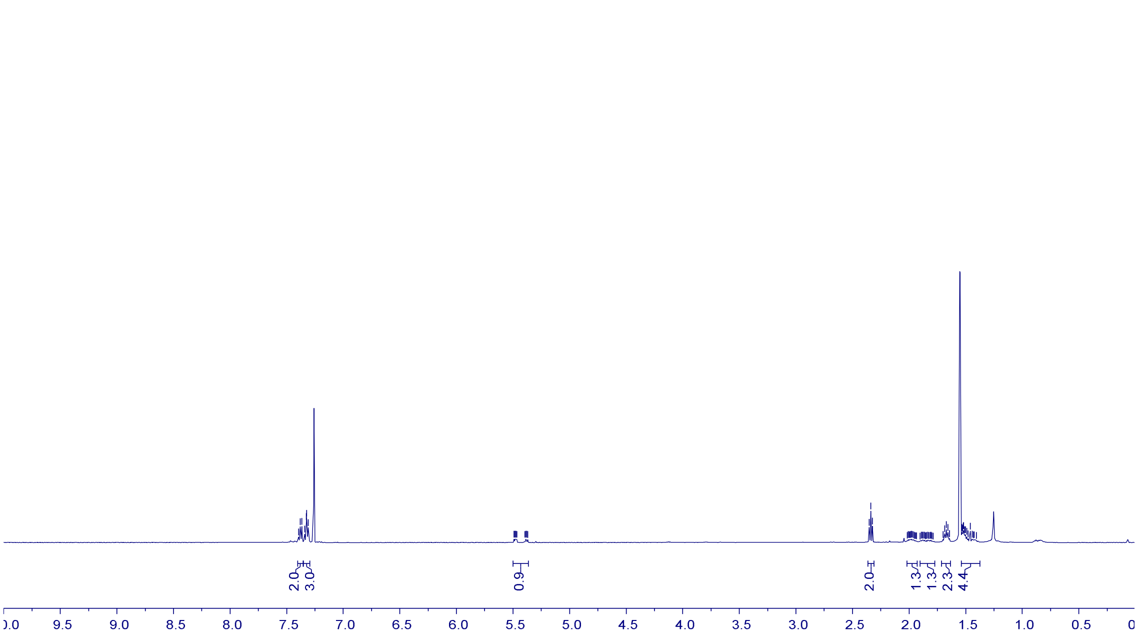

3h ^{13}C NMR (101 MHz, $CDCl_3$)

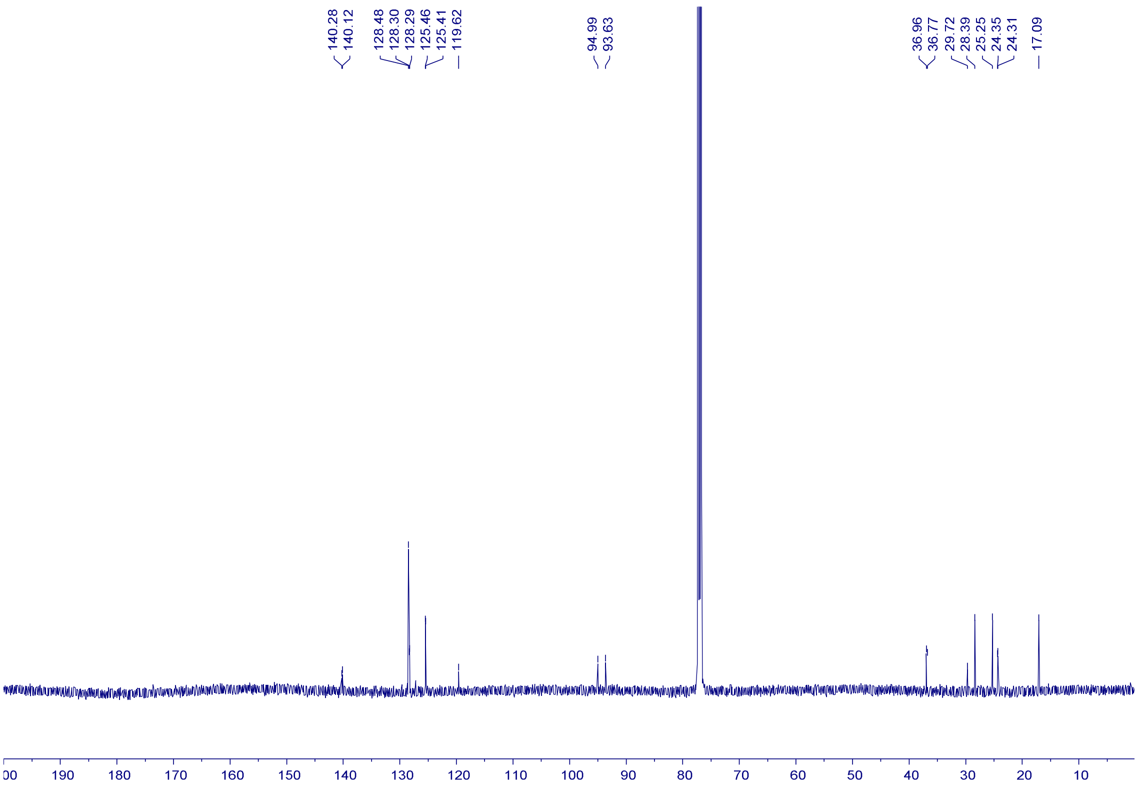

3h ^{19}F NMR (376 MHz, $CDCl_3$)

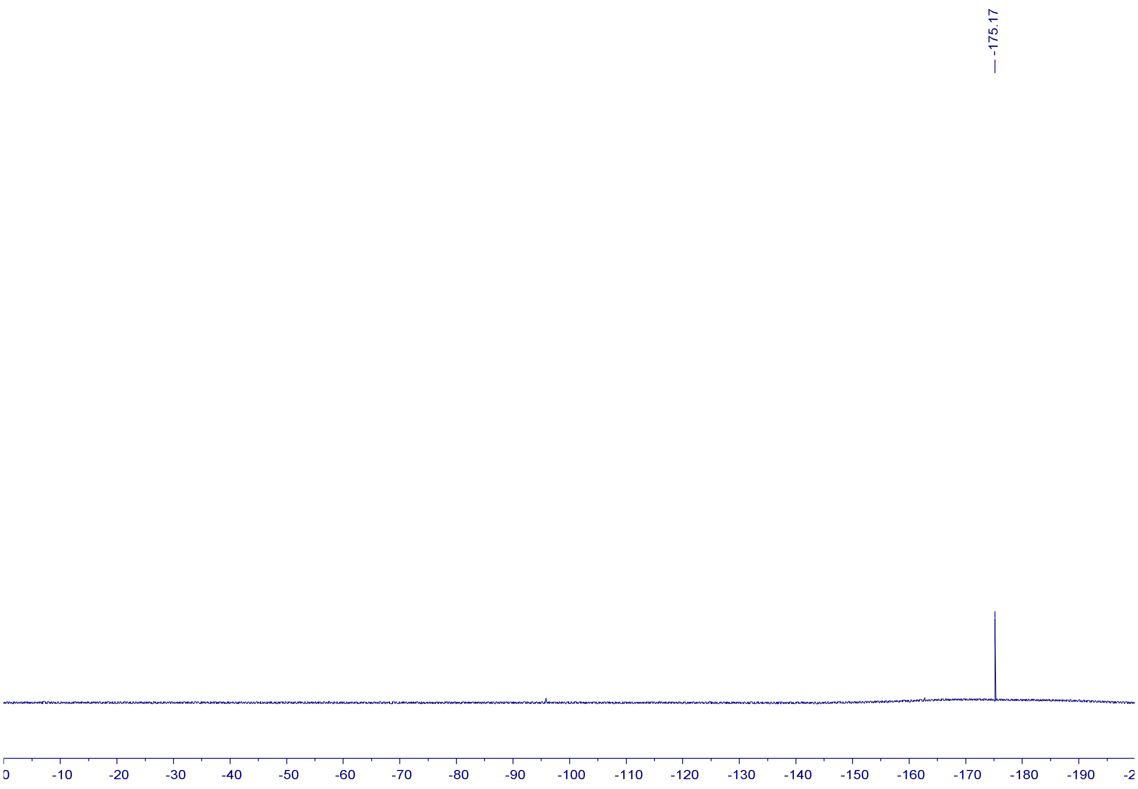

3i ^{1}H NMR (400 MHz, $CDCl_3$)

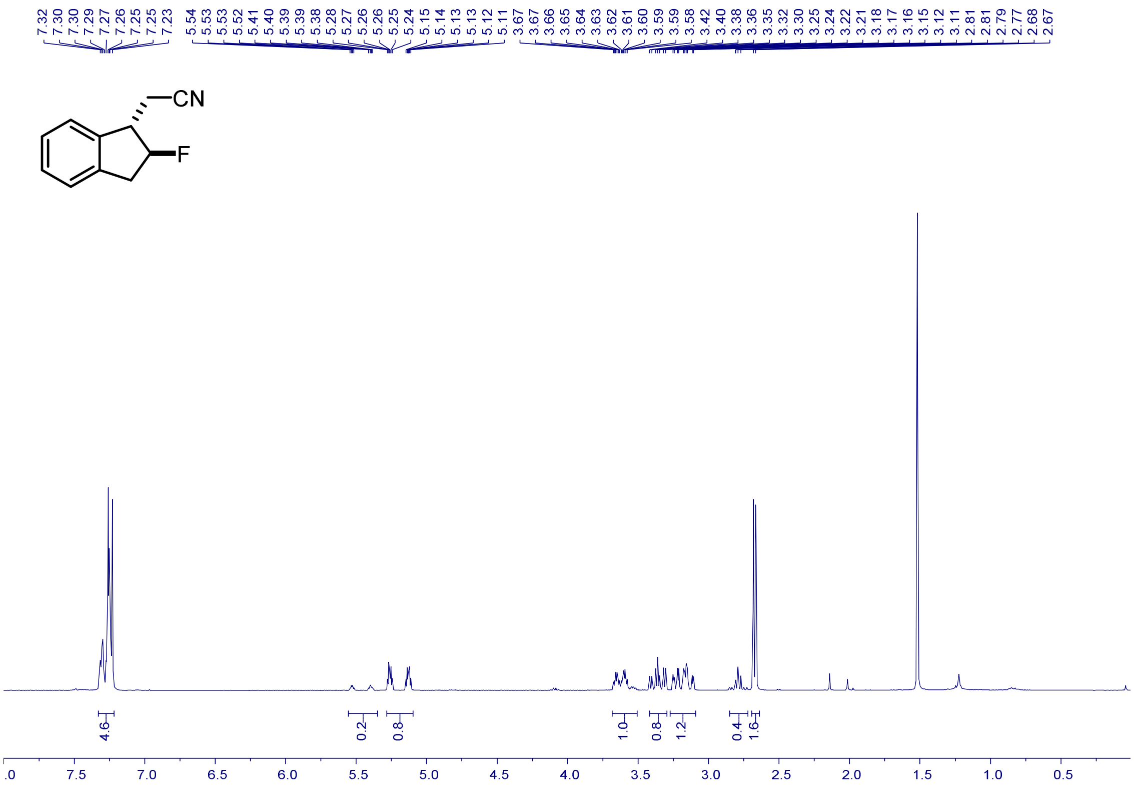

3i ^{13}C NMR (101 MHz, $CDCl_3$)

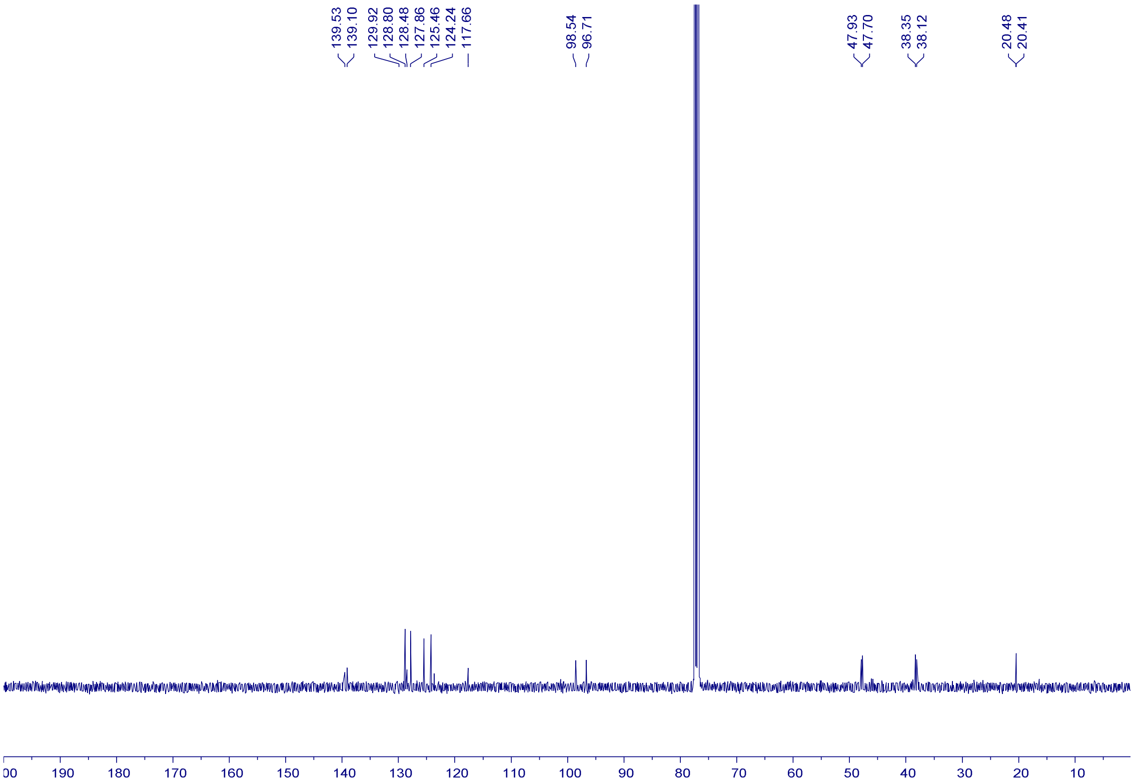

3i ^{19}F NMR (376 MHz, $CDCl_3$)

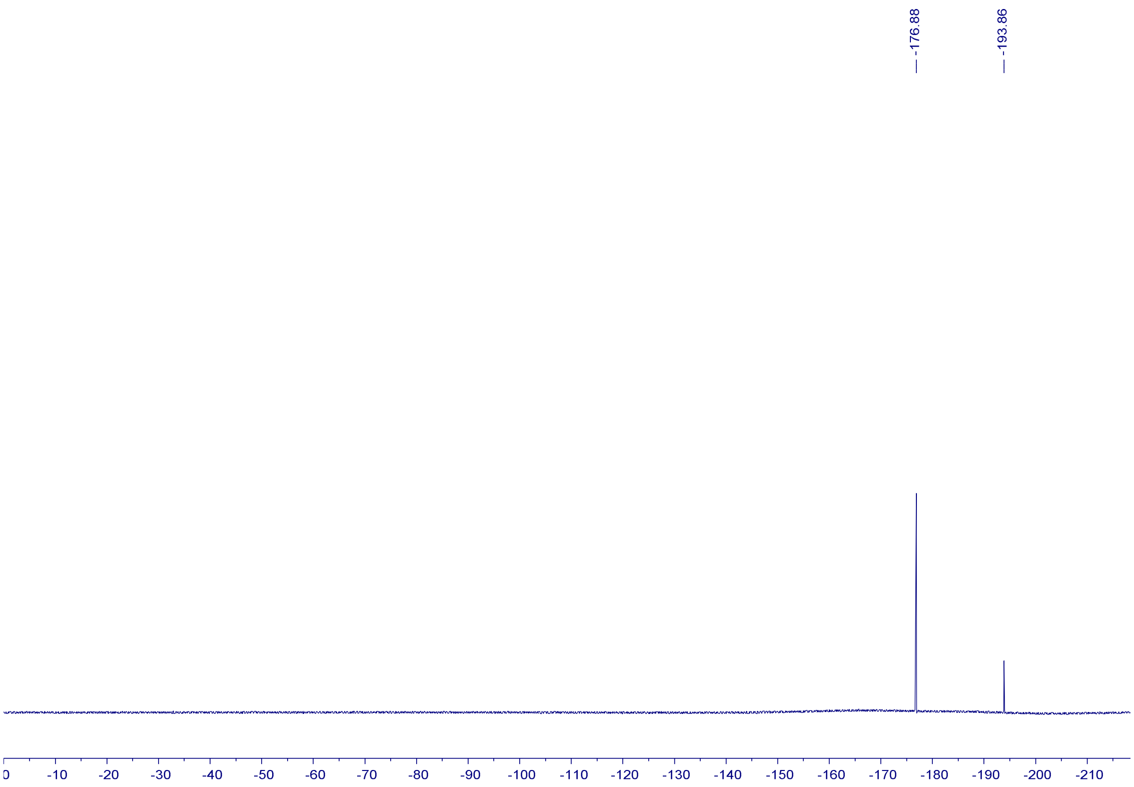

3j ^{1}H NMR (500 MHz, $CDCl_3$)

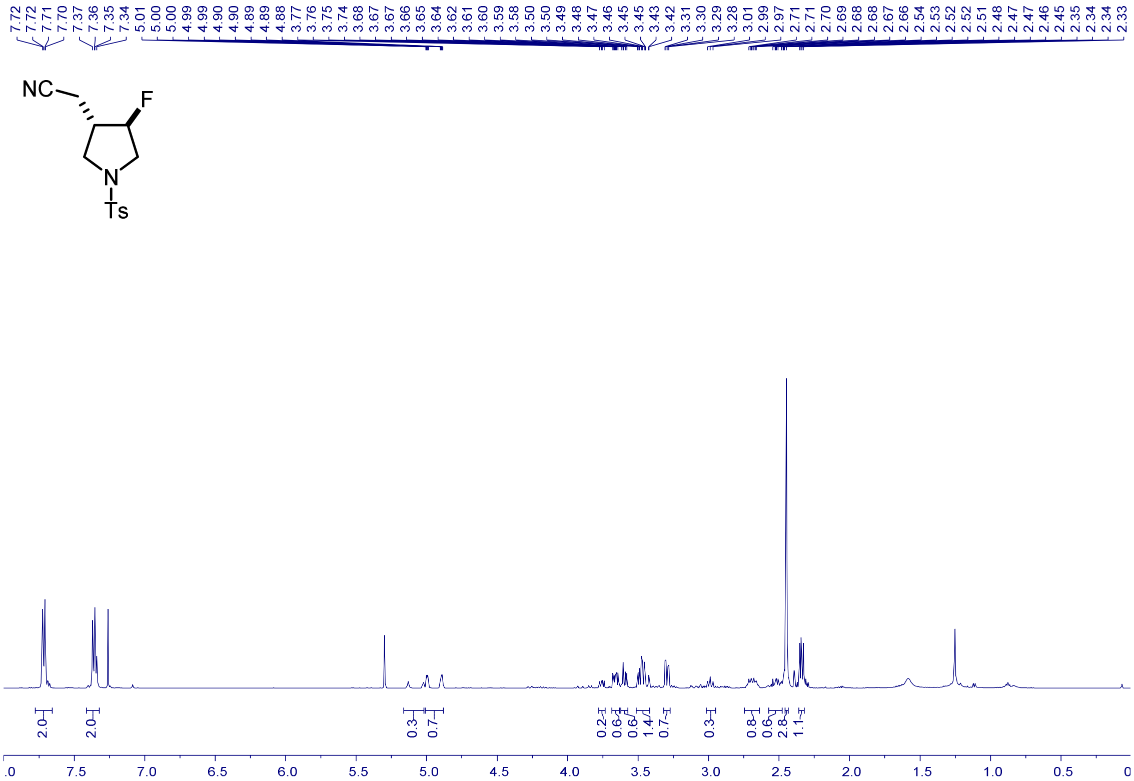

3j ^{13}C NMR (126 MHz, $CDCl_3$)

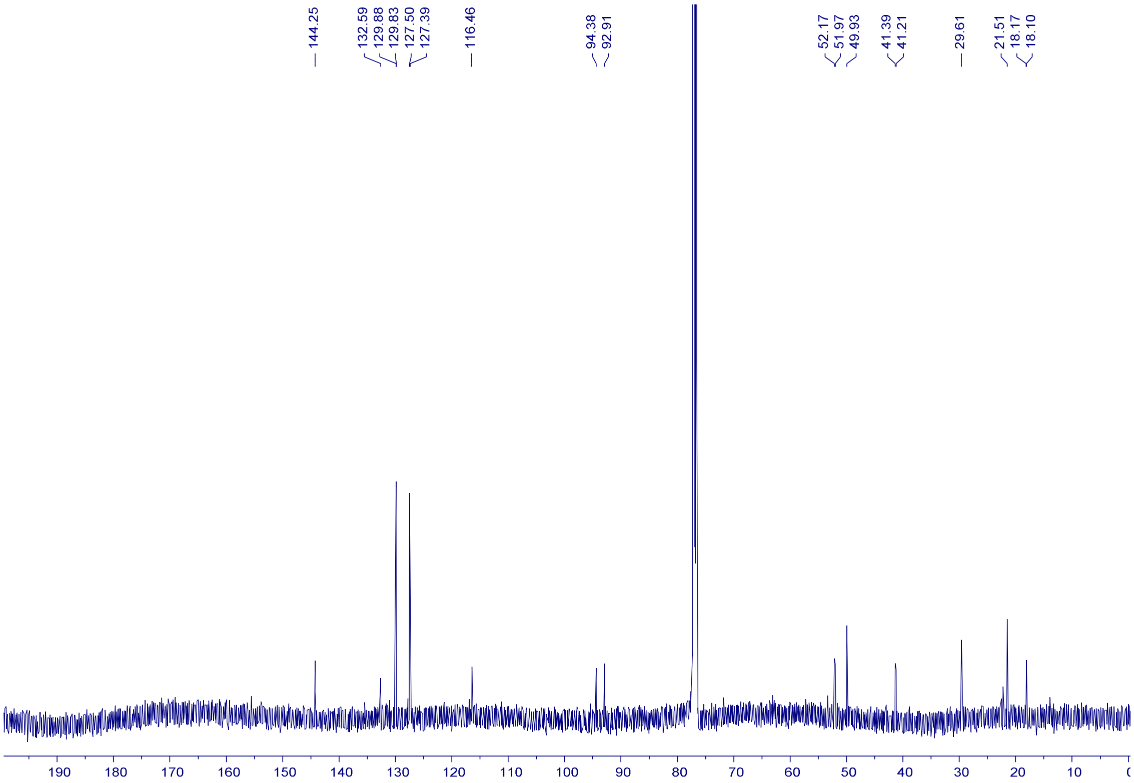

3j ^{19}F NMR (376 MHz, $CDCl_3$)

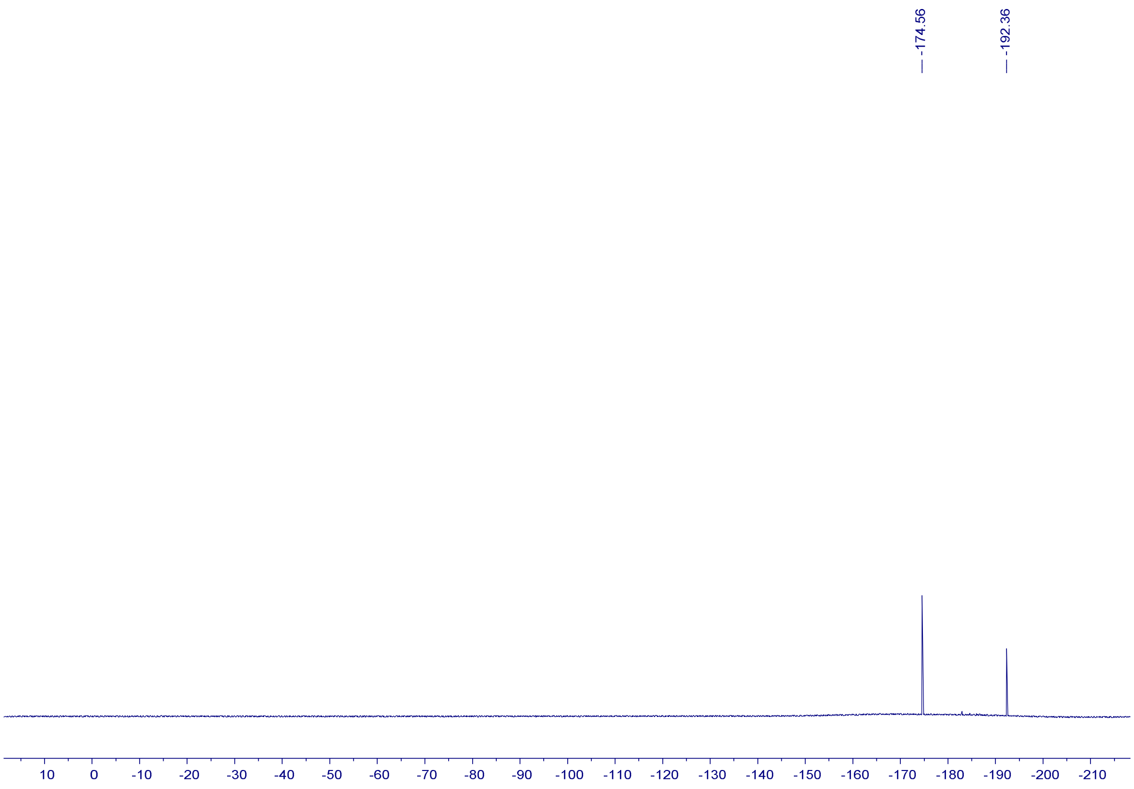

3k ^{1}H NMR (500 MHz, $CDCl_3$)

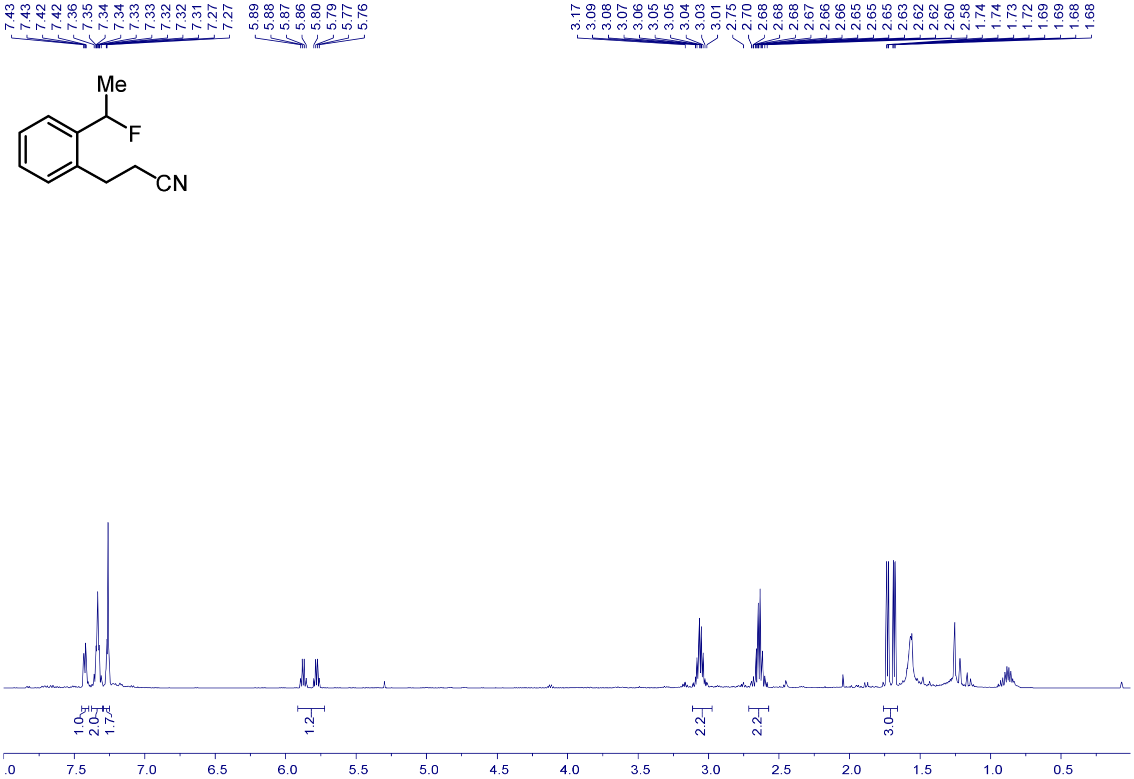

3k ^{13}C NMR (126 MHz, $CDCl_3$)

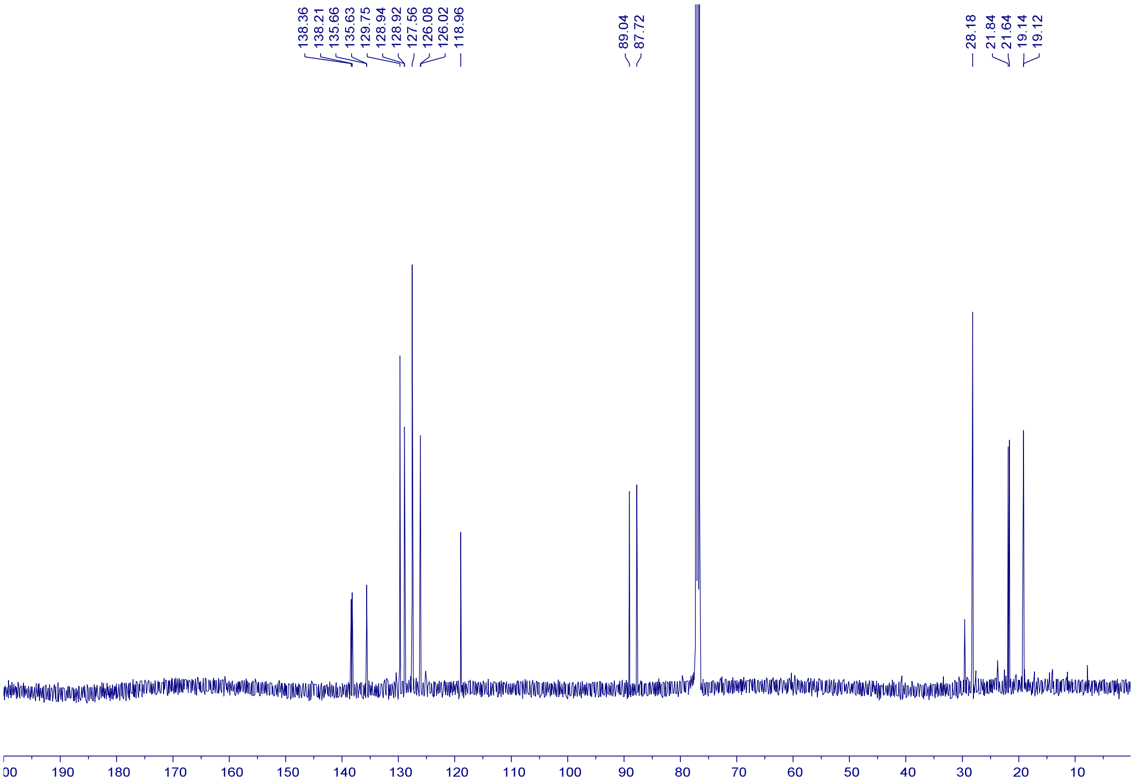

3k ^{19}F NMR (376 MHz, $CDCl_3$)

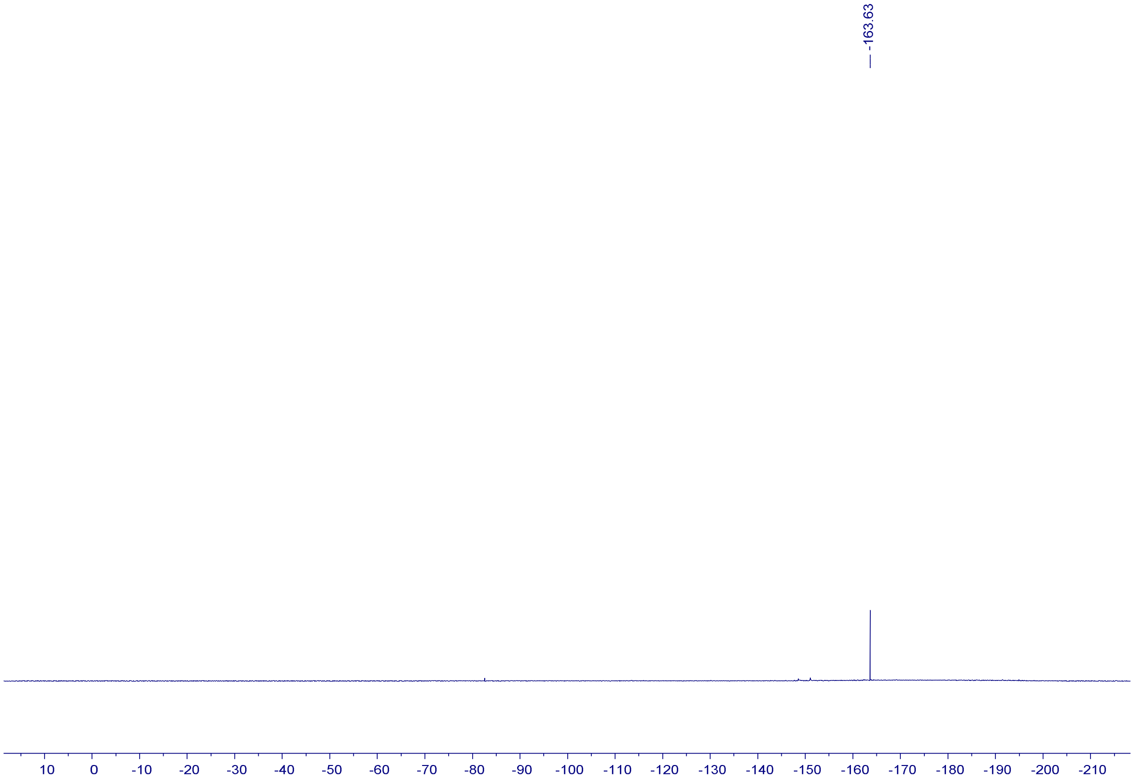

3n ^{1}H NMR (500 MHz, $CDCl_3$)

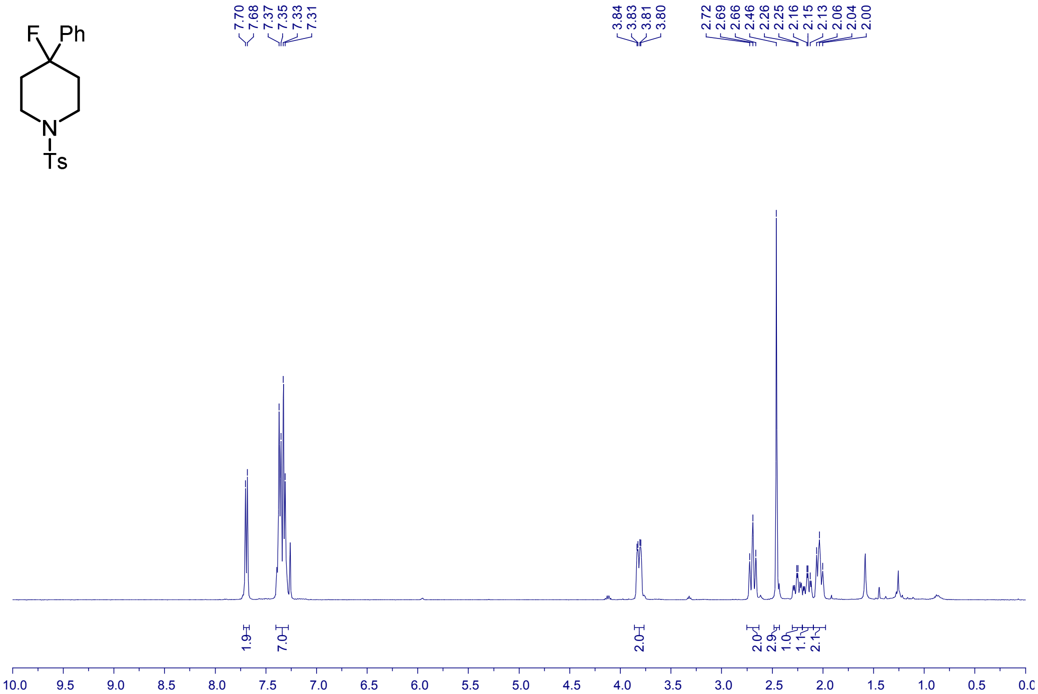

3n ^{13}C NMR (126 MHz, $CDCl_3$)

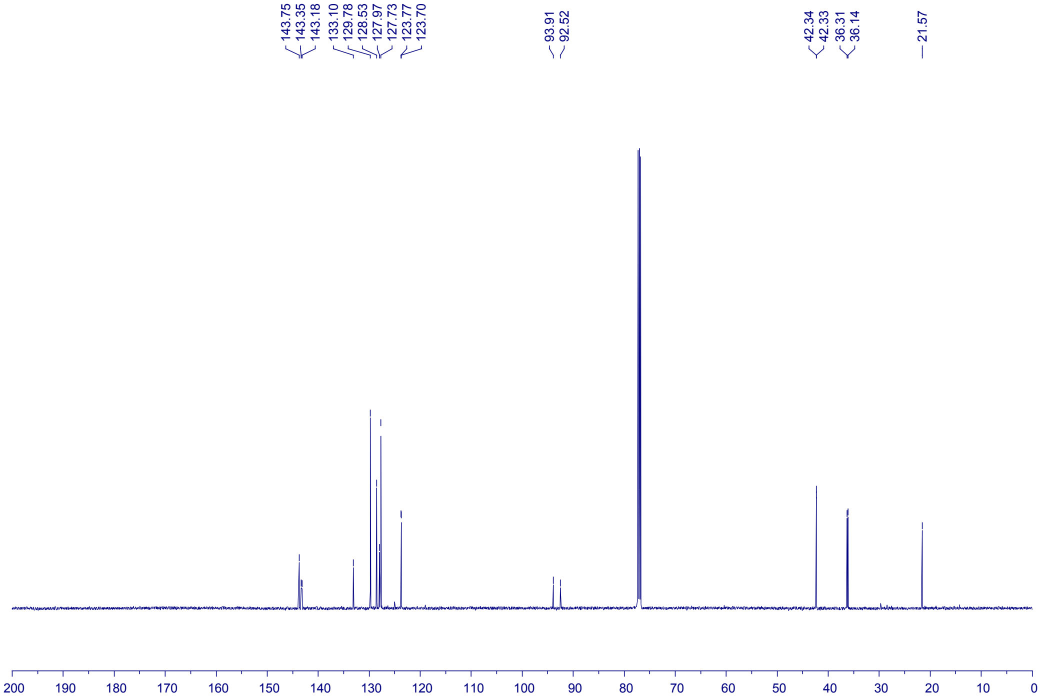

3n ^{1}F NMR (376 MHz, $CDCl_3$)

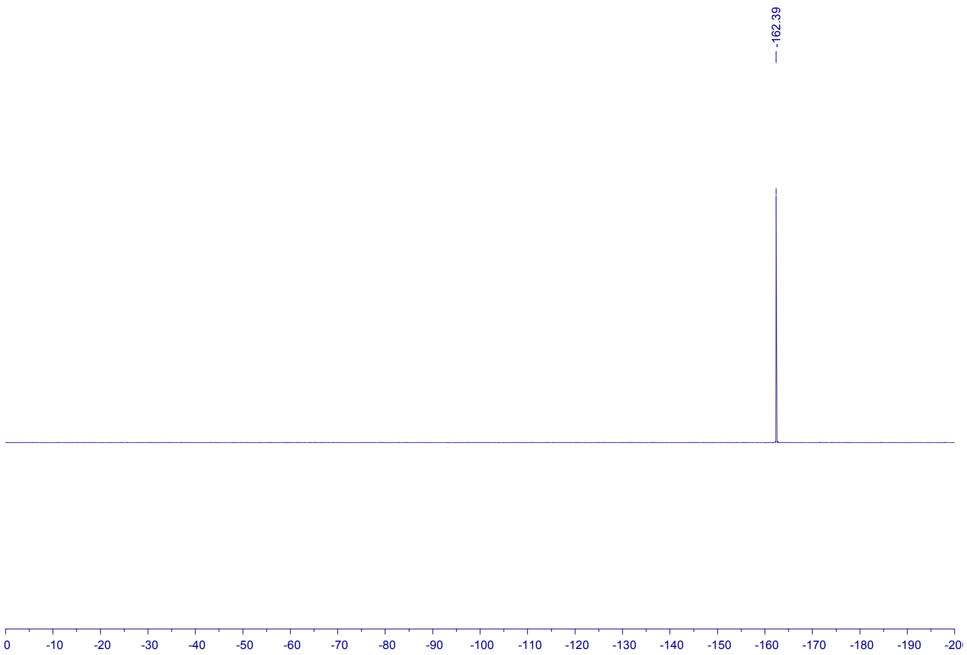

3o ^{1}H NMR (376 MHz, $CDCl_3$)

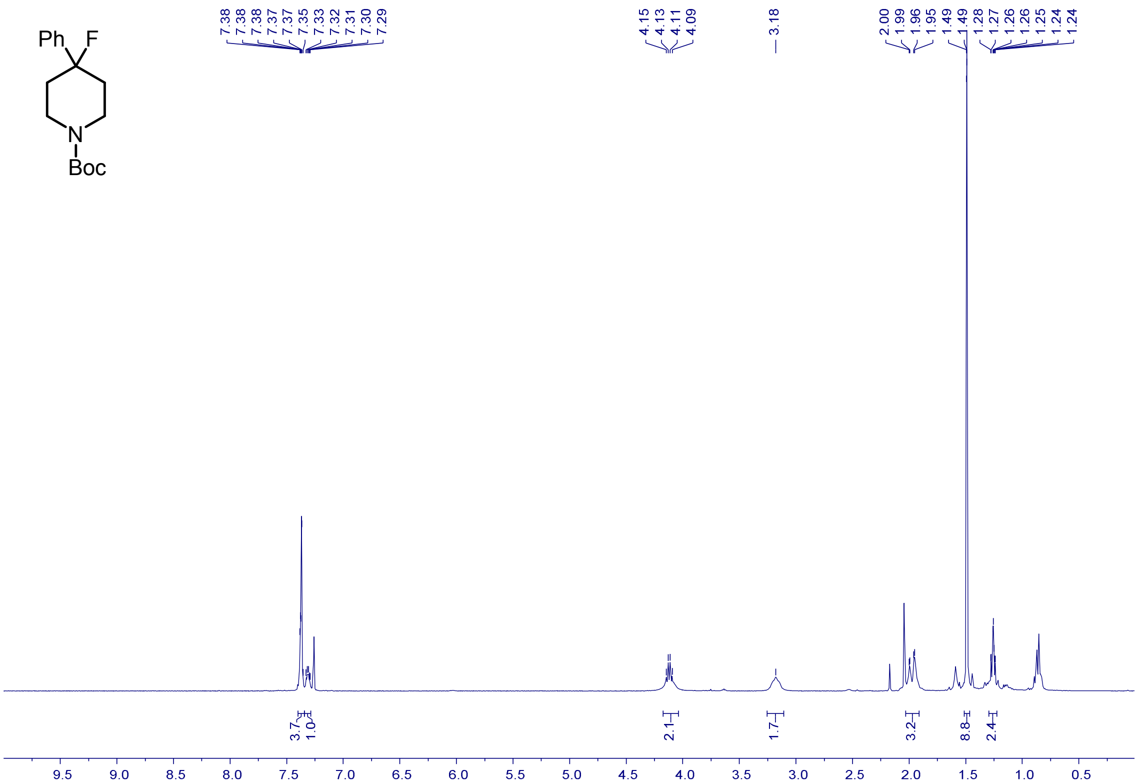

3o ^{13}C NMR (376 MHz, $CDCl_3$)

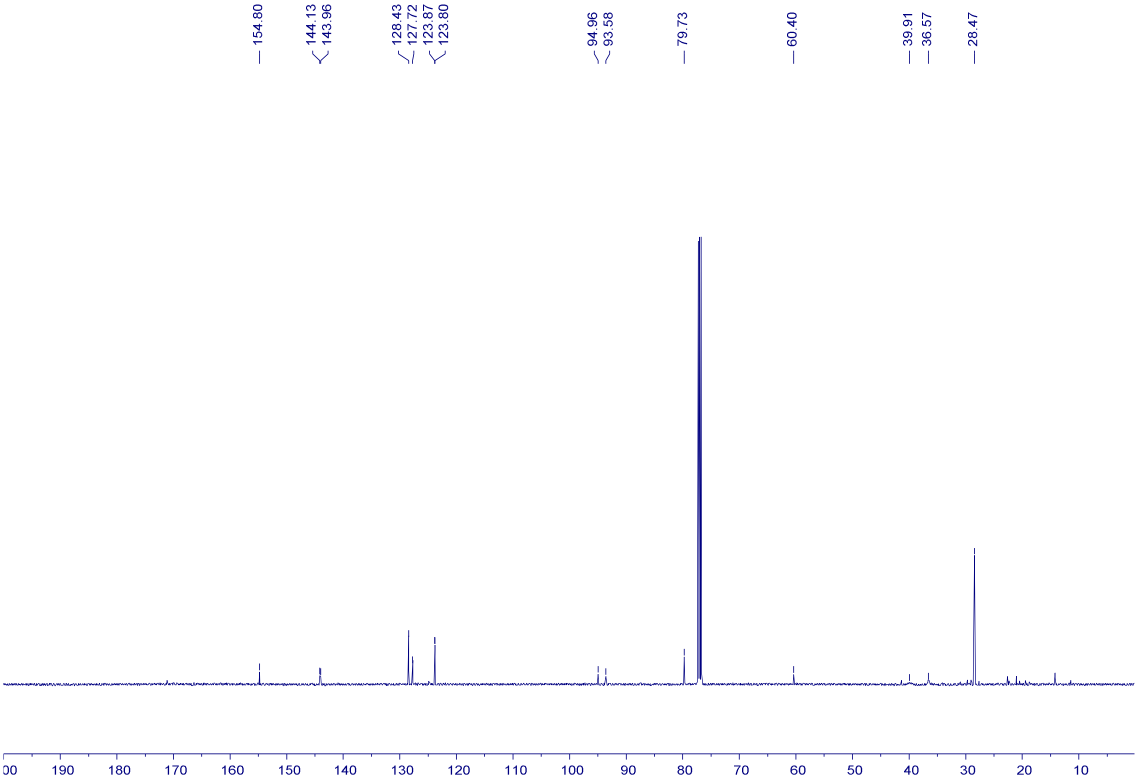

3o ^{19}F NMR (376 MHz, $CDCl_3$)

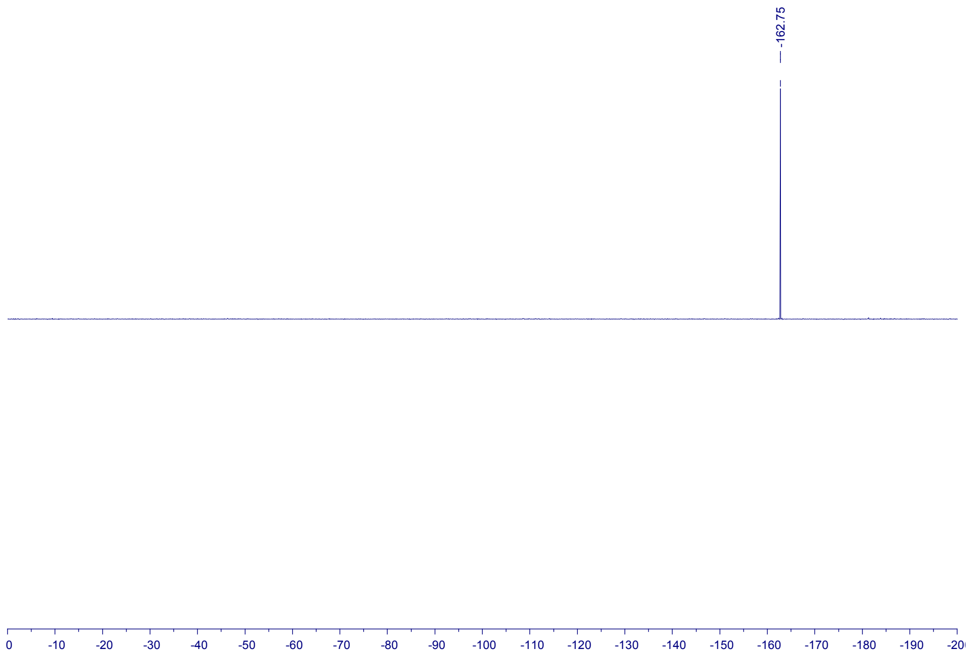

3r ^{1}H NMR (400 MHz, $CDCl_3$)

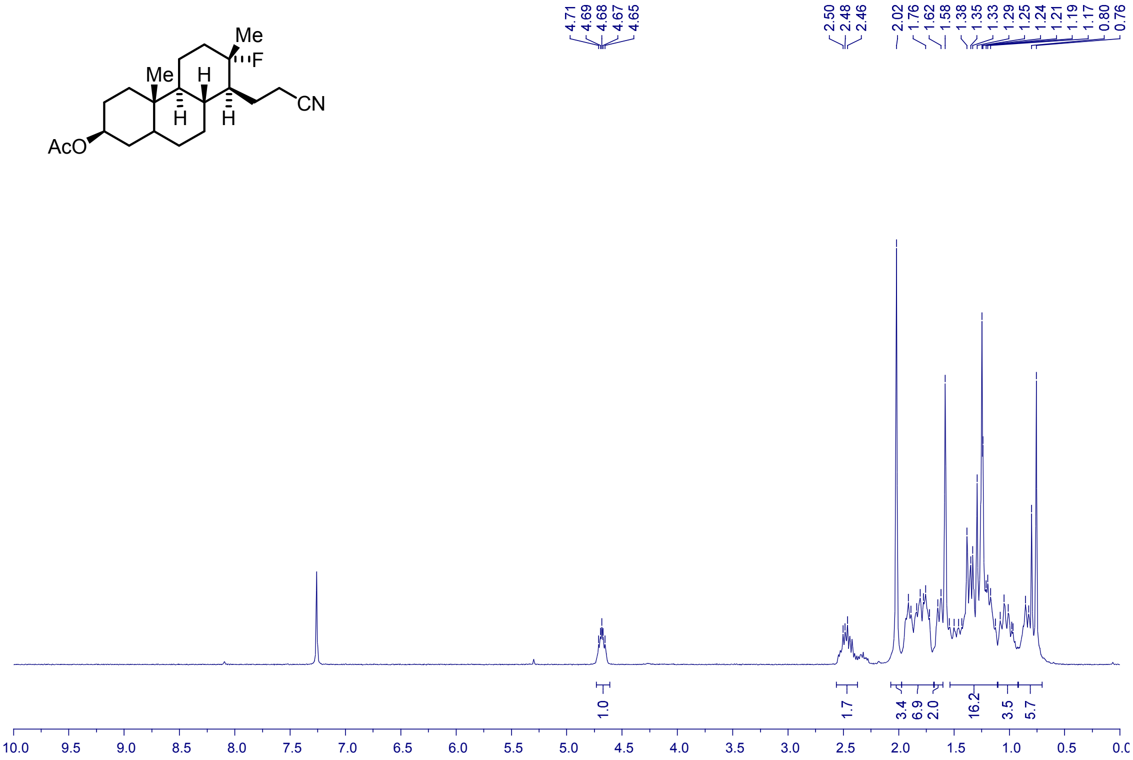

3r ^{13}C NMR (126 MHz, $CDCl_3$)

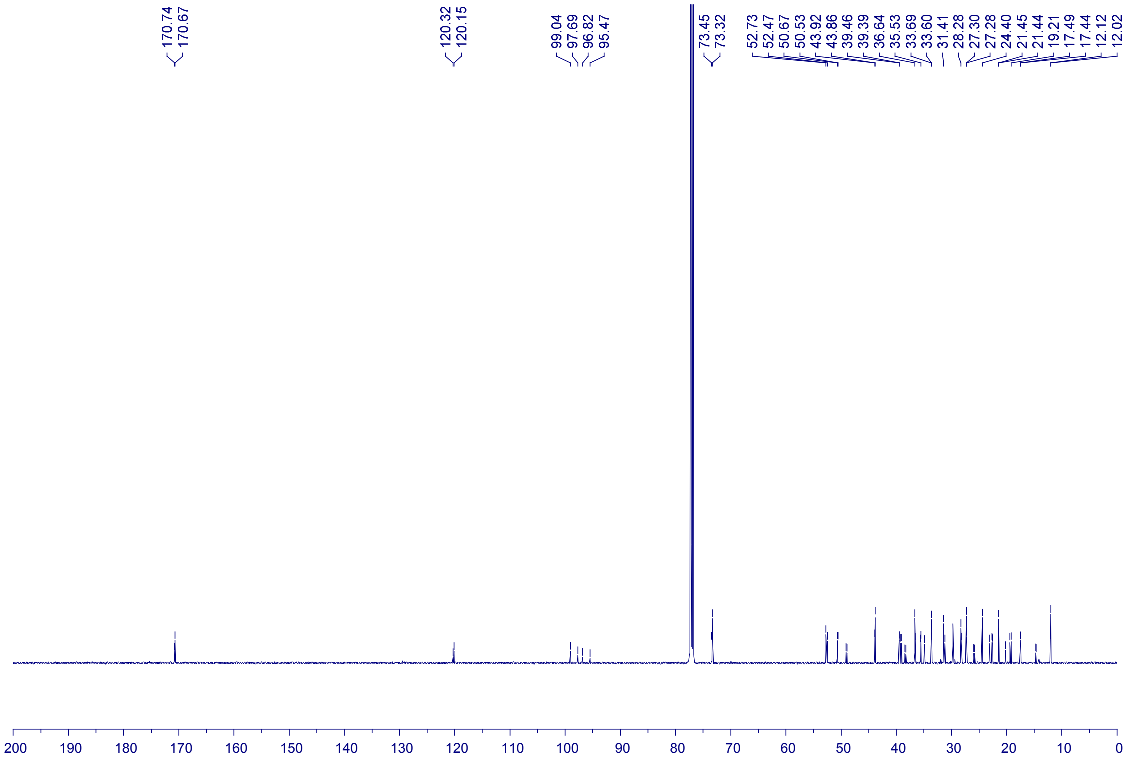

3r ^{19}F NMR (376 MHz, $CDCl_3$)

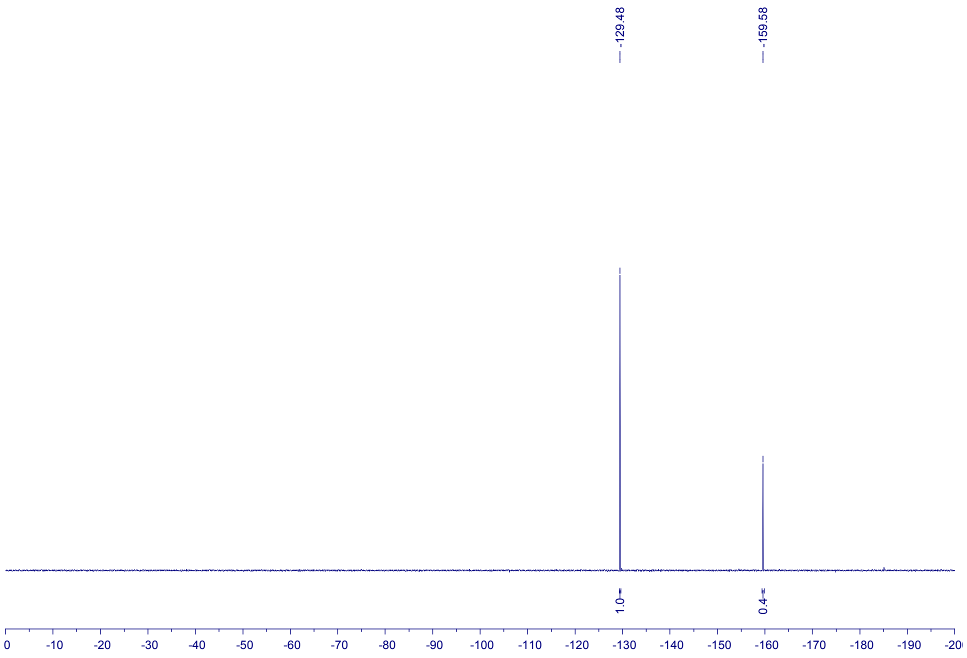

3s ^{1}H NMR (400 MHz, $CDCl_3$)

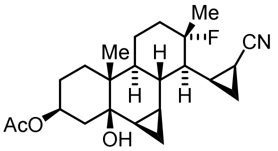

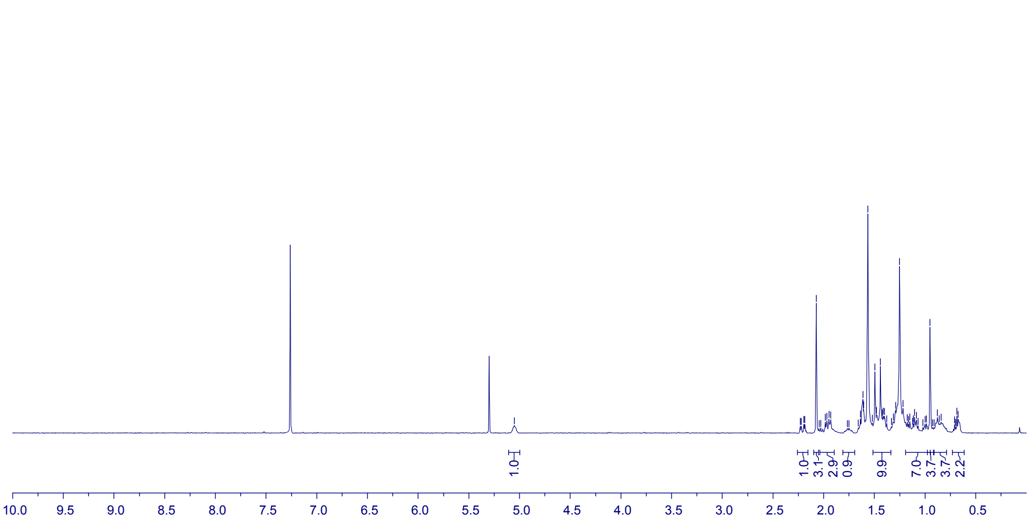

3s ^{13}C NMR (126 MHz, $CDCl_3$)

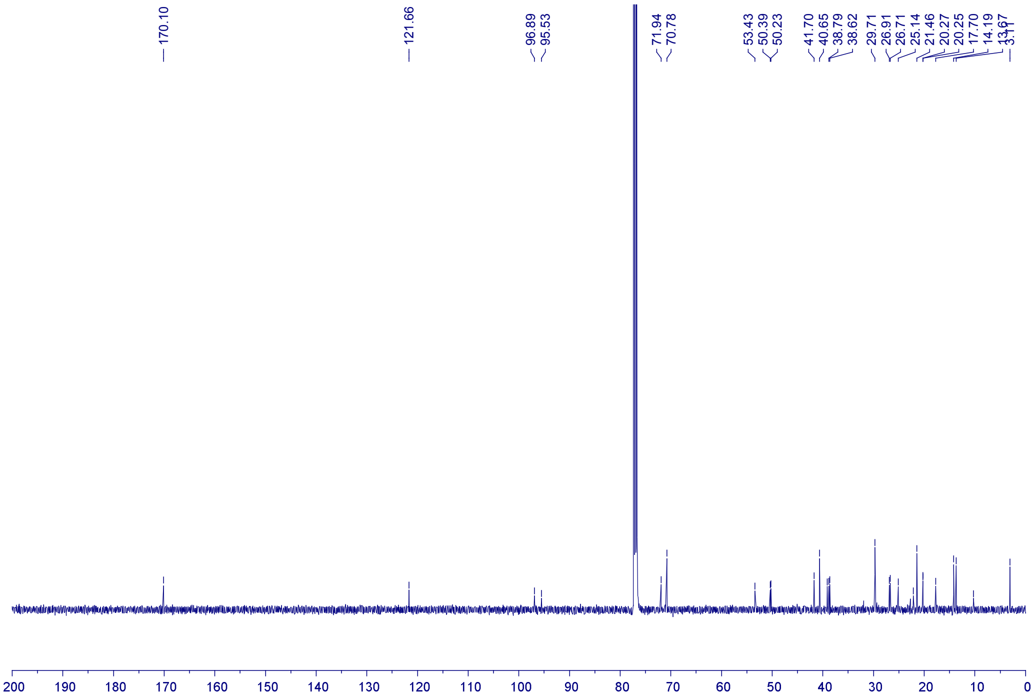

3s ^{19}F NMR (376 MHz, $CDCl_3$)

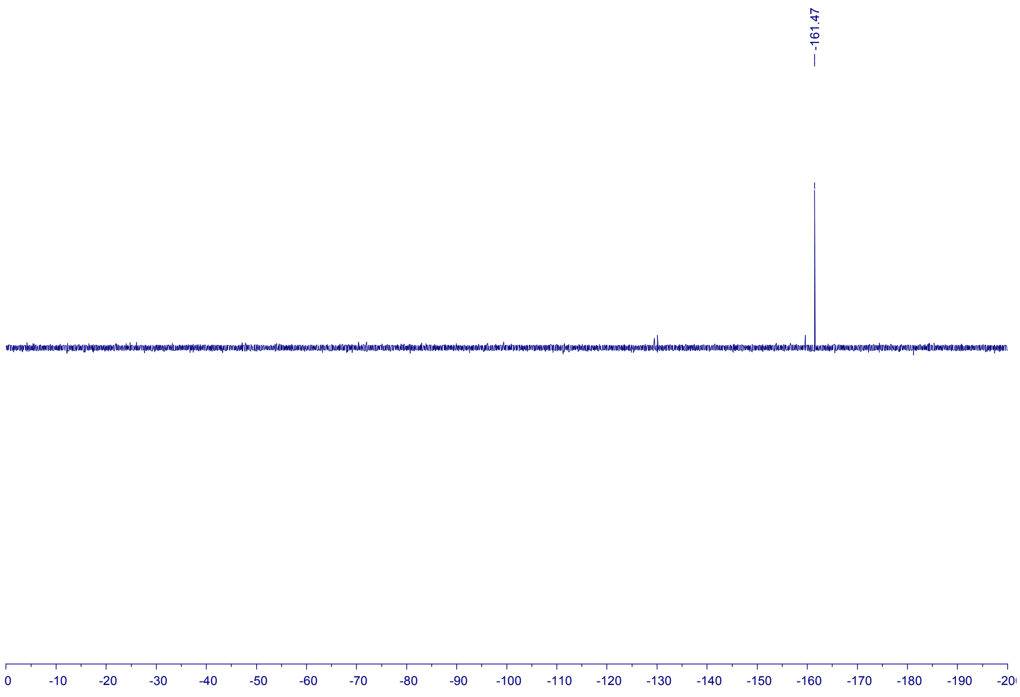

3t ^{1}H NMR (500 MHz, $CDCl_3$)

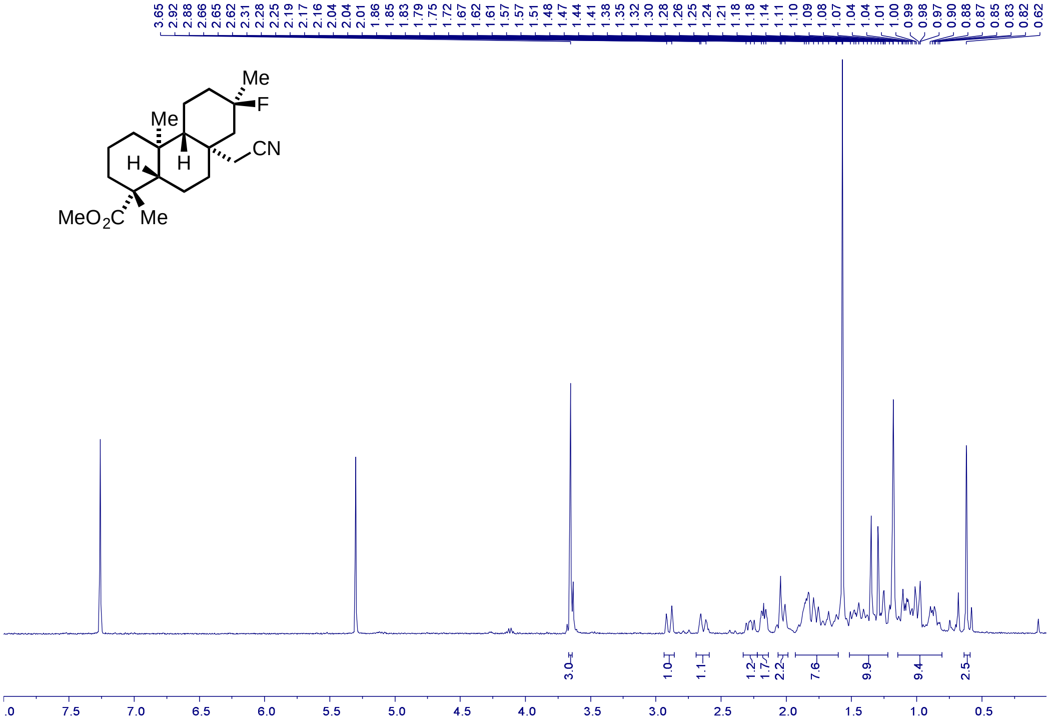

3t ^{13}C NMR (126 MHz, $CDCl_3$)

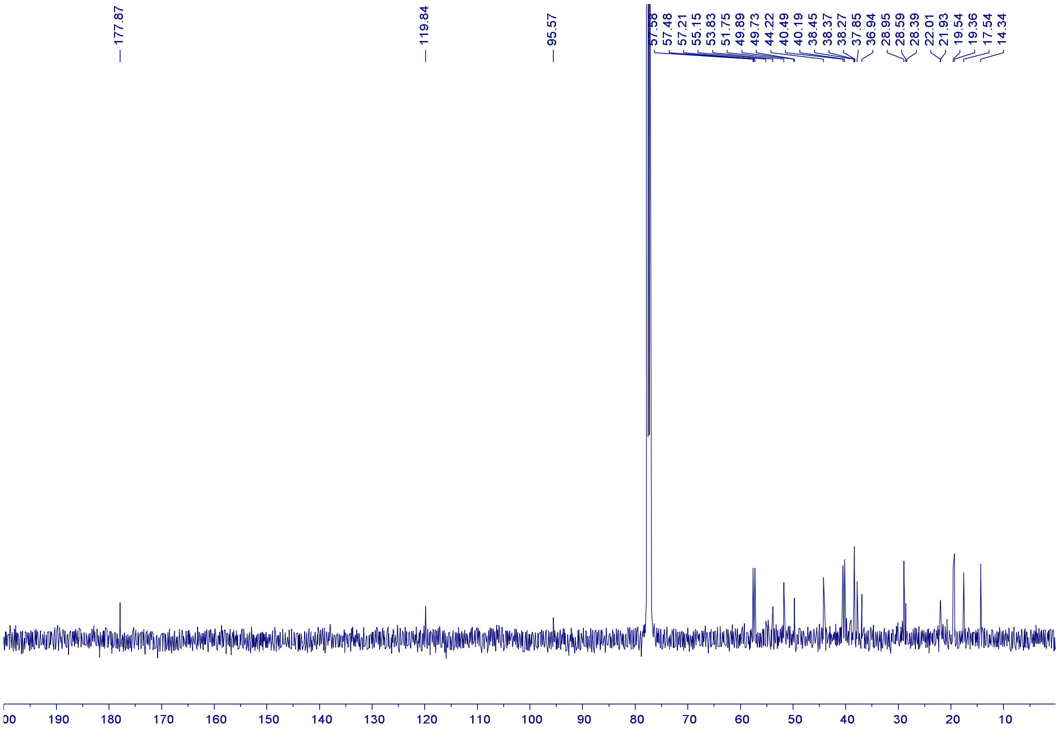

3t ^{19}F NMR (471 MHz, $CDCl_3$)

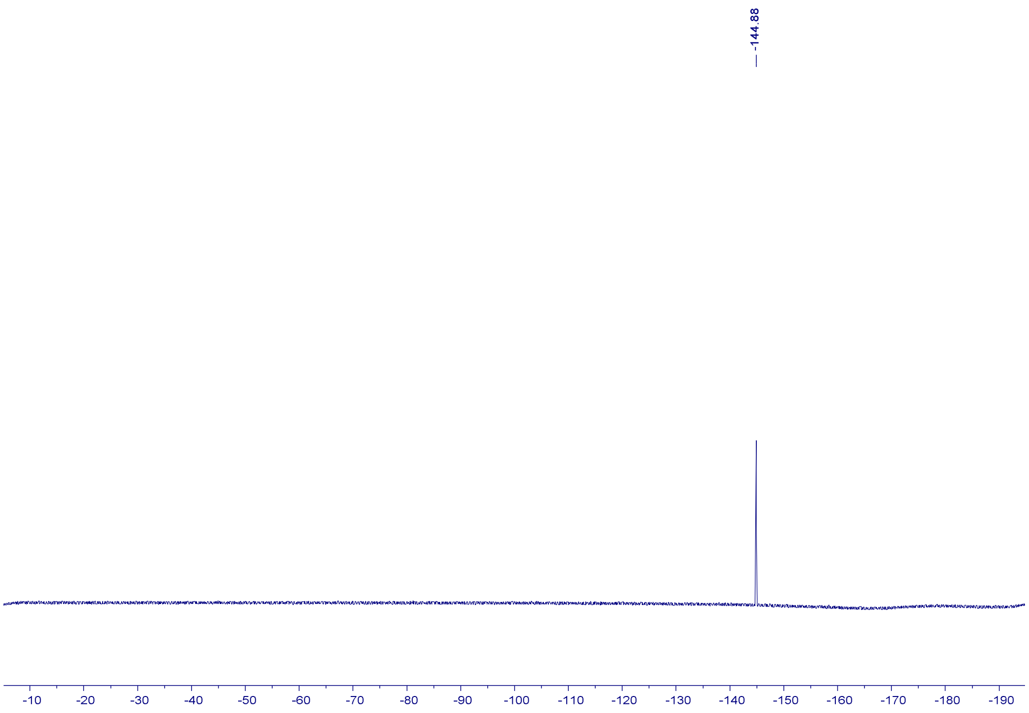

4a ^{1}H NMR (400 MHz, $CDCl_3$)

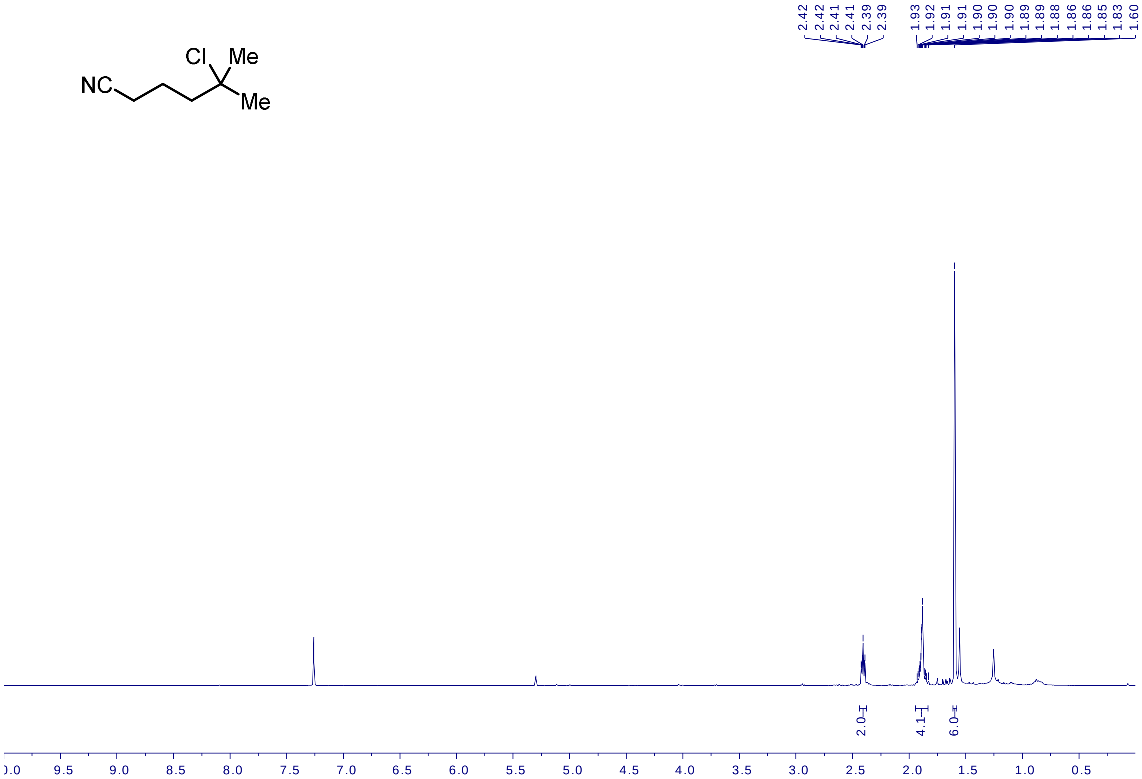

4a ^{13}C NMR (126 MHz, $CDCl_3$)

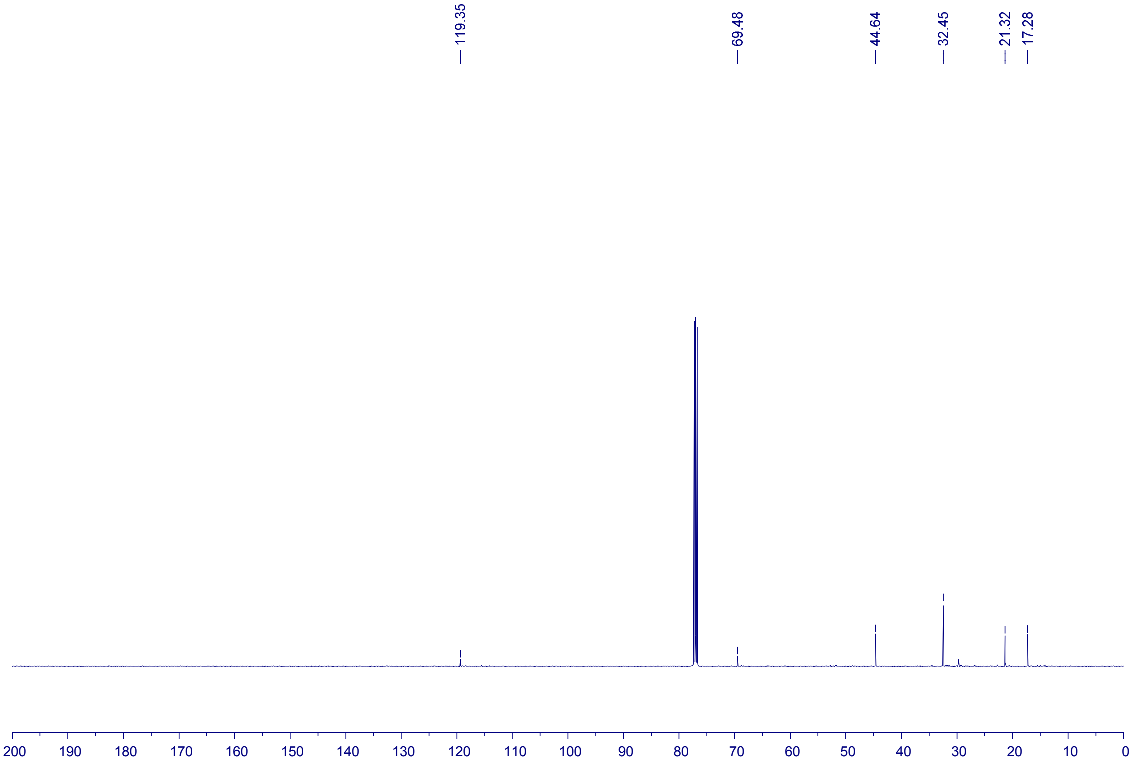

4c ^{1}H NMR (400 MHz, $CDCl_3$)

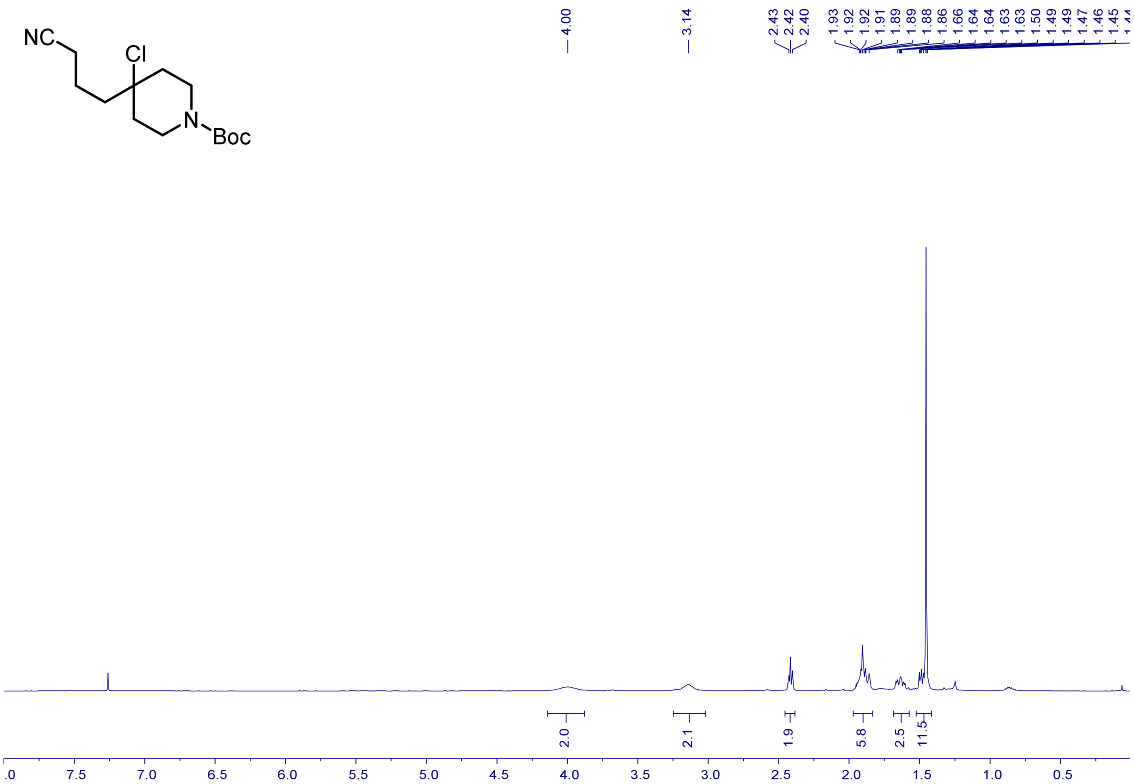

4c ^{13}C NMR (126 MHz, $CDCl_3$)

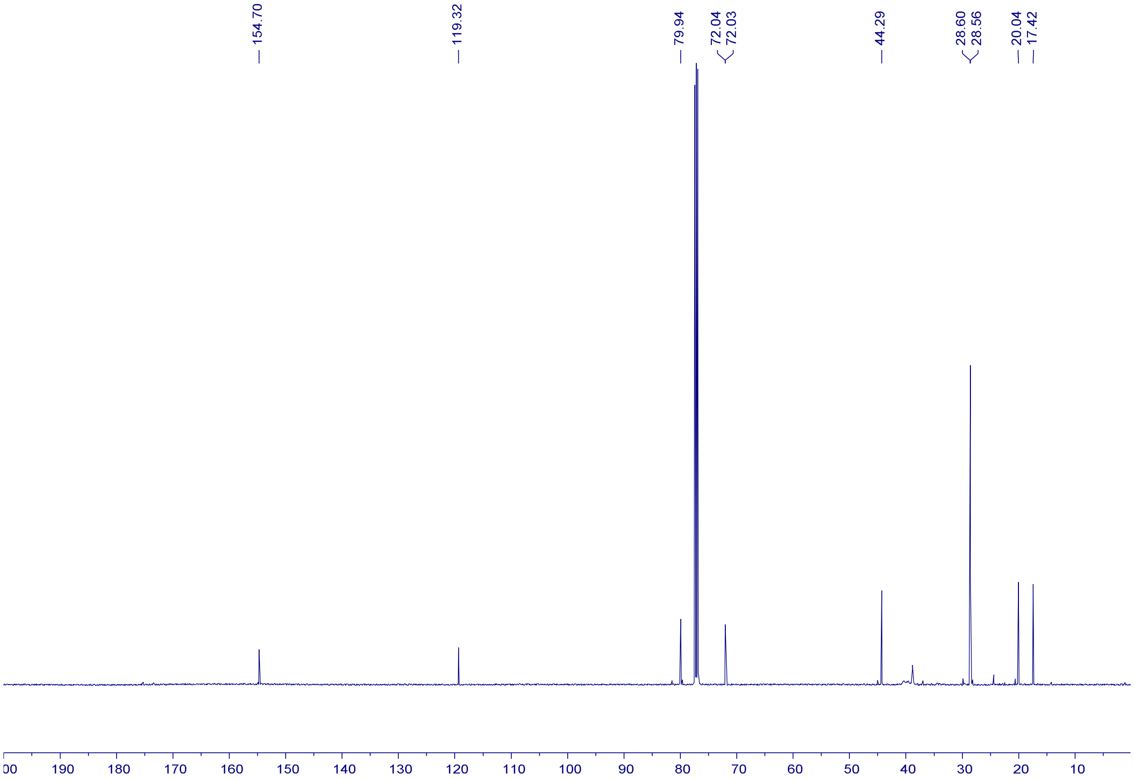

4d ^{1}H NMR (400 MHz, $CDCl_3$)

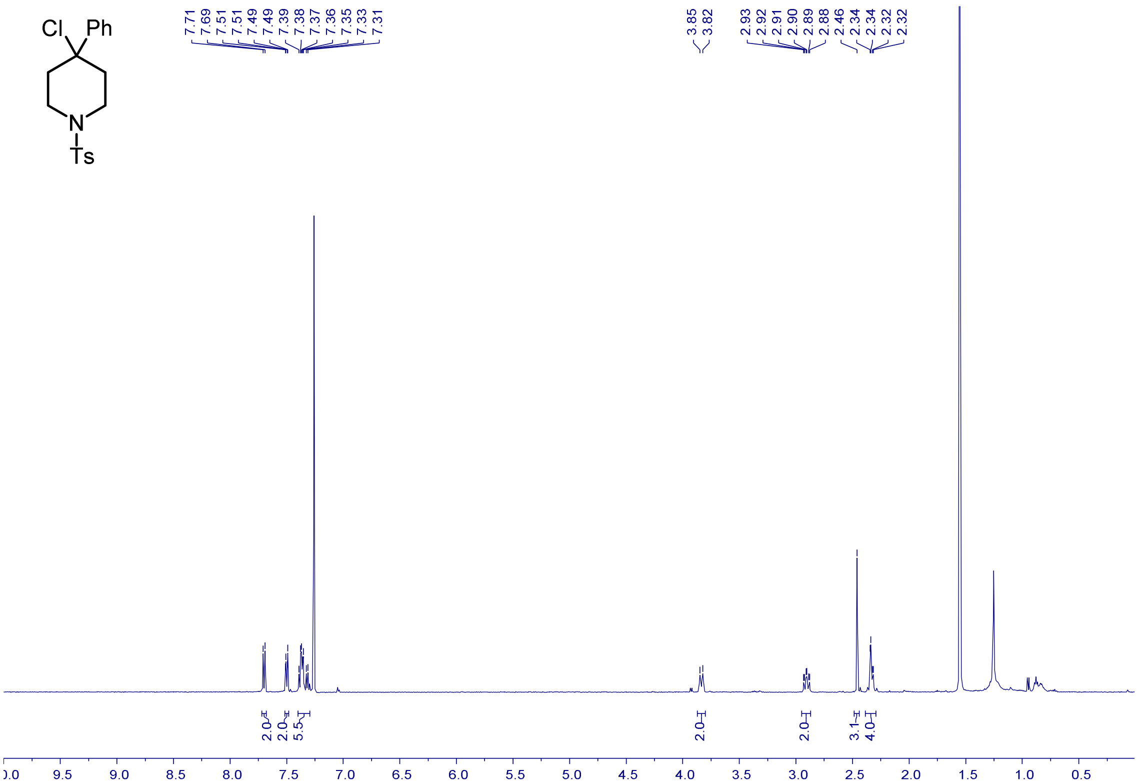

4d ^{13}C NMR (126 MHz, $CDCl_3$)

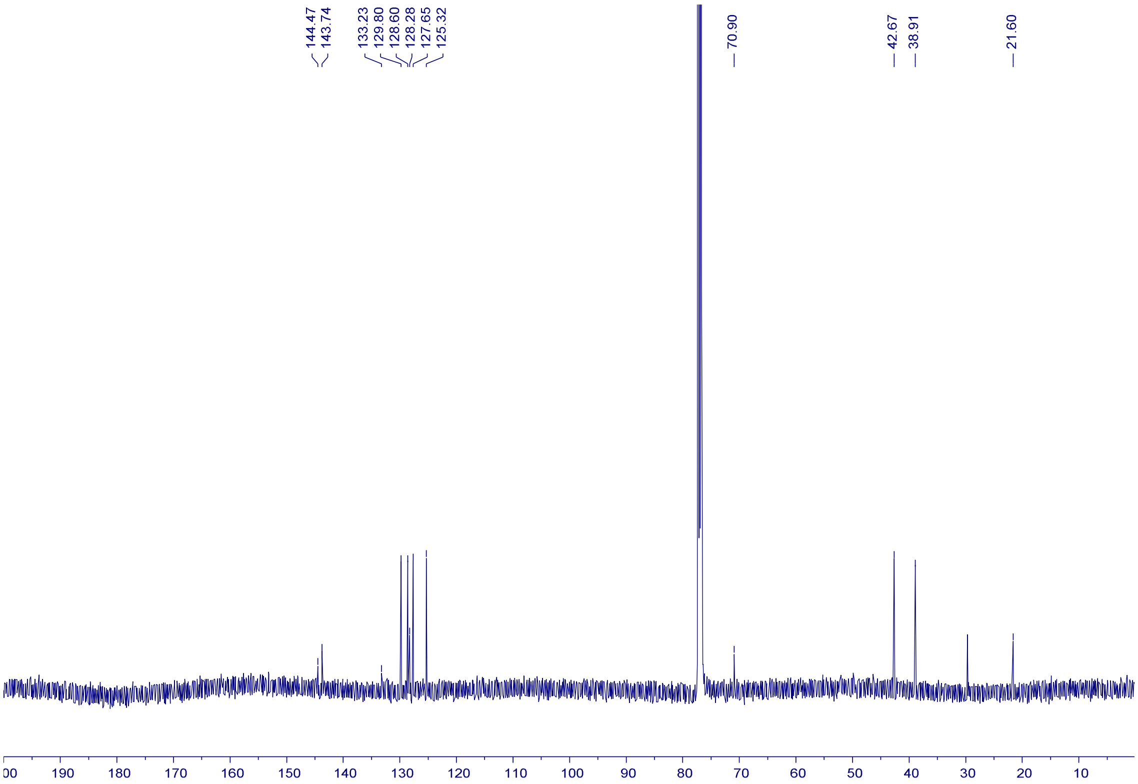

4e ^{1}H NMR (400 MHz, $CDCl_3$)

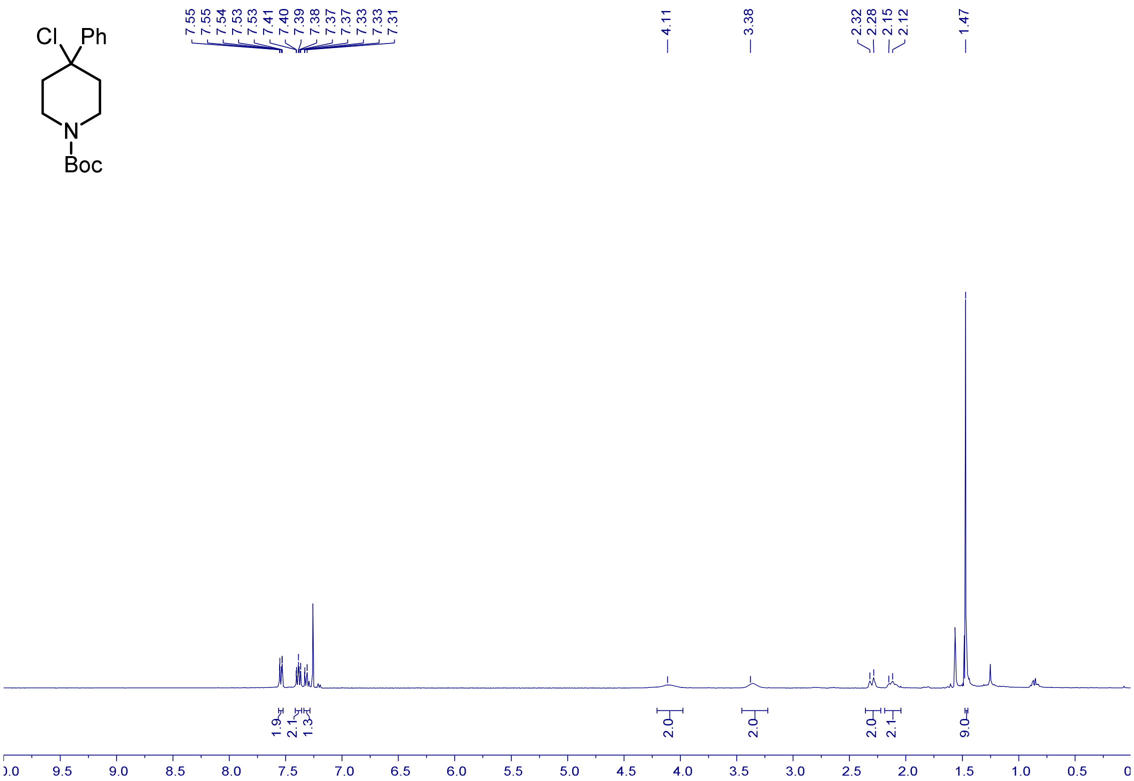

4e ^{13}C NMR (126 MHz, $CDCl_3$)

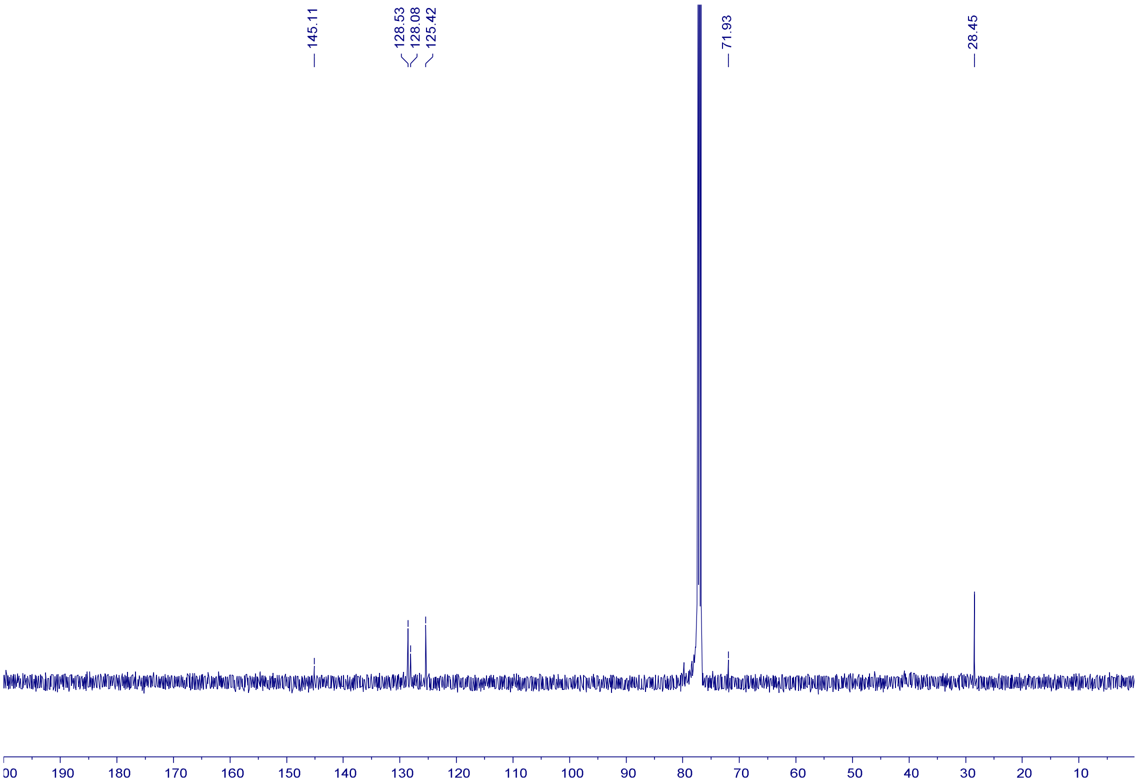

4f ^{1}H NMR (400 MHz, $CDCl_3$)

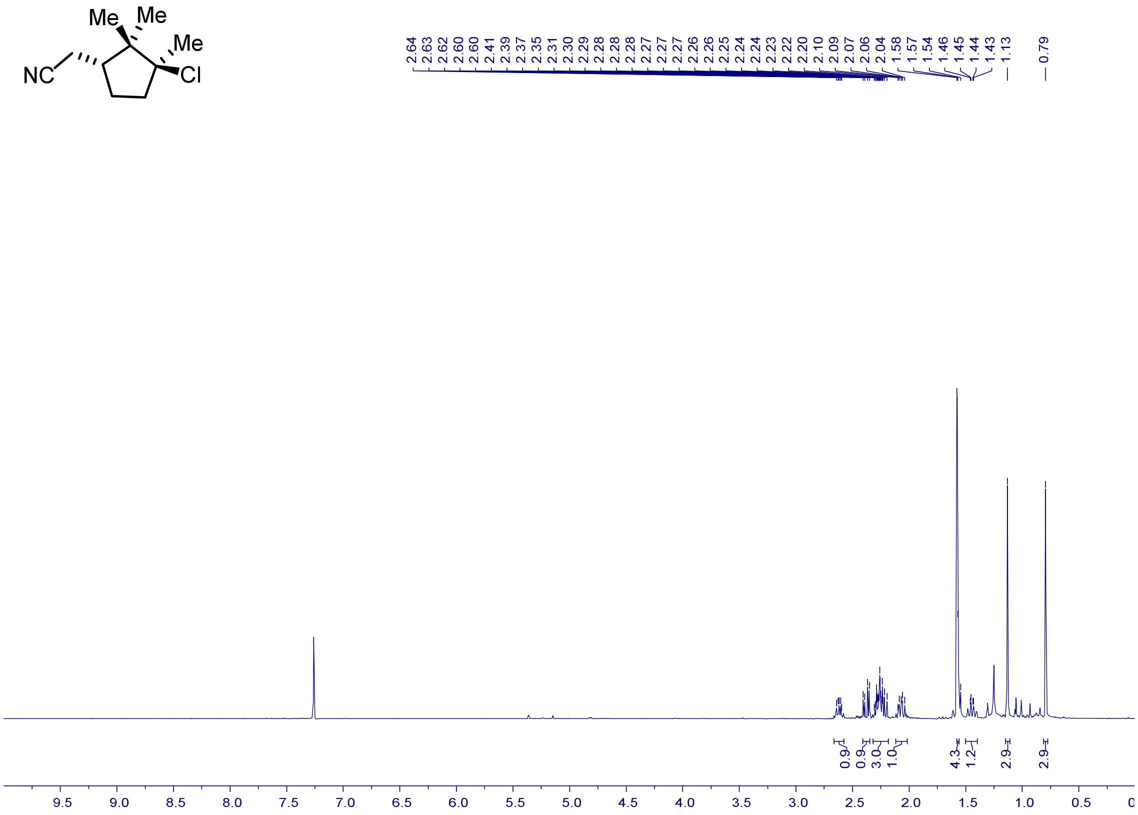

4f ^{13}C NMR (400 MHz, $CDCl_3$)

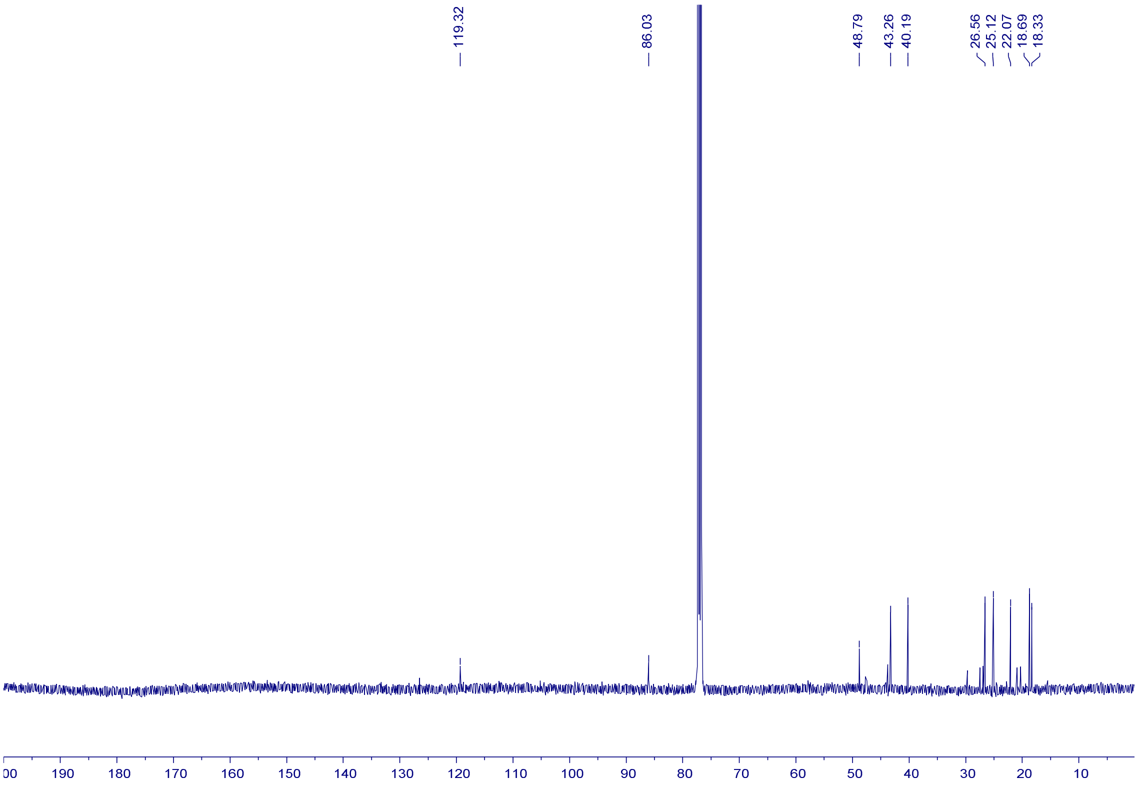

4g ^{1}H NMR (400 MHz, $CDCl_3$)

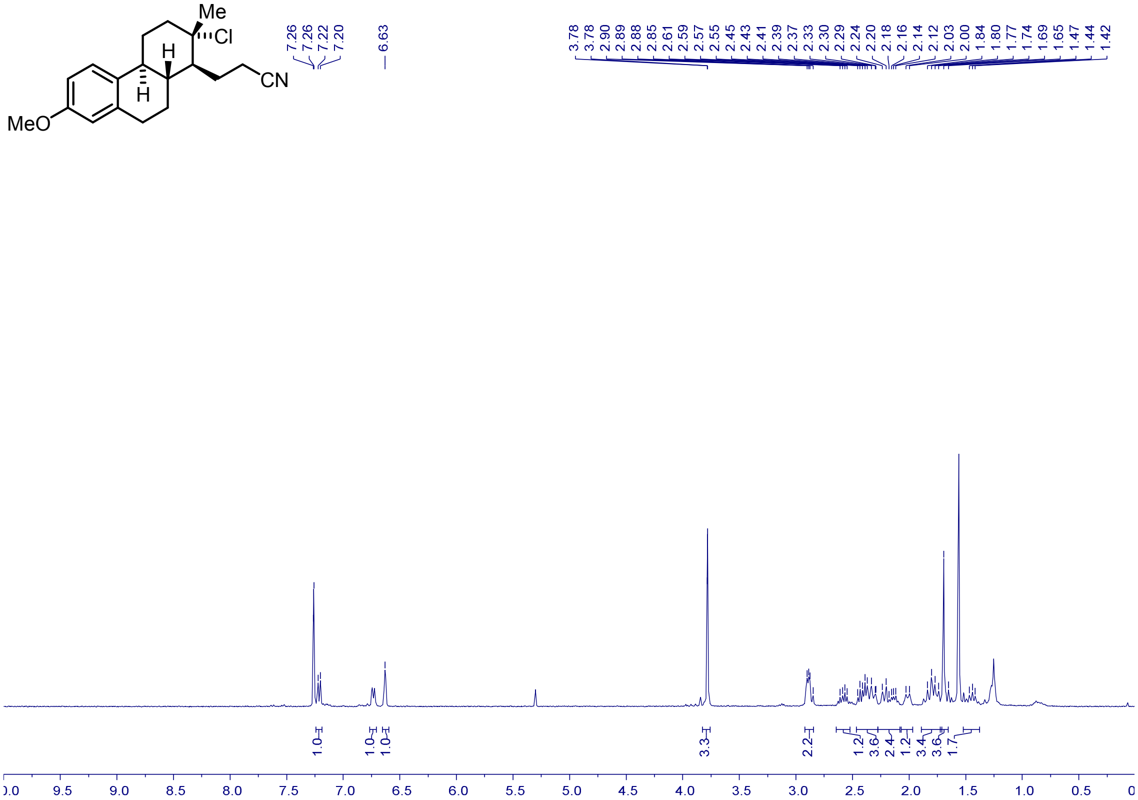

4g ^{13}C NMR (126 MHz, $CDCl_3$)

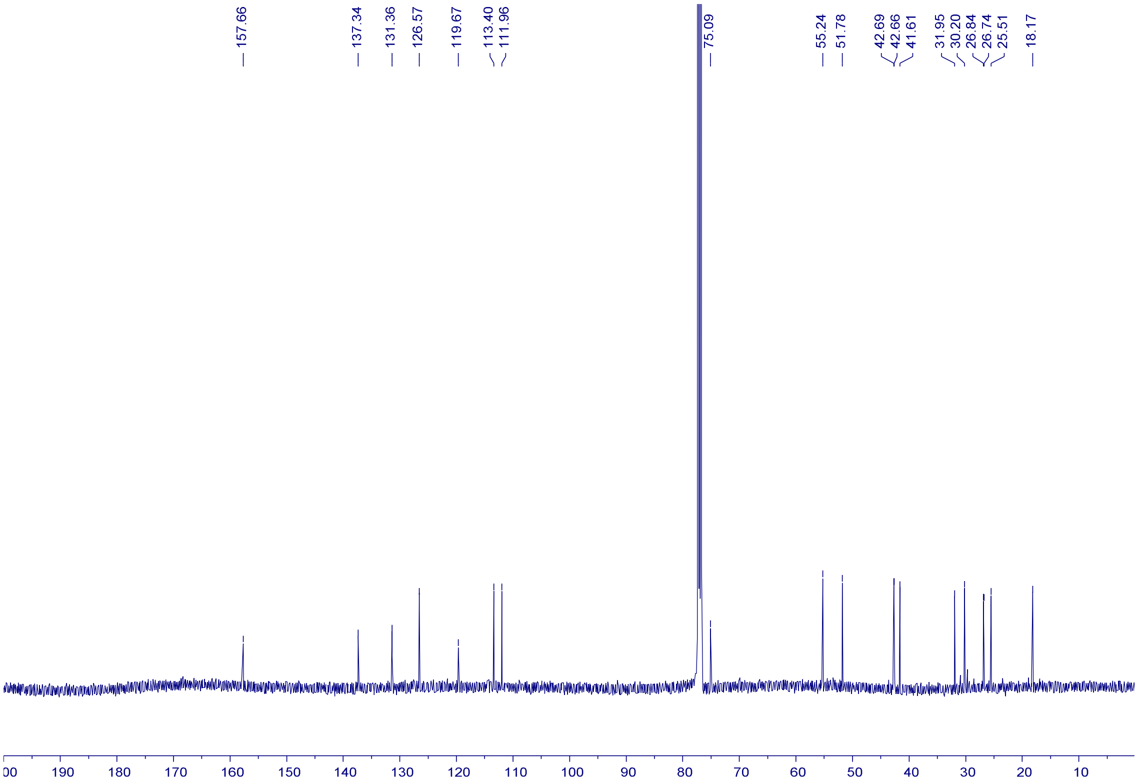

4h ^{1}H NMR (400 MHz, $CDCl_3$)

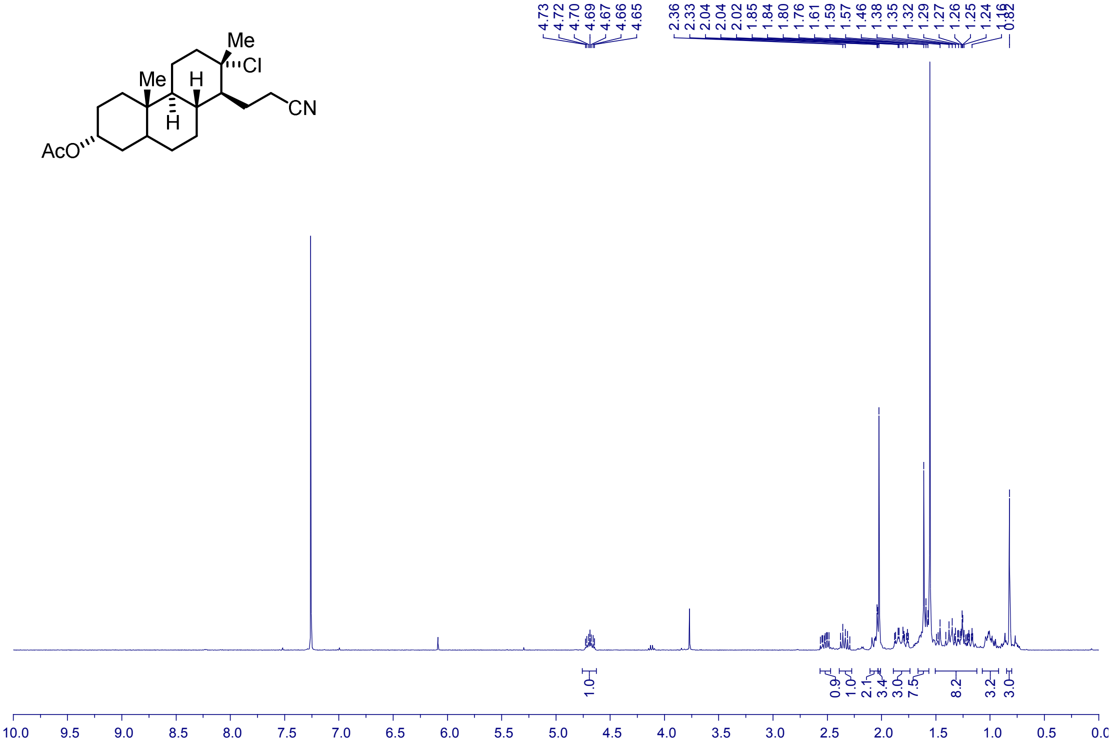

4h ^{13}C NMR (126 MHz, $CDCl_3$)

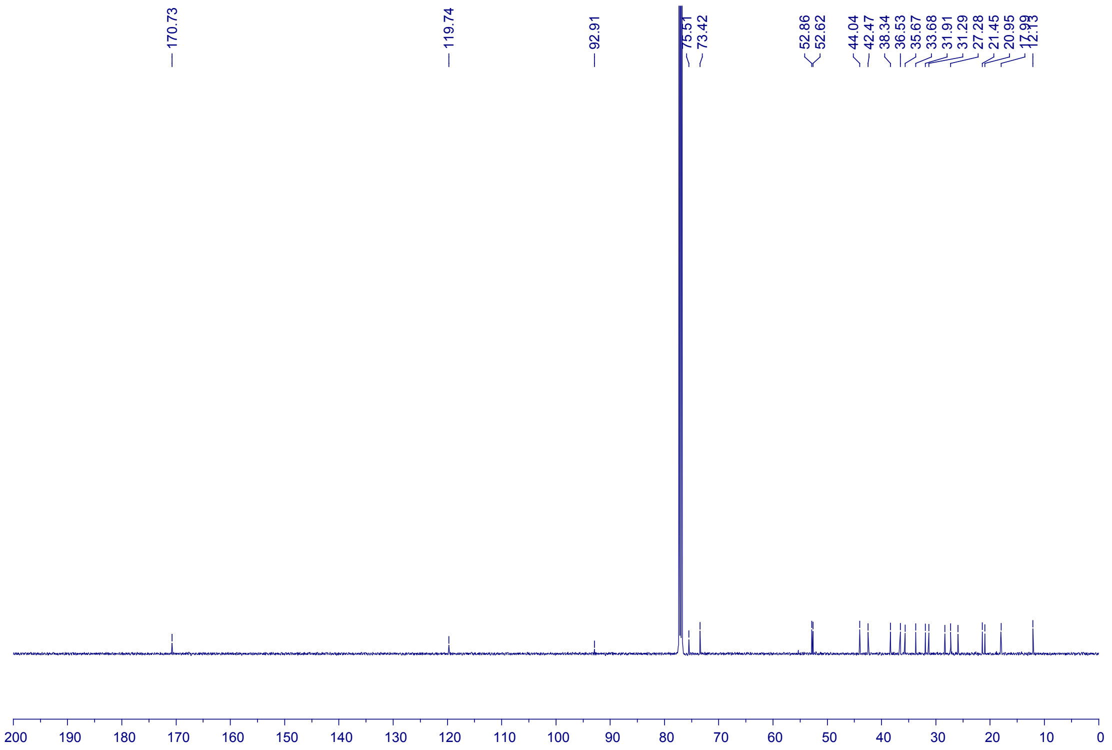

4i ^{1}H NMR (500 MHz, $CDCl_3$)

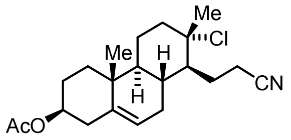

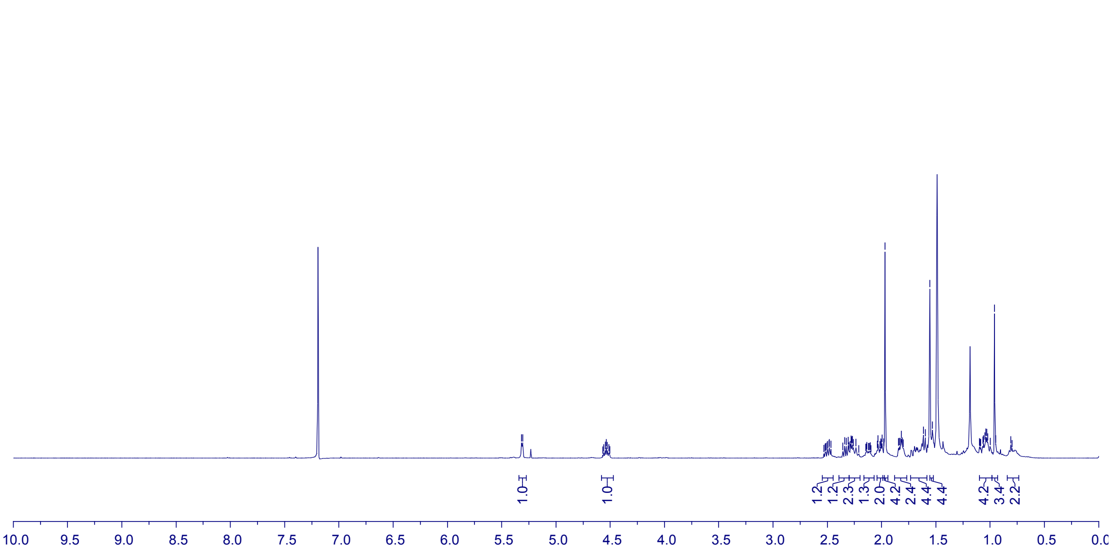

4i ^{13}C NMR (126 MHz, $CDCl_3$)

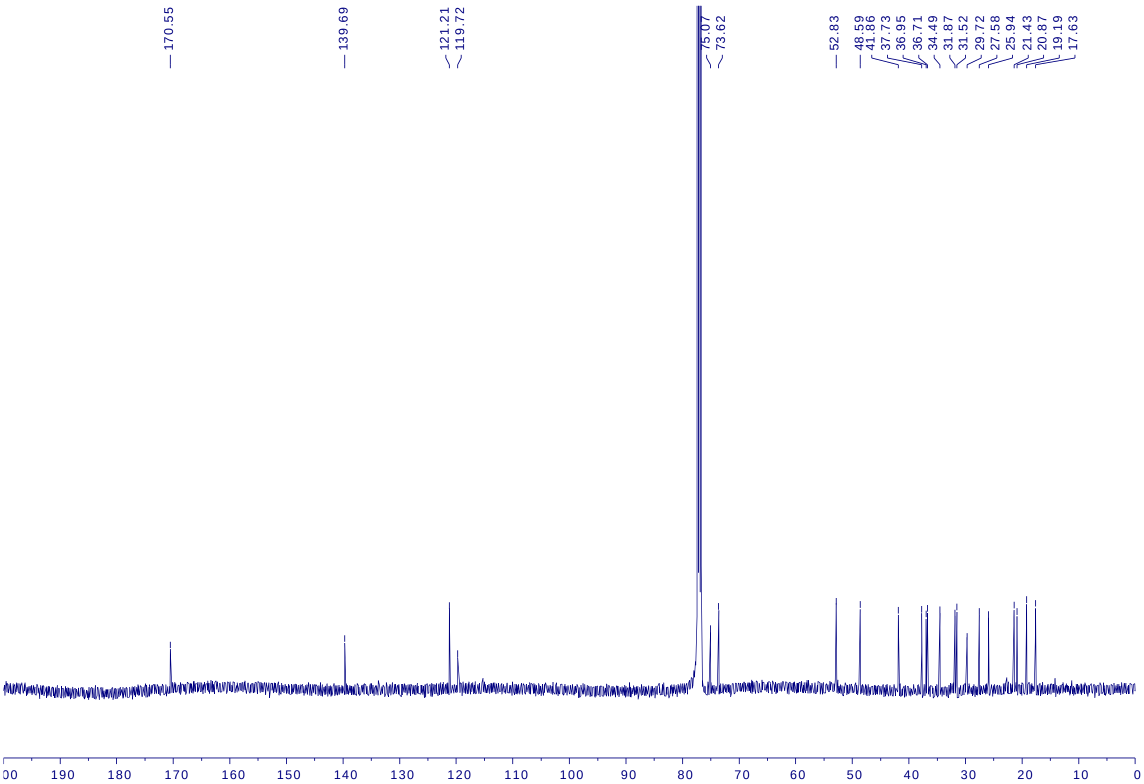

4j ^{1}H NMR (400 MHz, $CDCl_3$)

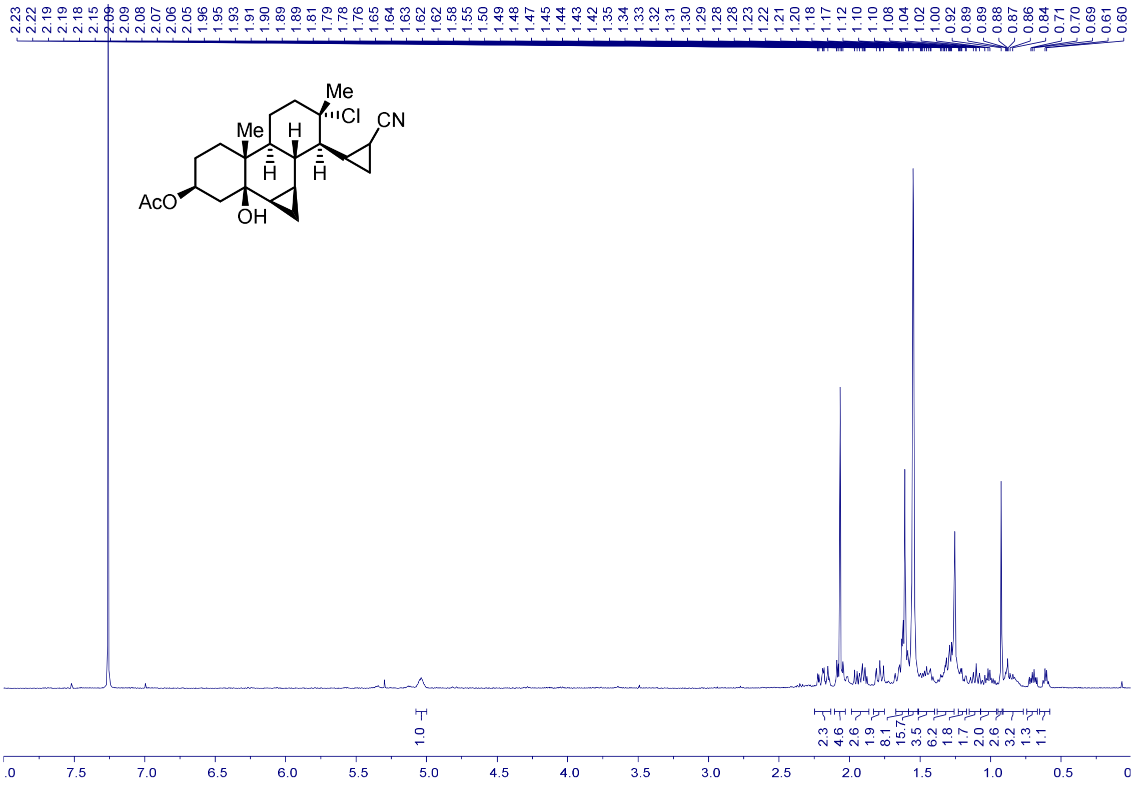

4j ^{13}C NMR (101 MHz, $CDCl_3$)

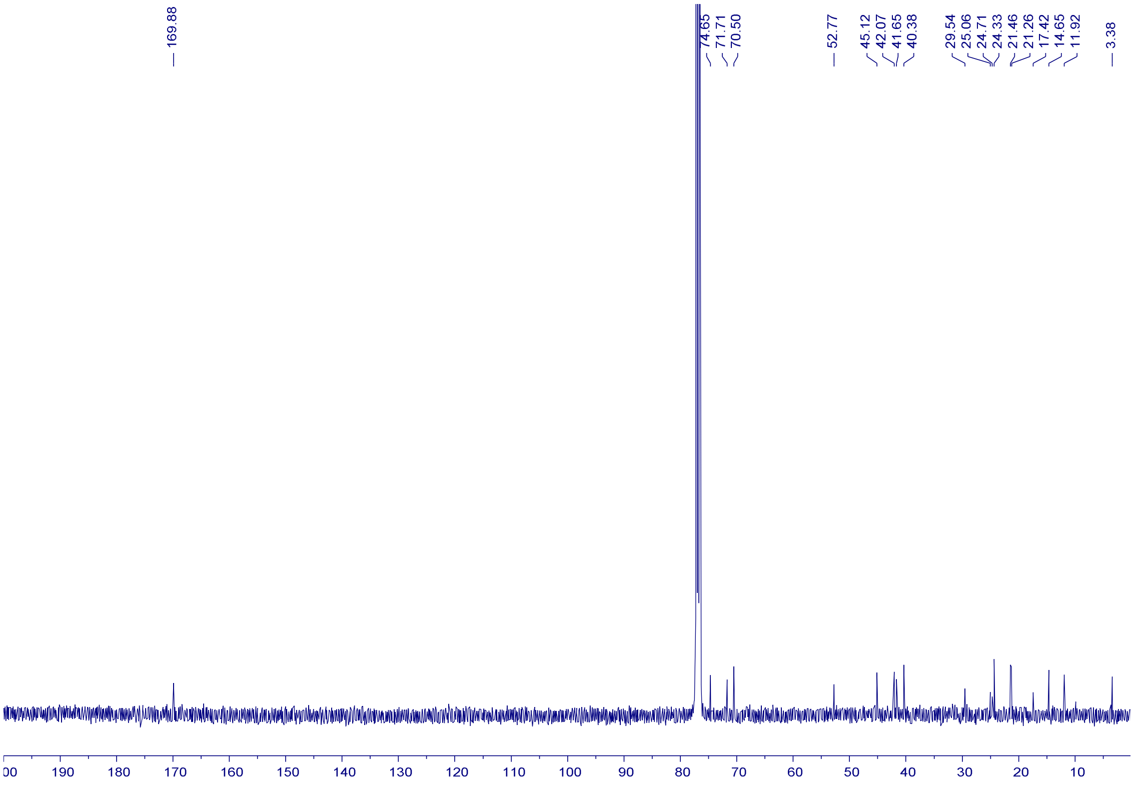

5b ^{1}H NMR (400 MHz, $CDCl_3$) inseparable 1:1 mixture with **S2**

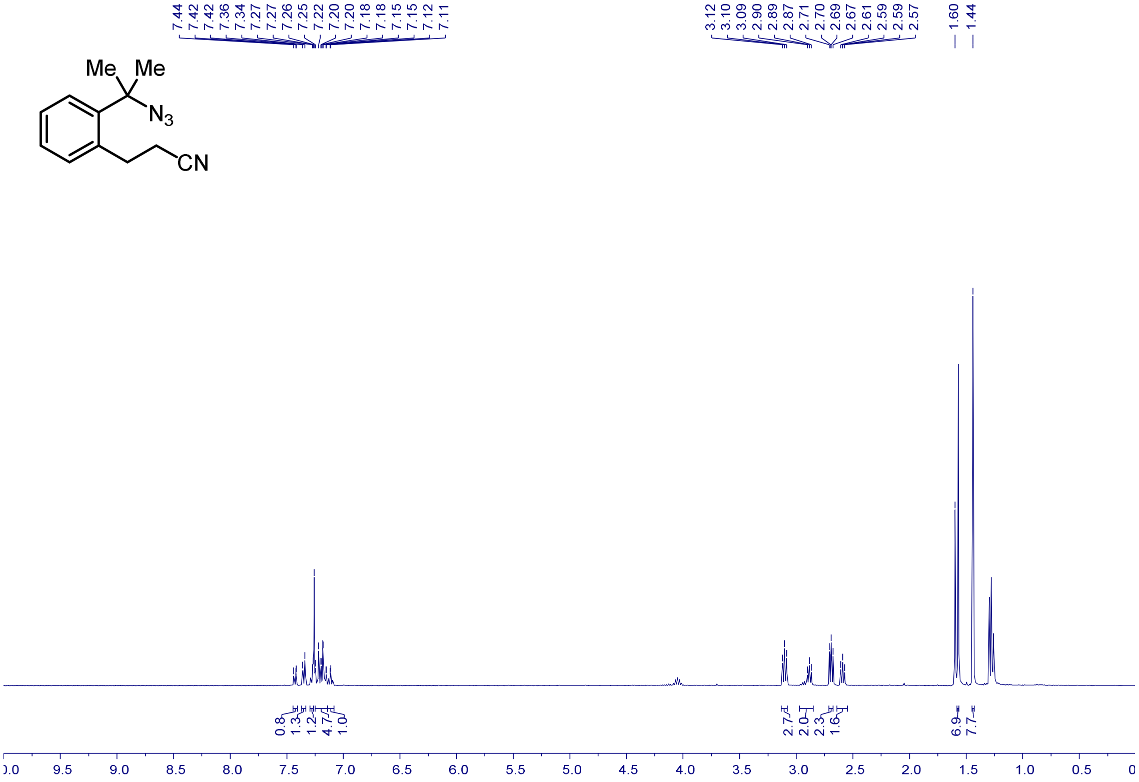

5b ^{13}C NMR (101 MHz, $CDCl_3$) inseparable 1:1 mixture with **S2**

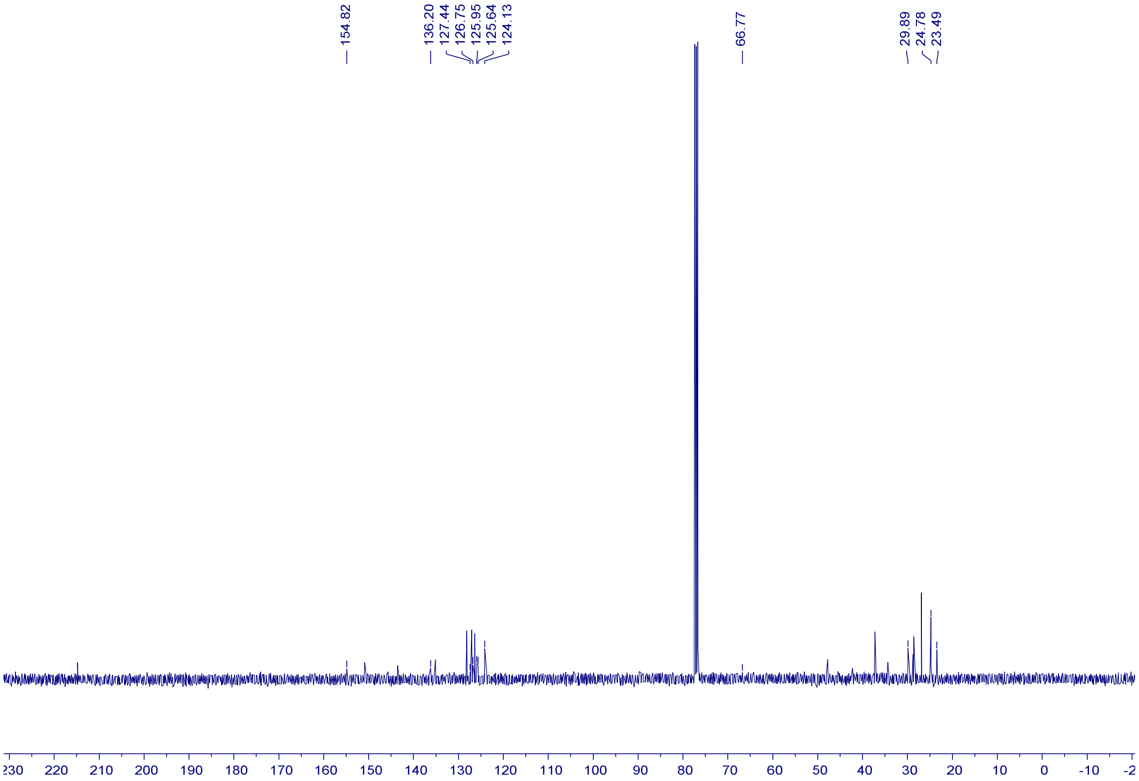

5c ^{1}H NMR (400 MHz, $CDCl_3$)

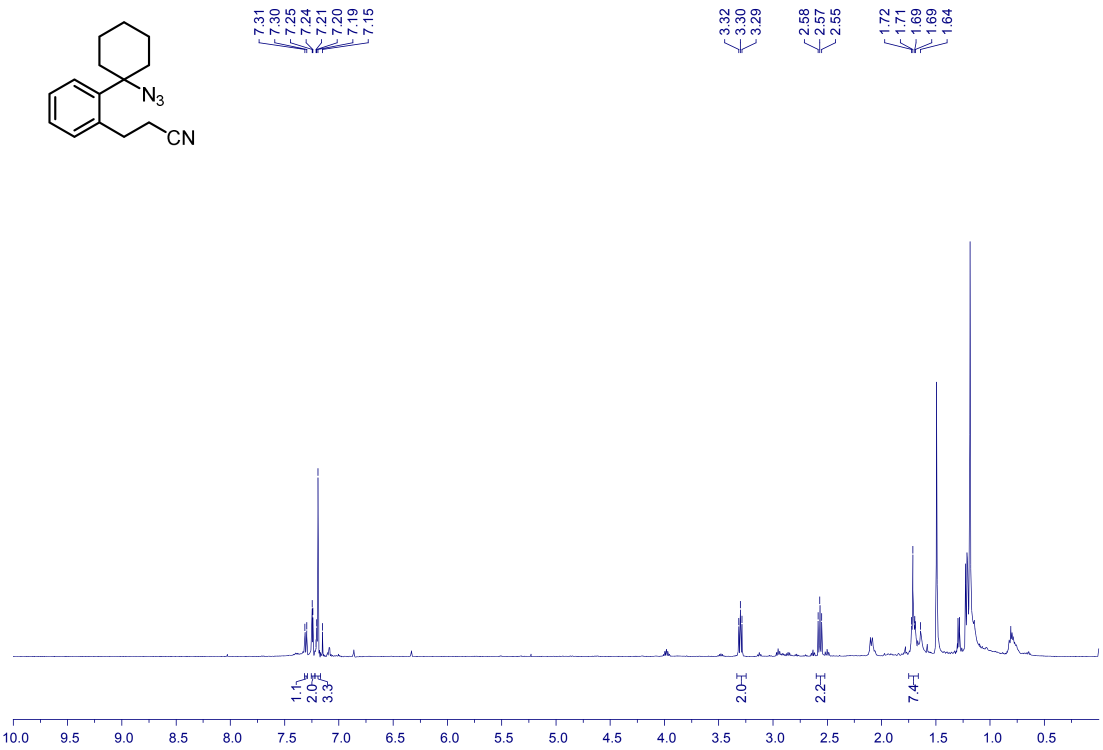

5c ^{13}C NMR (101 MHz, $CDCl_3$)

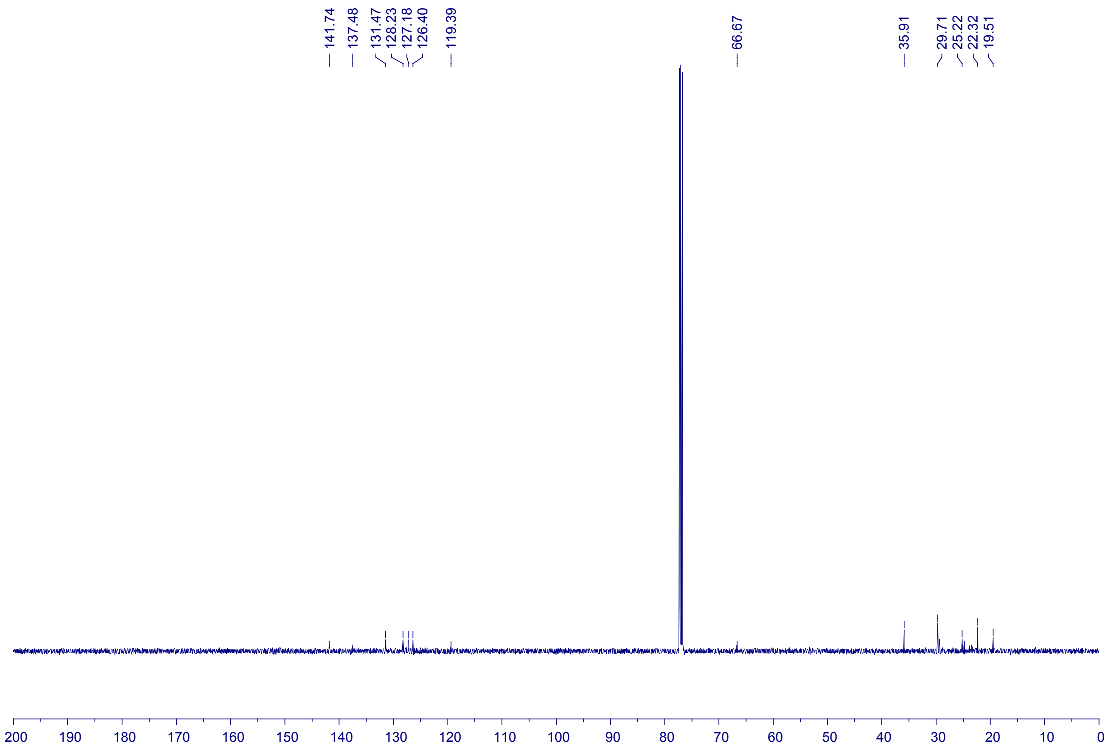

5d ^{1}H NMR (400 MHz, $CDCl_3$)

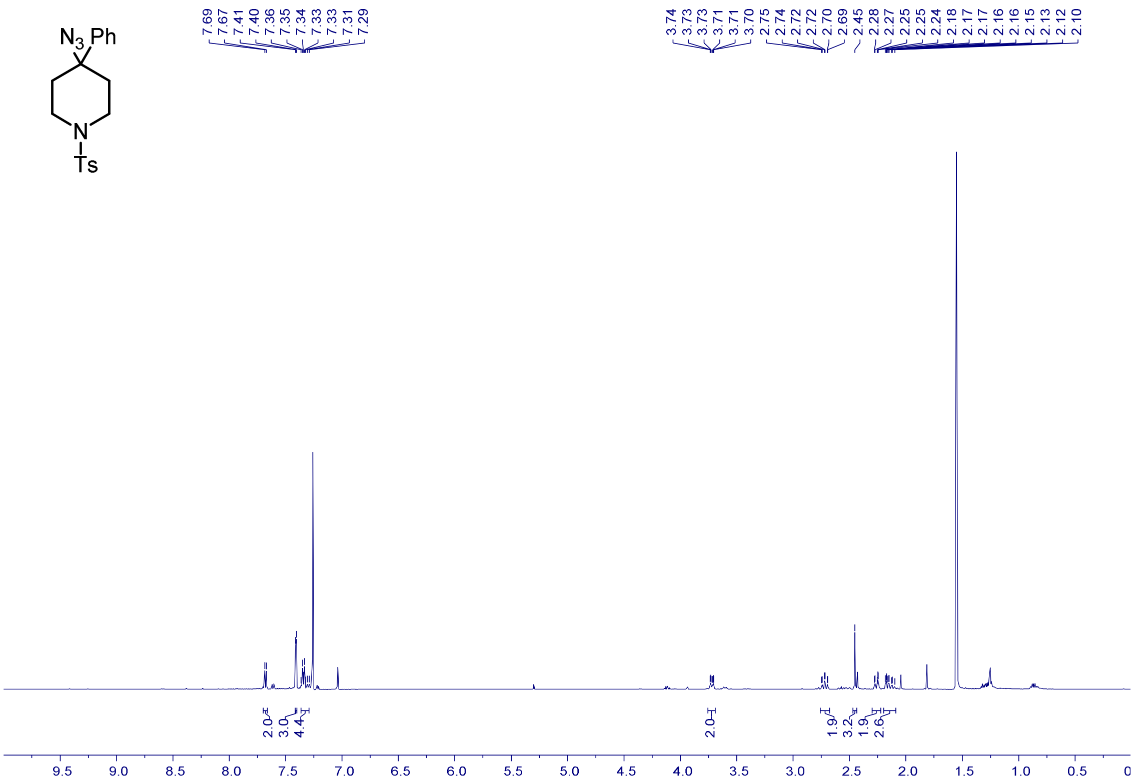

5d ^{13}C NMR (101 MHz, $CDCl_3$)

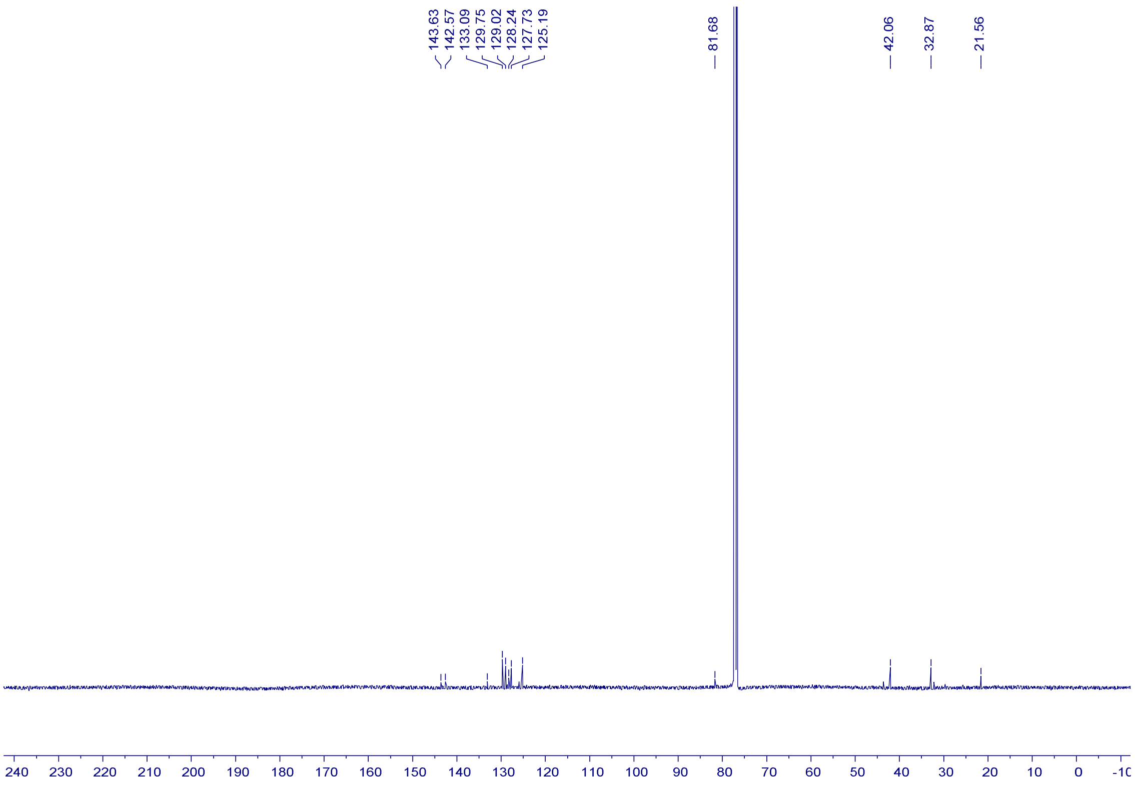

5e ^{1}H NMR (400 MHz, $CDCl_3$)

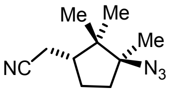

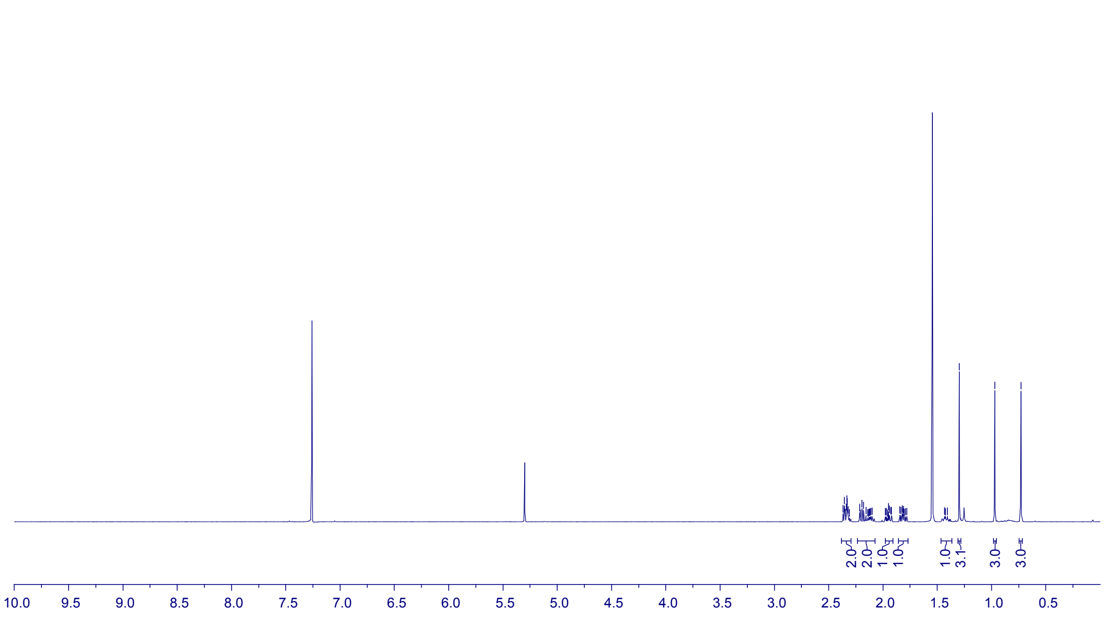

5e ^{13}C NMR (101 MHz, $CDCl_3$)

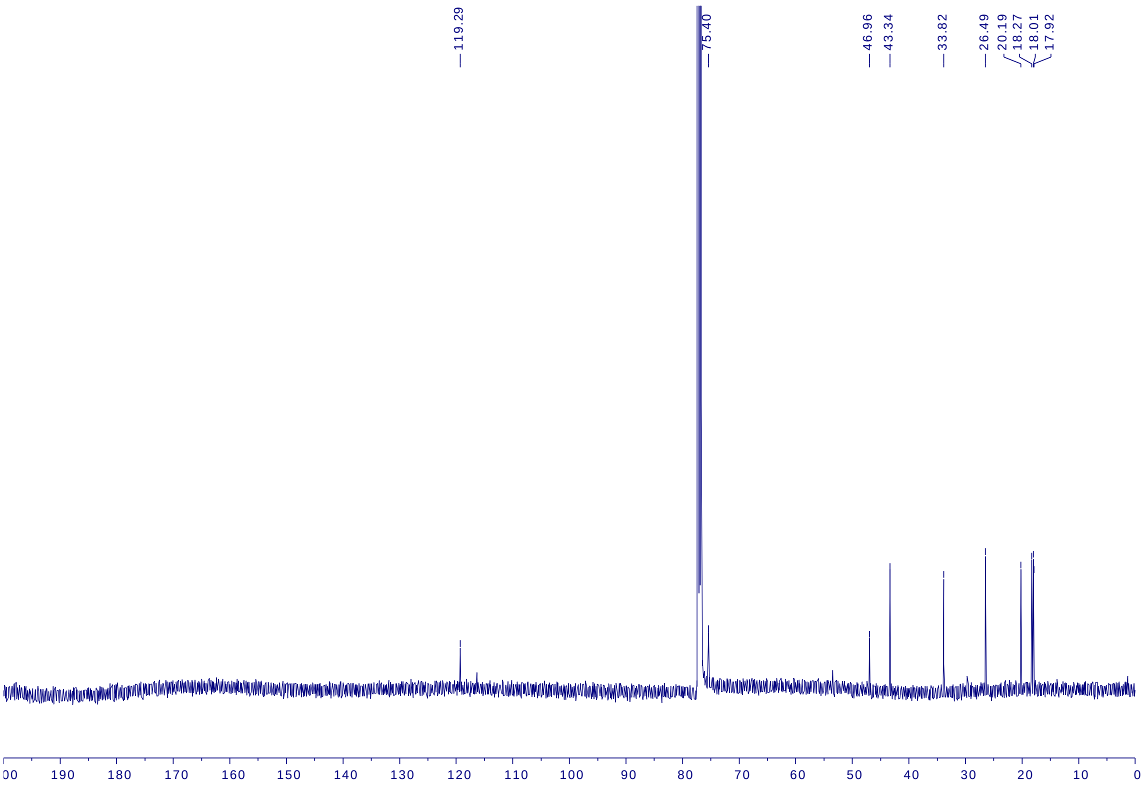

7a ^{1}H NMR (400 MHz, $CDCl_3$)

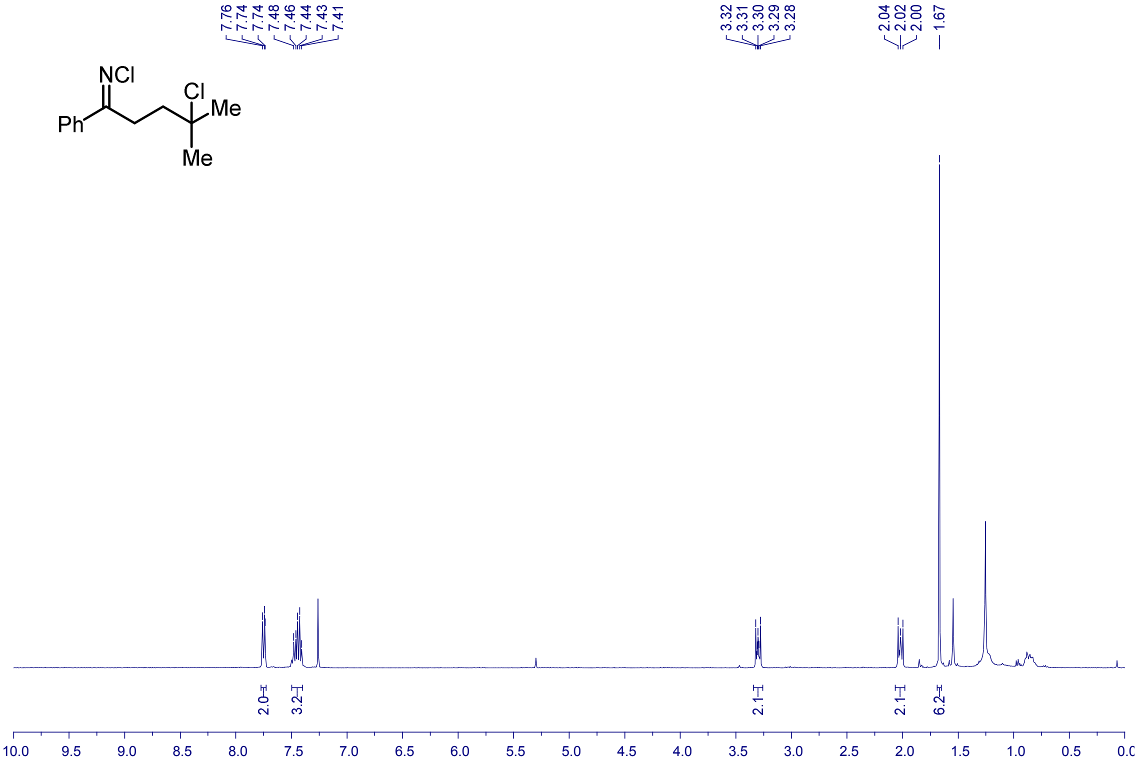

7a ^{13}C NMR (101 MHz, $CDCl_3$)

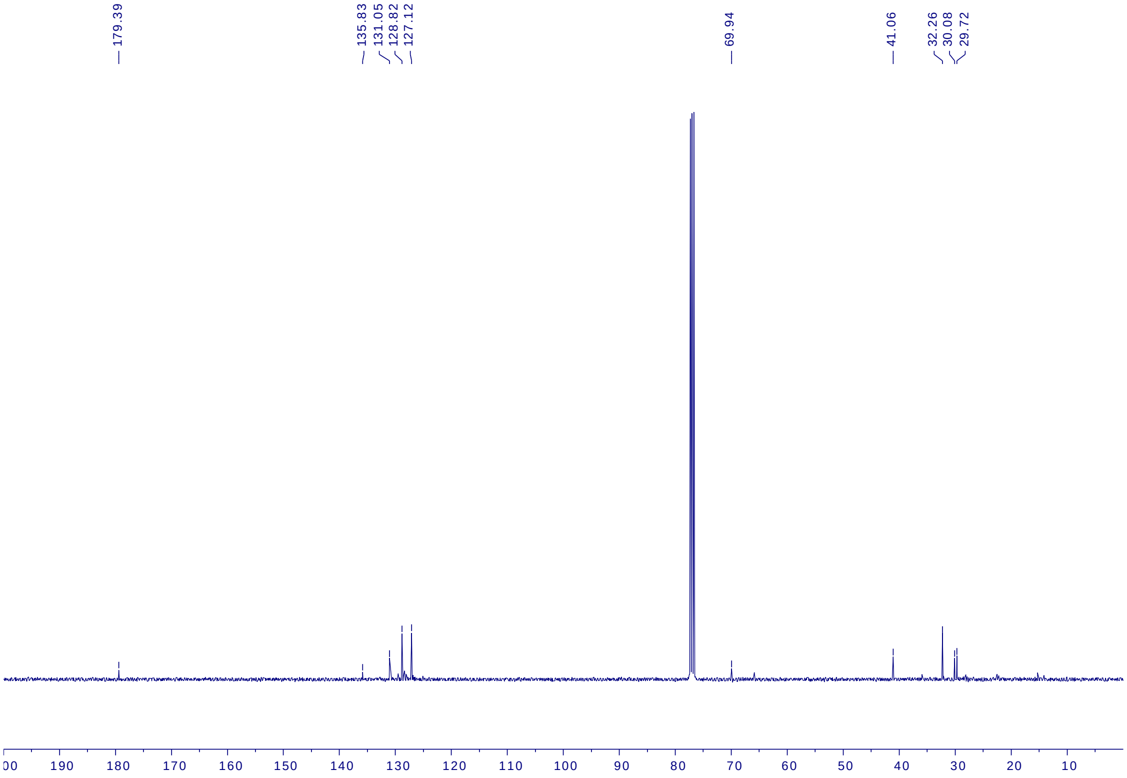

7b ^{1}H NMR (400 MHz, $CDCl_3$)

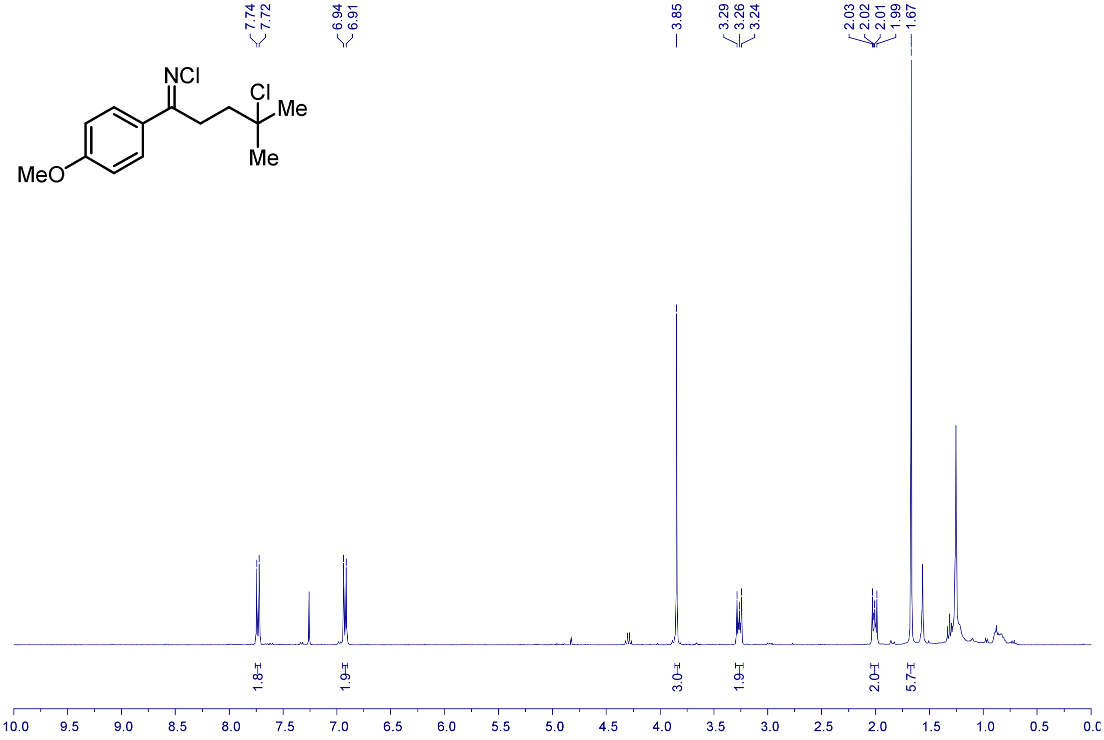

7b ^{13}C NMR (101 MHz, $CDCl_3$)

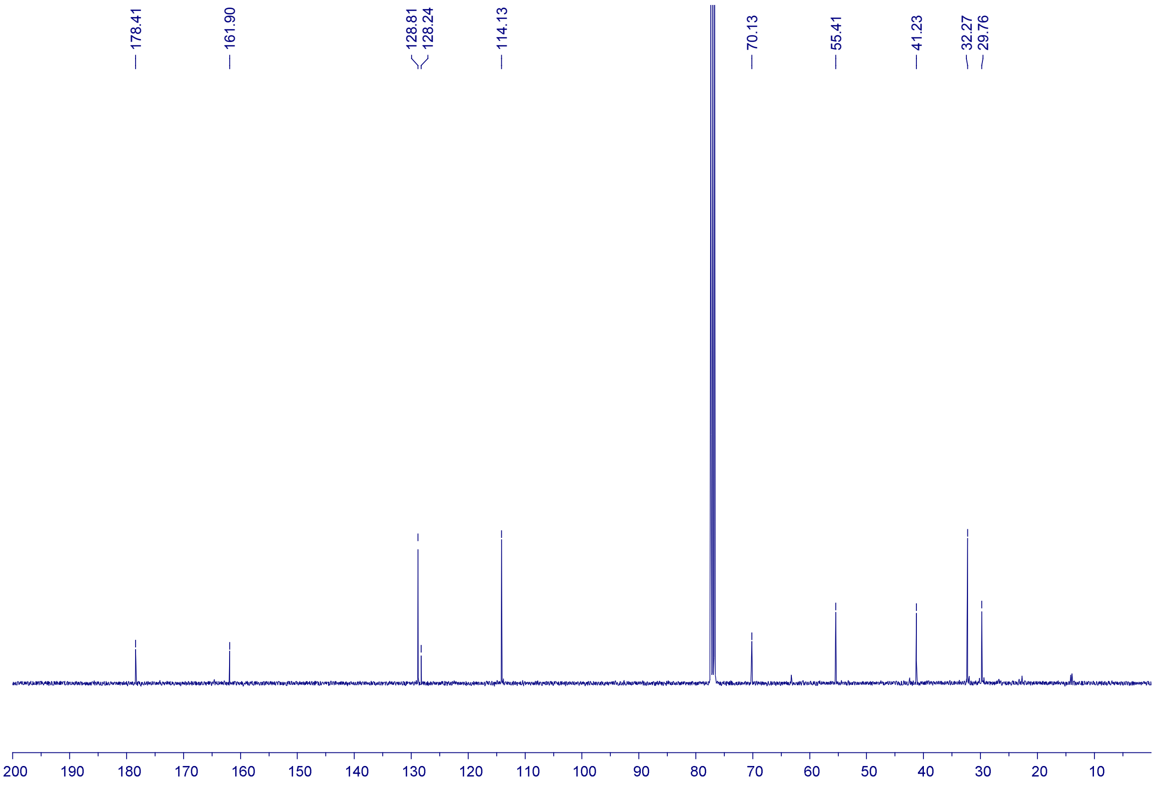

7c ^{1}H NMR (500 MHz, $CDCl_3$)

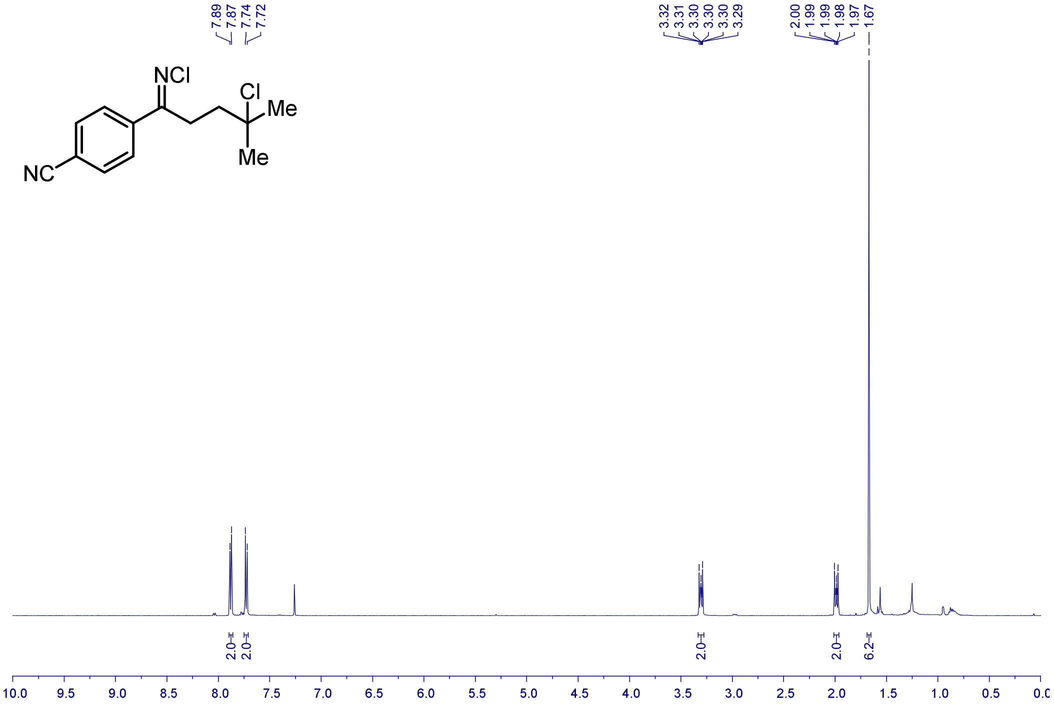

7c ^{13}C NMR (126 MHz, $CDCl_3$)

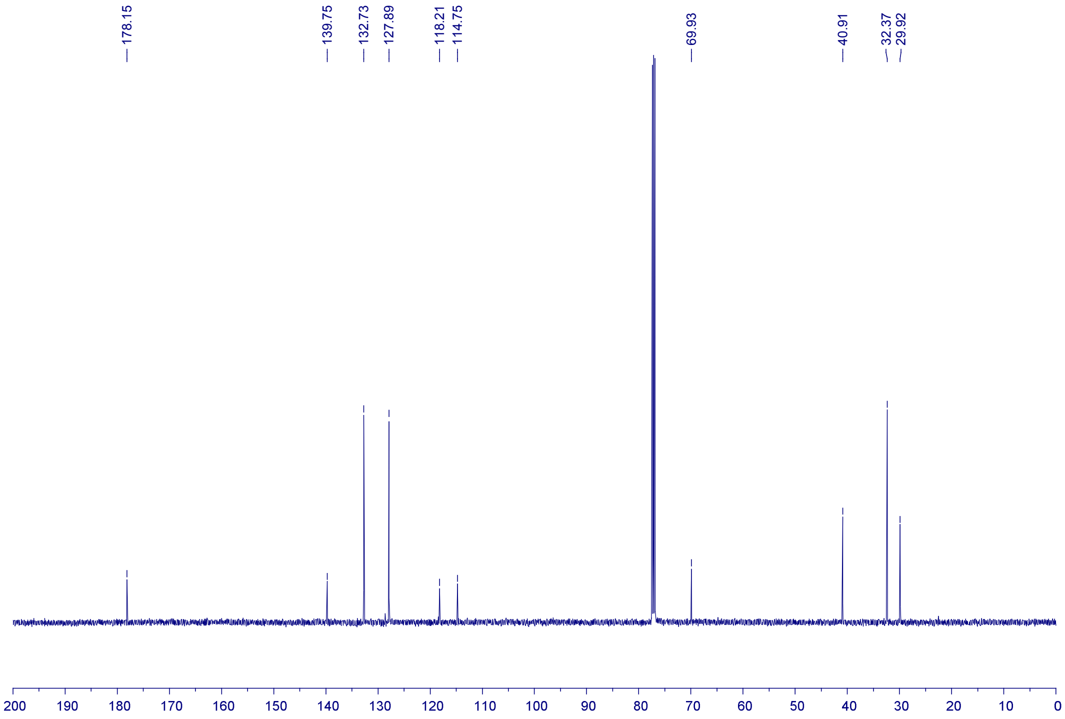

7d ^{1}H NMR (500 MHz, $CDCl_3$)

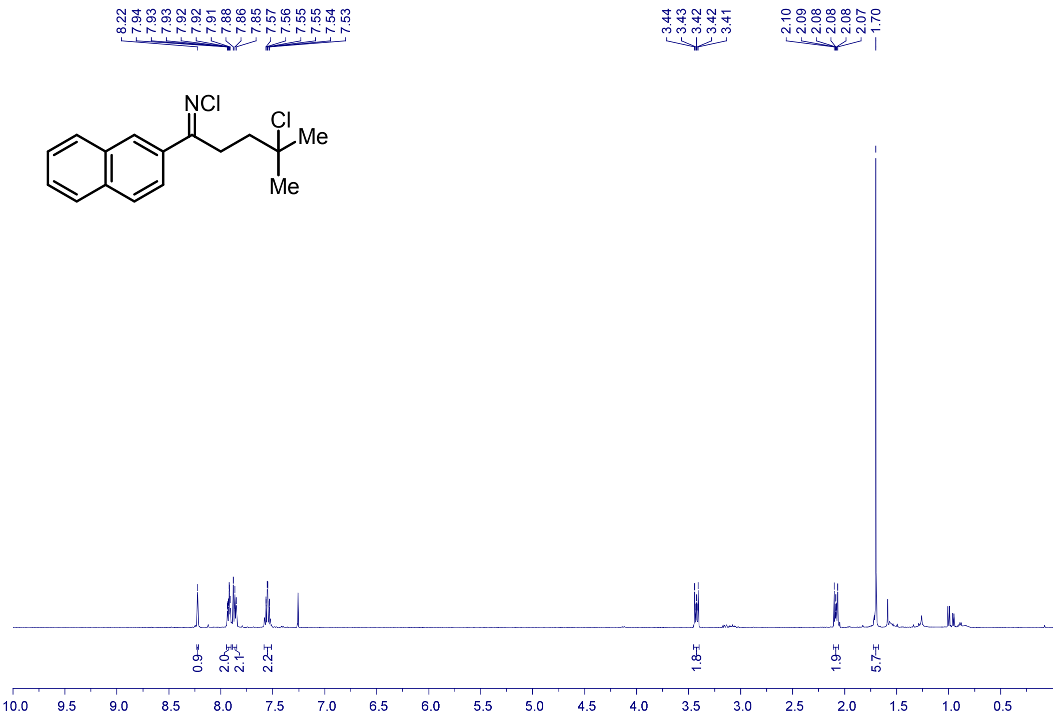

7d ^{13}C NMR (126 MHz, $CDCl_3$)

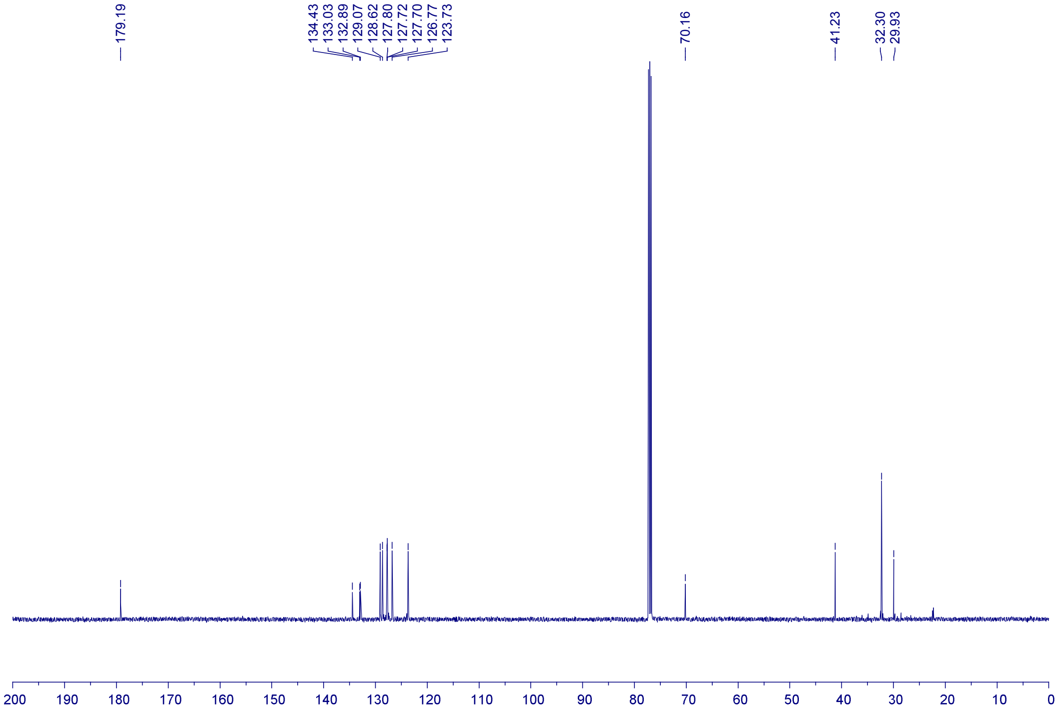

7e ^{1}H NMR (500 MHz, $CDCl_3$)

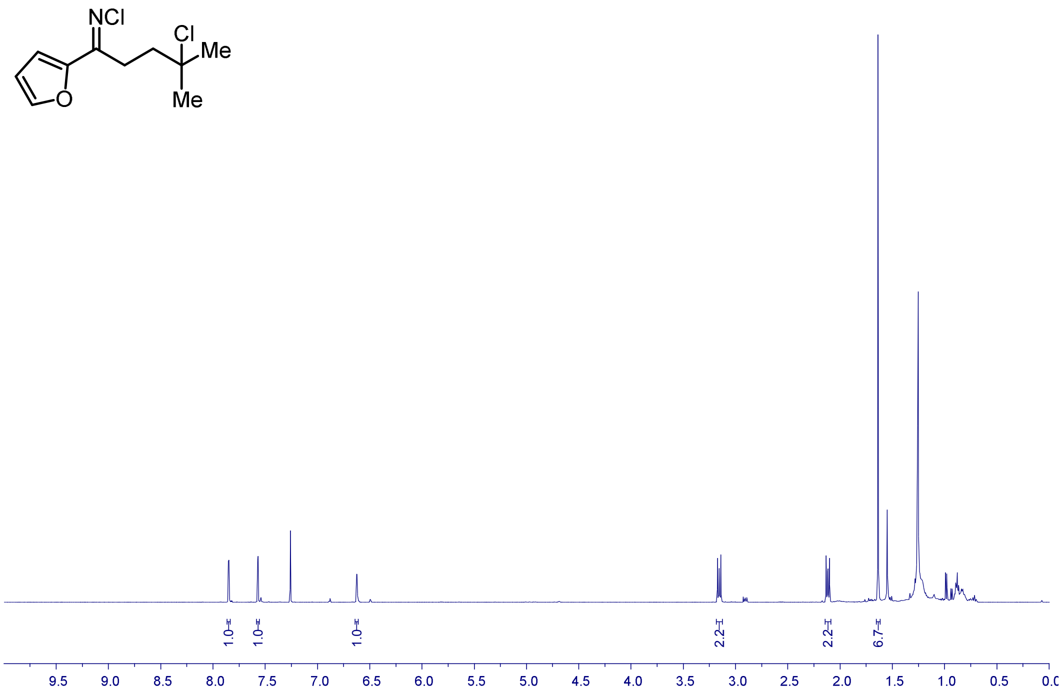

7e ^{13}C NMR (126 MHz, $CDCl_3$)

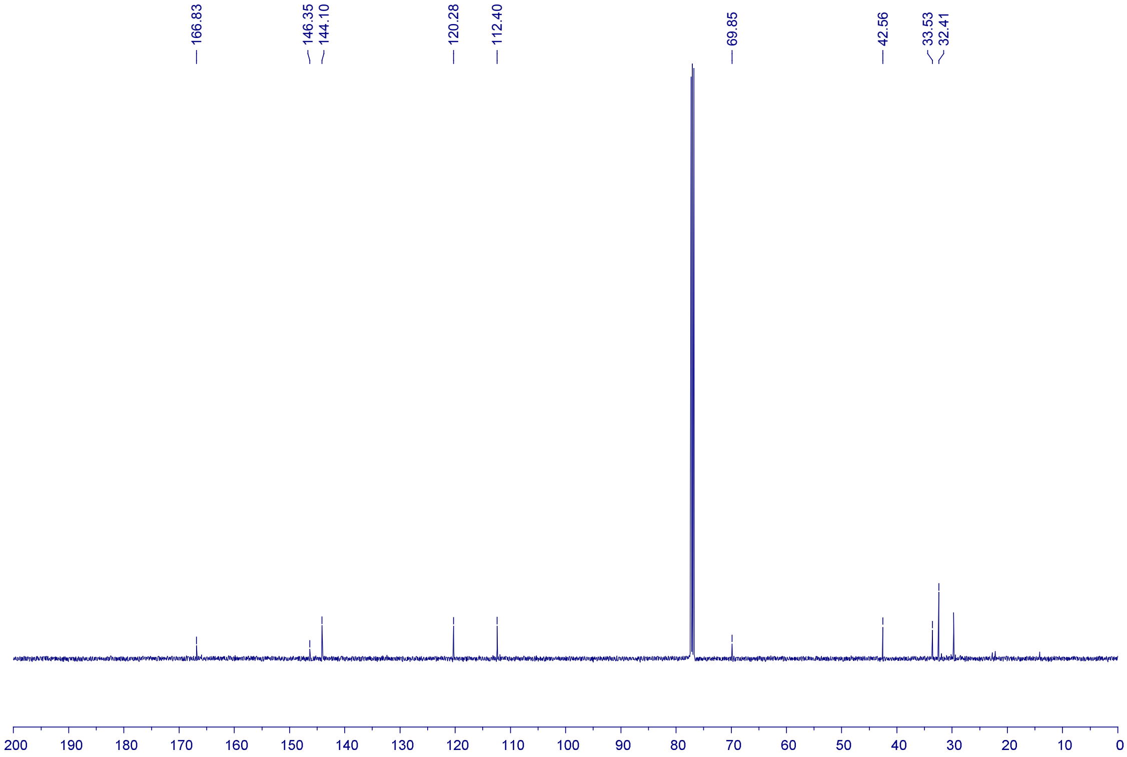

7f ^{1}H NMR (500 MHz, $CDCl_3$)

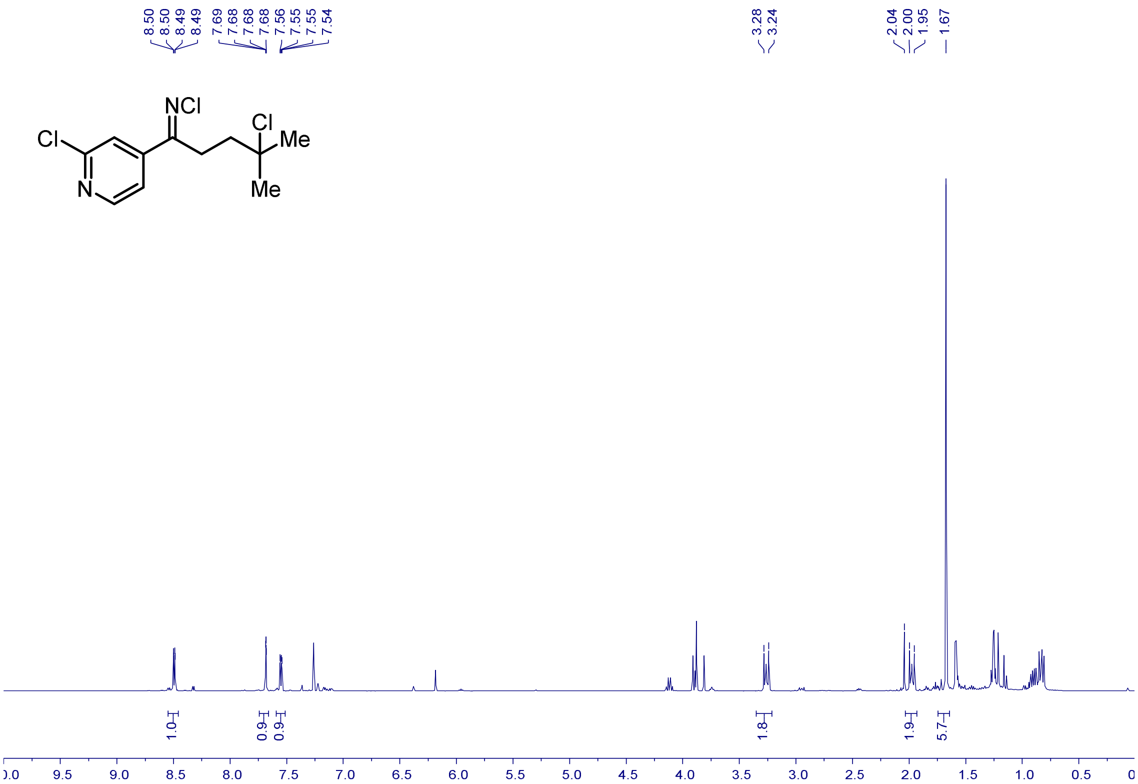

7f ^{13}C NMR (126 MHz, $CDCl_3$)

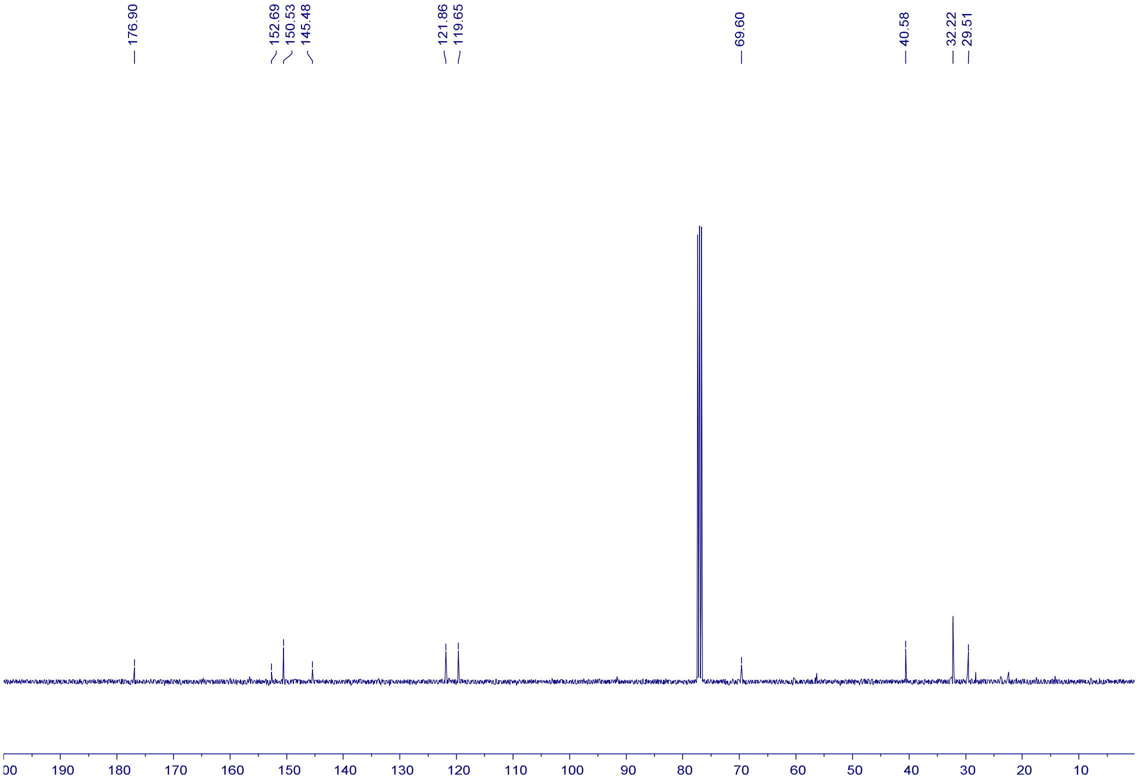

7g ^{1}H NMR (500 MHz, $CDCl_3$)

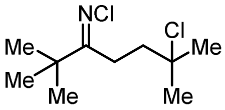

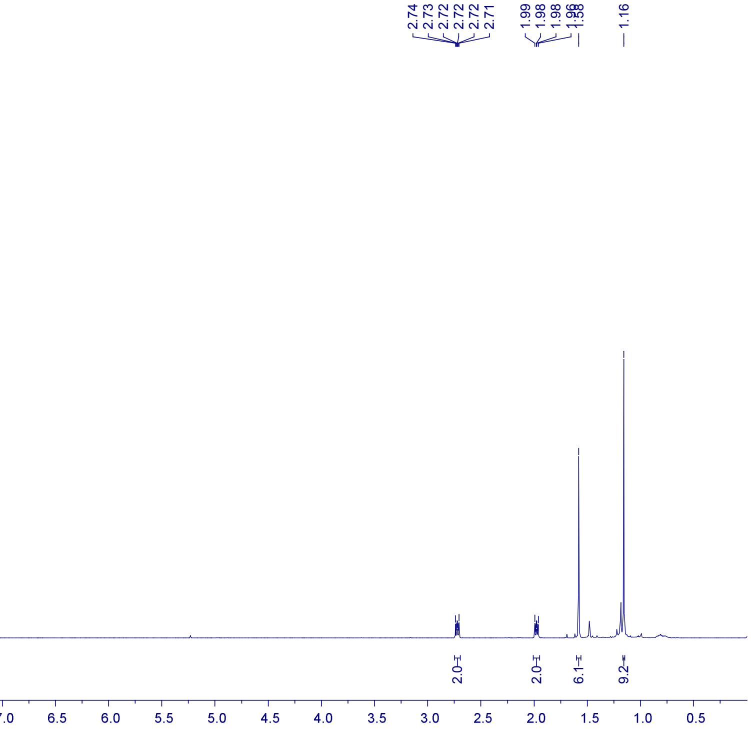

7g ^{13}C NMR (126 MHz, $CDCl_3$)

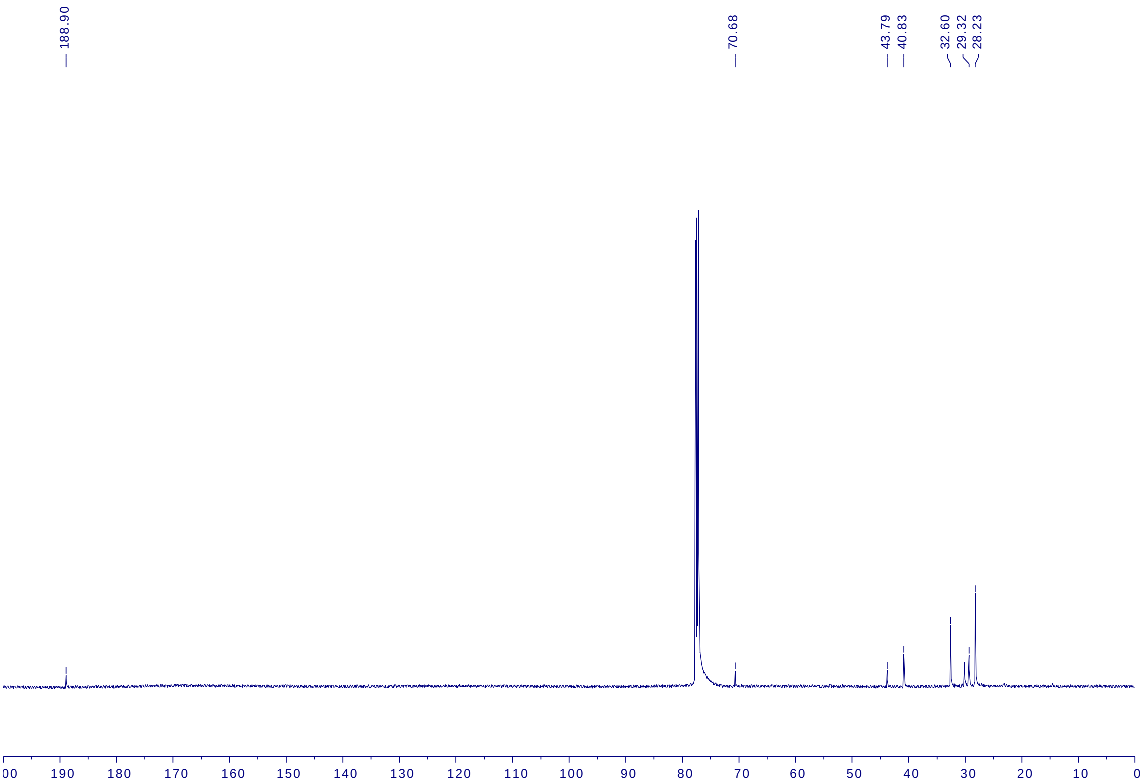

7h ^{1}H NMR (500 MHz, $CDCl_3$)

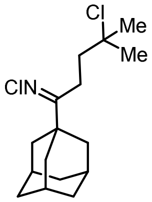

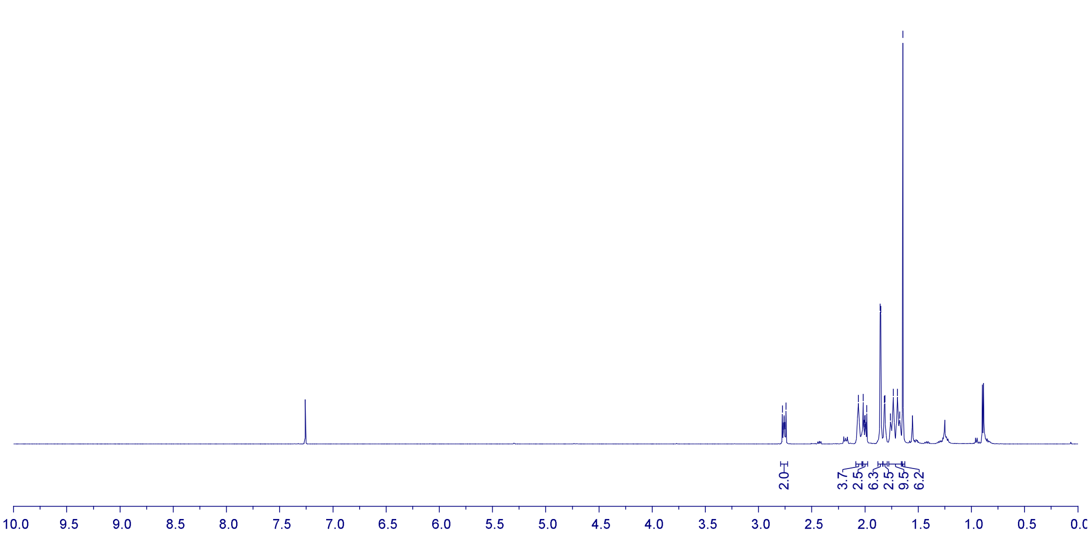

7h ^{13}C NMR (126 MHz, $CDCl_3$)

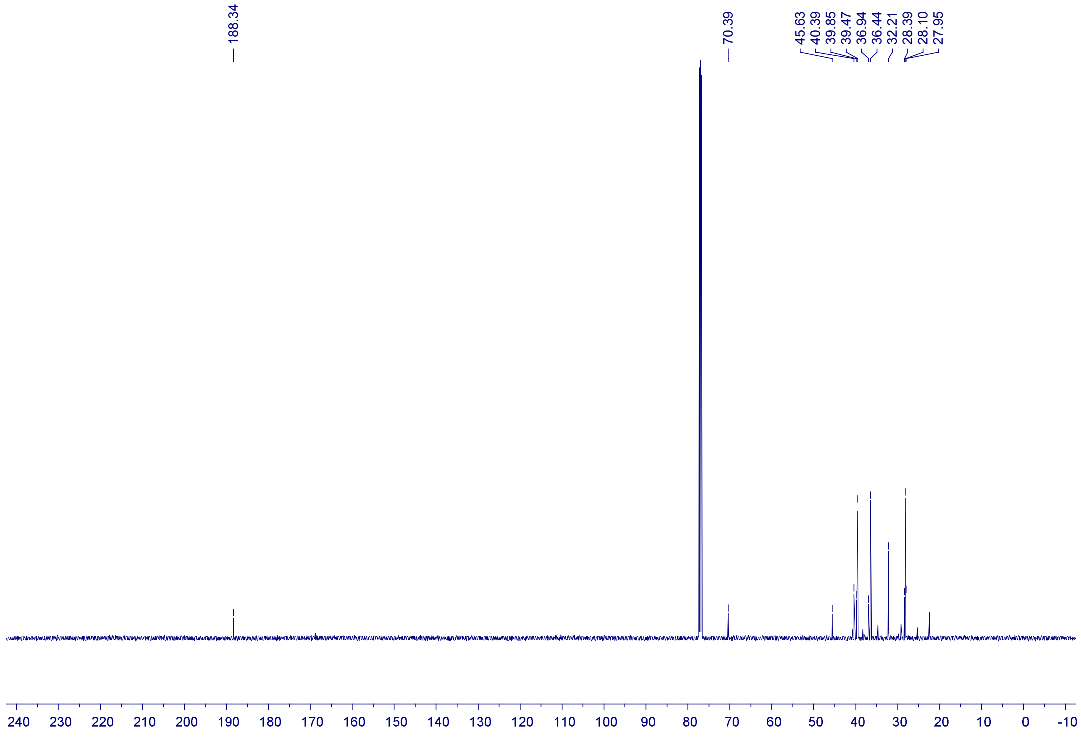

7i ^{1}H NMR (400 MHz, $CDCl_3$)

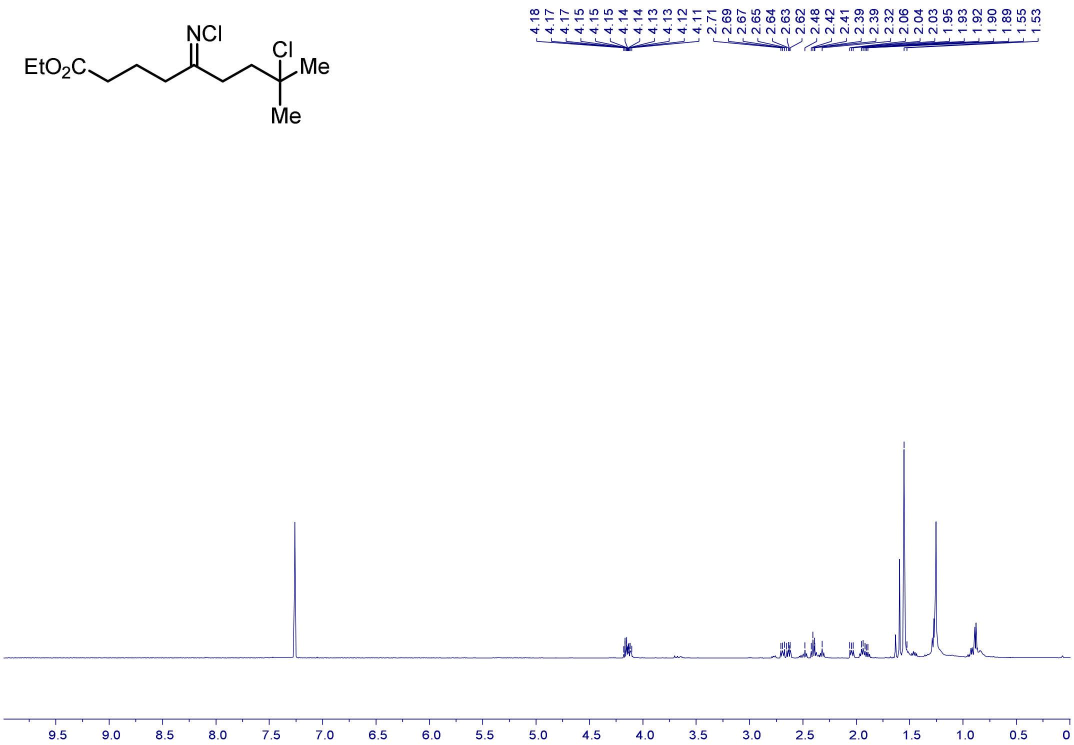

7i ^{13}C NMR (126 MHz, $CDCl_3$)

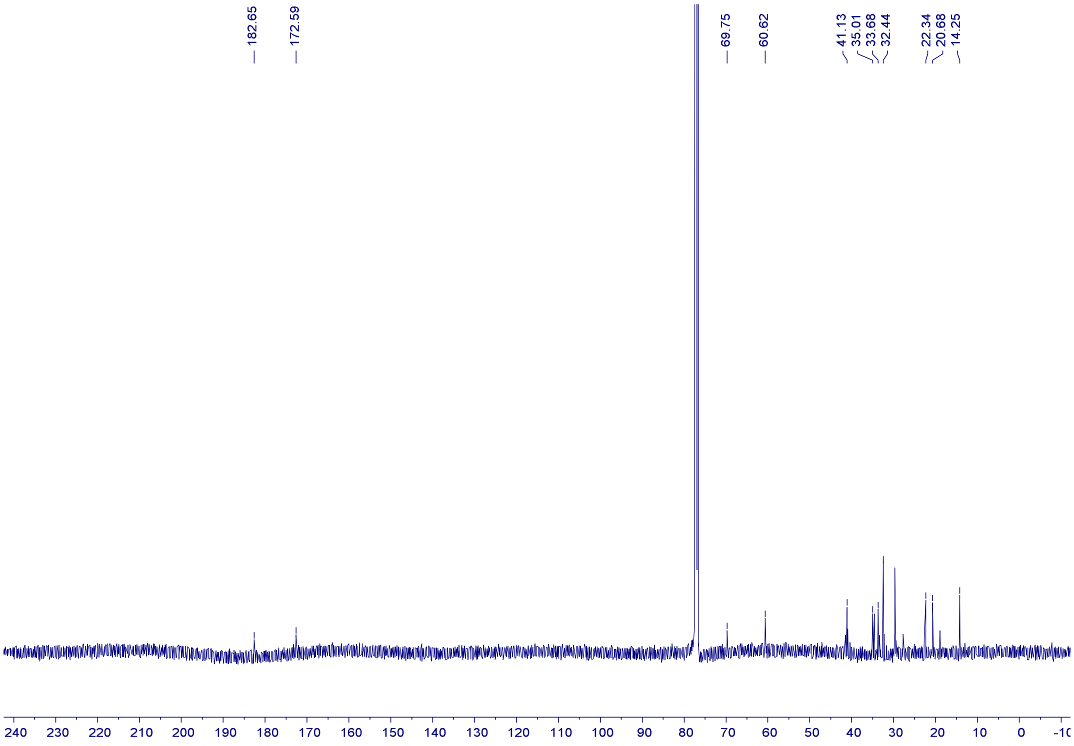

7j ^{1}H NMR (500 MHz, $CDCl_3$)

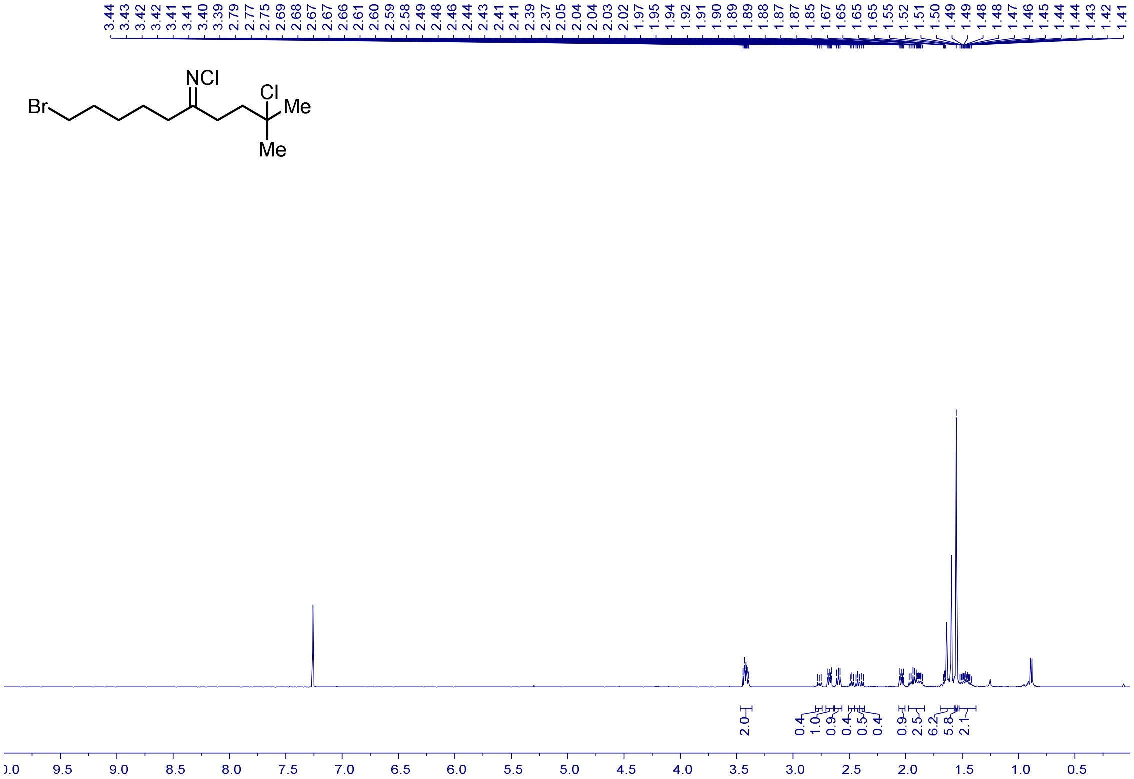

7j ^{13}C NMR (126 MHz, $CDCl_3$)

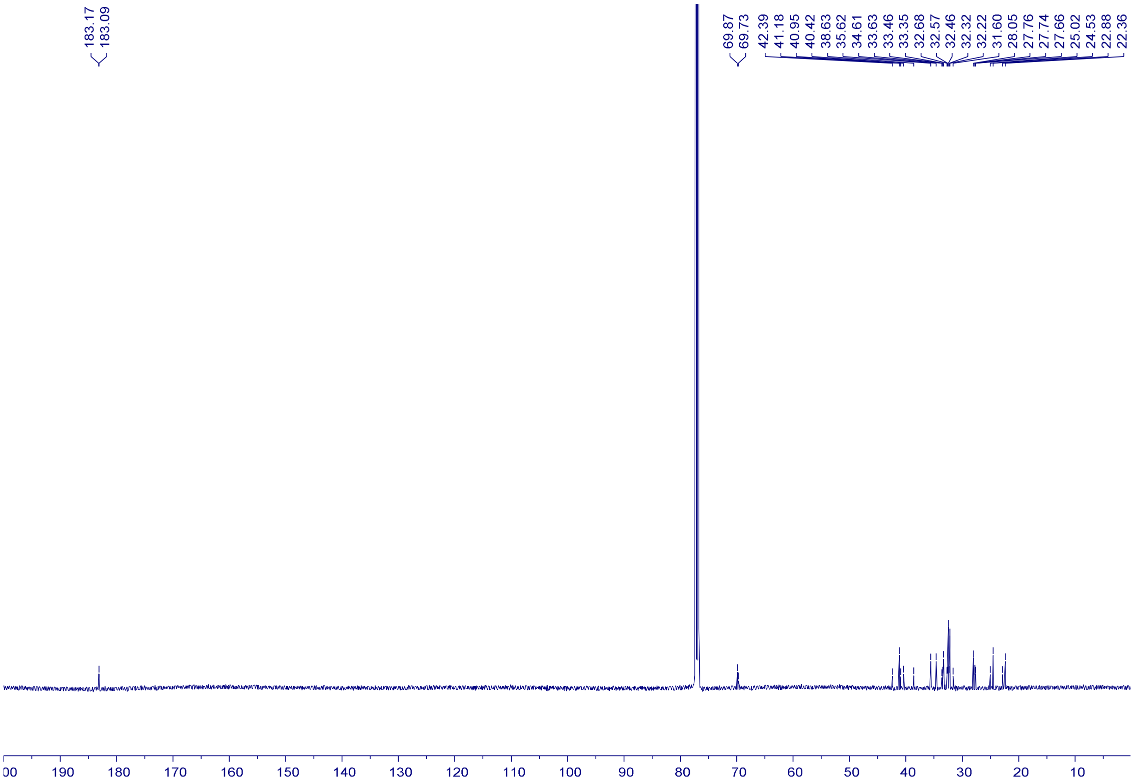

7k ^{1}H NMR (500 MHz, $CDCl_3$)

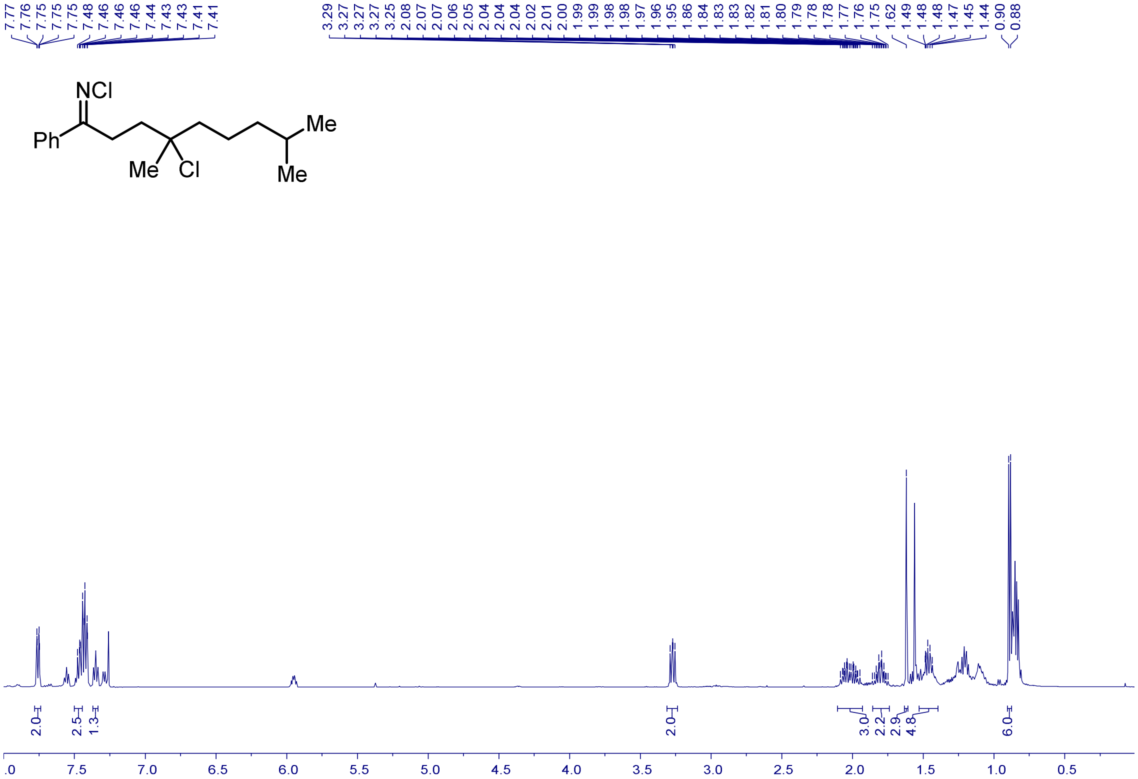

7k ^{13}C NMR (126 MHz, $CDCl_3$)

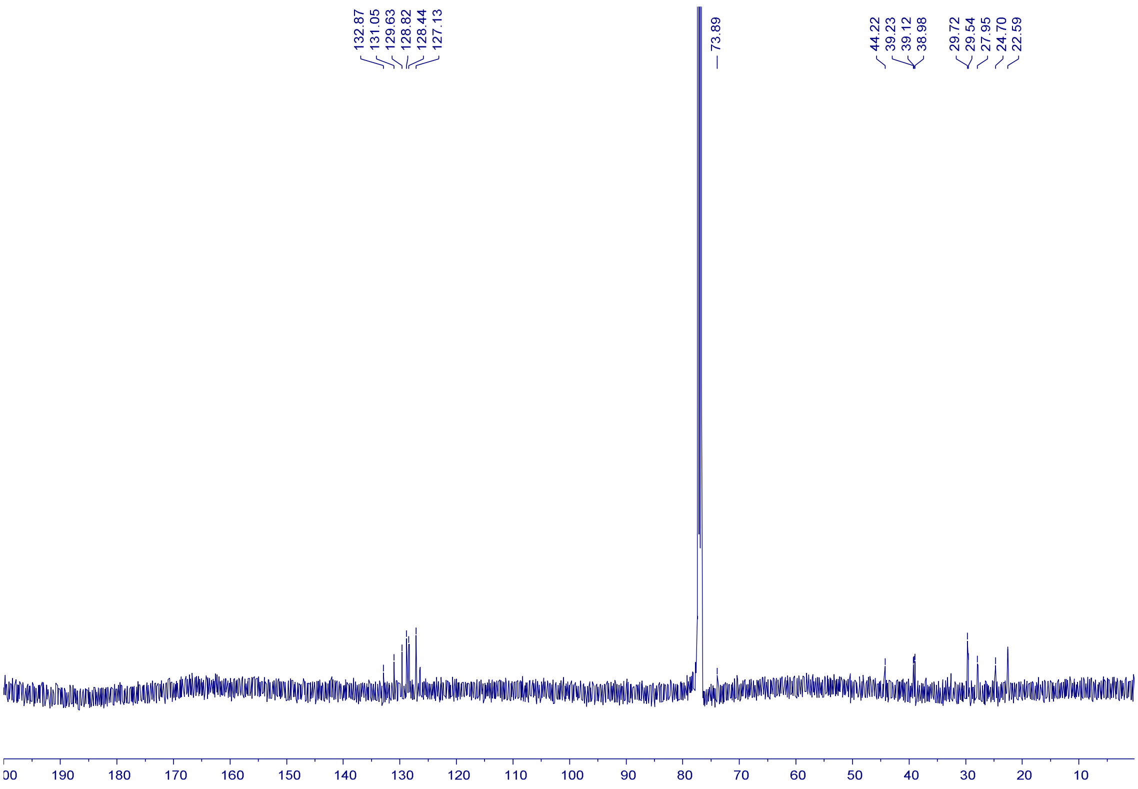

8b ^{1}H NMR (400 MHz, $CDCl_3$)

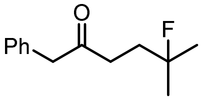

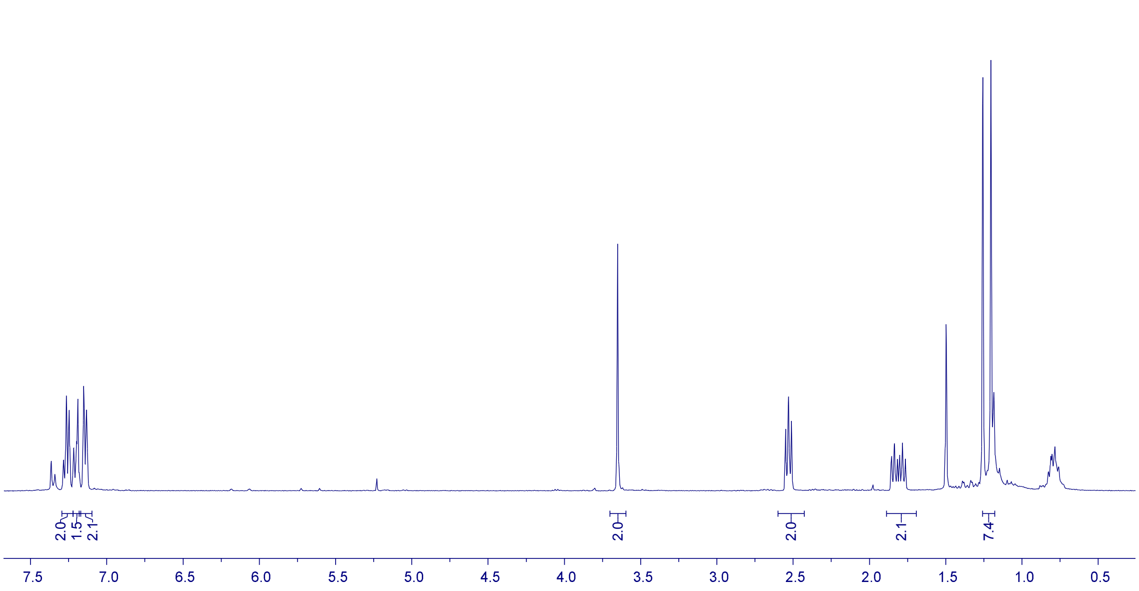

8b ^{13}C NMR (101 MHz, $CDCl_3$)

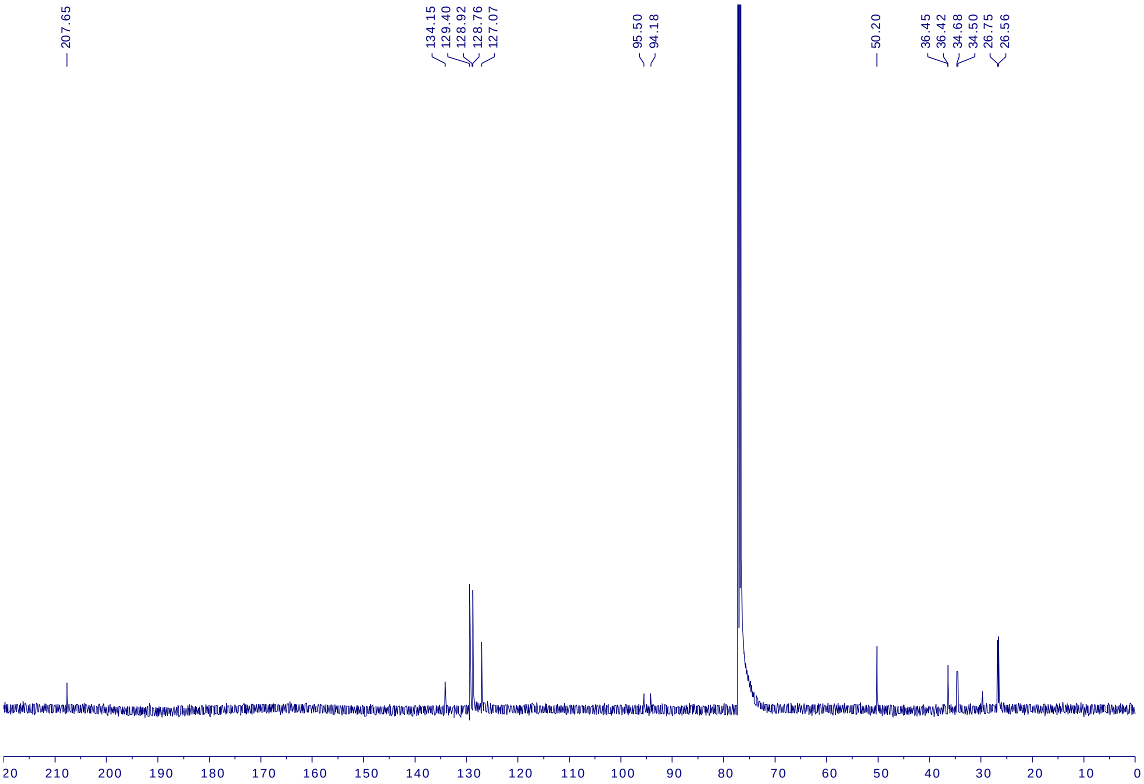

8b ^{19}F NMR (376 MHz, $CDCl_3$)

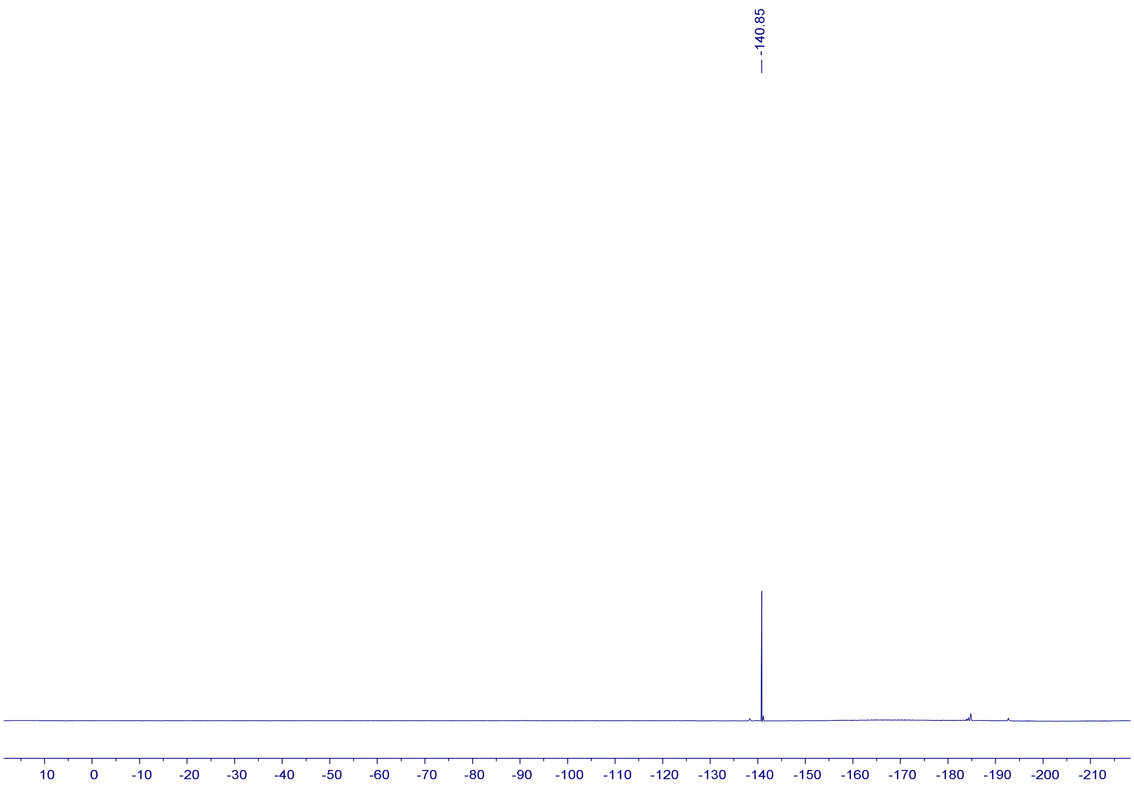

8c ^{1}H NMR (500 MHz, $CDCl_3$)

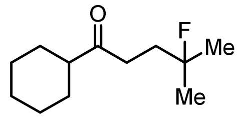

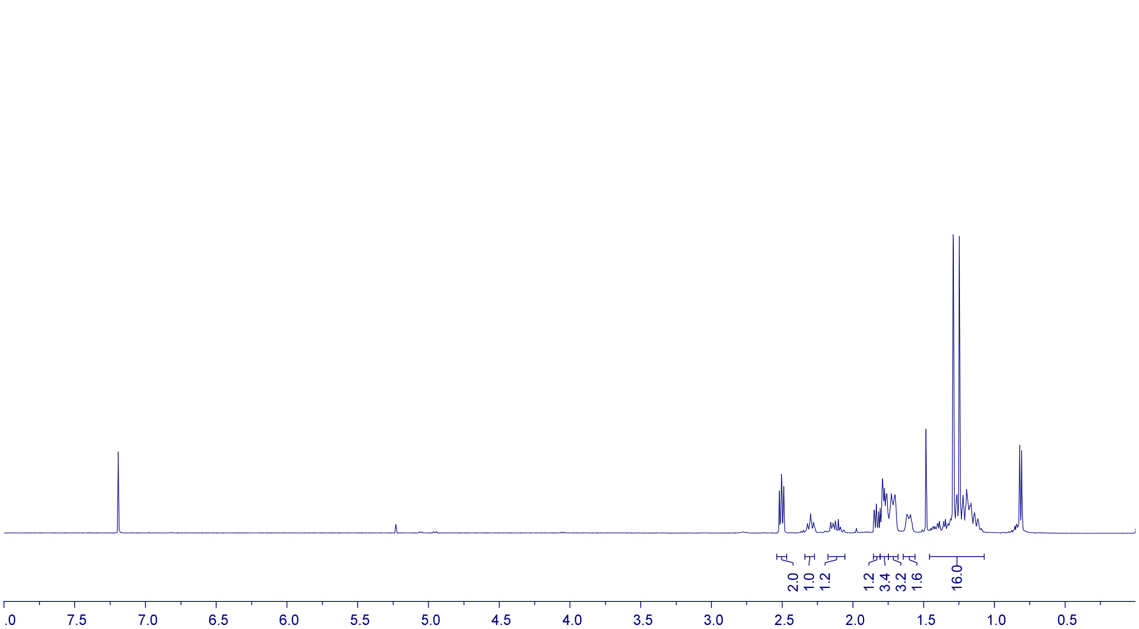

8c ^{13}C NMR (126 MHz, $CDCl_3$)

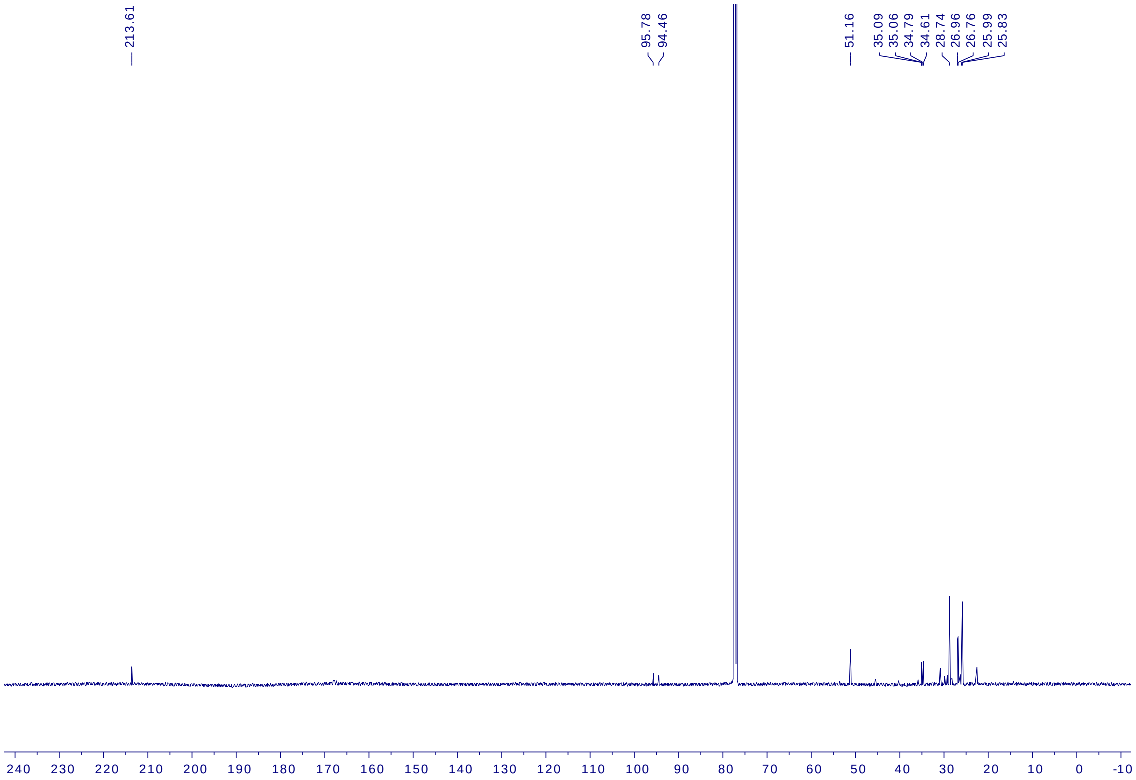

8c ^{19}F NMR (376 MHz, $CDCl_3$)

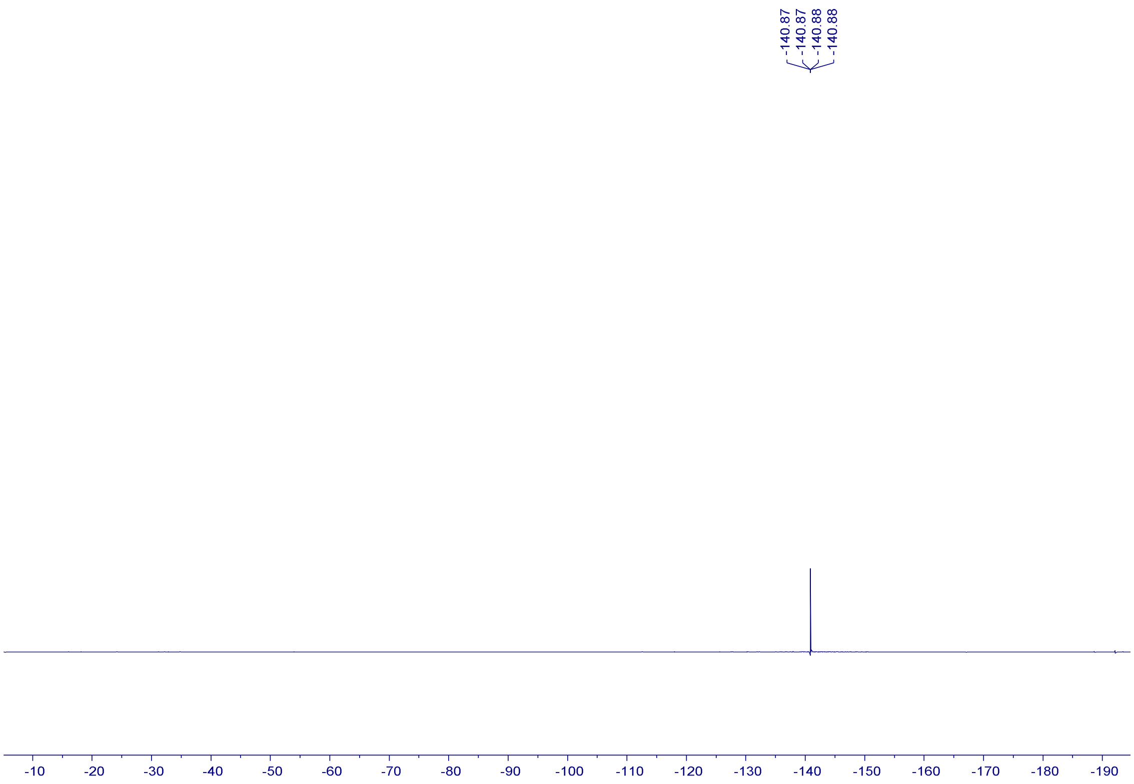

8d ^{1}H NMR (500 MHz, $CDCl_3$)

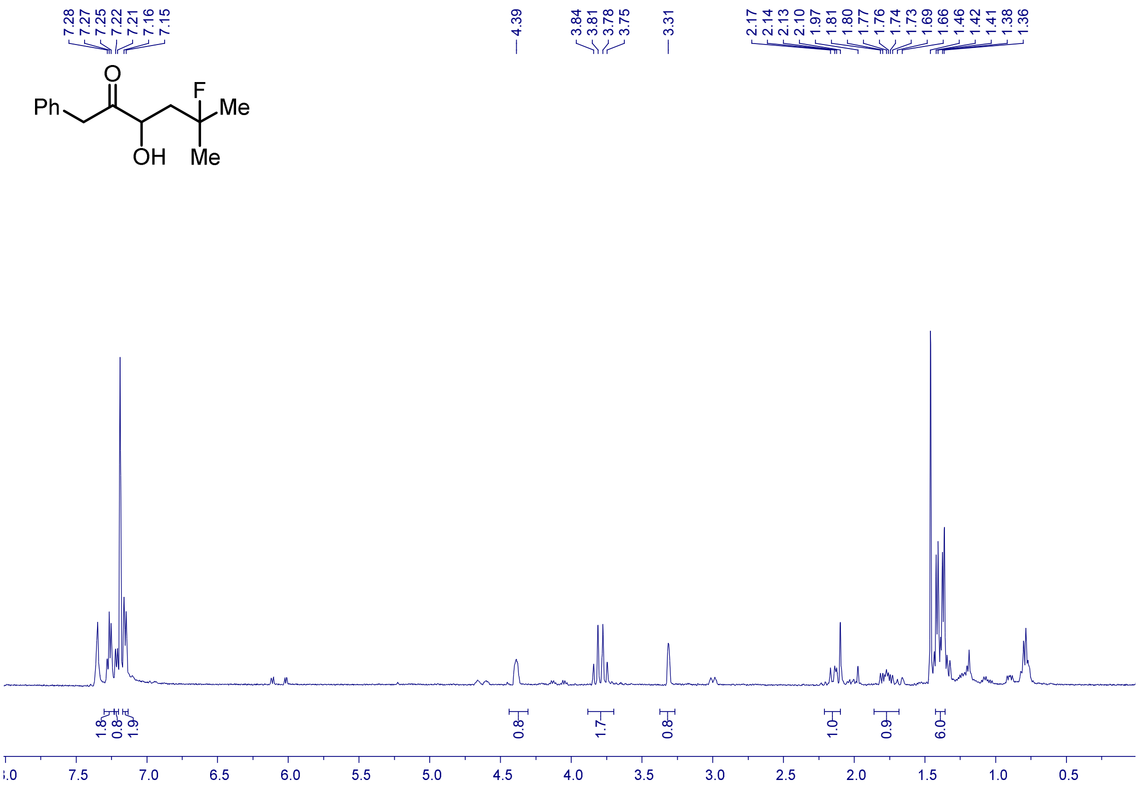

8d ^{13}C NMR (126 MHz, $CDCl_3$)

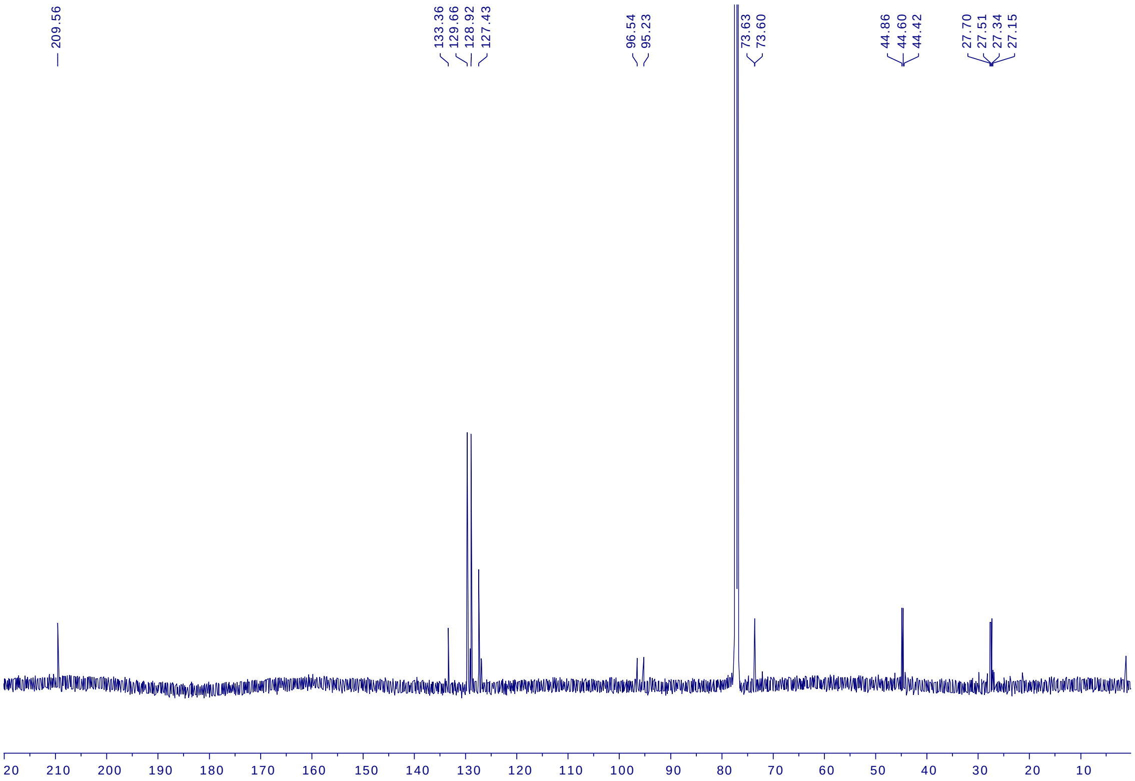

8d ^{19}F NMR (376 MHz, $CDCl_3$)

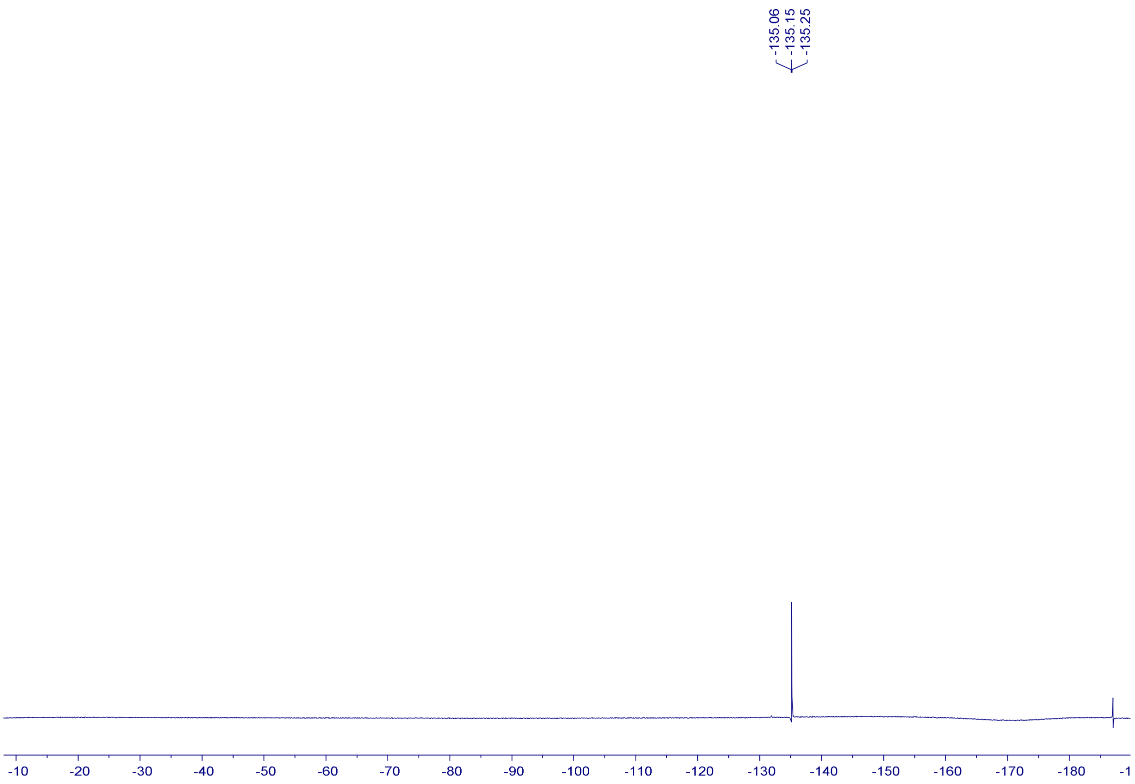

8e ^{1}H NMR (400 MHz, $CDCl_3$)

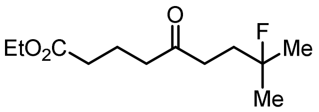

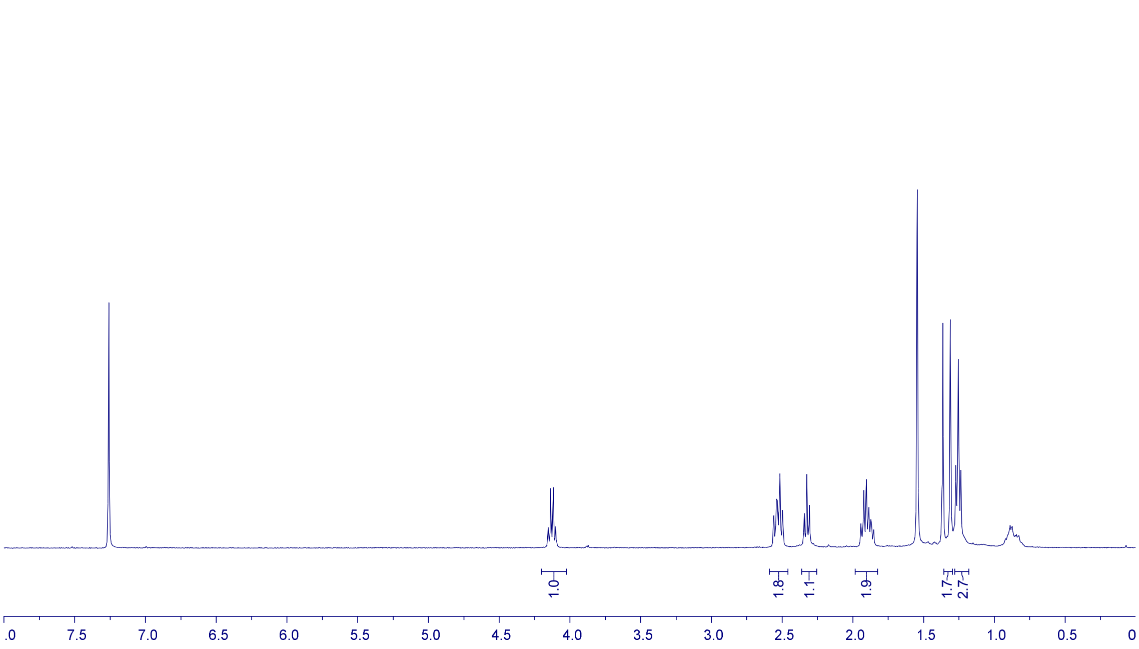

8e ^{13}C NMR (126 MHz, $CDCl_3$)

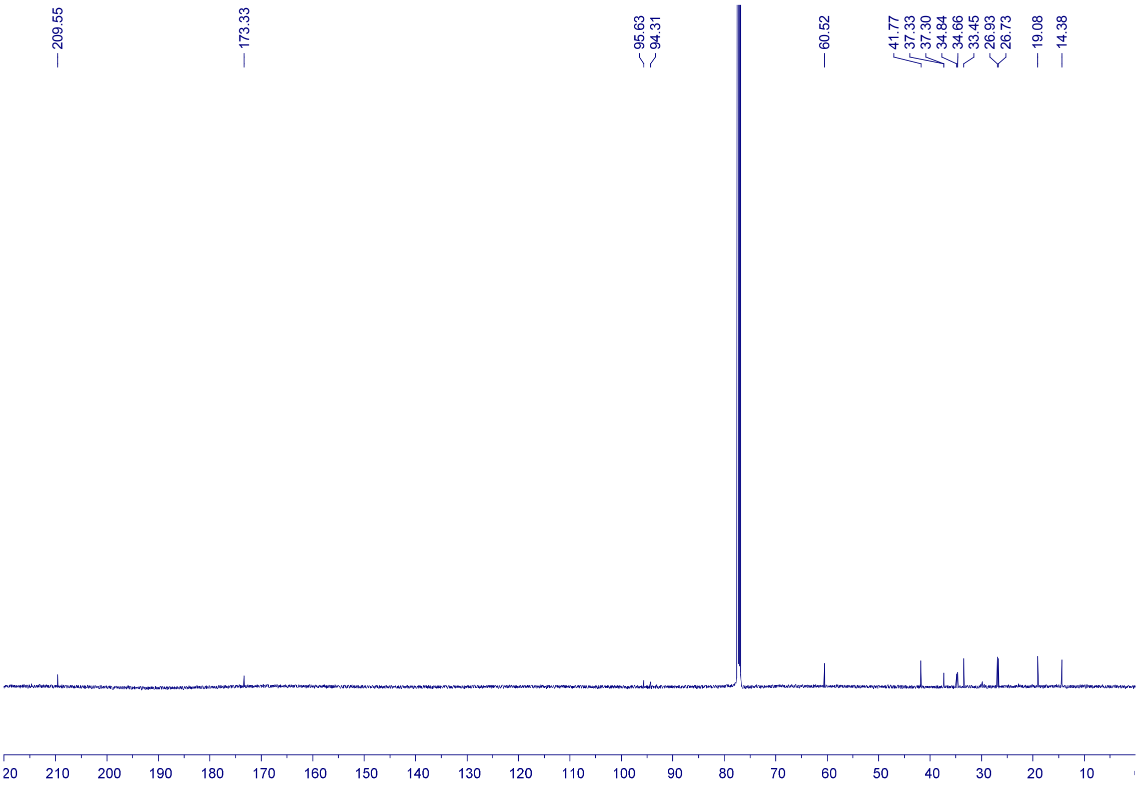

8e ^{19}F NMR (376 MHz, $CDCl_3$)

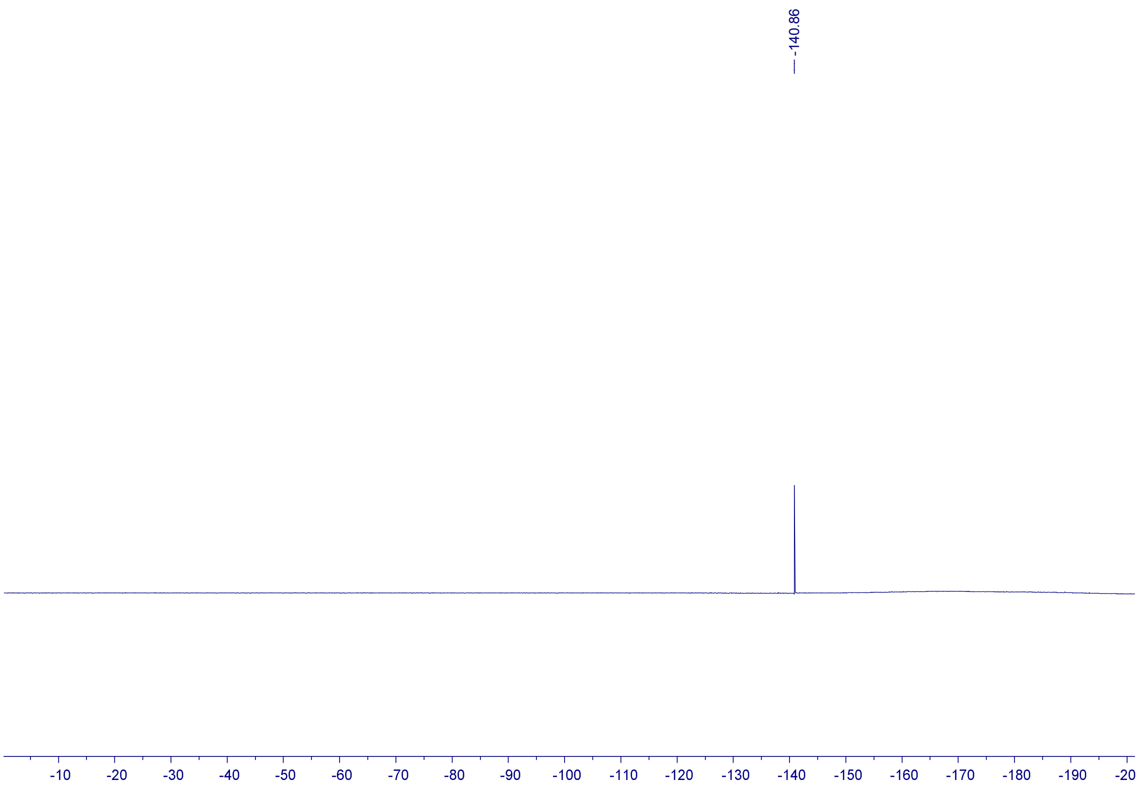

8f ^{1}H NMR (500 MHz, $CDCl_3$)

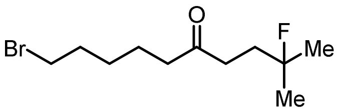

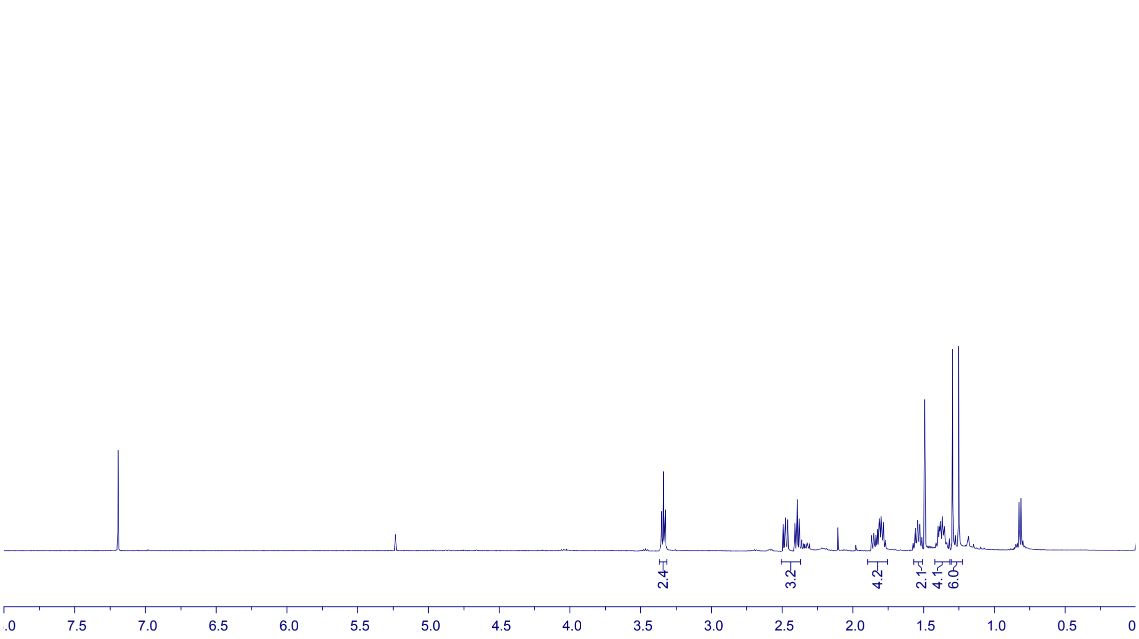

8f ^{13}C NMR (126 MHz, $CDCl_3$)

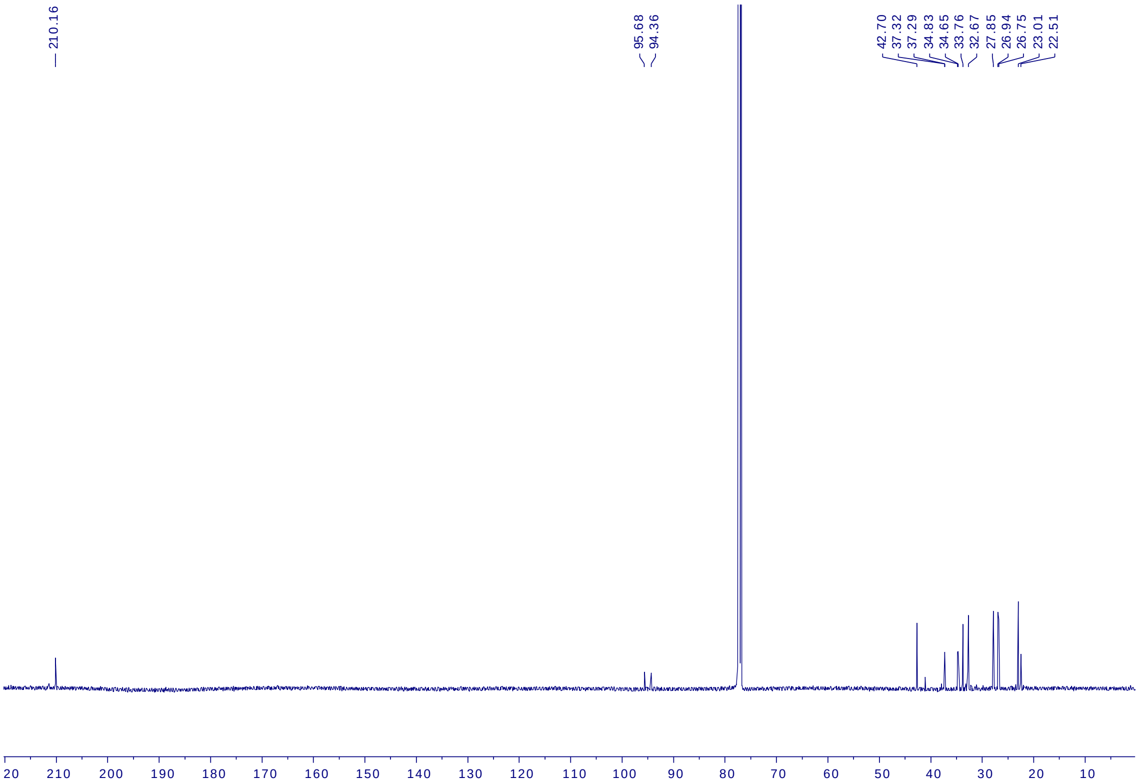

8f ^{19}F NMR (376 MHz, $CDCl_3$)

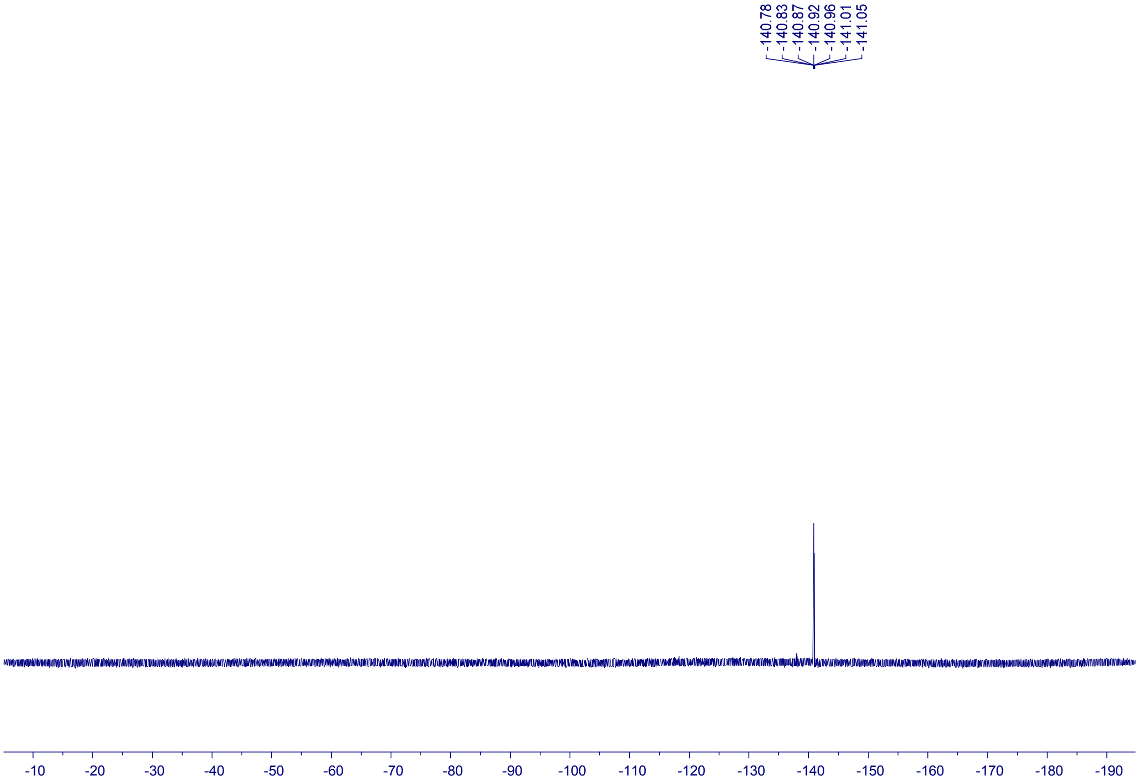

8g ^{1}H NMR (400 MHz, $CDCl_3$)

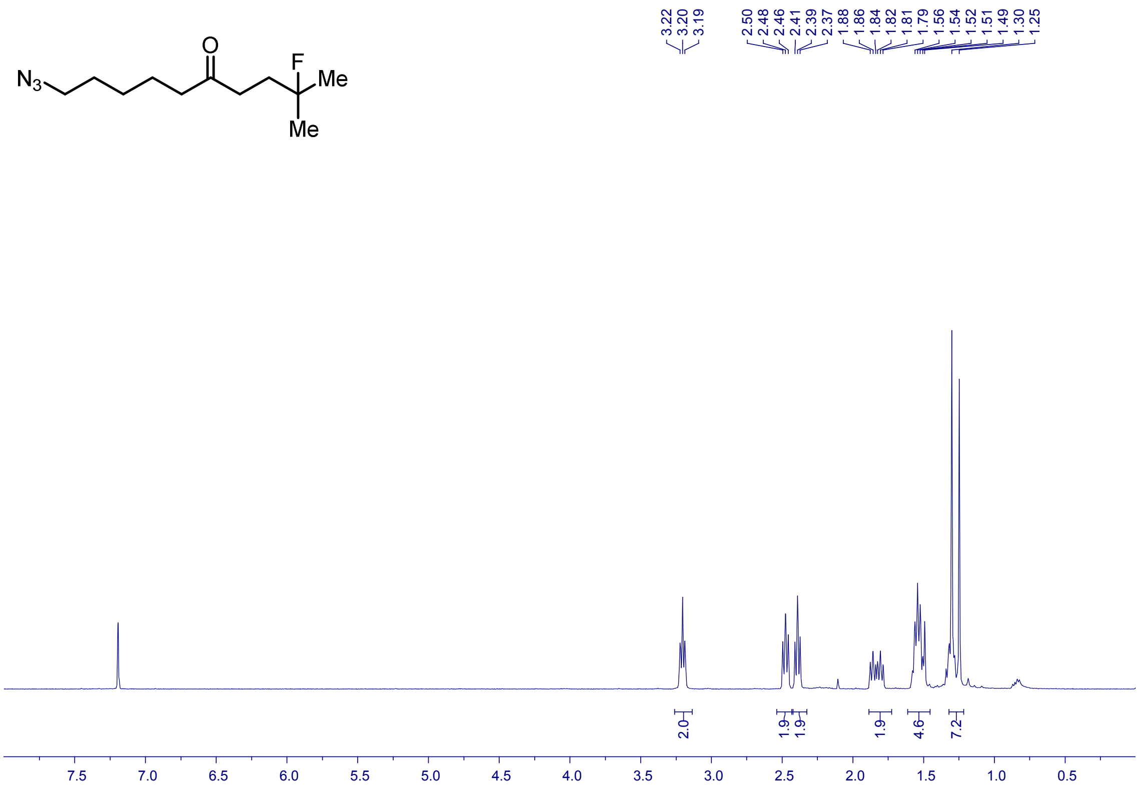

8g ^{13}C NMR (126 MHz, $CDCl_3$)

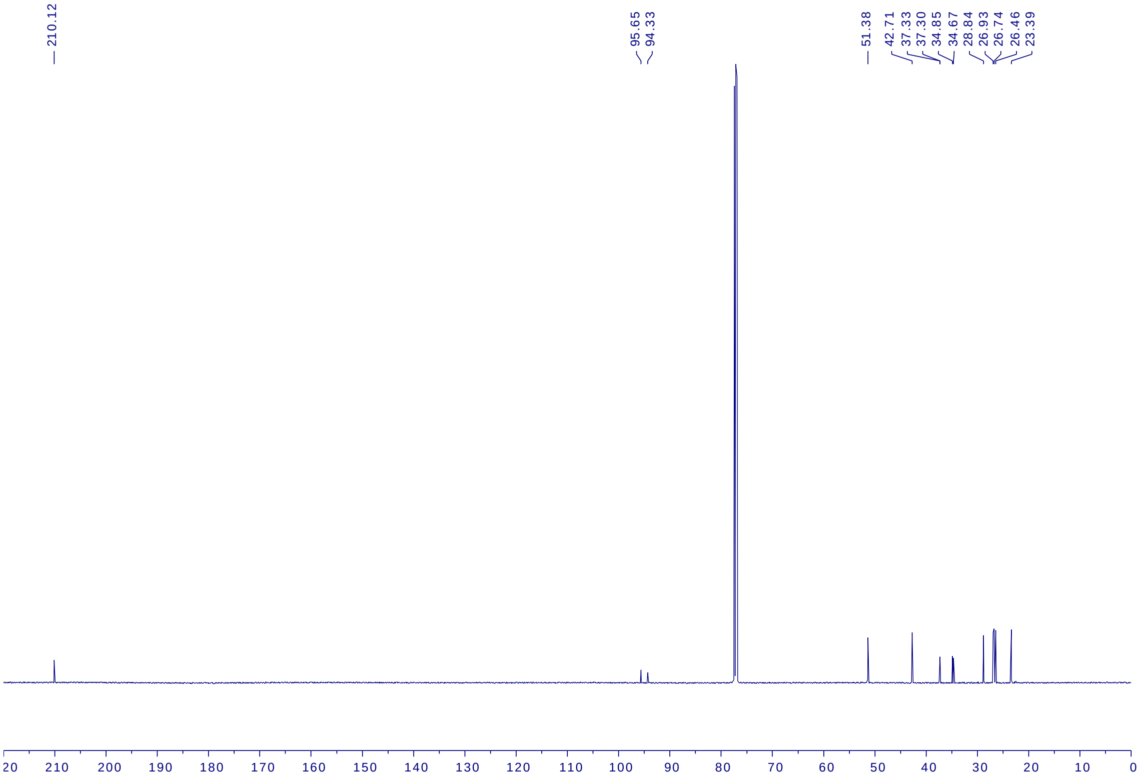

8g ^{19}F NMR (376 MHz, $CDCl_3$)

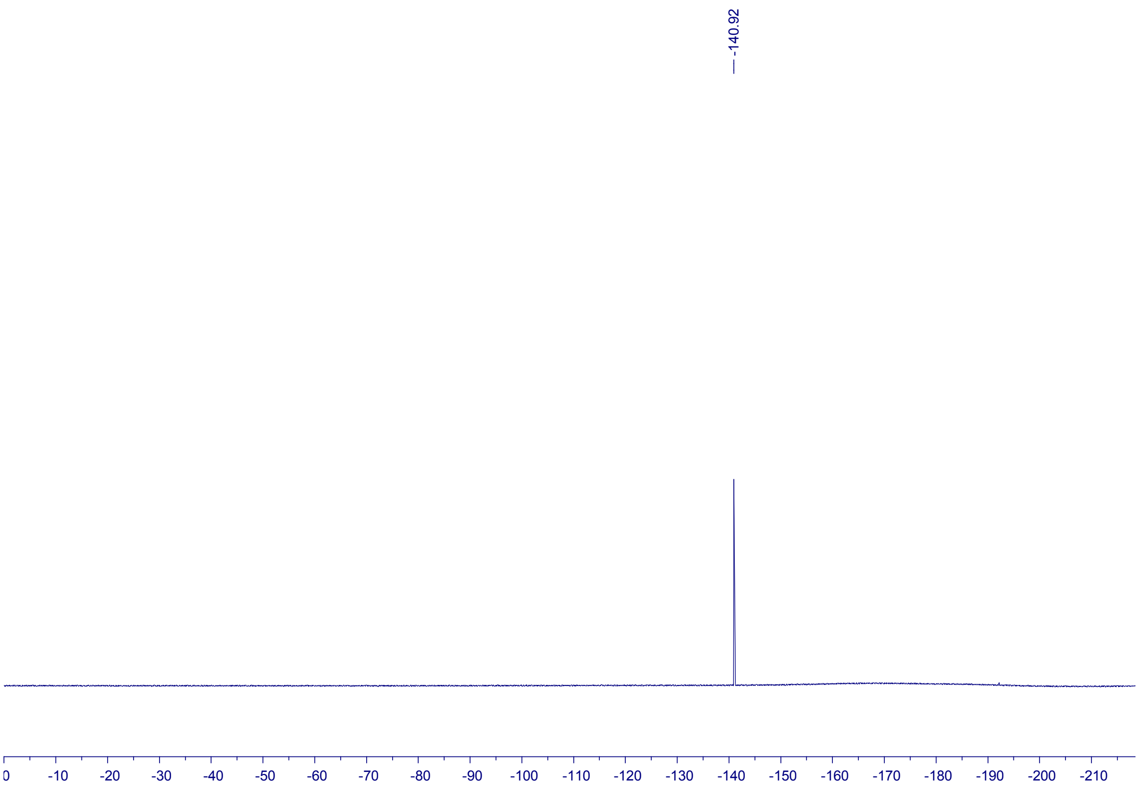

8h ^{1}H NMR (500 MHz, $CDCl_3$)

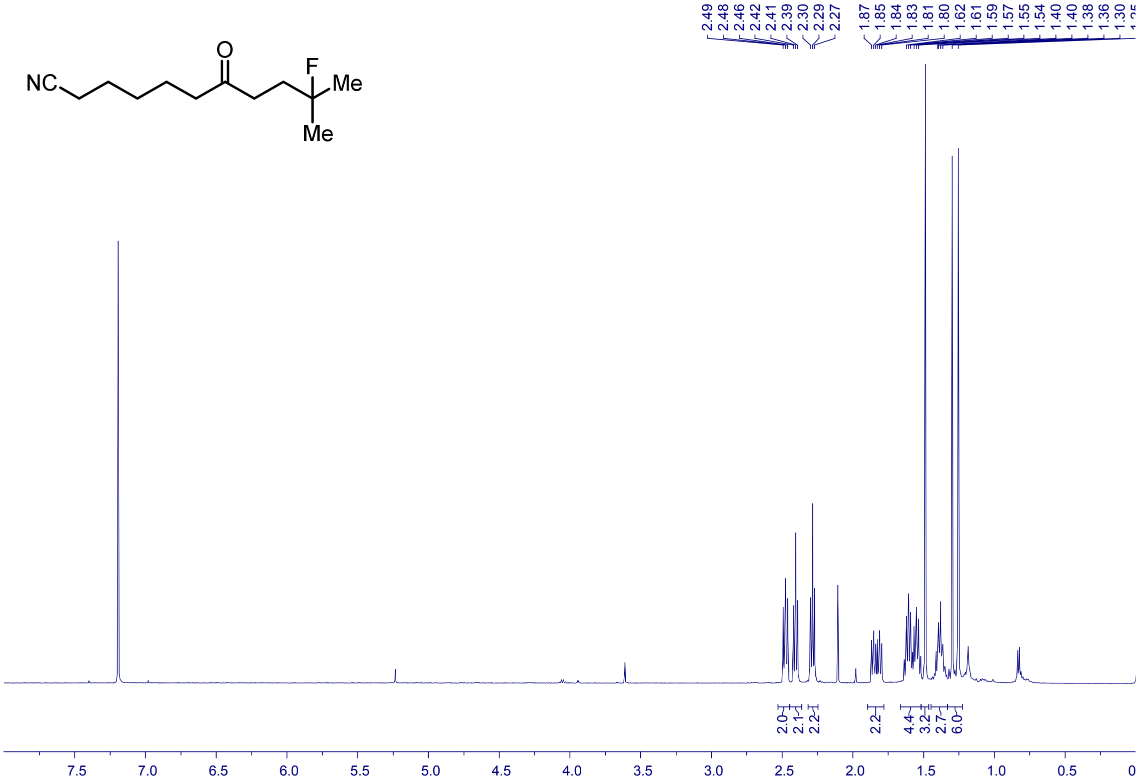

8h ^{13}C NMR (126 MHz, $CDCl_3$)

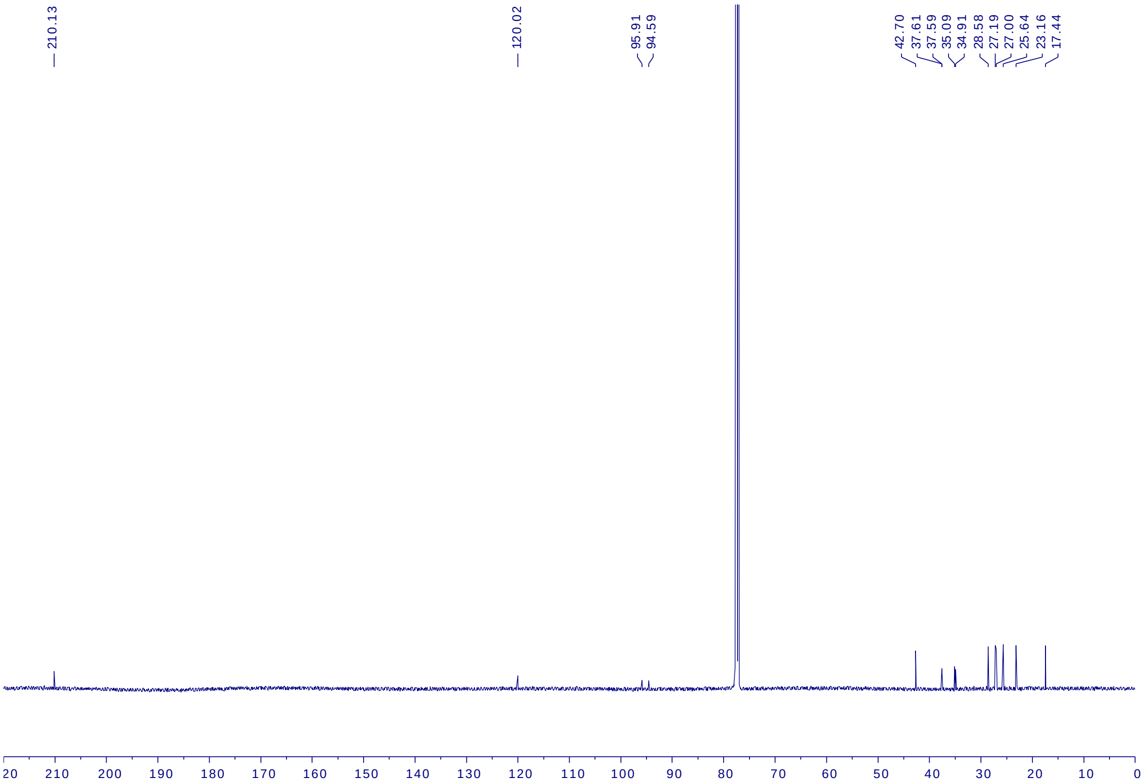

8h ^{19}F NMR (376 MHz, $CDCl_3$)

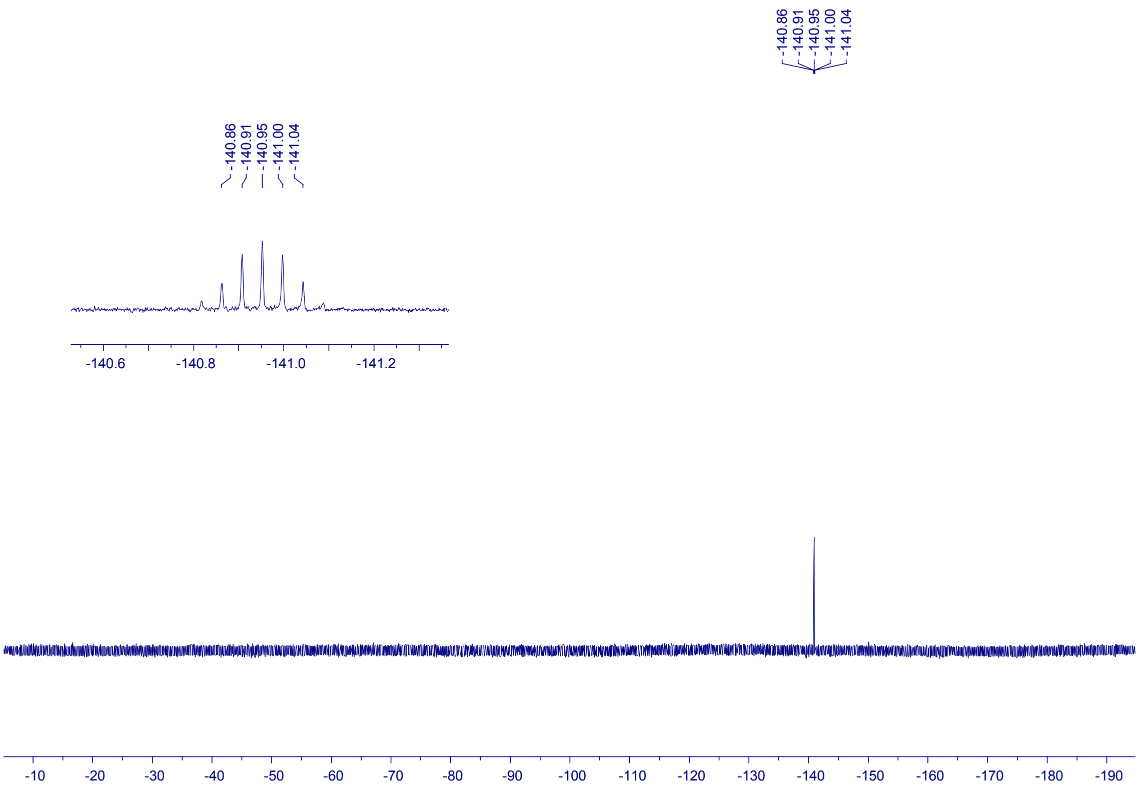

8i ^{1}H NMR (500 MHz, $CDCl_3$)

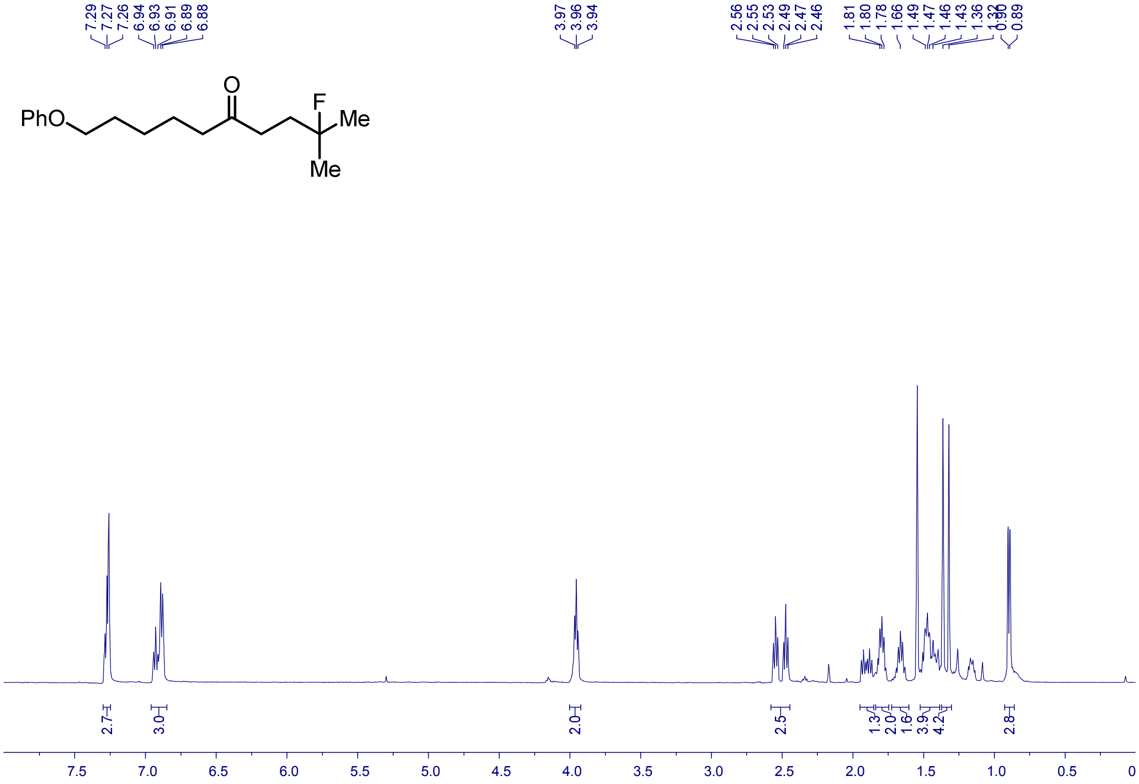

8i ^{13}C NMR (126 MHz, $CDCl_3$)

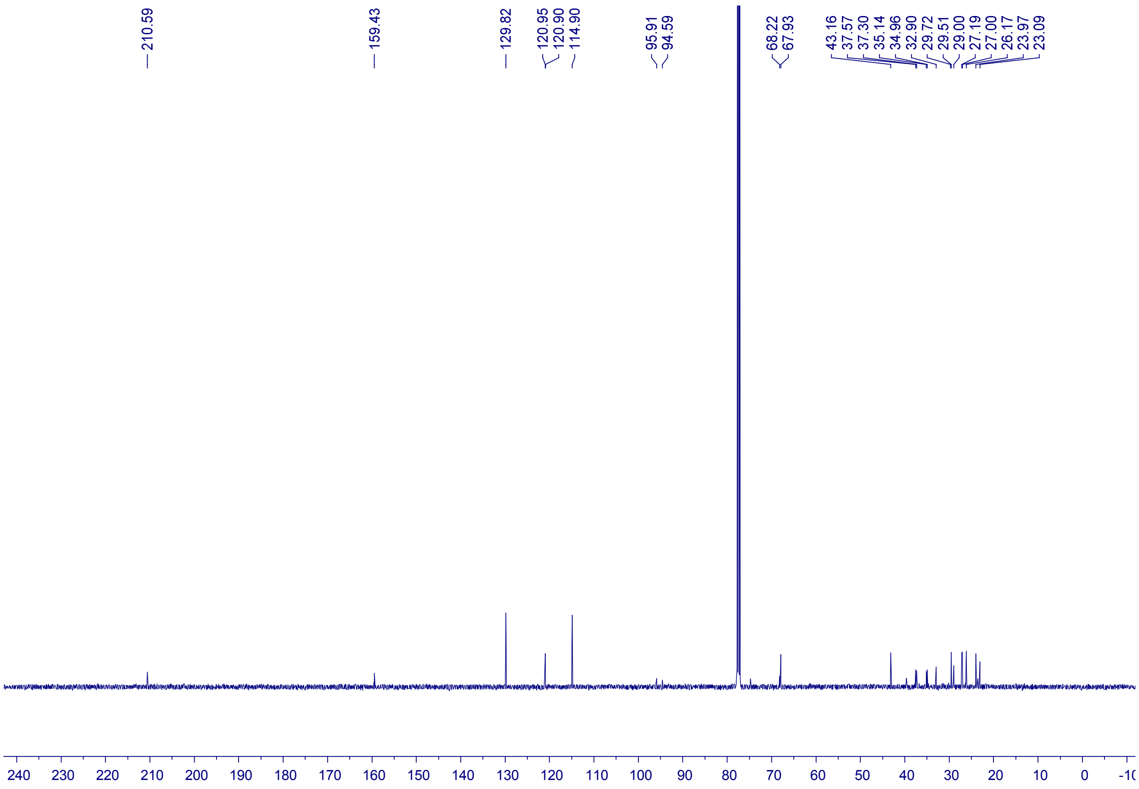

8i ^{19}F NMR (471 MHz, $CDCl_3$)

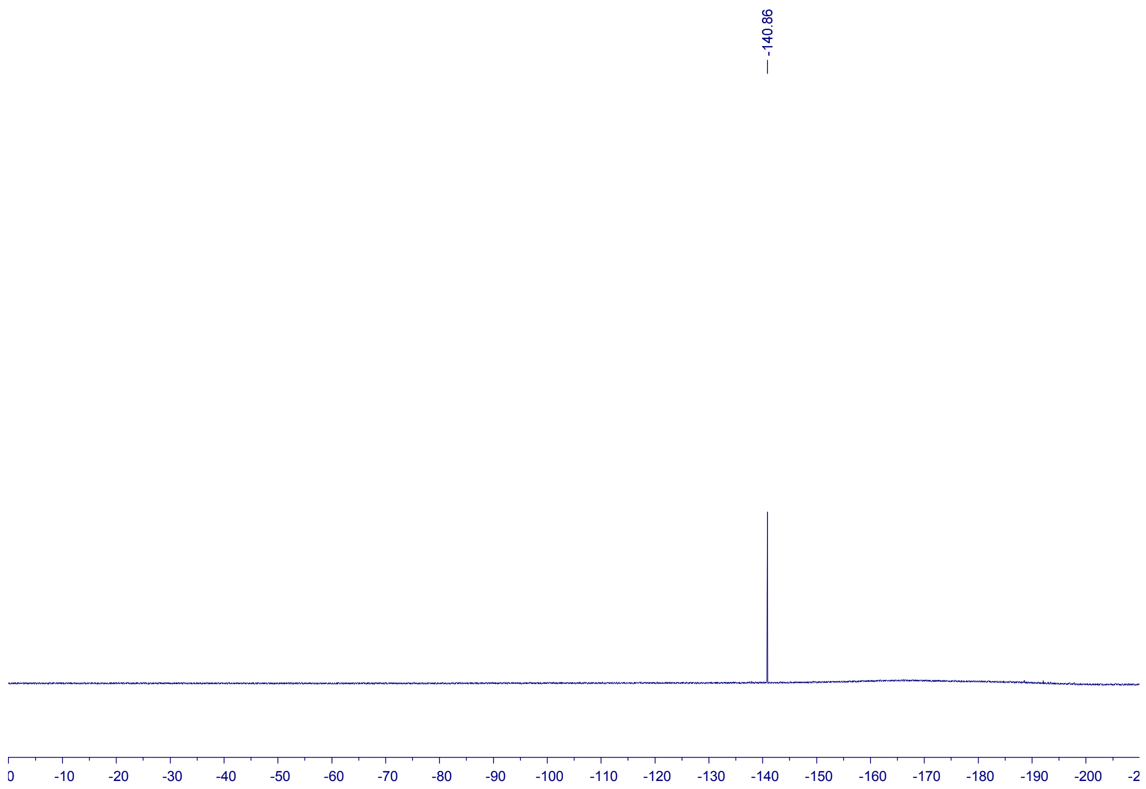

8j ^{1}H NMR (500 MHz, $CDCl_3$)

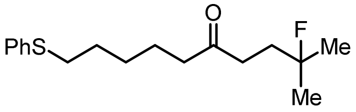

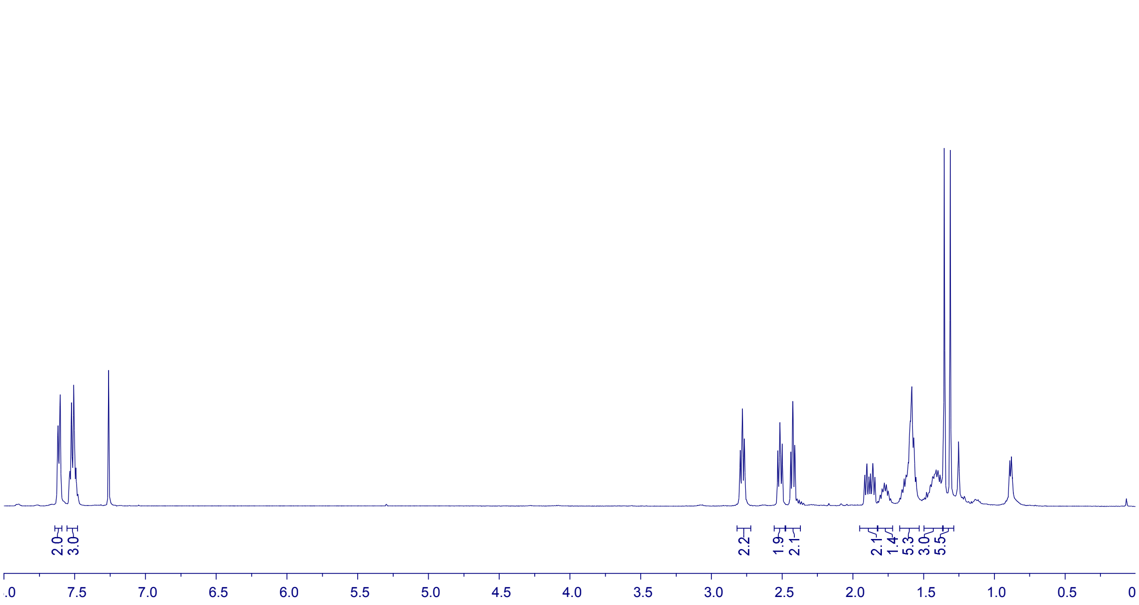

8j ^{13}C NMR (126 MHz, $CDCl_3$)

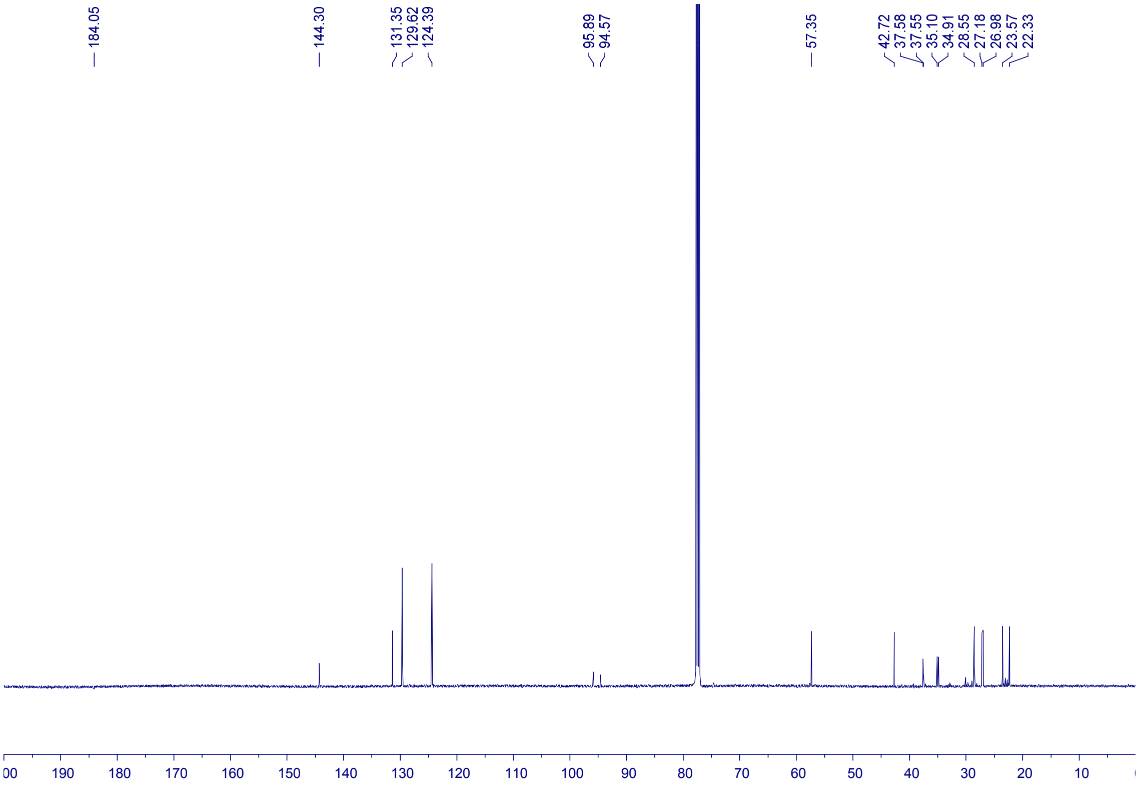

8j ^{19}F NMR (471 MHz, $CDCl_3$)

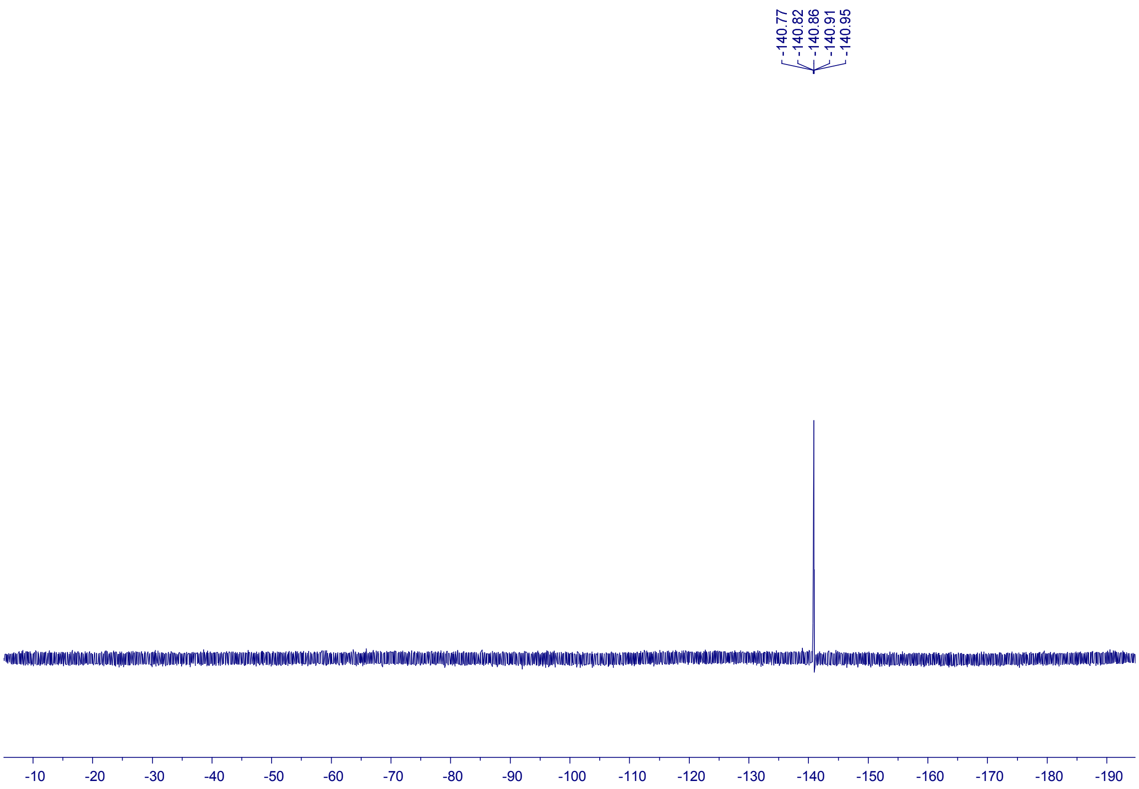

8k ^{1}H NMR (500 MHz, $CDCl_3$)

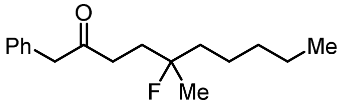

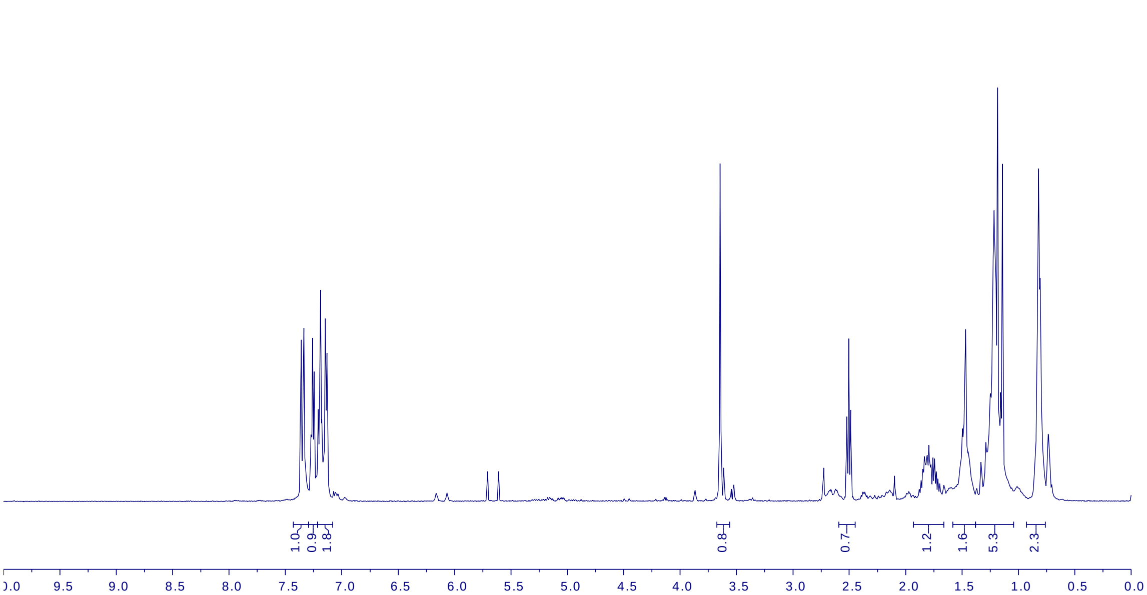

8k ^{13}C NMR (126 MHz, $CDCl_3$)

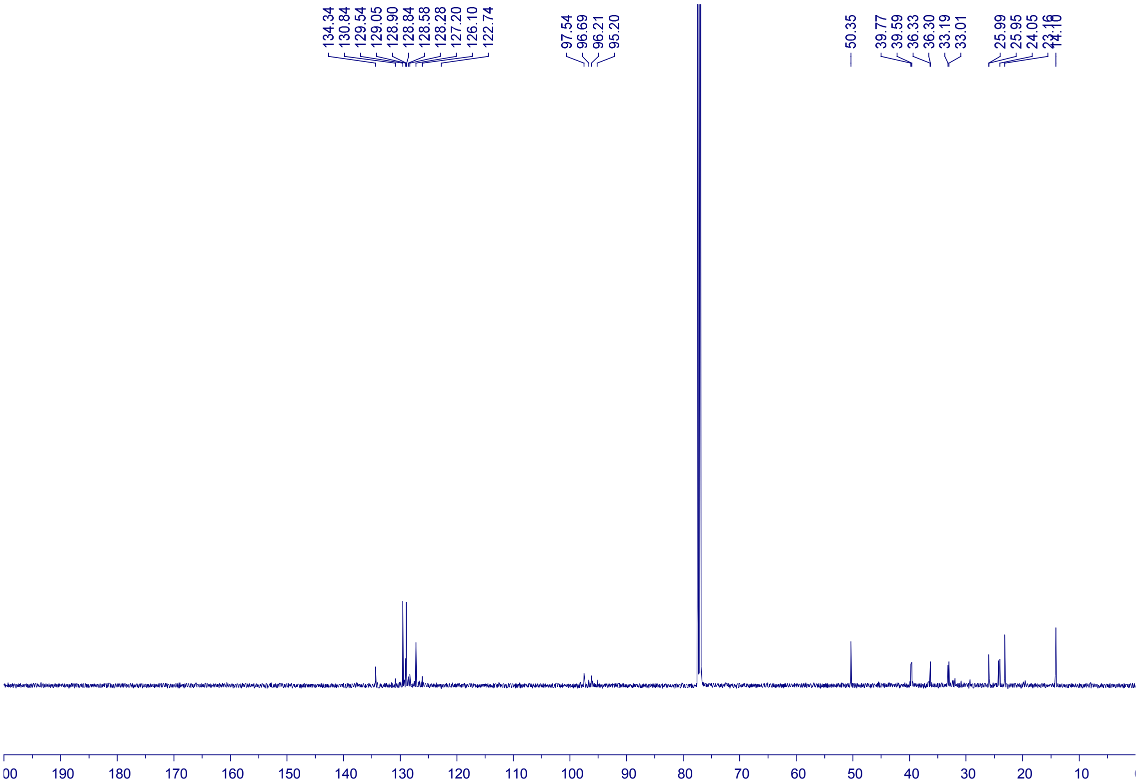

8k ^{19}F NMR (376 MHz, $CDCl_3$)

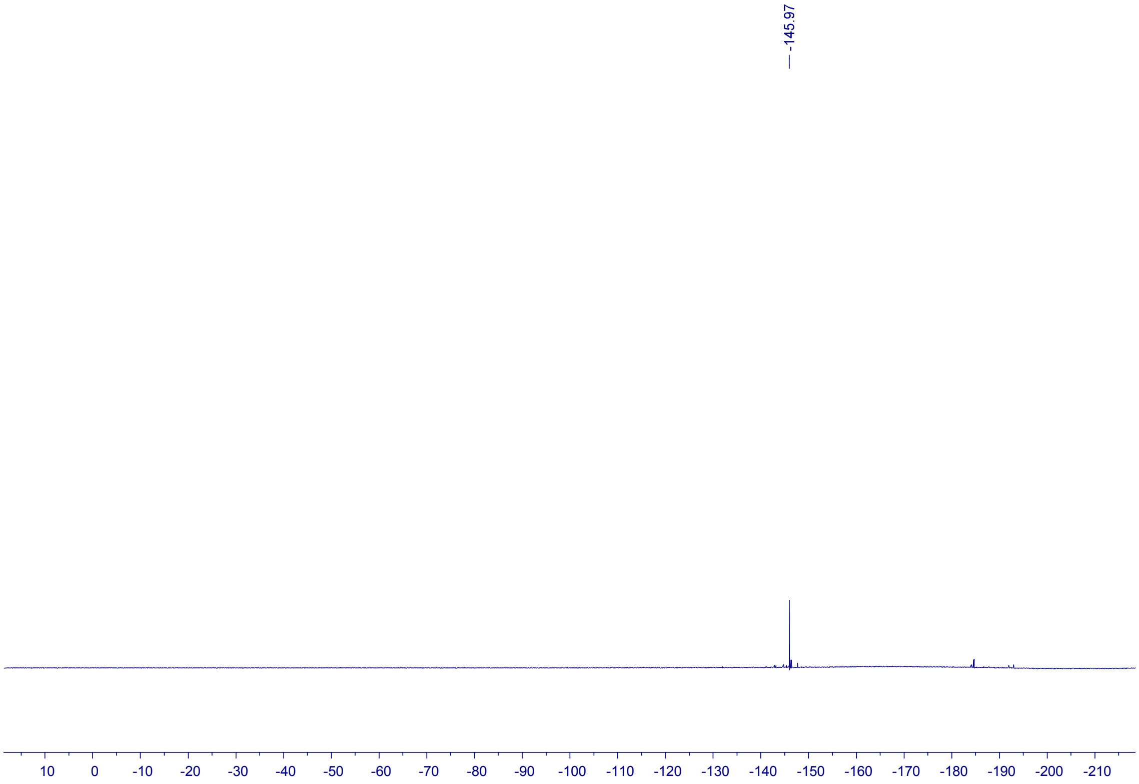

8l ^{1}H NMR (500 MHz, $CDCl_3$)

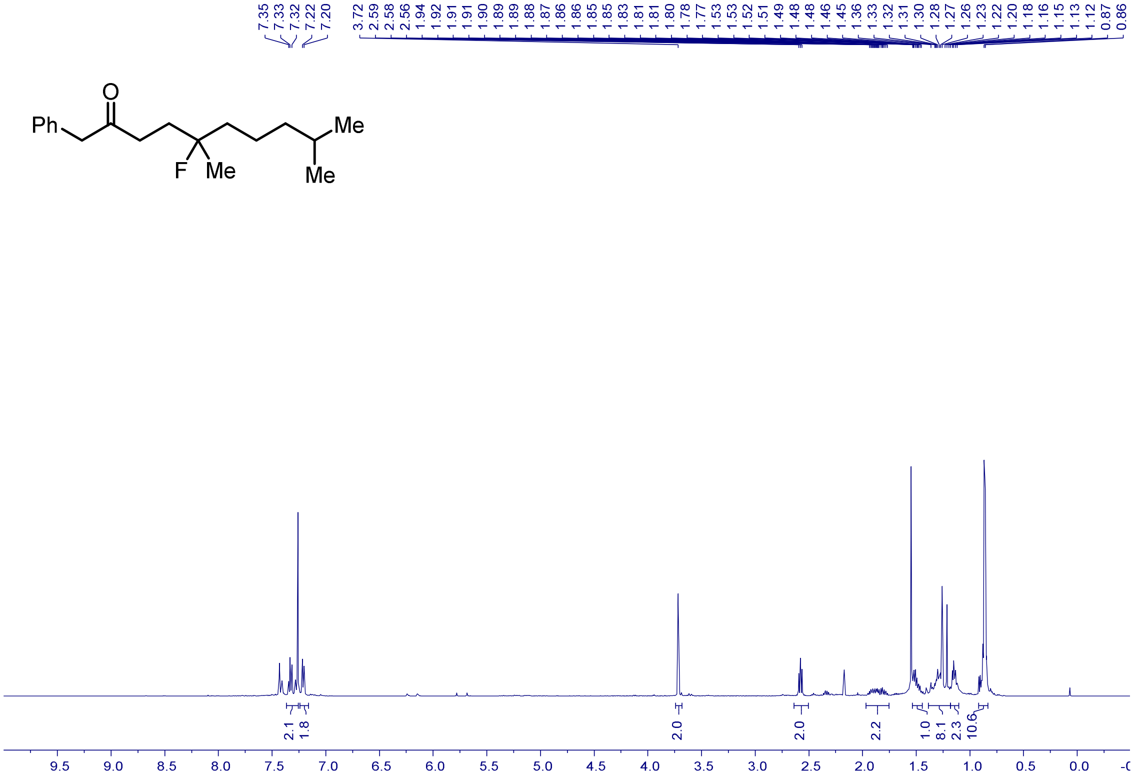

8l ^{13}C NMR (126 MHz, $CDCl_3$)

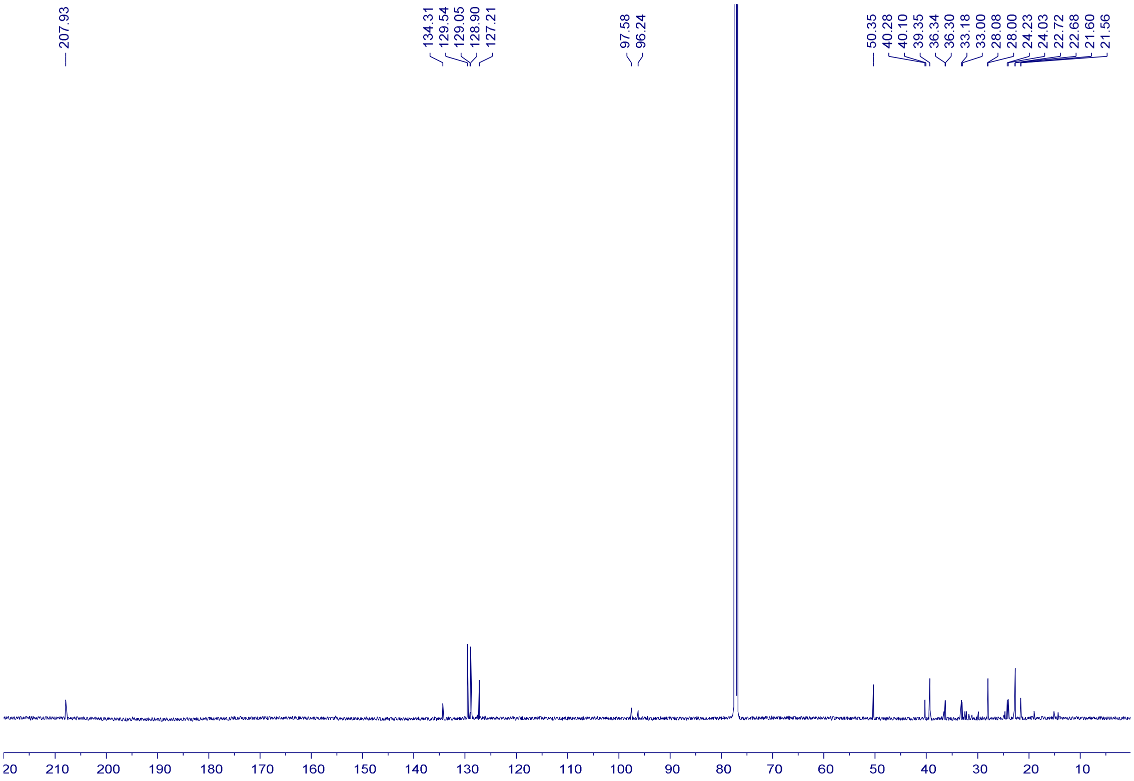

8l ^{19}F NMR (376 MHz, $CDCl_3$)

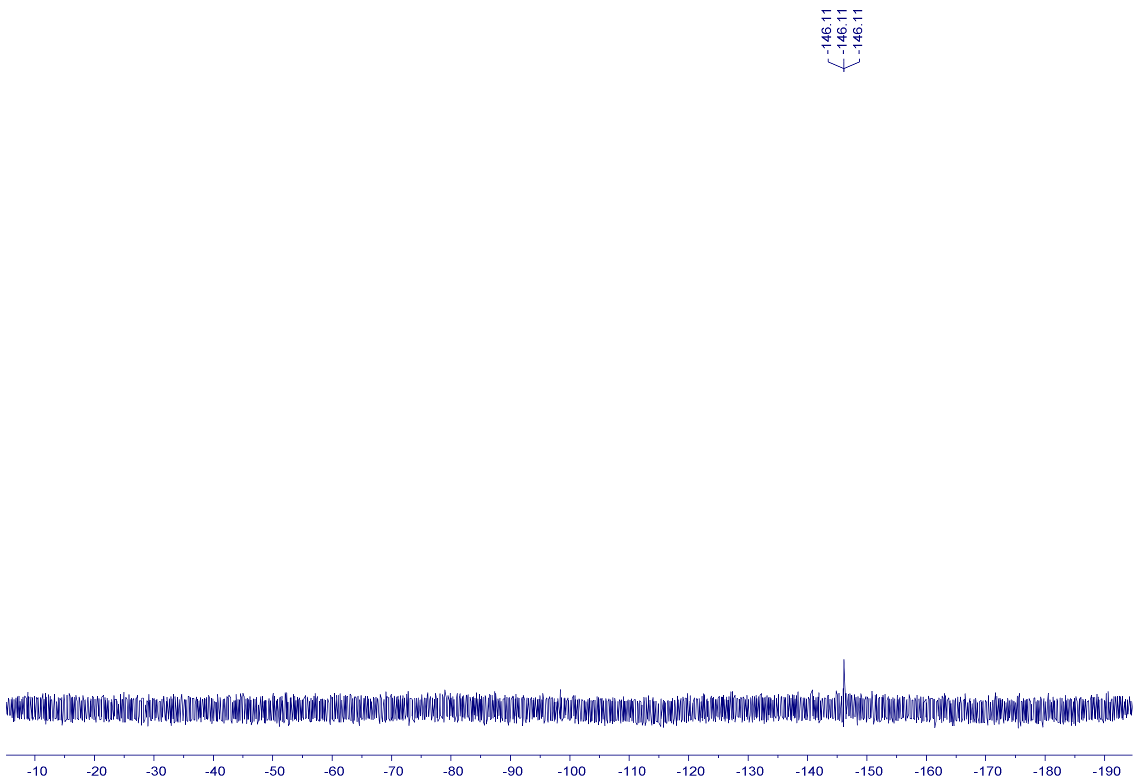

8m ^{1}H NMR (500 MHz, $CDCl_3$)

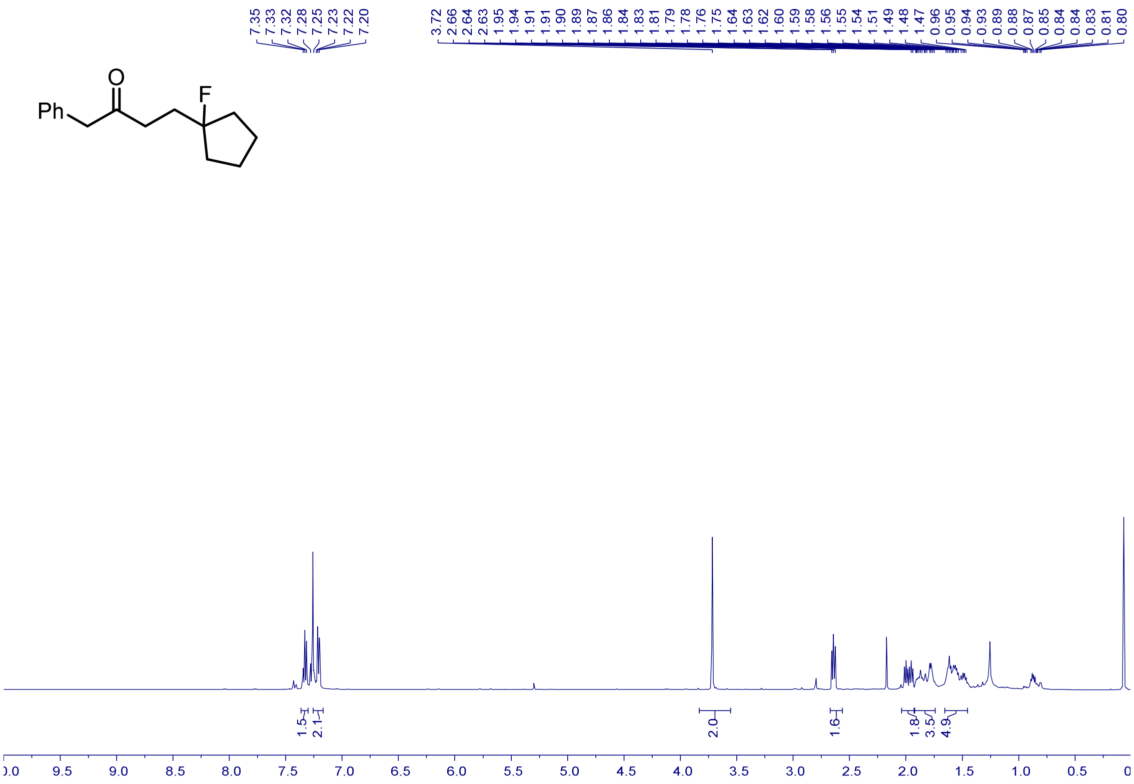

8m ^{13}C NMR (126 MHz, $CDCl_3$)

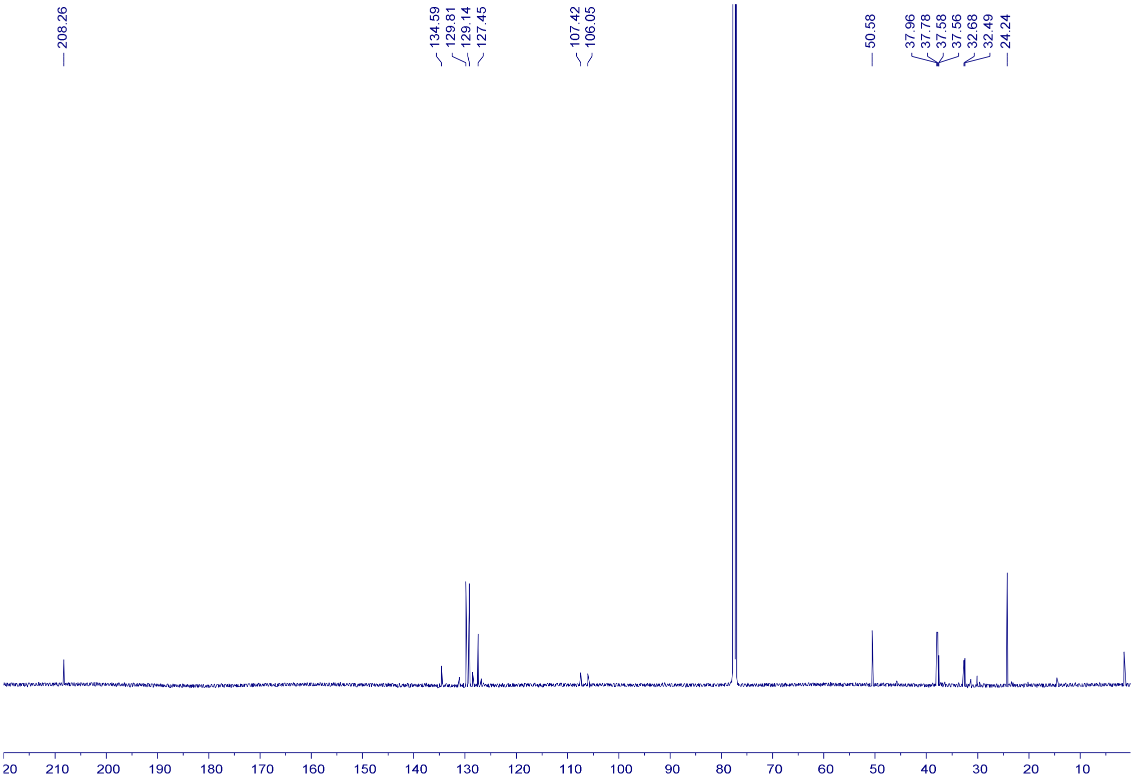

8m ^{19}F NMR (376 MHz, $CDCl_3$)

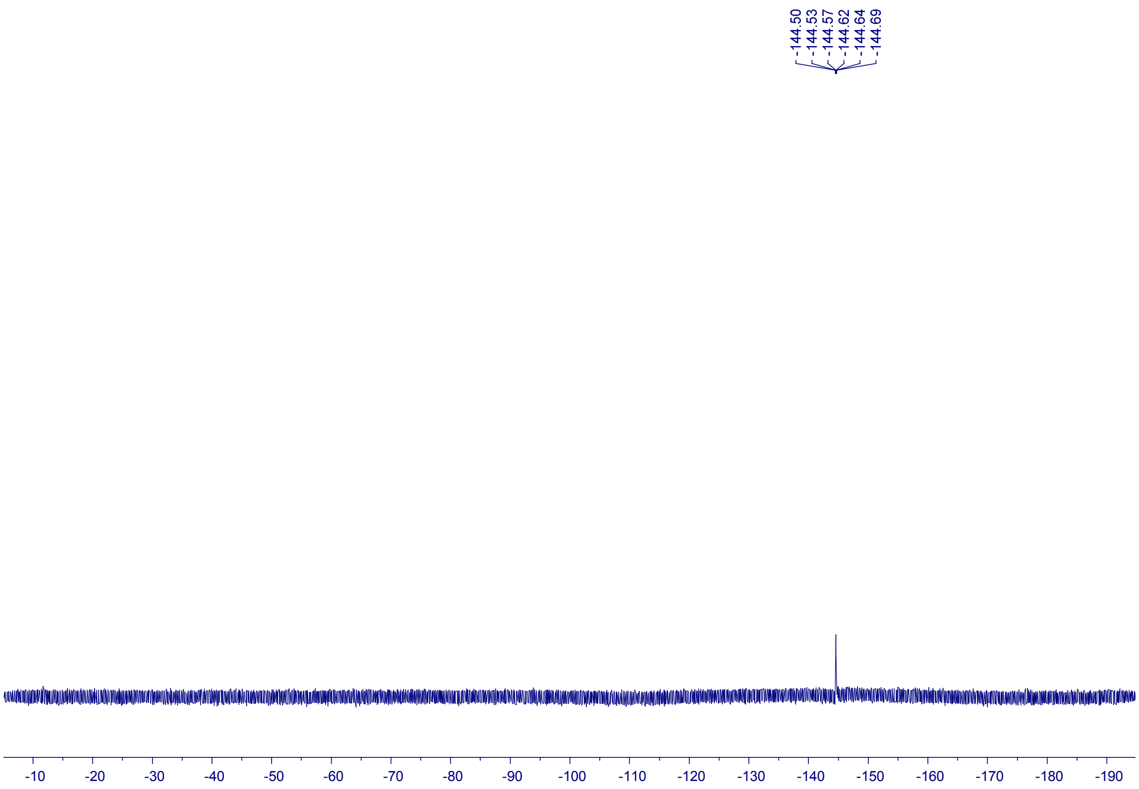

8n ^{1}H NMR (500 MHz, CDCl$_3$)

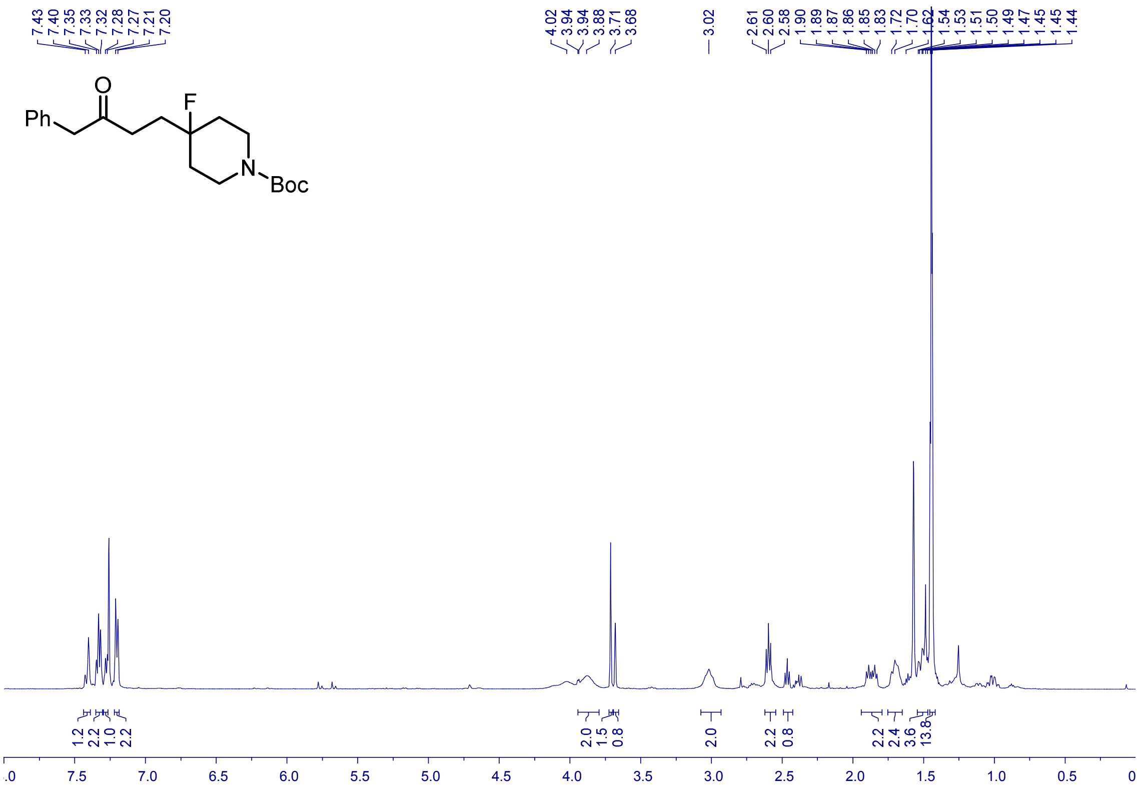

8n ^{13}C NMR (126 MHz, CDCl$_3$)

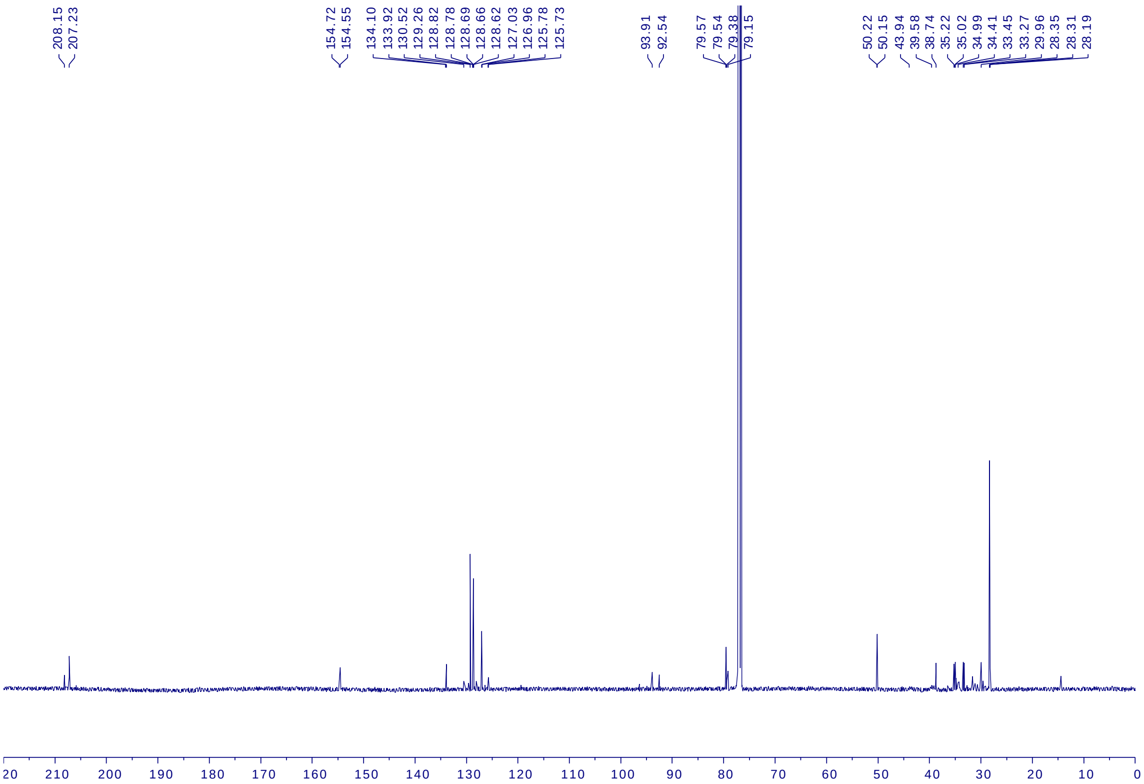

8n ^{19}F NMR (376 MHz, $CDCl_3$)

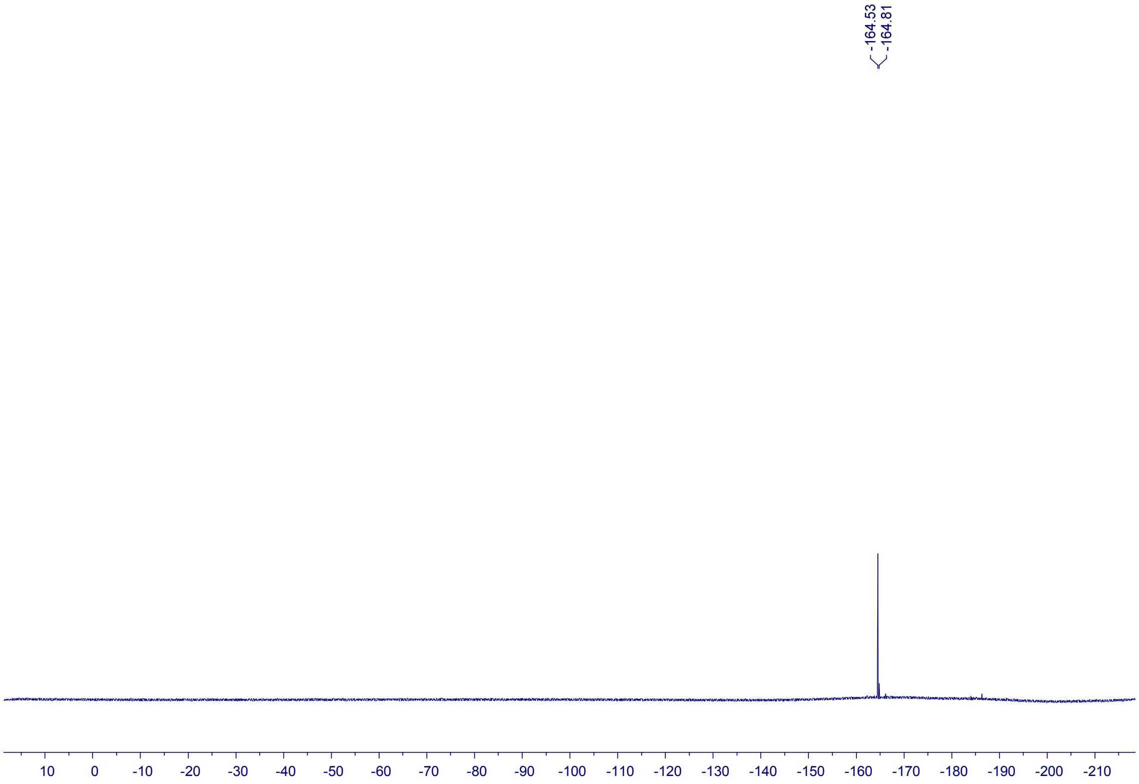

8o ^{1}H NMR (500 MHz, $CDCl_3$)

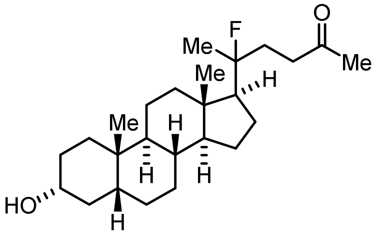

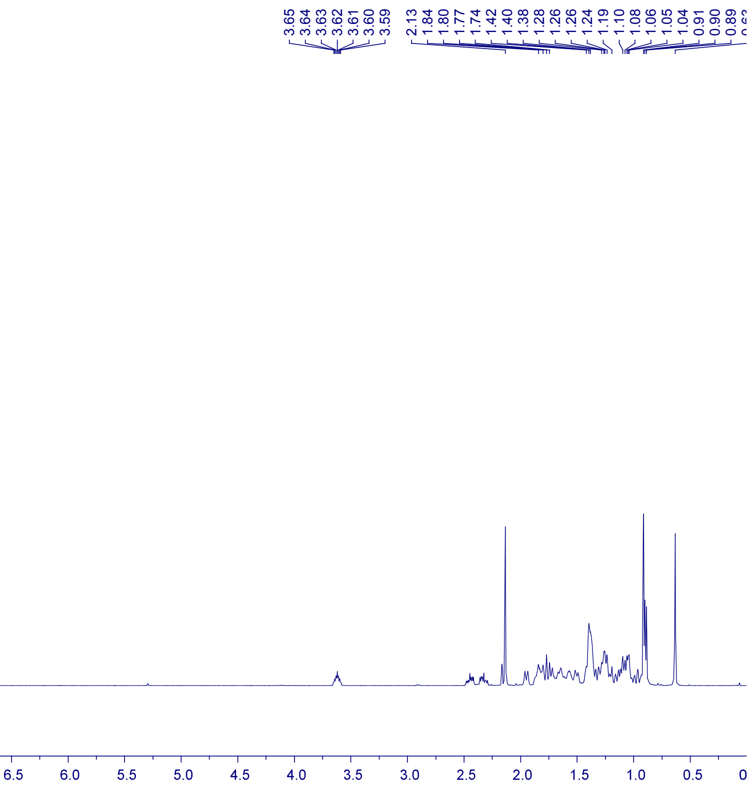

8o ^{13}C NMR (126 MHz, $CDCl_3$)

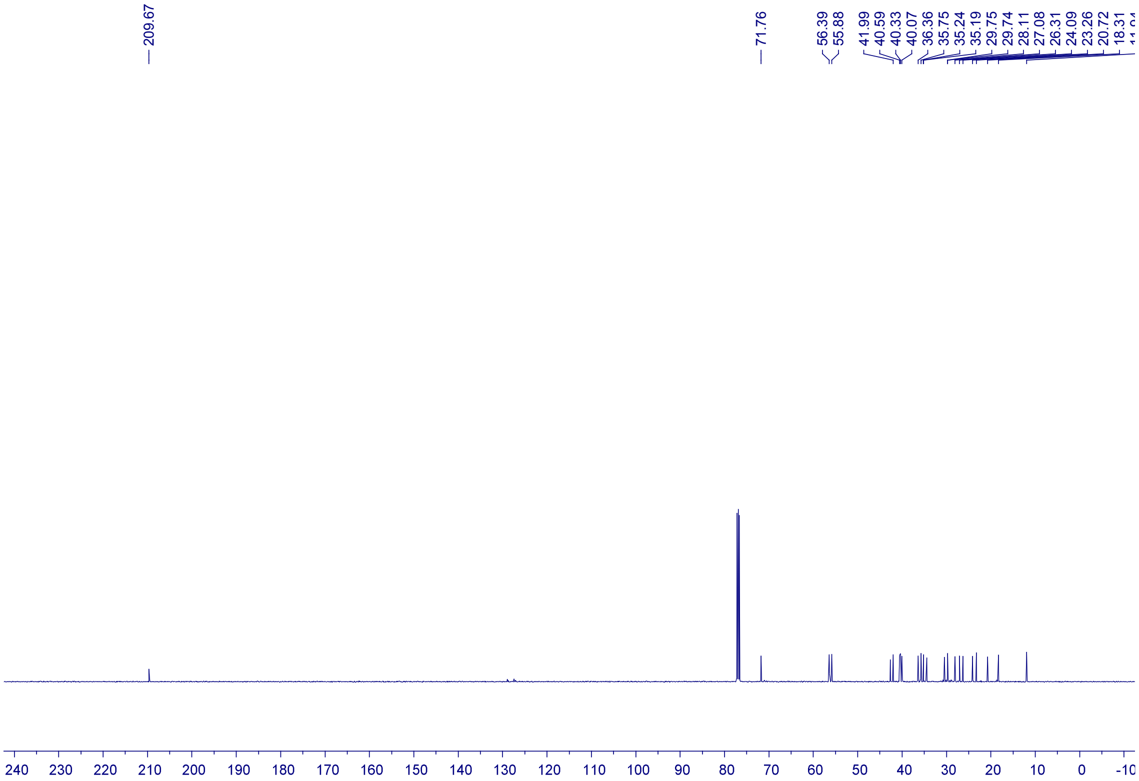

8o ^{19}F NMR (376 MHz, $CDCl_3$)

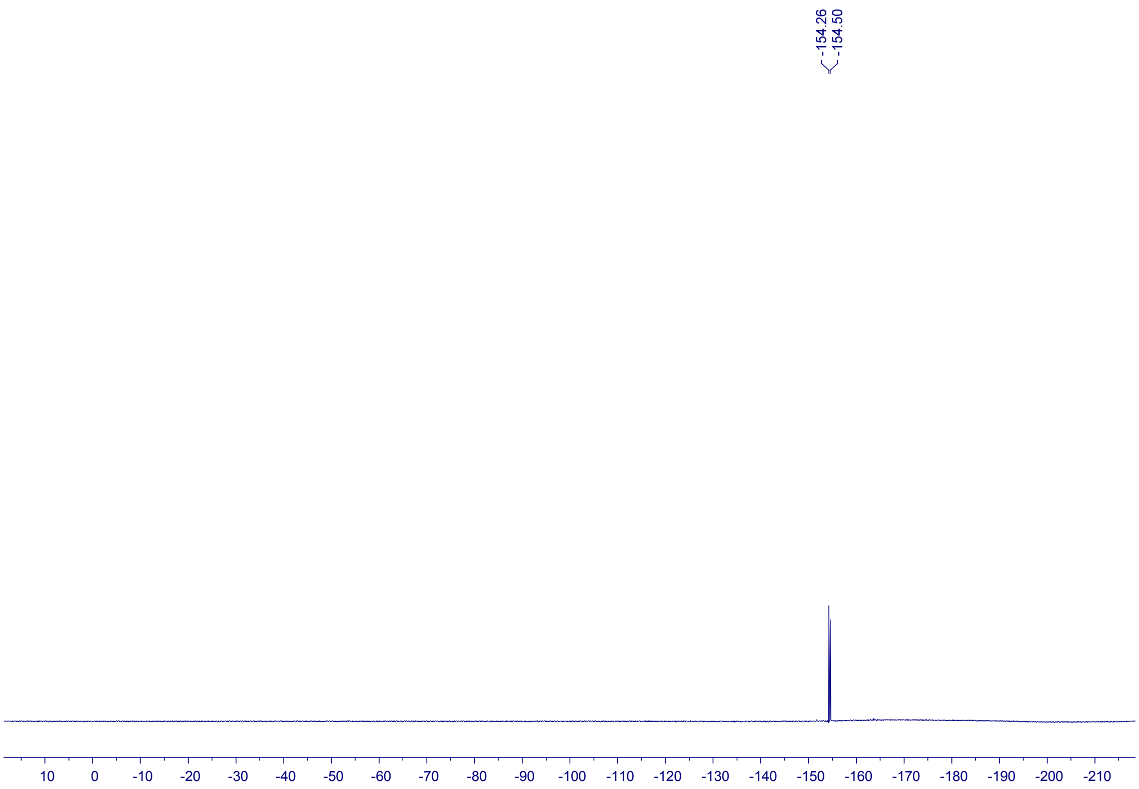

9 References

[1] J. Boivin, E. Fouquet, S. Z. Zard, *Tetrahedron* **1994**, *50*, 1757.

[2] P. Beak, S. T. Kerrick, D. J. Gallagher, *J. Am. Chem. Soc.* **1993**, *115*, 10628.

[3] M. Mousseron, *Bull. Soc. Chim. Fr.* **1957**, 346.

[4] V. Koch, M. Nieger, S. Brase, *Adv. Synth. Catal.* **2017**, *359*, 832.

[5] O. E. Hutt, T. L. Doan, G. I. Georg, *Org. Lett.* **2013**, *15*, 1602.

[6] Y.-F. Liang, X.-F. Zhou, S.-Y. Tang, Y.-B. Huang, Y.-S. Feng, H.-J. Xu, *RSC Adv.* **2013**, *3*, 7739.

[7] S. R. Stauffer, C. J. Coletta, R. Tedesco, G. Nishiguchi, K. Carlson, J. Sun, B. S. Katzenellenbogen, J. A. Katzenellenbogen, *J. Med. Chem.* **2000**, *43*, 4934.

[8] P. J. Wagner, E. J. Siebert, *J. Am. Chem. Soc.* **1981**, *103*, 7329.

[9] F. B. Stocker, M. W. Fordice, J. K. Larson, J. H. Thorstenson, *J. Org. Chem.* **1966**, *31*, 2380.

[10] Q. Dang, Y. Liu, D. K. Cashion, S. R. Kasibhatla, T. Jiang, F. Taplin, J. D. Jacintho, H. Li, Z. Sun, Y. Fan, J. DaRe, F. Tian, W. Li, T. Gibson, R. Lemus, P. D. v. Poelje, S. C. Potter, M. D. Erion, *J. Med. Chem.* **2011**, *54*, 153.

[11] H. C. Brown, M. M. Rogic, H. Nambu, M. W. Rathke, *J. Am. Chem. Soc.* **1969**, *91*, 2147.

[12] E. Larionov, L. Lin, L. Guénée, C. Mazet, *J. Am. Chem. Soc.* **2014**, *136*, 16882.

[13] M. Dukh, P. Drasar, I. Cerny, V. Pouzar, J. A. Shriver, V. Kral, J. Sessler, *Supramol. Chem.* **2010**, *14*, 237.

[14] A. Martin-Mingot, G. Compain, F. Liu, M.-P. Jouannetaud, C. Bachmann, G. Frapper, S. Thibaudeau, *J. Fluorine Chem.* **2012**, *134*, 56.

[15] X. Huang, W. Liu, J. M. Hooker, J. T. Groves, *Angew. Chem. Int. Ed.* **2015**, *54*, 5241.

[16] Y. Amaoka, M. Nagatomo, M. Inoue, *Org. Lett.* **2013**, *15*, 2160.

[17] M. Kirihara, K. Niimi, M. Okumura, T. Momose, *Chem. Pharm. Bull.* **2000**, *48*, 220.

[18] X. Zhang, S. Guo, P. Tang, *Org. Chem. Front.* **2015**, *2*, 806.

[19] M. A. Cismenia, T. P. Yoon, *Chem. Sci.* **2015**, *6*, 5426.

[20] M. Rueda-Becerril, O. Mahé, M. Drouin, M. B. Majewski, J. G. West, M. O. Wolf, G. M. Sammis, J.-F. Paquin, *J. Am. Chem. Soc.* **2014**, *136*, 2637.

[21] (a) H. Zhao, X. Fan, J. Yu, C. Zhu, *J. Am. Chem. Soc.* **2015**, *137*, 3490; (b) Y.-Y. Liu, J. Yang, R.-J. Song, J.-H. Li, *Adv. Synth. Catal.* **2014**, *356*, 2913; (c) Z. Li, L. Song, C. Li, *J. Am. Chem. Soc.* **2013**, *135*, 4640; (d) F. Yin, Z. Wang, Z. Li, C. Li, *J. Am. Chem. Soc.* **2012**, *134*, 10401.

[22] T. Xu, X. Mu, H. Peng, G. Liu, *Angew. Chem. Int. Ed.* **2011**, *50*, 8176.

[23] R. G. Parr, W. Yang, *Density-Functional Theory of Atoms and Molecules* **1989**, Oxford University Press, Oxford U.K.

[24] M. J. Frisch, G. W. Trucks, H. B. Schlegel, G. E. Scuseria, M. A. Robb, J. R. Cheeseman, G. Scalmani, V. BaronE, B. Mennucci, G. A. Petersson, H. Nakatsuji, M. Caricato, X. Li, H. P. Hratchian, A. F. Izmaylov, J. Bloino, G. Zheng, J. L. Sonnenberg, M. Hada, M. Ehara, K. Toyota, R. Fukuda, J. Hasegawa, M. Ishida, T. Nakajima, Y. Honda, O. Kitao, H. Nakai, T. Vreven, J. A. M. Jr., J. E. Peralta, F. Ogliaor, M. Bearpark, J. J. Heyd, E. Brothers, K. N. Kudin, V. N. taroverov, T. Keith, R. Kobayashi, J. Normand, K. Raghavachari, A. Rendell, J. C. Burant, S. S. Iyengar, J. Tomasi, M. CossI, N. Rega, J. M. Millam, M. Klene, J. E. Knox, J. B. CrossI, V. Bakken, C. Adamo, J. Jaramillo, R. Gomperts, R. E. Stratmann, O. Yazyev, A. J. Austin, R. Cammi, C. Pomelli, J. W. Ochterski, R. L. Martin, K. Morokuma, V. G. Zakrzewski, G. A. Voth, P. Salvador, J. J. Dannenberg, S. Dapprich, A. D. Daniels, O. Farkas, J. B. Foresman, J. V. Ortiz, J. Cioslowski, D. J. Fox, *Gaussian 09* **2013**, revision D.01; Gaussian, Inc.

[25] R. Denningtom, T. Keith, J. Millam, *GaussView* **2009**, version 5; Semichem Inc.: Shawnee Mission, KS, 2009.
[26] (a) P. J. Stephens, F. J. Devlin, C. F. Chabalowski, M. J. Frisch, *J. Chem. Phys.* **1994**, *98*, 11623; (b) A. D. Becke, *J. Chem. Phys.* **1993**, *98*, 1372; (c) A. D. Becke, *J. Phys. Chem.* **1993**, *98*, 5648; (d) C. Lee, W. Yang, R. G. Parr, *Phys. Rev. B* **1988**, *37*, 785.
[27] T. D. Svejstrup, W. Zawodny, J. J. Douglas, D. Bidgeli, N. S. Sheikh, D. Leonori, *Chem. Commun.* **2016**, *52*, 12302.
[28] (a) C. Gonzalez, H. B. Schlegel, *J. Phys. Chem.* **1990**, *94*, 5523; (b) C. Gonzalez, H. B. Schlegel, *J. Phys. Chem.* **1989**, *90*, 2154; (c) K. Fukui, *Acc. Chem. Res.* **1981**, *14*, 363.
[29] J. Liu, S. Niwayame, Y. You, K. N. Houk, *J. Org. Chem.* **1998**, *63*, 1064.
[30] (a) G. A. DiLabio, D. A. Pratt, *J. Phys. Chem. A* **2000**, *104*, 1938; (b) G. A. DiLabio, A. D. LoFaro, J. S. Wright, *J. Phys. Chem. A* **1999**, *103*, 1653; (c) A. P. Scot, L. Random, *J. Phys. Chem.* **1996**, *100*, 16502.
